# Enantioconvergent synthesis of chiral fluorenols from racemic secondary alcohols *via* Pd(ii)/chiral norbornene cooperative catalysis†

**DOI:** 10.1039/d4sc01004c

**Published:** 2024-04-25

**Authors:** Bo Ding, Qilin Xue, Han Wei, Jiangwei Chen, Ze-Shui Liu, Hong-Gang Cheng, Hengjiang Cong, Jianting Tang, Qianghui Zhou

**Affiliations:** a Engineering Research Center of Organosilicon Compounds & Materials (Ministry of Education), Hubei Key Lab on Organic and Polymeric OptoElectronic Materials, College of Chemistry and Molecular Sciences, TaiKang Center for Life and Medical Sciences, Wuhan University Wuhan 430072 China qhzhou@whu.edu.cn; b The Institute for Advanced Studies, Wuhan University Wuhan 430072 China; c Key Laboratory of Water Environment Evolution and Pollution Control in Three Gorges Reservoir, School of Environmental and Chemical Engineering, Chongqing Three Gorges University Chongqing 404100 China

## Abstract

An efficient protocol for the asymmetric synthesis of fluorenols has been developed through an enantioconvergent process enabled by Pd(ii)/chiral norbornene cooperative catalysis. This approach allows facile access to diverse functionalized chiral fluorenols with constantly excellent enantioselectivities, applying readily available racemic secondary *ortho*-bromobenzyl alcohols and aryl iodides as the starting materials.

## Abstract 2

An enantioconvergent strategy for the synthesis of chiral fluorenols from racemic secondary alcohols has been disclosed enabled by Pd(ii)/NBE* cooperative catalysis. The reaction has good functional group compatibility and high enantioselectivity.

## Graphical abstract

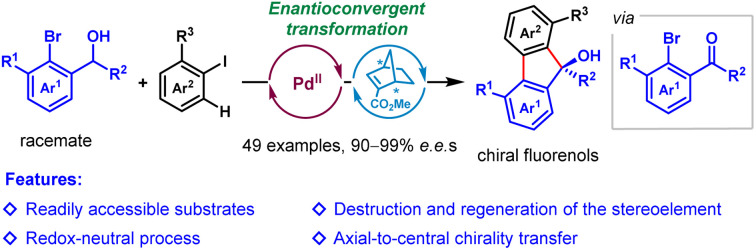

## Introduction

**Fig. 1:**
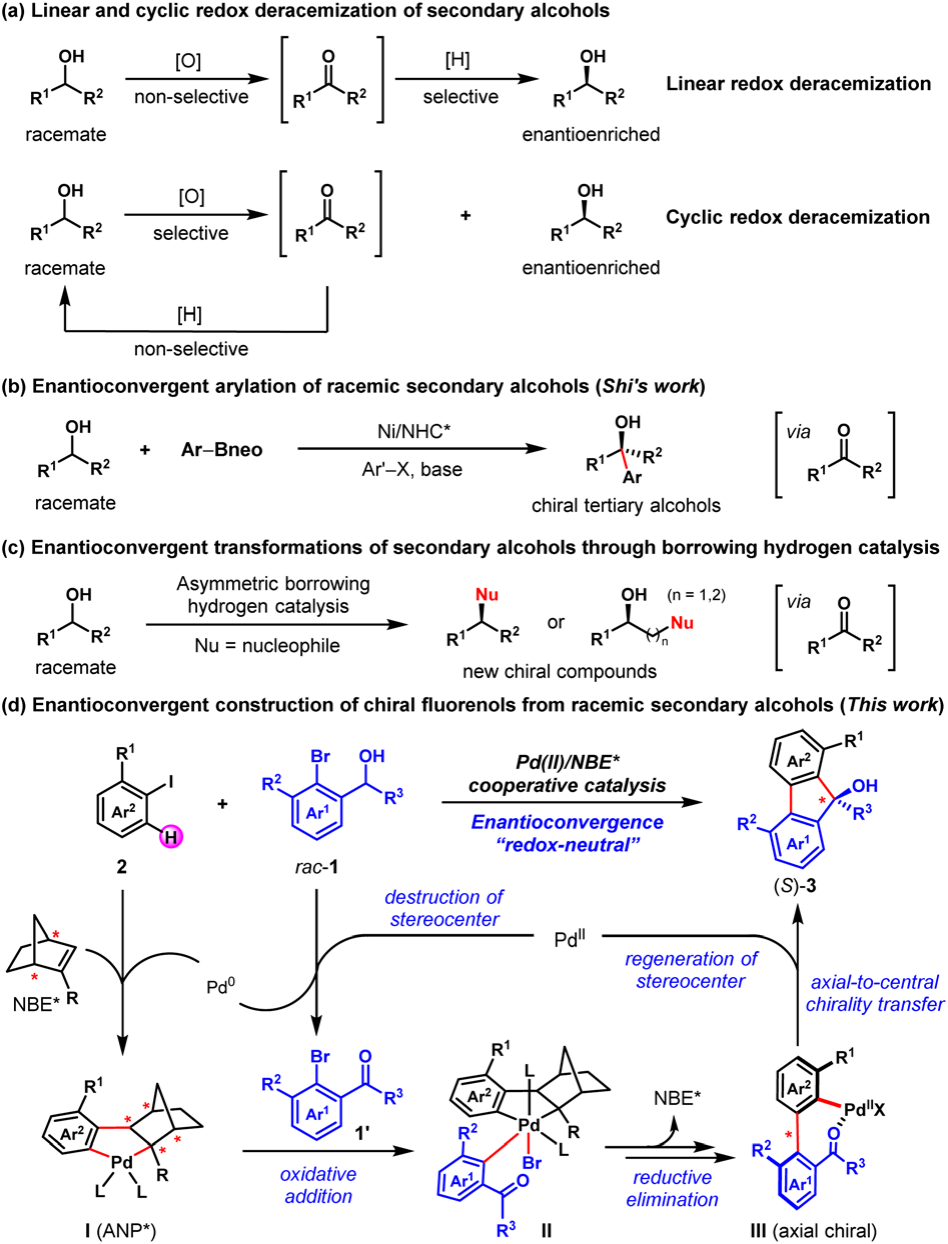
Typical strategies for transforming racemic secondary alcohols into enantioenriched compounds.

Racemic secondary alcohols have been recognized as one of the most useful feedstocks for organic transformations owing to their generally stable, nontoxic, and readily available properties.^1,2^ Therefore, the transformation of racemic secondary alcohols into value-added chiral compounds is an attractive approach in asymmetric synthesis.^1*a*,2–6^ Considerable progress has been made in this area so far, culminating in the development of several elegant strategies, including deracemization,^3,4^ enantioconvergent transformation,^1*a*,2,5^ and kinetic resolution (KR).^6^ For example, two common reaction modes, linear and cyclic redox deracemizations, have been established to prepare enantioenriched secondary alcohols from the corresponding racemates (Fig. 1a).^4*c*–*h*^ In particular, Zuo and co-workers reported a photoinduced deracemization of secondary alcohols enabled by asymmetric ligand-to-metal charge transfer (LMCT) catalysis.^4*i*^ In 2021, Shi and co-workers disclosed the nickel/N-heterocyclic carbene-catalyzed enantioconvergent arylation of racemic secondary alcohols to synthesize enantioenriched tertiary alcohols (Fig. 1b).^2*a*^ Moreover, enantioconvergent transformations of racemic secondary alcohols into enantioenriched chiral higher-order alcohols, amines, N-heterocycles and ketones through asymmetric borrowing hydrogen catalysis have also been developed (Fig. 1c).^1*a*,2*b*,5^

Palladium/norbornene (Pd/NBE) cooperative catalysis, firstly reported by Catellani and co-workers in 1997,^7^ has become a powerful strategy for expeditious synthesis of polysubstituted arenes.^8^ For instance, the synthesis of racemic fluorenols (FOLs) *via* Pd/NBE cooperative catalysis has been successfully developed by Lautens^9*a*^ and Della Ca',^9*b*^ respectively. Asymmetric palladium/chiral norbornene (Pd/NBE*) cooperative catalysis has been theoretically expected as a potential strategy for asymmetric synthesis. However, exploitation of this asymmetric tactic has rarely been initiated for a long time, due to the associated formidable challenges of this complex process.^10^ It is only until recently that remarkable breakthroughs have been achieved in this field, owing to the efforts of Yu,^11^ Dong,^12^ Song,^13^ Liang^14^ and us.^15^

Inspired by these elegant works, we envisioned an asymmetric method to access enantioenriched FOLs through an enantioconvergent process enabled by Pd/NBE* cooperative catalysis, with widely available racemic secondary benzyl alcohols and aryl iodides as the reactants. As depicted in Fig. 1d, this method includes two sequential steps. Firstly, a Pd(ii)-initiated oxidation of the racemic secondary *ortho*-bromobenzyl alcohol (1) will afford the achiral *ortho*-bromoacetophenone (1′) and the corresponding Pd(0) species.^9*b*,16^ Secondly, the Pd(0)/NBE* co-catalyzed asymmetric Catellani-type reaction between the *in situ* generated *ortho*-bromoacetophenone (1′) and aryl iodide (2) will lead to enantioenriched FOL (3) *via* Pd(iv) intermediate (II) and axial chiral Pd(ii) intermediate (III), and meanwhile regenerating the Pd(ii) species^9,15*a*^ (see Fig. S5 in ESI† for more details of the proposed catalytic cycle). The potential features of this protocol include readily available starting materials, redox-neutral process (no external redox reagent is needed), the intriguing asymmetric strategy for destruction/reconstruction of stereoelement and the unique axial-to-central chirality transfer mode. From a practical perspective, the FOL products are known for their biological activity and applications in material.^17^ Until now, extensive efforts have been devoted to the synthesis of racemic FOLs and a number of effective strategies have been developed.^9,18^ However, only three examples of the asymmetric synthesis of FOLs have been reported,^15*a*,19^ to the best of our knowledge. Thus, the development of new strategies for their asymmetric synthesis is highly desirable. Nevertheless, there are three foreseeable main challenges regarding this proposal: (1) the identification of an appropriate catalyst combination of Pd(ii) species, ligand and NBE* to ensure that the oxidation of racemic benzyl alcohol and the Catellani-type reaction connect well to each other and progress in an overall redox-neutral manner; (2) since NBE* is the only chiral source of this complex asymmetric transformation, the employment of a versatile NBE* cocatalyst is pivotal to achieve both good reactivity and enantioselectivity of this method; (3) multiple competitive side reactions, for example, the direct annulation of racemic secondary benzyl alcohol with aryl iodide to afford the benzo[*c*]chromene product,^15*d*,20^ premature *ipso*-protonation of the transient ary-Pd(ii) species (*e.g.*, III)^21^ and *etc.* (see Table 1).

**Table 1:** Optimization of reaction conditions^a^

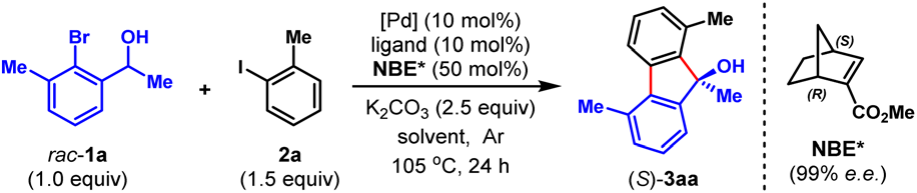					
Entry	[Pd]	Ligand	Solvent	Yield^b^ (%)	E.e*.*^c^ (%)
1	Pd(OAc)_2_	TFP^d^	Toluene	24	98
2	Pd(OAc)_2_	DPEPhos	Toluene	65	—
3	Pd(OAc)_2_	DPPE	Toluene	66	—
4	Pd(OAc)_2_	DPPP	Toluene	78	98
5	Pd(OAc)_2_	DPPPe	Toluene	73	—
6	PdI_2_	DPPP	Toluene	23	—
7	Pd(PPh_3_)_4_	DPPP	Toluene	Trace	—
8^e^	Pd(OAc)_2_	DPPP	Toluene	83	—
9^e^	Pd(OAc)_2_	DPPP	DCE	89	—
10^e^^,^^f^	Pd(OAc)_2_	DPPP	DCE	88	98
11^e^^,^^f^^,^^g^	Pd(OAc)_2_	DPPP	DCE	88 (82)	98
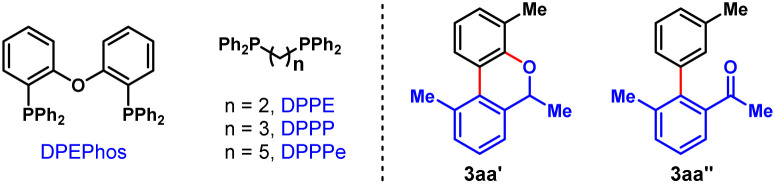					

a All reactions were performed on a 0.1 mmol scale.

b GC yield with biphenyl as an internal standard and isolated yield shown in parentheses.

c Determined by HPLC analysis on a chiral stationary phase.

d 20 mol% TFP.

e 110 °C instead of 105 °C.

f 25 mol% NBE* was applied.

g 1.5 equiv. of K_2_CO_3_ was applied. —: not detected. TFP: tri(2-furyl)phosphine. DCE: 1,2-dichloroethane.

## Results and discussion

To validate our hypothesis, we performed a model reaction using racemic secondary *ortho*-bromobenzyl alcohol (*rac*-1a) and 2-iodotoluene (2a) as the reactants (Table 1). To our delight, under our previously reported reaction conditions (Pd(OAc)_2_ (10 mol%), tri(2-furyl)phosphine (TFP) (20 mol%), (1*R*,4*S*)-2-methyl ester-substituted NBE (NBE*, 99% e.e., 50 mol%) and heating at 105 °C),^15*d*^ the desired chiral FOL 3aa was obtained with excellent enantioselectivity (98% e.e.), albeit in only 24% yield (entry 1). Meanwhile, two main expected side products were isolated, which were the benzo[*c*]chromene 3aa′ (12% isolated yield) formed through intramolecular *ipso*-etherification and the *ipso*-hydrogen-terminated one 3aa′′ (8% isolated yield). Encouraged by these preliminary results, we next focused on optimization of the reaction parameters to increase the reaction efficiency and chemical selectivity. Gratifyingly, the phosphine ligand proved to take a critical role for increasing the yield and chemical selectivity. The unique bidentate phosphine ligands with a flexible backbone,^22^ such as DPEPhos, DPPE, DPPP and DPPPe, are generally superior to monodentate phosphine ligands, to deliver 3aa in significantly improved yields (65–78%, entries 2–5). DPPP is identified as the optimal one (entry 4). Further screening of palladium catalyst, solvent, and reaction temperature indicated that Pd(OAc)_2_, 1,2-dichloroethane (DCE) and 110 °C was the optimal combination (entries 6–9), which provided 3aa in a substantially improved yield (89%, entry 9). It is worth mentioning that the use of DCE can reduce the production of side product 3aa′′. Importantly, the loading of NBE* could be reduced to a truly catalytic amount (25 mol%) without any erosion of the yield and enantiopurity of 3aa (entry 10). Moreover, the amount of K_2_CO_3_ could also be reduced to 1.5 equiv. without any deleterious effects on the reaction (entry 11). Additional optimization of other reaction parameters (*e.g.*, base, concentration and *etc.*) did not improve the efficiency further (see Tables S1–S5 in ESI† for more details). Thus, the optimal reaction conditions were identified to be entry 11 of Table 1, which delivered 3aa in 82% isolated yield and 98% e.e.

With the optimal reaction conditions on hand, we first examined the scope of aryl iodides (2), using *rac*-1a as the reaction partner. As shown in Table 2, a wide variety of *ortho*-substituted aryl iodides were suitable substrates, including sterically hindered isopropyl (3ab), functionalized alkyl (3ac and 3ad), electron-withdrawing trifluoromethyl (3ae), electron donating methoxy and benzyloxy (3af and 3ag), fluoro (3ah) and chloro (3as) groups, and the corresponding products were produced in 46–82% yields. In addition, the substituents at either the 3-, 4- or 5-positions of aryl iodides were well tolerated, which included alkyl (3ai), halogen (3aj, 3ak, 3am, 3an and 3ar), methoxy (3al), amide (3ao), ester (3ap), and nitro (3aq) groups. Moreover, 1-iodonaphtalene derivatives with extended π system were also suitable substrates for this reaction and afforded the benzo[*a*]fluorenol products in good yields (3at and 3au). More importantly, this method could be extended to heteroaryl iodides (2v–x) and 4-iodo-2-quinolone (2y), delivering the enantioenriched heterocyclic FOLs in 50–75% yields (3av–ay). These heterocyclic chiral FOLs may have potential applications in the development of new antimycobacterial and antiprotozoal agents.^17*d*,*g*,18*d*^ Remarkably, all the products (3aa–ay) were obtained with excellent enantioselectivities (90–99% e.e.s), and those compatible functional groups would provide handles for further manipulation of the obtained FOLs.

**Table 2:** Substrate scope of aryl iodides^a^

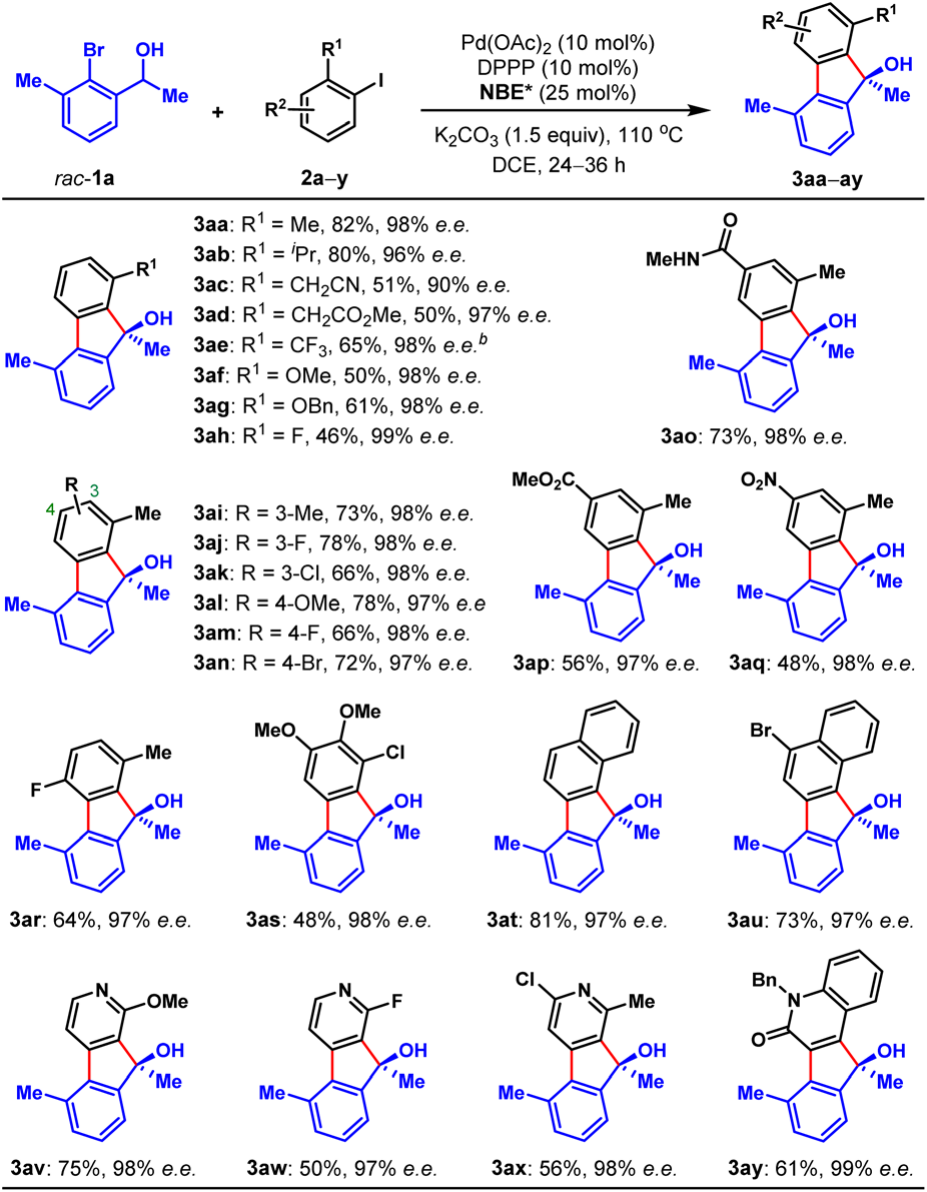

a All reactions were performed on a 0.1 mmol scale, and isolated yields were reported.

b 2.0 equiv. of aryl iodide 2e and 4 Å MS (40 mg) were applied, toluene as the solvent, 36 h.

Next, the scope of racemic secondary *ortho*-bromobenzyl alcohols (1) was investigated, with 2-iodotoluene (2a) as the reaction partner (Table 3). Various alkyl substituents other than methyl group at the *R*^2^ position were applicable for this protocol, including butyl (3ba), branched alkyl (3ca and 3da), and cycloalkyl (3ea and 3fa). It is worth mentioning that the alcohol substrate with a sterically very hindered *tert*-butyl substitution was able to provide the corresponding FOL in 76% yield after extending the reaction time to 36 h (3da). Additionally, the scope of *R*^2^ substitution of racemic alcohols could be extended to a wide range of aryl groups, and the corresponding enantioenriched FOLs (3ga–oa) were obtained in 77–87% yields. For example, besides the simple phenyl group (3ga), other functionalized aryl groups with various substitution including methyl (3ha–ja), electron-withdrawing trifluoromethyl (3la), electron-donating methoxy (3ma), fluoro (3na) and chloro (3oa) groups were tolerated. Notably, secondary benzyl alcohols with a heteroaryl motif were also suitable substrates to deliver corresponding products in moderate yields (3pa and 3qa). Finally, we probed the scope of *ortho*-substituent of the aryl bromide moiety of racemic alcohols, and found sterically hindered isopropyl (3ra), phenyl (3sa), methoxy (3ta and 3xa), chloro (3ua), morpholino-methyl (3wa) and naphthyl (3ya) were well tolerated (3ra–ya), delivering the desired products in good yields (60–89%). In particular, the protocol was applicable for densely functionalized secondary *ortho*-bromobenzyl alcohol, providing polysubstituted chiral FOL 3xa in 64% yield. Similar to Table 2, all the products in Table 3 were obtained with constantly excellent enantioselectivities (93–98% e.e.s). The absolute configuration of 3ha was unambiguously confirmed to be (*S*) by X-ray crystallographic analysis and those of other products were assigned by analogy.

**Table 3:** Substrate scope of racemic brominated benzyl alcohols^a^

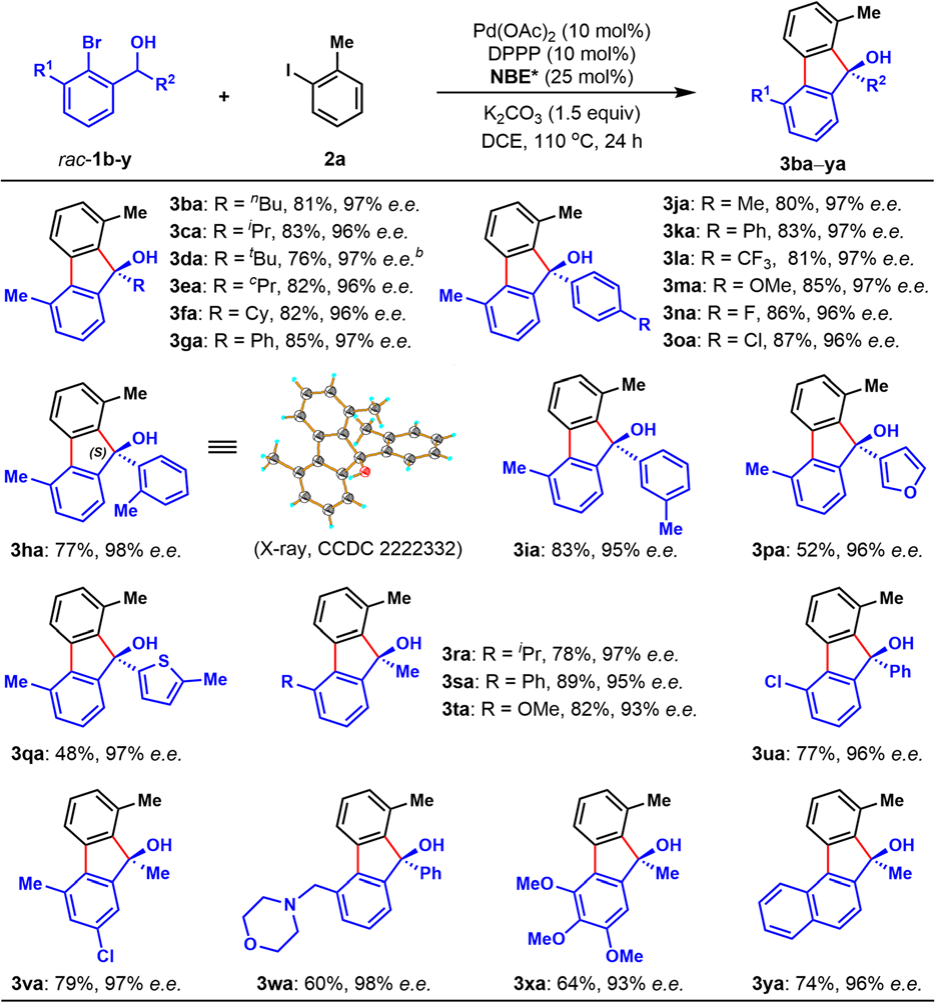

a All reactions were performed on a 0.1 mmol scale, and isolated yields were reported.

b 36 h.

**Scheme 1:**
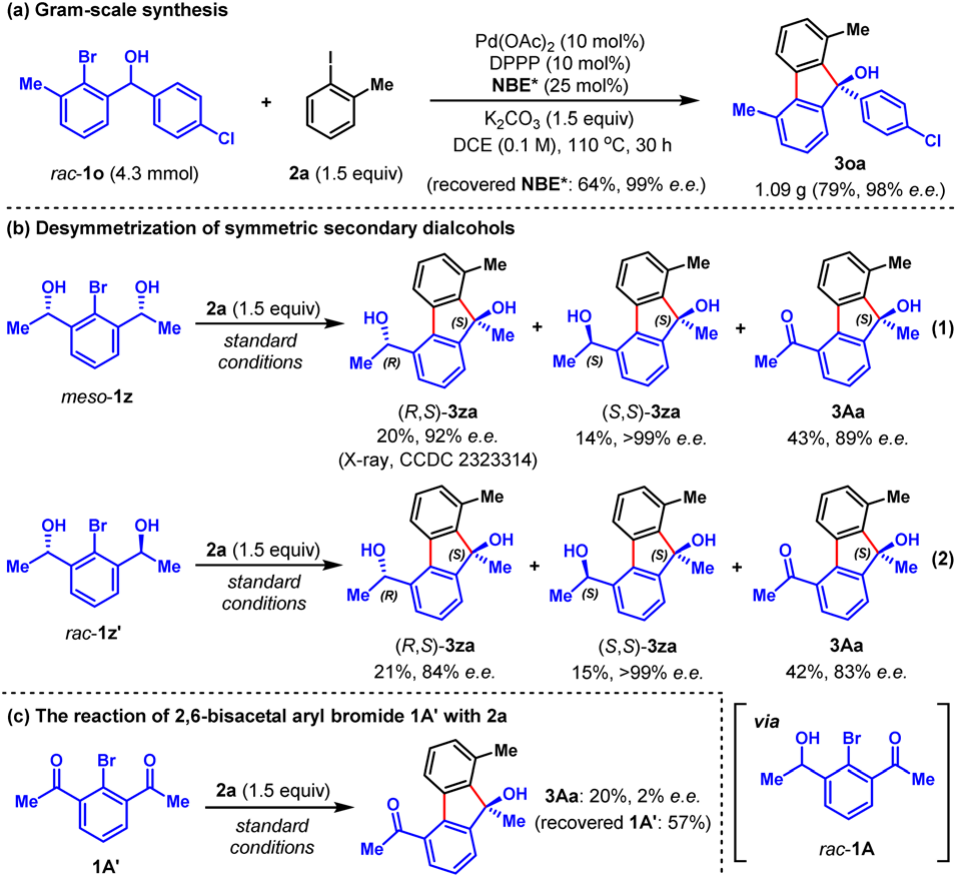
(a) Gram-scale synthesis; (b) desymmetrization of symmetric secondary dialcohols; (c) the reaction of 2,6-bisacetal aryl bromide with 2a.

To demonstrate the synthetic practicality of this method, a larger scale (4.3 mmol) reaction was performed, which proceeded smoothly to give 1.09 gram of the desired product 3oa in 79% yield and 98% e.e. (Scheme 1a), alongside the recovery of 64% of NBE* (99% e.e.) after work-up. Furthermore, based on this method, we developed an intriguing desymmetrization strategy possessing the ability to generate three chiral products in just one operation (Scheme 1b). For instance, by using readily available symmetric secondary dialcohols *meso*-1z as the reactant, the reaction with 2a under standard conditions delivered three separable chiral products (*R*,*S*)-3za (20%, 92% e.e.), (*S*,*S*)-3za (14%, >99% e.e.) and 3Aa (43%, 89% e.e.) (Scheme 1b, eqn (1)). The structure of (*R*,*S*)-3za was unambiguously confirmed by X-ray crystallographic analysis. The ratio of (*R*,*S*)-3za, (*S*,*S*)-3za and 3Aa can be simply tuned by the reaction time (see Fig. S1b in ESI† for details). Similar results were obtained from the reaction of racemic secondary dialcohols *rac*-1z′ with 2a under standard conditions (Scheme 1b, eqn (2)). We reasoned that both *meso*-1z and *rac*-1z′ could be partially oxidized to generate the same intermediate *rac*-1A, which was the real arylating reagent to react with 2a. Interestingly, a control experiment involving the reaction of fully oxidized symmetric diketone intermediate 1A′ with 2a resulted in almost racemic product 3Aa in a poor yield (Scheme 1c), which indicated that product 3Aa of Scheme 1b was mainly formed through *in situ* oxidation of the preformed products (*R*,*S*)-3za and (*S*,*S*)-3za.

**Fig. 2:**
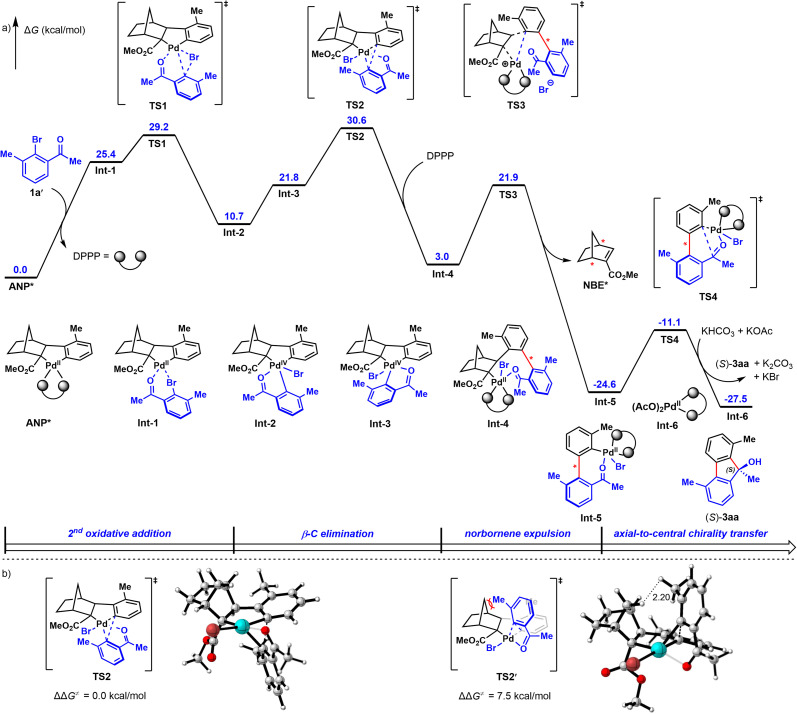
(a) DFT-computed free energy profile for the formations of (*S*)-3aa*via* the computational method of M06L/6-311+G(d,p)-SDD/SMD(DCE)//B3LYP-D3(BJ)/6-31G(d)-LANL2DZ. (b) The optimized structures and relative free energies of the transition states of the stereoselectivity-determining reductive elimination step.

Lastly, to probe the reaction mechanism^9*b*,23^ and the origin of enantioselectivity, density functional theory (DFT) calculations were performed. As revealed in Fig. 2a (the detailed free energy profiles of the generation of both (*S*)- and (*R*)-3aa are included in Fig. S3 and S4†), the reductive elimination step for *ortho*-C–H arylation is the stereoselectivity-determining step of this reaction. The corresponding transition state TS2′ leading to (*R*)-3aa is disfavored because of the steric repulsions between the methyl substituent and the NBE* fragment (Fig. 2b). In contrast, such steric repulsions are absent in the favored transition state TS2, which leads to (*S*)-3aa*via* a stereospecific axial-to-central chirality transfer process. The free energy difference between the two competing reductive elimination processes is calculated to be 7.5 kcal mol^−1^. These DFT calculations are in good agreement with the observed experimental results and the determined absolute configuration of 3ha by X-ray crystallographic analysis. Based on these experimental and calculated results, a possible catalytic cycle for this reaction was proposed in Fig. S5.†

## Conclusions

In summary, we have developed an enantioconvergent strategy for the synthesis of enantioenriched fluorenols from racemic secondary benzyl alcohols and aryl iodides *via* Pd(ii)/chiral norbornene cooperative catalysis. This is an overall redox-neutral transformation involving sequential steps of destroying and regenerating stereoelements. A wide range of readily available aryl iodides and racemic secondary benzyl alcohols are compatible with this protocol, affording a diverse of chiral fluorenols bearing various functional groups with constantly excellent enantioselectivities (49 examples, 90–99% e.e.s). Based on this method, an intriguing desymmetrization strategy possessing the ability to generate three chiral fluorenols in one operation is developed. This work provides a valuable addition to the toolbox of enantioconvergent transformations. Preliminary DFT calculations were carried out to elucidate the reaction mechanism and the origin of enantioselectivity. Lastly, we believe this work will also promote the research and application of chiral fluorenols.

## Data availability

All experimental procedures, characterisation data, mechanistic investigations, NMR spectra and HPLC spectra can be found in the ESI.†

## Author contributions

Q. Z. conceived the idea and directed the project. B. D., Q. X., H. W., J. C., Z.-S. L. and J. T. performed the experiments under the supervision of H.-G. C. and Q. Z. Q. X. performed the density functional theory calculations. H. C. performed the X-ray crystallographic analysis. B. D. and Q. Z. co-wrote the manuscript.

## Conflicts of interest

There are no conflicts to declare.

## Supplementary Material

SC-015-D4SC01004C-s001

SC-015-D4SC01004C-s002
