# Supplementary material for: Enantioconvergent synthesis of chiral fluorenols from racemic secondary alcohols via Pd(ii)/chiral norbornene cooperative catalysis
Source: Chem Sci. 2024 Apr 25;15(21):7975–81. doi: 10.1039/d4sc01004c (PMC11134410; doi:10.1039/d4sc01004c)

Electronic Supplementary Material (ESI) for Chemical Science.
This journal is © The Royal Society of Chemistry 2024

Supporting Information

Enantioconvergent Synthesis of Chiral Fluorenols from Racemic Secondary Alcohols via Pd(II)/Chiral Norbornene Cooperative Catalysis

Bo Ding,[1] Qilin Xue,[1] Han Wei,[2†] Jiangwei Chen,[1†] Ze-Shui Liu,[1] Hong-Gang Cheng,[1] Hengjiang Cong,[1] Jianting Tang[3] and Qianghui Zhou*[1,2]

[1]Engineering Research Center of Organosilicon Compounds & Materials (Ministry of Education), Hubei Key Lab on Organic and Polymeric OptoElectronic Materials, College of Chemistry and Molecular Sciences, and TaiKang Center for Life and Medical Sciences, Wuhan University, Wuhan, 430072, China.

Email: qhzhou@whu.edu.cn

[2]The Institute for Advanced Studies, Wuhan University, Wuhan, 430072, China.

[3]Key Laboratory of Water Environment Evolution and Pollution Control in Three Gorges Reservoir, School of Environmental and Chemical Engineering, Chongqing Three Gorges University, Chongqing, 404100, China

[†]These authors contributed equally: Han Wei and Jiangwei Chen.

Table of Contents

1. General information S2
2. General synthesis procedure of racemic secondary benzyl alcohols S3
3. Optimization of reaction conditions S18
4. General procedure for the synthesis of chiral fluorenols S21
5. Desymmetrization of symmetric secondary dialcohols and the reaction of ketone with aryl iodide S51
6. X-ray crystallographic data S54
7. Computational details and proposed catalytic cycle S56
8. References S112
9. HPLC traces of the products S113
10. NMR Spectra of the new compounds S165

1. General information

All reactions dealing with air- or moisture-sensitive compounds were performed in the argon-filled glove box or by standard Schlenk techniques in oven-dried reaction vessels under argon atmosphere. Anhydrous DMF, CH_3CN, THF, DCM and toluene were dried by JC Meyer Solvent Drying System. Anhydrous (over molecular sieve) DCE, DME, NMP and 1,4-dioxane were purchased from Energy Chemical and used as received. Most reagents were purchased from commercial sources (Energy Chemical, Macklin, Leyan.com, *etc.*) and used without further purification, unless otherwise stated. Reactions were monitored by thin layer chromatography (TLC) carried out on 0.2 mm commercial silica gel plates, using UV light as the visualizing agent or ethanol solution of phosphomolybdic acid (PMA) and heat as a developing agent.

All NMR spectra were recorded on a Bruker spectrometer at 400 MHz (^{1}H NMR), 100 MHz (^{13}C NMR), 376 MHz (^{19}F NMR). For ^{1}H NMR, chemical shifts (δ) were given in ppm using residual undeuterated solvent as internal standard ($CDCl_3$ at 7.26 ppm, DMSO-d_6 at 2.50 ppm, MeOH-d_4 at 3.31 ppm). For ^{13}C NMR, chemical shifts (δ) were reported in ppm using solvent as internal standard ($CDCl_3$ at 77.16 ppm, DMSO-d_6 at 39.52 ppm, MeOH-d_4 at 49.00 ppm). For ^{19}F NMR, chemical shifts (δ) were reported in ppm (no decoupling). The following abbreviations were used to explain multiplicities: s = singlet, d = doublet, t = triplet, q = quartet, dd = doublet of doublets, dt = doublet of triplets, m = multiplet, brs = broad singlet. Coupling constants are reported as a *J* value in Hertz (Hz). The number of protons (*n*) for a given resonance is indicated as *n*H and is based on spectral integration values. Gas chromatography (GC) were recorded on Agilent 7890 instrument with biphenyl as internal standard. High resolution mass spectra (HRMS) were recorded on DIONEX UltiMate 3000 & Bruker Compact TOF mass spectrometer. Optical rotations were measured with an Autopol V Plus/VI digital polarimeter. X-ray structure analyses were performed using a Bruker D8 Venture X-ray single crystal diffractometer. Enantiomeric excesses (*e.e.*) values were determined by chiral HPLC (Agilent 1260) using AD-H, OD-H, IA and IG columns with hexane and ^{i}PrOH as solvents. Melting point (M.P.) were measured with SGW® X-4 micro melting point apparatus. The DFT calculations were performed on the supercomputing system in the Supercomputing Center of Wuhan University.

2. General synthesis procedure of racemic secondary benzyl alcohols

Secondary 2-bromo-3-substitutedbenzyl alcohols **1a**, **1t**, **1x**, **1y**, **1z** and **1z'** were known compounds and others were synthesized as follows.

General procedure I[1]: Synthesis of secondary benzyl alcohols **1a–c**, **1e**, **1g–k**, **1m–o**, **1z** and **1z'**.

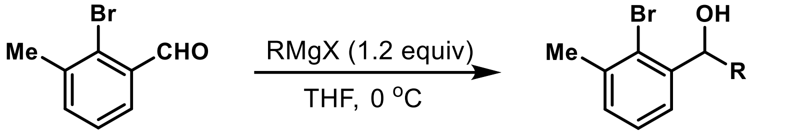

S1 **1a–c, 1e, 1g–k, 1m–o**

In a flame dried Schlenk tube, the required RMgBr or RMgCl (1.2 equiv) was added dropwise to a solution of 2-bromo-3-methylbenzaldehyde (**S1**) (1.0 equiv) in anhydrous THF (0.3 M) at 0 °C under an atmosphere of Ar. The resulting mixture was stirred at 0 °C for 1–4 h. The reaction progress was monitored by TLC. The reaction was quenched with saturated aqueous NH_4Cl, and the organic layer was separated. The aqueous layer was extracted with EtOAc. The combined organic layers were washed with brine, dried over Na_2SO_4, and concentrated under reduced pressure. The residue was purified by silica-gel column chromatography to give the corresponding products **1a–c**, **1e**, **1g–k** and **1m–o**.

1-(2-Bromo-3-methylphenyl)ethan-1-ol (1a)[2]

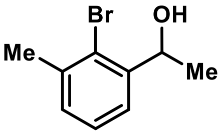

1a

Physical state: white solid;
Yield: 93% (2.01 g, 10 mmol scale);
R_f = 0.3 (PE:EtOAc = 10:1);
^{1}H NMR (400 MHz, $CDCl_3$) δ 7.42 (dd, J = 7.7, 1.7 Hz, 1H), 7.23 (t, J = 7.6 Hz, 1H), 7.16 (dd, J = 7.4, 1.6 Hz, 1H), 5.30 (q, J = 6.4 Hz, 1H), 2.42 (s, 3H), 2.08 (brs, 1H), 1.48 (d, J = 6.4 Hz, 3H);
^{13}C NMR (100 MHz, $CDCl_3$) δ 145.2, 138.4, 129.8, 127.5, 124.5, 124.0, 69.7, 23.9, 23.6.

1-(2-Bromo-3-methylphenyl)pentan-1-ol (1b)

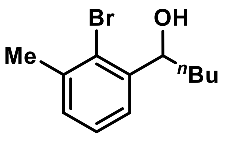

1b

Physical state: white solid;

Yield: 56% (144.6 mg, 1.0 mmol scale);

$\boldsymbol{R_f}$ = 0.3 (PE:EtOAc = 10:1);

M.P.: 36 – 38 ºC;

^{1}H NMR (400 MHz, $CDCl_3$) δ 7.38 (dd, J = 7.7, 1.7 Hz, 1H), 7.23 (t, J = 7.5 Hz, 1H), 7.15 (dd, J = 7.5, 1.7 Hz, 1H), 5.14 (dd, J = 8.3, 4.0 Hz, 1H), 2.42 (s, 3H), 1.99 (brs, 1H), 1.85 – 1.74 (m, 1H), 1.71 – 1.62 (m, 1H), 1.55 – 1.44 (m, 1H), 1.44 – 1.31 (m, 3H), 0.92 (t, J = 7.0 Hz, 3H);

^{13}C NMR (100 MHz, $CDCl_3$) δ 144.5, 138.4, 129.8, 127.3, 124.8, 124.7, 73.5, 37.5, 28.2, 24.0, 22.7, 14.2;

HRMS (ESI-TOF) calculated for $C_{12}H_{17}BrO$, $[M+Na]^+$ 279.0355, found 279.0352.

1-(2-Bromo-3-methylphenyl)-2-methylpropan-1-ol (1c)

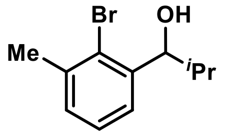

1c

Physical state: white solid;

Yield: 36% (86.8 mg, 1.0 mmol scale);

$\boldsymbol{R_f}$ = 0.3 (PE:EtOAc = 10:1);

M.P.: 33 – 35 ºC;

^{1}H NMR (400 MHz, $CDCl_3$) δ 7.32 (dd, J = 7.7, 1.8 Hz, 1H), 7.22 (t, J = 7.5 Hz, 1H), 7.15 (dd, J = 7.5, 1.8 Hz, 1H), 4.95 (d, J = 5.5 Hz, 1H), 2.43 (s, 3H), 2.13 – 2.02 (m, 1H), 1.94 (brs, 1H), 0.96 (dd, J = 6.8, 4.2 Hz, 6H);

^{13}C NMR (100 MHz, $CDCl_3$) δ 143.4, 138.4, 129.7, 127.0, 125.7, 125.4, 78.0, 33.8, 24.1, 19.8, 16.8;

HRMS (ESI-TOF) calculated for $C_{11}H_{15}BrO$, $[M+Na]^+$ 265.0198, found 265.0200.

(2-Bromo-3-methylphenyl)(cyclopropyl)methanol (1e)

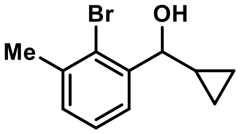

1e

Physical state: pale yellow solid;

Yield: 40% (143.0 mg, 1.5 mmol scale);

Rf = 0.25 (PE:EtOAc = 10:1);

M.P.: 65 – 67 ºC;

^{1}H NMR (400 MHz, $CDCl_3$) δ 7.45 (dd, *J* = 7.6, 1.8 Hz, 1H), 7.24 (t, *J* = 7.6 Hz, 1H), 7.18 (dd, *J* = 7.5, 1.3 Hz, 1H), 4.73 (d, *J* = 7.5 Hz, 1H), 2.43 (s, 3H), 2.10 (s, 1H), 1.35 – 1.25 (m, 1H), 0.65 – 0.58 (m, 1H), 0.56 – 0.44 (m, 3H);

^{13}C NMR (100 MHz, $CDCl_3$) δ 143.1, 138.5, 130.1, 127.3, 125.6, 125.5, 76.0, 24.1, 17.8, 3.5, 2.3;

HRMS (ESI-TOF) calculated for $C_{11}H_{13}BrO$, $[M+Na]^+$ 263.0042, found 263.0040.

(2-Bromo-3-methylphenyl)(phenyl)methanol (1g)

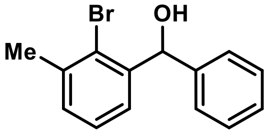

1g

Physical state: white solid;

Yield: 85% (235.0 mg, 1.0 mmol scale);

Rf = 0.2 (PE:EtOAc = 10:1);

M.P.: 59 – 61 ºC;

^{1}H NMR (400 MHz, $CDCl_3$) δ 7.44 – 7.38 (m, 3H), 7.37 – 7.31 (m, 2H), 7.30 – 7.26 (m, 1H), 7.24 (d, *J* = 7.6 Hz, 1H), 7.19 (dd, *J* = 7.5, 1.8 Hz, 1H), 6.27 (s, 1H), 2.42 (s, 3H), 2.36 (brs, 1H);

^{13}C NMR (100 MHz, $CDCl_3$) δ 143.0, 142.5, 138.8, 130.2, 128.6, 127.8, 127.33, 127.29, 126.0, 125.6, 75.4, 24.0;

HRMS (ESI-TOF) calculated for $C_{14}H_{13}BrO$, $[M+Na]^+$ 299.0042, found 299.0043.

(2-Bromo-3-methylphenyl)(o-tolyl)methanol (1h)

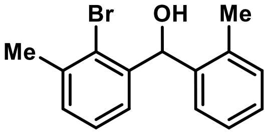

1h

Physical state: white solid;

Yield: 90% (261.5 mg, 1.0 mmol scale);

R_f = 0.35 (PE:EtOAc = 10:1);

M.P.: 101 – 103 ºC;

^{1}H NMR (400 MHz, $CDCl_3$) δ 7.29 (dd, *J* = 7.3, 2.0 Hz, 1H), 7.24 – 7.15 (m, 6H), 6.34 (s, 1H), 2.44 (s, 3H), 2.31 (s, 4H);

^{13}C NMR (100 MHz, $CDCl_3$) δ 142.3, 140.3, 138.8, 136.2, 130.6, 130.3, 127.9, 127.2, 126.4, 126.3, 126.2, 72.7, 23.9, 19.3;

HRMS (ESI-TOF) calculated for $C_{15}H_{15}BrO$, $[M+Na]^+$ 313.0198, found 313.0202.

(2-Bromo-3-methylphenyl)(m-tolyl)methanol (1i)

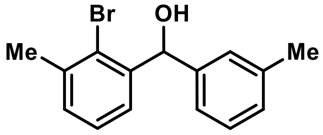

1i

Physical state: white solid;

Yield: 76% (221.0 mg, 1.0 mmol scale);

R_f = 0.3 (PE:EtOAc = 10:1);

M.P.: 49 – 51 ºC;

^{1}H NMR (400 MHz, $CDCl_3$) δ 7.41 (dd, *J* = 7.6, 1.8 Hz, 1H), 7.26 (d, *J* = 7.4 Hz, 1H), 7.24 – 7.16 (m, 4H), 7.09 (d, *J* = 7.0 Hz, 1H), 6.23 (s, 1H), 2.42 (s, 3H), 2.38 (brs, 1H), 2.34 (s, 3H);

^{13}C NMR (100 MHz, $CDCl_3$) δ 143.0, 142.4, 138.7, 138.2, 130.2, 128.6, 128.4, 127.9, 127.3, 126.0, 125.6, 124.4, 75.4, 24.0, 21.6;

HRMS (ESI-TOF) calculated for $C_{15}H_{15}BrO$, $[M+Na]^+$ 313.0198, found 313.0201.

(2-Bromo-3-methylphenyl)(p-tolyl)methanol (**1j**)

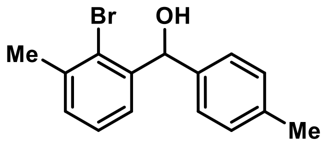

1j

Physical state: white solid;

Yield: 80% (232.3 mg, 1.0 mmol scale);

R_f = 0.4 (PE:EtOAc = 10:1);

M.P.: 63 – 65 ºC;

^{1}H NMR (400 MHz, $CDCl_3$) δ 7.44 (dd, *J* = 7.7, 1.8 Hz, 1H), 7.31 – 7.27 (m, 2H), 7.25 (t, *J* = 7.6 Hz, 1H), 7.18 (dd, *J* = 7.5, 1.8 Hz, 1H), 7.14 (d, *J* = 7.9 Hz, 2H), 6.22 (s, 1H), 2.41 (s, 3H), 2.35 (brs, 1H), 2.33 (s, 3H);

^{13}C NMR (100 MHz, $CDCl_3$) δ 143.1, 139.5, 138.7, 137.6, 130.1, 129.3, 127.3, 125.9, 125.5, 75.3, 23.9, 21.3;

HRMS (ESI-TOF) calculated for $C_{15}H_{15}BrO$, $[M+Na]^+$ 313.0198, found 313.0197.

[1,1'-Biphenyl]-4-yl(2-bromo-3-methylphenyl)methanol (**1k**)

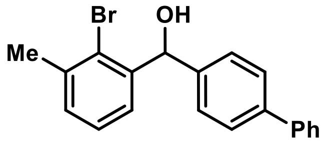

1k

Physical state: white solid;

Yield: 93% (330.0 mg, 1.0 mmol scale);

R_f = 0.5 (PE:DCM = 1:2);

M.P.: 107 – 109 ºC;

^{1}H NMR (400 MHz, $CDCl_3$) δ 7.62 – 7.53 (m, 4H), 7.51 – 7.40 (m, 5H), 7.37 – 7.31 (m, 1H), 7.27 (t, *J* = 7.6 Hz, 1H), 7.21 (dd, *J* = 7.5, 1.7 Hz, 1H), 6.32 (s, 1H), 2.44 (s, 4H);

^{13}C NMR (100 MHz, $CDCl_3$) δ 143.0, 141.5, 140.9, 140.7, 138.8, 130.3, 128.9, 127.7, 127.44, 127.40, 127.3, 127.2, 126.0, 125.6, 75.2, 24.0;

HRMS (ESI-TOF) calculated for $C_{20}H_{17}BrO$, $[M+Na]^+$ 375.0355, found 375.0358.

(2-Bromo-3-methylphenyl)(4-methoxyphenyl)methanol (1m)

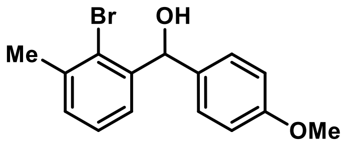

1m

Physical state: white solid;

Yield: 81% (247.6 mg, 1.0 mmol scale);

$\boldsymbol{R_f}$ = 0.15 (PE:EtOAc = 10:1);

M.P.: 81 – 83 ºC;

^{1}H NMR (400 MHz, $CDCl_3$) δ 7.46 (dd, *J* = 7.7, 2.0 Hz, 1H), 7.34 – 7.28 (m, 2H), 7.25 (t, *J* = 7.6 Hz, 1H), 7.18 (dd, *J* = 7.5, 1.9 Hz, 1H), 6.90 – 6.82 (m, 2H), 6.19 (d, *J* = 3.1 Hz, 1H), 3.79 (s, 3H), 2.41 (s, 3H), 2.32 (d, *J* = 3.4 Hz, 1H);

^{13}C NMR (100 MHz, $CDCl_3$) δ 159.2, 143.2, 138.7, 134.7, 130.1, 128.7, 127.3, 125.7, 125.4, 113.9, 75.0, 55.4, 23.9;

HRMS (ESI-TOF) calculated for $C_{15}H_{15}BrO_2$, $[M+Na]^+$ 329.0148, found 329.0151.

(2-Bromo-3-methylphenyl)(4-fluorophenyl)methanol (1n)

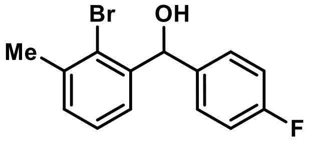

1n

Physical state: white solid;

Yield: 85% (251.8 mg, 1.0 mmol scale);

$\boldsymbol{R_f}$ = 0.5 (PE:EtOAc = 10:1);

M.P.: 61 – 63 ºC;

^{1}H NMR (400 MHz, $CDCl_3$) δ 7.43 – 7.33 (m, 3H), 7.25 (t, *J* = 7.5 Hz, 1H), 7.19 (dd, *J* = 7.5, 1.8 Hz, 1H), 7.05 – 6.97 (m, 2H), 6.23 (d, *J* = 3.3 Hz, 1H), 2.46 (d, *J* = 3.7 Hz, 1H), 2.42 (s, 3H);

^{13}C NMR (100 MHz, $CDCl_3$) δ 162.3 (d, J_{C-F} = 246.1 Hz), 142.9, 138.8, 138.2 (d, J_{C-F} = 3.1 Hz), 130.3, 129.0 (d, J_{C-F} = 8.2 Hz), 127.4, 125.8, 125.4, 115.4 (d, J_{C-F} = 21.4 Hz), 74.7, 23.9;

^{19}F NMR (376 MHz, $CDCl_3$) δ -114.6 – -114.7 (m);

HRMS (ESI-TOF) calculated for $C_{14}H_{12}BrFO$, $[M+Na]^+$ 316.9948, found 316.9947.

(2-Bromo-3-methylphenyl)(4-chlorophenyl)methanol (1o)

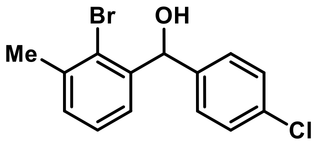

1o

Physical state: white solid;

Yield: 89% (277.0 mg, 1.0 mmol scale);

***R**$_f$* = 0.3 (PE:EtOAc = 10:1);

M.P.: 53 – 55 °C;

^{1}H NMR (400 MHz, $CDCl_3$) δ 7.39 – 7.27 (m, 5H), 7.24 (t, *J* = 7.5 Hz, 1H), 7.19 (dd, *J* = 7.5, 1.9 Hz, 1H), 6.23 (s, 1H), 2.45 (brs, 1H), 2.42 (s, 3H);

^{13}C NMR (100 MHz, $CDCl_3$) δ 142.7, 140.9, 138.9, 133.6, 130.4, 128.7, 128.6, 127.5, 125.9, 125.5, 74.7, 23.9;

HRMS (ESI-TOF) calculated for $C_{14}H_{12}BrClO$, $[M+Na]^+$ 332.9652, found 332.9655.

Synthesis of symmetric secondary benzyl alcohols **1z** and **1z'**.

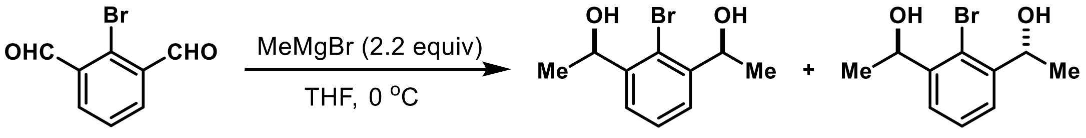

The symmetric secondary benzyl alcohols *meso*-**1z** and *rac*-**1z'** were synthesized following the above steps.

1,1'-(2-Bromo-1,3-phenylene)bis(ethan-1-ol) (*meso*-**1z**)[3]

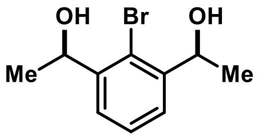

meso-**1z**

Physical state: white solid;

Yield: 38% (187.6 mg, 2.0 mmol scale);

***R**$_f$* = 0.2 (DCM:EtOAc = 10:1);

^{1}H NMR (400 MHz, DMSO-d_6) δ 7.47 (d, *J* = 7.5 Hz, 2H), 7.37 (dd, *J* = 8.3, 6.8 Hz, 1H), 5.34 (d, *J* = 4.3 Hz, 2H), 5.03 (qd, *J* = 6.3, 4.3 Hz, 2H), 1.29 (d, *J* = 6.3 Hz, 6H);

^{13}C NMR (100 MHz, DMSO-d_6) δ 146.1, 127.5, 125.4, 119.9, 67.6, 24.5.

1,1'-(2-Bromo-1,3-phenylene)bis(ethan-1-ol) (*rac*-1z')[3]

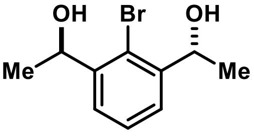

rac-**1z'**

Physical state: white solid;

Yield: 36% (178.1 mg, 2.0 mmol scale);

R_f = 0.35 (DCM:EtOAc = 10:1);

^{1}H NMR (400 MHz, DMSO-d_6) δ 7.49 (d, J = 7.5 Hz, 2H), 7.39 (dd, J = 8.3, 6.8 Hz, 1H), 5.35 (d, J = 4.1 Hz, 2H), 5.03 (qd, J = 6.3, 4.0 Hz, 2H), 1.28 (d, J = 6.3 Hz, 6H);

^{13}C NMR (100 MHz, DMSO-d_6) δ 146.2, 127.6, 125.5, 119.8, 67.6, 24.5.

General procedure II[4]: Synthesis of secondary benzyl alcohols **1l**, **1p** and **1q**.

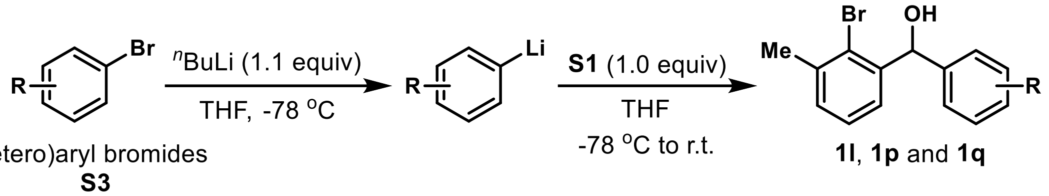

(hetero)aryl bromides **S3** — **1l**, **1p** and **1q**

To a solution of (hetero)aryl bromides **S3** (1.2 mmol, 1.2 equiv) in THF (1.5 mL) at -78 °C was added ^{n}BuLi (0.44 mL of a 2.5 M solution in hexane, 1.1 equiv) in a dropwise fashion. After 30 min, 2-bromo-3-methylbenzaldehyde **S1** (1.0 mmol, 1.0 equiv) in THF (0.5 mL) was added in dropwise. The reaction mixture was allowed to warm to r.t. and then stirred overnight. The reaction was quenched with saturated aqueous NH_4Cl, and most of THF solvent removed under reduced pressure. The residue was diluted with water and dichloromethane (DCM), and the aqueous layer was extracted with DCM. The combined organic layers were washed with brine, dried over Na_2SO_4, and concentrated under reduced pressure. The residue was purified by silica-gel column chromatography to give the products **1l**, **1p** and **1q**.

(2-Bromo-3-methylphenyl)(4-(trifluoromethyl)phenyl)methanol (1l)

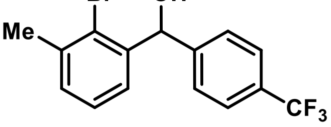

1l

Physical state: white solid;

Yield: 36% (123.0 mg, 1.0 mmol scale);

R_f = 0.3 (PE:EtOAc = 10:1);

M.P.: 60 – 62 ºC;

^{1}H NMR (400 MHz, $CDCl_3$, OH missing) δ 7.59 (d, *J* = 8.3 Hz, 2H), 7.54 (d, *J* = 8.3 Hz, 2H), 7.32 (dd, *J* = 7.5, 2.1 Hz, 1H), 7.25 (t, *J* = 7.4 Hz, 1H), 7.21 (dd, *J* = 7.6, 2.0 Hz, 1H), 6.34 (s, 1H), 2.43 (s, 3H);

^{13}C NMR (100 MHz, $CDCl_3$) δ 146.3 (q, $J_{C\text{-}F}$ = 1.1 Hz), 142.4, 139.0, 130.6, 129.9 (q, $J_{C\text{-}F}$ = 32.4 Hz), 127.6, 127.4, 126.1, 125.6, 125.5 (q, $J_{C\text{-}F}$ = 3.8 Hz), 124.2 (q, $J_{C\text{-}F}$ = 272.1 Hz), 74.8, 24.0;

^{19}F NMR (376 MHz, $CDCl_3$) δ -62.5;

HRMS (APCI-TOF) calculated for $C_{15}H_{12}BrF_3O$, $[M\text{-}OH]^+$ 326.9991, found 326.9996.

(2-Bromo-3-methylphenyl)(furan-3-yl)methanol (1p)

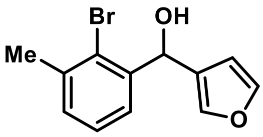

1p

Physical state: white solid;

Yield: 64% (170.0 mg, 1.0 mmol scale);

R$_f$ = 0.35 (PE:DCM = 1:2);

M.P.: 56 – 58 ºC;

^{1}H NMR (400 MHz, $CDCl_3$) δ 7.45 (dd, *J* = 7.6, 1.9 Hz, 1H), 7.36 (t, *J* = 1.8 Hz, 1H), 7.33 (s, 1H), 7.25 (t, *J* = 7.5 Hz, 1H), 7.19 (dd, *J* = 7.6, 1.8 Hz, 1H), 6.37 (d, *J* = 2.2 Hz, 1H), 6.20 (s, 1H), 2.43 (s, 3H), 2.39 (brs, 1H);

^{13}C NMR (100 MHz, $CDCl_3$) δ 143.4, 142.5, 140.4, 138.7, 130.3, 127.6, 127.4, 125.4, 125.1, 109.4, 68.9, 23.9;

HRMS (ESI-TOF) calculated for $C_{12}H_{11}BrO_2$, $[M+Na]^+$ 288.9835, found 288.9832.

(2-Bromo-3-methylphenyl)(5-methylthiophen-2-yl)methanol (1q)

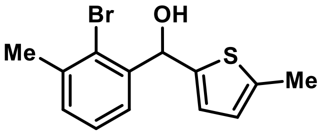

1q

Physical state: light brown solid;

Yield: 96% (285.8 mg, 1.0 mmol scale);

R$_f$ = 0.4 (PE:EtOAc = 10:1);

M.P.: 57 – 59 ºC;

^{1}H NMR (400 MHz, $CDCl_3$) δ 7.54 (dd, *J* = 7.7, 1.8 Hz, 1H), 7.26 (t, *J* = 7.5 Hz, 1H),

7.19 (dd, J = 7.5, 1.8 Hz, 1H), 6.69 (d, J = 3.4 Hz, 1H), 6.55 (dd, J = 3.5, 1.3 Hz, 1H), 6.34 (s, 1H), 2.55 (s, 1H), 2.42 (s, 3H), 2.41 (s, 3H);

^{13}C NMR (100 MHz, $CDCl_3$) δ 144.0, 142.6, 140.4, 138.6, 130.3, 127.4, 125.7, 125.2, 125.0, 124.8, 71.7, 23.9, 15.5;

HRMS (ESI-TOF) calculated for $C_{13}H_{13}BrOS$, $[M+Na]^+$ 318.9763, found 318.9760.

General procedure III: Synthesis of secondary benzyl alcohols **1r** and **1s**.

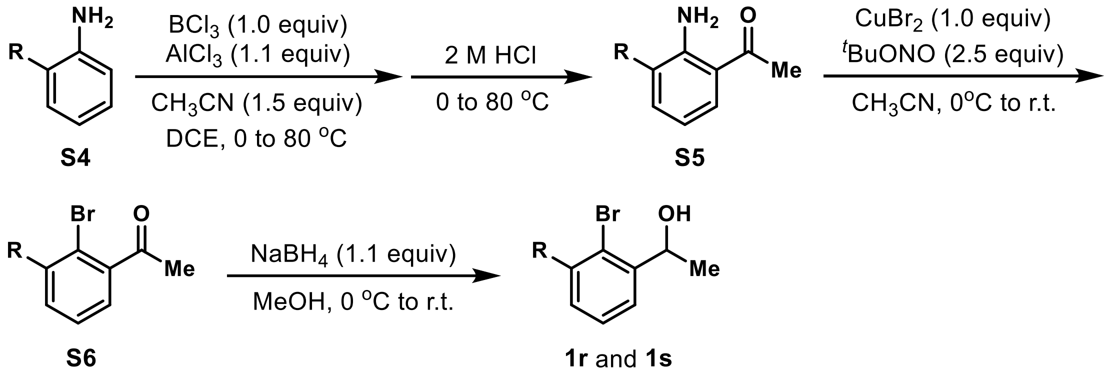

The *ortho*-aminoacetophenones **S5** were synthesized following the reported procedure.[5] To a stirred solution of 2-substitutedanilines **S4** (2.0 mmol, 1.0 equiv) in DCE (4.0 mL) at 0 °C was added boron trichloride (2.0 mL of a 1.0 M solution in DCM, 1.0 equiv) in a dropwise fashion. Then, to the mixture was added aluminum chloride (2.2 mmol, 1.1 equiv) and acetonitrile (3.0 mmol, 1.5 equiv). The reaction mixture was allowed to reflux at 80 °C for 20 h. The reaction mixture was then cooled to 0 °C, followed by addition of 2 M HCl (1.0 mL). The reaction mixture was heated to 80℃ and stirred for an additional 30 min. The reaction mixture was then cooled to r.t., extracted with DCM. The organic layer was washed with 1M NaOH (1.0 mL) and brine, dried over anhydrous Na_2SO_4, and concentrated under reduced pressure. The residue was purified by silica-gel column chromatography (PE/EtOAc) to give the product **S5**.

The *ortho*-bromoacetophenones **S6** were synthesized following the reported procedure.[6] To a solution of above obtained **S5** (1.0 equiv) and ^{t}BuONO (2.5 equiv) in dry CH_3CN (0.3 M) was added a solution of $CuBr_2$ (1.0 equiv) in dry CH_3CN (0.5 M) slowly at 0 °C, then the reaction was warmed to r.t. and stirred for 4 h. Water was added and the pH of the solution was turned down 2, extracted with EtOAc and the combined organic layers were washed with brine, dried over Na_2SO_4, filtered, and concentrated *in vacuo*. The residue was purified by column chromatography on silica-gel to give the product **S6**.

The secondary benzyl alcohols **1r** and **1s** were synthesized following the reported procedure.[1] Sodium borohydride (1.1 equiv) was added portionwise to a solution of the above obtained ketones **S6** (1.0 equiv) dissolved in methanol (0.5 M) at 0 °C. Then, the mixture was warmed to r.t. The reaction progress was monitored by TLC. The mixture was concentrated under reduced pressure before being diluted with DCM, washed with water and brine and then dried over anhydrous Na_2SO_4, filtered and evaporated *in vacuo*. The residue was purified by silica-gel column chromatography to give the corresponding products **1r** and **1s**.

1-(2-Bromo-3-isopropylphenyl)ethan-1-ol (**1r**)

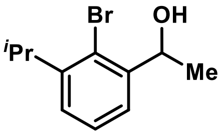

1r

Physical state: yellow viscous liquid;

Yield: 33% (three steps, 160.0 mg, 2.0 mmol scale);

***R**f* = 0.35 (PE:EtOAc = 10:1);

^{1}H NMR (400 MHz, $CDCl_3$) δ 7.44 (dd, *J* = 7.6, 1.8 Hz, 1H), 7.31 (t, *J* = 7.7 Hz, 1H), 7.21 (dd, *J* = 7.7, 1.8 Hz, 1H), 5.34 (q, *J* = 6.4 Hz, 1H), 3.54 – 3.41 (m, 1H), 2.10 (s, 1H), 1.49 (d, *J* = 6.4 Hz, 3H), 1.25 (t, *J* = 6.7 Hz, 6H);

^{13}C NMR (100 MHz, $CDCl_3$) δ 147.9, 145.3, 127.9, 125.8, 124.3, 124.0, 70.0, 33.2, 23.7, 23.2, 23.0;

HRMS (ESI-TOF) calculated for $C_{11}H_{15}BrO$, $[M+Na]^+$ 265.0198, found 265.0189.

1-(2-Bromo-[1,1'-biphenyl]-3-yl)ethan-1-ol (**1s**)

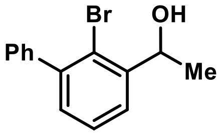

1s

Physical state: white solid;

Yield: 30% (three steps, 165.1 mg, 2.0 mmol scale);

***R**f* = 0.25 (PE:EtOAc = 10:1);

M.P.: 93 – 95 ºC;

^{1}H NMR (400 MHz, $CDCl_3$) δ 7.62 (dd, *J* = 7.7, 1.8 Hz, 1H), 7.46 – 7.33 (m, 6H), 7.23 (dd, *J* = 7.5, 1.8 Hz, 1H), 5.37 (qd, *J* = 6.3, 2.8 Hz, 1H), 2.12 (d, *J* = 3.3 Hz, 1H), 1.54 (d, *J* = 6.4 Hz, 3H);

^{13}C NMR (100 MHz, $CDCl_3$) δ 145.6, 143.5, 141.9, 130.3, 129.6, 128.1, 127.7, 127.6, 125.7, 122.3, 69.9, 23.8;
HRMS (ESI-TOF) calculated for $C_{14}H_{13}BrO$, $[M+Na]^+$ 299.0042, found 299.0044.

General procedure IV: Synthesis of secondary benzyl alcohols **1d**, **1f** and **1u**.

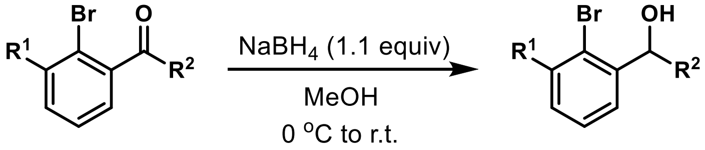

S7 **1d**, **1f** and **1u**

The corresponding ketones **S7** were synthesized following the reported procedure.[6] Next, the secondary benzyl alcohols **1d**, **1f** and **1u** were synthesized following the general procedure III (step three).

1-(2-Bromo-3-methylphenyl)-2,2-dimethylpropan-1-ol (1d)

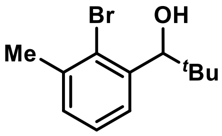

1d

Physical state: white solid;
Yield: 89% (228.0 mg, 1.0 mmol scale);
R$_f$ = 0.3 (PE:EtOAc = 10:1);
M.P.: 54 – 56 °C;
^{1}H NMR (400 MHz, $CDCl_3$) δ 7.37 (dd, *J* = 7.7, 2.0 Hz, 1H), 7.21 (t, *J* = 7.5 Hz, 1H), 7.16 (dd, *J* = 7.6, 1.8 Hz, 1H), 5.14 (s, 1H), 2.43 (s, 3H), 1.84 (brs, 1H), 1.00 (s, 9H);
^{13}C NMR (100 MHz, $CDCl_3$) δ 142.2, 138.2, 129.9, 127.1, 127.0, 126.4, 79.4, 37.2, 26.2, 24.6;
HRMS (ESI-TOF) calculated for $C_{12}H_{17}BrO$, $[M+Na]^+$ 279.0355, found 279.0357.

(2-Bromo-3-methylphenyl)(cyclohexyl)methanol (1f)

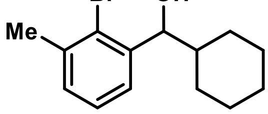

1f

Physical state: white solid;
Yield: 95% (268.6 mg, 1.0 mmol scale);
R$_f$ = 0.35 (PE:EtOAc = 10:1);
M.P.: 70 – 72 °C;

^{1}H NMR (400 MHz, $CDCl_3$) δ 7.30 (dd, J = 7.7, 1.8 Hz, 1H), 7.21 (t, J = 7.5 Hz, 1H), 7.15 (dd, J = 7.5, 1.8 Hz, 1H), 4.96 (d, J = 6.0 Hz, 1H), 2.43 (s, 3H), 1.91 (brs, 1H), 1.86 – 1.63 (m, 5H), 1.52 – 1.45 (m, 1H), 1.29 – 1.11(m, 5H);
^{13}C NMR (100 MHz, $CDCl_3$) δ 143.2, 138.3, 129.7, 127.0, 125.8, 125.6, 77.6, 43.9, 29.9, 27.5, 26.6, 26.5, 26.2, 24.2;
HRMS (ESI-TOF) calculated for $C_{14}H_{19}BrO$, $[M+Na]^+$ 305.0511, found 305.0510.

(2-Bromo-3-chlorophenyl)(phenyl)methanol (1u)

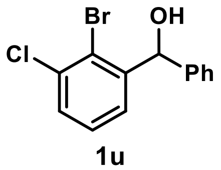

1u

Physical state: white solid;
Yield: 94% (278.6 mg, 1.0 mmol scale);
R_f = 0.3 (PE:EtOAc = 10:1);
M.P.: 63 – 65 °C;
^{1}H NMR (400 MHz, $CDCl_3$) δ 7.55 (dd, J = 7.8, 1.7 Hz, 1H), 7.42 (dd, J = 8.0, 1.7 Hz, 1H), 7.40 – 7.27 (m, 6H), 6.21 (d, J = 3.7 Hz, 1H), 2.51 (d, J = 3.9 Hz, 1H);
^{13}C NMR (100 MHz, $CDCl_3$) δ 145.3, 141.8, 135.3, 129.6, 128.7, 128.3, 128.2, 127.3, 126.5, 122.9, 75.6;
HRMS (ESI-TOF) calculated for $C_{13}H_{10}BrClO$, $[M+Na]^+$ 318.9496, found 318.9501.

General procedure V: Synthesis of secondary benzyl alcohol **1v**.

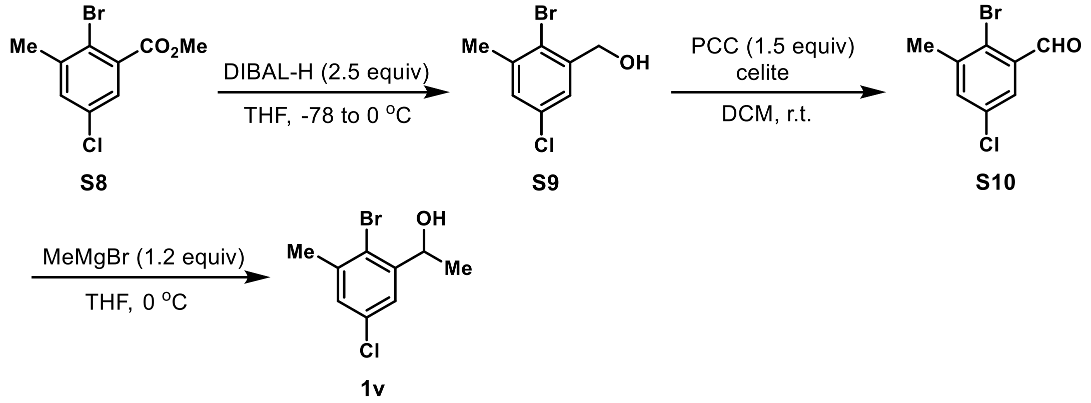

The methyl 2-bromo-5-chloro-3-methylbenzoate **S8** was known compound and was synthesized following the reported procedure.[6] DIBAL-H (1.0 M in hexane, 2.5 mL) was added dropwise to a cooled solution of **S8** (1.0 mmol, 1.0 equiv) in THF (4.0 mL) at -78 °C. The mixture was stirred at -78 °C for 30 min and warmed to 0 °C and then stirred for additional 30 min. The reaction mixture was cooled to -78 °C and

methanol was added dropwise followed by saturated aqueous Rochelle salt and EtOAc. After being stirred at r.t. for 2 h, the organic layer was separated and dried over anhydrous Na_2SO_4, filtered and evaporated *in vacuo*. The residue was purified by silica-gel column chromatography (PE/EtOAc) to give the product **S9** (216.0 mg, 92%).

PCC (1.38 mmol, 1.5 equiv) was added to a solution of (2-bromo-5-chloro-3-methylphenyl)methanol **S9** (0.92 mmol) in DCM (4.6 mL). The mixture was stirred at r.t. for 4 h. After filtration, the filtrate was washed with brine, dried over anhydrous Na_2SO_4, filtered and evaporated *in vacuo.* The crude product 2-bromo-5-chloro-3-methylbenzaldehyde **S10** was used in next step without further purification. Next, following the general procedure I, **1v** (206.2 mg, 90% yield, two steps) was obtained as a white solid.

1-(2-Bromo-5-chloro-3-methylphenyl)ethan-1-ol (**1v**)

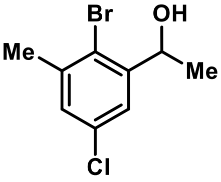

1v

Physical state: white solid;

Yield: 83% (three steps, 206.2 mg, 1.0 mmol scale);

***R**_f_* = 0.3 (PE:EtOAc = 10:1);

M.P.: 54 – 56 °C;

^{1}H NMR (400 MHz, $CDCl_3$) δ 7.43 (d, *J* = 2.6 Hz, 1H), 7.15 (d, *J* = 2.6 Hz, 1H), 5.25 (q, *J* = 6.3 Hz, 1H), 2.39 (s, 3H), 2.08 (brs, 1H), 1.46 (d, *J* = 6.4 Hz, 3H);

^{13}C NMR (100 MHz, $CDCl_3$) δ 146.9, 140.2, 133.5, 129.5, 124.3, 122.1, 69.5, 23.7, 23.6;

HRMS (APCI-TOF) calculated for $C_9H_{10}BrClO$, $[M-OH]^+$ 230.9571, found 230.9583.

General procedure VI: Synthesis of secondary benzyl alcohol **1w**.

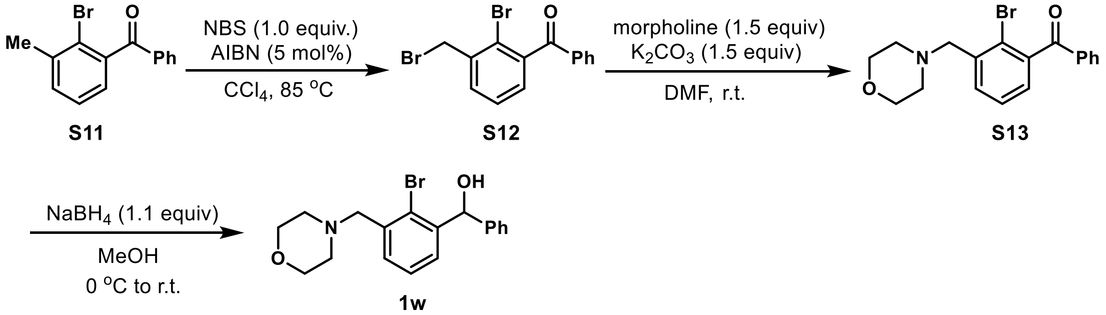

The (2-bromo-3-methylphenyl)(phenyl)methanone **S11** was synthesized following the reported procedure.[6] A solution of **S11** (1.0 mmol, 1.0 equiv), NBS (1.0 mmol, 1.0 equiv) and AIBN (0.05 mmol, 0.05 equiv) in dry CCl_4 (4 mL) was stirred at 85 °C for 3 h. Then, the reaction mixture was cooled to r.t. and filtered. After removal of solvent, EtOAc (5 mL) was added into the residue, washed with 2 N $Na_2S_2O_3$, the aqueous layer was extracted with EtOAc. The combined organic layers were washed with brine, dried over Na_2SO_4, filtered and concentrated *in vacuo*. The residue was purified by column chromatography on silica gel (PE/EtOAc) to give the product **S12** (213.0 mg, 60% yield) as a white solid.

To a flask charged with **S12** (0.6 mmol, 1.0 equiv), K_2CO_3 (0.9 mmol, 1.5 equiv) and DMF (2 mL), then morpholine (0.9 mmol, 1.5 equiv) was added. The mixture was stirred at r.t. for 4 h, then water (10 mL) was added, and extracted with EtOAc. The combined organic layers were washed with brine, dried over Na_2SO_4, filtered and concentrated *in vacuo.* The crude product **S13** was used in next step without further purification. Next, following the general procedure III (step three), **1w** (175.8 mg, 81% yield, two steps) was obtained as a colorless viscous liquid.

(2-Bromo-3-(morpholinomethyl)phenyl)(phenyl)methanol (1w)

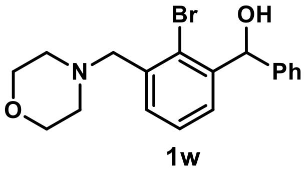

1w

Physical state: colorless viscous liquid;

Yield: 49% (three steps, 175.8 mg, 1.0 mmol scale);

R_f = 0.4 (PE:EtOAc = 2:1);

^{1}H NMR (400 MHz, $CDCl_3$) δ 7.49 (dd, *J* = 7.7, 1.8 Hz, 1H), 7.45 – 7.36 (m, 3H), 7.35 – 7.23 (m, 4H), 6.27 (s, 1H), 3.69 (t, *J* = 4.6 Hz, 4H), 3.59 (q, *J* = 14.4 Hz, 2H), 2.70 (brs, 1H), 2.50 (t, *J* = 4.6 Hz, 4H);

^{13}C NMR (100 MHz, $CDCl_3$) δ 143.4, 142.5, 137.8, 130.0, 128.5, 127.8, 127.4, 127.30, 127.25, 125.3, 75.1, 67.1, 62.9, 53.7;

HRMS (ESI-TOF) calculated for $C_{18}H_{20}BrNO_2$, $[M+H]^+$ 362.0750, found 362.0746.

3. Optimization of reaction conditions

Table S1. Screening of ligand[a]

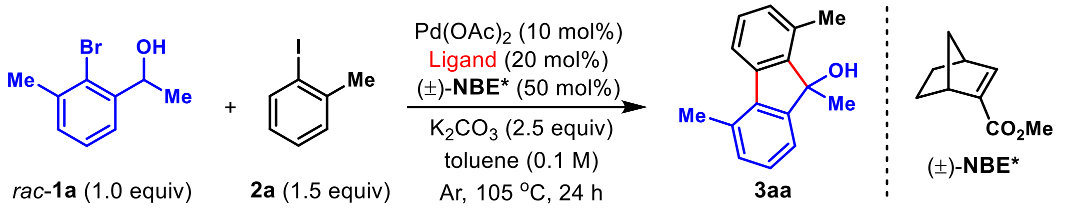

Entry	Ligand	Yield (%)[b]	*E.e.* (%)[c]
1[d]	None	8	98
2[d]	TFP	24 (20)	98
3	PPh_3	6	--
4	PCy_3	n.d.	--
5	MePhos	trace	--
6	JohnPhos	10	--
7	$AsPh_3$	57	--
8[e]	BINAP	26	--
9[e]	XantPhos	5	--
10[e]	DPEPhos	65	--
11[e]	DPPF	39	--
12[e]	DPPM	5	--
13[e]	DPPE	66	--
14[e]	DPPP	78 (73)	--
15[e]	DPPB	64	--
16[e]	DPPPe	73	--
17[e]	DPPH	59	--
18[d,e]	DPPP	(75)	98

[a]All reactions were performed on a 0.1 mmol scale. [b]GC yield with biphenyl as an internal standard and isolated yield shown in parentheses. [c]*E.e.* was determined by chiral HPLC analysis. [d]**NBE*** (99% *e.e.*) instead of (±)-**NBE***. [e]10 mol% ligand was applied. --: not detected.

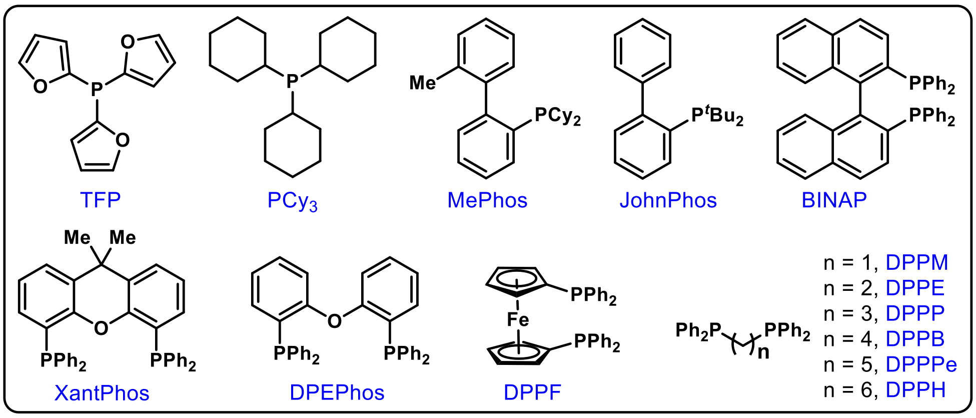

Table S2. Screening of base[a]

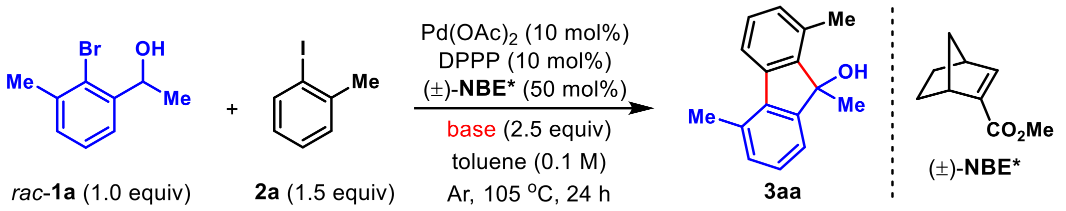

Entry	Base	Yield (%)[b]
1	Na_2CO_3	6
2	K_2CO_3	78
3	Cs_2CO_3	38
4	K_3PO_4	27
5	CsF	25
6	KOAc	6
7	CsOAc	5
8	KOPiv	8

[a]All reactions were performed on a 0.1 mmol scale. [b]GC yield with biphenyl as an internal standard.

Table S3. Screening of palladium catalyst and temperature[a]

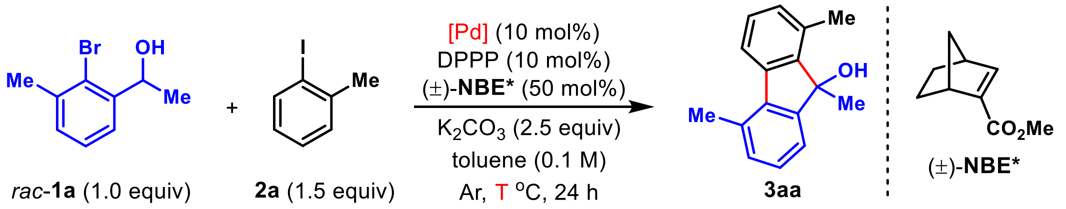

Entry	[Pd]	Temp. (°C)	Yield (%)[b]
1	$Pd(OAc)_2$	105	78
2	$PdCl_2$	105	39
3	PdI_2	105	23
4	$Pd(TFA)_2$	105	43
5	$Pd(CH_3CN)Cl_2$	105	35
6[c]	$[Pd(allyl)Cl]_2$	105	5
7[c]	Pd_2dba_3	105	12
8	$Pd(PPh_3)_4$	105	n.d.
9	$Pd(OAc)_2$	100	63
10	$Pd(OAc)_2$	110	83
11	$Pd(OAc)_2$	115	73
12	$Pd(OAc)_2$	120	66

[a]All reactions were performed on a 0.1 mmol scale. [b]GC yield with biphenyl as an internal standard. [c]5 mol% palladium catalyst was applied. dba: dibenzylideneacetone.

Table S4. Screening of solvent and concentration[a]

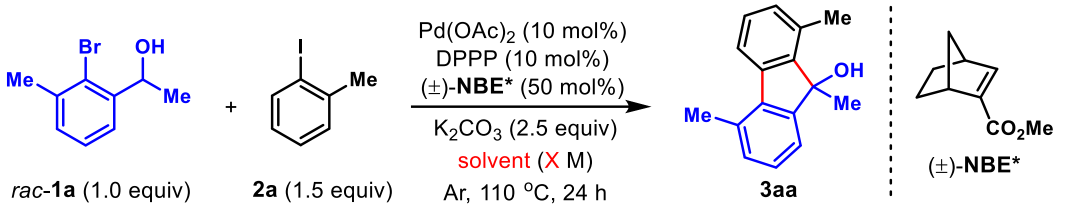

Entry	Solvent	Concentration	Yield (%)[b]
1	toluene	0.1 M	83
2	CH_3CN	0.1 M	12
3	1,4-dioxane	0.1 M	27
4	DCE[c]	0.1 M	89
5	DME	0.1 M	13
6	DMF	0.1 M	8
7	NMP	0.1 M	trace
8	DCE	0.2 M	82
9	DCE	0.05 M	68

[a]All reactions were performed on a 0.1 mmol scale. [b]GC yield with biphenyl as an internal standard. [c]The yield of side product **3aa''** was reduced to 5% (9% yield of **3aa''** using toluene as solvent).

Table S5. Screening of the loading of catalyst and ligand[a]

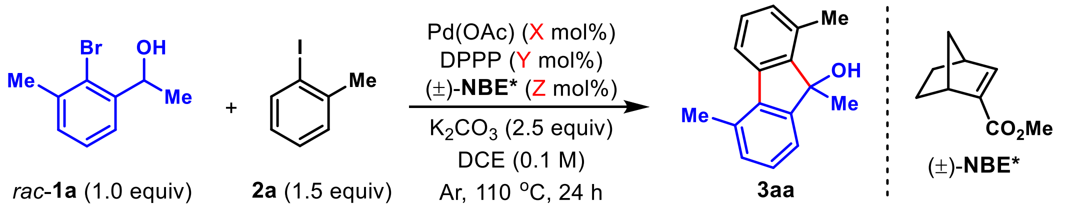

Entry	X (mol%)	Y (mol%)	Z (mol%)	Yield (%)[b]	*E.e.* (%)[c]
1	10	10	50	89	--
2	10	10	30	89	--
3	10	10	25	88	--
4	10	10	20	79	--
5	10	10	10	63	--
6	5	5	25	70	--
7	5	10	25	n.d.	--
8	10	5	25	55	--
9[d]	10	10	25	87	--
10[e]	10	10	25	72	--
11[d,f]	10	10	25	88 (82)	98

[a]All reactions were performed on a 0.1 mmol scale. [b]GC yield with biphenyl as an internal standard and isolated yield shown in parentheses. [c]*E.e.* was determined by chiral HPLC analysis. [d]1.5 equiv of K_2CO_3 was applied. [e]1.0 equiv of K_2CO_3 was applied. [f]**NBE*** (99% *e.e.*) instead of (±)-**NBE***.

4. General procedure for the synthesis of chiral fluorenols

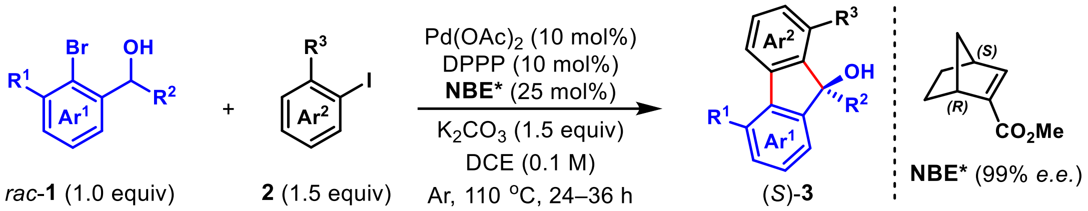

To a 10 mL of oven-dried vial equipped with a magnetic stir bar was charged with secondary alcohol **1** (0.1 mmol, 1.0 equiv), $Pd(OAc)_2$ (0.01 mmol, 10 mol%), DPPP (0.01 mmol, 10 mol%), **NBE*** (0.025 mmol, 25 mol%), K_2CO_3 (0.15 mmol, 1.5 equiv), aryl iodide **2** (0.15 mmol, 1.5 equiv) and dry DCE (1 mL) under Ar. Then the reaction was stirred at 110 °C for 24–36 h. After cooling to r.t., the mixture was filtered through a thin pad of celite eluting with ethyl acetate (10 mL), and the combined filtrate was concentrated *in vacuo*. The residue was purified by column chromatography on silica gel to give the desired product **3**.

(*S*)-1,5,9-Trimethyl-9*H*-fluoren-9-ol (3aa)[6]

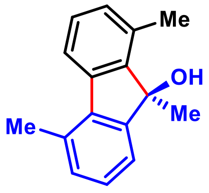

3aa

Physical state: pale yellow solid;
Yield: 82% (18.3 mg);
R_f = 0.3 (PE:EtOAc = 10:1);
M.P.: 155 – 157 °C;
HPLC: 98% *e.e.* (Daicel chiralpak AD-H column, 10% ^{i}PrOH in ^{n}hexane, 1 mL/min, λ = 290 nm), t_R (major) = 6.58 min, t_R (minor) = 7.12 min;
$[\alpha]_D^{25}$: 31.5 (c 1.0, $CHCl_3$);
^{1}H NMR (400 MHz, $CDCl_3$) δ 7.61 (d, J = 7.6 Hz, 1H), 7.40 (d, J = 7.4 Hz, 1H), 7.27 (t, J = 7.6 Hz, 1H), 7.22 (t, J = 7.5 Hz, 1H), 7.13 (d, J = 7.5 Hz, 1H), 7.07 (d, J = 7.6 Hz, 1H), 2.65 (s, 3H), 2.63 (s, 3H), 1.87 (s, 1H), 1.79 (s, 3H);
^{13}C NMR (100 MHz, $CDCl_3$) δ 151.2, 146.8, 140.2, 136.5, 135.4, 133.2, 131.3, 129.9, 128.7, 127.7, 120.9, 120.5, 80.6, 25.0, 21.1, 18.1;
HRMS (ESI-TOF) calculated for $C_{16}H_{16}O$, $[M+Na]^+$ 247.1093, found 247.1094.

4,6,10-Trimethyl-6*H*-benzo[*c*]chromene (**3aa'**)

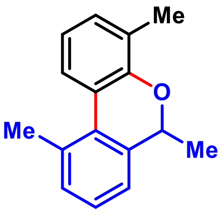

3aa'

Physical state: colorless oil;

R_f = 0.6 (PE:EtOAc = 25:1);

^{1}H NMR (400 MHz, $CDCl_3$) δ 7.62 (dd, *J* = 8.0, 1.7 Hz, 1H), 7.24 – 7.18 (m, 2H), 7.12 (ddd, *J* = 7.4, 1.6, 0.8 Hz, 1H), 7.09 – 7.05 (m, 1H), 6.97 (t, *J* = 7.7 Hz, 1H), 5.10 (q, *J* = 6.6 Hz, 1H), 2.65 (s, 3H), 2.31 (s, 3H), 1.59 (d, *J* = 6.6 Hz, 3H);

^{13}C NMR (100 MHz, $CDCl_3$) δ 153.2, 138.9, 134.4, 131.9, 130.1, 129.3, 127.2, 127.1, 125.6, 123.8, 121.8, 120.5, 74.5, 23.4, 19.5, 16.2;

HRMS (ESI-TOF) calculated for $C_{16}H_{16}O$, $[M+H]^+$ 225.1274, found 225.1276.

1-(3',6-Dimethyl-[1,1'-biphenyl]-2-yl)ethan-1-one (**3aa''**)

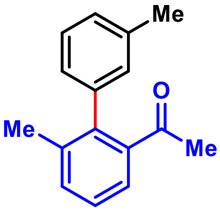

3aa''

Physical state: colorless oil;

R_f = 0.4 (PE:EtOAc = 25:1);

^{1}H NMR (400 MHz, $CDCl_3$) δ 7.37 (dd, *J* = 7.5, 3.7 Hz, 2H), 7.33 – 7.27 (m, 2H), 7.19 (d, *J* = 7.6 Hz, 1H), 7.06 – 6.99 (m, 1H), 2.38 (s, 3H), 2.17 (s, 3H), 1.88 (s, 3H);

^{13}C NMR (100 MHz, $CDCl_3$) δ 205.1, 142.0, 139.65, 139.63, 138.2, 136.8, 132.6, 130.3, 128.5, 128.4, 127.4, 126.8, 125.2, 30.6, 21.6, 20.7;

HRMS (ESI-TOF) calculated for $C_{16}H_{16}O$, $[M+H]^+$ 225.1274, found 225.1271.

(*S*)**-1-Isopropyl-5,9-dimethyl-9*H*-fluoren-9-ol** (**3ab**)[6]

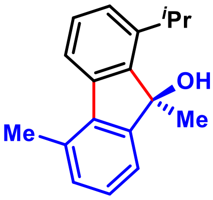

3ab

Physical state: pale yellow solid;

Yield: 80% (20.2 mg);

R_f = 0.45 (PE:EtOAc = 10:1);

M.P.: 160 – 162 ºC;

HPLC: 96% *e.e.* (Daicel chiralpak AD-H column, 2% ^{i}PrOH in ^{n}hexane, 1 mL/min, λ = 230 nm), t_R (major) = 16.07 min, t_R (minor) = 14.97 min;

$[\alpha]_D^{25}$: 33.8 (c 1.0, $CHCl_3$);

^{1}H NMR (400 MHz, $CDCl_3$) δ 7.62 (d, *J* = 7.4 Hz, 1H), 7.37 (t, *J* = 7.8 Hz, 2H), 7.26 (d, *J* = 7.6 Hz, 1H), 7.21 (t, *J* = 7.5 Hz, 1H), 7.12 (d, *J* = 7.4 Hz, 1H), 3.94 – 3.82 (m, 1H), 2.65 (s, 3H), 2.04 (s, 1H), 1.78 (s, 3H), 1.34 (d, *J* = 7.0 Hz, 3H), 1.30 (d, *J* = 6.8 Hz, 3H);

^{13}C NMR (100 MHz, $CDCl_3$) δ 151.4, 147.1, 145.5, 140.0, 136.3, 133.1, 131.3, 129.2, 127.7, 125.3, 120.9, 120.4, 80.7, 28.1, 26.9, 24.6, 24.4, 21.2.

(*S*)**-2-(9-Hydroxy-5,9-dimethyl-9*H*-fluoren-1-yl)acetonitrile** (**3ac**)

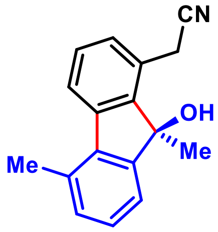

3ac

Physical state: brown solid;

Yield: 51% (12.7 mg);

R_f = 0.3 (PE:EtOAc = 5:1);

M.P.: 128 – 130 ºC;

HPLC: 90% *e.e.* (Daicel chiralpak AD-H column, 20% ^{i}PrOH in ^{n}hexane, 1 mL/min, λ = 230 nm), t_R (major) = 13.99 min, t_R (minor) = 6.70 min;

$[\alpha]_D^{25}$: 34.6 (c 1.0, $CHCl_3$);

^{1}H NMR (400 MHz, $CDCl_3$) δ 7.73 (d, *J* = 7.4 Hz, 1H), 7.39 (dt, *J* = 14.4, 7.4 Hz, 3H), 7.24 (t, *J* = 7.5 Hz, 1H), 7.15 (d, *J* = 7.5 Hz, 1H), 4.28 – 4.08 (m, 2H), 2.64 (s, 3H), 2.06 (brs, 1H), 1.72 (s, 3H);

^{13}C NMR (100 MHz, $CDCl_3$) δ 150.6, 146.8, 140.8, 135.8, 133.6, 131.7, 129.8, 128.3, 127.8, 127.4, 123.2, 120.5, 118.4, 80.5, 26.1, 21.1, 19.6;

HRMS (ESI-TOF) calculated for $C_{17}H_{15}NO$, $[M+H]^+$ 250.1226, found 250.1218.

(*S*)-Methyl-2-(9-hydroxy-5,9-dimethyl-9*H*-fluoren-1-yl)acetate (3ad)

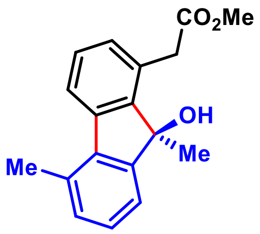

3ad

Physical state: pale yellow solid;

Yield: 50% (14.1 mg);

R$_f$ = 0.15 (PE:EtOAc = 10:1);

M.P.: 84 – 86 ºC;

HPLC: 97% *e.e.* (Daicel chiralpak AD-H column, 10% ^{i}PrOH in ^{n}hexane, 1 mL/min, λ = 230 nm), t_R (major) = 11.36 min, t_R (minor) = 12.36 min;

$[\alpha]_D^{25}$: -20.3 (c 1.0, $CHCl_3$);

^{1}H NMR (400 MHz, $CDCl_3$) δ 7.72 (d, *J* = 7.6 Hz, 1H), 7.41 (d, *J* = 7.4 Hz, 1H), 7.35 (t, *J* = 7.7 Hz, 1H), 7.23 (t, *J* = 7.5 Hz, 1H), 7.15 (t, *J* = 8.0 Hz, 2H), 4.16 – 3.98 (m, 2H), 3.71 (s, 3H), 2.65 (s, 3H), 2.51 (brs, 1H), 1.74 (s, 3H);

^{13}C NMR (100 MHz, $CDCl_3$) δ 173.0, 151.3, 147.6, 140.7, 136.0, 133.3, 131.3, 131.2, 129.6, 129.1, 128.0, 122.5, 120.4, 80.4, 52.3, 37.0, 26.6, 21.1;

HRMS (ESI-TOF) calculated for $C_{18}H_{18}O_3$, $[M+Na]^+$ 305.1148, found 305.1146.

(*S*)-5,9-Dimethyl-1-(trifluoromethyl)-9*H*-fluoren-9-ol (3ae)

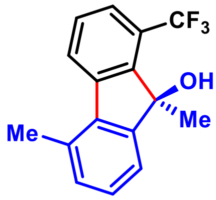

3ae

Physical state: pale yellow solid;

Yield: 65% (18.2 mg);

R$_f$ = 0.4 (PE:EtOAc = 10:1);

M.P.: 111 – 113 ºC;

HPLC: 98% *e.e.* (Daicel chiralpak AD-H column, 5% ^{i}PrOH in ^{n}hexane, 1 mL/min, λ = 230 nm), t_R (major) = 11.06 min, t_R (minor) = 8.61 min;

$[\alpha]_D^{25}$: -3.9 (c 1.0, $CHCl_3$);

^{1}H NMR (400 MHz, $CDCl_3$) δ 8.00 (d, *J* = 7.6 Hz, 1H), 7.57 (d, *J* = 7.8 Hz, 1H), 7.50

(t, J = 7.8 Hz, 1H), 7.44 (d, J = 7.5 Hz, 1H), 7.28 (t, J = 7.5 Hz, 1H), 7.17 (d, J = 7.5 Hz, 1H), 2.68 (s, 3H), 2.39 (brs, 1H), 1.82 (s, 3H);

^{13}C NMR (100 MHz, $CDCl_3$) δ 151.0, 147.2 (q, $J_{C\text{-}F}$ = 1.8 Hz), 142.5, 134.7, 133.2, 131.6, 129.2, 128.8, 127.0 (q, $J_{C\text{-}F}$ = 32.7 Hz), 126.8, 125.0 (q, $J_{C\text{-}F}$ = 5.8 Hz), 124.6 (q, $J_{C\text{-}F}$ = 272.1 Hz), 120.7, 80.6, 27.3 (q, $J_{C\text{-}F}$ = 3.5 Hz), 21.3;

^{19}F NMR (376 MHz, $CDCl_3$) δ -56.7;

HRMS (ESI-TOF) calculated for $C_{16}H_{13}F_3O$, $[M+Na]^+$ 301.0811, found 301.0806.

(*S*)**-1-Methoxy-5,9-dimethyl-9*H*-fluoren-9-ol** (**3af**)[6]

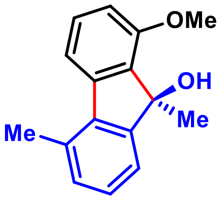

3af

Physical state: pale yellow solid;

Yield: 50% (12.1 mg);

R$_f$ = 0.1 (PE:EtOAc = 10:1);

M.P.: 78 – 80 ºC;

HPLC: 98% *e.e.* (Daicel chiralpak OJ column, 10% ^{i}PrOH in ^{n}hexane, 1 mL/min, λ = 254 nm), t_R (major) = 8.97 min, t_R (minor) = 10.51 min;

$[\alpha]_D^{25}$: -32.5 (c 1.0, $CHCl_3$);

^{1}H NMR (400 MHz, $CDCl_3$) δ 7.46 – 7.38 (m, 2H), 7.35 (t, J = 7.9 Hz, 1H), 7.24 (t, J = 7.6 Hz, 1H), 7.14 (d, J = 7.5 Hz, 1H), 6.85 (d, J = 8.0 Hz, 1H), 3.96 (s, 3H), 2.71 (s, 1H), 2.65 (s, 3H), 1.85 (s, 3H);

^{13}C NMR (100 MHz, $CDCl_3$) δ 156.4, 150.2, 141.8, 136.8, 135.7, 133.3, 131.2, 130.3, 127.9, 120.6, 116.2, 110.0, 79.9, 55.5, 25.6, 20.9.

(*S*)**-1-(Benzyloxy)-5,9-dimethyl-9*H*-fluoren-9-ol** (**3ag**)[6]

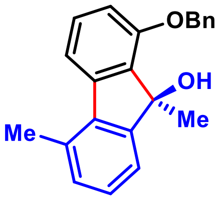

3ag

Physical state: yellowish viscous liquid;

Yield: 61% (19.2 mg);

***R**$_f$* = 0.2 (PE:EtOAc = 10:1);

HPLC: 98% *e.e.* (Daicel chiralpak AD-H column, 20% ^{i}PrOH in ^{n}hexane, 1 mL/min, λ = 230 nm), t_R (major) = 8.54 min, t_R (minor) = 12.73 min;

$[\alpha]_D^{25}$: 1.2 (c 1.0, $CHCl_3$);

^{1}H NMR (400 MHz, $CDCl_3$) δ 7.49 (d, *J* = 7.0 Hz, 2H), 7.46 – 7.39 (m, 4H), 7.39 – 7.30 (m, 2H), 7.25 (t, *J* = 7.4 Hz, 1H), 7.15 (d, *J* = 7.5 Hz, 1H), 6.89 (d, *J* = 8.2 Hz, 1H), 5.24 (s, 2H), 2.78 (brs, 1H), 2.66 (s, 3H), 1.90 (s, 3H);

^{13}C NMR (100 MHz, $CDCl_3$) δ 155.5, 150.2, 142.0, 136.9, 136.8, 136.2, 133.4, 131.2, 130.2, 128.9, 128.2, 128.0, 127.4, 120.7, 116.5, 111.2, 79.9, 70.1, 26.0, 20.9.

(*S*)-**1-Fluoro-5,9-dimethyl-9*H*-fluoren-9-ol (3ah)**[6]

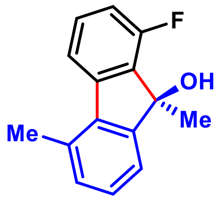

3ah

Physical state: pale yellow solid;

Yield: 46% (10.6 mg);

***R**$_f$* = 0.3 (PE:EtOAc = 10:1);

M.P.: 116 – 118 ºC;

HPLC: 99% *e.e.* (Daicel chiralpak OD-H column, 3% ^{i}PrOH in ^{n}hexane, 1 mL/min, λ = 290 nm), t_R (major) = 11.59 min, t_R (minor) =10.07 min;

$[\alpha]_D^{25}$: -11.1 (c 1.0, $CHCl_3$);

^{1}H NMR (400 MHz, $CDCl_3$) δ 7.54 (d, *J* = 7.6 Hz, 1H), 7.42 (d, *J* = 7.4 Hz, 1H), 7.39 – 7.31 (m, 1H), 7.26 (t, *J* = 7.5 Hz, 1H), 7.16 (d, *J* = 7.5 Hz, 1H), 6.97 (t, *J* = 8.9 Hz, 1H), 2.64 (s, 3H), 2.21 (s, 1H), 1.87 (s, 3H);

^{13}C NMR (100 MHz, $CDCl_3$) δ 160.0 (d, J_{C-F} = 249.0 Hz), 150.1, 143.0 (d, J_{C-F} = 5.9 Hz), 136.2 (d, J_{C-F} = 2.3 Hz), 134.8 (d, J_{C-F} = 14.7 Hz), 133.6, 131.6, 130.9 (d, J_{C-F} = 7.6 Hz), 128.5, 120.8, 119.3 (d, J_{C-F} = 3.2 Hz), 115.0 (d, J_{C-F} = 20.7 Hz), 79.4, 25.4 (d, J_{C-F} = 1.5 Hz), 21.0;

^{19}F NMR (376 MHz, $CDCl_3$) δ -121.2 (q).

(*S*)-1,2,5,9-Tetramethyl-9*H*-fluoren-9-ol (3ai)

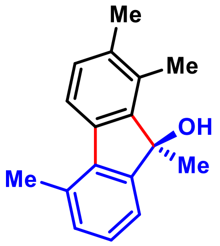

3ai

Physical state: pale yellow solid;

Yield: 73% (17.3 mg);

Rf = 0.3 (PE:EtOAc = 10:1);

M.P.: 134 – 136 ºC;

HPLC: 98% *e.e.* (Daicel chiralpak AD-H column, 5% ^{i}PrOH in ^{n}hexane, 1 mL/min, λ = 230 nm), t_R (major) = 9.60 min, t_R (minor) = 10.18 min;

$[\alpha]_D^{25}$: 36.4 (c 1.0, $CHCl_3$);

^{1}H NMR (400 MHz, $CDCl_3$) δ 7.50 (d, *J* = 7.7 Hz, 1H), 7.38 (d, *J* = 7.4 Hz, 1H), 7.22 – 7.14 (m, 2H), 7.11 (d, *J* = 7.5 Hz, 1H), 2.62 (s, 3H), 2.53 (s, 3H), 2.32 (s, 3H), 1.92 (s, 1H), 1.77 (s, 3H);

^{13}C NMR (100 MHz, $CDCl_3$) δ 151.2, 147.0, 138.1, 137.0, 136.5, 134.3, 132.8, 131.2, 130.1, 127.3, 120.6, 120.4, 80.7, 25.7, 21.0, 19.9, 14.8;

HRMS (ESI-TOF) calculated for $C_{17}H_{18}O$, $[M+Na]^+$ 261.1250, found 261.1252.

(*S*)-2-Fluoro-1,5,9-trimethyl-9*H*-fluoren-9-ol (3aj)

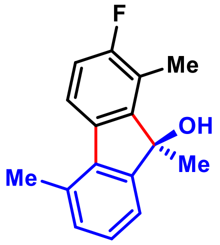

3aj

Physical state: pale yellow solid;

Yield: 78% (18.9 mg);

Rf = 0.4 (PE:EtOAc = 10:1);

M.P.: 131 – 133 ºC;

HPLC: 98% *e.e.* (Daicel chiralpak AD-H column, 5% ^{i}PrOH in ^{n}hexane, 1 mL/min, λ = 230 nm), t_R (major) = 10.33 min, t_R (minor) = 9.59 min;

$[\alpha]_D^{25}$: 33.5 (c 1.0, $CHCl_3$);

^{1}H NMR (400 MHz, $CDCl_3$) δ 7.52 (dd, J = 8.4, 4.7 Hz, 1H), 7.36 (d, J = 7.3 Hz, 1H), 7.19 (t, J = 7.5 Hz, 1H), 7.12 (d, J = 7.5 Hz, 1H), 7.01 (dd, J = 9.8, 8.4 Hz, 1H), 2.60 (s, 3H), 2.52 (d, J = 2.2 Hz, 3H), 1.97 (s, 1H), 1.75 (s, 3H);

^{13}C NMR (100 MHz, $CDCl_3$) δ 161.5 (d, $J_{C\text{-}F}$ = 245.1 Hz), 151.2 (d, $J_{C\text{-}F}$ = 2.0 Hz), 149.4 (d, $J_{C\text{-}F}$ = 4.5 Hz), 135.83, 135.78 (d, $J_{C\text{-}F}$ = 3.0 Hz), 132.7, 131.4, 127.4, 123.2 (d, $J_{C\text{-}F}$ = 18.7 Hz), 121.3 (d, $J_{C\text{-}F}$ = 8.7 Hz), 120.5, 114.9 (d, $J_{C\text{-}F}$ = 24.0 Hz), 80.5 (d, $J_{C\text{-}F}$ = 2.7 Hz), 25.4, 21.0, 10.0 (d, $J_{C\text{-}F}$ = 5.1 Hz);

^{19}F NMR (376 MHz, $CDCl_3$) δ -119.9 (m);

HRMS (ESI-TOF) calculated for $C_{16}H_{15}FO$, $[M+Na]^+$ 265.0999, found 265.1001.

(*S*)-**2-Chloro-1,5,9-trimethyl-9*H*-fluoren-9-ol** (**3ak**)[6]

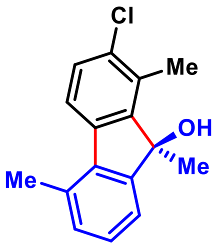

3ak

Physical state: pale yellow solid;

Yield: 66% (17.1 mg);

***R*$_f$** = 0.4 (PE:EtOAc = 10:1);

M.P.: 144 – 146 ºC;

HPLC: 98% *e.e.* (Daicel chiralpak OD-H column, 3% ^{i}PrOH in ^{n}hexane, 1 mL/min, λ = 220 nm), t_R (major) = 7.32 min, t_R (minor) = 8.39 min;

$[\alpha]_D^{25}$: 35.8 (c 1.0, $CHCl_3$);

^{1}H NMR (400 MHz, $CDCl_3$) δ 7.51 (d, J = 8.1 Hz, 1H), 7.37 (dd, J = 7.9, 4.6 Hz, 2H), 7.22 (t, J = 7.5 Hz, 1H), 7.13 (d, J = 7.5 Hz, 1H), 2.63 (s, 3H), 2.61 (s, 3H), 1.94 (s, 1H), 1.74 (s, 3H);

^{13}C NMR (101 MHz, $CDCl_3$) δ 151.2, 148.7, 138.7, 135.5, 134.7, 133.8, 133.1, 131.5, 129.4, 128.0, 121.5, 120.5, 80.7, 25.6, 21.0, 15.3.

(*S*)-**3-Methoxy-1,5,9-trimethyl-9*H*-fluoren-9-ol** (**3al**)

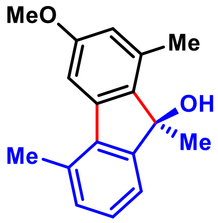

3al

Physical state: pale yellow solid;

Yield: 78% (19.8 mg);

R R_f = 0.2 (PE:EtOAc = 10:1);

M.P.: 149 – 151 ºC;

HPLC: 97% *e.e.* (Daicel chiralpak IG column, 10% ^{i}PrOH in ^{n}hexane, 1 mL/min, λ = 230 nm), t_R (major) = 10.55 min, t_R (minor) = 8.45 min;

$[\alpha]_D^{25}$: 26.5 (c 1.0, $CHCl_3$);

^{1}H NMR (400 MHz, $CDCl_3$) δ 7.38 (d, *J* = 7.4 Hz, 1H), 7.21 (t, *J* = 7.5 Hz, 1H), 7.14 (d, *J* = 2.3 Hz, 1H), 7.11 (d, *J* = 7.5 Hz, 1H), 6.58 (d, *J* = 2.3 Hz, 1H), 3.84 (s, 3H), 2.62 (s, 3H), 2.59 (s, 3H), 1.91 (s, 1H), 1.76 (s, 3H);

^{13}C NMR (100 MHz, $CDCl_3$) δ 160.1, 152.2, 141.7, 139.3, 136.3, 136.2, 133.1, 131.3, 127.9, 120.5, 114.0, 107.7, 80.0, 55.5, 25.2, 21.0, 18.4;

HRMS (ESI-TOF) calculated for $C_{17}H_{18}O_2$, $[M+Na]^+$ 277.1199, found 277.1198.

(*S*)-**3-Fluoro-1,5,9-trimethyl-9*H*-fluoren-9-ol (3am)**[6]

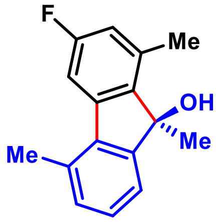

3am

Physical state: white solid;

Yield: 66% (15.9 mg);

R_f = 0.35 (PE:EtOAc = 10:1);

M.P.: 94 – 96 ºC;

HPLC: 98% *e.e.* (Daicel chiralpak AD-H column, 5% ^{i}PrOH in ^{n}hexane, 1 mL/min, λ = 230 nm), t_R (major) = 7.87 min, t_R (minor) = 11.19 min;

$[\alpha]_D^{25}$: 28.1 (c 1.0, $CHCl_3$);

^{1}H NMR (400 MHz, $CDCl_3$) δ 7.37 (d, *J* = 7.4 Hz, 1H), 7.30 – 7.20 (m, 2H), 7.13 (d, *J* = 7.5 Hz, 1H), 6.76 (dd, *J* = 10.0, 2.3 Hz, 1H), 2.60 (s, 3H), 2.58 (s, 3H), 1.91 (s, 1H), 1.74 (s, 3H);

^{13}C NMR (100 MHz, $CDCl_3$) δ 163.3 (d, J_{C-F} = 243.8 Hz), 152.0, 142.4 (d, J_{C-F} = 2.6 Hz), 142.0 (d, J_{C-F} = 9.4 Hz), 137.1 (d, J_{C-F} = 8.7 Hz), 135.5 (d, J_{C-F} = 3.0 Hz), 133.4, 131.4, 128.3, 120.5, 115.8 (d, J_{C-F} = 21.8 Hz), 108.1 (d, J_{C-F} = 23.9 Hz), 80.1, 25.1, 20.8, 18.1 (d, J_{C-F} = 1.5 Hz);

^{19}F NMR (376 MHz, $CDCl_3$) δ -114.7 (t).

(*S*)-**3-Bromo-1,5,9-trimethyl-9*H*-fluoren-9-ol** (**3an**)

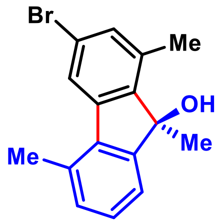

3an

Physical state: pale yellow solid;

Yield: 72% (21.9 mg);

$\boldsymbol{R_f}$ = 0.25 (PE:EtOAc = 10:1);

M.P.: 102 – 104 ºC;

HPLC: 97% *e.e.* (Daicel chiralpak AD-H column, 5% ^{i}PrOH in ^{n}hexane, 1 mL/min, λ = 230 nm), t_R (major) = 8.45 min, t_R (minor) = 10.19 min;

$[\alpha]_D^{25}$: 12.8 (c 1.0, $CHCl_3$);

^{1}**H NMR** (400 MHz, $CDCl_3$) δ 7.69 (d, *J* = 1.7 Hz, 1H), 7.35 (d, *J* = 7.4 Hz, 1H), 7.25 – 7.18 (m, 2H), 7.12 (d, *J* = 7.5 Hz, 1H), 2.61 (s, 3H), 2.55 (s, 3H), 1.94 (brs, 1H), 1.71 (s, 3H);

^{13}**C NMR** (100 MHz, $CDCl_3$) δ 151.4, 145.7, 142.1, 137.2, 135.3, 133.5, 132.3, 131.5, 128.4, 123.9, 122.6, 120.6, 80.2, 25.0, 21.0, 17.9;

HRMS (ESI-TOF) calculated for $C_{16}H_{15}BrO$, $[M+Na]^+$ 325.0199, found 325.0198.

(*S*)-**9-Hydroxy-*N*,1,5,9-tetramethyl-9*H*-fluorene-3-carboxamide** (**3ao**)[6]

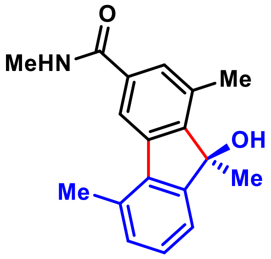

3ao

Physical state: pale yellow solid;

Yield: 73% (20.5 mg);

$\boldsymbol{R_f}$ = 0.2 (PE:EtOAc = 1:1);

M.P.: 134 – 136 ºC;

HPLC: 98% *e.e.* (Daicel chiralpak AD-H column, 10% ^{i}PrOH in ^{n}hexane, 1 mL/min, λ = 254 nm), t_R (major) = 14.69 min, t_R (minor) = 13.54 min;

$[\alpha]_D^{25}$: 13.1 (c 1.0, $CHCl_3$);

^{1}**H NMR** (400 MHz, $CDCl_3$, OH missing) δ 7.76 (s, 1H), 7.35 (d, *J* = 7.4 Hz, 1H), 7.27

(s, 1H), 7.19 (t, J = 7.5 Hz, 1H), 7.08 (d, J = 7.5 Hz, 1H), 6.47 (q, J = 5.1 Hz, 1H), 2.94 (d, J = 4.7 Hz, 3H), 2.54 (s, 3H), 2.51 (s, 3H), 1.66 (s, 3H);

^{13}C NMR (100 MHz, $CDCl_3$) δ 168.8, 151.5, 150.0, 140.5, 135.5, 135.2, 134.9, 133.4, 131.3, 128.3, 128.1, 120.5, 119.3, 80.1, 27.0, 25.1, 21.1, 18.0.

(*S*)-Methyl-9-hydroxy-1,5,9-trimethyl-9*H*-fluorene-3-carboxylate (3ap)[6]

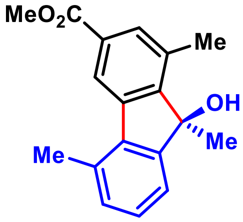

3ap

Physical state: pale yellow solid;

Yield: 56% (15.8 mg);

R$_f$ = 0.1 (PE:EtOAc = 10:1);

M.P.: 128 – 130 ºC;

HPLC: 97% *e.e.* (Daicel chiralpak AD-H column, 10% ^{i}PrOH in ^{n}hexane, 1 mL/min, λ = 254 nm), t_R (major) = 9.50 min, t_R (minor) = 8.68 min;

$[\alpha]_D^{25}$: 12.8 (c 1.0, $CHCl_3$);

^{1}H NMR (400 MHz, $CDCl_3$) δ 8.12 (s, 1H), 7.68 (s, 1H), 7.40 (d, J = 7.4 Hz, 1H), 7.23 (t, J = 7.6 Hz, 1H), 7.14 (d, J = 7.5 Hz, 1H), 3.82 (s, 3H), 2.67 (s, 3H), 2.63 (s, 3H), 2.25 (brs, 1H), 1.75 (s, 3H);

^{13}C NMR (101 MHz, $CDCl_3$) δ 167.4, 151.7, 151.2, 140.4, 135.6, 135.3, 133.6, 131.53, 131.47, 130.3, 128.3, 121.7, 120.6, 80.3, 52.3, 24.9, 21.1, 18.0.

(*S*)-1,5,9-Trimethyl-3-nitro-9*H*-fluoren-9-ol (3aq)[6]

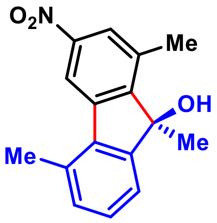

3aq

Physical state: yellow solid;

Yield: 48% (12.8 mg);

R$_f$ = 0.2 (PE:EtOAc = 10:1);

M.P.: 156 – 158 ºC;

HPLC: 98% *e.e.* (Daicel chiralpak AD-H column, 5% ^{i}PrOH in ^{n}hexane, 1 mL/min, λ

= 254 nm), t_R (major) = 11.71 min, t_R (minor) = 15.21 min;

$[\alpha]_D^{25}$: 12.6 (c 1.0, $CHCl_3$);

^{1}H NMR (400 MHz, $CDCl_3$) δ 8.33 (d, *J* = 2.1 Hz, 1H), 7.93 (d, *J* = 2.1 Hz, 1H), 7.39 (d, *J* = 7.4 Hz, 1H), 7.28 (t, *J* = 7.5 Hz, 1H), 7.18 (d, *J* = 7.5 Hz, 1H), 2.67 (s, 6H), 2.12 (brs, 1H), 1.74 (s, 3H);

^{13}C NMR (100 MHz, $CDCl_3$) δ 153.4, 151.3, 148.6, 141.5, 136.6, 134.4, 134.0, 131.9, 129.2, 124.9, 120.7, 115.4, 80.3, 24.9, 21.0, 18.2.

(*S*)-4-Fluoro-1,5,9-trimethyl-9*H*-fluoren-9-ol (3ar)

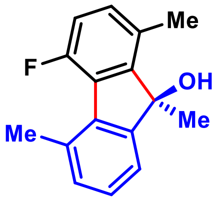

3ar

Physical state: white solid;

Yield: 64% (15.6 mg);

R_f = 0.3 (PE:EtOAc = 10:1);

M.P.: 93 – 95 ºC;

HPLC: 97% *e.e.* (Daicel chiralpak AD-H column, 5% ^{i}PrOH in ^{n}hexane, 1 mL/min, λ = 254 nm), t_R (major) = 7.49 min, t_R (minor) = 8.39 min;

$[\alpha]_D^{25}$: 22.0 (c 1.0, $CHCl_3$);

^{1}H NMR (400 MHz, $CDCl_3$) δ 7.35 (d, *J* = 7.3 Hz, 1H), 7.23 (t, *J* = 7.5 Hz, 1H), 7.16 (d, *J* = 7.5 Hz, 1H), 7.01 (dd, *J* = 8.4, 4.5 Hz, 1H), 6.93 (dd, *J* = 11.2, 8.3 Hz, 1H), 2.67 (d, *J* = 8.7 Hz, 3H), 2.57 (s, 3H), 1.93 (s, 1H), 1.73 (s, 3H);

^{13}C NMR (100 MHz, $CDCl_3$) δ 155.1 (d, $J_{C\text{-}F}$ = 248.0 Hz), 151.4, 149.0 (d, $J_{C\text{-}F}$ = 4.3 Hz), 134.5 (d, $J_{C\text{-}F}$ = 2.1 Hz), 133.9, 132.4, 131.9 (d, $J_{C\text{-}F}$ = 7.4 Hz), 130.9 (d, $J_{C\text{-}F}$ = 3.2 Hz), 128.5, 125.5 (d, $J_{C\text{-}F}$ = 15.5 Hz), 120.4, 116.6 (d, $J_{C\text{-}F}$ = 25.1 Hz), 80.4, 25.3, 23.0 (d, $J_{C\text{-}F}$ = 19.4 Hz), 17.5;

^{19}F NMR (376 MHz, $CDCl_3$) δ -110.7;

HRMS (ESI-TOF) calculated for $C_{16}H_{15}FO$, $[M+Na]^+$ 265.0999, found 265.1002.

(*S*)-**1-Chloro-2,3-dimethoxy-5,9-dimethyl-9*H*-fluoren-9-ol** (**3as**)

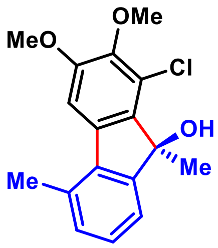

3as

Physical state: yellowish viscous liquid;

Yield: 48% (14.5 mg);

R_f = 0.4 (PE:EtOAc = 3:1);

HPLC: 98% *e.e.* (Daicel chiralpak IA column, 10% ^{i}PrOH in ^{n}hexane, 1 mL/min, λ = 230 nm), t_R (major) = 11.83 min, t_R (minor) = 10.52 min;

$[\alpha]_D^{25}$: -2.1 (c 1.0, $CHCl_3$);

^{1}**H NMR** (400 MHz, $CDCl_3$) δ 7.41 (d, J = 7.4 Hz, 1H), 7.27 (s, 1H), 7.22 (t, J = 7.5 Hz, 1H), 7.13 (d, J = 7.5 Hz, 1H), 3.96 (s, 3H), 3.90 (s, 3H), 2.64 (s, 3H), 2.48 (brs, 1H), 1.88 (s, 3H);

^{13}**C NMR** (100 MHz, $CDCl_3$) δ 154.2, 151.0, 144.9, 138.3, 137.3, 135.5, 132.6, 131.5, 128.0, 125.7, 120.8, 106.6, 80.7, 61.0, 56.6, 25.2, 20.8;

HRMS (ESI-TOF) calculated for $C_{17}H_{17}ClO_3$, $[M+Na]^+$ 327.0758, found 327.0761.

(*S*)-**7,11-Dimethyl-11*H*-benzo[*a*]fluoren-11-ol** (**3at**)[6]

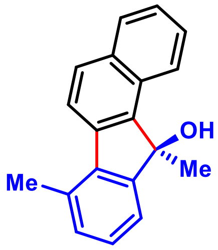

3at

Physical state: white solid;

Yield: 81% (21.1 mg);

R_f = 0.3 (PE:EtOAc = 10:1);

M.P.: 161 – 163 ºC;

HPLC: 97% *e.e.* (Daicel chiralpak AD-H column, 5% ^{i}PrOH in ^{n}hexane, 1 mL/min, λ = 320 nm), t_R (major) = 19.77 min, t_R (minor) = 17.35 min;

$[\alpha]_D^{25}$: 19.6 (c 1.0, $CHCl_3$);

^{1}**H NMR** (400 MHz, $CDCl_3$) δ 8.52 (d, J = 8.4 Hz, 1H), 7.99 (dd, J = 8.5, 1.4 Hz, 1H),

7.91 (dd, J = 11.4, 8.3 Hz, 2H), 7.63 – 7.55 (m, 1H), 7.54 – 7.45 (m, 2H), 7.25 (t, J = 7.5 Hz, 1H), 7.17 (d, J = 7.5 Hz, 1H), 2.75 (s, 3H), 2.08 (s, 1H), 1.90 (s, 3H);

^{13}C NMR (100 MHz, $CDCl_3$) δ 152.2, 144.3, 137.2, 136.8, 133.4, 132.9, 131.5, 129.7, 129.5, 129.0, 127.5, 126.6, 125.6, 124.8, 121.5, 120.4, 81.1, 27.0, 21.0.

(*S*)**-5-Bromo-7,11-dimethyl-11*H*-benzo[*a*]fluoren-11-ol (3au)**[6]

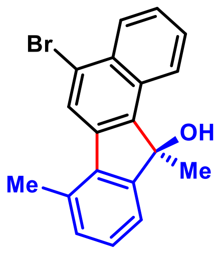

3au

Physical state: brown solid;

Yield: 73% (24.9 mg);

***R**$_f$* = 0.3 (PE:EtOAc = 10:1);

M.P.: 106 – 108 ºC;

HPLC: 97% *e.e.* (Daicel chiralpak AD-H column, 10% ^{i}PrOH in ^{n}hexane, 1 mL/min, λ = 230 nm), t_R (major) = 7.97 min, t_R (minor) = 10.37 min;

$[\alpha]_D^{25}$: 7.6 (c 1.0, $CHCl_3$);

^{1}H NMR (400 MHz, $CDCl_3$) δ 8.53 – 8.47 (m, 1H), 8.35 – 8.29 (m, 1H), 8.27 (s, 1H), 7.64 – 7.55(m, 2H), 7.43 (d, J = 7.4 Hz, 1H), 7.24 (t, J = 7.3 Hz, 1H), 7.14 (d, J = 7.5 Hz, 1H), 2.70 (s, 3H), 2.12 (brs, 1H), 1.83 (s, 3H);

^{13}C NMR (100 MHz, $CDCl_3$) δ 152.1, 144.1, 137.7, 135.6, 133.0, 131.6, 131.3, 130.4, 128.3, 128.0, 127.3, 126.9, 125.6, 125.1, 124.4, 120.4, 81.0, 27.2, 20.9.

(*S*)**-1-Methoxy-5,9-dimethyl-9*H*-indeno[2,1-*c*]pyridin-9-ol (3av)**[6]

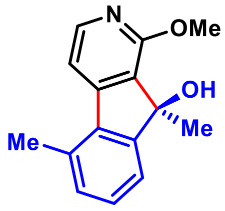

3av

Physical state: yellowish viscous liquid;

Yield: 75% (18.1 mg);

***R**$_f$* = 0.25 (PE:EtOAc = 3:1);

HPLC: 98% *e.e.* (Daicel chiralpak AD-H column, 20% ^{i}PrOH in ^{n}hexane, 1 mL/min, λ = 230 nm), t_R (major) = 9.66 min, t_R (minor) = 6.98 min;

$[\alpha]_D^{25}$: -28.2 (c 1.0, $CHCl_3$);

^{1}H NMR (400 MHz, $CDCl_3$) δ 8.18 (d, *J* = 5.3 Hz, 1H), 7.46 (d, *J* = 7.5 Hz, 1H), 7.36 – 7.29 (m, 2H), 7.17 (d, *J* = 7.5 Hz, 1H), 4.09 (s, 3H), 2.64 (s, 3H), 2.57 (brs, 1H), 1.82 (s, 3H);

^{13}C NMR (100 MHz, $CDCl_3$) δ 160.6, 150.9, 150.4, 147.8, 135.1, 134.6, 131.3, 129.9, 129.0, 121.0, 112.6, 79.3, 53.6, 24.9, 20.5.

(*S*)-1-Fluoro-5,9-dimethyl-9*H*-indeno[2,1-*c*]pyridin-9-ol (3aw)

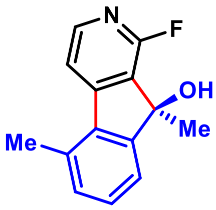

3aw

Physical state: pale yellow solid;

Yield: 50% (11.4 mg);

R_f = 0.25 (PE:EtOAc = 3:1);

M.P.: 102 – 104 ºC;

HPLC: 97% *e.e.* (Daicel chiralpak AD-H column, 20% ^{i}PrOH in ^{n}hexane, 1 mL/min, λ = 230 nm), t_R (major) = 7.16 min, t_R (minor) = 5.87 min;

$[\alpha]_D^{25}$: -40.8 (c 1.0, $CHCl_3$);

^{1}H NMR (400 MHz, $CDCl_3$) δ 8.20 (d, *J* = 5.2 Hz, 1H), 7.53 (dd, *J* = 5.2, 2.6 Hz, 1H), 7.47 (d, *J* = 7.5 Hz, 1H), 7.38 (t, *J* = 7.5 Hz, 1H), 7.22 (d, *J* = 7.5 Hz, 1H), 2.65 (s, 3H), 2.24 (brs, 1H), 1.85 (s, 3H);

^{13}C NMR (100 MHz, $CDCl_3$) δ 160.6 (d, $J_{C\text{-}F}$ = 239.6 Hz), 153.6 (d, $J_{C\text{-}F}$ = 7.2 Hz), 151.0, 148.7 (d, $J_{C\text{-}F}$ = 14.7 Hz), 135.0, 134.2 (d, $J_{C\text{-}F}$ = 3.2 Hz), 131.7, 130.8, 128.5 (d, $J_{C\text{-}F}$ = 30.3 Hz), 121.2, 116.5 (d, $J_{C\text{-}F}$ = 4.6 Hz), 78.9 (d, $J_{C\text{-}F}$ = 4.3 Hz), 25.0 (d, $J_{C\text{-}F}$ = 1.1 Hz), 20.6;

^{19}F NMR (376 MHz, $CDCl_3$) δ -74.8;

HRMS (ESI-TOF) calculated for $C_{14}H_{12}FNO$, $[M+H]^+$ 230.0976, found 230.0968.

(*S*)-**3-Chloro-1,5,9-trimethyl-9*H*-indeno[2,1-*c*]pyridin-9-ol** (**3ax**)

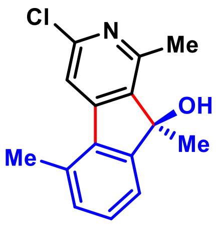

3ax

Physical state: pale yellow solid;

Yield: 56% (14.6 mg);

$\boldsymbol{R_f}$ = 0.3 (PE:EtOAc = 3:1);

M.P.: 156 – 158 ºC;

HPLC: 98% *e.e.* (Daicel chiralpak AD-H column, 10% ^{i}PrOH in ^{n}hexane, 1 mL/min, λ = 254 nm), t_R (major) = 6.09 min, t_R (minor) = 6.63 min;

$[\alpha]_D^{25}$: -18.6 (c 1.0, $CHCl_3$);

^{1}**H NMR** (400 MHz, $CDCl_3$) δ 7.43 (d, *J* = 7.7 Hz, 2H), 7.35 (t, *J* = 7.5 Hz, 1H), 7.18 (d, *J* = 7.4 Hz, 1H), 2.67 (s, 3H), 2.62 (s, 3H), 2.26 (brs, 1H), 1.72 (s, 3H);

^{13}**C NMR** (100 MHz, $CDCl_3$) δ 155.2, 152.4, 151.1, 150.6, 140.2, 135.3, 133.3, 131.7, 130.7, 121.0, 115.9, 79.3, 25.0, 21.1, 20.8;

HRMS (ESI-TOF) calculated for $C_{15}H_{14}ClNO$, $[M+H]^+$260.0837, found 260.0830.

(*S*)-**5-Benzyl-11-hydroxy-7,11-dimethyl-5,11-dihydro-6*H*-indeno[1,2-*c*]quinolin-6-one** (**3ay**)

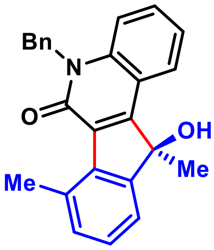

3ay

Physical state: pale yellow solid;

Yield: 61% (22.3 mg);

$\boldsymbol{R_f}$ = 0.2 (PE:EtOAc = 5:1);

M.P.: 86 – 88 ºC;

HPLC: 99% *e.e.* (Daicel chiralpak AD-H column, 20% ^{i}PrOH in ^{n}hexane, 1 mL/min, λ = 254 nm), t_R (major) = 11.85 min, t_R (minor) = 9.61 min;

$[\alpha]_D^{25}$: -129.4 (c 1.0, $CHCl_3$);

^{1}**H NMR** (400 MHz, $CDCl_3$) δ 8.47 (dd, *J* = 8.0, 1.8 Hz, 1H), 7.45 – 7.37 (m, 2H), 7.33 (t, *J* = 7.6 Hz, 1H), 7.22 (dt, *J* = 15.0, 7.1 Hz, 4H), 7.04 (d, *J* = 7.6 Hz, 2H), 6.99 (d, *J*

= 8.0 Hz, 2H), 5.64 (d, J = 16.6 Hz, 1H), 4.32 (s, 1H), 3.79 (s, 1H), 2.56 (s, 3H), 1.77 (s, 3H);

^{13}C NMR (100 MHz, $CDCl_3$) δ 159.3, 155.7, 151.7, 139.6, 136.4, 135.4, 134.5, 133.2, 130.0, 129.1, 128.8, 127.7, 127.3, 126.9, 126.4, 122.2, 119.6, 117.9, 115.7, 80.6, 45.7, 27.5, 23.9;

HRMS (ESI-TOF) calculated for $C_{25}H_{21}NO_2$, $[M+H]^+$368.1645, found 368.1646.

(*S*)-9-Butyl-1,5-dimethyl-9*H*-fluoren-9-ol (3ba)

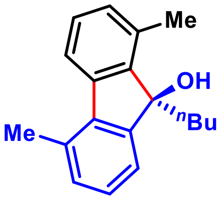

3ba

Physical state: pale yellow solid;

Yield: 81% (21.9 mg);

R_f = 0.35 (PE:EtOAc = 10:1);

M.P.: 116 – 118 ºC;

HPLC: 97% *e.e.* (Daicel chiralpak AD-H column, 10% ^{i}PrOH in ^{n}hexane, 1 mL/min, λ = 254 nm), t_R (major) = 5.31 min, t_R (minor) = 5.96 min;

$[\alpha]_D^{25}$: 11.3 (c 1.0, $CHCl_3$);

^{1}H NMR (400 MHz, $CDCl_3$) δ 7.60 (d, J = 7.6 Hz, 1H), 7.35 (d, J = 7.4 Hz, 1H), 7.26 (t, J = 7.6 Hz,1H), 7.20 (t, J = 7.5 Hz, 1H), 7.12 (d, J = 7.5 Hz, 1H), 7.05 (d, J = 7.6 Hz, 1H), 2.64 (s, 3H), 2.58 (s, 3H), 2.50 – 2.40 (m, 1H), 2.23 – 2.13 (m, 1H), 1.92 (s, 1H), 1.20 – 1.08 (m, 2H), 0.71 (t, J = 7.4 Hz, 3H), 0.68 – 0.56 (m, 2H);

^{13}C NMR (100 MHz, $CDCl_3$) δ 149.8, 145.6, 141.2, 137.7, 135.5, 132.9, 131.4, 129.9, 128.7, 127.6, 120.8, 120.6, 83.7, 37.5, 26.1, 22.9, 21.2, 18.2, 13.9;

HRMS (ESI-TOF) calculated for $C_{19}H_{22}O$, $[M+Na]^+$ 289.1563, found 289.1566.

(*S*)-9-Isopropyl-1,5-dimethyl-9*H*-fluoren-9-ol (3ca)

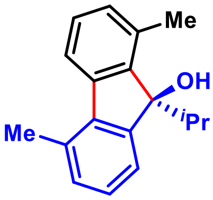

3ca

Physical state: colorless viscous liquid;

Yield: 83% (21.0 mg);

R_f = 0.4 (PE:EtOAc = 10:1);

HPLC: 96% *e.e.* (Daicel chiralpak AD-H column, 3% ^{i}PrOH in ^{n}hexane, 1 mL/min, λ = 230 nm), t_R (major) = 7.46 min, t_R (minor) = 6.89 min;

$[\alpha]_D^{25}$: 33.1 (c 1.0, $CHCl_3$);

^{1}H NMR (400 MHz, $CDCl_3$) δ 7.58 (d, J = 7.6 Hz, 1H), 7.40 (d, J = 7.1 Hz, 1H), 7.24 (t, J = 7.5 Hz, 1H), 7.20 – 7.09 (m, 2H), 7.04 (d, J = 7.6 Hz, 1H), 2.80 (p, J = 6.8 Hz, 1H), 2.64 (s, 3H), 2.60 (s, 3H), 1.95 (s, 1H), 1.29 (d, J = 6.8 Hz, 3H), 0.27 (d, J = 6.9 Hz, 3H);

^{13}C NMR (100 MHz, $CDCl_3$) δ 147.4, 147.1, 141.3, 138.3, 134.8, 132.8, 131.3, 130.0, 128.4, 126.6, 122.6, 120.6, 86.7, 34.3, 21.3, 18.3, 17.8, 17.2;

HRMS (ESI-TOF) calculated for $C_{18}H_{20}O$, $[M+Na]^+$ 275.1406, found 275.1408.

(*S*)**-9-(Tert-butyl)-1,5-dimethyl-9*H*-fluoren-9-ol** (**3da**)

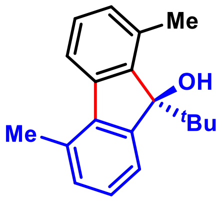

3da

Physical state: white solid;

Yield: 76% (20.3 mg);

R_f = 0.5 (PE:EtOAc = 10:1);

M.P.: 90 – 92 °C;

HPLC: 97% *e.e.* (Daicel chiralpak AD-H column, 5% ^{i}PrOH in ^{n}hexane, 1 mL/min, λ = 220 nm), t_R (major) = 6.38 min, t_R (minor) = 5.55 min;

$[\alpha]_D^{25}$: 29.6 (c 1.0, $CHCl_3$);

^{1}H NMR (400 MHz, $CDCl_3$) δ 7.57 (d, J = 7.6 Hz, 1H), 7.34 (dd, J = 6.3, 2.3 Hz, 1H), 7.23 (t, J = 7.6 Hz, 1H), 7.14 – 7.04 (m, 2H), 6.99 (d, J = 7.6 Hz, 1H), 2.62 (s, 3H), 2.61 (s, 3H), 1.97 (s, 1H), 0.92 (s, 9H);

^{13}C NMR (100 MHz, $CDCl_3$) δ 150.4, 147.2, 142.4, 138.1, 135.6, 132.2, 131.0, 129.8, 128.1, 125.7, 121.9, 120.2, 90.2, 40.6, 26.8, 21.4, 20.4;

HRMS (ESI-TOF) calculated for $C_{19}H_{22}O$, $[M+Na]^+$ 289.1563, found 289.1565.

(*S*)-**9-Cyclopropyl-1,5-dimethyl-9*H*-fluoren-9-ol** (**3ea**)

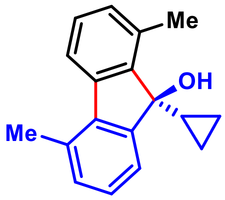

3ea

Physical state: pale yellow solid;

Yield: 82% (20.5 mg);

$\boldsymbol{R_f}$ = 0.4 (PE:EtOAc = 10:1);

M.P.: 84 – 86 ºC;

HPLC: 96% *e.e.* (Daicel chiralpak AD-H column, 2% ^{i}PrOH in ^{n}hexane, 1 mL/min, λ = 220 nm), t_R (major) = 13.97 min, t_R (minor) =12.96 min;

$[\alpha]_D^{25}$: 8.3 (c 1.0, $CHCl_3$);

^{1}H NMR (400 MHz, $CDCl_3$) δ 7.61 (d, *J* = 7.6 Hz, 1H), 7.37 (d, *J* = 7.2 Hz, 1H), 7.26 (t, *J* = 7.6 Hz, 1H), 7.17 (t, *J* = 7.4 Hz, 1H), 7.12 (d, *J* = 7.5 Hz, 1H), 7.08 (d, *J* = 7.6 Hz, 1H), 2.65 (s, 4H), 2.64 (s, 4H), 1.87 (s, 1H), 1.34 – 1.26 (m, 1H), 0.93 – 0.85 (m, 1H), 0.62 – 0.54 (m, 1H), 0.42 – 0.29 (m, 2H);

^{13}C NMR (100 MHz, $CDCl_3$) δ 149.6, 147.4, 140.4, 137.3, 135.5, 133.0, 131.4, 130.0, 128.6, 127.1, 121.6, 120.8, 81.9, 21.2, 19.1, 18.5, 3.4, 1.1;

HRMS (ESI-TOF) calculated for $C_{18}H_{18}O$, $[M+Na]^+$ 273.1250, found 273.1250.

(*S*)-**9-Cyclohexyl-1,5-dimethyl-9*H*-fluoren-9-ol** (**3fa**)

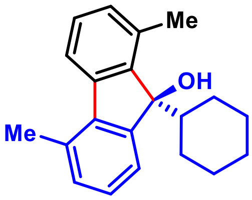

3fa

Physical state: colorless viscous liquid;

Yield: 82% (24.2 mg);

$\boldsymbol{R_f}$ = 0.4 (PE:EtOAc = 10:1);

HPLC: 96% *e.e.* (Daicel chiralpak AD-H column, 5% ^{i}PrOH in ^{n}hexane, 1 mL/min, λ = 230 nm), t_R (major) = 7.05 min, t_R (minor) = 8.39 min;

$[\alpha]_D^{25}$: -8.9 (c 1.0, $CHCl_3$);

^{1}H NMR (400 MHz, $CDCl_3$) δ 7.58 (d, *J* = 7.6 Hz, 1H), 7.39 (d, *J* = 7.0 Hz, 1H), 7.24 (t, *J* = 7.5 Hz, 1H), 7.15 (t, *J* = 7.4 Hz, 1H), 7.11 (d, *J* = 7.5 Hz, 1H), 7.04 (d, *J* = 7.6

Hz, 1H), 2.63 (s, 3H), 2.61 (s, 3H), 2.46 – 2.36 (m, 2H), 1.93 (s, 1H), 1.89 – 1.80 (m, 1H), 1.60 – 1.53 (m, 1H), 1.49 – 1.40 (m, 1H), 1.39 – 1.22 (m, 2H), 1.12 – 0.92 (m, 2H), 0.92 – 0.85 (m, 1H), 0.32 – 0.19 (m, 1H);

^{13}C NMR (100 MHz, $CDCl_3$) δ 148.3, 147.1, 141.3, 138.1, 134.9, 132.8, 131.2, 130.1, 128.4, 126.6, 122.6, 120.5, 86.5, 45.0, 27.8, 27.3, 26.9, 26.8, 26.6, 21.3, 18.4;

HRMS (ESI-TOF) calculated for $C_{21}H_{24}O$, $[M+Na]^+$ 315.1719, found 315.1718.

(*S*)**-1,5-Dimethyl-9-phenyl-9*H*-fluoren-9-ol** (**3ga**)

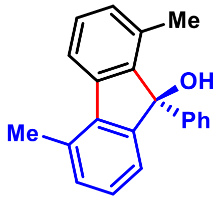

3ga

Physical state: yellowish viscous liquid;

Yield: 85% (24.3 mg);

R_f = 0.4 (PE:EtOAc = 10:1);

HPLC: 97% *e.e.* (Daicel chiralpak AD-H column, 10% ^{i}PrOH in ^{n}hexane, 1 mL/min, λ = 290 nm), t_R (major) = 7.21 min, t_R (minor) = 6.67 min;

$[\alpha]_D^{25}$: -215.2 (c 1.0, $CHCl_3$);

^{1}H NMR (400 MHz, $CDCl_3$) δ 7.68 (d, *J* = 7.7 Hz, 1H), 7.39 – 7.29 (m, 3H), 7.27 – 7.16 (m, 3H), 7.08 (s, 3H), 7.03 (d, *J* = 7.6 Hz, 1H), 2.68 (s, 3H), 2.26 (s, 1H), 2.11 (s, 3H);

^{13}C NMR (100 MHz, $CDCl_3$) δ 151.8, 147.7, 142.7, 141.5, 137.0, 136.2, 133.3, 131.4, 130.1, 129.4, 128.3, 128.1, 126.9, 125.3, 122.1, 120.9, 83.8, 21.1, 18.2;

HRMS (ESI-TOF) calculated for $C_{21}H_{18}O$, $[M+Na]^+$ 309.1250, found 309.1256.

(*S*)**-1,5-Dimethyl-9-(*o*-tolyl)-9*H*-fluoren-9-ol** (**3ha**)

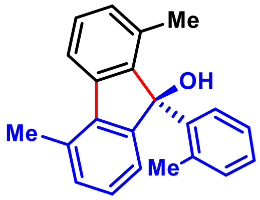

3ha

Physical state: white solid;

Yield: 77% (23.1 mg);

R_f = 0.5 (PE:EtOAc = 10:1);

M.P.: 154 – 156 °C;

HPLC: 98% *e.e.* (Daicel chiralpak AD-H column, 5% ^{i}PrOH in ^{n}hexane, 1 mL/min, λ = 254 nm), t_R (major) = 8.93 min, t_R (minor) = 7.68 min;

$[\alpha]_D^{25}$: -68.7 (c 1.0, $CHCl_3$);

^{1}H NMR (400 MHz, $CDCl_3$) δ 8.37 (d, *J* = 8.0 Hz, 1H), 7.69 (d, *J* = 7.6 Hz, 1H), 7.37 (t, *J* = 7.7 Hz, 1H), 7.31 (t, *J* = 7.6 Hz, 1H), 7.20 (t, *J* = 7.3 Hz, 1H), 7.15 – 7.08 (m, 2H), 7.03 – 6.90 (m, 3H), 2.70 (s, 3H), 2.20 (s, 1H), 2.00 (s, 3H), 1.29 (s, 3H);

^{13}C NMR (100 MHz, $CDCl_3$) δ 149.6, 146.5, 141.6, 140.3, 138.3, 135.9, 134.8, 133.2, 131.5, 131.4, 130.0, 129.3, 128.2, 127.3 127.1, 125.8, 121.9, 121.2, 82.7, 21.1, 19.4, 18.0;

HRMS (ESI-TOF) calculated for $C_{22}H_{20}O$, $[M+Na]^+$ 323.1406, found 323.1407.

(*S*)**-1,5-Dimethyl-9-(*m*-tolyl)-9*H*-fluoren-9-ol** (**3ia**)

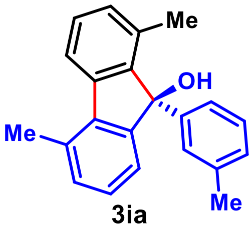

3ia

Physical state: colorless viscous liquid;

Yield: 83% (25.0 mg);

R$_f$ = 0.5 (PE:EtOAc = 10:1);

HPLC: 95% *e.e.* (Daicel chiralpak AD-H column, 2% ^{i}PrOH in ^{n}hexane, 1 mL/min, λ = 230 nm), t_R (major) = 14.35 min, t_R (minor) = 13.01 min;

$[\alpha]_D^{25}$: -170.4 (c 1.0, $CHCl_3$);

^{1}H NMR (400 MHz, $CDCl_3$) δ 7.69 (d, *J* = 7.6 Hz, 1H), 7.34 (t, *J* = 7.7 Hz, 1H), 7.22 (s, 1H), 7.17 – 7.12 (m, 2H), 7.12 – 7.08 (m, 3H), 7.07 – 7.00 (m, 2H), 2.70 (s, 3H), 2.29 (s, 3H), 2.28 (s, 1H), 2.14 (s, 3H);

^{13}C NMR (100 MHz, $CDCl_3$) δ 151.8, 147.7, 142.6, 141.5, 137.8, 137.0, 136.2, 133.2, 131.3, 130.1, 129.3, 128.2, 128.1, 127.7, 125.8, 122.3, 122.0, 120.8, 83.8, 21.7, 21.1, 18.2;

HRMS (ESI-TOF) calculated for $C_{22}H_{20}O$, $[M+Na]^+$ 323.1406, found 323.1410.

(*S*)-**1,5-Dimethyl-9-(*p*-tolyl)-9*H*-fluoren-9-ol** (**3ja**)

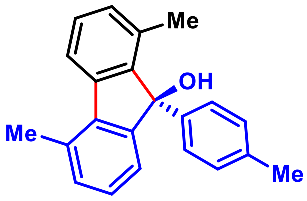

3ja

Physical state: yellowish viscous liquid;

Yield: 80% (23.9 mg);

$\boldsymbol{R_f}$ = 0.4 (PE:EtOAc = 10:1);

HPLC: 97% *e.e.* (Daicel chiralpak OD-H column, 3% ^{i}PrOH in ^{n}hexane, 1 mL/min, λ = 230 nm), t_R (major) = 8.43 min, t_R (minor) = 7.48 min;

$[\alpha]_D^{25}$: -185.8 (c 1.0, $CHCl_3$);

^{1}H NMR (400 MHz, $CDCl_3$) δ 7.68 (d, *J* = 7.7 Hz, 1H), 7.32 (t, *J* = 7.6 Hz, 1H), 7.28 – 7.22 (m, 2H), 7.12 – 7.06 (m, 4H), 7.04 (d, *J* = 7.6 Hz, 2H), 2.69 (s, 3H), 2.30 (s, 3H), 2.26 (brs, 1H), 2.13 (s, 3H);

^{13}C NMR (100 MHz, $CDCl_3$) δ 151.9, 147.8, 141.4, 139.7, 137.0, 136.4, 136.2, 133.2, 131.3, 130.1, 129.3, 129.0, 128.1, 125.1, 122.0, 120.8, 83.7, 21.2, 21.1, 18.2;

HRMS (ESI-TOF) calculated for $C_{22}H_{20}O$, $[M+Na]^+$ 323.1406, found 323.1405.

(*S*)-**1,5-Dimethyl-9-(*p*-tolyl)-9*H*-fluoren-9-ol** (**3ka**)

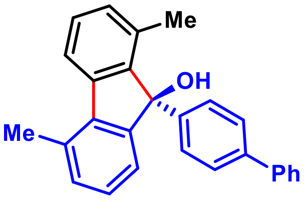

3ka

Physical state: yellowish viscous liquid;

Yield: 83% (30.2 mg);

$\boldsymbol{R_f}$ = 0.35 (PE:EtOAc = 10:1);

HPLC: 97% *e.e.* (Daicel chiralpak AD-H column, 10% ^{i}PrOH in ^{n}hexane, 1 mL/min, λ = 254 nm), t_R (major) = 8.43 min, t_R (minor) = 9.10 min;

$[\alpha]_D^{25}$: -219.3 (c 1.0, $CHCl_3$);

^{1}H NMR (400 MHz, $CDCl_3$) δ 7.71 (d, *J* = 7.7 Hz, 1H), 7.57 (d, *J* = 7.6 Hz, 2H), 7.49 (d, *J* = 8.3 Hz, 2H), 7.42 (dd, *J* = 14.3, 7.6 Hz, 4H), 7.34 (dt, *J* = 14.6, 7.4 Hz, 2H), 7.19 – 7.09 (m, 3H), 7.07 (d, *J* = 7.6 Hz, 1H), 2.71 (s, 3H), 2.33 (s, 1H), 2.18 (s, 3H);

^{13}C NMR (100 MHz, $CDCl_3$) δ 151.7, 147.6, 141.8, 141.4, 141.0, 139.6, 137.0, 136.3,

133.3, 131.4, 130.1, 129.4, 128.8, 128.1, 127.2, 127.1, 127.0, 125.7, 122.1, 120.9, 83.8, 21.1, 18.3;

HRMS (ESI-TOF) calculated for $C_{27}H_{22}O$, $[M+Na]^+$ 385.1563, found 385.1568.

(*S*)-1,5-Dimethyl-9-(4-(trifluoromethyl)phenyl)-9*H*-fluoren-9-ol (3la)

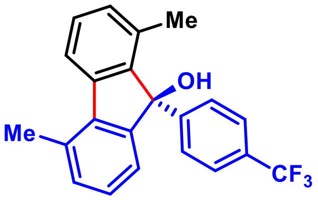

3la

Physical state: white solid;

Yield: 81% (28.6 mg);

***R**$_f$* = 0.3 (PE:EtOAc = 10:1);

M.P.: 93 – 95 ºC;

HPLC: 97% *e.e.* (Daicel chiralpak AD-H column, 10% ^{i}PrOH in ^{n}hexane, 1 mL/min, λ = 290 nm), t_R (major) = 4.92 min, t_R (minor) = 5.53 min;

$[\alpha]_D^{25}$: -167.8 (c 1.0, $CHCl_3$);

^{1}H NMR (400 MHz, $CDCl_3$) δ 7.70 (d, *J* = 7.7 Hz, 1H), 7.55 – 7.44 (m, 4H), 7.35 (t, *J* = 7.6 Hz, 1H), 7.15 – 7.08 (m, 2H), 7.08 – 7.02 (m, 2H), 2.70 (s, 3H), 2.34 (s, 1H), 2.09 (s, 3H);

^{13}C NMR (100 MHz, $CDCl_3$) δ 151.0, 147.13, 147.08 (q, J_{C-F} = 1.2 Hz), 141.4, 137.1, 136.1, 133.5, 131.7, 130.3, 129.8, 129.1 (q, J_{C-F} = 32.2 Hz), 128.2, 125.7, 125.3 (q, J_{C-F} = 3.7 Hz), 122.0, 121.7 (q, J_{C-F} = 271.9 Hz), 121.0, 83.6, 21.1, 18.1;

^{19}F NMR (376 MHz, $CDCl_3$) δ -62.4;

HRMS (ESI-TOF) calculated for $C_{22}H_{17}F_3O$, $[M+Na]^+$ 377.1124, found 377.1121.

(*S*)-9-(4-Methoxyphenyl)-1,5-dimethyl-9*H*-fluoren-9-ol (3ma)

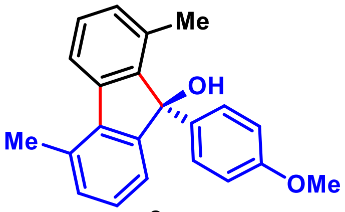

3ma

Physical state: colorless viscous liquid;

Yield: 85% (27.0 mg);

***R**$_f$* = 0.3 (PE:EtOAc = 10:1);

HPLC: 97% *e.e.* (Daicel chiralpak AD-H column, 2% ^{i}PrOH in ^{n}hexane, 1 mL/min, λ = 230 nm), t_R (major) = 21.72 min, t_R (minor) = 20.27 min;

$[\alpha]_D^{25}$: -157.9 (c 1.0, $CHCl_3$);

^{1}H NMR (400 MHz, $CDCl_3$) δ 7.67 (d, J = 7.7 Hz, 1H), 7.36 – 7.26 (m, 3H), 7.13 – 7.07 (m, 3H), 7.04 (d, J = 7.5 Hz, 1H), 6.81 – 6.75 (m, 2H), 3.76 (s, 3H), 2.68 (s, 3H), 2.30 (s, 1H), 2.14 (s, 3H);

^{13}C NMR (100 MHz, $CDCl_3$) δ 158.5, 151.9, 147.7, 141.3, 136.9, 136.2, 134.7, 133.2, 131.3, 130.1, 129.3, 128.1, 126.4, 121.9, 120.8, 113.6, 83.6, 55.3, 21.1, 18.1;

HRMS (ESI-TOF) calculated for $C_{22}H_{20}O_2$, $[M+Na]^+$ 339.1356, found 339.1357.

(*S*)-9-(4-Fluorophenyl)-1,5-dimethyl-9*H*-fluoren-9-ol (3na)

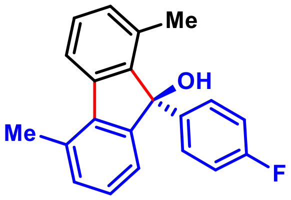

3na

Physical state: pale yellow liquid;

Yield: 86% (26.2 mg);

R_f = 0.4 (PE:EtOAc = 10:1);

HPLC: 96% *e.e.* (Daicel chiralpak AD-H column, 2% ^{i}PrOH in ^{n}hexane, 1 mL/min, λ = 220 nm), t_R (major) = 14.86 min, t_R (minor) =13.48 min;

$[\alpha]_D^{25}$: -171.1 (c 1.0, $CHCl_3$);

^{1}H NMR (400 MHz, $CDCl_3$) δ 7.68 (d, J = 7.7 Hz, 1H), 7.38 – 7.30 (m, 3H), 7.13 – 7.02 (m, 4H), 6.97 – 6.89 (m, 2H), 2.69 (s, 3H), 2.29 (s, 1H), 2.12 (s, 3H);

^{13}C NMR (100 MHz, $CDCl_3$) δ 161.9 (d, $J_{C\text{-}F}$ = 244.7 Hz), 151.5, 147.4, 141.3, 138.5 (d, $J_{C\text{-}F}$ = 3.0 Hz), 136.9, 136.2, 133.3, 131.5, 130.2, 129.5, 128.2, 127.0 (d, $J_{C\text{-}F}$ = 8.0 Hz), 121.9, 120.9, 115.1 (d, $J_{C\text{-}F}$ = 21.4 Hz), 83.4, 21.1, 18.1;

^{19}F NMR (376 MHz, $CDCl_3$) δ -116.3 – -116.4 (m);

HRMS (ESI-TOF) calculated for $C_{21}H_{17}FO$, $[M+Na]^+$ 327.1156, found 327.1159.

(*S*)-9-(4-Chlorophenyl)-1,5-dimethyl-9*H*-fluoren-9-ol (3oa)

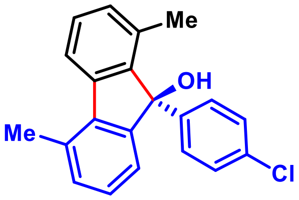

3oa

Physical state: yellowish viscous liquid;

Yield: 87% (28.0 mg);

$\boldsymbol{R_f}$ = 0.45 (PE:EtOAc = 10:1);

HPLC: 96% *e.e.* (Daicel chiralpak AD-H column, 10% ^{i}PrOH in ^{n}hexane, 1 mL/min, λ = 230 nm), t_R (major) = 6.12 min, t_R (minor) = 6.49 min;

$[\alpha]_D^{25}$: -210.1 (c 1.0, $CHCl_3$);

^{1}**H NMR** (400 MHz, $CDCl_3$) δ 7.68 (d, *J* = 7.7 Hz, 1H), 7.36 – 7.28 (m, 3H), 7.24 – 7.19 (m, 2H), 7.11 (d, *J* = 4.6 Hz, 2H), 7.08 – 7.02 (m, 2H), 2.69 (s, 3H), 2.27 (brs, 1H), 2.11 (s, 3H);

^{13}**C NMR** (100 MHz, $CDCl_3$) δ 151.3, 147.2, 141.43, 141.36, 137.0, 136.2, 133.4, 132.6, 131.6, 130.2, 129.6, 128.4, 128.2, 126.8, 122.0, 120.9, 83.4, 21.1, 18.1;

HRMS (ESI-TOF) calculated for $C_{21}H_{17}ClO$, $[M+Na]^+$ 343.0860, found 343.0861.

(*S*)**-9-(Furan-3-yl)-1,5-dimethyl-9*H*-fluoren-9-ol** (**3pa**)

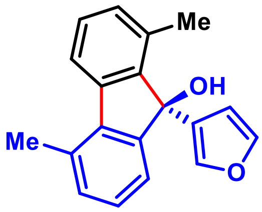

3pa

Physical state: pale yellow solid;

Yield: 52% (14.3 mg);

$\boldsymbol{R_f}$ = 0.35 (PE:EtOAc = 10:1);

M.P.: 122 – 124 °C;

HPLC: 96% *e.e.* (Daicel chiralpak AD-H column, 10% ^{i}PrOH in ^{n}hexane, 1 mL/min, λ = 230 nm), t_R (major) = 8.43 min, t_R (minor) = 7.76 min;

$[\alpha]_D^{25}$: -155.0 (c 1.0, $CHCl_3$);

^{1}**H NMR** (400 MHz, $CDCl_3$) δ 7.63 (d, *J* = 7.7 Hz, 1H), 7.54 (s, 1H), 7.30 (t, *J* = 7.7 Hz, 1H), 7.27 – 7.23 (m, 2H), 7.14 (t, *J* = 7.4 Hz, 1H), 7.10 (d, *J* = 7.4 Hz, 1H), 7.04 (d, *J* = 7.6 Hz, 1H), 5.96 (d, *J* = 1.6 Hz, 1H), 2.66 (s, 3H), 2.31 (s, 3H), 2.21 (s, 1H);

^{13}**C NMR** (100 MHz, $CDCl_3$) δ 150.3, 145.9, 143.2, 140.8, 139.8, 136.7, 136.4, 133.2, 131.6, 130.1, 129.5, 128.2, 128.0, 121.8, 120.9, 108.8, 80.4, 21.1, 17.9;

HRMS (ESI-TOF) calculated for $C_{19}H_{16}O_2$, $[M+Na]^+$ 299.1043, found 299.1039.

(*S*)-**1,5-Dimethyl-9-(5-methylthiophen-2-yl)-9*H*-fluoren-9-ol** (**3qa**)

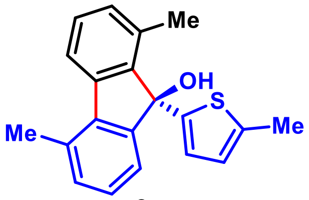

3qa

Physical state: brown viscous liquid;

Yield: 48% (14.6 mg);

R$_f$ = 0.4 (PE:EtOAc = 10:1);

HPLC: 97% *e.e.* (Daicel chiralpak AD-H column, 10% ^{i}PrOH in ^{n}hexane, 1 mL/min, λ = 254 nm), t_R (major) = 7.97 min, t_R (minor) = 9.00 min;

$[\alpha]_D^{25}$: -313.2 (c 1.0, $CHCl_3$);

^{1}H NMR (400 MHz, $CDCl_3$) δ 7.65 (d, *J* = 7.6 Hz, 1H), 7.37 – 7.30 (m, 2H), 7.14 (t, *J* = 7.4 Hz, 1H), 7.09 (dd, *J* = 7.5, 4.9 Hz, 2H), 6.57 (d, *J* = 3.5 Hz, 1H), 6.50 (d, *J* = 3.3 Hz, 1H), 2.66 (s, 3H), 2.45 (s, 1H), 2.41 (s, 3H), 2.33 (s, 3H);

^{13}C NMR (100 MHz, $CDCl_3$) δ 150.7, 146.4, 144.6, 141.0, 138.6, 136.6, 136.3, 133.3, 131.5, 130.2, 129.6, 128.0, 124.8, 122.9, 121.7, 120.9, 82.7, 21.1, 18.3, 15.5;

HRMS (ESI-TOF) calculated for $C_{20}H_{18}OS$, $[M+Na]^+$ 329.0971, found 329.0971.

(*S*)-**5-Isopropyl-1,9-dimethyl-9*H*-fluoren-9-ol** (**3ra**)

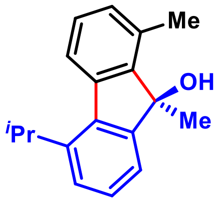

3ra

Physical state: white solid;

Yield: 78% (19.6 mg);

R$_f$ = 0.4 (PE:EtOAc = 10:1);

M.P.: 156 – 158 ºC;

HPLC: 97% *e.e.* (Daicel chiralpak AD-H column, 5% ^{i}PrOH in ^{n}hexane, 1 mL/min, λ = 230 nm), t_R (major) = 7.68 min, t_R (minor) = 8.30 min;

$[\alpha]_D^{25}$: -7.9 (c 1.0, $CHCl_3$);

^{1}H NMR (400 MHz, $CDCl_3$) δ 7.62 (d, *J* = 7.7 Hz, 1H), 7.34 (dd, J = 6.6, 2.0 Hz, 1H), 7.29 – 7.18 (m, 3H), 7.02 (d, *J* = 7.5 Hz, 1H), 3.74 – 3.62 (m, 1H), 2.58 (s, 3H), 1.90 (s, 1H), 1.72 (s, 3H), 1.34 (dd, *J* = 6.8, 4.7 Hz, 6H);

^{13}C NMR (100 MHz, $CDCl_3$) δ 151.5, 147.1, 144.4, 139.7, 135.4, 135.2, 129.9, 128.8,

128.1, 125.4, 121.4, 120.5, 80.3, 29.5, 25.1, 23.0, 22.9, 18.2;

HRMS (ESI-TOF) calculated for $C_{18}H_{20}O$, $[M+Na]^+$ 275.1406, found 275.1410.

(*S*)-1,9-Dimethyl-5-phenyl-9*H*-fluoren-9-ol (3sa)

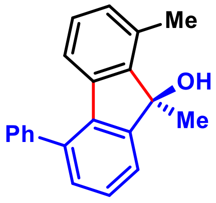

3sa

Physical state: yellowish viscous liquid;

Yield: 89% (25.6 mg);

R_f = 0.3 (PE:EtOAc = 10:1);

HPLC: 95% *e.e.* (Daicel chiralpak AD-H column, 10% ^{i}PrOH in ^{n}hexane, 1 mL/min, λ = 230 nm), t_R (major) = 6.32 min, t_R (minor) = 7.22 min;

$[\alpha]_D^{25}$: 39.2 (c 1.0, $CHCl_3$);

^{1}H NMR (400 MHz, $CDCl_3$) δ 7.58 – 7.37 (m, 6H), 7.33 (t, *J* = 7.5 Hz, 1H), 7.19 (d, *J* = 7.5 Hz, 1H), 6.95 (dt, *J* = 15.0, 7.6 Hz, 2H), 6.65 (d, *J* = 7.4 Hz, 1H), 2.60 (s, 3H), 2.07 (s, 1H), 1.84 (s, 3H);

^{13}C NMR (100 MHz, $CDCl_3$) δ 151.6, 146.9, 141.0, 139.1, 137.9, 135.5, 135.3, 130.9, 130.2, 129.4, 128.9, 128.7, 128.5, 128.4, 127.7, 127.5, 122.0, 120.7, 80.5, 77.5, 25.0, 18.1;

HRMS (ESI-TOF) calculated for $C_{21}H_{18}O$, $[M+Na]^+$ 309.1250, found 309.1253.

(*S*)-5-Methoxy-1,9-dimethyl-9*H*-fluoren-9-ol (3ta)

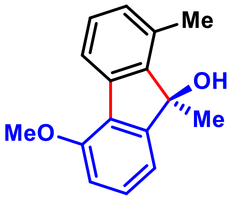

3ta

Physical state: yellowish viscous liquid;

Yield: 82% (19.6 mg);

R_f = 0.2 (PE:EtOAc = 10:1);

HPLC: 93% *e.e.* (Daicel chiralpak AD-H column, 5% ^{i}PrOH in ^{n}hexane, 1 mL/min, λ = 220 nm), t_R (major) = 12.16 min, t_R (minor) =15.36 min;

$[\alpha]_D^{25}$: 44.1 (c 1.0, $CHCl_3$);

^{1}**H NMR** (400 MHz, $CDCl_3$) δ 7.84 (d, *J* = 7.5 Hz, 1H), 7.30 – 7.20 (m, 2H), 7.14 (d, *J* = 7.5 Hz, 1H), 7.02 (d, *J* = 7.6 Hz, 1H), 6.87 (d, *J* = 8.1 Hz, 1H), 3.97 (s, 3H), 2.59 (s, 3H), 1.96 (brs, 1H), 1.77 (s, 3H);

^{13}**C NMR** (100 MHz, $CDCl_3$) δ 155.7, 152.7, 145.9, 138.4, 134.7, 129.6, 129.1, 128.8, 126.3, 121.6, 115.1, 111.0, 81.2, 55.5, 24.8, 18.0;

HRMS (ESI-TOF) calculated for $C_{16}H_{16}O_2$, $[M+Na]^+$ 263.1043, found 263.1042.

(*S*)**-5-Chloro-1-methyl-9-phenyl-9*H*-fluoren-9-ol** (**3ua**)

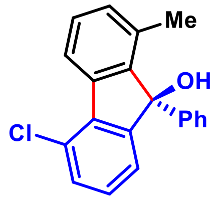

3ua

Physical state: pale yellow solid;

Yield: 77% (23.5 mg);

R_f = 0.4 (PE:EtOAc = 10:1);

M.P.: 80 – 82 ºC;

HPLC: 96% *e.e.* (Daicel chiralpak AD-H column, 5% ^{i}PrOH in ^{n}hexane, 1 mL/min, λ = 230 nm), t_R (major) = 8.54 min, t_R (minor) = 9.40 min;

$[\alpha]_D^{25}$: -236.5 (c 1.0, $CHCl_3$);

^{1}**H NMR** (400 MHz, $CDCl_3$) δ 8.22 (d, *J* = 7.7 Hz, 1H), 7.39 – 7.31 (m, 3H), 7.29 – 7.18 (m, 4H), 7.16 – 7.05 (m, 3H), 2.29 (brs, 1H), 2.09 (s, 3H);

^{13}**C NMR** (100 MHz, $CDCl_3$) δ 153.8, 147.3, 141.9, 139.1, 136.1, 135.9, 131.2, 130.5, 129.6, 129.1, 128.8, 128.4, 127.2, 125.2, 122.9, 121.6, 83.9, 18.0;

HRMS (ESI-TOF) calculated for $C_{20}H_{15}ClO$, $[M+Na]^+$ 329.0704, found 329.0705.

(*S*)**-7-Chloro-1,5,9-trimethyl-9*H*-fluoren-9-ol** (**3va**)

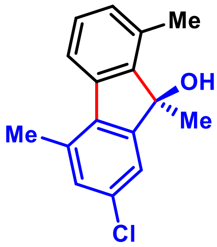

3va

Physical state: pale yellow solid;

Yield: 79% (20.4 mg);

***R**$_f$* = 0.4 (PE:EtOAc = 10:1);

M.P.: 88 – 90 ºC;

HPLC: 97% *e.e.* (Daicel chiralpak AD-H column, 5% ^{i}PrOH in ^{n}hexane, 1 mL/min, λ = 220 nm), t_R (major) = 10.88 min, t_R (minor) = 11.95 min;

$[\alpha]_D^{25}$: 15.5 (c 1.0, $CHCl_3$);

^{1}H NMR (400 MHz, $CDCl_3$) δ 7.53 (d, *J* = 7.6 Hz, 1H), 7.32 (d, *J* = 1.9 Hz, 1H), 7.25 (t, *J* = 7.6 Hz, 1H), 7.10 (s, 1H), 7.06 (d, *J* = 7.7 Hz, 1H), 2.582 (s, 3H), 2.576 (s, 3H), 1.95 (s, 1H), 1.72 (s, 3H);

^{13}C NMR (100 MHz, $CDCl_3$) δ 153.0, 146.6, 139.2, 135.5, 135.0, 134.7, 133.1, 131.1, 130.2, 128.9, 121.0, 120.8, 80.5, 24.9, 20.9, 18.0;

HRMS (ESI-TOF) calculated for $C_{16}H_{15}ClO$, $[M+Na]^+$ 281.0704, found 281.0705.

(*S*)-**1-Methyl-5-(morpholinomethyl)-9-phenyl-9*H*-fluoren-9-ol** (**3wa**)

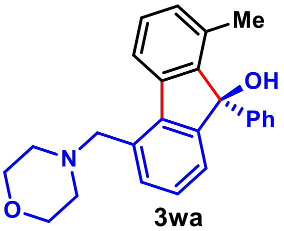

3wa

Physical state: white solid;

Yield: 60% (22.3 mg);

***R**$_f$* = 0.5 (PE:EtOAc = 2:1);

M.P.: 159 – 161 ºC;

HPLC: 98% *e.e.* (Daicel chiralpak OD-H column, 10% ^{i}PrOH in ^{n}hexane, 1 mL/min, λ = 230 nm), t_R (major) = 7.01 min, t_R (minor) = 5.80 min;

$[\alpha]_D^{25}$: -219.2 (c 1.0, $CHCl_3$);

^{1}H NMR (400 MHz, $CDCl_3$) δ 7.67 (d, *J* = 7.7 Hz, 1H), 7.39 – 7.33 (m, 2H), 7.31 (t, *J* = 7.6 Hz, 1H), 7.23 (t, *J* = 7.6 Hz, 2H), 7.20 – 7.11 (m, 3H), 7.08 (d, *J* = 7.5 Hz, 1H), 7.05 (d, *J* = 7.4 Hz, 1H), 3.90 (d, *J* = 12.8 Hz, 1H), 3.55 (d, *J* = 12.7 Hz, 1H), 3.48 – 3.38 (m, 2H), 3.35 (s, 1H), 3.33 – 3.25 (m, 2H), 2.52 – 2.32 (m, 4H), 2.12 (s, 3H);

^{13}C NMR (100 MHz, $CDCl_3$) δ 152.9, 148.1, 142.8, 140.6, 138.1, 135.9, 132.5, 131.3, 130.2, 129.1, 128.2, 127.6, 126.8, 125.3, 123.8, 122.3, 83.5, 66.6, 61.6, 53.5, 18.1;

HRMS (ESI-TOF) calculated for $C_{25}H_{25}NO_2$, $[M+H]^+$ 372.1958, found 372.1960.

(*S*)-**5,6,7-Trimethoxy-1,9-dimethyl-9*H*-fluoren-9-ol** (**3xa**)

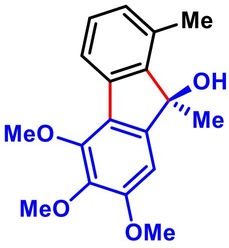

3xa

Physical state: yellow solid;

Yield: 64% (19.1 mg);

R_f = 0.5 (PE:EtOAc = 2:1);

M.P.: 76 – 78 ºC;

HPLC: 93% *e.e.* (Daicel chiralpak AD-H column, 5% ^{i}PrOH in ^{n}hexane, 1 mL/min, λ = 220 nm), t_R (major) = 16.08 min, t_R (minor) = 14.58 min;

$[\alpha]_D^{25}$: 3.2 (c 1.0, $CHCl_3$);

^{1}H NMR (400 MHz, $CDCl_3$) δ 7.70 (d, *J* = 7.5 Hz, 1H), 7.22 (t, *J* = 7.6 Hz, 1H), 6.98 (d, *J* = 7.5 Hz, 1H), 6.88 (s, 1H), 4.00 (s, 3H), 3.93 (s, 3H), 3.90 (s, 3H), 2.57 (s, 3H), 1.98 (brs, 1H), 1.75 (s, 3H);

^{13}C NMR (100 MHz, $CDCl_3$) δ 154.1, 149.3, 146.7, 145.9, 142.7, 138.2, 134.8, 129.2, 129.1, 123.8, 120.2, 102.4, 81.2, 61.1, 60.7, 56.4, 25.0, 17.9;

HRMS (ESI-TOF) calculated for $C_{18}H_{20}O_4$, $[M+Na]^+$ 323.1254, found 323.1255.

(*S*)-**7,8-Dimethyl-7*H*-benzo[*c*]fluoren-7-ol** (**3ya**)

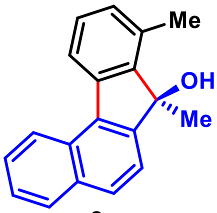

3ya

Physical state: pale yellow solid;

Yield: 74% (19.3 mg);

R_f = 0.2 (PE:EtOAc = 10:1);

M.P.: 132 – 134 ºC;

HPLC: 96% *e.e.* (Daicel chiralpak AD-H column, 7% ^{i}PrOH in ^{n}hexane, 1 mL/min, λ = 230 nm), t_R (major) = 13.13 min, t_R (minor) = 15.36 min;

$[\alpha]_D^{25}$: 83.3 (c 1.0, $CHCl_3$);

^{1}H NMR (400 MHz, $CDCl_3$) δ 8.63 (d, *J* = 8.5 Hz, 1H), 8.05 (d, *J* = 7.7 Hz, 1H), 7.92 (dd, *J* = 8.2, 1.3 Hz, 1H), 7.82 (d, *J* = 8.3 Hz, 1H), 7.65 (d, *J* = 8.4 Hz, 1H), 7.63 – 7.58

(m, 1H), 7.56 – 7.48 (m, 1H), 7.33 (t, *J* = 7.7 Hz, 1H), 7.08 (d, *J* = 7.6 Hz, 1H), 2.63 (s, 3H), 1.99 (s, 1H), 1.81 (s, 3H);

^{13}C NMR (100 MHz, $CDCl_3$) δ 149.3, 147.4, 140.3, 135.2, 134.7, 132.9, 129.8, 129.4, 129.3, 129.2, 128.9, 127.0, 125.7, 124.2, 120.8, 120.7, 80.7, 24.3, 18.1;

HRMS (ESI-TOF) calculated for $C_{19}H_{16}O$, $[M+Na]^+$ 283.1093, found 283.1091.

5. Desymmetrization of symmetric secondary dialcohols and the reaction of ketone with aryl iodide

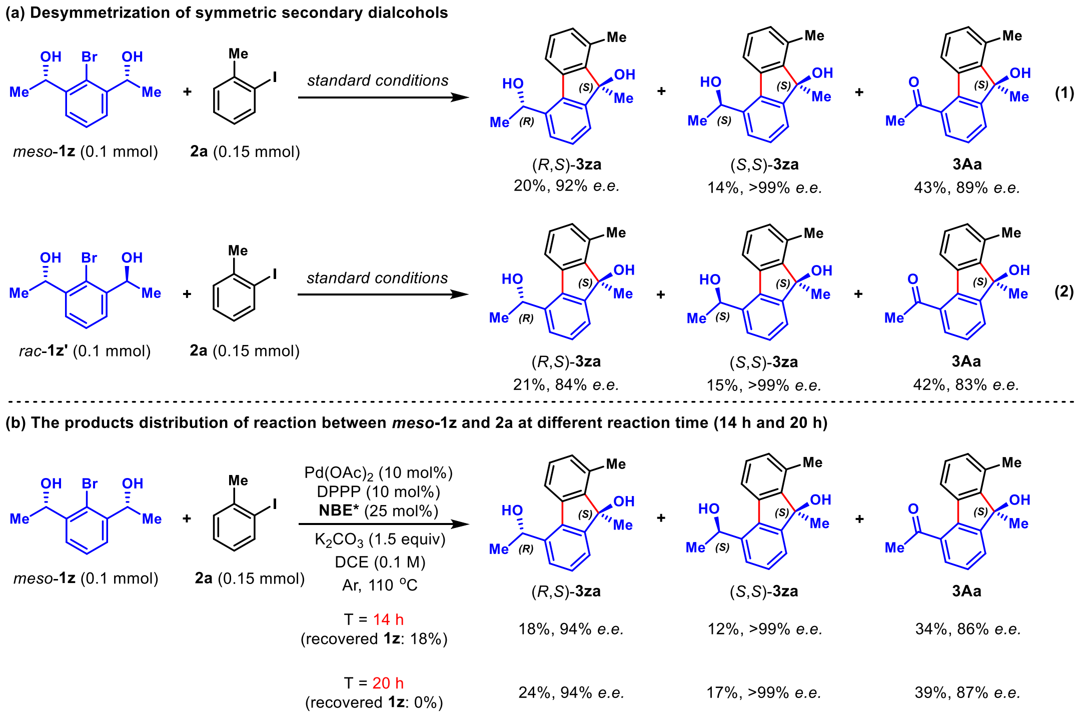

Figure S1. (a) Desymmetrization of symmetric secondary dialcohols; (b) The products distribution of reaction between *meso*-**1z** and **2a** at different reaction time (14 h and 20 h).

(*R,S*)-5-(1-Hydroxyethyl)-1,9-dimethyl-9*H*-fluoren-9-ol ((*R,S*)-3za)

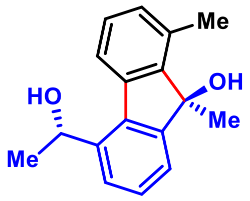

(*R,S*)-**3za**

Physical state: pale yellow solid;

Yield: 21% (5.4 mg);

R$_f$ = 0.3 (DCM:MeOH = 25:1);

M.P.: 206 – 208 ºC;

HPLC: 92% *e.e.* (Daicel chiralpak AD-H column, 15% ^{i}PrOH in ^{n}hexane, 1 mL/min, λ = 290 nm), t_R (major) = 12.99 min, t_R (minor) = 10.69 min;

$[\alpha]_D^{25}$: 66.5 (c 1.0, MeOH);

^{1}H NMR (400 MHz, MeOH-*d$_4$*, OH missing) δ 7.59 (dd, *J* = 7.8, 4.3 Hz, 2H), 7.44 (dd, *J* = 7.5, 1.2 Hz, 1H), 7.32 (t, *J* = 7.6 Hz, 1H), 7.25 (t, *J* = 7.7 Hz, 1H), 7.07 (d, *J* = 7.6 Hz, 1H), 5.59 (q, *J* = 6.4 Hz, 1H), 2.61 (s, 3H), 1.73 (s, 3H), 1.54 (d, *J* = 6.5 Hz, 3H);

^{13}C NMR (100 MHz, MeOH-*d$_4$*) δ 153.3, 148.6, 143.1, 140.3, 136.3, 135.5, 130.9, 129.5, 128.8, 125.7, 122.7, 122.4, 80.7, 67.2, 26.0, 24.4, 18.3;

HRMS (ESI-TOF) calculated for $C_{17}H_{18}O_2$, $[M+Na]^+$ 277.1199, found 277.1195.

(*S*,*S*)**-5-(1-Hydroxyethyl)-1,9-dimethyl-9*H*-fluoren-9-ol** ((*S*,*S*)-**3za**)

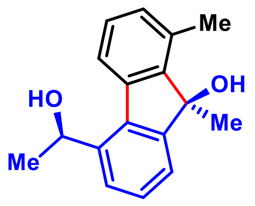

(*S*,*S*)-**3za**

Physical state: pale yellow solid;

Yield: 15% (3.8 mg);

R$_f$ = 0.4 (DCM:MeOH = 25:1);

M.P.: 228 – 230 ºC;

HPLC: >99% *e.e.* (Daicel chiralpak AD-H column, 15% ^{i}PrOH in ^{n}hexane, 1 mL/min, λ = 290 nm), t_R (major) = 9.03 min, t_R (minor) = 14.20 min;

$[\alpha]_D^{25}$: -40.2 (c 1.0, MeOH);

^{1}H NMR (400 MHz, MeOH-*d$_4$*, OH missing) δ 7.58 (dd, *J* = 7.4, 2.2 Hz, 2H), 7.44 (dd, *J* = 7.4, 1.1 Hz, 1H), 7.32 (t, *J* = 7.6 Hz, 1H), 7.25 (t, *J* = 7.7 Hz, 1H), 7.06 (d, *J* = 7.6 Hz, 1H), 5.62 (q, *J* = 6.4 Hz, 1H), 2.61 (s, 3H), 1.72 (s, 3H), 1.53 (d, *J* = 6.4 Hz, 3H);

^{13}C NMR (100 MHz, MeOH-*d$_4$*) δ 153.2, 148.5, 143.1, 140.3, 136.4, 135.6, 130.9, 129.5, 128.8, 125.7, 122.7, 122.4, 80.7, 67.1, 25.9, 24.2, 18.3;

HRMS (ESI-TOF) calculated for $C_{17}H_{18}O_2$, $[M+Na]^+$ 277.1199, found 277.1195.

(*S*)-**1-(9-Hydroxy-8,9-dimethyl-9*H*-fluoren-4-yl)ethan-1-one (3Aa)**

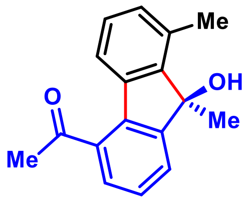

3Aa

Physical state: yellowish viscous liquid;

Yield: 43% (10.9 mg);

R_f = 0.6 (DCM:MeOH = 25:1);

HPLC: 89% *e.e.* (Daicel chiralpak AD-H column, 10% ^{i}PrOH in ^{n}hexane, 1 mL/min, λ = 230 nm), t_R (major) = 9.24 min, t_R (minor) = 10.55 min;

$[\alpha]_D^{25}$: 312.6 (c 1.0, $CHCl_3$);

^{1}H NMR (400 MHz, $CDCl_3$) δ 7.64 (dt, *J* = 7.8, 1.8 Hz, 2H), 7.47 (dd, *J* = 7.7, 1.2 Hz, 1H), 7.34 (t, *J* = 7.6 Hz, 1H), 7.20 (t, *J* = 7.7 Hz, 1H), 7.08 (d, *J* = 7.5 Hz, 1H), 2.68 (s, 3H), 2.60 (s, 3H), 1.95 (brs, 1H), 1.79 (s, 3H);

^{13}C NMR (100 MHz, $CDCl_3$) δ 203.5, 152.8, 147.0, 137.5, 136.1, 135.6, 135.3, 131.3, 128.9, 128.1, 127.5, 125.6, 121.6, 80.2, 30.5, 24.8, 18.1;

HRMS (ESI-TOF) calculated for $C_{17}H_{16}O_2$, $[M+Na]^+$ 275.1043, found 275.1043.

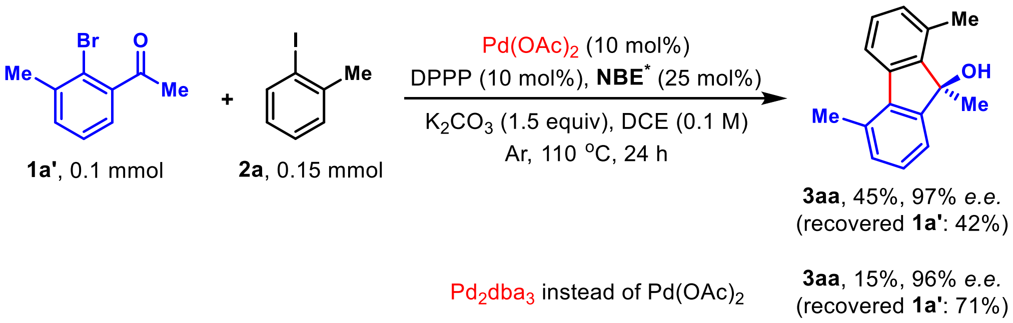

Figure S2. The reaction of *ortho*-bromoacetophenone **1a'** with aryl iodide **2a**. dba: dibenzylideneacetone.

6. X-ray crystallographic data

Table S6. Crystal data and structure refinement for 3ha

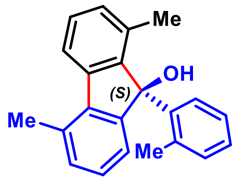 3ha	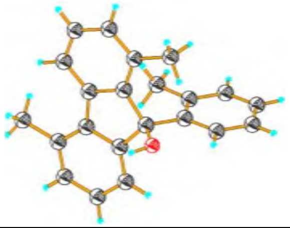
Identification code	CCDC 2222332
Empirical formula	$C_{22}H_{20}O$
Formula weight	300.38
Temperature	173 K
Wavelength	1.54178 Å
Crystal system	Orthorhombic
Space group	P212121
Unit cell dimensions	a = 7.7777(3) Å α = 90°<br>b = 8.0764(3) Å β = 90°<br>c = 25.7905(9) Å γ = 90°
Volume	1620.05(10) $Å^3$
Z	4
Density (calculated)	1.232 Mg/m3
Absorption coefficient	0.567 mm^{-1}
F(000)	640
Crystal size	0.04 x 0.04 x 0.02 mm^3
Theta range for data collection	3.427 to 65.219°
Index ranges	-9<=h<=9, -9<=k<=9, -30<=l<=29
Reflections collected	19056
Independent reflections	2770 [R(int) = 0.0636]
Completeness to theta = 65.219°	99.9 %
Absorption correction	None
Refinement method	Full-matrix least-squares on F^2
Data / restraints / parameters	2770 / 0 / 216
Goodness-of-fit on F^2	1.051
Final R indices [I>2sigma(I)]	R1 = 0.0350, wR2 = 0.0874
R indices (all data)	R1 = 0.0352, wR2 = 0.0881
Absolute structure parameter	-0.06(8)
Extinction coefficient	0.0078(9)
Largest diff. peak and hole	0.189 and -0.184 e.$Å^{-3}$

Table S7. Crystal data and structure refinement for (*R*,*S*)-3za

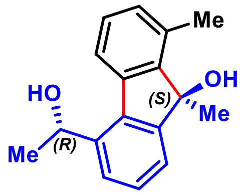 (*R*,*S*)-**3za**	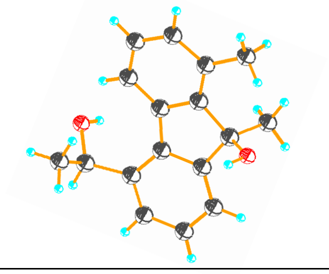
Identification code	CCDC 2323314
Empirical formula	$C_{17}H_{18}O_2$
Formula weight	254.31
Temperature	296.88(15) K
Wavelength	1.54184 Å
Crystal system	Triclinic
Space group	P1
Unit cell dimensions	a = 12.20290(10) Å α = 106.0410(10)°<br>b = 12.20700(10) Å β = 106.0660(10)°<br>c = 20.2310(2) Å γ = 90.6580(10)°
Volume	2770.52(5) $Å^3$
Z	8
Density (calculated)	1.219 Mg/m3
Absorption coefficient	0.620 mm^{-1}
F(000)	1088
Crystal size	0.05 x 0.04 x 0.03 mm^3
Theta range for data collection	3.785 to 71.398°
Index ranges	-14<=h<=14, -15<=k<=14, -24<=l<=24
Reflections collected	94592
Independent reflections	20130 [R(int) = 0.0244]
Completeness to theta = 67.684°	98.7 %
Absorption correction	Semi-empirical from equivalents
Refinement method	Full-matrix least-squares on F^2
Data / restraints / parameters	20130 / 3 / 1454
Goodness-of-fit on F^2	1.031
Final R indices [I>2sigma(I)]	R1 = 0.0324, wR2 = 0.0885
R indices (all data)	R1 = 0.0329, wR2 = 0.0890
Absolute structure parameter	-0.02(3)
Extinction coefficient	n/a
Largest diff. peak and hole	0.161 and -0.129 e.$Å^{-3}$

7. Computational details and proposed catalytic cycle

7.1 Computational methods

All density functional theory (DFT) calculations were carried out using the Gaussian 16 software package.[7] Geometries were optimized using the B3LYP-D3(BJ) (Becke−Johnson damping function) functional with the basis set of LANL2DZ for palladium, bromine and iodine, and 6-31G(d) for other atoms.[8] Vibrational frequencies were calculated for all the stationary points to confirm if each optimized structure is a local minimum on the respective potential energy surface or a transition state structure with only one imaginary frequency. Gibbs free energies were calculated under 398.15 K and 1 M for species in solution using the Shermo 2.3.4 package.[9] Grimme's entropy interpolation between harmonic and free-rotor approximations were applied for vibrational entropy calculations to correct the harmonic oscillator model for free energies of low-frequency vibrations.[10] Standard state correction was considered for all structures. Solvation energy corrections were calculated in DCE solvent with the SMD continuum solvation model based on the gas-phase optimized geometries.[11] The M06L functional with the basis set of SDD for palladium, bromine and iodine, 6-311+G(d,p) for other atoms was used for single-point energy calculations.[12,13] The 3D diagrams of molecules were generated using CYLview20.[14]

The conformational space of transition states and intermediates were explored by manually changing the orientation of substituents. All of the transition states were further sampled using the conformer-rotamer ensemble sampling tool (CREST) program that uses the semiempirical tight-binding based quantum chemistry methods GFN2-xTB to perform metadynamic sampling (MTD) of conformers.[15] iMTD-GC (iterative metadynamic sampling with genetic crossing approach) was used where an iterative root mean square deviation (RMSD) based metadynamic sampling was performed with an extra genetic z matrix crossing (GC) step at the end. For transition states sampling, the corresponding forming/breaking bonds were constrained at the transition state geometry obtained from DFT-optimized geometries with a force constant of 0.5 hartree/bohr^2. The conformers generated from the CREST/xTB sampling that within an energy threshold of 10 kcal/mol were sorted manually to retain representative structures. The conformers obtained from conformational sampling and clustering were further optimized at the DFT level.

7.2 DFT-computed free energy profile for the formations of chiral fluorenols

To shed light on this catalytic cycle, DFT calculations were carried out to deepen the understanding of the mechanism (Figure S3 and S4). The corresponding two pathways from **ANP*** to (*S*)- and (*R*)-**3aa**, and optimized structures and relative free energies of the transition states of the stereoselectivity-determining reductive elimination step were computed and discussed.

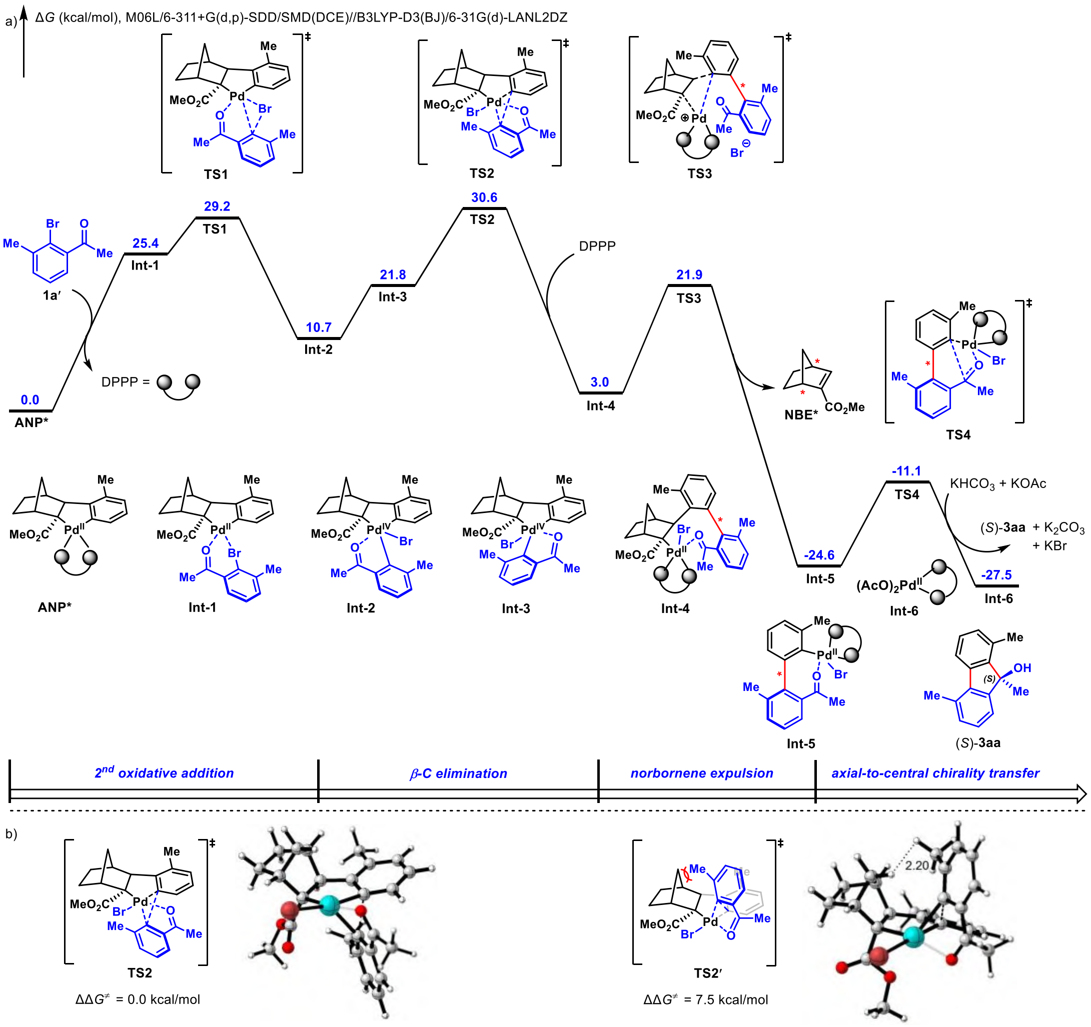

Figure S3. a) DFT-computed free energy profile for the formations of (*S*)-**3aa**. b) The optimized structures and relative free energies of the transition states of the stereoselectivity-determining reductive elimination step.

From chiral **ANP***, the subsequent oxidative addition of aryl bromide **1a'** and reductive elimination generate a series of C–C atropisomeric intermediates (Figure S3a). Based on the DFT-computed free energies changes of the possibility pathway, the generation of the Pd(IV) intermediate **Int-2** via the oxidative addition of aryl bromide **1a'** to the **ANP*** through the transition state **TS-1** necessitates an activation free energy

of 29.2 kcal/mol. The Pd(IV) intermediate **Int-2**, undergoes the irreversible and stereoselectivity-determining reductive elimination step via **TS2** to produce the C–C axially chiral Pd(II) intermediate **Int-4**. This axial chirality control is dictated by the chirality transfer from the chiral cyclometallated fragment. As revealed in Figure S3b, transition state **TS2'** is disfavored because of the steric repulsions between the bulky methyl substituent and the NBE* fragment. In contrast, such steric repulsions are not present in the favored transition state **TS2**, which leads to the 7.5 kcal/mol free energy difference between the two competing reductive elimination processes. Due to the steric hindrance around the arene, the resulting intermediate **Int-4** readily undergoes *β*-carbon elimination through **TS3** to produce axially chiral intermediate **Int-5** and regenerate NBE*. Finally, the intermediate **Int-5** undergoes intramolecular addition of Pd(II) species to the carbonyl group via **TS4**, and subsequent protonolysis to afford the chiral FOL product (*S*)-**3aa** and regenerate the Pd(II) complex **Int-6**, alongside the disappearance of the transient axial chirality.

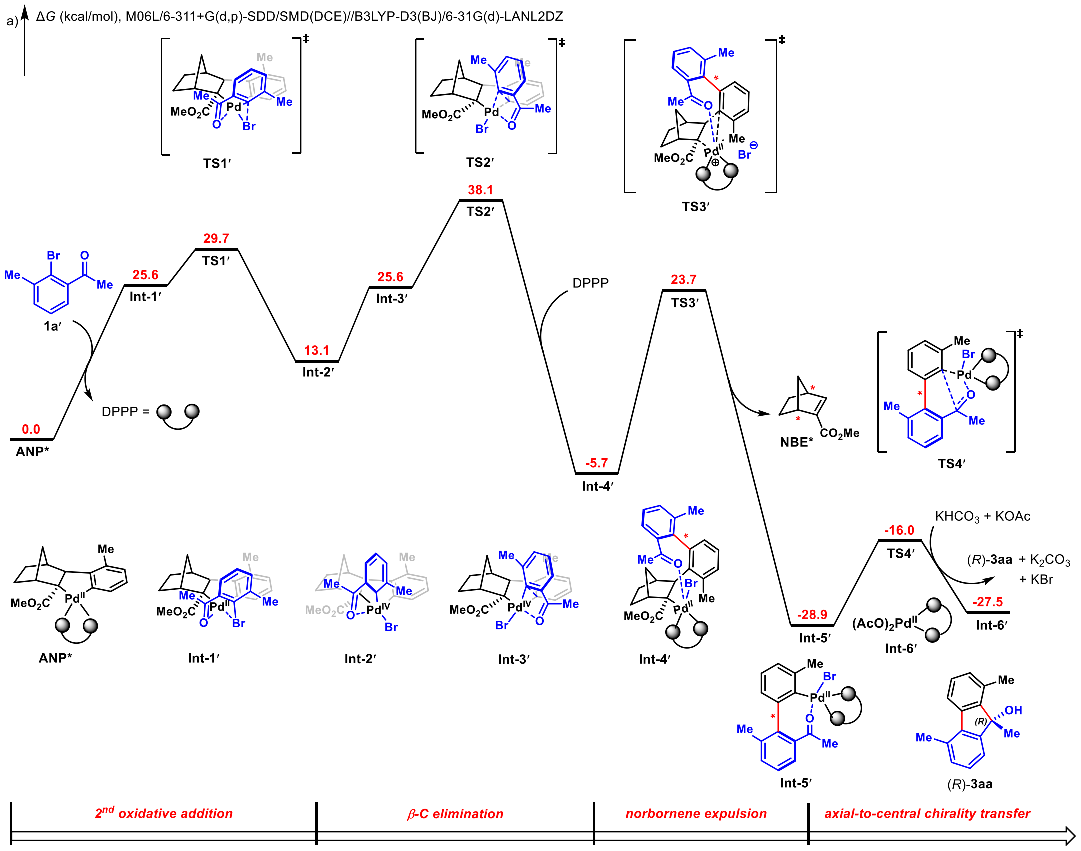

Figure S4. DFT-computed free energy profile for the formations of (*R*)-**3aa**. All energies were calculated at M06L/6-311+G(d,p)-SDD/SMD(DCE)//B3LYP-D3(BJ)/6-31G(d)-LANL2DZ level of theory and in kcal/mol.

7.3 Proposed catalytic cycle

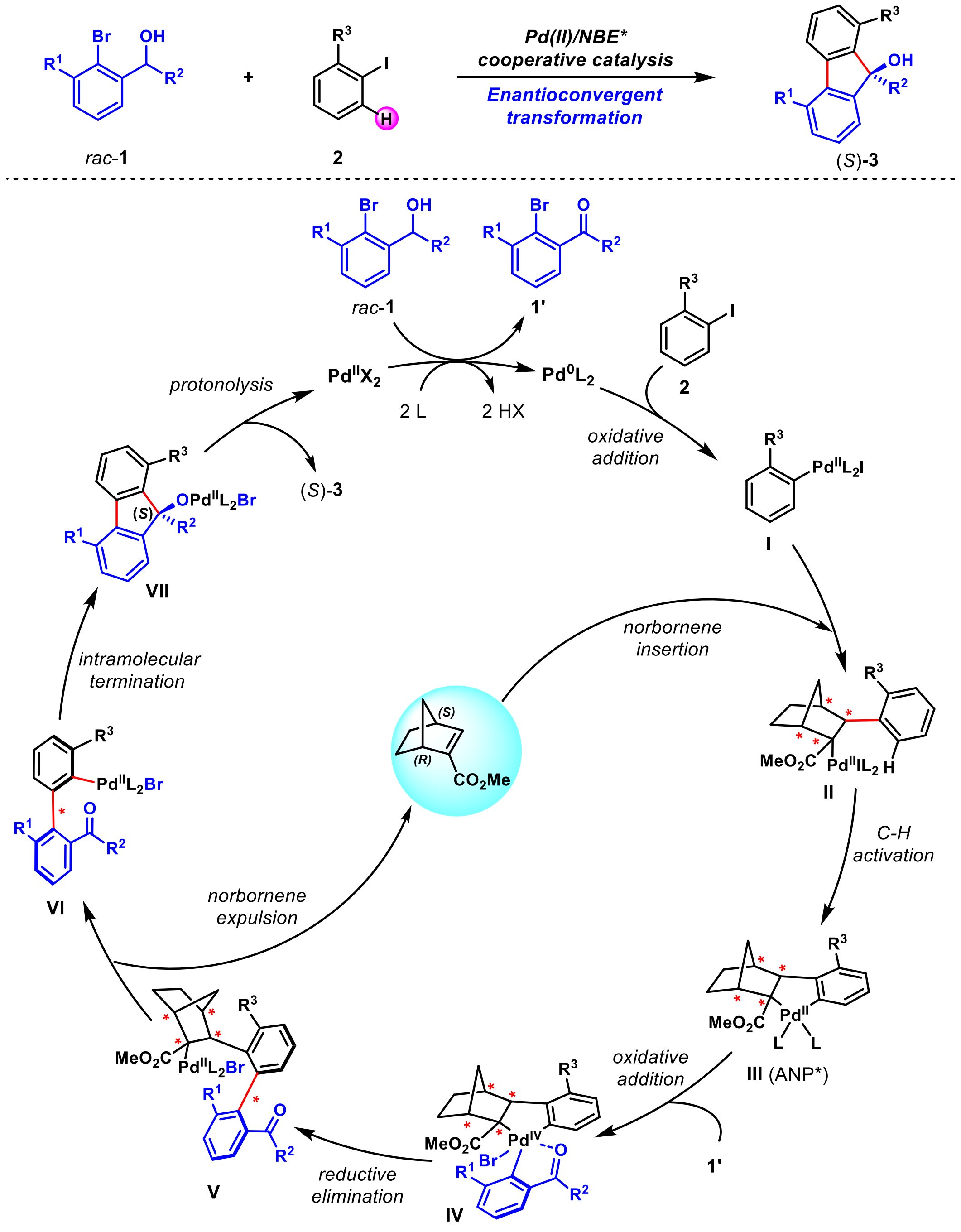

Figure S5. Proposed catalytic cycle.

7.4 Cartesian coordinates (Å) and energies of optimized structures

ANP*

B3LYP-D3(BJ) SCF energy = -2625.262633 a.u.

M06L SCF energy in solution = -2626.537892 a.u.

M06L free energy in solution = -2625.882041 a.u.

C	-1.36428500	-2.95101200	0.53653900
C	-0.37871700	-3.75140800	-0.33409700
C	0.65458100	-2.94194800	-1.13549600
P	-1.86838600	-1.28964700	-0.11887700
C	-2.66999700	-1.68000100	-1.71790400
C	-2.51242200	-0.76372200	-2.76956700
C	-3.06834200	-1.02575800	-4.02170900
C	-3.77610700	-2.20900300	-4.24067700
C	-3.92618900	-3.13312200	-3.20449900
C	-3.37463500	-2.87148600	-1.95036800
C	-3.19317300	-0.88407600	1.08229400
C	-4.49854700	-1.37882700	0.97750000
C	-5.44675000	-1.06589400	1.95211200
C	-5.09545500	-0.26442800	3.03976200
C	-3.79521400	0.23309100	3.14700000
C	-2.84893000	-0.06656100	2.16869800
P	1.44918200	-1.51705000	-0.22143800
C	3.04156600	-1.39042400	-1.11570900
C	4.23519100	-1.09401000	-0.44265400
C	5.41593500	-0.91351300	-1.16172700
C	5.42045600	-1.01664800	-2.55379700
C	4.23367800	-1.30350300	-3.23019200
C	3.05082400	-1.48890600	-2.51591600
C	1.88033600	-2.26958400	1.38984800
C	1.27448400	-1.76248700	2.54621800
C	1.51326000	-2.35460500	3.78633800
C	2.36850100	-3.45399700	3.88100500
C	2.98350500	-3.96033900	2.73344800
C	2.73966700	-3.37175300	1.49263700
H	-2.25829000	-3.54990200	0.74123800
H	-0.91627500	-2.72950900	1.50947600
H	-0.94444900	-4.35564000	-1.05231200
H	0.15052200	-4.45171500	0.32125800
H	0.17368800	-2.49608700	-2.01353200
H	1.43342700	-3.61849500	-1.50166900
H	-1.95371800	0.15105900	-2.59422900
H	-2.94394300	-0.30693000	-4.82641700
H	-4.20474700	-2.41453200	-5.21746300

H	-4.46937900	-4.05869300	-3.37341600
H	-3.49094000	-3.60166200	-1.15515900
H	-4.78432500	-1.98868000	0.12624900
H	-6.46113200	-1.44367300	1.85820400
H	-5.83640400	-0.01866500	3.79528700
H	-3.52258500	0.87416000	3.98035500
H	-1.85260000	0.36358800	2.21727600
H	4.22746500	-0.96616000	0.63149200
H	6.33396300	-0.67972200	-0.63011600
H	6.34195400	-0.86785600	-3.10952800
H	4.22594400	-1.37914700	-4.31390600
H	2.13171900	-1.69332000	-3.05691800
H	0.61870100	-0.90066300	2.46215800
H	1.03726700	-1.95432500	4.67708400
H	2.55843500	-3.91398900	4.84663900
H	3.65282600	-4.81303600	2.80537100
H	3.22722000	-3.76071600	0.60320300
Pd	-0.12537900	0.33144600	-0.17057600
C	-3.58970300	3.03392400	-0.40041500
C	-3.01415100	4.25850900	-0.06897400
C	-2.79418000	1.89158300	-0.48040300
C	-1.40881000	1.94541700	-0.25615400
C	-0.83273000	3.21051500	-0.00630400
C	-1.63513500	4.36431000	0.12906500
C	-1.03434600	5.70113400	0.50046100
H	-4.66096800	2.96205000	-0.57489700
H	-3.63686700	5.14497200	0.03288800
H	-3.29000100	0.95579700	-0.70917000
H	-1.81829100	6.44944900	0.65719400
H	-0.44562800	5.63319400	1.42409200
H	-0.35810900	6.08542500	-0.27209200
C	3.46336600	2.94465500	-0.79489100
C	2.32507300	1.93877900	-1.10785200
C	1.33868300	1.88552100	0.07829900
C	0.65964800	3.29426100	0.05975000
C	2.74681100	4.32923700	-0.87904900
C	1.29093000	3.94261700	-1.21490600
C	1.49780900	2.72828100	-2.13700700

C	1.92748600	1.48868900	1.37131000
O	2.93038100	0.80251300	1.54130700
O	1.22386200	1.96177500	2.44955600
C	1.79799900	1.67329200	3.72533200
H	4.25493000	2.86098300	-1.54894100
H	3.92156900	2.74929600	0.17745000
H	2.69697200	0.97367000	-1.43384300
H	0.96398900	3.87723100	0.93764600
H	2.81150900	4.89885800	0.05518500
H	3.17711400	4.95178100	-1.67219600
H	0.69307700	4.75810500	-1.62792100
H	0.56277700	2.22861500	-2.40749300
H	2.06049700	2.97387700	-3.04594000
H	1.11284800	2.10206500	4.45878300
H	1.90225900	0.59644700	3.88001300
H	2.78773800	2.13133600	3.81880900

Int-1

B3LYP-D3(BJ) SCF energy = -1334.649280 a.u.

M06L SCF energy in solution = -1336.144570 a.u.

M06L free energy in solution = -1335.766222 a.u.

C	-3.13090500	1.22132700	1.24368800
C	-3.49475400	0.56487500	2.42906800
C	-3.53783400	-0.82340700	2.51360400
C	-3.18017500	-1.59319700	1.41258500
C	-2.78598900	-0.98973200	0.20946900
C	-2.80542300	0.41386800	0.15066800
Br	-2.46802700	1.35544000	-1.57083500
C	-2.24617700	-1.87478400	-0.86808600
C	-2.78068900	-3.27685200	-1.00067700
O	-1.33684300	-1.50657700	-1.60932500
C	-3.06782700	2.72503300	1.19938300
H	-3.74904400	1.16888100	3.29531000
H	-3.82932800	-1.30475700	3.44179100
H	-3.14667600	-2.67242600	1.49529500
H	-3.84933900	-3.34771400	-0.78303200
H	-2.56765600	-3.64024600	-2.00765600

H	-2.22864600	-3.90511600	-0.29045200
H	-3.33321300	3.14609200	2.17257300
H	-2.05484900	3.05768200	0.94482800
H	-3.74761800	3.13974000	0.44769900
Pd	0.04802200	0.17467500	-0.88175800
C	1.83370700	3.89190900	0.17021600
C	3.05132400	3.49320100	0.72235500
C	0.93420300	2.93399700	-0.30478300
C	1.26705300	1.57691900	-0.23339200
C	2.50209000	1.17694800	0.31149300
C	3.40219100	2.14008900	0.80438600
C	4.71444500	1.73329000	1.43215000
H	1.58348500	4.94896600	0.11499500
H	3.74200900	4.24245400	1.10304000
H	-0.01554000	3.24779500	-0.73205400
H	5.23694200	2.60068200	1.84816400
H	4.56297400	1.00995100	2.24349800
H	5.38578300	1.25568800	0.70839500
C	2.86505700	-3.11101800	-0.45846700
C	1.90906800	-2.07134700	-1.10685600
C	1.45602200	-1.08378000	-0.02007000
C	2.75229600	-0.30071100	0.36263100
C	4.16573500	-2.28586700	-0.19596500
C	3.77736700	-0.86818400	-0.67014900
C	2.90202700	-1.19484800	-1.89586000
C	0.65201900	-1.66020700	1.08788500
O	-0.14650300	-2.58802500	0.99036900
O	0.88710400	-1.04732500	2.27801700
C	0.10559800	-1.50801300	3.38116500
H	3.05181200	-3.93699800	-1.15392900
H	2.43872600	-3.54521000	0.45061600
H	1.08578900	-2.51935400	-1.66428000
H	3.08243300	-0.58009800	1.37074600
H	4.46976000	-2.29829000	0.85659600
H	5.00397600	-2.67504000	-0.78476200
H	4.62745000	-0.20650500	-0.85132000
H	2.43060400	-0.31551900	-2.34367200
H	3.44802000	-1.75077600	-2.66720200

H	0.50988100	-1.00117200	4.25869400
H	-0.94591200	-1.24287100	3.24355600
H	0.18746000	-2.59293500	3.49155600

Int-1'

B3LYP-D3(BJ) SCF energy = -1334.644004 a.u.

M06L SCF energy in solution = -1336.143117 a.u.

M06L free energy in solution = -1335.765838 a.u.

C	2.55014800	1.49554900	1.42338500
C	2.64134400	1.04799700	2.75094600
C	2.80581900	-0.29743600	3.06519100
C	2.85889600	-1.24566600	2.04703300
C	2.74341700	-0.85303500	0.70760500
C	2.61667700	0.51562900	0.43109900
Br	2.66400600	1.12210800	-1.46502000
C	2.57012600	-1.90860700	-0.34111700
C	3.56246400	-3.03749600	-0.41057700
O	1.62263800	-1.85626800	-1.12054900
C	2.33121200	2.95190700	1.11809300
H	2.57846500	1.78464200	3.54655200
H	2.87494700	-0.60981600	4.10211700
H	2.93706900	-2.30020500	2.28977400
H	4.53529700	-2.63817500	-0.72450100
H	3.71234300	-3.50053400	0.57017700
H	3.21951500	-3.78464900	-1.12781000
H	2.30473500	3.53631300	2.04141600
H	3.12082900	3.35556500	0.47565900
H	1.37725600	3.08896000	0.59416400
Pd	0.08404500	-0.10901900	-0.89438500
C	-1.63445800	3.79173600	-0.90617000
C	-2.80086600	3.61049100	-0.16353500
C	-0.77850600	2.71177600	-1.13988600
C	-1.08750500	1.45260500	-0.61091500
C	-2.25815200	1.27737700	0.15149200
C	-3.13322600	2.35850600	0.36822500
C	-4.42226600	2.18249000	1.13622400
H	-1.39865200	4.77112900	-1.31616900

H	-3.47320400	4.44992500	-0.00046000
H	0.11519600	2.85393300	-1.74173600
H	-5.04176700	3.08284800	1.07331500
H	-5.00899000	1.34170800	0.74590200
H	-4.24583500	1.97496100	2.19865400
C	-1.92970000	-2.67634700	1.98586800
C	-0.84105300	-1.78252400	1.33024300
C	-1.44680600	-1.10408600	0.09014900
C	-2.49965300	-0.10634700	0.67392300
C	-2.91139400	-1.63770600	2.61427600
C	-2.28658800	-0.28487600	2.21089700
C	-0.78194700	-0.60104800	2.31600700
C	-1.88034700	-2.03095900	-0.98821300
O	-1.38379400	-3.12389900	-1.21482500
O	-2.90593000	-1.53194200	-1.73091400
C	-3.28294400	-2.33089500	-2.85460500
H	-1.47858400	-3.31244600	2.75626300
H	-2.40319700	-3.33820500	1.25678400
H	0.08763800	-2.31742600	1.13188800
H	-3.51549300	-0.43018400	0.41478700
H	-3.93549100	-1.74351300	2.23992800
H	-2.94865300	-1.73852900	3.70493600
H	-2.63798600	0.56459100	2.80058900
H	-0.14019900	0.21294500	1.97126100
H	-0.48023000	-0.89820600	3.32757500
H	-4.11950600	-1.80803500	-3.32012800
H	-2.44994000	-2.41995900	-3.55882300
H	-3.58329600	-3.33388200	-2.53822700

Int-2

B3LYP-D3(BJ) SCF energy = -1334.671785 a.u.

M06L SCF energy in solution = -1336.171366 a.u.

M06L free energy in solution = -1335.789537 a.u.

C	1.38811600	-0.49013300	2.22315600
C	2.48526000	-0.22071200	3.06562800
C	3.66010500	0.37695400	2.61867500
C	3.76288400	0.74945900	1.28733200

C	2.70217800	0.49363200	0.40566200
C	1.53088400	-0.15025600	0.87480700
Br	1.47489200	-2.61767900	-1.52688800
C	2.77057200	0.92576500	-0.99554200
C	3.93460300	1.72027900	-1.51788400
O	1.84233100	0.64365600	-1.76785100
C	0.15798300	-1.07824000	2.86069700
H	2.39153900	-0.48612900	4.11527300
H	4.47665100	0.56242600	3.30928700
H	4.65852400	1.24026700	0.92294300
H	4.00917100	2.66949000	-0.97597500
H	4.87422800	1.17420700	-1.38104400
H	3.78057800	1.91751100	-2.57934100
H	0.27078500	-1.08921000	3.94885000
H	-0.72791900	-0.49395800	2.60746900
H	-0.01990700	-2.10140200	2.52405200
Pd	0.27564200	-0.47896300	-0.67827100
C	-2.47415600	-3.25593500	1.07351300
C	-3.54432800	-2.41883200	1.39433400
C	-1.32196400	-2.73406900	0.48713400
C	-1.27490400	-1.36700500	0.20943800
C	-2.35772000	-0.52054000	0.48617400
C	-3.51057400	-1.05261500	1.10323400
C	-4.67658400	-0.16698500	1.47426800
H	-2.53097300	-4.31874300	1.29280800
H	-4.42497500	-2.83251700	1.87957700
H	-0.47927200	-3.37174100	0.25193700
H	-5.43285100	-0.73260400	2.02662900
H	-4.35873400	0.67270800	2.10483400
H	-5.16349300	0.26701900	0.59323600
C	-1.77307900	3.16874700	-1.78122700
C	-0.99086800	1.82620700	-1.82139600
C	-0.83125500	1.31749900	-0.37957200
C	-2.26821100	0.91649000	0.06324900
C	-3.21737100	2.73107700	-1.39138700
C	-3.08641500	1.20000000	-1.24268200
C	-2.07473200	0.86411300	-2.35195600
C	-0.04693600	2.20723000	0.51638700

O	0.84868300	2.95222600	0.15396400
O	-0.41716000	2.09361200	1.81595700
C	0.38322800	2.83689100	2.74487900
H	-1.75062500	3.63072700	-2.77409400
H	-1.32710900	3.88011900	-1.08252500
H	-0.05533700	1.88698500	-2.37471200
H	-2.61881800	1.57063100	0.86817600
H	-3.56785700	3.20106800	-0.46623500
H	-3.93506800	2.98203500	-2.17942500
H	-4.03308200	0.65832500	-1.28104200
H	-1.77770200	-0.19309100	-2.36291200
H	-2.42727600	1.12837800	-3.35423600
H	-0.04850400	2.63676800	3.72585700
H	1.42078700	2.49676000	2.70775800
H	0.34535800	3.90498700	2.51632400

Int-2'

B3LYP-D3(BJ) SCF energy = -1334.666546 a.u.

M06L SCF energy in solution = -1336.167195 a.u.

M06L free energy in solution = -1335.785671 a.u.

C	-1.25984600	-0.51682000	2.47788100
C	-2.15935000	-0.08690300	3.46929700
C	-3.20504800	0.79843300	3.21570100
C	-3.38367100	1.27438100	1.92829100
C	-2.50515500	0.87738600	0.90474800
C	-1.42483800	-0.00386400	1.18415700
Br	-1.98811200	-2.35049400	-1.04063300
C	-2.71372600	1.34143100	-0.46818800
C	-3.86843400	2.23593100	-0.83644500
O	-1.93380400	0.98360400	-1.36768700
C	-0.19376800	-1.49896200	2.88565200
H	-2.02579700	-0.47220300	4.47672000
H	-3.87431600	1.09914200	4.01546700
H	-4.20421700	1.94734100	1.70410100
H	-4.81936600	1.73103000	-0.63260700
H	-3.85043900	3.16201000	-0.25168800
H	-3.80601100	2.47406800	-1.89893200

H	-0.26306600	-1.70928200	3.95697800
H	-0.30591500	-2.43941200	2.34199400
H	0.80987500	-1.12548500	2.66946000
Pd	-0.38271600	-0.35728600	-0.49925000
C	2.39848700	-3.40932500	0.59407700
C	3.57971000	-2.68283200	0.74315600
C	1.20326000	-2.75706200	0.29385000
C	1.21203600	-1.36893200	0.16447800
C	2.39159000	-0.62279100	0.30288500
C	3.60174000	-1.29449700	0.58576100
C	4.91076000	-0.54776600	0.69109300
H	2.40718600	-4.49074000	0.70113800
H	4.50821300	-3.20254500	0.96639700
H	0.28594300	-3.31516000	0.15589900
H	5.75218100	-1.24619600	0.72424400
H	5.06540100	0.12242800	-0.16345300
H	4.96231400	0.07424000	1.59242500
C	1.56343500	3.68518100	0.08750200
C	0.59013800	2.51148400	0.40471800
C	0.94064600	1.31489800	-0.47515600
C	2.34272100	0.85464600	0.03838200
C	2.91960900	3.19970300	0.69106900
C	2.56540100	1.80690300	1.25396900
C	1.13476200	2.04053300	1.76761200
C	0.83301400	1.46925300	-1.96253100
O	0.69807100	2.45920900	-2.63892300
O	0.81850800	0.19270300	-2.49985900
C	0.43018100	0.07127700	-3.88318500
H	1.20840400	4.59039900	0.59208000
H	1.61345500	3.89761000	-0.98013500
H	-0.46143300	2.79856000	0.36557700
H	3.10081300	1.08583000	-0.72393900
H	3.71991600	3.15111200	-0.05535400
H	3.25908300	3.86528900	1.49176700
H	3.27713900	1.41800300	1.98395300
H	0.65083000	1.14040600	2.14231100
H	1.08257600	2.82141800	2.53418500
H	0.35659400	-0.99941600	-4.06589300

H	-0.53414000	0.55911700	-4.03649400
H	1.18947800	0.53061800	-4.51946900

Int-3

B3LYP-D3(BJ) SCF energy = -1334.647396 a.u.

M06L SCF energy in solution = -1336.151780 a.u.

M06L free energy in solution = -1335.771838 a.u.

C	-1.07933800	1.31766000	1.99394100
C	-2.08636800	1.64806800	2.92144900
C	-3.44456300	1.51767500	2.64579500
C	-3.84143400	1.11088100	1.38040600
C	-2.87374800	0.77287400	0.42249000
C	-1.50124700	0.78098600	0.77613500
Br	-1.75173900	-2.49987100	-0.81276700
C	-3.23821300	0.53715000	-0.98280100
C	-4.67267500	0.42762900	-1.41762600
O	-2.33777000	0.45986300	-1.83536300
C	0.33678800	1.68637200	2.35623100
H	-1.77636900	2.03620800	3.88819200
H	-4.18197400	1.77159900	3.40052900
H	-4.89407400	1.06147600	1.12383400
H	-5.11245600	-0.46787300	-0.96266200
H	-5.26191000	1.29428800	-1.10107300
H	-4.70817100	0.33182900	-2.50332600
H	1.06811500	0.91613400	2.13345000
H	0.63830100	2.57984400	1.79939000
H	0.40558400	1.91259200	3.42440400
Pd	-0.49726800	-0.18208300	-0.68752600
C	0.64338300	3.89736900	-1.42456300
C	1.98892700	3.87612400	-1.05098100
C	-0.12777500	2.73883600	-1.32813900
C	0.47894600	1.56882100	-0.87632200
C	1.82874200	1.52787700	-0.51635700
C	2.60338400	2.70340300	-0.59898000
C	4.05266100	2.71772800	-0.17509800
H	0.19323200	4.82124600	-1.77798300
H	2.57567600	4.78979100	-1.10550900

H	-1.17737500	2.74750800	-1.60051300
H	4.46126200	3.73178200	-0.21681700
H	4.17377500	2.34888000	0.85129000
H	4.67608900	2.08084400	-0.81333900
C	3.00406800	-2.67270300	-0.24862300
C	1.67993900	-2.03258400	-0.75571100
C	1.27492400	-0.85994000	0.18218600
C	2.39677200	0.20943000	-0.07863700
C	4.07901600	-1.59103600	-0.55777000
C	3.24706900	-0.46493800	-1.20575400
C	2.18948300	-1.25872000	-1.98945000
C	1.05130500	-1.30133600	1.61013000
O	0.02461100	-1.72273200	2.08655700
O	2.20092400	-1.20136900	2.34094800
C	2.08481500	-1.63888400	3.70292700
H	3.17890300	-3.58696500	-0.82537000
H	2.96855300	-2.95132800	0.80542000
H	0.88323400	-2.75547900	-0.90583600
H	3.01254400	0.33725700	0.81569500
H	4.59773700	-1.24633600	0.34229000
H	4.83500200	-1.96665800	-1.25538400
H	3.82811200	0.25562500	-1.78422100
H	1.43435800	-0.62356100	-2.46997300
H	2.61630300	-1.91856500	-2.75174900
H	1.77785900	-2.68677800	3.74477600
H	3.07618000	-1.50738800	4.13730700
H	1.34542300	-1.03319300	4.23377900

Int-3'

B3LYP-D3(BJ) SCF energy = -1334.645461 a.u.

M06L SCF energy in solution = -1335.331929 a.u.

M06L free energy in solution = -1335.765777 a.u.

C	0.87507300	2.29151100	1.47130300
C	1.84544700	2.97514000	2.23724900
C	3.21028300	2.76629600	2.09569200
C	3.65371700	1.91432500	1.09074200

C	2.72241500	1.21813800	0.31236300
C	1.32473900	1.30038900	0.59104400
Br	1.98355300	-2.19054700	0.47684400
C	3.14138100	0.54143700	-0.92375700
C	4.58942700	0.29050900	-1.23965400
O	2.27222900	0.21823400	-1.75497300
C	-0.53804800	2.81285200	1.61747000
H	1.49155600	3.71328600	2.95366000
H	3.91784700	3.30363100	2.71922100
H	4.71464000	1.80438900	0.89382000
H	5.00701000	-0.37806800	-0.47848500
H	4.66335600	-0.18433100	-2.21865500
H	5.17033000	1.21866100	-1.23656800
H	-0.56162300	3.84733400	1.25299400
H	-1.26213300	2.25547900	1.03698300
H	-0.85570700	2.82831900	2.66573300
Pd	0.45048200	-0.20256500	-0.48616500
C	-1.52709300	3.05319100	-2.45811700
C	-2.84722100	2.87020700	-2.04501800
C	-0.52634100	2.18953400	-2.00737200
C	-0.87374700	1.15689600	-1.14367200
C	-2.19206000	0.97139900	-0.70317600
C	-3.20350700	1.83125500	-1.17744100
C	-4.64511900	1.63848300	-0.77212300
H	-1.27830500	3.86660400	-3.13471300
H	-3.62182600	3.54188400	-2.40657900
H	0.50347600	2.31613100	-2.32589800
H	-5.29456100	2.35385600	-1.28520400
H	-4.99934900	0.62899800	-1.01761500
H	-4.79058800	1.76871900	0.30643500
C	-2.56854900	-2.24125000	2.21967200
C	-1.24132900	-1.49489500	1.86250300
C	-1.26082100	-1.12893800	0.38263100
C	-2.47878700	-0.17627000	0.22700000
C	-3.64328700	-1.10702900	2.21530200
C	-2.83306300	0.11442300	1.72895500
C	-1.50703500	-0.12196300	2.48724500
C	-1.21833500	-2.27518500	-0.59875000

O	-1.63278900	-3.39786200	-0.48484800
O	-0.57800700	-1.82023800	-1.73206300
C	-0.03043900	-2.81270000	-2.63126000
H	-2.45697800	-2.67933300	3.21667900
H	-2.78848100	-3.05092700	1.52516000
H	-0.33640000	-2.01975200	2.16165200
H	-3.31213100	-0.75216100	-0.20267300
H	-4.49265800	-1.33013700	1.56083800
H	-4.04022900	-0.93094900	3.22078700
H	-3.31746500	1.08016400	1.88677000
H	-0.70821200	0.57963600	2.27821200
H	-1.66064000	-0.17351200	3.57076000
H	-0.82260600	-3.49642500	-2.93714100
H	0.35696000	-2.24836300	-3.47770300
H	0.77052000	-3.34358200	-2.11233600

Int-4

B3LYP-D3(BJ) SCF energy = -3062.124275 a.u.

M06L SCF energy in solution = -3063.612744 a.u.

M06L free energy in solution = -3062.812414 a.u.

C	4.71098700	0.56604300	1.51797600
C	4.89168800	-0.26100800	2.62810700
C	3.83820600	-0.54522000	3.49705500
C	2.59319500	0.02010900	3.26513200
C	2.39398300	0.90172000	2.18801000
C	3.45003200	1.16219300	1.29078700
Br	-0.44964600	2.29010300	-1.67014500
C	1.03949300	1.55451000	2.14885600
C	0.87452700	2.99180900	1.73251200
O	0.06671400	0.91466100	2.55200900
C	5.86167800	0.81755900	0.57436700
H	5.87255000	-0.69441900	2.80835200
H	3.99326400	-1.19796800	4.35161800
H	1.76304400	-0.17024200	3.93566500
H	-0.05879300	3.36884500	2.14911900
H	1.71725500	3.60412000	2.05697300
H	0.81452500	3.06130000	0.64062000

H	6.76367700	0.29901400	0.91356100
H	5.62820000	0.46496300	-0.43690300
H	6.08693000	1.88538500	0.48833100
Pd	-0.47249700	0.02897500	-0.32046000
C	3.68673800	4.46660900	-0.41706700
C	3.21160100	4.17541400	-1.68720700
C	3.74037800	3.43717700	0.50806200
C	3.32212200	2.14420800	0.16745300
C	2.88469000	1.81397200	-1.14187200
C	2.82560900	2.88486300	-2.07827400
C	2.39109000	2.80278100	-3.52906600
H	4.00030700	5.47309000	-0.15488200
H	3.14265400	4.97176500	-2.42380400
H	4.09715400	3.61886700	1.51862500
H	1.63031900	2.05456600	-3.71305900
H	1.96881800	3.76724000	-3.82680800
H	3.24234600	2.60659800	-4.19415400
C	2.41843600	-2.45318900	-2.68240800
C	1.27176500	-1.46847900	-2.34883400
C	1.56994700	-0.67136300	-1.02326500
C	2.76179800	0.30920500	-1.48094400
C	3.58461800	-1.48990900	-3.04994400
C	2.92869200	-0.08512500	-2.97666100
C	1.49024200	-0.39993200	-3.42123100
C	2.02937700	-1.67755900	0.00148800
O	1.31673300	-2.48852200	0.57798700
O	3.36985200	-1.76405900	0.17705900
C	3.81535600	-2.86707000	0.97412400
H	2.12055400	-3.06999700	-3.53806400
H	2.65845500	-3.14219000	-1.86879800
H	0.29789600	-1.94269500	-2.35615900
H	3.63538900	-0.09116500	-0.97118600
H	4.43440100	-1.58080300	-2.36622300
H	3.95392700	-1.68168300	-4.06376800
H	3.48257100	0.64590300	-3.56057000
H	0.78896800	0.42682900	-3.31580500
H	1.43244100	-0.80808100	-4.43805500
H	3.33115200	-2.85695400	1.95068700

H	3.59242300	-3.81052800	0.46652600
H	4.89186500	-2.73174800	1.07766100
C	-3.14768100	-2.13705300	0.15261200
C	-3.86502500	-1.48515200	1.34373500
C	-3.26639700	-0.16647500	1.85910600
P	-1.27597800	-2.15376100	0.23760300
C	-1.07404000	-2.91987500	1.88910600
C	-1.62385200	-4.17904500	2.16478000
C	-1.52830700	-4.72165100	3.44685800
C	-0.89261000	-4.00569500	4.46316500
C	-0.35135000	-2.74781700	4.19109000
C	-0.43535500	-2.20604900	2.90859000
C	-0.99563300	-3.48946500	-0.99305300
C	-0.12448800	-4.56029600	-0.75859700
C	0.07653700	-5.53091300	-1.73976900
C	-0.57854000	-5.44067600	-2.96904100
C	-1.44570800	-4.37450200	-3.21181800
C	-1.65542400	-3.40768600	-2.22940000
P	-2.56459800	0.91549800	0.52649000
C	-2.62263000	2.56785900	1.30754100
C	-2.78127100	3.71172300	0.51128700
C	-2.81309500	4.97565200	1.09767700
C	-2.69487000	5.11345300	2.48203100
C	-2.54301900	3.97850500	3.27998600
C	-2.50149900	2.71186300	2.69733200
C	-3.91341100	1.00123800	-0.71281400
C	-3.65471000	0.66938100	-2.04884800
C	-4.68871100	0.67538400	-2.98611700
C	-5.98543300	1.01425600	-2.59646600
C	-6.24803600	1.35533700	-1.26727900
C	-5.21597400	1.35161100	-0.32988000
H	-3.48911600	-3.17221300	0.05299800
H	-3.42236500	-1.62363400	-0.77141700
H	-3.88809400	-2.19296900	2.17912000
H	-4.90410400	-1.30965300	1.04322500
H	-2.42874200	-0.36427200	2.53447700
H	-4.02136900	0.38400900	2.42904900
H	-2.11527400	-4.74420000	1.37809300

H	-1.95112000	-5.70159700	3.65069900
H	-0.82093700	-4.42743100	5.46200600
H	0.14374000	-2.18551400	4.97853100
H	-0.01778600	-1.22858000	2.69669100
H	0.40883300	-4.61845800	0.18126100
H	0.75482100	-6.35688700	-1.54425400
H	-0.41185700	-6.19378200	-3.73391900
H	-1.95607100	-4.29034100	-4.16704800
H	-2.31692500	-2.57302600	-2.44150200
H	-2.86697600	3.61029800	-0.56345000
H	-2.92940500	5.85404700	0.46945000
H	-2.72070600	6.10001300	2.93637200
H	-2.44654200	4.07656700	4.35769900
H	-2.34109300	1.84418500	3.32440500
H	-2.63819000	0.43214800	-2.34593900
H	-4.47776100	0.42410500	-4.02172900
H	-6.78956400	1.01978100	-3.32702200
H	-7.25484300	1.62700300	-0.96254100
H	-5.42041500	1.63184600	0.69978500

Int-4'

B3LYP-D3(BJ) SCF energy = -3062.130286 a.u.

M06L SCF energy in solution = -3063.626247 a.u.

M06L free energy in solution = -3062.826269 a.u.

C	-5.20505300	-1.12719000	-0.28793600
C	-6.19934300	-0.30716900	-0.82825600
C	-5.94012000	0.53965200	-1.90566300
C	-4.68396600	0.51698600	-2.49238800
C	-3.65488100	-0.27475700	-1.95196200
C	-3.88406300	-1.04714900	-0.79173100
Br	1.47663100	-2.02490600	1.66250100
C	-2.34543700	-0.32913700	-2.65816700
C	-2.32353200	-0.00205800	-4.14729700
O	-1.30841400	-0.64636700	-2.09269200
C	-5.57529400	-2.13833900	0.77306100
H	-7.20128600	-0.35256900	-0.40946300

H	-6.72463800	1.17383300	-2.30758300
H	-4.49465000	1.12235500	-3.37239400
H	-1.35440600	-0.29230300	-4.55544400
H	-3.12281000	-0.52519500	-4.68154800
H	-2.46772600	1.07180700	-4.31990900
H	-4.97096100	-2.04145600	1.67830800
H	-6.62981100	-2.04124900	1.04771700
H	-5.40637100	-3.15505500	0.39980900
Pd	0.52928700	-0.00060200	0.30697100
C	-2.15226500	-4.30531500	-0.42912300
C	-1.68584900	-4.26027300	0.87802200
C	-2.77777000	-3.18073300	-0.94384700
C	-2.87820100	-1.99168700	-0.20009100
C	-2.28323900	-1.88937800	1.08644800
C	-1.77120900	-3.09516400	1.64552900
C	-1.40289900	-3.22458900	3.10609400
H	-2.08584800	-5.21936300	-1.01289000
H	-1.25960700	-5.15062900	1.33070000
H	-3.24131700	-3.21587700	-1.92620000
H	-2.28350600	-3.05592600	3.74193000
H	-1.03990600	-4.23618500	3.30881300
H	-0.63357000	-2.52054800	3.40978900
C	-2.27205200	2.13139600	3.14843200
C	-1.62145800	1.76034500	1.79078800
C	-0.95306400	0.35734100	1.87393500
C	-2.21433700	-0.62987300	1.97299800
C	-3.47120700	1.14675100	3.23410500
C	-3.39181700	0.38344400	1.89044800
C	-2.85826300	1.46765600	0.94236900
C	-0.02639100	0.40026900	3.05438000
O	0.87312700	1.22171000	3.18280200
O	-0.31529200	-0.46333900	4.05381900
C	0.63264000	-0.49795200	5.12832100
H	-2.61300300	3.17310200	3.11075600
H	-1.58092500	2.05177300	3.99097200
H	-0.94712900	2.53736400	1.44802100
H	-2.20125200	-1.01687200	2.99027300
H	-3.40266000	0.47051300	4.09282800

H	-4.42405800	1.68287700	3.31234500
H	-4.34399000	-0.05699100	1.61883300
H	-2.60508500	1.07962300	-0.04129400
H	-3.52970000	2.32554000	0.83274100
H	0.69091500	0.47458800	5.62362600
H	0.26304300	-1.25987600	5.81606600
H	1.61709300	-0.77202000	4.74190000
C	2.68140300	0.38568700	-2.67680300
C	1.33570100	0.62964300	-3.36613200
C	0.55441400	1.82140500	-2.80354900
P	2.40485500	-0.59502700	-1.11711500
C	2.21226000	-2.26554300	-1.83845600
C	3.26848600	-2.86842000	-2.53855800
C	3.08940200	-4.10565900	-3.15406400
C	1.84849000	-4.74390500	-3.08475400
C	0.79529200	-4.14565600	-2.39306900
C	0.97327800	-2.91358600	-1.76264500
C	4.06212700	-0.55834900	-0.34512000
C	4.61949200	-1.70942300	0.23182500
C	5.86276000	-1.64850000	0.85906600
C	6.55851700	-0.44027700	0.93137500
C	6.00519900	0.70993800	0.36820200
C	4.76612300	0.65236700	-0.26799600
P	0.41080600	1.99103600	-0.94922600
C	-0.86139700	3.32951100	-0.92128700
C	-0.65524200	4.51876100	-0.20764200
C	-1.64110200	5.50516000	-0.17532100
C	-2.84555500	5.32130400	-0.85579000
C	-3.06060000	4.14064500	-1.56828300
C	-2.07738800	3.15484800	-1.59708800
C	1.92382600	2.90647500	-0.43232800
C	2.34351200	2.75891200	0.89820700
C	3.47404700	3.43800200	1.35705600
C	4.20431700	4.25583800	0.49559600
C	3.78814900	4.41199900	-0.82951400
C	2.64797300	3.75178300	-1.28732300
H	3.35355700	-0.19383100	-3.31646700
H	3.18192000	1.32865000	-2.44560200

H	0.71463900	-0.26644700	-3.28566800
H	1.49686600	0.81169100	-4.43636500
H	-0.47098200	1.77971900	-3.17102600
H	0.96638500	2.76429000	-3.17719900
H	4.23652400	-2.37764900	-2.58762500
H	3.91550700	-4.57052800	-3.68523100
H	1.70667500	-5.70792800	-3.56605400
H	-0.16931900	-4.63701100	-2.32772900
H	0.15698300	-2.46168800	-1.21281200
H	4.07522700	-2.64477900	0.19670900
H	6.28299100	-2.54752900	1.30094400
H	7.52452600	-0.39557300	1.42680900
H	6.53168600	1.65859500	0.42420800
H	4.35219500	1.56092900	-0.68699700
H	0.27404200	4.67206300	0.32962300
H	-1.46530900	6.41906300	0.38489800
H	-3.61257900	6.08982700	-0.82693900
H	-3.99989700	3.97533400	-2.08849600
H	-2.28025500	2.23156200	-2.12469000
H	1.79304900	2.11684200	1.58121700
H	3.78694200	3.30562500	2.38821800
H	5.09289600	4.77088000	0.85016400
H	4.34802200	5.05115400	-1.50662600
H	2.33484400	3.90249200	-2.31489600

Int-5

B3LYP-D3(BJ) SCF energy = -2561.450897 a.u.

M06L SCF energy in solution = -2562.923916 a.u.

M06L free energy in solution = -2562.312573 a.u.

C	0.94415200	3.75936000	-0.37967200
C	0.02000500	4.67686100	0.13235500
C	-1.32789600	4.62014900	-0.20843800
C	-1.76901500	3.63383300	-1.08151500
C	-0.87079900	2.68552200	-1.58969300
C	0.50530500	2.74439300	-1.25785600
C	-1.42837700	1.63897600	-2.49206000

C	-2.30845000	2.07836400	-3.63441100
O	-1.21303300	0.43105900	-2.34669300
C	2.38571900	3.85067500	0.06056400
H	0.37018400	5.43977500	0.82284000
H	-2.03433500	5.33104900	0.20898700
H	-2.82151200	3.56910000	-1.32575100
H	-2.62446900	1.20846500	-4.21239500
H	-3.18943900	2.60791300	-3.25597000
H	-1.76123500	2.78009800	-4.27397400
H	2.45833100	4.38465700	1.01242100
H	2.81616800	2.85617400	0.19344800
H	3.00872500	4.37444900	-0.67298900
Pd	0.01133400	-0.29981600	-0.59278800
C	3.39661100	1.74036200	-3.43639400
C	3.43059100	0.36170500	-3.25760300
C	2.43021500	2.48750400	-2.77147500
C	1.51604400	1.85724600	-1.91112700
C	1.57041900	0.46295700	-1.71381200
C	2.52088800	-0.29909300	-2.41614200
C	2.59173900	-1.80245500	-2.35043100
H	4.10570900	2.22821300	-4.09992300
H	4.16869600	-0.23364200	-3.79124500
H	2.36773000	3.56258700	-2.92001700
H	1.87128000	-2.23610800	-1.65685500
H	2.38430100	-2.22938600	-3.34031500
H	3.59383500	-2.13403500	-2.05732700
C	-1.65388700	-1.06868600	2.49561500
C	-0.66988100	-2.24358800	2.62756800
C	0.82492700	-1.90932200	2.49665500
P	-1.97747500	-0.71848900	0.70867300
C	-3.11353700	-2.04193500	0.17394200
C	-4.35195000	-2.22064600	0.81246300
C	-5.22076700	-3.21993400	0.38407500
C	-4.85963900	-4.04452000	-0.68723900
C	-3.63272700	-3.86775200	-1.32391200
C	-2.75565600	-2.86727000	-0.89749500
C	-3.08614100	0.74817400	0.71201200
C	-3.99407100	0.90811000	-0.34944300

C	-4.84536300	2.01059100	-0.39650900
C	-4.79783300	2.97988800	0.60952700
C	-3.88887700	2.83814300	1.65767700
C	-3.03959000	1.73128200	1.71002600
P	1.37829200	-0.71021400	1.17676000
C	3.12064900	-1.14281000	0.84562800
C	3.55409900	-2.47504000	0.82954100
C	4.88128700	-2.76994900	0.51523200
C	5.77651400	-1.74898800	0.19600000
C	5.34147000	-0.42138200	0.18806300
C	4.02141200	-0.12007200	0.51020700
C	1.49677900	0.83129500	2.18506500
C	0.50273500	1.80951300	2.08240300
C	0.48963700	2.90510500	2.94749500
C	1.48827900	3.04796700	3.90958100
C	2.49877800	2.08662200	4.00703200
C	2.49829700	0.98148800	3.15830400
H	-2.60661500	-1.30695500	2.97984300
H	-1.25639600	-0.16655400	2.97155300
H	-0.89917200	-3.01330500	1.88397200
H	-0.80643700	-2.70185100	3.61436800
H	1.34475800	-2.84591000	2.29439800
H	1.21106400	-1.48475400	3.43070300
H	-4.64248900	-1.56836200	1.63190800
H	-6.17676300	-3.35724900	0.88171800
H	-5.53703500	-4.82673000	-1.01901200
H	-3.33983600	-4.51683700	-2.14329000
H	-1.78029600	-2.76319500	-1.35813000
H	-4.05123800	0.14898200	-1.12296700
H	-5.55567100	2.10662800	-1.21344800
H	-5.46645800	3.83537000	0.57693300
H	-3.84477700	3.58458500	2.44586100
H	-2.34738200	1.63916000	2.53940500
H	2.84596600	-3.28269300	0.98018600
H	5.20488300	-3.80646800	0.49884600
H	6.80614900	-1.98547600	-0.05795800
H	6.02561400	0.37908000	-0.07805400
H	3.68832400	0.91106600	0.48966300

H	-0.25145200	1.72287700	1.30889000
H	-0.29163200	3.65215000	2.84747400
H	1.48508800	3.90392600	4.57863100
H	3.28480200	2.19564700	4.74893600
H	3.28306400	0.23627100	3.24402000
Br	0.45651600	-3.93431300	-0.23931000

Int-5'

B3LYP-D3(BJ) SCF energy = -2561.459667 a.u.

M06L SCF energy in solution = -2562.931126 a.u.

M06L free energy in solution = -2562.319325 a.u.

C	1.44297000	3.51481400	0.83948100
C	0.68582300	4.58656100	0.35409200
C	-0.68668400	4.66628500	0.56973700
C	-1.32056100	3.66848500	1.29962700
C	-0.59092300	2.57285700	1.78146700
C	0.80582600	2.48497900	1.56467800
C	-1.34744700	1.53599600	2.54174900
C	-2.20442300	1.99148000	3.69505100
O	-1.30381900	0.32981600	2.28051500
C	2.91869300	3.45661600	0.52527500
H	1.18659500	5.35985500	-0.22224400
H	-1.26210700	5.49517600	0.16904500
H	-2.39133500	3.71577500	1.45243800
H	-2.88518500	2.79190900	3.38884400
H	-2.76984500	1.14855100	4.09577600
H	-1.55519300	2.40872200	4.47474800
H	3.53617500	3.75938700	1.37792000
H	3.21825600	2.44086600	0.25680500
H	3.15349800	4.11348000	-0.31698000
Pd	-0.05008800	-0.37543900	0.51260700
C	3.33056400	0.80063800	3.79253700
C	3.18344600	-0.53516900	3.43099700
C	2.54491600	1.76690500	3.17151700
C	1.63008700	1.39658000	2.17280300
C	1.50344800	0.04694800	1.79330400
C	2.26800200	-0.93706600	2.44521800

C	2.12611500	-2.41295000	2.16584700
H	4.04032700	1.08634700	4.56435100
H	3.77881800	-1.29740700	3.92976600
H	2.62526500	2.81140600	3.46210600
H	3.08245800	-2.84492700	1.85081700
H	1.81704300	-2.93830200	3.07858700
H	1.38167300	-2.63290500	1.39878700
C	-1.84685200	-1.29364900	-2.55396800
C	-0.54870700	-0.92341400	-3.29393200
C	0.73135500	-1.52747900	-2.69832200
P	-2.02245100	-0.63161900	-0.83427900
C	-2.83957400	1.01048900	-1.02418400
C	-2.52230000	1.88266800	-2.07590300
C	-3.09305200	3.15418000	-2.14774100
C	-4.00058200	3.57396300	-1.17484800
C	-4.32913100	2.71238300	-0.12583700
C	-3.74765400	1.44779000	-0.04483300
C	-3.37121700	-1.61440300	-0.09319600
C	-3.15357300	-2.30393800	1.10497700
C	-4.19428900	-3.02624000	1.68971400
C	-5.44753400	-3.07362900	1.07832300
C	-5.66698600	-2.39240600	-0.12167100
C	-4.63346000	-1.66044900	-0.70311600
P	1.39542700	-0.65166100	-1.20826100
C	1.84747200	0.95948900	-1.96119000
C	0.93106000	2.01521000	-1.88866800
C	1.14946300	3.19236200	-2.60526400
C	2.30245800	3.33815200	-3.37558700
C	3.23306600	2.29649300	-3.43735600
C	3.00218700	1.10876400	-2.74582200
C	2.97643000	-1.48088800	-0.85719400
C	3.12618000	-2.85509300	-1.08928300
C	4.32444700	-3.48802700	-0.75748200
C	5.36926100	-2.76403400	-0.18218300
C	5.21510200	-1.39800400	0.06832100
C	4.02372800	-0.75925800	-0.26448100
H	-2.71728900	-0.97909400	-3.14084300
H	-1.88809000	-2.37914300	-2.41963900

H	-0.44029900	0.16224900	-3.39866900
H	-0.64343300	-1.31141600	-4.31560400
H	1.53810500	-1.49364000	-3.43963300
H	0.54781600	-2.56337400	-2.39965900
H	-1.82893600	1.57518400	-2.85013800
H	-2.83574800	3.81098400	-2.97426700
H	-4.45373800	4.55926400	-1.23724400
H	-5.04592800	3.02303800	0.63014400
H	-4.01766600	0.78256100	0.76858500
H	-2.16728400	-2.30321700	1.55159100
H	-4.01624500	-3.56699300	2.61445100
H	-6.25300700	-3.64443100	1.53237200
H	-6.64059400	-2.43041900	-0.60238000
H	-4.81350700	-1.11430600	-1.62508100
H	0.05309700	1.91892900	-1.25994100
H	0.42322500	3.99651600	-2.53955200
H	2.47945500	4.25821600	-3.92568200
H	4.13539700	2.40676700	-4.03216700
H	3.72095800	0.29810400	-2.80905500
H	2.29414100	-3.44133300	-1.46233900
H	4.42931700	-4.55464900	-0.93222700
H	6.29730600	-3.26337100	0.08211500
H	6.01698200	-0.83185600	0.53315100
H	3.90163500	0.29626400	-0.04799700
Br	-0.39014400	-3.80451100	-0.33208300

Int-6

B3LYP-D3(BJ) SCF energy = -2311.213069 a.u.

M06L SCF energy in solution = -2312.543201 a.u.

M06L free energy in solution = -2312.081084 a.u.

C	-1.01793800	-0.83407000	-2.15695900
C	0.00007100	0.00027500	-2.96231400
C	1.01795700	0.83451200	-2.15671200
P	-1.64117000	-0.01736400	-0.60746600
C	-2.18659100	1.60440000	-1.23490000
C	-1.77606300	2.77527600	-0.58450700

C	-2.09932400	4.02051600	-1.12038300
C	-2.84687700	4.10706800	-2.29716700
C	-3.27069600	2.94378600	-2.94167500
C	-2.93427200	1.69524300	-2.41735400
C	-3.08305400	-1.04402500	-0.18882200
C	-4.39128400	-0.54899800	-0.21408200
C	-5.45429200	-1.38313400	0.13082000
C	-5.21695400	-2.70567600	0.51167700
C	-3.90999500	-3.19542500	0.55419400
C	-2.84393100	-2.36581000	0.21020800
P	1.64117800	0.01746300	-0.60738900
C	3.08291400	1.04419500	-0.18840000
C	4.39113700	0.54911400	-0.21301400
C	5.45403000	1.38327400	0.13219500
C	5.21657500	2.70589000	0.51272200
C	3.90961700	3.19569400	0.55459300
C	2.84367100	2.36606100	0.21029300
C	2.18684100	-1.60402200	-1.23531000
C	1.77608600	-2.77517800	-0.58557000
C	2.09954300	-4.02018600	-1.12186600
C	2.84752300	-4.10622400	-2.29841700
C	3.27156800	-2.94266100	-2.94226800
C	2.93494900	-1.69434600	-2.41753100
H	-1.86677800	-1.09390900	-2.79774300
H	-0.56760100	-1.77773300	-1.83346700
H	-0.54533700	0.69101700	-3.61355500
H	0.54557600	-0.69037900	-3.61356800
H	0.56753000	1.77807000	-1.83304200
H	1.86681800	1.09454500	-2.79739000
H	-1.23744600	2.69247500	0.35215300
H	-1.77863000	4.92456600	-0.61105700
H	-3.10284300	5.07923200	-2.70885800
H	-3.85840200	3.00634600	-3.85304200
H	-3.26279100	0.79405800	-2.92722100
H	-4.57063200	0.48831000	-0.47128000
H	-6.46846700	-0.99445100	0.11490200
H	-6.04745900	-3.34963000	0.78667700
H	-3.71860200	-4.21720400	0.86865300

H	-1.82469700	-2.73633200	0.27720900
H	4.57054700	-0.48824800	-0.46994300
H	6.46819900	0.99455500	0.11676400
H	6.04698400	3.34986400	0.78796500
H	3.71812900	4.21753600	0.86878900
H	1.82442700	2.73664900	0.27677300
H	1.23712800	-2.69278600	0.35093100
H	1.77867600	-4.92445900	-0.61304400
H	3.10365000	-5.07821000	-2.71043000
H	3.85960300	-3.00482400	-3.85345000
H	3.26365100	-0.79293600	-2.92688300
Pd	-0.00006100	-0.00020100	0.99803700
C	-2.48620000	0.39774000	2.51735400
O	-2.77134600	1.24845900	1.65775100
O	-1.40916600	-0.31044500	2.53860100
C	-3.48586400	0.06108400	3.61457300
H	-2.98917900	-0.33947600	4.50067900
H	-4.07522700	0.94510000	3.86930200
H	-4.16823000	-0.70349900	3.22269600
C	2.48598500	-0.39868600	2.51732000
O	2.77105800	-1.24910400	1.65738100
O	1.40907500	0.30966000	2.53878000
C	3.48568900	-0.06263800	3.61469600
H	2.98911800	0.33812900	4.50077000
H	4.07452500	-0.94700400	3.86945300
H	4.16855600	0.70155000	3.22293600

TS1

B3LYP-D3(BJ) SCF energy = -1334.642747 a.u.

M06L SCF energy in solution = -1336.139757 a.u.

M06L free energy in solution = -1335.760053 a.u.

Imaginary Frequency = -140.21 cm^{-1}

C	-2.20845200	1.25733700	1.53222200
C	-2.83079700	0.64437100	2.62637000
C	-3.43009600	-0.61440100	2.54482900
C	-3.36596300	-1.32182600	1.35313800

C	-2.72292300	-0.77777000	0.23153500
C	-2.16473900	0.52035800	0.33644300
Br	-2.30210300	1.75990400	-1.44260800
C	-2.46286100	-1.61386300	-0.95157900
C	-3.26950400	-2.85902100	-1.20399500
O	-1.53763600	-1.32757300	-1.72392900
C	-1.62894300	2.63466300	1.67923500
H	-2.86255000	1.19332200	3.56371400
H	-3.92535900	-1.04066400	3.41128900
H	-3.76314600	-2.32891300	1.29514700
H	-2.89052900	-3.64804700	-0.54264200
H	-4.33465700	-2.71624200	-1.00210400
H	-3.11802500	-3.17493900	-2.23747800
H	-1.77948400	3.00637200	2.69635200
H	-0.55556400	2.62412600	1.46503000
H	-2.09126300	3.34014900	0.98122200
Pd	-0.11986900	0.22383100	-0.85885300
C	2.13833200	3.72531300	0.09333400
C	3.29198500	3.18891800	0.66486800
C	1.11550400	2.88054400	-0.34346400
C	1.26384100	1.49478000	-0.21747900
C	2.44895300	0.95210300	0.31338700
C	3.46778000	1.80560200	0.78054200
C	4.72505600	1.25243200	1.40929700
H	2.02958000	4.80278200	-0.00285800
H	4.07383700	3.85301100	1.02621700
H	0.21248600	3.30187200	-0.77321200
H	5.33139100	2.05475000	1.84129800
H	4.49297200	0.53739200	2.20818300
H	5.34953300	0.71945900	0.68244300
C	2.43757900	-3.29852000	-0.69472300
C	1.56317400	-2.13965600	-1.24830700
C	1.20395200	-1.20067800	-0.08299100
C	2.55981200	-0.54138200	0.32157100
C	3.79893800	-2.60181200	-0.37967900
C	3.52804000	-1.12913200	-0.75595600
C	2.61768600	-1.29343200	-1.98793300
C	0.39693400	-1.81752300	1.00081800

O	-0.42725300	-2.71049200	0.85110900
O	0.66244300	-1.28372900	2.22022200
C	-0.06233400	-1.85328600	3.31477800
H	2.55567500	-4.07306700	-1.46062500
H	1.98104900	-3.77435100	0.17715300
H	0.70037800	-2.47812400	-1.82130000
H	2.87480600	-0.88993600	1.31197700
H	4.09676900	-2.70828900	0.66927200
H	4.60620900	-3.01613200	-0.99360300
H	4.42853100	-0.52975700	-0.90456100
H	2.22340900	-0.34559900	-2.36815400
H	3.10578900	-1.83471900	-2.80630200
H	0.29819500	-1.33402900	4.20381800
H	-1.13524800	-1.69739900	3.19041000
H	0.13665400	-2.92627300	3.39108500

TS1'

B3LYP-D3(BJ) SCF energy = -1334.637875 a.u.

M06L SCF energy in solution = -1336.137609 a.u.

M06L free energy in solution = -1335.759321 a.u.

Imaginary Frequency = -127.55 cm^{-1}

C	1.96888200	1.39549000	1.68417600
C	2.42487900	0.85561800	2.89263900
C	3.04765400	-0.39170500	2.97561100
C	3.20055800	-1.15396600	1.82572700
C	2.71609000	-0.68898200	0.59394500
C	2.09643700	0.58559500	0.54226600
Br	2.42669300	1.70487000	-1.28339600
C	2.66894000	-1.57718300	-0.57940300
C	3.66244000	-2.69909200	-0.73690000
O	1.77706900	-1.42688600	-1.42354800
C	1.36339900	2.76947700	1.65349600
H	2.30312400	1.45309700	3.79216400
H	3.40509200	-0.76055500	3.93128100
H	3.65079600	-2.14006700	1.87843300
H	4.68009100	-2.37611300	-0.49812300

H	3.39913000	-3.51918200	-0.05706000
H	3.61021400	-3.07643100	-1.75926400
H	1.30682100	3.18608700	2.66280500
H	1.95513100	3.44964800	1.03154500
H	0.35490300	2.74018400	1.22775800
Pd	0.21953700	0.13370800	-0.79688300
C	-2.22454900	3.62491200	-0.67788200
C	-3.41244600	3.14520800	-0.12661200
C	-1.13059600	2.77129700	-0.83566400
C	-1.23311400	1.43689900	-0.42623400
C	-2.42664800	0.95626300	0.14297200
C	-3.53565700	1.81312800	0.28386700
C	-4.84802900	1.31061200	0.83778200
H	-2.15258800	4.66220000	-0.99539900
H	-4.26563600	3.81141100	-0.02140700
H	-0.21251300	3.14399600	-1.27835300
H	-5.62775600	2.07336300	0.74803600
H	-5.19042500	0.41493400	0.30479700
H	-4.77200200	1.03613400	1.89665800
C	-1.46372300	-2.96814900	1.81693300
C	-0.54496400	-1.81557900	1.32422500
C	-1.14973300	-1.21347600	0.04647800
C	-2.44313500	-0.48647300	0.54242100
C	-2.72949200	-2.21762100	2.33701200
C	-2.38113800	-0.73599000	2.08166100
C	-0.86660800	-0.71796800	2.35613100
C	-1.29808400	-2.16782800	-1.09097800
O	-0.65742600	-3.19484000	-1.23700700
O	-2.23349900	-1.75944800	-1.99032400
C	-2.35752400	-2.58455000	-3.15332700
H	-0.96623100	-3.51212200	2.62794200
H	-1.67635700	-3.68827600	1.02491200
H	0.49830100	-2.11267500	1.22577000
H	-3.33421500	-0.97563500	0.13033800
H	-3.64383900	-2.51854400	1.81368500
H	-2.88884400	-2.40070100	3.40539900
H	-2.97941900	-0.02778900	2.65890200
H	-0.39934300	0.24079400	2.12800100

H	-0.61161000	-1.00844000	3.38165000
H	-3.14508900	-2.12821000	-3.75393100
H	-1.41564000	-2.60664000	-3.70920800
H	-2.62722500	-3.60771700	-2.87689000

TS2

B3LYP-D3(BJ) SCF energy = -1334.634782 a.u.

M06L SCF energy in solution = -1336.139177 a.u.

M06L free energy in solution = -1335.757789 a.u.

Imaginary Frequency = -317.39 cm^{-1}

C	-1.05859700	1.49307000	1.92514600
C	-2.05055200	1.70862200	2.89637300
C	-3.39003400	1.39141500	2.70413200
C	-3.77522100	0.86386300	1.48354800
C	-2.82421000	0.60233900	0.48555000
C	-1.43284200	0.87038000	0.71231200
Br	-0.82104700	-3.11919900	-0.83605200
C	-3.29016100	0.00210200	-0.77771100
C	-4.75981200	-0.08850900	-1.10894800
O	-2.47688900	-0.44098900	-1.60033200
C	0.30037000	2.06098200	2.24871100
H	-1.74789700	2.16975700	3.83233000
H	-4.11919500	1.57546400	3.48631000
H	-4.81634300	0.62206200	1.30387800
H	-5.24083400	-0.83505800	-0.46573100
H	-5.27268400	0.86652100	-0.95862200
H	-4.86015000	-0.40919600	-2.14664400
H	1.10142600	1.32650900	2.24258200
H	0.56976900	2.84177100	1.53146600
H	0.28286200	2.51130000	3.24491400
Pd	-0.51041700	-0.58802000	-0.56952300
C	-0.58323600	3.56842800	-2.00999200
C	0.74862700	3.90290900	-1.76324300
C	-1.09549100	2.37834100	-1.51262100
C	-0.26783100	1.50478400	-0.79197800
C	1.11113500	1.78031500	-0.64211500

C	1.60801000	3.02502500	-1.09790800
C	3.03968400	3.45906000	-0.86582400
H	-1.22847000	4.25144300	-2.55479600
H	1.13390800	4.86324900	-2.09677000
H	-2.14368900	2.14912400	-1.65185400
H	3.08428700	4.54620100	-0.74301400
H	3.47267500	3.00107100	0.02768400
H	3.69298500	3.20324100	-1.70822400
C	3.68184100	-1.72854200	0.00712700
C	2.22546600	-1.67685600	-0.53046700
C	1.37633700	-0.63749800	0.25941500
C	2.06031700	0.73046500	-0.10595900
C	4.28764600	-0.36809300	-0.44556900
C	3.10763100	0.29092500	-1.18958100
C	2.43450800	-0.91879200	-1.85334800
C	1.24363200	-0.97406000	1.72578900
O	0.31567600	-1.54257600	2.25401300
O	2.33846200	-0.57125300	2.43918900
C	2.29527400	-0.88614800	3.83878000
H	4.19179200	-2.56918800	-0.47577900
H	3.73906000	-1.88279700	1.08454800
H	1.75074800	-2.65120300	-0.58709100
H	2.60195700	1.12772900	0.75804400
H	4.63311800	0.24058600	0.39677500
H	5.13775100	-0.51189500	-1.12110400
H	3.39600000	1.09680400	-1.86272200
H	1.50318500	-0.67839900	-2.37596800
H	3.09856300	-1.44987300	-2.54368000
H	2.20386500	-1.96484500	3.98646700
H	3.23484100	-0.51659200	4.25064500
H	1.44336300	-0.39154300	4.31355400

TS2'

B3LYP-D3(BJ) SCF energy = -1334.624366 a.u.

M06L SCF energy in solution = -1336.127162 a.u.

M06L free energy in solution = -1335.745885 a.u.

Imaginary Frequency = -211.39 cm^{-1}

C	1.60152400	2.03407200	1.44793500
C	2.74654300	2.29653000	2.22574300
C	3.95325700	1.63627100	2.05660800
C	4.05376200	0.72389000	1.01905900
C	2.93895100	0.40376700	0.23002100
C	1.64884100	0.98233800	0.50475000
Br	0.51953400	-3.10144800	0.49614400
C	3.14988600	-0.54470900	-0.87987100
C	4.53732700	-0.96882100	-1.29947700
O	2.18710200	-1.00231300	-1.50732600
C	0.48574000	3.02994400	1.67767500
H	2.67269600	3.08600200	2.96975800
H	4.80733700	1.86455500	2.68567900
H	5.00018600	0.23428000	0.82287900
H	4.97231400	-1.62477600	-0.53595300
H	4.45593600	-1.52957500	-2.23148700
H	5.20715300	-0.11409900	-1.43321600
H	0.91756000	4.03656500	1.69745400
H	-0.26596500	3.00920100	0.89781100
H	-0.01003000	2.87125300	2.63950400
Pd	0.37117900	-0.65262700	-0.27441400
C	0.42359300	2.97684000	-2.71091000
C	-0.87755100	3.40725200	-2.45056100
C	0.96768400	1.93672900	-1.96583400
C	0.21060600	1.32828700	-0.95832100
C	-1.12655300	1.72783900	-0.71370600
C	-1.66861000	2.78304000	-1.48138500
C	-3.09669600	3.23470800	-1.28269300
H	1.01445300	3.45783200	-3.48514500
H	-1.29686500	4.23205100	-3.02079200
H	1.98279000	1.60963200	-2.15559200
H	-3.29369700	4.15152200	-1.84605600
H	-3.81064100	2.47538000	-1.62827800
H	-3.32449600	3.42958400	-0.22930000

C	-3.35261200	-0.70144900	2.21915500
C	-1.82596400	-0.78520000	1.91592500
C	-1.58153300	-0.52818500	0.42799800
C	-2.03870400	0.93906500	0.20523600
C	-3.66579600	0.82127800	2.08780400
C	-2.30282700	1.41758000	1.67093600
C	-1.34248200	0.54416400	2.51226500
C	-2.20712400	-1.51069800	-0.53610400
O	-3.01620300	-2.37159000	-0.27642500
O	-1.74358200	-1.29329100	-1.79973400
C	-2.10395600	-2.29242600	-2.76813800
H	-3.52484900	-1.05205500	3.24227200
H	-3.93972400	-1.32848300	1.55079100
H	-1.35064300	-1.69607200	2.27441100
H	-3.00837400	0.91207300	-0.30705200
H	-4.45033700	1.03257800	1.35258200
H	-3.99112800	1.24747200	3.04307700
H	-2.23670200	2.49850300	1.80343500
H	-0.28286200	0.71000100	2.33378300
H	-1.54188200	0.62768600	3.58655900
H	-3.18996900	-2.35114200	-2.87144300
H	-1.64085700	-1.97068700	-3.70056900
H	-1.71169300	-3.26282200	-2.45387800

TS3

B3LYP-D3(BJ) SCF energy = -3062.081368 a.u.

M06L SCF energy in solution = -3063.580805 a.u.

M06L free energy in solution = -3062.782341 a.u.

Imaginary Frequency = -206.37 cm^{-1}

C	-2.98275800	3.19358700	-2.06769800
C	-3.09718300	4.54201900	-1.71691900
C	-3.23862500	4.93434600	-0.38798900
C	-3.28656700	3.96398200	0.60372500

C	-3.15919700	2.60634900	0.27921800
C	-2.97078400	2.20238400	-1.05113600
Br	3.48945900	-2.44229700	-2.09187700
C	-3.39144900	1.66433300	1.42594400
C	-4.65296600	0.84583600	1.35001800
O	-2.67893300	1.67335800	2.41892600
C	-2.90347500	2.82485600	-3.53252900
H	-3.08879200	5.29130200	-2.50392700
H	-3.33047600	5.98594800	-0.13257900
H	-3.43646700	4.24391200	1.64187500
H	-4.57865200	0.11090900	0.54576900
H	-4.82537100	0.33362400	2.29469300
H	-5.49639800	1.50448400	1.11021000
H	-3.85712700	2.41033800	-3.88017900
H	-2.67866400	3.70608100	-4.14017500
H	-2.14699100	2.06391500	-3.73607400
Pd	0.19176200	0.02527400	-0.23157900
C	-3.94641500	-0.94751700	-2.80452600
C	-2.71823000	-1.54507500	-3.06091400
C	-3.97508100	0.21835500	-2.05009700
C	-2.80234500	0.77854600	-1.51570900
C	-1.55732100	0.10971800	-1.69402700
C	-1.52282100	-1.03252700	-2.53323800
C	-0.25707800	-1.74214200	-2.93932400
H	-4.86295400	-1.36505000	-3.21155100
H	-2.66700500	-2.43846500	-3.67820000
H	-4.91152500	0.75017300	-1.90488400
H	0.11782600	-1.35148400	-3.89221300
H	0.55180900	-1.63492800	-2.22132300
H	-0.43597400	-2.81122400	-3.07325000
C	2.22340900	3.12925100	-2.83486800
C	2.28660700	1.83546200	-1.96494700
C	1.03558400	1.87828900	-1.08477400
C	-0.07609800	1.65819400	-1.97701300

C	1.00365600	2.86270400	-3.76618600
C	0.54314500	1.42753500	-3.34280800
C	1.89951700	0.77729100	-3.00307500
C	0.98568100	2.91777600	-0.02437600
O	1.90269900	3.68029700	0.21683500
O	-0.17953600	2.93926500	0.66029300
C	-0.24822700	3.90560300	1.72119600
H	3.14983500	3.21266600	-3.41191400
H	2.12334400	4.03272900	-2.23209400
H	3.22484100	1.69835700	-1.43739200
H	-0.91350300	2.32469900	-1.87250900
H	0.20591700	3.60438900	-3.65026600
H	1.29060200	2.85192600	-4.82243600
H	-0.09871100	0.93371800	-4.07211800
H	1.86525700	-0.22859200	-2.59179200
H	2.58087300	0.76219400	-3.85862300
H	-1.12334000	3.62315600	2.29979100
H	0.65424000	3.85850100	2.33258000
H	-0.35675300	4.91070200	1.30528200
C	2.55599900	-1.84880900	1.51515100
C	1.61014600	-2.75050600	2.33929200
C	0.12215600	-2.36819500	2.43839800
P	2.03010500	-0.09438700	1.27054600
C	1.53946000	0.48686300	2.93522400
C	2.41716100	0.35313500	4.02256100
C	2.00242700	0.71154400	5.30383500
C	0.70994600	1.20420800	5.51121900
C	-0.16462200	1.34368500	4.43314800
C	0.25084100	0.98781200	3.14910400
C	3.61277600	0.75866200	0.96401500
C	3.89135600	1.99291600	1.56946900
C	5.09932900	2.63862400	1.31276700
C	6.03466500	2.06173000	0.45257000
C	5.75777300	0.83445500	-0.15271600

C	4.55210100	0.17969400	0.09556900
P	-0.71387000	-1.98106800	0.82830500
C	-2.48351000	-2.00288800	1.31469200
C	-3.44409900	-2.29760500	0.33517100
C	-4.76878000	-2.53998200	0.69250900
C	-5.15632100	-2.48219800	2.03307500
C	-4.21764300	-2.14197400	3.00740300
C	-2.89027200	-1.89433700	2.65170000
C	-0.52952300	-3.61388900	0.00311700
C	0.62761200	-3.85336900	-0.74221700
C	0.88813100	-5.11939700	-1.26679000
C	-0.00976600	-6.16274300	-1.04818100
C	-1.16136700	-5.93713300	-0.28791900
C	-1.42030800	-4.67500900	0.24392500
H	3.54220800	-1.82500200	1.98863400
H	2.71936700	-2.23835700	0.50159500
H	1.97764300	-2.79507600	3.37032600
H	1.68640200	-3.76396200	1.93395200
H	-0.00225800	-1.50663600	3.09552000
H	-0.42274700	-3.20724400	2.88331100
H	3.42384300	-0.02243500	3.86414200
H	2.68583000	0.60468000	6.14147700
H	0.38967600	1.47805100	6.51269400
H	-1.17296200	1.71950400	4.57211600
H	-0.43620500	1.09382200	2.31842400
H	3.17138800	2.45340200	2.23416200
H	5.30222700	3.59775900	1.78008300
H	6.97425600	2.56966500	0.25246200
H	6.47272200	0.37878800	-0.83146100
H	4.33672500	-0.75270300	-0.42457400
H	-3.14667400	-2.38078100	-0.70247500
H	-5.49384900	-2.78335500	-0.07887400
H	-6.18487100	-2.68872500	2.31462800
H	-4.51328300	-2.07265200	4.05039200

H	-2.17723600	-1.63547000	3.42544000
H	1.35362400	-3.07364100	-0.94901600
H	1.79764400	-5.25213400	-1.84506200
H	0.18416900	-7.14937000	-1.46021800
H	-1.86135300	-6.74763500	-0.10316600
H	-2.31149600	-4.52491900	0.84167900

TS3'

B3LYP-D3(BJ) SCF energy = -3062.075796 a.u.

M06L SCF energy in solution = -3063.577657 a.u.

M06L free energy in solution = -3062.779408 a.u.

Imaginary Frequency = -170.37 cm^{-1}

C	4.55657700	0.19401800	-2.25219200
C	5.67258100	1.03253600	-2.16276700
C	6.04006900	1.62682200	-0.96149000
C	5.26939200	1.38496500	0.16585000
C	4.10739700	0.59611000	0.09964700
C	3.72645900	-0.01975300	-1.11860700
Br	-4.56580000	-2.25096600	-1.68400300
C	3.31289400	0.58678900	1.36300100
C	4.04451300	0.49581300	2.68981400
O	2.10106700	0.76446000	1.36468100
C	4.29366000	-0.46429600	-3.59245300
H	6.27068500	1.20236200	-3.05421900
H	6.91693000	2.26447300	-0.90345000
H	5.54979700	1.84027300	1.10947400
H	3.37434500	0.04802000	3.42504200
H	4.96727800	-0.08323500	2.62577500
H	4.29390400	1.50854400	3.03279900
H	3.23322000	-0.64852600	-3.77284400
H	4.68277400	0.16258900	-4.40143200
H	4.79474800	-1.43675900	-3.65944900
Pd	0.00247100	0.26612300	-0.16848500

C	2.46275100	-3.41681900	-1.99189900
C	1.12852800	-3.21053600	-2.32032000
C	3.21673700	-2.34267300	-1.53911200
C	2.65340800	-1.06585500	-1.32584900
C	1.25760600	-0.91131600	-1.52572900
C	0.51827400	-1.96852700	-2.11354600
C	-0.91651600	-1.81572100	-2.55248800
H	2.91948800	-4.39417600	-2.11656000
H	0.52682000	-4.02715300	-2.70730600
H	4.28061200	-2.47002800	-1.35456100
H	-0.96997400	-1.34464400	-3.54393900
H	-1.41848000	-2.77953300	-2.63088300
H	-1.51663600	-1.19692700	-1.88721600
C	0.36746300	3.87094700	-2.89427000
C	0.44882600	3.18975700	-1.49679000
C	-0.32834000	1.87683000	-1.65154100
C	0.49488100	1.02671600	-2.45481400
C	1.20823400	2.91558700	-3.78833400
C	1.72813200	1.83992600	-2.77360200
C	1.89705400	2.68803800	-1.49932400
C	-1.80287700	2.01109000	-1.84756500
O	-2.42198700	2.98343400	-1.44779000
O	-2.34202800	1.00465500	-2.54140500
C	-3.75842600	1.08626400	-2.83762200
H	0.82108800	4.86628700	-2.84145400
H	-0.66271900	3.99652400	-3.23435900
H	0.13718500	3.82673300	-0.67848700
H	0.04975300	0.37479500	-3.19148600
H	0.62924600	2.46703600	-4.60188700
H	2.06557700	3.42600200	-4.23914200
H	2.58425800	1.28842100	-3.13755000
H	2.13526300	2.09934500	-0.61623400
H	2.63243200	3.48988900	-1.61974100
H	-4.09815600	0.05339400	-2.92717400

H	-4.27750300	1.58961900	-2.02280400
H	-3.88412400	1.65196300	-3.76604100
C	-1.51359200	-1.53983700	2.56251900
C	-1.54569400	-0.35389400	3.54940100
C	-0.74913300	0.88879300	3.14157700
P	-0.08285600	-1.69254100	1.38131300
C	1.37258000	-2.07459000	2.43477000
C	1.30705300	-2.11318900	3.83362000
C	2.42403100	-2.47670300	4.59144300
C	3.61859400	-2.82677000	3.96346500
C	3.69284200	-2.79783100	2.56917400
C	2.58611300	-2.41451900	1.81549100
C	-0.48940700	-3.32856200	0.65299100
C	0.34198000	-4.45327700	0.76673300
C	-0.04404100	-5.66726800	0.20115500
C	-1.26308500	-5.77733000	-0.47265100
C	-2.10094100	-4.66692500	-0.57219000
C	-1.71490000	-3.44878500	-0.01351600
P	-1.18656400	1.51557900	1.44835100
C	-0.69747800	3.26931700	1.58206200
C	-1.54591900	4.31212300	1.18655200
C	-1.10031700	5.63308100	1.24660200
C	0.18996500	5.92419100	1.69134900
C	1.04010800	4.88647600	2.08101000
C	0.60134300	3.56508000	2.02705400
C	-3.00200900	1.46028600	1.44401300
C	-3.65971900	0.55322300	0.60533900
C	-5.04758200	0.41103000	0.66873500
C	-5.78054900	1.18291900	1.56942700
C	-5.13092900	2.08996600	2.41388400
C	-3.74613100	2.22622600	2.35855500
H	-1.60447300	-2.48938200	3.09899400
H	-2.37885100	-1.48437900	1.89552400
H	-1.17896700	-0.67194800	4.53099600

H	-2.59257300	-0.07029100	3.69574300
H	0.32647700	0.68616300	3.12983300
H	-0.93111300	1.69366400	3.86112000
H	0.38318200	-1.88388800	4.34723200
H	2.34913700	-2.50146000	5.67491400
H	4.48136800	-3.12160000	4.55370000
H	4.61457400	-3.07182200	2.06309700
H	2.65546700	-2.40506000	0.73679500
H	1.28765200	-4.39290300	1.28978300
H	0.61125700	-6.52962400	0.29237900
H	-1.55804700	-6.72620700	-0.91269500
H	-3.05420400	-4.69801700	-1.09228700
H	-2.39845500	-2.61420500	-0.15003100
H	-2.53178400	4.09011300	0.80026000
H	-1.76282800	6.43427200	0.93233900
H	0.53387200	6.95391500	1.73035400
H	2.04787000	5.10628200	2.42277000
H	1.27473900	2.76028200	2.30054500
H	-3.11380300	-0.05791400	-0.10809700
H	-5.51061000	-0.31830200	0.00770000
H	-6.86072500	1.07699000	1.61975500
H	-5.70292300	2.68805700	3.11815000
H	-3.24462800	2.92850200	3.01845200

TS4

B3LYP-D3(BJ) SCF energy = -2561.437757 a.u.

M06L SCF energy in solution = -2562.904248 a.u.

M06L free energy in solution = -2562.291008 a.u.

Imaginary Frequency = -207.31 cm^{-1}

C	1.82126600	3.52271700	-1.02850900
C	0.83144700	4.51371900	-1.02583600
C	-0.44043000	4.29005400	-1.55621000
C	-0.75153200	3.05515500	-2.12239800
C	0.22787200	2.06632100	-2.16263000

C	1.50554800	2.27753900	-1.60716400
C	-0.06873500	0.70785100	-2.75269000
C	0.36608500	0.48001300	-4.18985300
O	-1.14140200	0.09739900	-2.36093900
C	3.13862500	3.79141300	-0.34389600
H	1.05759900	5.47303100	-0.56625200
H	-1.19255700	5.07165700	-1.51433100
H	-1.74007700	2.85425000	-2.51691000
H	0.35057300	-0.58444900	-4.42941300
H	-0.36096300	0.99146600	-4.83546200
H	1.35855900	0.89361400	-4.38027100
H	3.04713200	4.63738200	0.34359600
H	3.46987500	2.92308000	0.23410400
H	3.93391900	4.03405000	-1.06005200
Pd	-0.20437200	-0.42675400	-0.51817400
C	4.37130800	-0.14610800	-2.14074200
C	3.63688500	-1.31084300	-2.35633900
C	3.72990700	1.05272700	-1.83053200
C	2.33936300	1.06384300	-1.66553500
C	1.60804600	-0.14553100	-1.78351500
C	2.24230800	-1.32614600	-2.21655700
C	1.47094900	-2.57390400	-2.53372100
H	5.45071400	-0.16130900	-2.26408700
H	4.14821000	-2.22583800	-2.64359800
H	4.30236900	1.97110600	-1.78433200
H	1.29676100	-3.18165300	-1.63867200
H	0.47291900	-2.33789700	-2.91450100
H	2.00385200	-3.18140800	-3.27331800
C	-1.97419700	-0.36703800	2.56028300
C	-1.25978200	-1.68810800	2.89438300
C	0.27609400	-1.68013100	2.77364300
P	-2.15800300	-0.15758900	0.73294000
C	-3.56706000	-1.20644200	0.25008600
C	-4.80562300	-1.05336100	0.89361500

C	-5.89702900	-1.82204400	0.49903300
C	-5.75982900	-2.73987500	-0.54725200
C	-4.53391900	-2.88484300	-1.19473300
C	-3.43348400	-2.12138800	-0.79956500
C	-2.82777900	1.53093400	0.49785200
C	-3.58634700	1.78771800	-0.65599600
C	-4.07782700	3.06699100	-0.90596200
C	-3.81051400	4.11053500	-0.01656700
C	-3.05280200	3.86417300	1.12844100
C	-2.56678700	2.58202300	1.38713600
P	1.02410700	-0.68997200	1.38903300
C	2.70254700	-1.38540900	1.18950700
C	2.85204900	-2.76236800	0.95746600
C	4.12420400	-3.30017500	0.77284200
C	5.25295000	-2.47882300	0.81238700
C	5.10462100	-1.10888800	1.02581800
C	3.83522200	-0.56078000	1.20818400
C	1.29359900	0.94618300	2.18527000
C	0.70704800	2.09277500	1.64097600
C	0.84010400	3.32901400	2.27801400
C	1.57353600	3.43208700	3.45794200
C	2.17633500	2.29371200	4.00405500
C	2.03427600	1.05960800	3.37521600
H	-2.97444100	-0.34866000	3.00499600
H	-1.42224300	0.48987800	2.95868200
H	-1.63840700	-2.48607100	2.24929400
H	-1.50484000	-1.96228200	3.92709200
H	0.60873600	-2.70850200	2.62181900
H	0.73614600	-1.29631900	3.69119500
H	-4.92246200	-0.32251000	1.68946500
H	-6.85263500	-1.70456500	1.00222600
H	-6.61161200	-3.34051100	-0.85443500
H	-4.42133800	-3.60257000	-2.00146700
H	-2.47151500	-2.25646900	-1.27701500

H	-3.78784800	0.98340500	-1.35489300
H	-4.66979200	3.24941900	-1.79854000
H	-4.19505400	5.10744300	-0.21289000
H	-2.84408200	4.66762300	1.82943100
H	-1.98070300	2.41389800	2.28262800
H	1.97214400	-3.39733500	0.88549900
H	4.22930800	-4.36631300	0.59330100
H	6.24272500	-2.90380700	0.66928400
H	5.97552300	-0.45963100	1.04243600
H	3.73125500	0.50703900	1.35950400
H	0.14302400	2.01853200	0.71901100
H	0.37230300	4.20287200	1.83624700
H	1.68049500	4.39322100	3.95302000
H	2.75461400	2.36918700	4.92052000
H	2.51371200	0.18314900	3.80134400
Br	-0.53177800	-3.73062000	0.12754200

TS4'

B3LYP-D3(BJ) SCF energy = -2561.447258 a.u.

M06L SCF energy in solution = -2562.912229 a.u.

M06L free energy in solution = -2562.298887 a.u.

Imaginary Frequency = -213.33 cm^{-1}

C	1.86291300	3.26176700	1.69892300
C	0.94807200	4.27097900	2.02180600
C	-0.26634900	3.99169700	2.65133000
C	-0.61347500	2.67338700	2.94388800
C	0.28391600	1.65370300	2.63282500
C	1.52569400	1.93496500	2.03196600
C	-0.06501600	0.20566400	2.90051100
C	0.35769100	-0.34973800	4.24864300
O	-1.16007000	-0.25499400	2.38623400
C	3.12865300	3.61448600	0.95717600
H	1.19119900	5.29814700	1.76103200
H	-0.94377400	4.80296300	2.90288700

H	-1.56480200	2.43519100	3.41056200
H	-0.34126600	0.04632300	4.99812700
H	0.28636900	-1.43839800	4.25446100
H	1.37041900	-0.03726500	4.51217000
H	3.98657700	3.71885700	1.63359400
H	3.38077200	2.84906400	0.21742200
H	3.00953900	4.56504000	0.42917500
Pd	-0.23836100	-0.52437900	0.47262800
C	4.33512900	-0.60099000	2.02298000
C	3.57673200	-1.77031000	2.05052500
C	3.71832700	0.64730900	1.93869700
C	2.32517300	0.71513500	1.81825600
C	1.56768900	-0.47852200	1.74165500
C	2.18058400	-1.73231400	1.93610800
C	1.38321700	-3.00165900	2.02138100
H	5.41599300	-0.66029300	2.11502600
H	4.07133000	-2.73155300	2.16083800
H	4.31251800	1.54814000	2.03477000
H	1.92113700	-3.76064700	2.59949500
H	0.40557500	-2.82850700	2.48101700
H	1.16055500	-3.41164900	1.02968200
C	-2.25004100	-0.99815600	-2.47325600
C	-1.07138100	-0.58204400	-3.37106900
C	0.28563300	-1.16934300	-2.96080500
P	-2.21367400	-0.31196500	-0.76601000
C	-2.62131700	1.46518500	-0.93867400
C	-2.90546000	2.08487500	-2.16147400
C	-3.13178300	3.46188100	-2.21892200
C	-3.08568400	4.22920600	-1.05570700
C	-2.81989400	3.61377600	0.17107300
C	-2.58845300	2.24281600	0.22961100
C	-3.66724500	-1.00807000	0.08729500
C	-3.51746200	-2.14395000	0.89451100
C	-4.63110200	-2.68394400	1.53741400

C	-5.88796600	-2.09633300	1.38613800
C	-6.03745300	-0.96220100	0.58474100
C	-4.93029100	-0.41557300	-0.06184100
P	1.01749800	-0.42608200	-1.43569500
C	1.26854800	1.32163100	-1.92264900
C	0.62542200	2.33523100	-1.20328200
C	0.71380200	3.66506800	-1.61670500
C	1.45686300	3.99404400	-2.74889900
C	2.11119700	2.99007900	-3.47088800
C	2.01360500	1.66105500	-3.06528900
C	2.67165600	-1.19072900	-1.36085200
C	2.75742700	-2.59296800	-1.34502200
C	4.00597400	-3.20861600	-1.28760500
C	5.17210100	-2.44060000	-1.24431800
C	5.08656200	-1.04881500	-1.24812400
C	3.84078500	-0.42283800	-1.30056600
H	-3.20522800	-0.71569100	-2.93103000
H	-2.24441800	-2.08348100	-2.33015000
H	-0.99602000	0.50897500	-3.44484300
H	-1.29082400	-0.94319600	-4.38326400
H	1.01632900	-1.01587900	-3.76285900
H	0.19165100	-2.24157700	-2.76479700
H	-2.94959400	1.50529600	-3.07624700
H	-3.34261000	3.93194000	-3.17527900
H	-3.25829900	5.30077900	-1.10263500
H	-2.77914100	4.20361300	1.08122200
H	-2.35873300	1.76393000	1.17515500
H	-2.54419600	-2.61274300	0.98804300
H	-4.51158400	-3.56769400	2.15717700
H	-6.75125800	-2.51986400	1.89228900
H	-7.01399200	-0.50103000	0.46567300
H	-5.04675400	0.47378300	-0.67358400
H	0.05086500	2.08236200	-0.32225200
H	0.19778000	4.43081800	-1.04701500

H	1.52838500	5.02839700	-3.07370600
H	2.69334800	3.24383000	-4.35226800
H	2.52468100	0.88734300	-3.63079400
H	1.85009300	-3.19342400	-1.35098400
H	4.06425200	-4.29318500	-1.27477200
H	6.14334400	-2.92598100	-1.20218100
H	5.98855400	-0.44489400	-1.20229900
H	3.78243500	0.65936700	-1.29102400
Br	-0.73975300	-3.74121400	-0.75491500

NBE*

B3LYP-D3(BJ) SCF energy = -500.641955 a.u.

M06L SCF energy in solution = -500.690513 a.u.

M06L free energy in solution = -500.5439149 a.u.

C	-1.77937400	1.10139100	0.86346200
C	-1.06057800	0.92441300	-0.51387500
C	0.06115800	-0.07385500	-0.27300000
C	-0.50248500	-1.28080800	-0.07338900
C	-2.42695100	-0.29707600	1.09947600
C	-2.00312600	-1.10191500	-0.18071700
C	-2.07353400	0.01758800	-1.24867700
C	1.46525300	0.32055200	-0.11051200
O	1.86108700	1.47177800	-0.12950000
O	2.28663700	-0.74518700	0.06356200
C	3.67164600	-0.41765700	0.23312300
H	-2.54143700	1.88472300	0.79563400
H	-1.07612500	1.38744800	1.64980600
H	-0.75907600	1.85546100	-0.99332800
H	0.00644900	-2.18763400	0.23314500
H	-2.07986000	-0.77973100	2.01721000
H	-3.51878900	-0.22939200	1.15111100
H	-2.58599700	-2.00829700	-0.35579900
H	-1.72080300	-0.30886200	-2.23158000

H	-3.07055900	0.46301400	-1.34221400
H	4.18601700	-1.37183500	0.35201000
H	4.05142500	0.11745400	-0.64158300
H	3.81490100	0.20912100	1.11774700

DPPP

B3LYP-D3(BJ) SCF energy = -1727.353751 a.u.

M06L SCF energy in solution = -1727.383878 a.u.

M06L free energy in solution = -1727.010632 a.u.

C	1.43542200	-1.81211500	0.56388500
C	0.13391400	-2.03544800	1.34953100
C	-0.84526600	-0.85308400	1.30941200
P	2.52635900	-0.46181200	1.28999500
C	4.02748200	-0.68564600	0.23312900
C	4.59093400	0.35017200	-0.52629800
C	5.77072400	0.14836900	-1.24509600
C	6.41060000	-1.09046000	-1.21847500
C	5.86843100	-2.12608600	-0.45425100
C	4.69601400	-1.92152300	0.27050300
C	1.77691100	1.06885800	0.57441400
C	1.42526800	1.20219400	-0.77835800
C	0.80234500	2.36079400	-1.23947900
C	0.51962000	3.40545000	-0.35723900
C	0.87363900	3.29133700	0.98621500
C	1.50093200	2.13162500	1.44674500
P	-1.50343900	-0.54708500	-0.42295500
C	-2.24876300	1.12940800	-0.25460300
C	-2.61208900	1.78569100	-1.44185400
C	-3.12980000	3.07899700	-1.41808200
C	-3.28202600	3.74799700	-0.20103600
C	-2.92494300	3.10880700	0.98606400
C	-2.41654400	1.80865900	0.95977600
C	-2.99376200	-1.64019600	-0.35441400
C	-2.87516200	-2.92974900	-0.89403200
C	-3.93872600	-3.83181900	-0.83034900

C	-5.14428500	-3.44762200	-0.24243900
C	-5.27905400	-2.16068700	0.28436800
C	-4.21142100	-1.26504000	0.23252200
H	2.03443500	-2.72885100	0.59623400
H	1.22094500	-1.60932200	-0.49075100
H	0.38007400	-2.25093700	2.39736800
H	-0.36607100	-2.93293100	0.95977500
H	-0.34098000	0.05925700	1.63673000
H	-1.68161900	-1.03380500	1.99386700
H	4.10348500	1.31836700	-0.56350500
H	6.18712600	0.96392400	-1.83050900
H	7.32616100	-1.24728300	-1.78176200
H	6.36234300	-3.09342000	-0.41773700
H	4.30154900	-2.73344700	0.87667200
H	1.63622100	0.39601900	-1.47345800
H	0.51731800	2.44049500	-2.28430400
H	0.00565300	4.29286900	-0.71312000
H	0.65245300	4.09886100	1.67841100
H	1.76715000	2.03838000	2.49647600
H	-2.47315500	1.27780500	-2.39322200
H	-3.40552700	3.56909300	-2.34795900
H	-3.67617700	4.76026700	-0.17951300
H	-3.04106500	3.62179200	1.93706900
H	-2.14361000	1.33119600	1.89444700
H	-1.94455700	-3.22404500	-1.37333600
H	-3.82892700	-4.82870800	-1.24873900
H	-5.97717400	-4.14404000	-0.20017000
H	-6.21834600	-1.85483800	0.73772700
H	-4.32387200	-0.26654500	0.64436300

1a

B3LYP-D3(BJ) SCF energy = -437.998726 a.u.

M06L SCF energy in solution = -438.241301 a.u.

M06L free energy in solution = -438.1152762 a.u.

C	-1.45252500	1.21732100	0.03369200
C	-1.04814400	2.55775900	0.09750100
C	0.29848300	2.90679000	0.07553700
C	1.27409500	1.91809200	-0.01548400
C	0.93198500	0.56128000	-0.08654300
C	-0.43439700	0.26272200	-0.05313000
Br	-0.99434600	-1.65354400	-0.11745400
C	1.99743800	-0.53268500	-0.16093500
C	3.38499300	-0.04339500	-0.55101800
O	2.14825200	-1.18318400	1.10132300
C	-2.91608800	0.86276700	0.06004800
H	-1.81104600	3.32836500	0.16524100
H	0.58943800	3.95174200	0.12824600
H	2.32026800	2.20056900	-0.03522300
H	1.66624900	-1.25849300	-0.91691100
H	4.04916800	-0.90677400	-0.64404900
H	3.36453800	0.49140600	-1.50561200
H	3.79916100	0.61347700	0.22017700
H	1.31578900	-1.64632400	1.28945200
H	-3.52732100	1.76761600	0.11739400
H	-3.20800000	0.30541500	-0.83672300
H	-3.15717400	0.22860600	0.92032900

1a'

B3LYP-D3(BJ) SCF energy = -436.796201 a.u.

M06L SCF energy in solution = -437.037662 a.u.

M06L free energy in solution = -436.9352143 a.u.

C	-1.65192300	0.82199800	0.07898200
C	-1.62656600	2.22241300	0.11328500
C	-0.42938600	2.93251400	0.06097600
C	0.77405100	2.24503500	-0.01195000
C	0.80879400	0.84008700	-0.00887800
C	-0.41576000	0.16306100	0.02272600

Br	-0.47391800	-1.81200600	-0.13806000
C	2.19770800	0.24399000	-0.03249600
C	2.53363400	-0.98744800	0.78796200
O	3.07401000	0.82722500	-0.64802100
C	-2.97046700	0.09312400	0.09324400
H	-2.57178900	2.75525800	0.16929800
H	-0.43831500	4.01810000	0.07354900
H	1.72118600	2.76988100	-0.06772400
H	1.85333400	-1.13537600	1.62956400
H	2.48818100	-1.88195700	0.15894500
H	3.56156700	-0.87376600	1.14222000
H	-3.79769500	0.80331100	0.17603900
H	-3.11070400	-0.49372800	-0.82122900
H	-3.03123600	-0.60794200	0.93251800

(*S*)-**3aa**

B3LYP-D3(BJ) SCF energy = -694.636535 a.u.

M06L SCF energy in solution = -694.682011 a.u.

M06L free energy in solution = -694.464618 a.u.

C	2.37865000	1.02184900	0.00113500
C	3.49849100	0.17798500	-0.04600500
C	3.37931600	-1.21188700	-0.08370900
C	2.11442400	-1.80826300	-0.06911400
C	0.99702200	-0.98887300	-0.02948600
C	1.11274900	0.41298400	0.00453400
C	-0.46776000	-1.41404000	0.01040200
C	-0.76157100	-2.22129800	1.28491700
C	2.56216700	2.51764100	0.04104100
H	4.48844300	0.62744400	-0.05287900
H	4.27284000	-1.82852500	-0.12040200
H	2.01847000	-2.89185500	-0.08824400
H	-1.81736700	-2.49928000	1.33038500
H	-0.16075400	-3.13976900	1.29745500

H	-0.50714600	-1.64302500	2.17856700
H	3.62442100	2.77992700	0.03673600
H	2.09357500	3.00363600	-0.82341500
H	2.11266600	2.95551800	0.94060300
C	-2.06125700	2.56209100	0.01770900
C	-2.97756700	1.51364000	-0.03869700
C	-0.68701200	2.31122000	0.04769200
C	-0.24883300	0.98564100	0.02448100
C	-1.18723700	-0.06936900	-0.00905100
C	-2.56005300	0.17142400	-0.05059100
C	-3.58105700	-0.94042700	-0.10679200
H	-2.41942700	3.58793900	0.02770100
H	-4.04189200	1.73226700	-0.07937700
H	0.01098200	3.13801500	0.07870800
H	-4.50665100	-0.59178600	-0.57643300
H	-3.19986900	-1.79332100	-0.67220200
H	-3.84212100	-1.29205700	0.90031100
O	-0.85587500	-2.16039400	-1.15261200
H	-0.34200500	-2.98388900	-1.14741400

K_2CO_3

B3LYP-D3(BJ) SCF energy = -1463.700780 a.u.

M06L SCF energy in solution = -1463.845680 a.u.

M06L free energy in solution = -1463.874166 a.u.

C	-0.00007000	0.80442900	0.00003700
O	1.12762200	1.41511500	-0.00019100
O	0.00022600	-0.54772900	-0.00002000
O	-1.12755900	1.41512100	0.00017300
K	2.47580100	-0.60754800	0.00003700
K	-2.47590000	-0.60753800	-0.00003300

$KHCO_3$

B3LYP-D3(BJ) SCF energy = -864.363744 a.u.

M06L SCF energy in solution = -864.472937 a.u.

M06L free energy in solution = -864.4863777 a.u.

C	-0.99173200	0.04280400	-0.00017900
O	-0.40428900	1.15282900	-0.00023400
O	-0.46043600	-1.11076600	-0.00021400
K	1.81265200	-0.02191400	0.00010600
O	-2.36715300	0.08413100	0.00034900
H	-2.63497700	-0.85001000	-0.00013600

KOAc

B3LYP-D3(BJ) SCF energy = -828.446850 a.u.

M06L SCF energy in solution = -828.531397 a.u.

M06L free energy in solution = -828.5245061 a.u.

C	-2.49446000	-0.00228000	0.00021400
H	-2.89948400	1.01205400	-0.00454400
H	-2.85643300	-0.55015000	-0.87714700
H	-2.85569000	-0.54093700	0.88360800
C	-0.96351200	0.00998900	-0.00032600
O	-0.38231200	-1.11989800	-0.00023400
O	-0.37660700	1.13497800	-0.00022600
K	1.86477800	-0.00462400	0.00012800

KBr

B3LYP-D3(BJ) SCF energy = -613.149749 a.u.

M06L SCF energy in solution = -613.398759 a.u.

M06L free energy in solution = -613.4327063 a.u.

K	0.00000000	0.00000000	-1.89011100
Br	0.00000000	0.00000000	1.02606000

8. References

[1] A. Casnati, M. Fontana, E. Motti, N. Della Ca'. Synthesis of fluorenyl alcohols via cooperative palladium/norbornene catalysis. *Org. Biomol. Chem.* **2019**, *17*, 6165–6173.

[2] B. Schulte, R. Fröhlich, A. Studer. Atroposelective radical aryl migration reactions from sulfur to carbon. *Tetrahedron* **2008**, *64*, 11852–11859.

[3] R. Déziel, E. Malenfant, G. Bélanger. Practical synthesis of (*R,R*)- and (*S,S*)-bis[2,6-bis(1-ethoxyethyl)phenyl] diselenide. *J. Org. Chem.* **1996**, *61*, 1875–1876.

[4] R. J. Perkins, R. Feng, Q. Lu, K. D. Moeller. Anodic cyclizations, seven-membered rings, and the choice of radical cation vs. radical pathways. *Chin. J. Chem.* **2019**, *37*, 672–678.

[5] C. Zhang, F. Li, Y. Yu, A. Huang, P. He, M. Lei, J. Wang, L. Huang, Z. Liu, J. Liu, Y. Wei. Design, synthesis, and evaluation of a series of novel benzocyclobutene derivatives as general anesthetics. *J. Med. Chem.* **2017**, *60*, 3618–3625.

[6] Z.-S. Liu, Y. Hua, Q. Gao, Y. Ma, H. Tang, Y. Shang, H.-G. Cheng, Q. Zhou. Construction of axial chirality via palladium/chiral norbornene cooperative catalysis. *Nat. Catal.* **2020**, *3*, 727–733.

[7] M. J. Frisch, G. W. Trucks, H. B. Schlegel, *et al. Gaussian 16*, Revision C.01; Gaussian, Inc.: Wallingford, CT, **2016**.

[8] (a) M. Head-Gordon, J. A. Pople, M. Frisch, *Chem. Phys. Lett.* **1988**, *153*, 503-506; (b) C. Lee, W. Yang, R. G. Parr, *Phys. Rev. B: Condens. Matter Mater. Phys.* **1988**, *37*, 785–789; (c) S. Grimme, J. Antony, S. Ehrlich, H. Krieg, *J. Chem. Phys.* **2010**, *132*, 154104; (d) P. J. Hay, W. R. Wadt, *J. Chem. Phys.* **1985**, *82*, 299–310.

[9] T. Lu, Q. Chen, *Comput. Theor. Chem.* **2021**, *1200*, 113249–113256.

[10] S. Grimme, *Chem. Eur. J.* **2012**, *18*, 9955–9964.

[11] A. V. Marenich, C. J. Cramer, D. G. Truhlar, *J. Phys. Chem. B.* **2009**, *113*, 6378–6396.

[12] Y. Zhao, D. G. Truhlar, *J. Chem. Phys.* **2006**, *125*, 194101.

[13] (a) U. Häussermann, M. Dolg, H. Stoll, H.Preuss, P. Schwerdtfeger, R. M. Pitzer, *Mol. Phys.* **1993**, *78*, 1211–1224; (b) W. Küchle, M. Dolg, H. Stoll, H. Preuss, *J. Chem. Phys.* **1994**, *100*, 7535–7542.

[14] CYLview 20, C. Y. Legault, Université de Sherbrooke, **2020** (http://www.cylview.org).

[15] (a) C. Bannwarth, S. Ehlert, S. Grimme, *J. Chem. Theory Comput.* **2019**, *15*, 1652–1671; (b) P. Pracht, F. Bohle, S. Grimme, *Phys. Chem. Chem. Phys.* **2020**, *22*, 7169–7192.

9. HPLC traces of the products

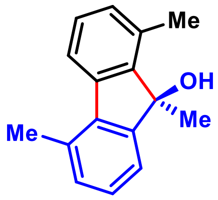

3aa

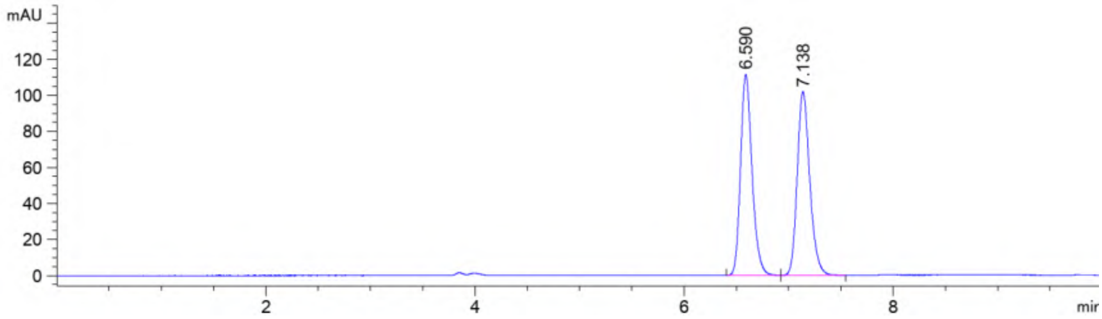

Signal 1: DAD1 F, Sig=290,4 Ref=360,100

Peak #	RetTime [min]	Type	Width [min]	Area [mAU*s]	Height [mAU]	Area %
1	6.590	BB	0.1171	849.89893	111.32468	50.0695
2	7.138	BB	0.1255	847.54034	101.90073	49.9305

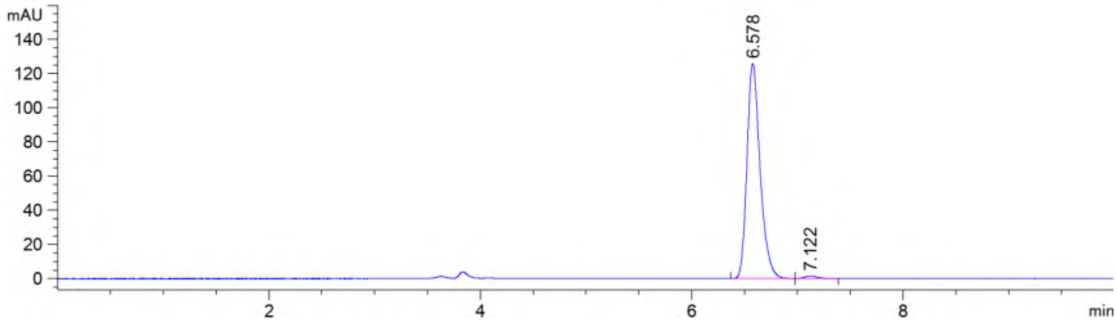

Signal 1: DAD1 F, Sig=290,4 Ref=360,100

Peak #	RetTime [min]	Type	Width [min]	Area [mAU*s]	Height [mAU]	Area %
1	6.578	BB	0.1306	1085.65503	125.91789	98.7756
2	7.122	BB	0.1086	13.45780	1.46652	1.2244

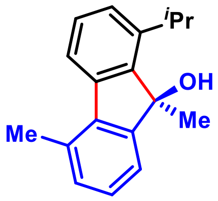

3ab

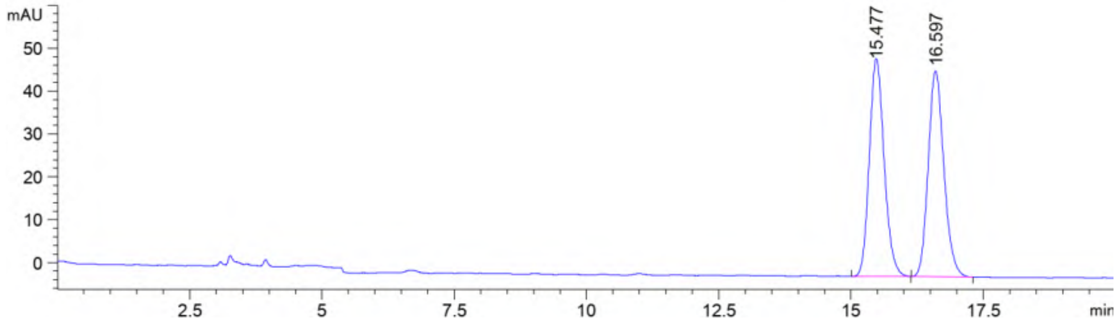

Signal 1: DAD1 D, Sig=230,4 Ref=360,100

Peak #	RetTime [min]	Type	Width [min]	Area [mAU*s]	Height [mAU]	Area %
1	15.477	BB	0.3015	991.17853	50.83115	50.0591
2	16.597	BB	0.3165	988.83630	47.97024	49.9409

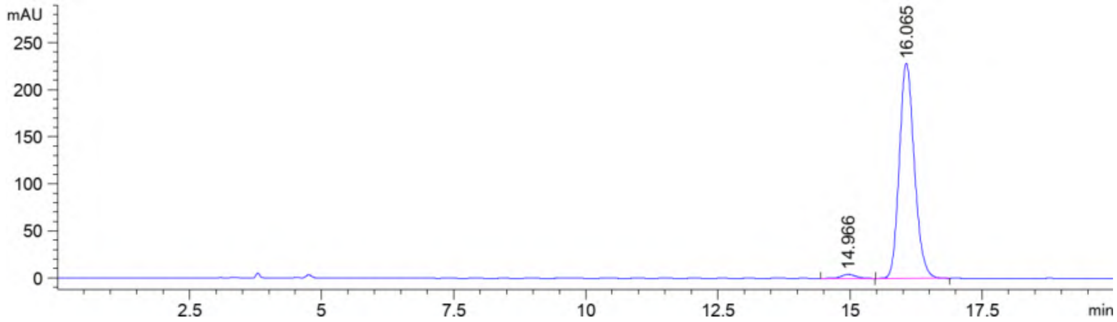

Signal 1: DAD1 D, Sig=230,4 Ref=360,100

Peak #	RetTime [min]	Type	Width [min]	Area [mAU*s]	Height [mAU]	Area %
1	14.966	BB	0.2885	81.23684	4.22193	1.7654
2	16.065	BB	0.3047	4520.49561	228.61519	98.2346

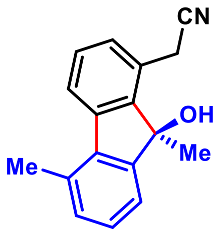

3ac

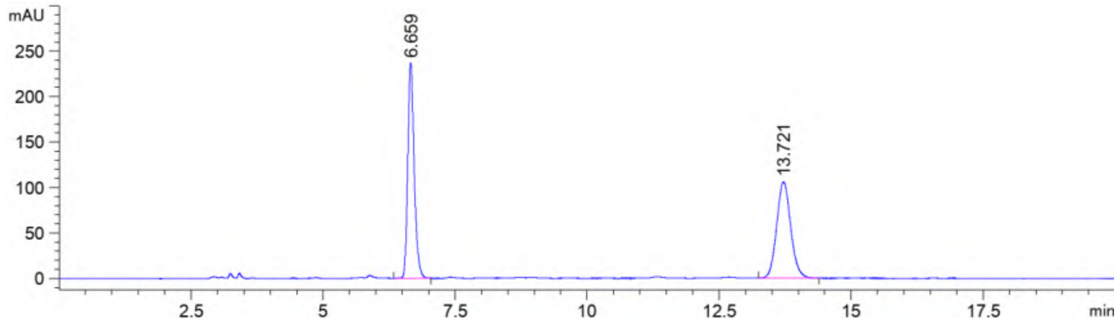

Signal 1: DAD1 D, Sig=230,4 Ref=360,100

Peak #	RetTime [min]	Type	Width [min]	Area [mAU*s]	Height [mAU]	Area %
1	6.659	VV R	0.1290	2003.91833	237.23634	50.6211
2	13.721	VV R	0.2416	1954.74036	106.00918	49.3789

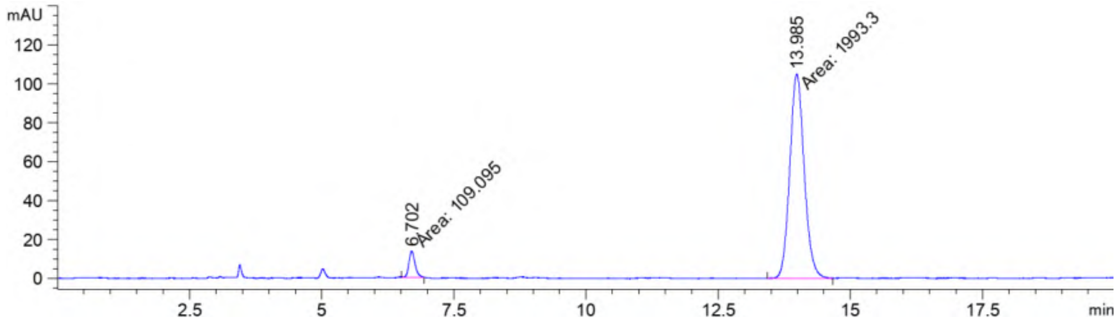

Signal 1: DAD1 D, Sig=230,4 Ref=360,100

Peak #	RetTime [min]	Type	Width [min]	Area [mAU*s]	Height [mAU]	Area %
1	6.702	MM	0.1338	109.09492	13.58861	5.1891
2	13.985	MM	0.3167	1993.29553	104.89879	94.8109

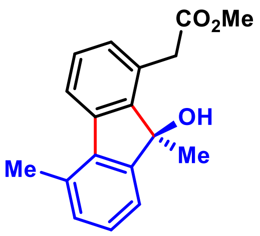

3ad

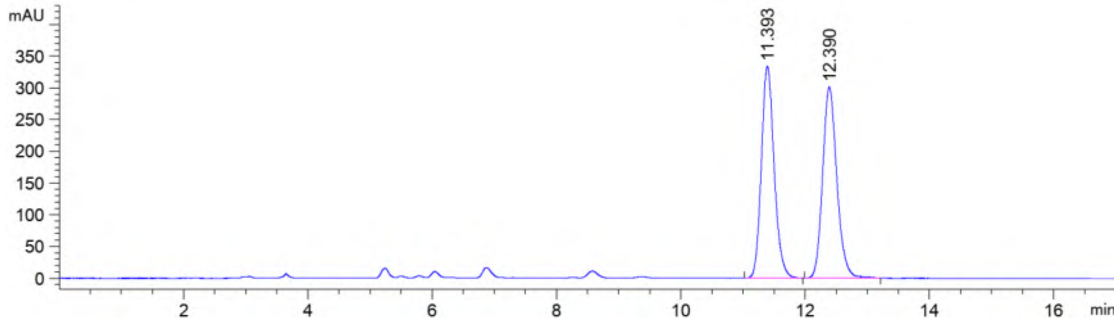

Signal 1: DAD1 D, Sig=230,4 Ref=360,100

Peak #	RetTime [min]	Type	Width [min]	Area [mAU*s]	Height [mAU]	Area %
1	11.393	BV R	0.2062	4769.78906	333.84943	49.9311
2	12.390	BV R	0.2328	4782.95459	301.12408	50.0689

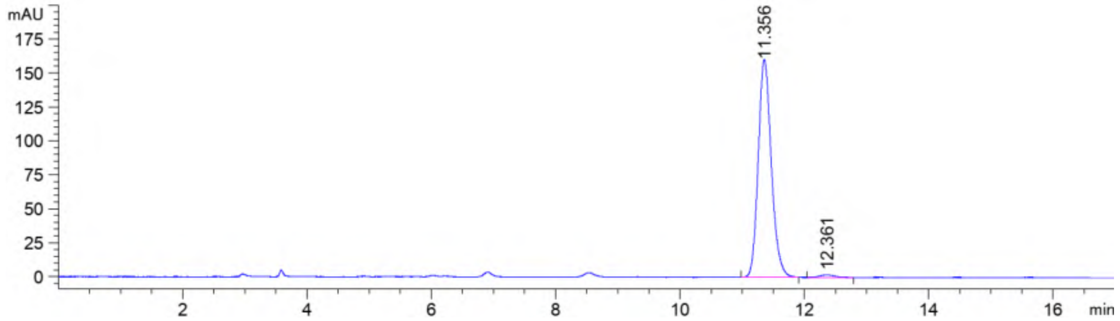

Signal 1: DAD1 D, Sig=230,4 Ref=360,100

Peak #	RetTime [min]	Type	Width [min]	Area [mAU*s]	Height [mAU]	Area %
1	11.356	VV R	0.2075	2343.51978	160.36266	98.6740
2	12.361	VV R	0.1971	31.49369	1.90307	1.3260

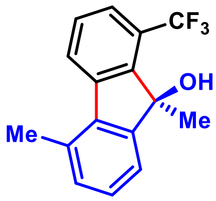

3ae

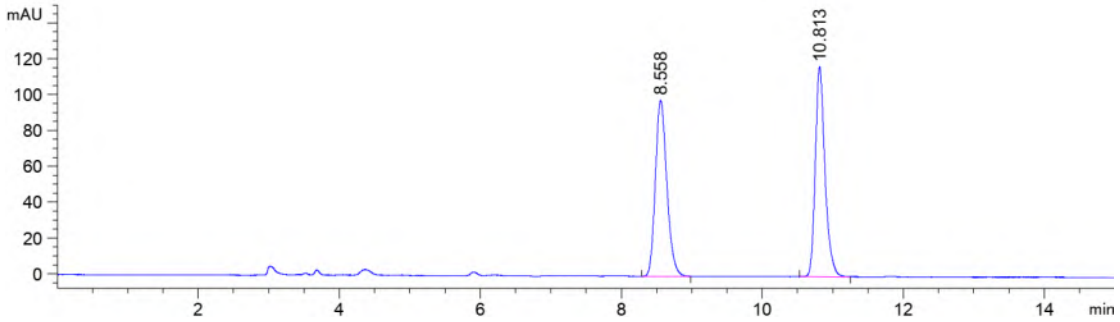

Signal 1: DAD1 D, Sig=230,4 Ref=360,100

Peak #	RetTime [min]	Type	Width [min]	Area [mAU*s]	Height [mAU]	Area %
1	8.558	BV R	0.1677	1111.17065	98.09077	49.9137
2	10.813	VV R	0.1427	1115.01147	116.97891	50.0863

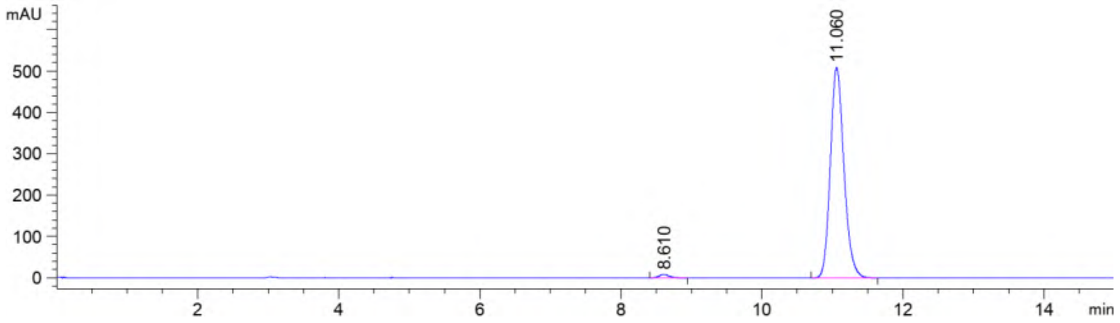

Signal 1: DAD1 D, Sig=230,4 Ref=360,100

Peak #	RetTime [min]	Type	Width [min]	Area [mAU*s]	Height [mAU]	Area %
1	8.610	VV R	0.1230	84.48999	8.21551	1.2219
2	11.060	BV R	0.2005	6830.12158	509.11465	98.7781

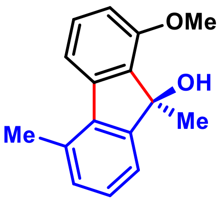

3af

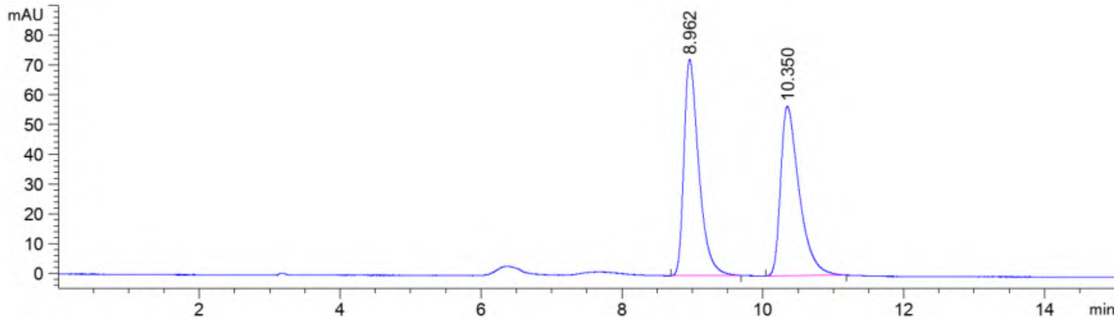

Signal 1: DAD1 A, Sig=254,4 Ref=360,100

Peak #	RetTime [min]	Type	Width [min]	Area [mAU*s]	Height [mAU]	Area %
1	8.962	BB	0.2072	1061.92029	72.56107	50.4437
2	10.350	BB	0.2444	1043.23853	56.90609	49.5563

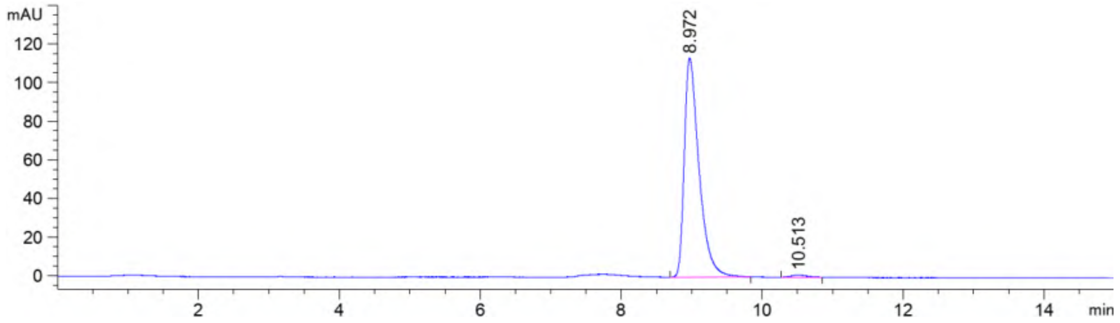

Signal 1: DAD1 A, Sig=254,4 Ref=360,100

Peak #	RetTime [min]	Type	Width [min]	Area [mAU*s]	Height [mAU]	Area %
1	8.972	BB	0.2090	1646.99377	113.34753	98.9651
2	10.513	BB	0.1925	17.22253	1.05745	1.0349

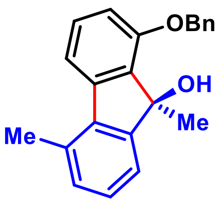

3ag

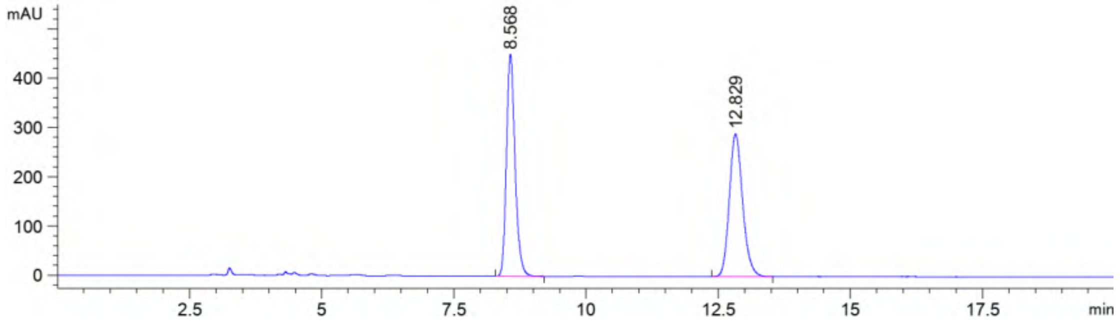

Signal 1: DAD1 D, Sig=230,4 Ref=360,100

Peak #	RetTime [min]	Type	Width [min]	Area [mAU*s]	Height [mAU]	Area %
1	8.568	BB	0.1721	5081.13770	450.49026	50.0726
2	12.829	BB	0.2664	5066.40820	289.23068	49.9274

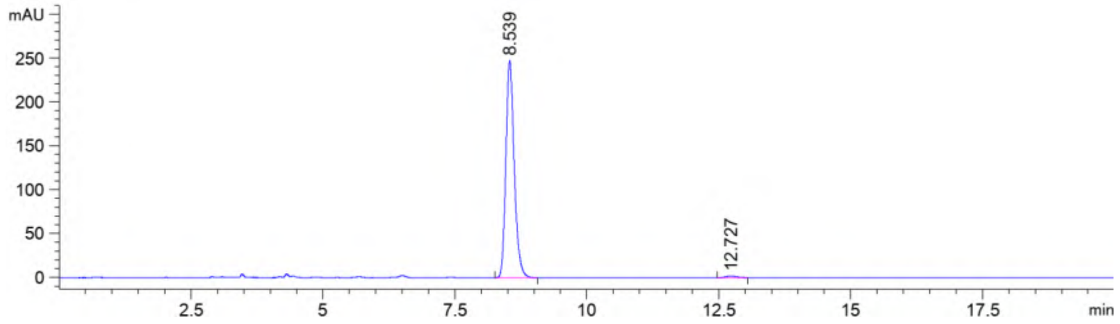

Signal 1: DAD1 D, Sig=230,4 Ref=360,100

Peak #	RetTime [min]	Type	Width [min]	Area [mAU*s]	Height [mAU]	Area %
1	8.539	BB	0.1681	2725.26929	247.26173	98.9957
2	12.727	BB	0.1787	27.64846	1.83071	1.0043

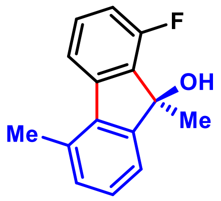

3ah

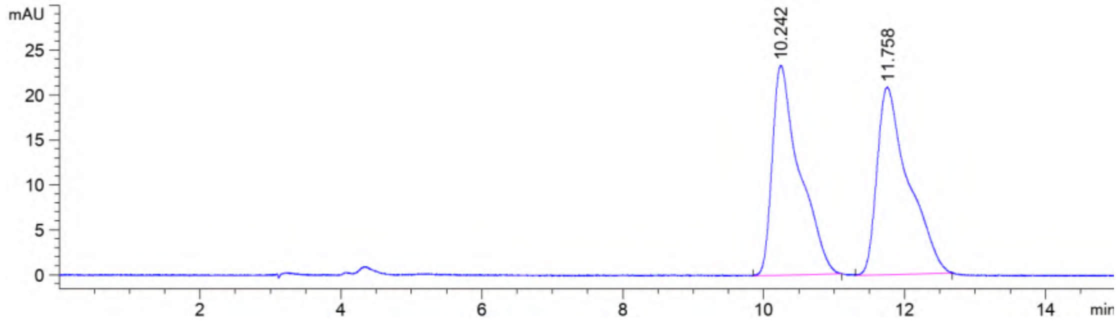

Signal 1: DAD1 F, Sig=290,4 Ref=360,100

Peak #	RetTime [min]	Type	Width [min]	Area [mAU*s]	Height [mAU]	Area %
1	10.242	BV R	0.3157	629.15021	23.36352	49.3294
2	11.758	VB R	0.3642	646.25513	20.84261	50.6706

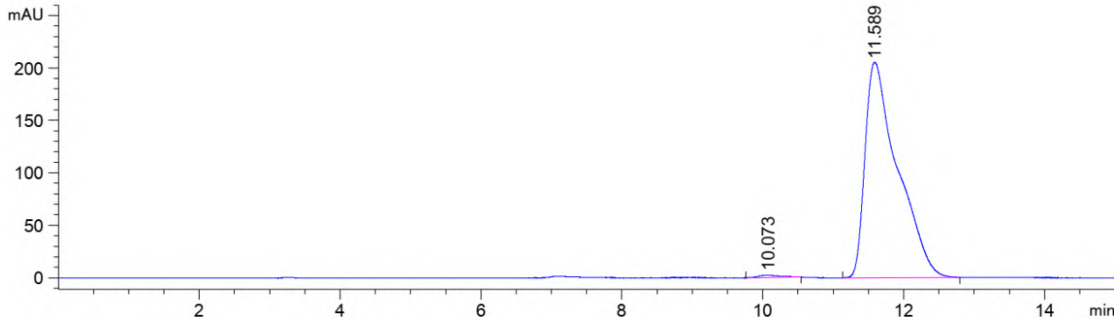

Signal 1: DAD1 F, Sig=290,4 Ref=360,100

Peak #	RetTime [min]	Type	Width [min]	Area [mAU*s]	Height [mAU]	Area %
1	10.073	BB	0.2505	46.92313	2.20321	0.7071
2	11.589	BB	0.4229	6589.15625	205.31233	99.2929

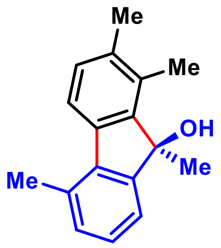

3ai

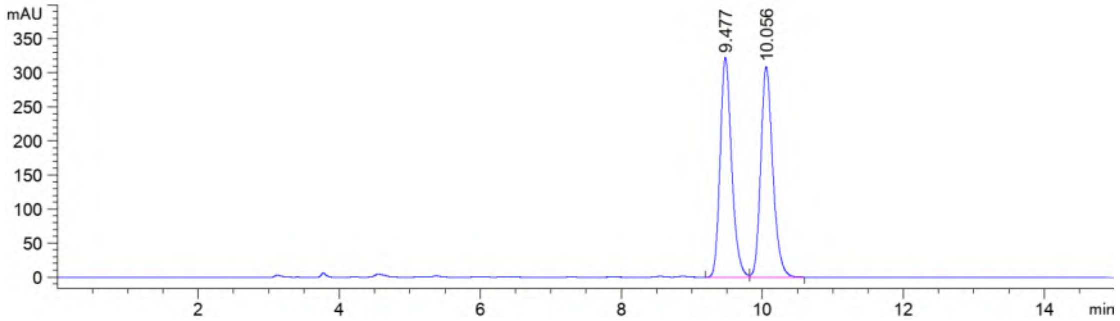

Signal 1: DAD1 D, Sig=230,4 Ref=360,100

Peak #	RetTime [min]	Type	Width [min]	Area [mAU*s]	Height [mAU]	Area %
1	9.477	BV	0.1723	3654.73657	322.39844	49.9382
2	10.056	VB	0.1794	3663.78662	308.76761	50.0618

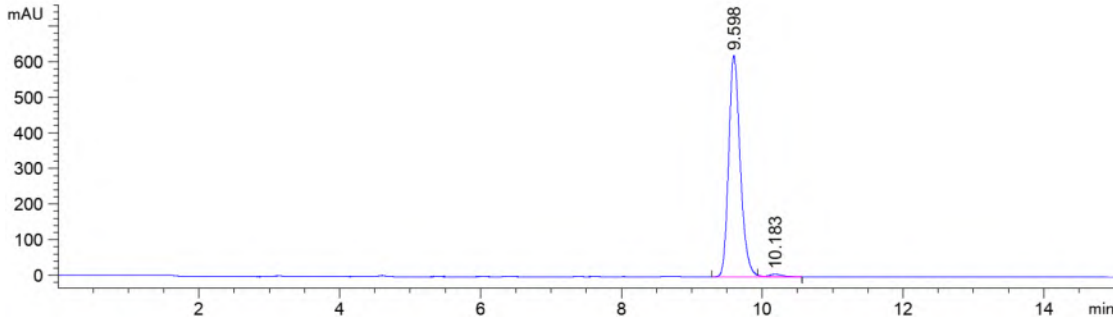

Signal 1: DAD1 D, Sig=230,4 Ref=360,100

Peak #	RetTime [min]	Type	Width [min]	Area [mAU*s]	Height [mAU]	Area %
1	9.598	BV R	0.1723	7072.60303	621.51422	98.8072
2	10.183	VB E	0.1426	85.37730	7.21042	1.1928

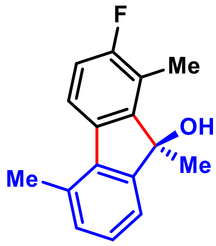

3aj

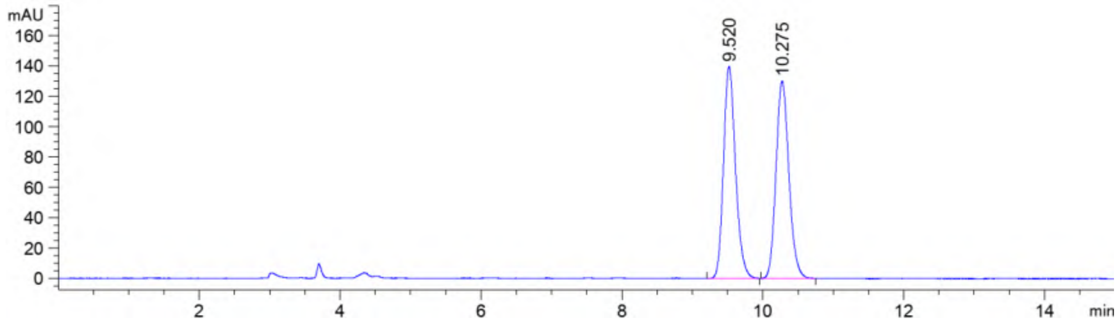

Signal 1: DAD1 D, Sig=230,4 Ref=360,100

Peak #	RetTime [min]	Type	Width [min]	Area [mAU*s]	Height [mAU]	Area %
1	9.520	VB R	0.1772	1655.54529	139.79631	50.0233
2	10.275	BV R	0.1902	1654.00586	130.20059	49.9767

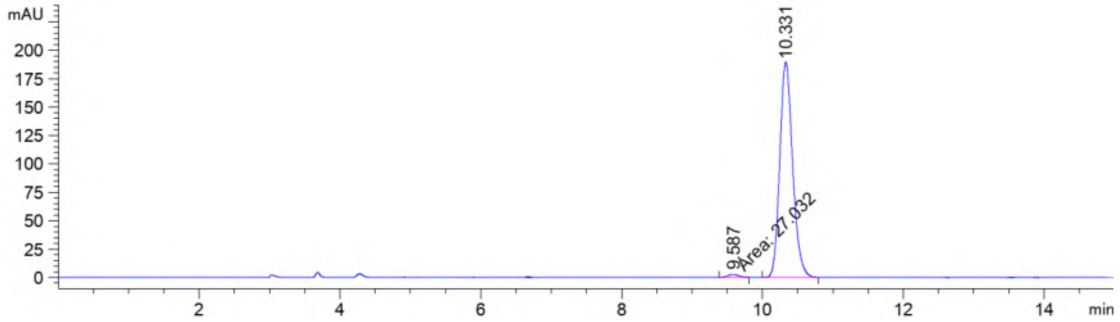

Signal 1: DAD1 D, Sig=230,4 Ref=360,100

Peak #	RetTime [min]	Type	Width [min]	Area [mAU*s]	Height [mAU]	Area %
1	9.587	MM	0.1796	27.03201	2.50867	1.1132
2	10.331	BV R	0.1792	2401.18799	189.64044	98.8868

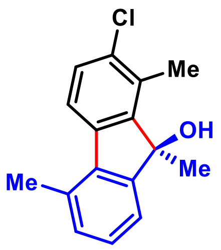

3ak

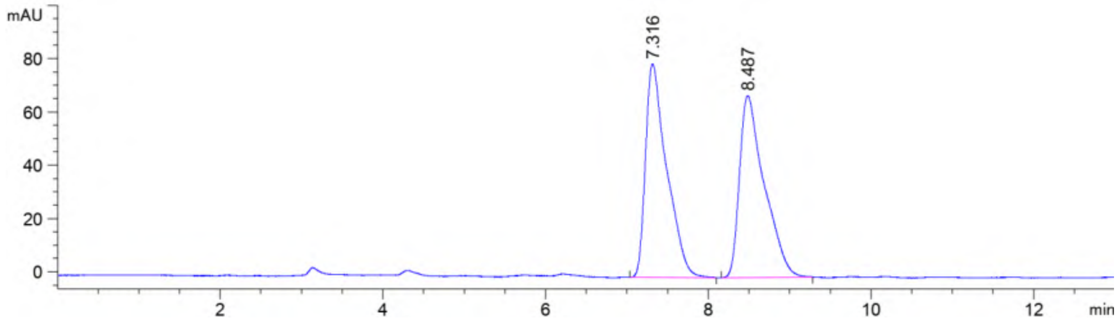

Signal 1: DAD1 C, Sig=220,4 Ref=360,100

Peak #	RetTime [min]	Type	Width [min]	Area [mAU*s]	Height [mAU]	Area %
1	7.316	BB	0.2389	1449.38989	80.17080	50.1570
2	8.487	BV R	0.2765	1440.31787	68.16839	49.8430

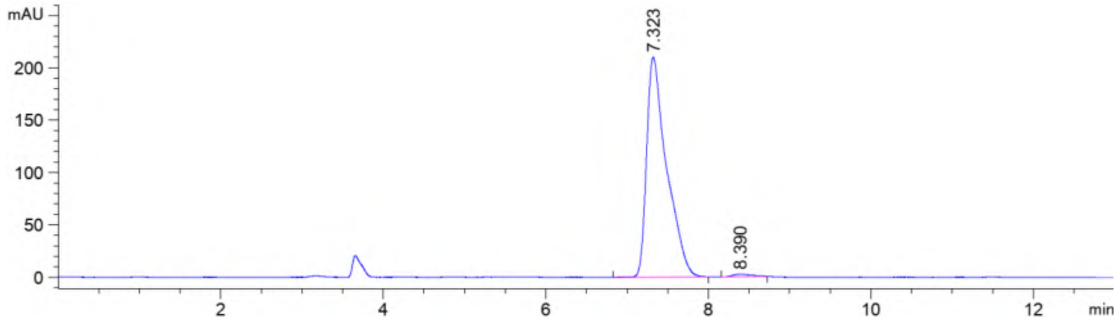

Signal 1: DAD1 C, Sig=220,4 Ref=360,100

Peak #	RetTime [min]	Type	Width [min]	Area [mAU*s]	Height [mAU]	Area %
1	7.323	VB R	0.2387	3593.68066	209.98779	98.9340
2	8.390	BB	0.1772	38.72121	2.57091	1.0660

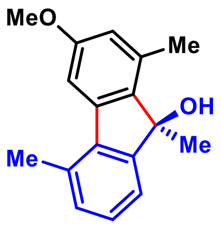

3al

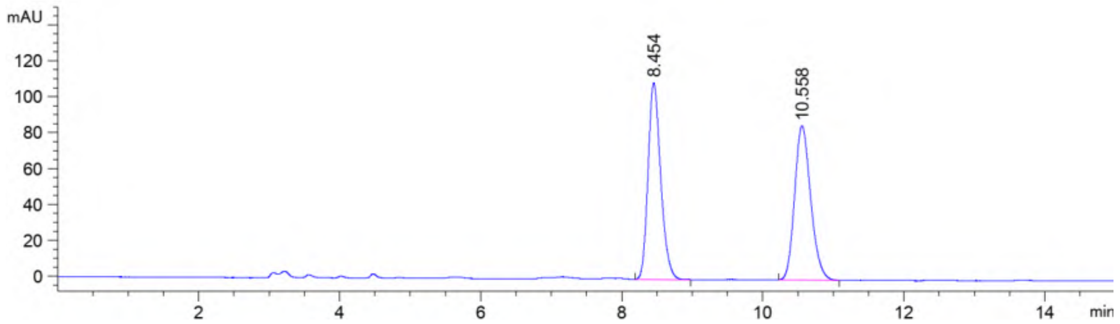

Signal 1: DAD1 D, Sig=230,4 Ref=360,100

Peak #	RetTime [min]	Type	Width [min]	Area [mAU*s]	Height [mAU]	Area %
1	8.454	BB	0.1872	1363.11633	109.51041	50.0851
2	10.558	BB	0.2386	1358.48511	85.84546	49.9149

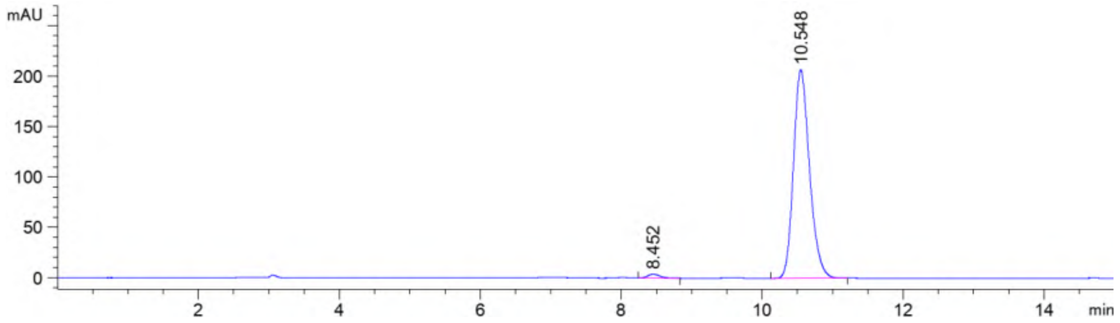

Signal 1: DAD1 D, Sig=230,4 Ref=360,100

Peak #	RetTime [min]	Type	Width [min]	Area [mAU*s]	Height [mAU]	Area %
1	8.452	BB	0.1482	48.34602	3.89690	1.4593
2	10.548	BB	0.2383	3264.56299	206.69452	98.5407

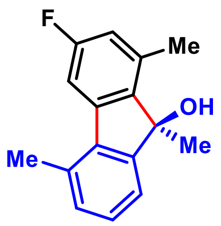

3am

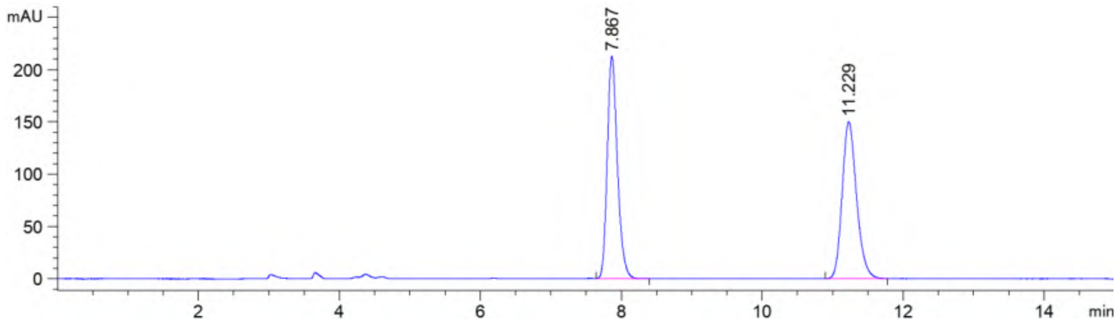

Signal 1: DAD1 D, Sig=230,4 Ref=360,100

Peak #	RetTime [min]	Type	Width [min]	Area [mAU*s]	Height [mAU]	Area %
1	7.867	BV R	0.1494	2120.42285	212.54572	49.9086
2	11.229	BV R	0.2090	2128.18823	150.00612	50.0914

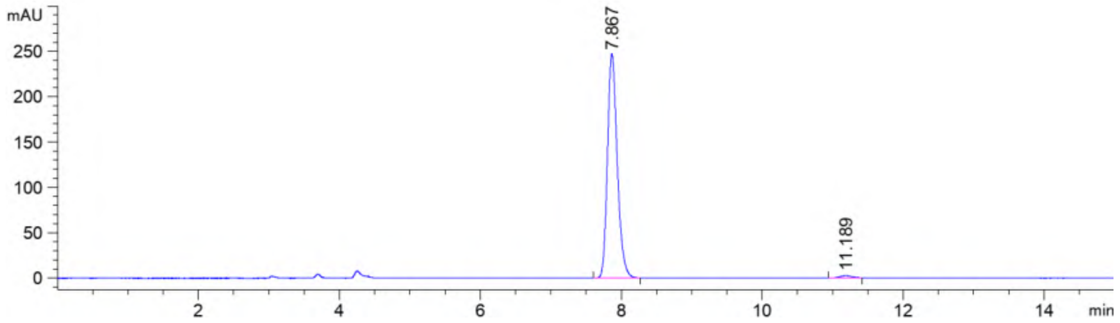

Signal 1: DAD1 D, Sig=230,4 Ref=360,100

Peak #	RetTime [min]	Type	Width [min]	Area [mAU*s]	Height [mAU]	Area %
1	7.867	VV R	0.1463	2381.33423	247.37079	98.7993
2	11.189	BV R	0.1500	28.93966	2.31178	1.2007

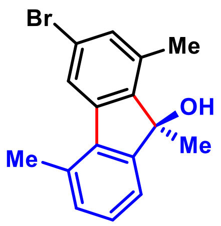

3an

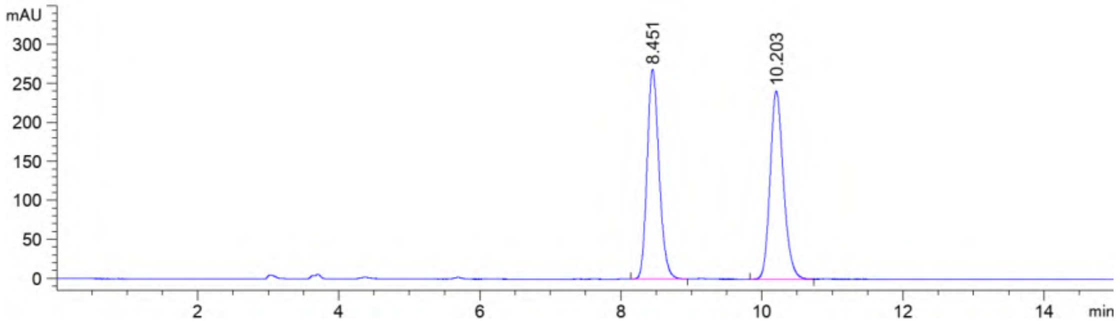

Signal 1: DAD1 D, Sig=230,4 Ref=360,100

Peak #	RetTime [min]	Type	Width [min]	Area [mAU*s]	Height [mAU]	Area %
1	8.451	BV R	0.1775	3169.59814	269.03186	49.8910
2	10.203	VV R	0.1972	3183.45410	241.57362	50.1090

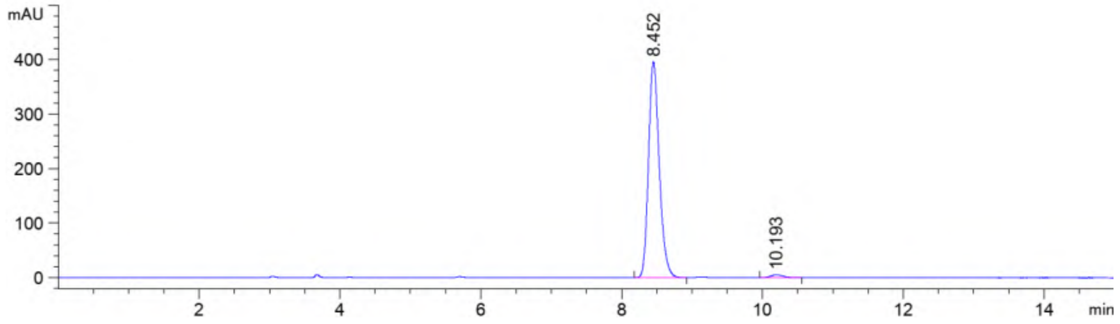

Signal 1: DAD1 D, Sig=230,4 Ref=360,100

Peak #	RetTime [min]	Type	Width [min]	Area [mAU*s]	Height [mAU]	Area %
1	8.452	VV R	0.1629	4204.72021	396.02402	98.4038
2	10.193	VV R	0.1451	68.20345	5.53935	1.5962

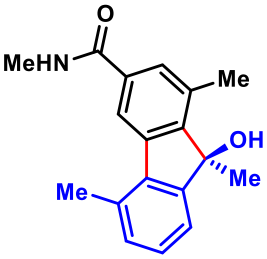

3ao

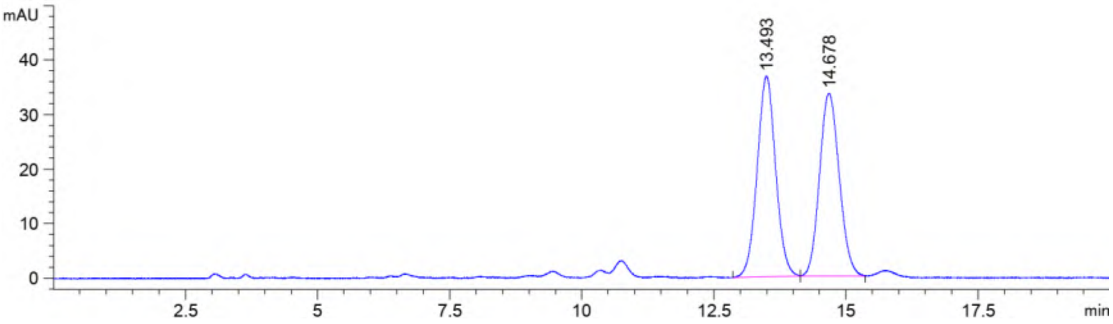

Signal 1: DAD1 A, Sig=254,4 Ref=360,100

Peak #	RetTime [min]	Type	Width [min]	Area [mAU*s]	Height [mAU]	Area %
1	13.493	VV R	0.2861	896.63757	36.82526	50.7931
2	14.678	VV R	0.3046	868.63513	33.48488	49.2069

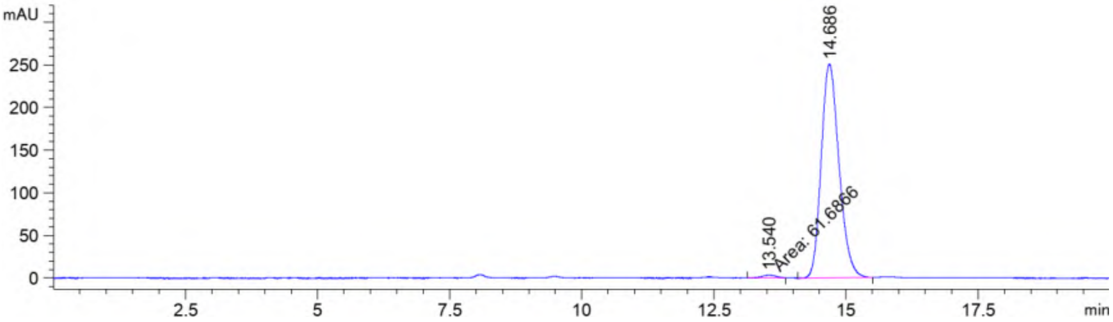

Signal 1: DAD1 A, Sig=254,4 Ref=360,100

Peak #	RetTime [min]	Type	Width [min]	Area [mAU*s]	Height [mAU]	Area %
1	13.540	MP	0.3272	61.68657	3.14180	1.0384
2	14.686	VB R	0.2846	5878.90430	250.69986	98.9616

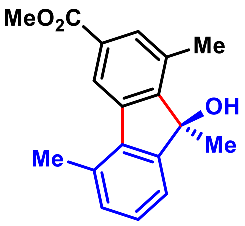

3ap

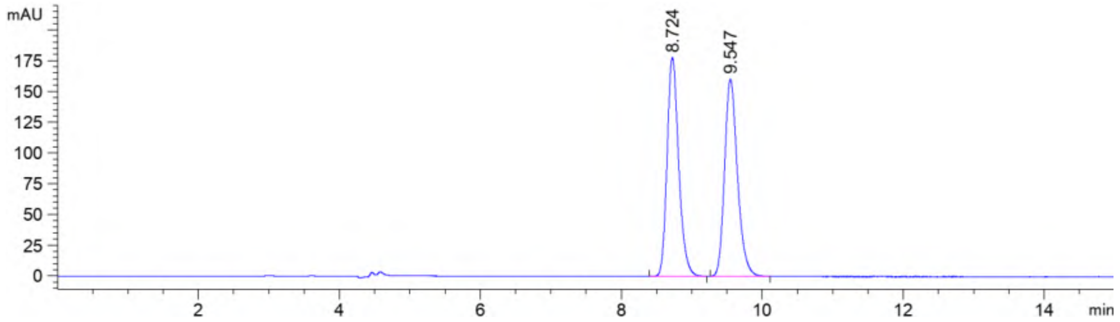

Signal 1: DAD1 A, Sig=254,4 Ref=360,100

Peak #	RetTime [min]	Type	Width [min]	Area [mAU*s]	Height [mAU]	Area %
1	8.724	BB	0.1706	2039.15894	178.12386	50.0125
2	9.547	BV R	0.1928	2038.14075	160.34293	49.9875

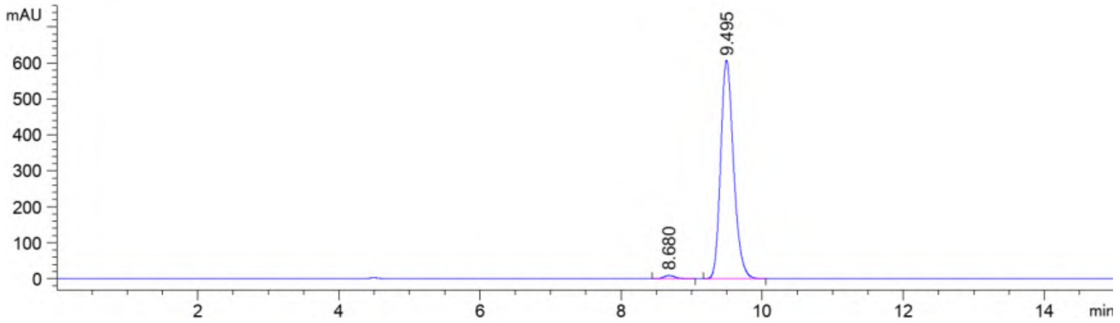

Signal 1: DAD1 A, Sig=254,4 Ref=360,100

Peak #	RetTime [min]	Type	Width [min]	Area [mAU*s]	Height [mAU]	Area %
1	8.680	VB R	0.1427	102.98231	8.82426	1.3154
2	9.495	BV R	0.1955	7725.72461	606.98486	98.6846

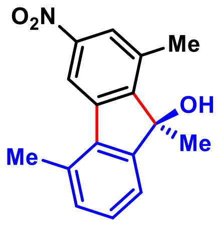

3aq

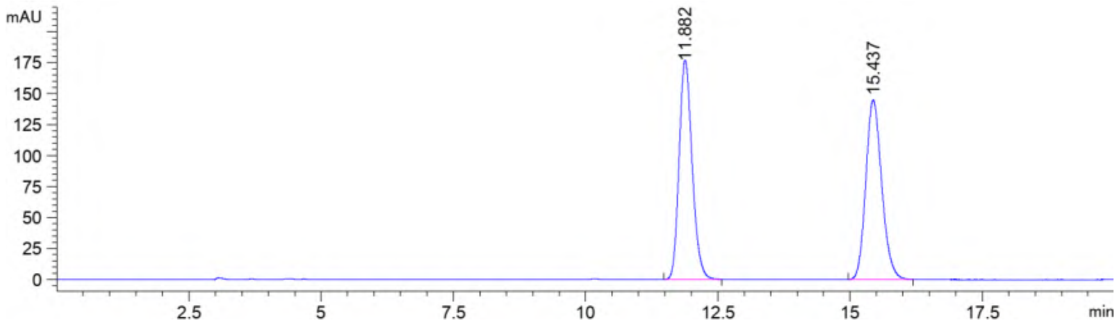

Signal 1: DAD1 A, Sig=254,4 Ref=360,100

Peak #	RetTime [min]	Type	Width [min]	Area [mAU*s]	Height [mAU]	Area %
1	11.882	BB	0.2474	3024.59180	176.99170	49.9330
2	15.437	BB	0.2752	3032.70459	144.86618	50.0670

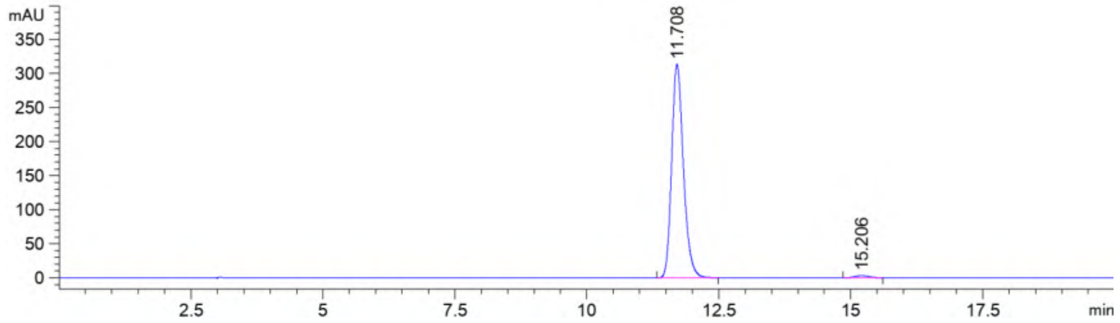

Signal 1: DAD1 A, Sig=254,4 Ref=360,100

Peak #	RetTime [min]	Type	Width [min]	Area [mAU*s]	Height [mAU]	Area %
1	11.708	BV R	0.2300	4852.18555	313.56400	98.7863
2	15.206	BB	0.2202	59.61583	3.21731	1.2137

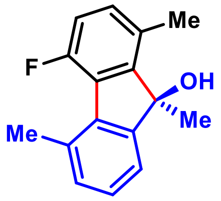

3ar

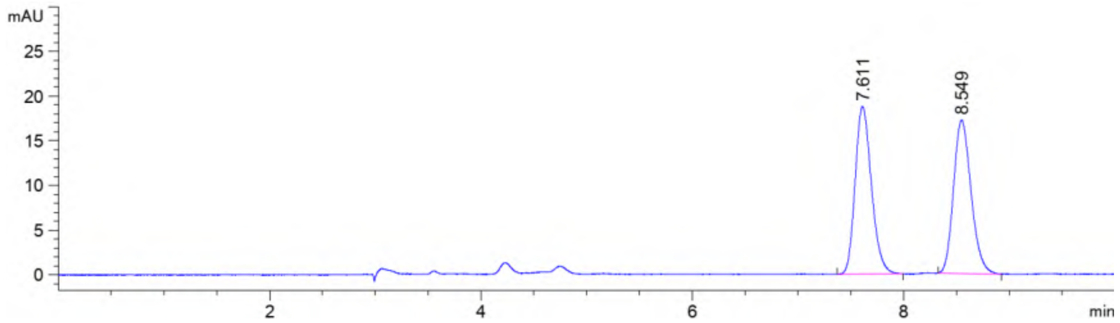

Signal 1: DAD1 A, Sig=254,4 Ref=360,100

Peak #	RetTime [min]	Type	Width [min]	Area [mAU*s]	Height [mAU]	Area %
1	7.611	BB	0.1516	200.95416	18.74254	50.3596
2	8.549	BB	0.1586	198.08443	17.16640	49.6404

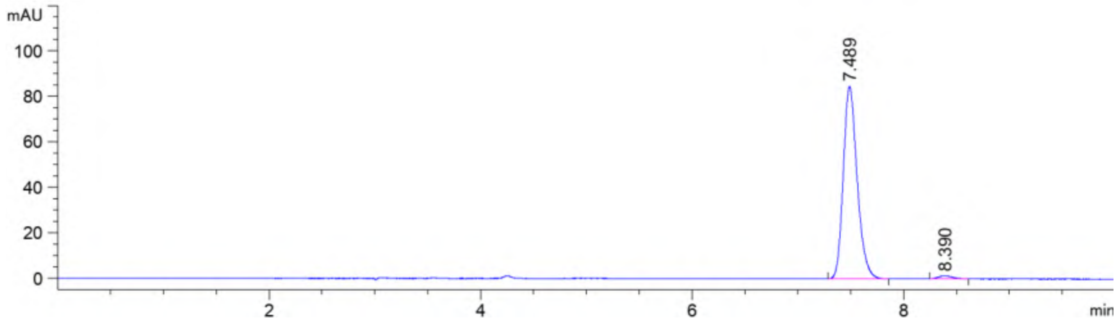

Signal 1: DAD1 A, Sig=254,4 Ref=360,100

Peak #	RetTime [min]	Type	Width [min]	Area [mAU*s]	Height [mAU]	Area %
1	7.489	BB	0.1398	772.48230	84.75629	98.4003
2	8.390	BB	0.1145	12.55841	1.30224	1.5997

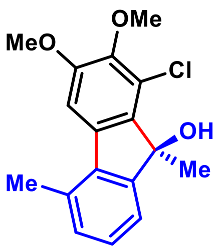

3as

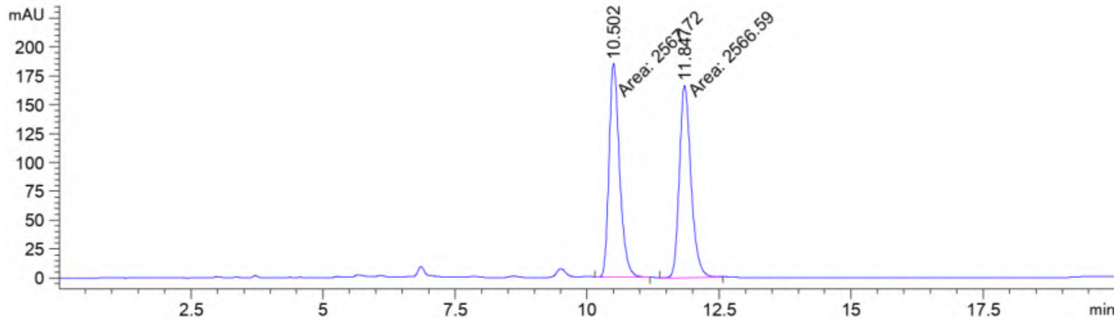

Signal 1: DAD1 D, Sig=230,4 Ref=360,100

Peak #	RetTime [min]	Type	Width [min]	Area [mAU*s]	Height [mAU]	Area %
1	10.502	MM	0.2314	2567.72339	184.93939	50.0111
2	11.847	MM	0.2570	2566.58789	166.41389	49.9889

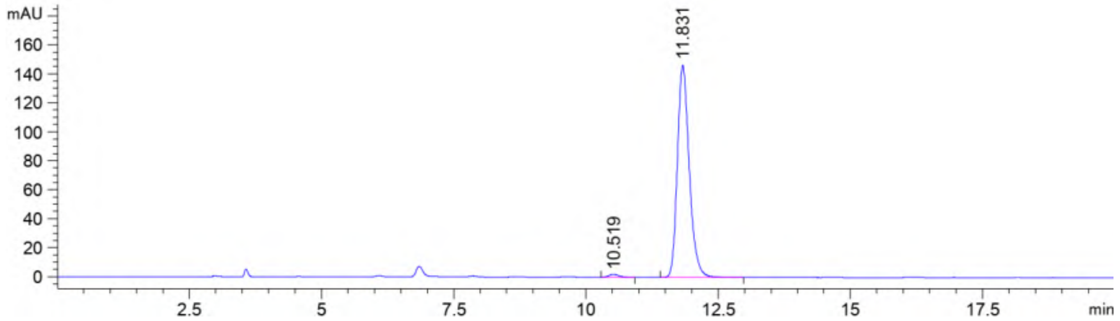

Signal 1: DAD1 D, Sig=230,4 Ref=360,100

Peak #	RetTime [min]	Type	Width [min]	Area [mAU*s]	Height [mAU]	Area %
1	10.519	BB	0.1942	26.36166	1.94453	1.1387
2	11.831	BB	0.2381	2288.79614	146.64592	98.8613

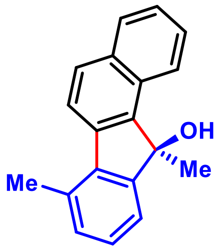

3at

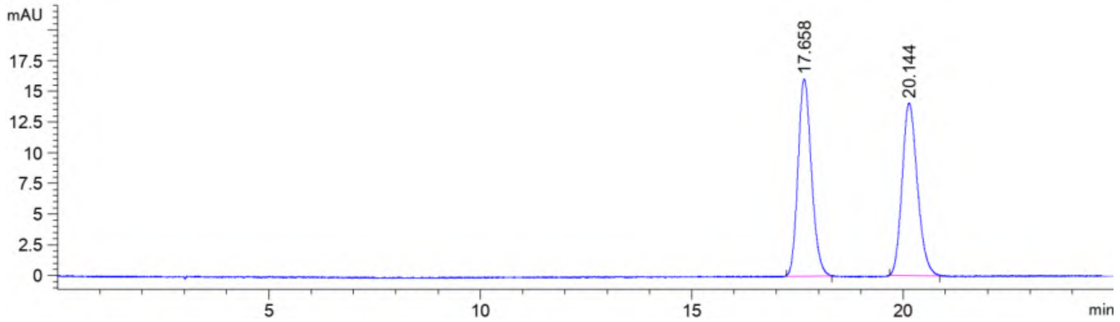

Signal 1: DAD1 E, Sig=320,4 Ref=360,100

Peak #	RetTime [min]	Type	Width [min]	Area [mAU*s]	Height [mAU]	Area %
1	17.658	BB	0.2625	353.89822	16.03546	50.1996
2	20.144	BB	0.2940	351.08435	14.02566	49.8004

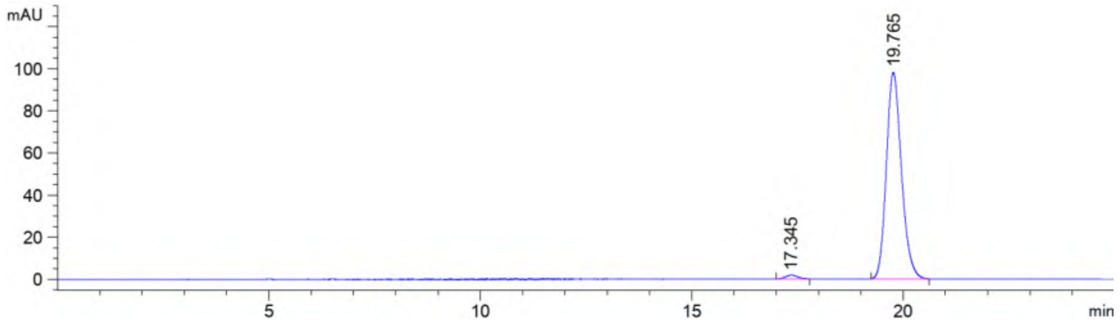

Signal 1: DAD1 E, Sig=320,4 Ref=360,100

Peak #	RetTime [min]	Type	Width [min]	Area [mAU*s]	Height [mAU]	Area %
1	17.345	BB	0.2388	37.46108	1.84144	1.5206
2	19.765	BB	0.3072	2426.11206	98.04559	98.4794

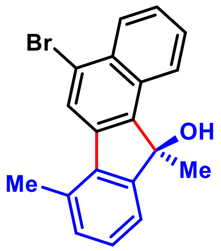

3au

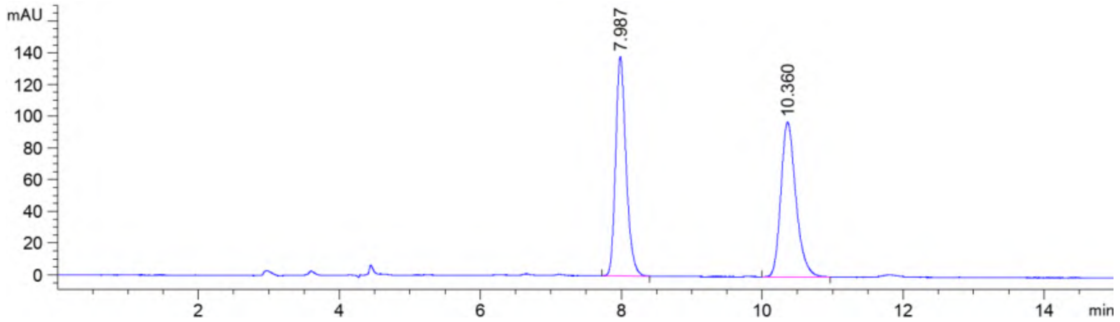

Signal 1: DAD1 D, Sig=230,4 Ref=360,100

Peak #	RetTime [min]	Type	Width [min]	Area [mAU*s]	Height [mAU]	Area %
1	7.987	BB	0.1526	1424.70190	138.38045	49.6852
2	10.360	VV R	0.2035	1442.75696	97.55508	50.3148

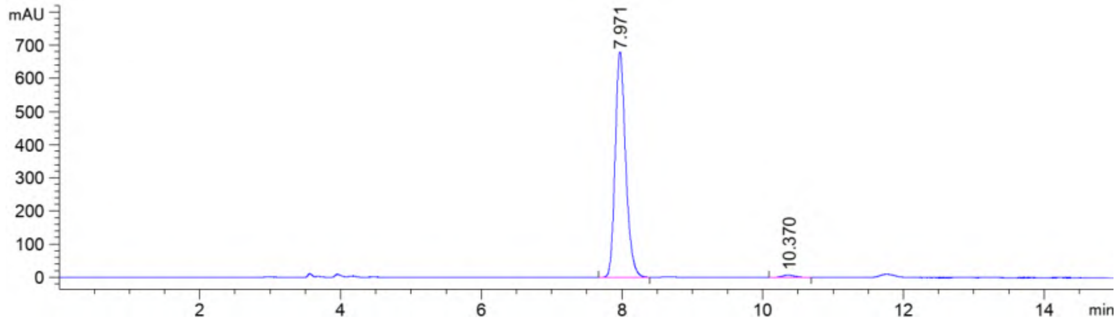

Signal 1: DAD1 D, Sig=230,4 Ref=360,100

Peak #	RetTime [min]	Type	Width [min]	Area [mAU*s]	Height [mAU]	Area %
1	7.971	BB	0.1585	7048.28809	679.71826	98.7116
2	10.370	VV R	0.1556	91.99767	6.96287	1.2884

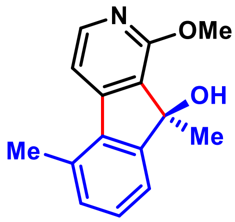

3av

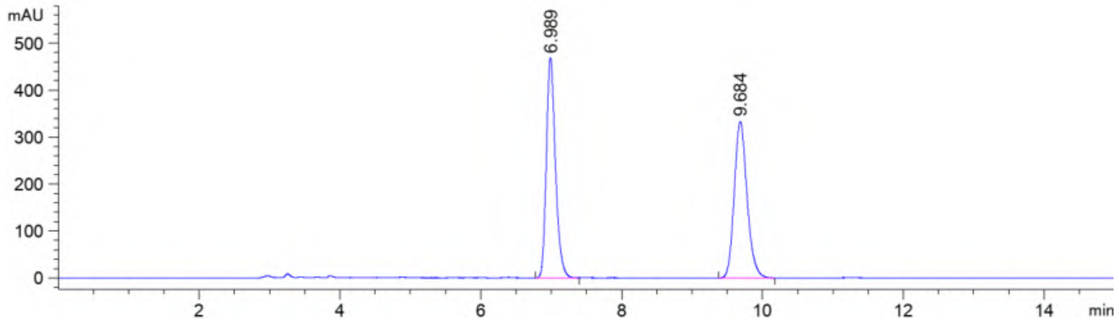

Signal 1: DAD1 D, Sig=230,4 Ref=360,100

Peak #	RetTime [min]	Type	Width [min]	Area [mAU*s]	Height [mAU]	Area %
1	6.989	BV R	0.1354	4196.79688	469.10056	49.9384
2	9.684	VV R	0.1864	4207.15723	332.94958	50.0616

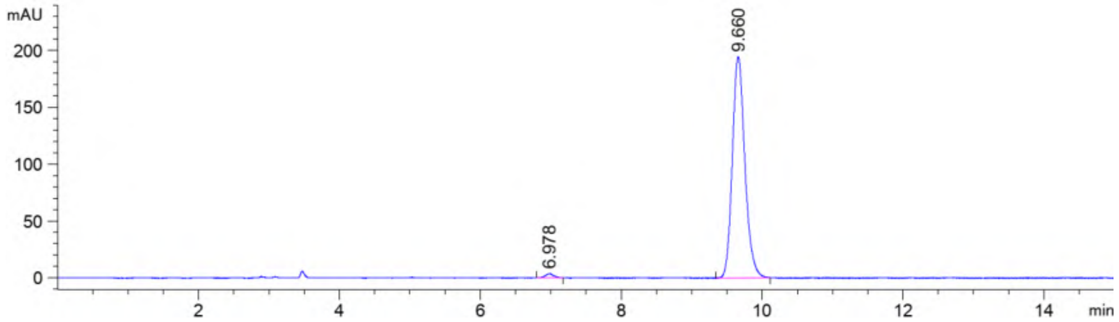

Signal 1: DAD1 D, Sig=230,4 Ref=360,100

Peak #	RetTime [min]	Type	Width [min]	Area [mAU*s]	Height [mAU]	Area %
1	6.978	VV R	0.0968	29.98355	3.67082	1.2294
2	9.660	VV R	0.1803	2408.93066	194.72133	98.7706

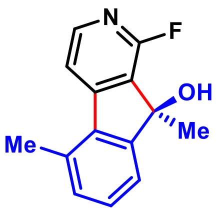

3aw

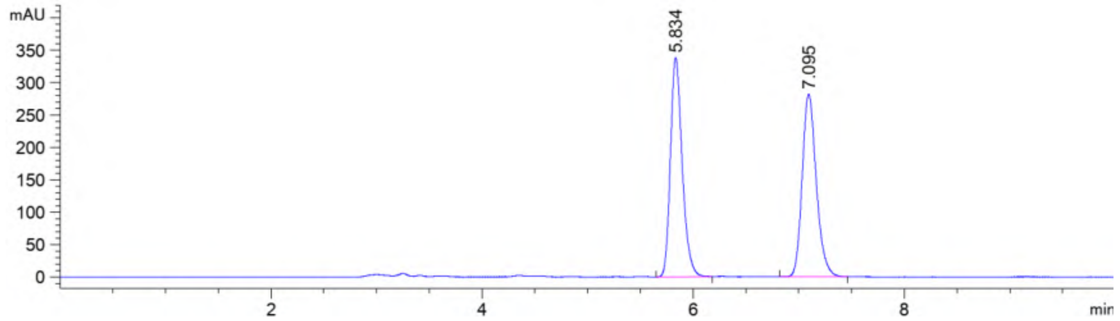

Signal 1: DAD1 D, Sig=230,4 Ref=360,100

Peak #	RetTime [min]	Type	Width [min]	Area [mAU*s]	Height [mAU]	Area %
1	5.834	BV R	0.1178	2619.55786	338.31390	50.0023
2	7.095	VV R	0.1393	2619.31396	282.21066	49.9977

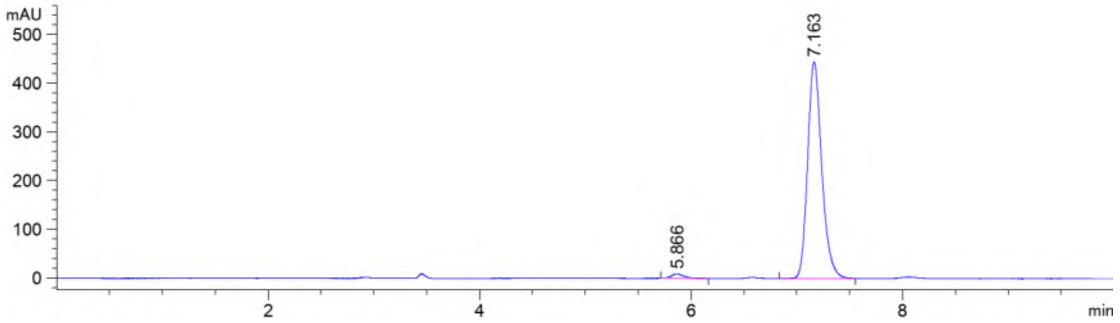

Signal 1: DAD1 D, Sig=230,4 Ref=360,100

Peak #	RetTime [min]	Type	Width [min]	Area [mAU*s]	Height [mAU]	Area %
1	5.866	BV R	0.0970	70.43430	8.83640	1.6781
2	7.163	VV R	0.1392	4126.89404	445.05521	98.3219

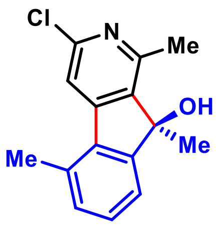

3ax

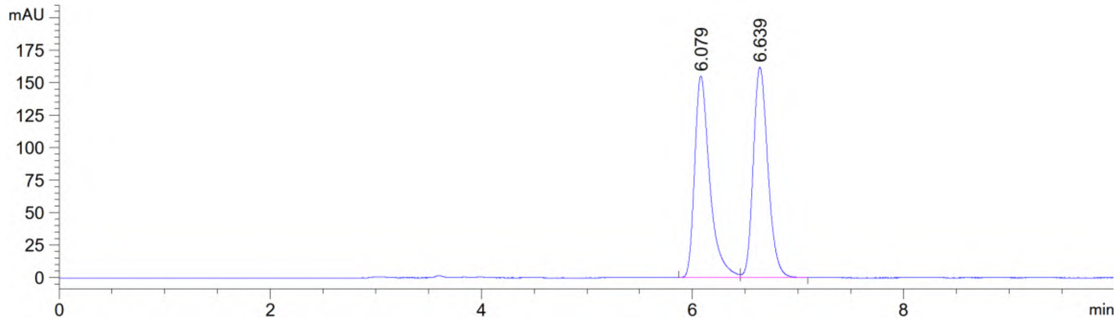

Signal 1: DAD1 A, Sig=254,4 Ref=360,100

Peak #	RetTime [min]	Type	Width [min]	Area [mAU*s]	Height [mAU]	Area %
1	6.079	BV	0.1463	1549.03394	155.43782	49.7382
2	6.639	VV R	0.1486	1565.33948	162.13165	50.2618

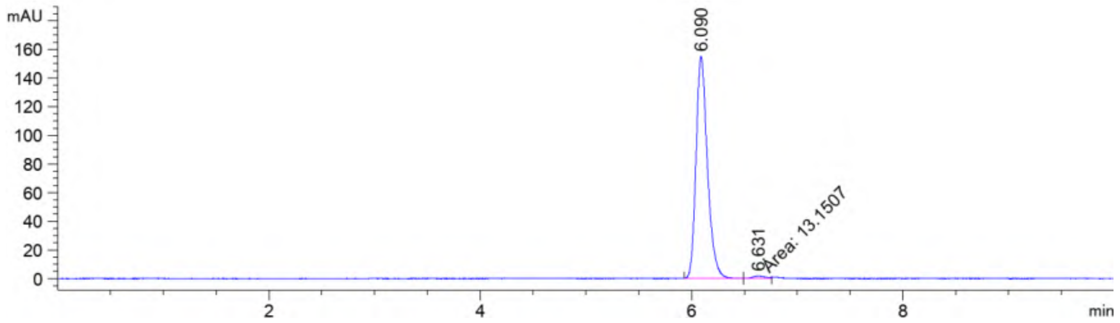

Signal 1: DAD1 A, Sig=254,4 Ref=360,100

Peak #	RetTime [min]	Type	Width [min]	Area [mAU*s]	Height [mAU]	Area %
1	6.090	BV R	0.1130	1160.76575	154.77463	98.8798
2	6.631	MM	0.1341	13.15072	1.63427	1.1202

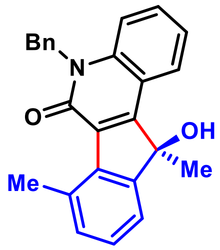

3ay

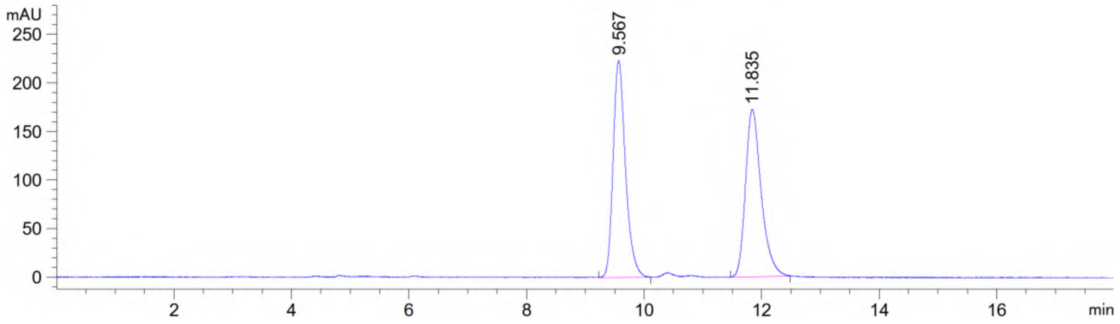

Signal 1: DAD1 A, Sig=254,4 Ref=360,100

Peak #	RetTime [min]	Type	Width [min]	Area [mAU*s]	Height [mAU]	Area %
1	9.567	VV R	0.1728	3253.71777	223.51363	50.4108
2	11.835	VV R	0.2201	3200.69312	172.38033	49.5892

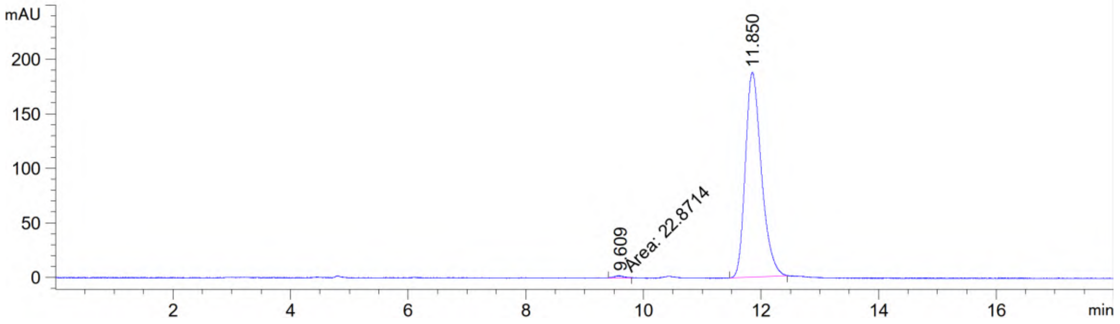

Signal 1: DAD1 A, Sig=254,4 Ref=360,100

Peak #	RetTime [min]	Type	Width [min]	Area [mAU*s]	Height [mAU]	Area %
1	9.609	MM	0.1825	22.87144	2.08923	0.6374
2	11.850	VV R	0.2244	3565.40991	187.87999	99.3626

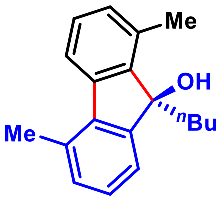

3ba

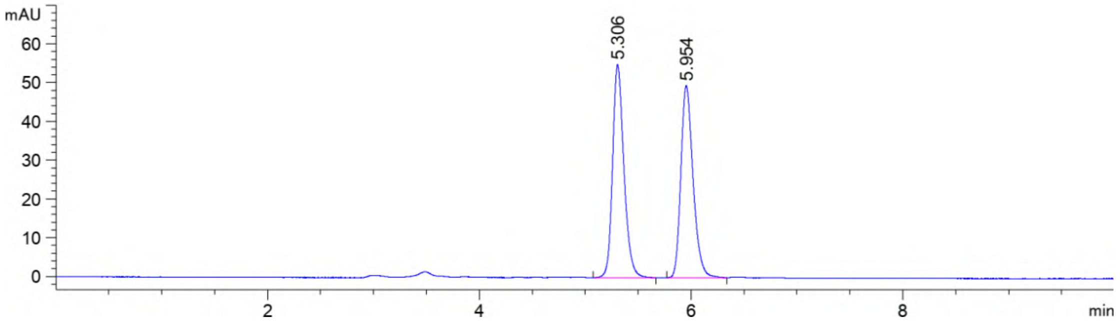

Signal 1: DAD1 A, Sig=254,4 Ref=360,100

Peak #	RetTime [min]	Type	Width [min]	Area [mAU*s]	Height [mAU]	Area %
1	5.306	BB	0.1131	407.81915	54.93470	50.7475
2	5.954	BB	0.1218	395.80484	49.48656	49.2525

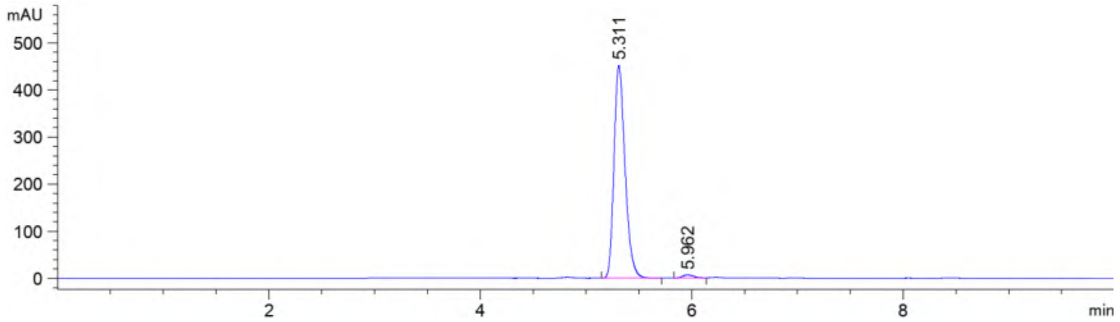

Signal 1: DAD1 A, Sig=254,4 Ref=360,100

Peak #	RetTime [min]	Type	Width [min]	Area [mAU*s]	Height [mAU]	Area %
1	5.311	BB	0.1116	3275.56616	451.95529	98.4183
2	5.962	BV	0.0992	52.64090	6.73660	1.5817

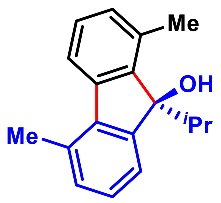

3ca

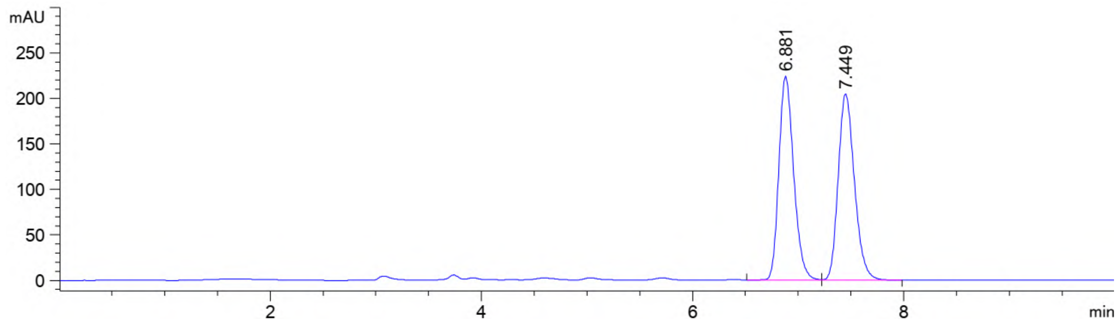

Signal 1: DAD1 D, Sig=230,4 Ref=360,100

Peak #	RetTime [min]	Type	Width [min]	Area [mAU*s]	Height [mAU]	Area %
1	6.881	BV	0.1515	2177.38672	223.78226	49.9184
2	7.449	VB	0.1647	2184.50122	204.36736	50.0816

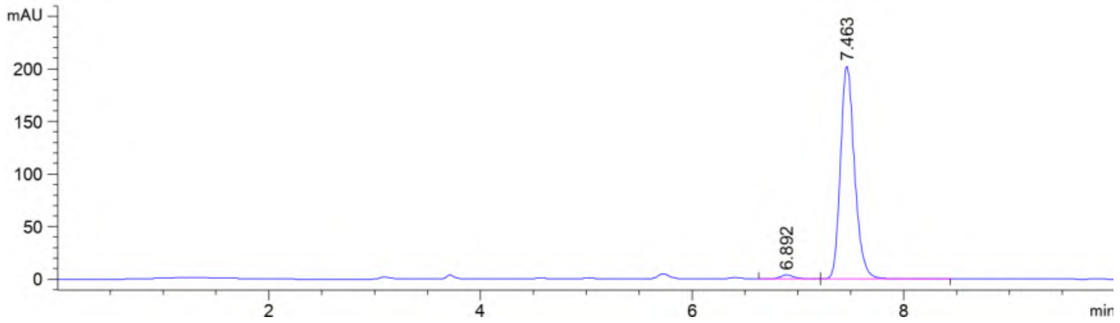

Signal 1: DAD1 D, Sig=230,4 Ref=360,100

Peak #	RetTime [min]	Type	Width [min]	Area [mAU*s]	Height [mAU]	Area %
1	6.892	BV	0.1358	34.00029	3.81980	1.7792
2	7.463	VB	0.1421	1877.01440	202.57722	98.2208

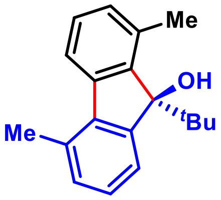

3da

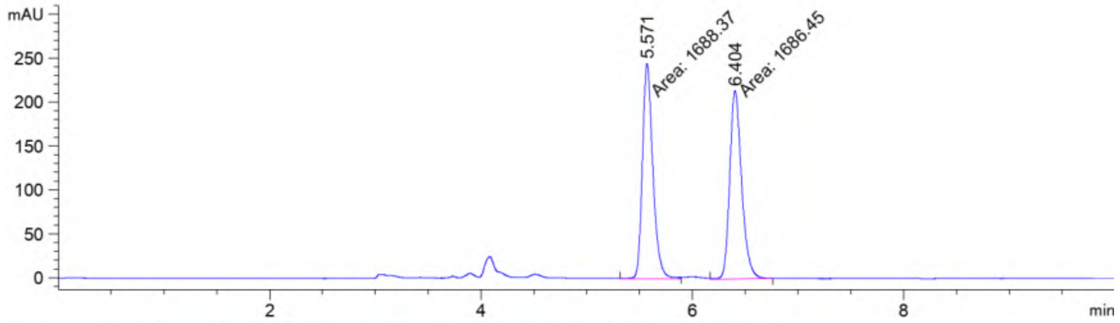

Signal 1: DAD1 B, Sig=220,4 Ref=360,100

Peak #	RetTime [min]	Type	Width [min]	Area [mAU*s]	Height [mAU]	Area %
1	5.571	MM	0.1149	1688.37476	244.93930	50.0285
2	6.404	MM	0.1310	1686.45056	214.57951	49.9715

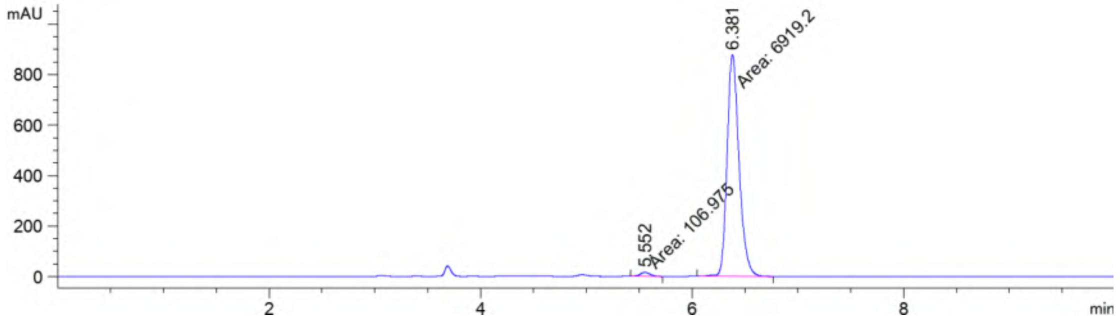

Signal 1: DAD1 B, Sig=220,4 Ref=360,100

Peak #	RetTime [min]	Type	Width [min]	Area [mAU*s]	Height [mAU]	Area %
1	5.552	MM	0.1145	106.97501	15.56902	1.5225
2	6.381	MM	0.1311	6919.20166	879.39630	98.4775

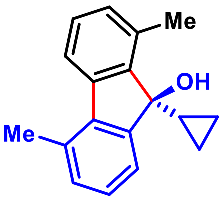

3ea

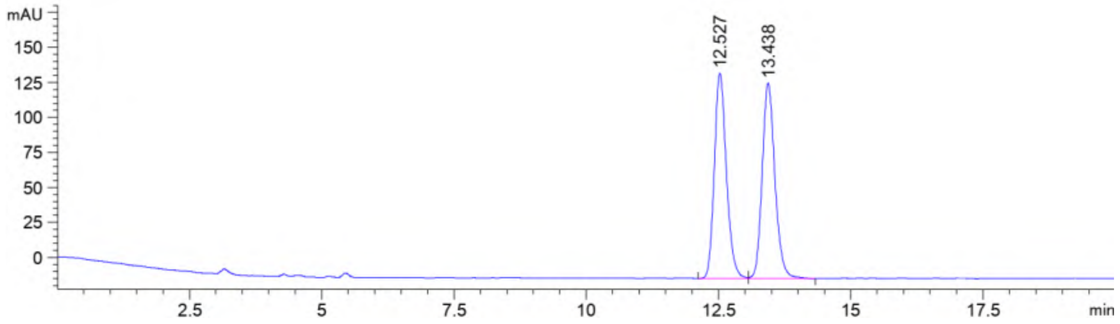

Signal 1: DAD1 A, Sig=220,4 Ref=360,100

Peak #	RetTime [min]	Type	Width [min]	Area [mAU*s]	Height [mAU]	Area %
1	12.527	BV	0.2430	2322.29639	146.45718	49.7851
2	13.438	VB	0.2559	2342.34375	139.48067	50.2149

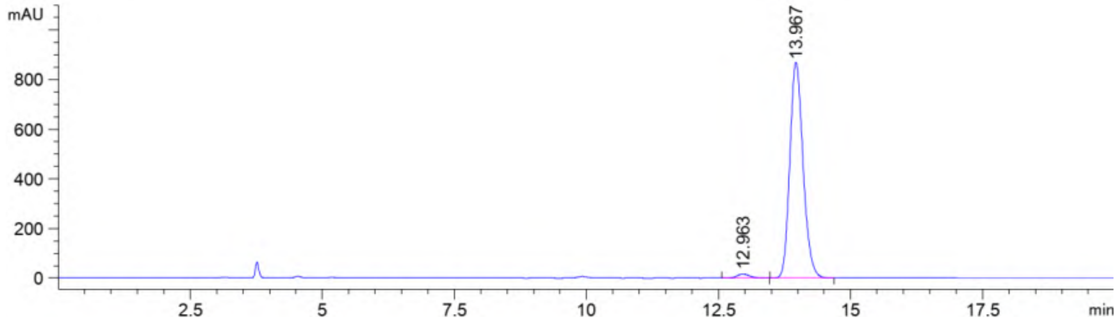

Signal 1: DAD1 A, Sig=220,4 Ref=360,100

Peak #	RetTime [min]	Type	Width [min]	Area [mAU*s]	Height [mAU]	Area %
1	12.963	BB	0.2526	271.39990	16.44066	1.7591
2	13.967	BB	0.2671	1.51566e4	870.56726	98.2409

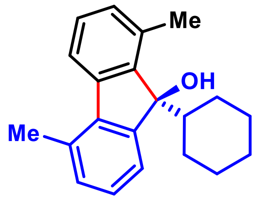

3fa

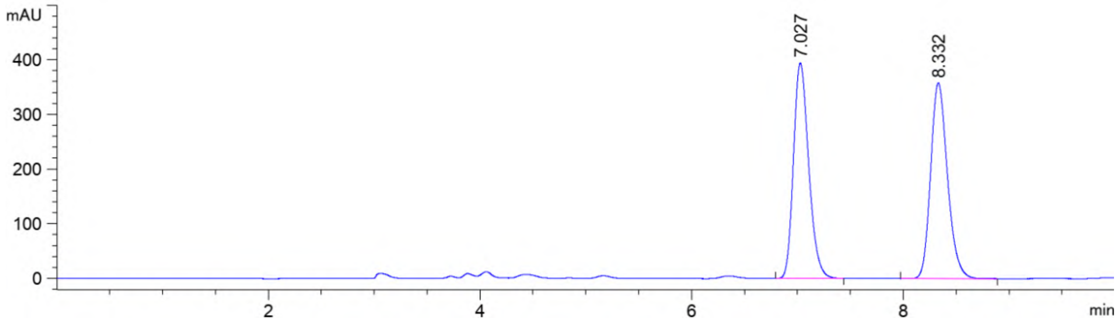

Signal 1: DAD1 B, Sig=220,4 Ref=360,100

Peak #	RetTime [min]	Type	Width [min]	Area [mAU*s]	Height [mAU]	Area %
1	7.027	BB	0.1535	3933.64404	394.06396	49.8266
2	8.332	BB	0.1705	3961.02637	358.10507	50.1734

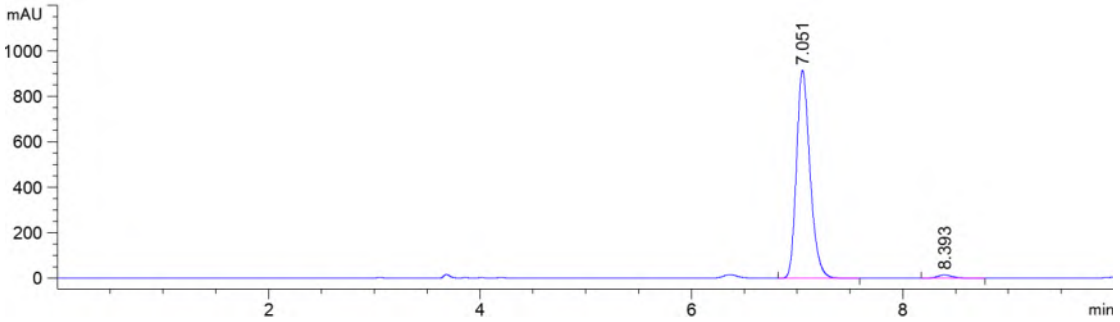

Signal 1: DAD1 D, Sig=230,4 Ref=360,100

Peak #	RetTime [min]	Type	Width [min]	Area [mAU*s]	Height [mAU]	Area %
1	7.051	BB	0.1364	8143.51221	914.17029	98.2078
2	8.393	BB	0.1418	148.60887	14.12356	1.7922

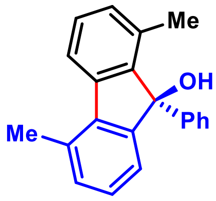

3ga

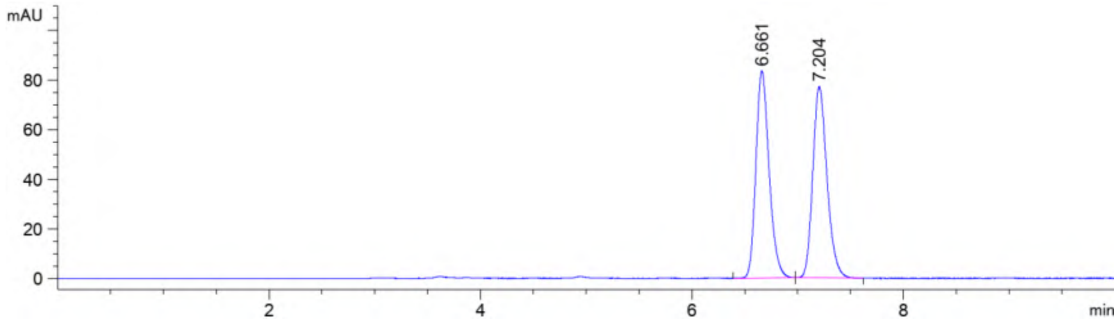

Signal 1: DAD1 F, Sig=290,4 Ref=360,100

Peak #	RetTime [min]	Type	Width [min]	Area [mAU*s]	Height [mAU]	Area %
1	6.661	BB	0.1314	725.24799	83.45788	50.1030
2	7.204	BB	0.1429	722.26733	77.05671	49.8970

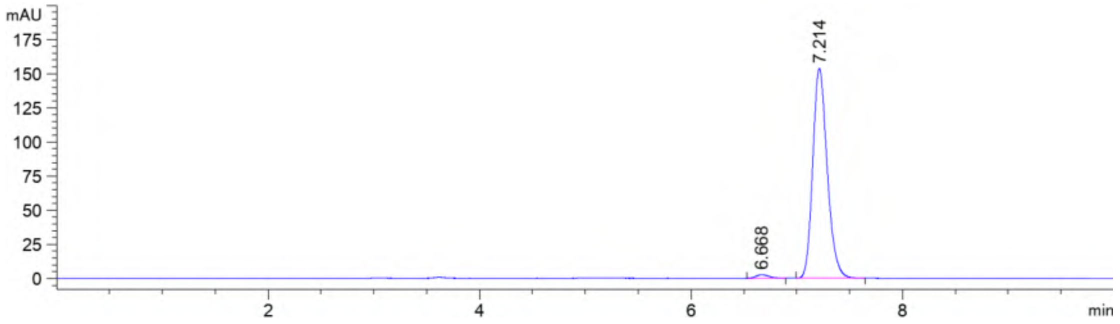

Signal 1: DAD1 F, Sig=290,4 Ref=360,100

Peak #	RetTime [min]	Type	Width [min]	Area [mAU*s]	Height [mAU]	Area %
1	6.668	BB	0.1019	22.14500	2.62667	1.4952
2	7.214	BB	0.1463	1458.93921	153.63699	98.5048

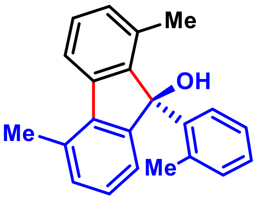

3ha

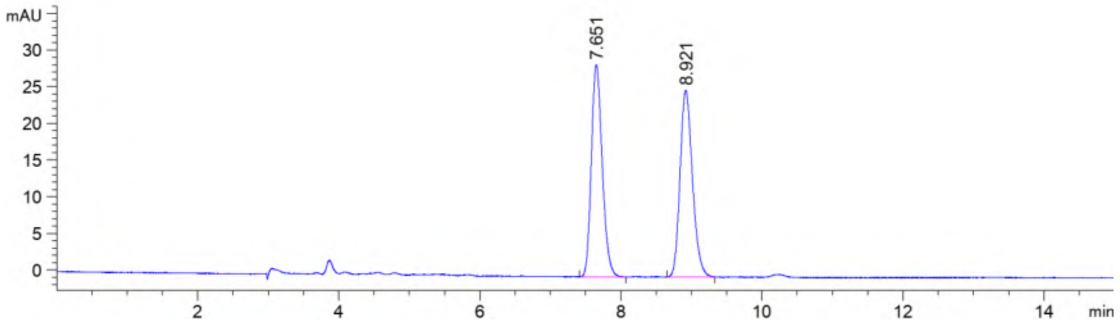

Signal 1: DAD1 A, Sig=254,4 Ref=360,100

Peak #	RetTime [min]	Type	Width [min]	Area [mAU*s]	Height [mAU]	Area %
1	7.651	BB	0.1559	311.03030	28.93756	49.9408
2	8.921	BB	0.1682	311.76743	25.48261	50.0592

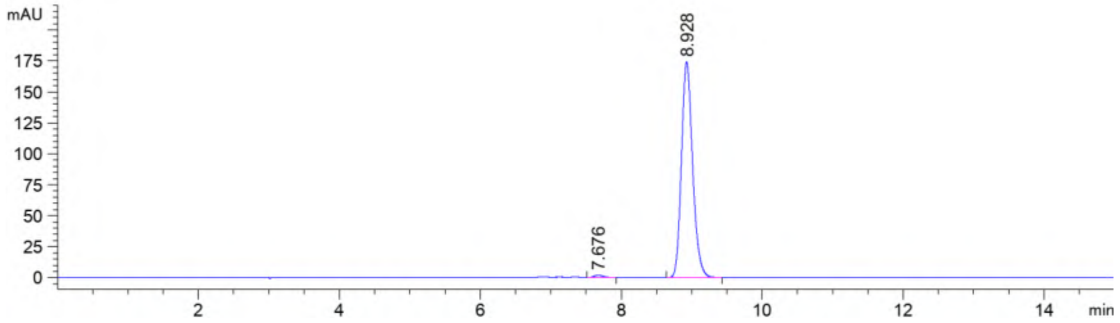

Peak #	RetTime [min]	Type	Width [min]	Area [mAU*s]	Height [mAU]	Area %
1	7.676	BB	0.1100	17.62322	1.91225	0.9111
2	8.928	BB	0.1685	1916.72729	174.02098	99.0889

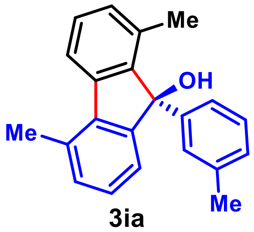

3ia

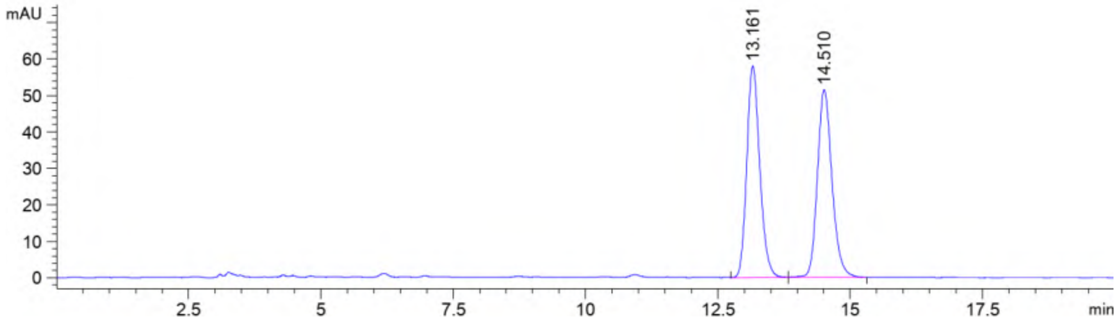

Signal 1: DAD1 D, Sig=230,4 Ref=360,100

Peak #	RetTime [min]	Type	Width [min]	Area [mAU*s]	Height [mAU]	Area %
1	13.161	BB	0.2606	986.88245	57.98687	50.0596
2	14.510	BB	0.2915	984.53168	51.38142	49.9404

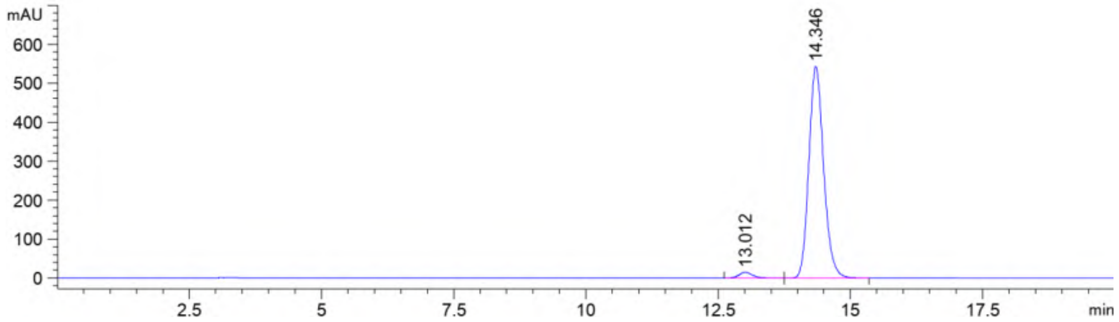

Signal 1: DAD1 D, Sig=230,4 Ref=360,100

Peak #	RetTime [min]	Type	Width [min]	Area [mAU*s]	Height [mAU]	Area %
1	13.012	BB	0.2706	259.81799	14.67437	2.4063
2	14.346	BB	0.2987	1.05374e4	542.33954	97.5937

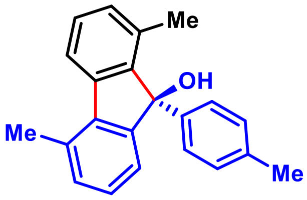

3ja

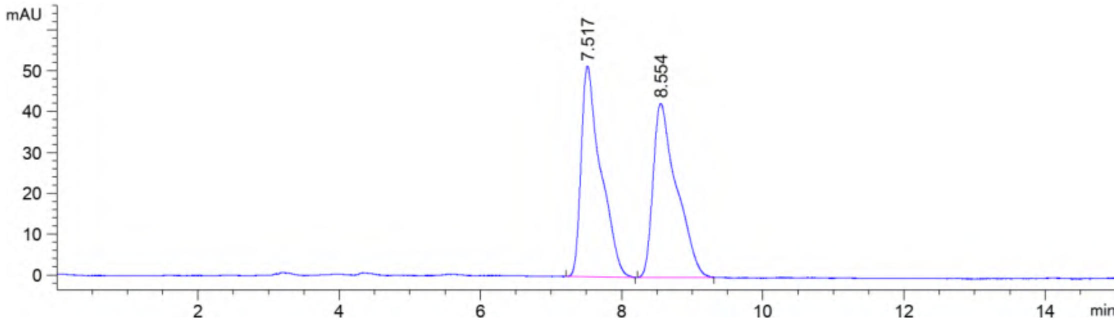

Signal 1: DAD1 D, Sig=230,4 Ref=360,100

Peak #	RetTime [min]	Type	Width [min]	Area [mAU*s]	Height [mAU]	Area %
1	7.517	BB	0.2419	992.66205	51.49978	50.2252
2	8.554	BB	0.2727	983.76099	42.54759	49.7748

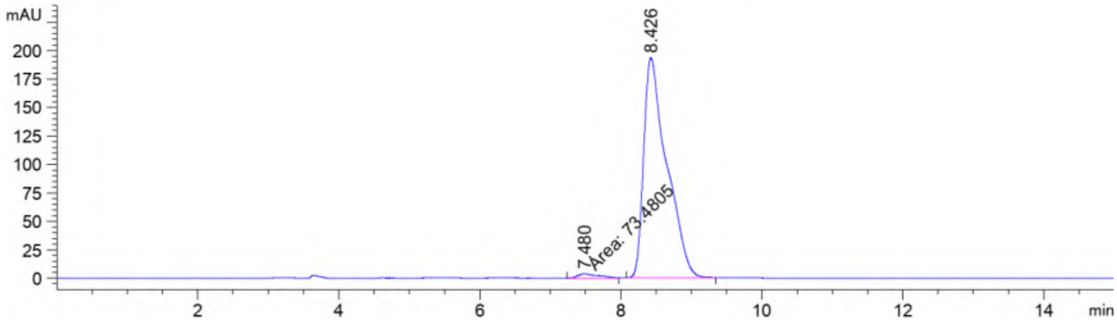

Signal 1: DAD1 D, Sig=230,4 Ref=360,100

Peak #	RetTime [min]	Type	Width [min]	Area [mAU*s]	Height [mAU]	Area %
1	7.480	MM	0.3255	73.48053	3.76213	1.6476
2	8.426	BB	0.3116	4386.24072	193.40601	98.3524

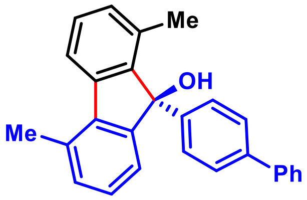

3ka

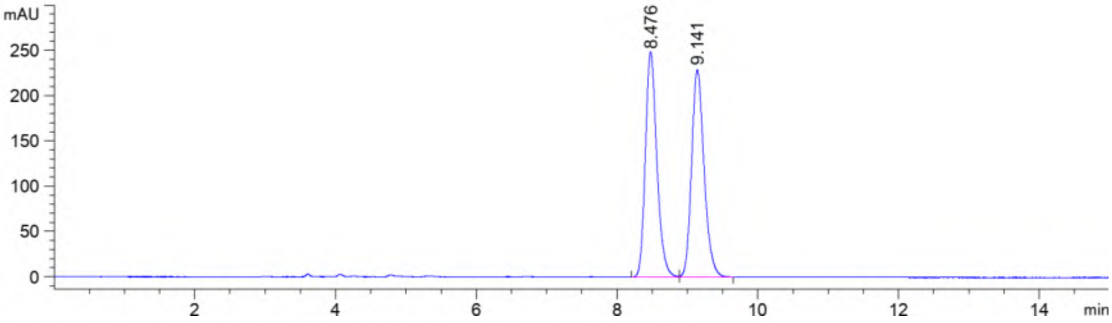

Signal 1: DAD1 A, Sig=254,4 Ref=360,100

Peak #	RetTime [min]	Type	Width [min]	Area [mAU*s]	Height [mAU]	Area %
1	8.476	BV	0.1720	2823.09473	248.60750	49.9448
2	9.141	VV R	0.1833	2829.33057	228.79869	50.0552

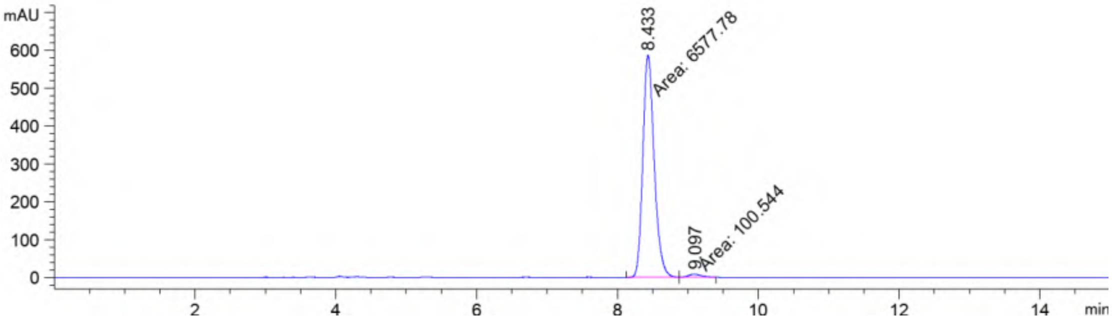

Signal 1: DAD1 A, Sig=254,4 Ref=360,100

Peak #	RetTime [min]	Type	Width [min]	Area [mAU*s]	Height [mAU]	Area %
1	8.433	MF	0.1866	6577.78320	587.44818	98.4945
2	9.097	FM	0.2151	100.54359	7.78874	1.5055

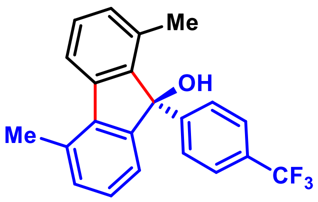

3la

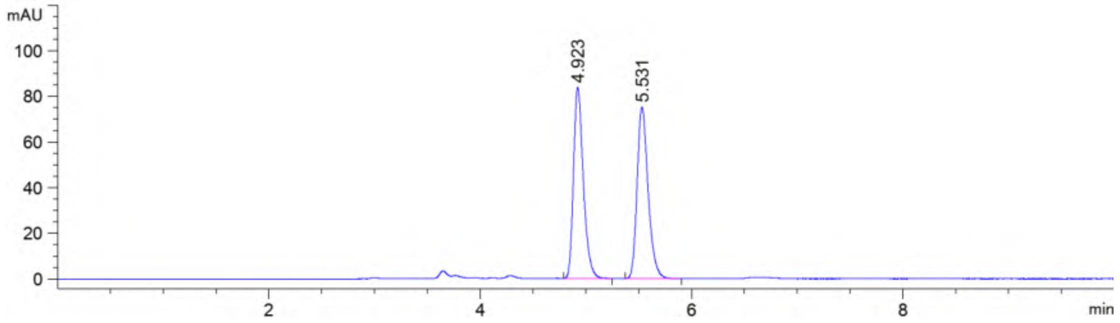

Signal 1: DAD1 F, Sig=290,4 Ref=360,100

Peak #	RetTime [min]	Type	Width [min]	Area [mAU*s]	Height [mAU]	Area %
1	4.923	BB	0.0991	542.07751	83.65853	49.8776
2	5.531	BB	0.1101	544.73743	75.13272	50.1224

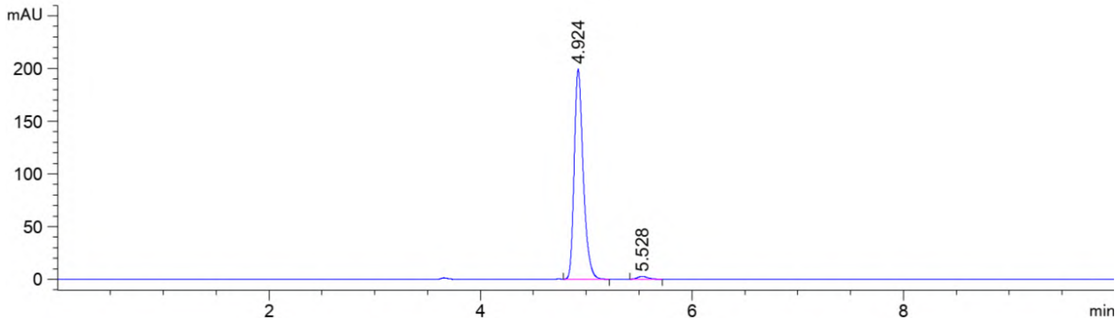

Signal 1: DAD1 F, Sig=290,4 Ref=360,100

Peak #	RetTime [min]	Type	Width [min]	Area [mAU*s]	Height [mAU]	Area %
1	4.924	BB	0.0894	1176.67676	199.30147	98.5317
2	5.528	BB	0.0804	17.53511	2.67628	1.4683

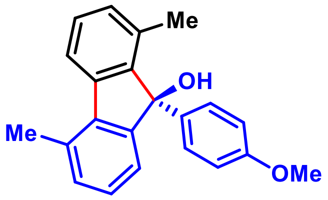

3ma

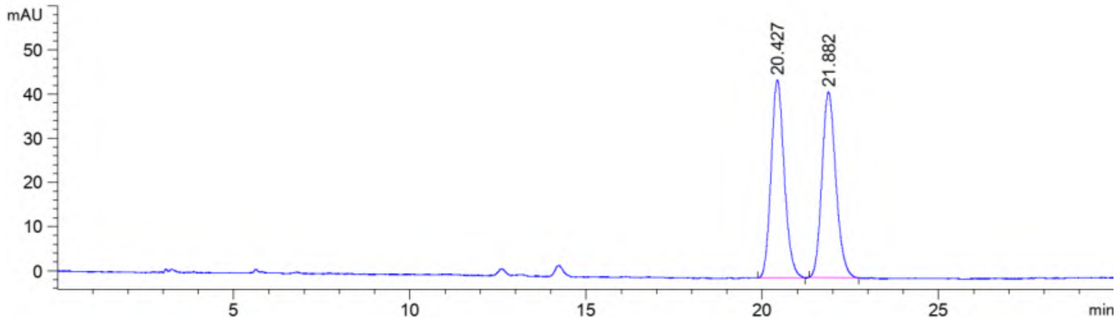

Signal 1: DAD1 D, Sig=230,4 Ref=360,100

Peak #	RetTime [min]	Type	Width [min]	Area [mAU*s]	Height [mAU]	Area %
1	20.427	BB	0.3098	1161.04016	44.81277	50.0875
2	21.882	BB	0.3243	1156.98169	42.01575	49.9125

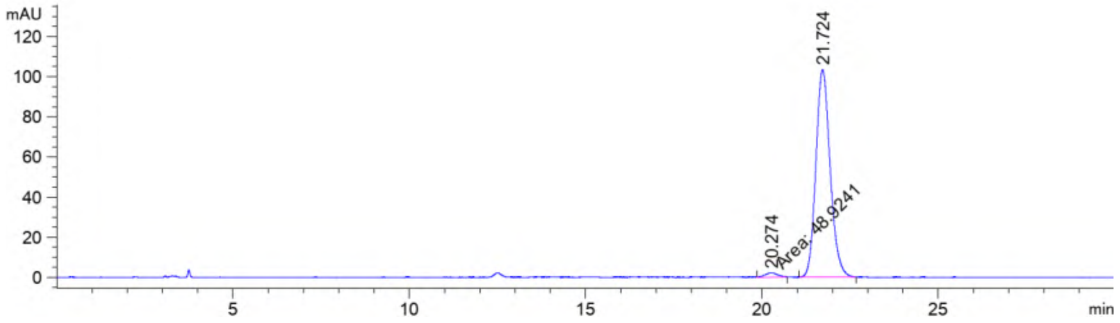

Signal 1: DAD1 D, Sig=230,4 Ref=360,100

Peak #	RetTime [min]	Type	Width [min]	Area [mAU*s]	Height [mAU]	Area %
1	20.274	MM	0.4060	48.92410	2.00837	1.6558
2	21.724	BB	0.3424	2905.75537	103.39957	98.3442

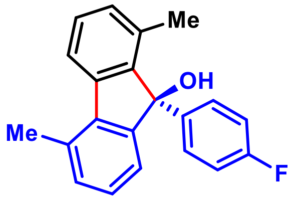

3na

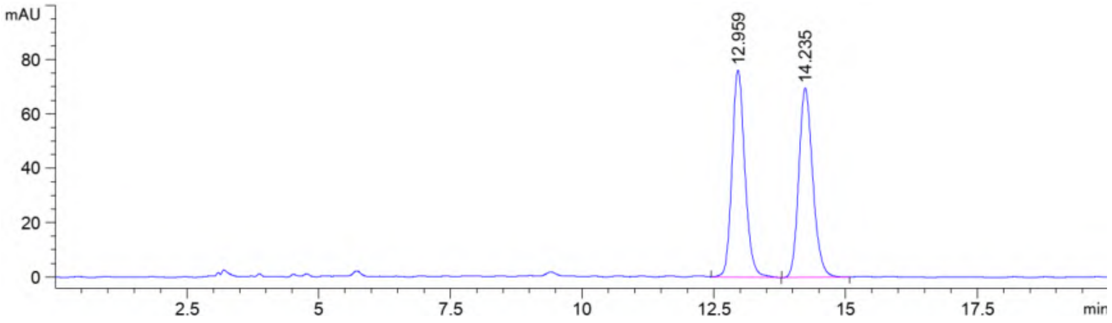

Signal 1: DAD1 A, Sig=220,4 Ref=360,100

Peak #	RetTime [min]	Type	Width [min]	Area [mAU*s]	Height [mAU]	Area %
1	12.959	BB	0.2627	1297.77832	76.20190	50.1705
2	14.235	BB	0.2816	1288.95520	69.74934	49.8295

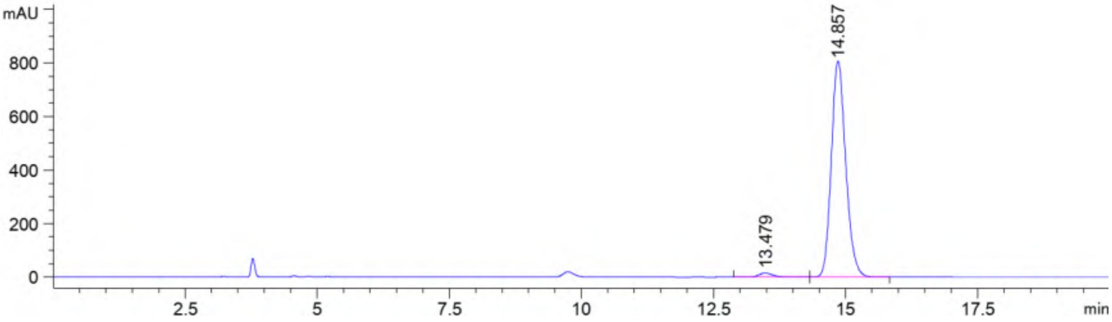

Signal 1: DAD1 A, Sig=220,4 Ref=360,100

Peak #	RetTime [min]	Type	Width [min]	Area [mAU*s]	Height [mAU]	Area %
1	13.479	BB	0.2864	300.62180	15.63046	1.9280
2	14.857	BB	0.2909	1.52919e4	807.48975	98.0720

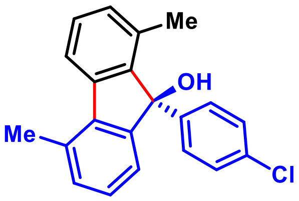

3oa

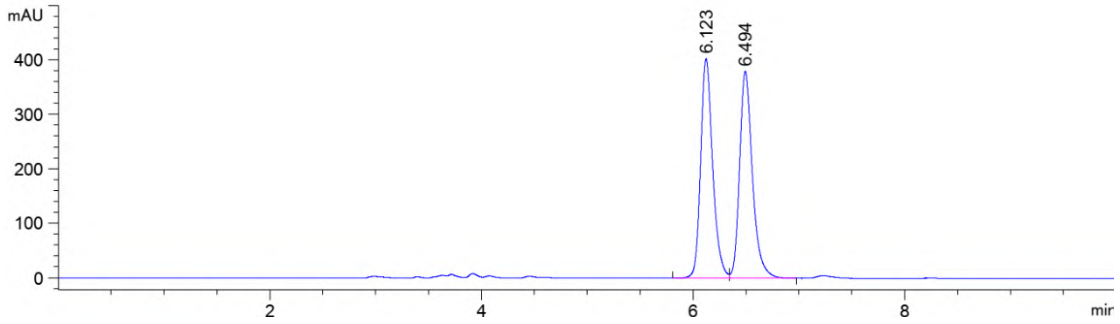

Signal 1: DAD1 D, Sig=230,4 Ref=360,100

Peak #	RetTime [min]	Type	Width [min]	Area [mAU*s]	Height [mAU]	Area %
1	6.123	BV	0.1207	3180.54932	402.51312	49.8817
2	6.494	VV R	0.1278	3195.63306	379.41641	50.1183

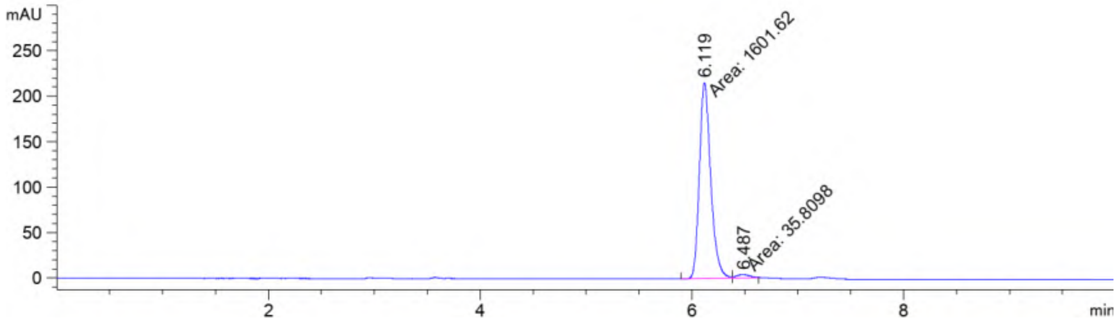

Signal 1: DAD1 D, Sig=230,4 Ref=360,100

Peak #	RetTime [min]	Type	Width [min]	Area [mAU*s]	Height [mAU]	Area %
1	6.119	MF	0.1240	1601.62280	215.32928	97.8131
2	6.487	FM	0.1471	35.80976	4.05618	2.1869

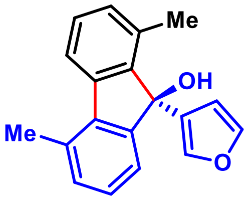

3pa

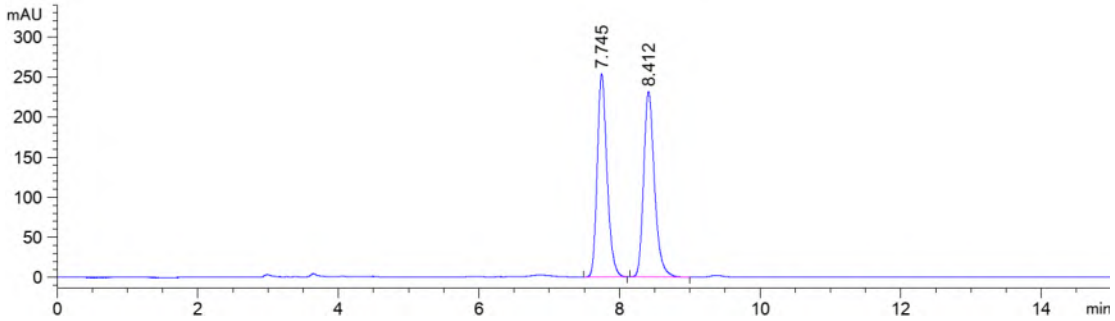

Peak #	RetTime [min]	Type	Width [min]	Area [mAU*s]	Height [mAU]	Area %
1	7.745	BB	0.1476	2444.14429	253.32896	49.6924
2	8.412	BB	0.1617	2474.40332	231.44200	50.3076

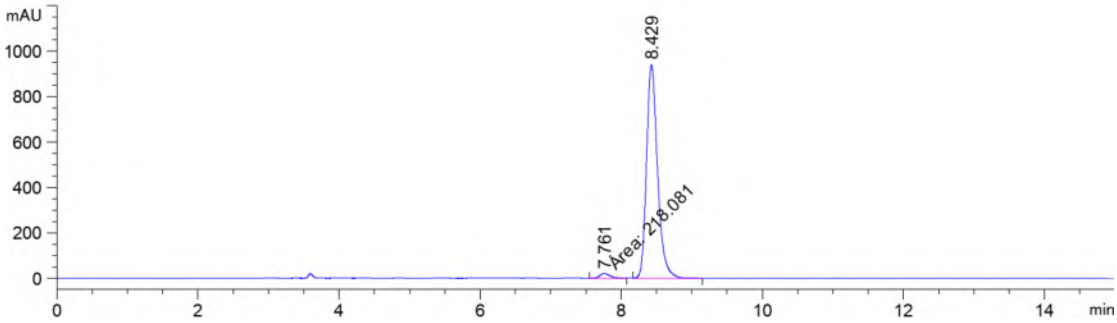

Signal 1: DAD1 D, Sig=230,4 Ref=360,100

Peak #	RetTime [min]	Type	Width [min]	Area [mAU*s]	Height [mAU]	Area %
1	7.761	MF	0.1742	218.08055	20.86007	2.0945
2	8.429	VB	0.1648	1.01941e4	938.28815	97.9055

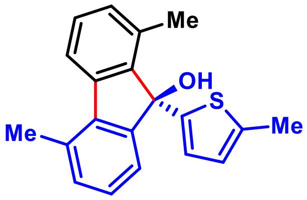

3qa

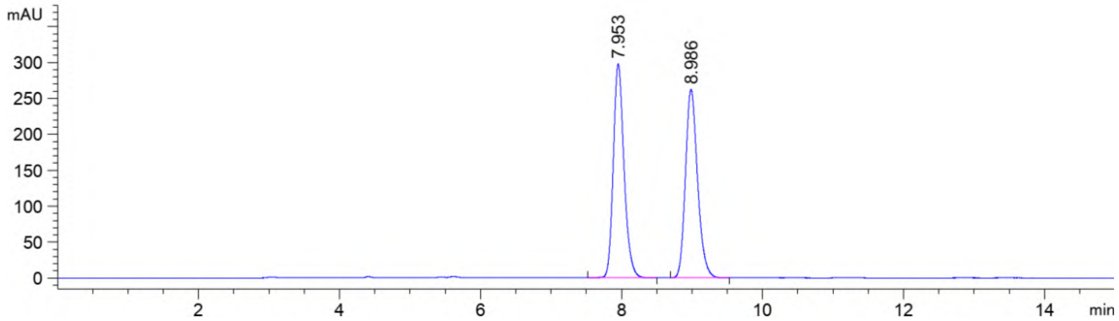

Signal 1: DAD1 A, Sig=254,4 Ref=360,100

Peak #	RetTime [min]	Type	Width [min]	Area [mAU*s]	Height [mAU]	Area %
1	7.953	BB	0.1600	3151.62964	297.73566	50.1591
2	8.986	BB	0.1827	3131.63306	262.56067	49.8409

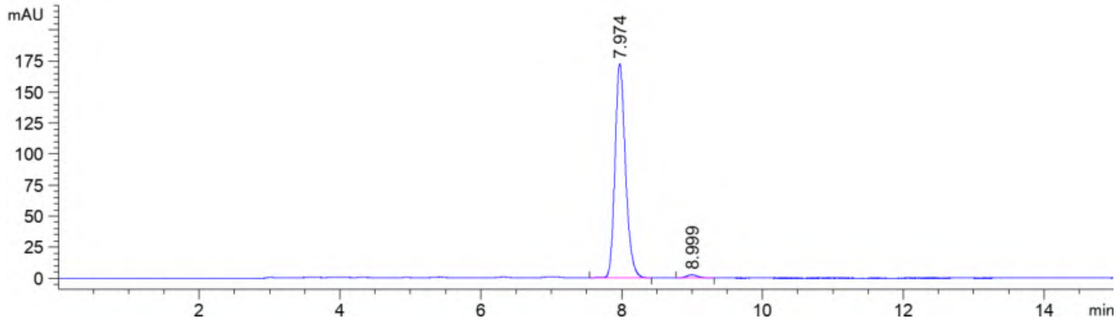

Signal 1: DAD1 A, Sig=254,4 Ref=360,100

Peak #	RetTime [min]	Type	Width [min]	Area [mAU*s]	Height [mAU]	Area %
1	7.974	BB	0.1597	1793.24072	172.65329	98.4200
2	8.999	BB	0.1366	28.78770	2.51315	1.5800

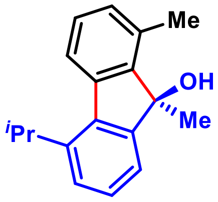

3ra

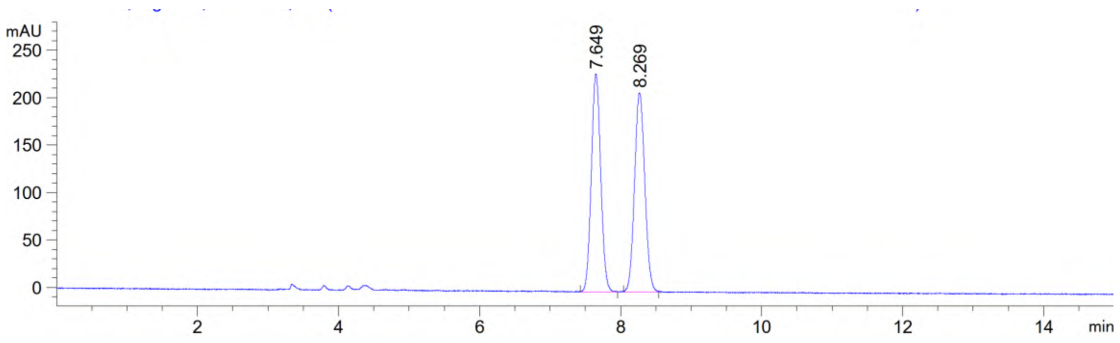

Signal 1: DAD1 D, Sig=230,4 Ref=360,100

Peak #	RetTime [min]	Type	Width [min]	Area [mAU*s]	Height [mAU]	Area %
1	7.649	VV R	0.1248	2132.60156	229.78181	50.1847
2	8.269	VV R	0.1213	2116.90674	209.66721	49.8153

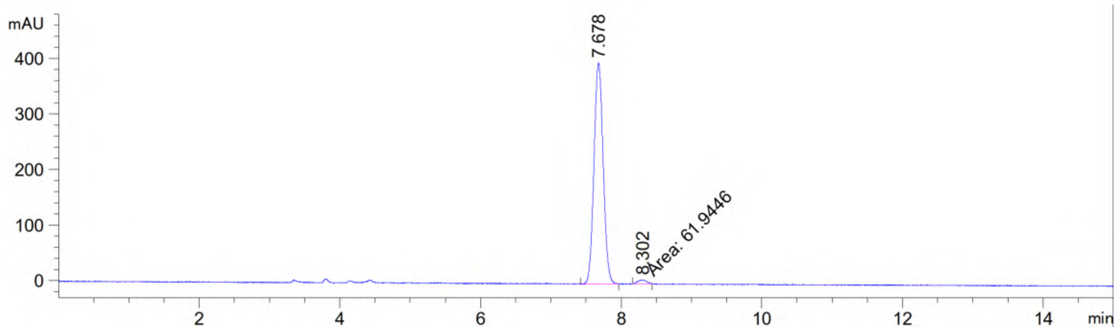

Signal 1: DAD1 D, Sig=230,4 Ref=360,100

Peak #	RetTime [min]	Type	Width [min]	Area [mAU*s]	Height [mAU]	Area %
1	7.678	VV R	0.1206	3603.72534	398.19174	98.3101
2	8.302	MM	0.1453	61.94461	7.10617	1.6899

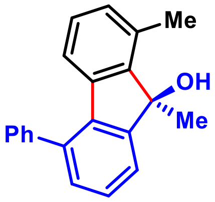

3sa

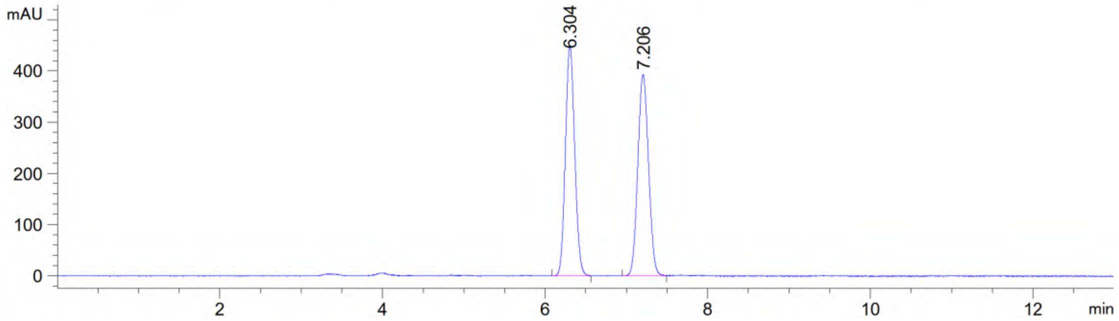

Signal 1: DAD1 D, Sig=230,4 Ref=360,100

Peak #	RetTime [min]	Type	Width [min]	Area [mAU*s]	Height [mAU]	Area %
1	6.304	VV R	0.1039	3581.75757	450.00714	49.8752
2	7.206	VV R	0.1128	3599.68066	393.14920	50.1248

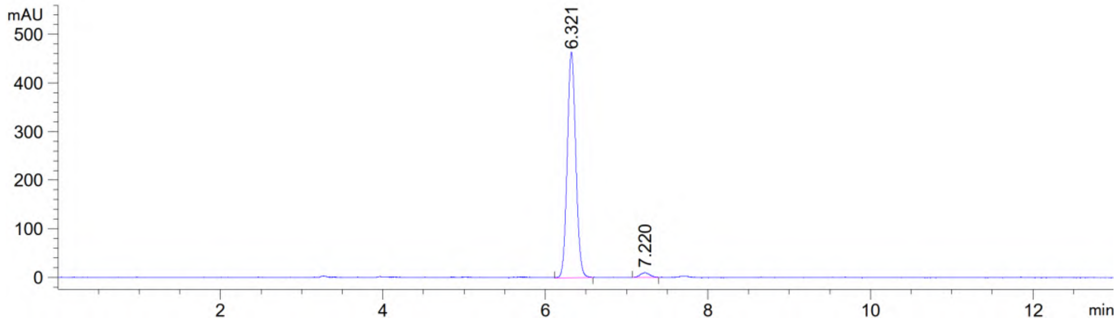

Signal 1: DAD1 D, Sig=230,4 Ref=360,100

Peak #	RetTime [min]	Type	Width [min]	Area [mAU*s]	Height [mAU]	Area %
1	6.321	VV R	0.1042	3386.94727	463.51840	97.4880
2	7.220	VV R	0.1006	87.27064	10.32226	2.5120

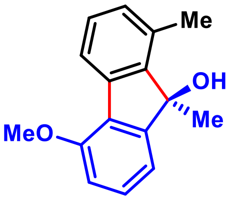

3ta

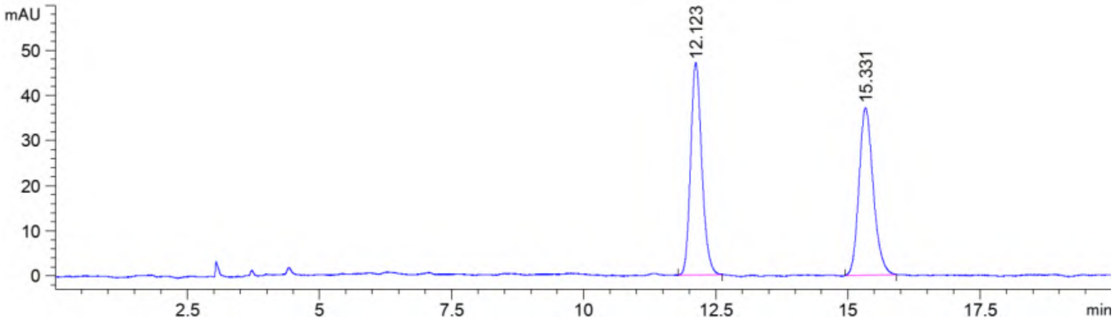

Signal 1: DAD1 B, Sig=220,4 Ref=off

Peak #	RetTime [min]	Type	Width [min]	Area [mAU*s]	Height [mAU]	Area %
1	12.123	BV R	0.1873	692.55377	47.03762	49.8845
2	15.331	BV R	0.2233	695.76215	37.10342	50.1155

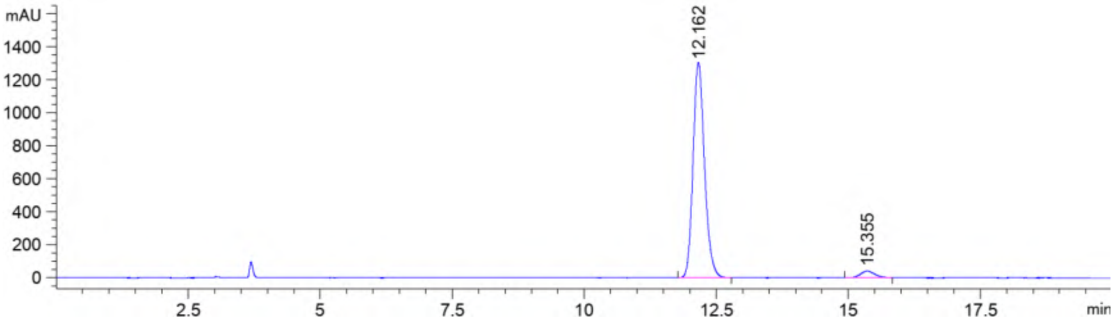

Signal 1: DAD1 B, Sig=220,4 Ref=off

Peak #	RetTime [min]	Type	Width [min]	Area [mAU*s]	Height [mAU]	Area %
1	12.162	BV R	0.2244	1.96710e4	1304.69824	96.4049
2	15.355	BB	0.2183	733.56598	39.56051	3.5951

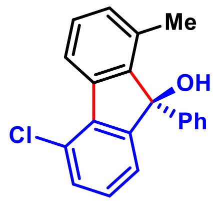

3ua

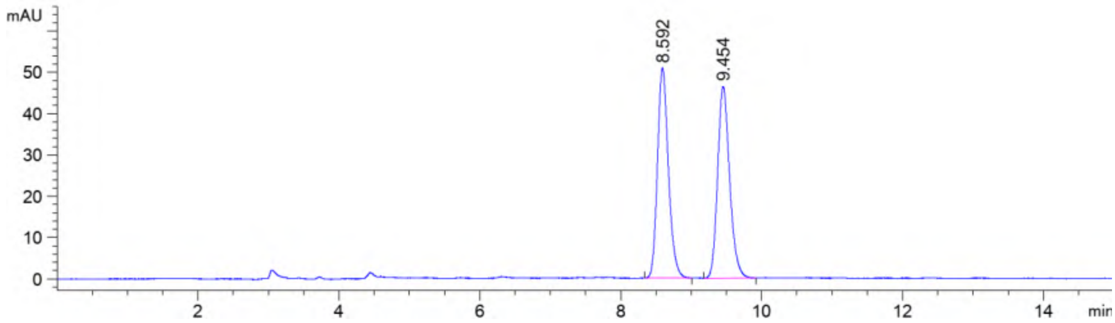

Signal 1: DAD1 D, Sig=230,4 Ref=360,100

Peak #	RetTime [min]	Type	Width [min]	Area [mAU*s]	Height [mAU]	Area %
1	8.592	BB	0.1485	547.54083	50.88694	49.9394
2	9.454	BV R	0.1633	548.86938	46.46769	50.0606

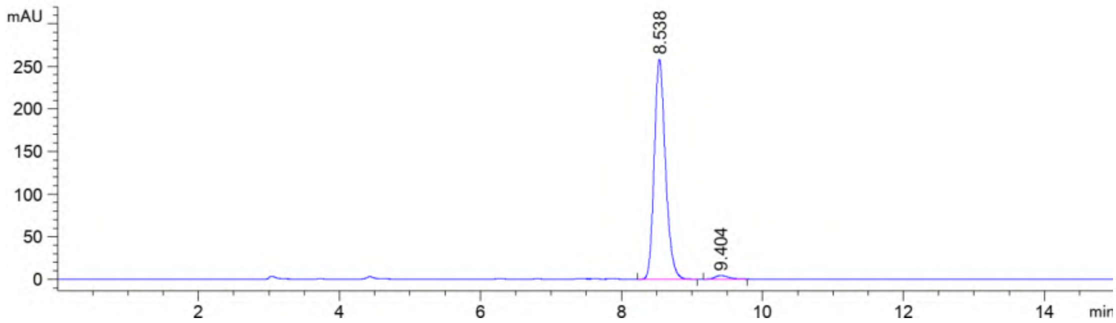

Signal 1: DAD1 D, Sig=230,4 Ref=360,100

Peak #	RetTime [min]	Type	Width [min]	Area [mAU*s]	Height [mAU]	Area %
1	8.538	BB	0.1686	2853.45093	257.77637	98.0139
2	9.404	BB	0.1579	57.81960	4.31225	1.9861

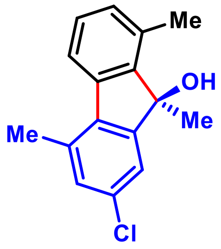

3va

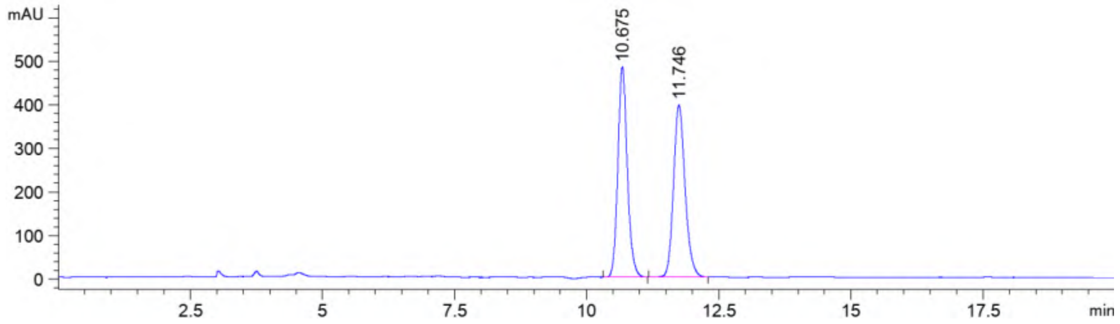

Signal 1: DAD1 B, Sig=220,4 Ref=off

Peak #	RetTime [min]	Type	Width [min]	Area [mAU*s]	Height [mAU]	Area %
1	10.675	BB	0.1907	5990.75342	482.84769	49.9141
2	11.746	BB	0.2343	6011.38477	395.55243	50.0859

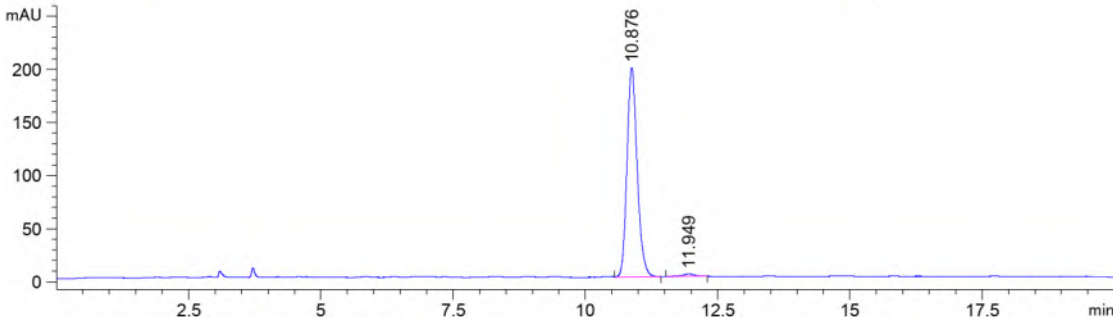

Signal 1: DAD1 B, Sig=220,4 Ref=off

Peak #	RetTime [min]	Type	Width [min]	Area [mAU*s]	Height [mAU]	Area %
1	10.876	BB	0.2050	2632.86670	196.90004	98.4983
2	11.949	BB	0.1948	40.14148	2.42308	1.5017

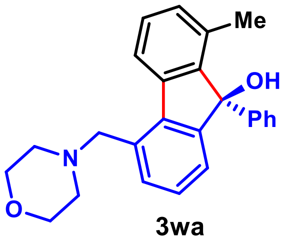

3wa

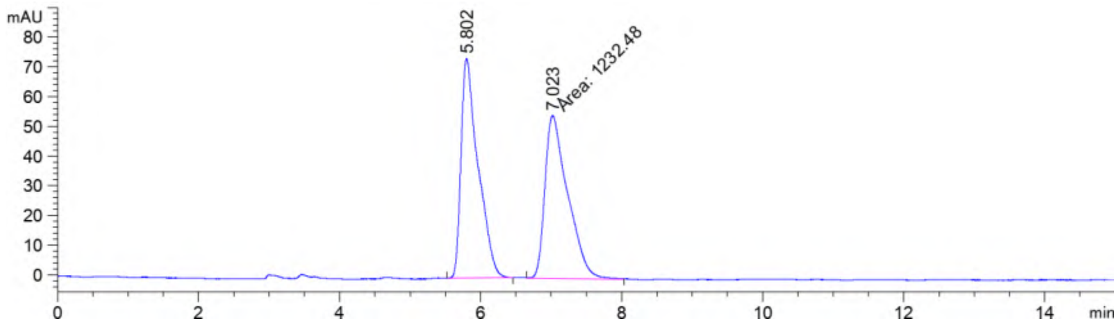

Signal 1: DAD1 D, Sig=230,4 Ref=360,100

Peak #	RetTime [min]	Type	Width [min]	Area [mAU*s]	Height [mAU]	Area %
1	5.802	BB	0.2339	1238.24878	73.93929	50.1167
2	7.023	MM	0.3748	1232.48267	54.80887	49.8833

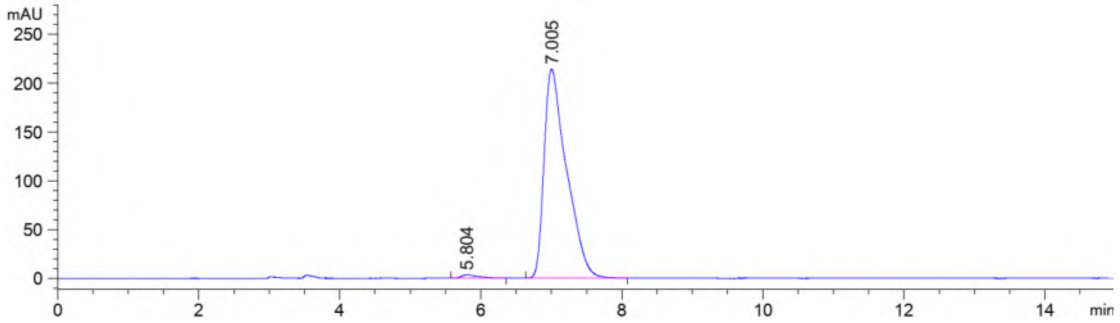

Signal 1: DAD1 D, Sig=230,4 Ref=360,100

Peak #	RetTime [min]	Type	Width [min]	Area [mAU*s]	Height [mAU]	Area %
1	5.804	BB	0.1980	56.96270	3.44421	1.1884
2	7.005	BB	0.3149	4736.25732	213.44194	98.8116

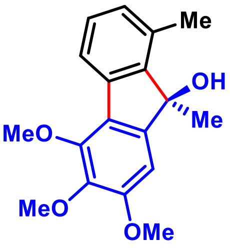

3xa

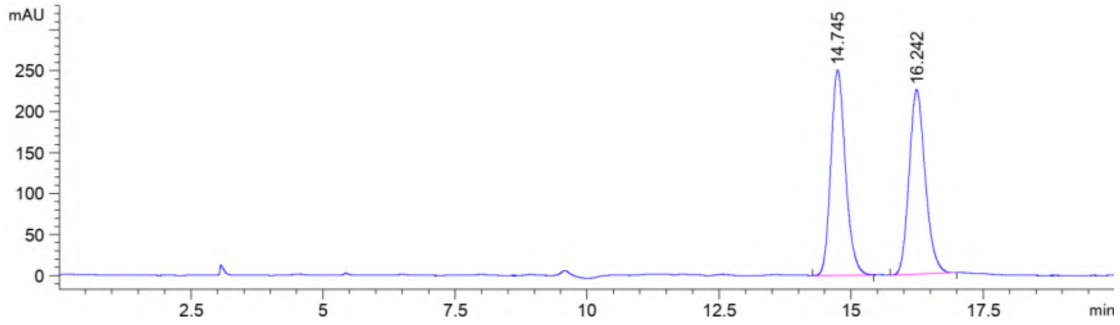

Signal 1: DAD1 B, Sig=220,4 Ref=off

Peak #	RetTime [min]	Type	Width [min]	Area [mAU*s]	Height [mAU]	Area %
1	14.745	BB	0.2863	4830.27197	250.72440	50.2363
2	16.242	BB	0.3105	4784.82861	225.50558	49.7637

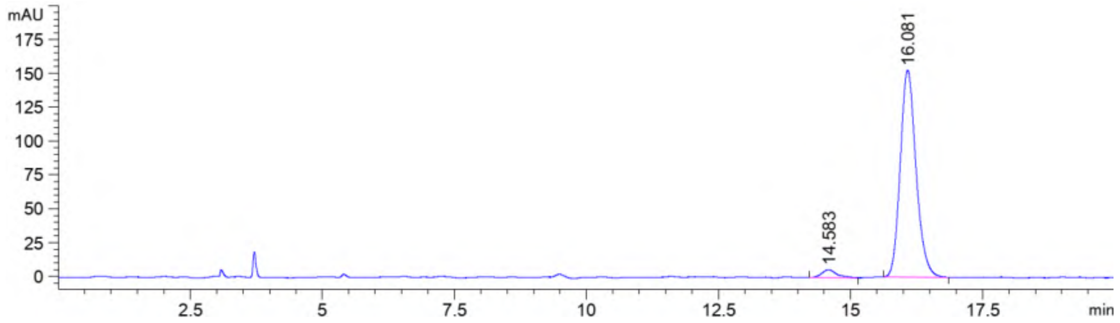

Signal 1: DAD1 B, Sig=220,4 Ref=off

Peak #	RetTime [min]	Type	Width [min]	Area [mAU*s]	Height [mAU]	Area %
1	14.583	BB	0.2298	113.96405	5.83568	3.4628
2	16.081	BB	0.3046	3177.13647	152.76363	96.5372

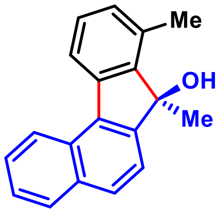

3ya

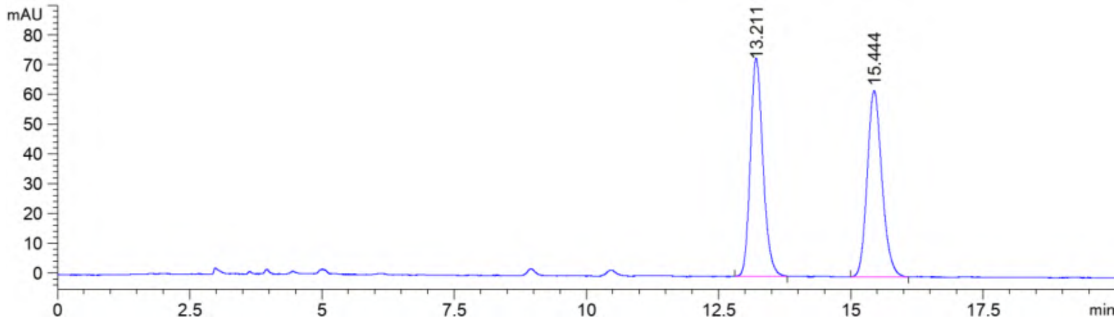

Signal 1: DAD1 D, Sig=230,4 Ref=360,100

Peak #	RetTime [min]	Type	Width [min]	Area [mAU*s]	Height [mAU]	Area %
1	13.211	BB	0.2295	1212.39331	73.27695	49.9546
2	15.444	BB	0.2456	1214.59607	62.66016	50.0454

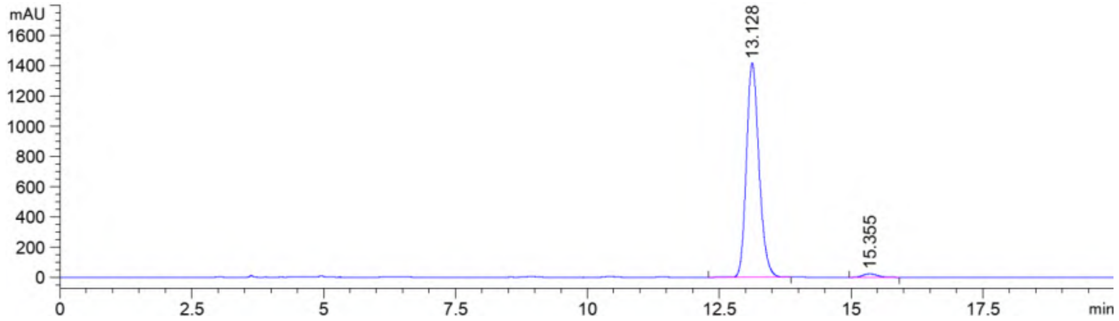

Signal 1: DAD1 D, Sig=230,4 Ref=360,100

Peak #	RetTime [min]	Type	Width [min]	Area [mAU*s]	Height [mAU]	Area %
1	13.128	VB R	0.2306	2.35635e4	1414.83533	98.1680
2	15.355	BV R	0.2262	439.74475	22.88313	1.8320

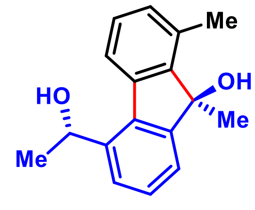

(*R*,*S*)-**3za**

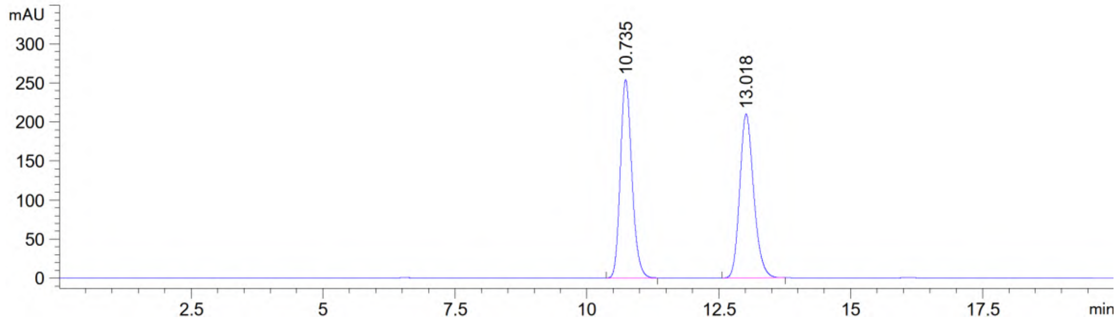

Signal 1: DAD1 F, Sig=290,4 Ref=360,100

Peak #	RetTime [min]	Type	Width [min]	Area [mAU*s]	Height [mAU]	Area %
1	10.735	BB	0.2312	3860.35742	254.13708	49.9537
2	13.018	BB	0.2754	3867.50928	209.98126	50.0463

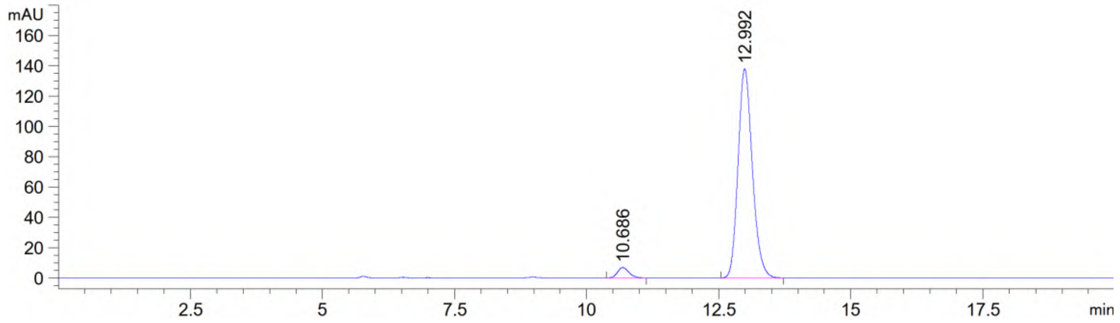

Signal 1: DAD1 F, Sig=290,4 Ref=360,100

Peak #	RetTime [min]	Type	Width [min]	Area [mAU*s]	Height [mAU]	Area %
1	10.686	BB	0.1830	104.72060	6.94076	3.8774
2	12.992	BB	0.2821	2596.08691	137.89778	96.1226

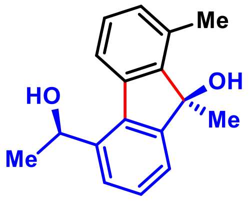

(*S*,*S*)-**3za**

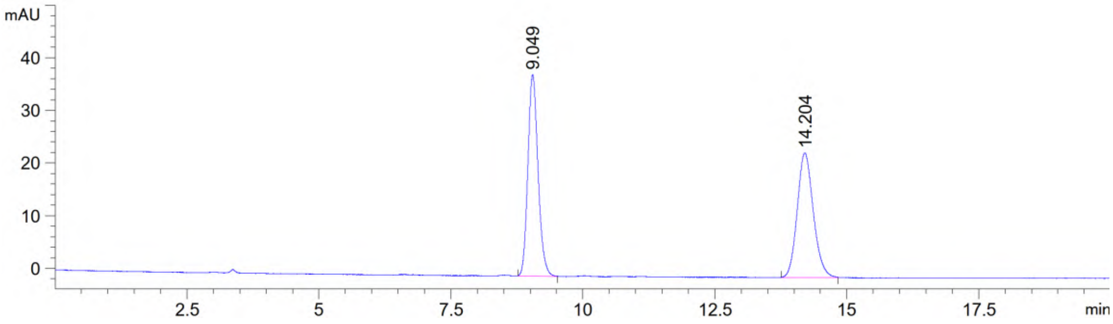

Signal 1: DAD1 F, Sig=290,4 Ref=360,100

Peak #	RetTime [min]	Type	Width [min]	Area [mAU*s]	Height [mAU]	Area %
1	9.049	BB	0.1890	511.00177	38.21356	50.5406
2	14.204	BB	0.2480	500.07068	23.66797	49.4594

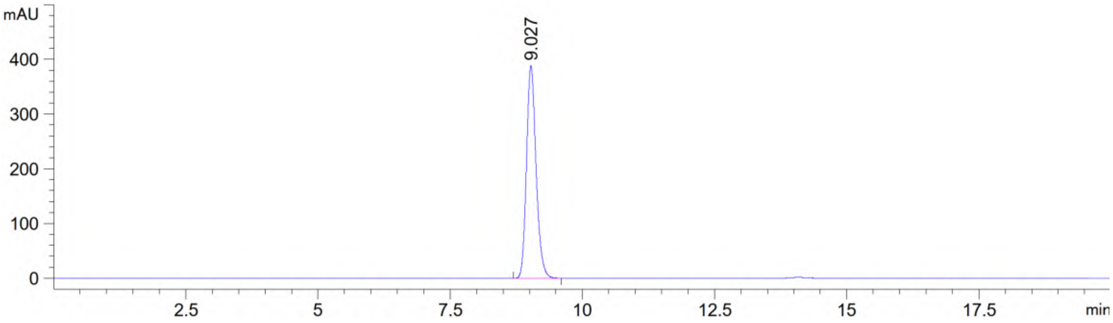

Signal 1: DAD1 F, Sig=290,4 Ref=360,100

Peak #	RetTime [min]	Type	Width [min]	Area [mAU*s]	Height [mAU]	Area %
1	9.027	BB	0.1924	4889.17480	388.30212	100.0000

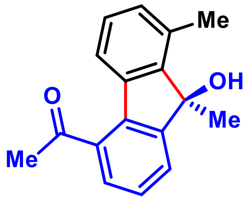

3Aa

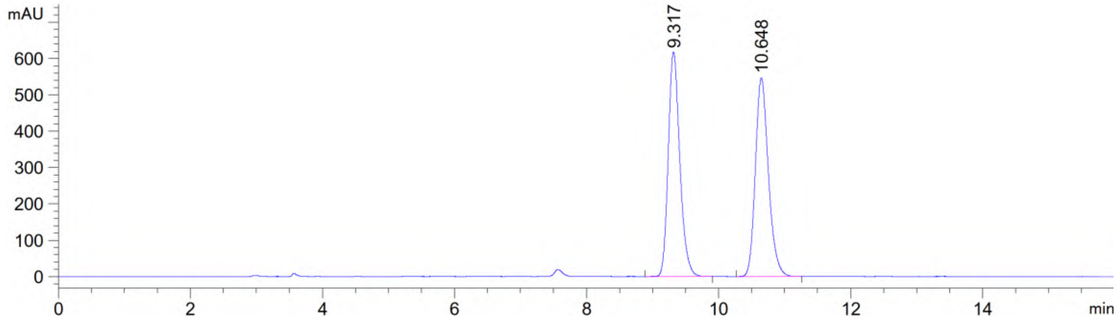

Signal 1: DAD1 D, Sig=230,4 Ref=360,100

Peak #	RetTime [min]	Type	Width [min]	Area [mAU*s]	Height [mAU]	Area %
1	9.317	VB R	0.1830	7505.68652	618.65619	49.9935
2	10.648	BB	0.2087	7507.63428	546.76013	50.0065

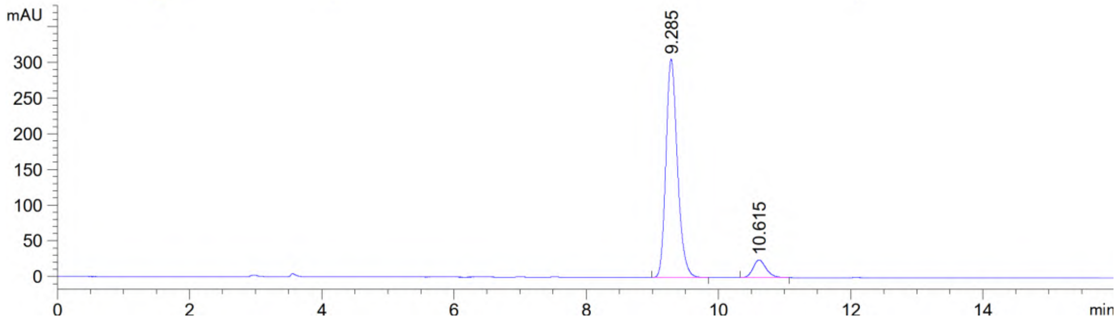

Signal 1: DAD1 D, Sig=230,4 Ref=360,100

Peak #	RetTime [min]	Type	Width [min]	Area [mAU*s]	Height [mAU]	Area %
1	9.285	BB	0.1828	3689.10229	305.69308	91.6189
2	10.615	BB	0.1840	337.46976	24.68760	8.3811

10. NMR Spectra of the new compounds

^{1}H NMR (400 MHz, $CDCl_3$)

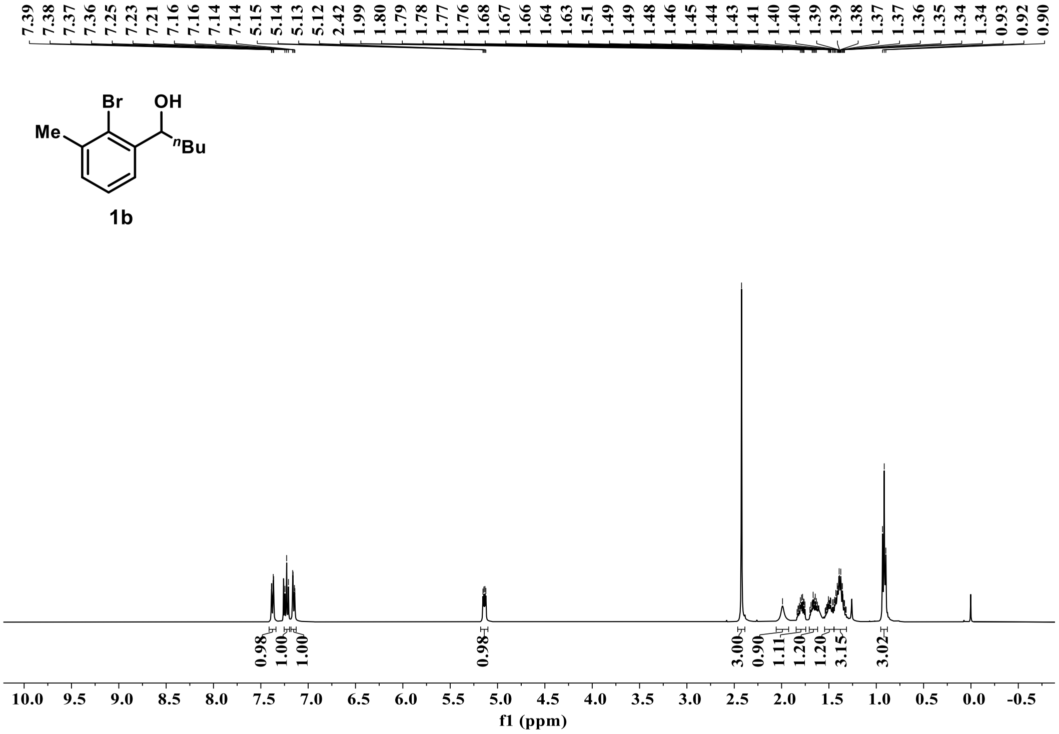

^{13}C NMR (100 MHz, $CDCl_3$)

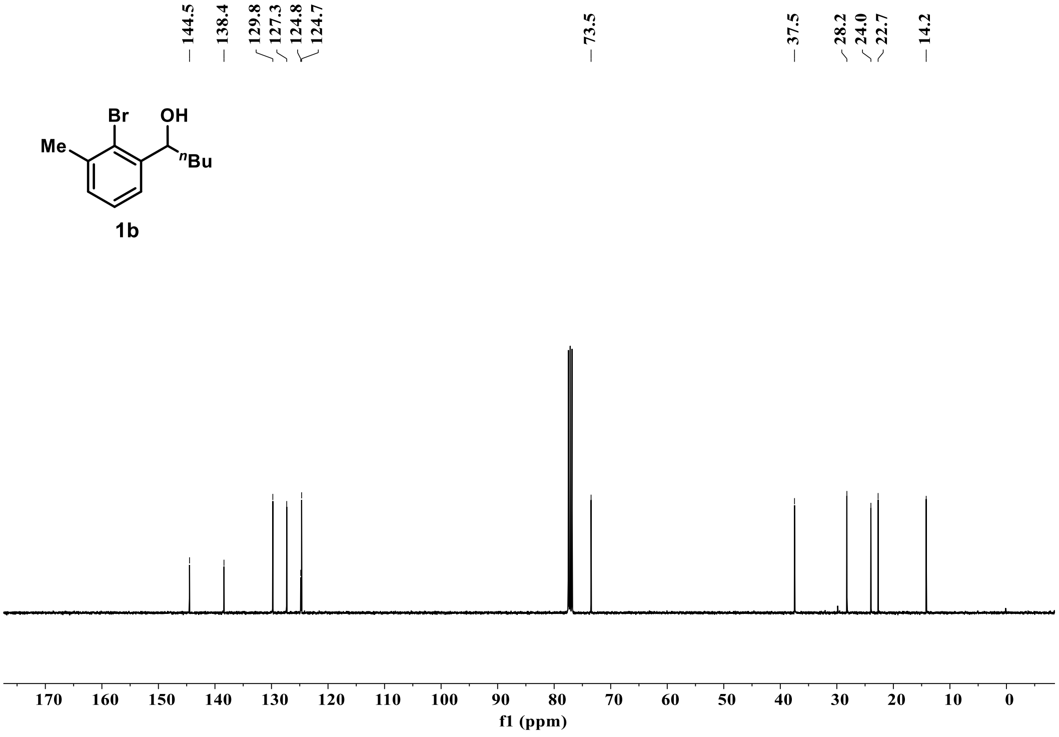

^{1}H NMR (400 MHz, $CDCl_3$)

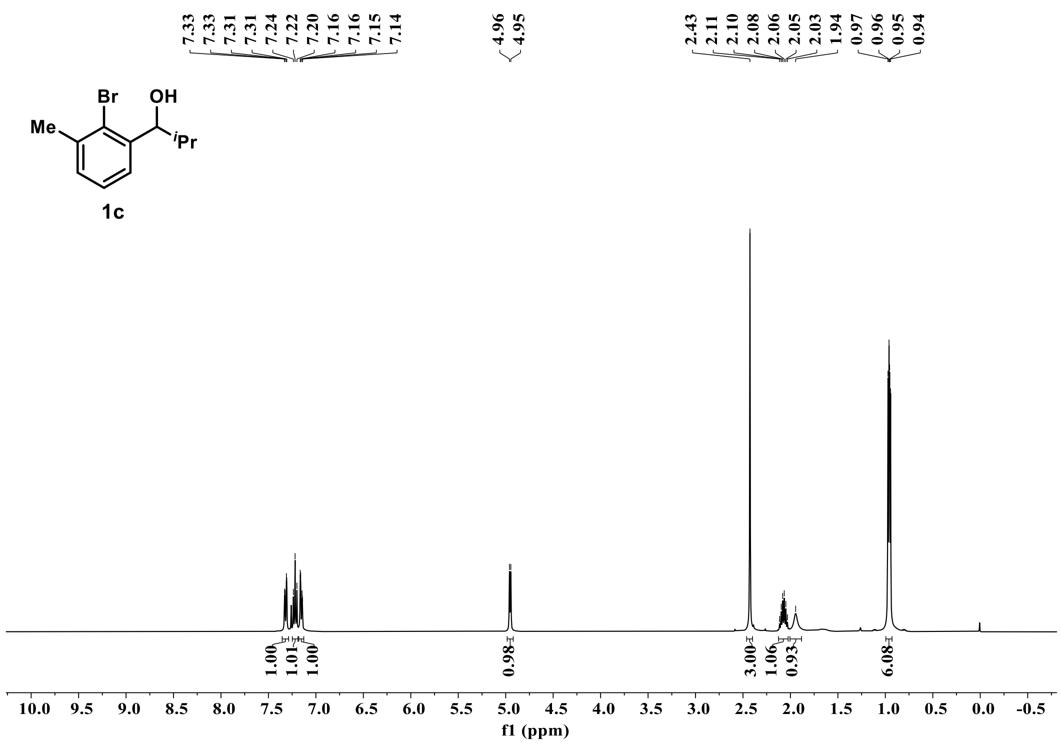

^{13}C NMR (100 MHz, $CDCl_3$)

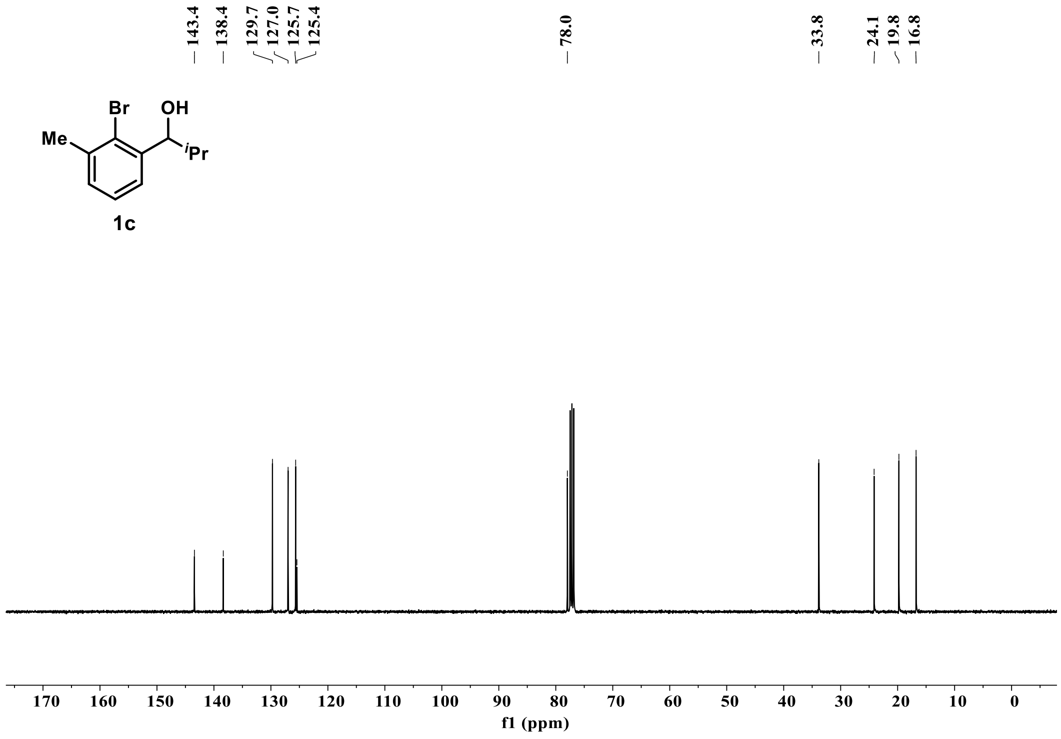

^{1}H NMR (400 MHz, $CDCl_3$)

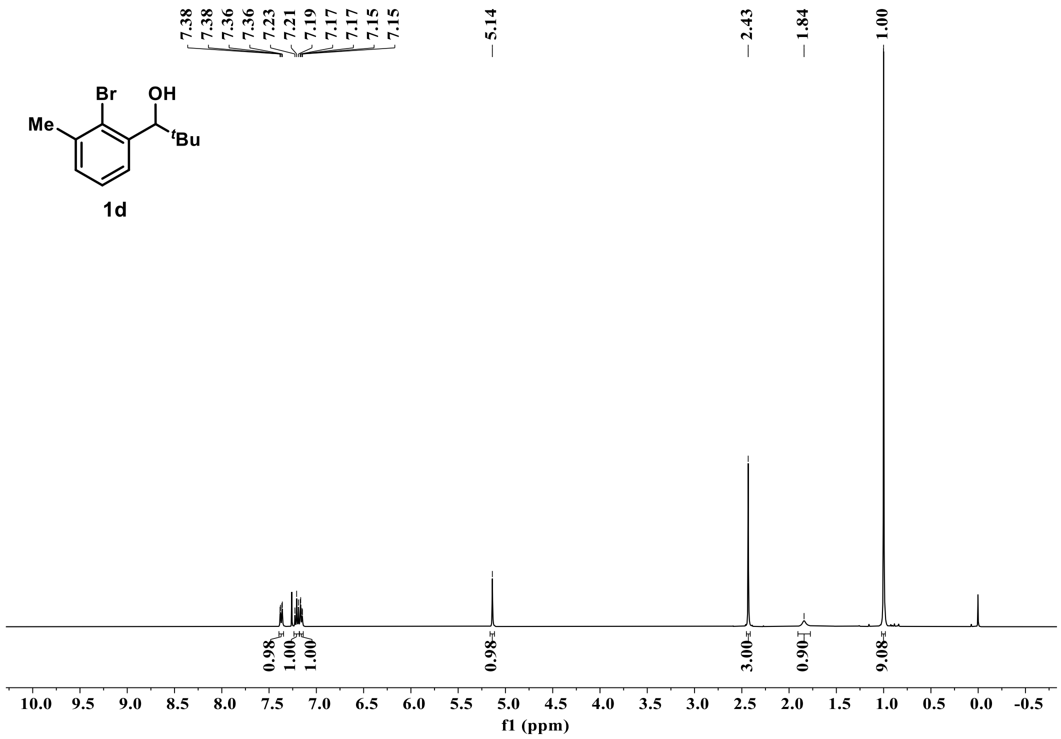

^{13}C NMR (100 MHz, $CDCl_3$)

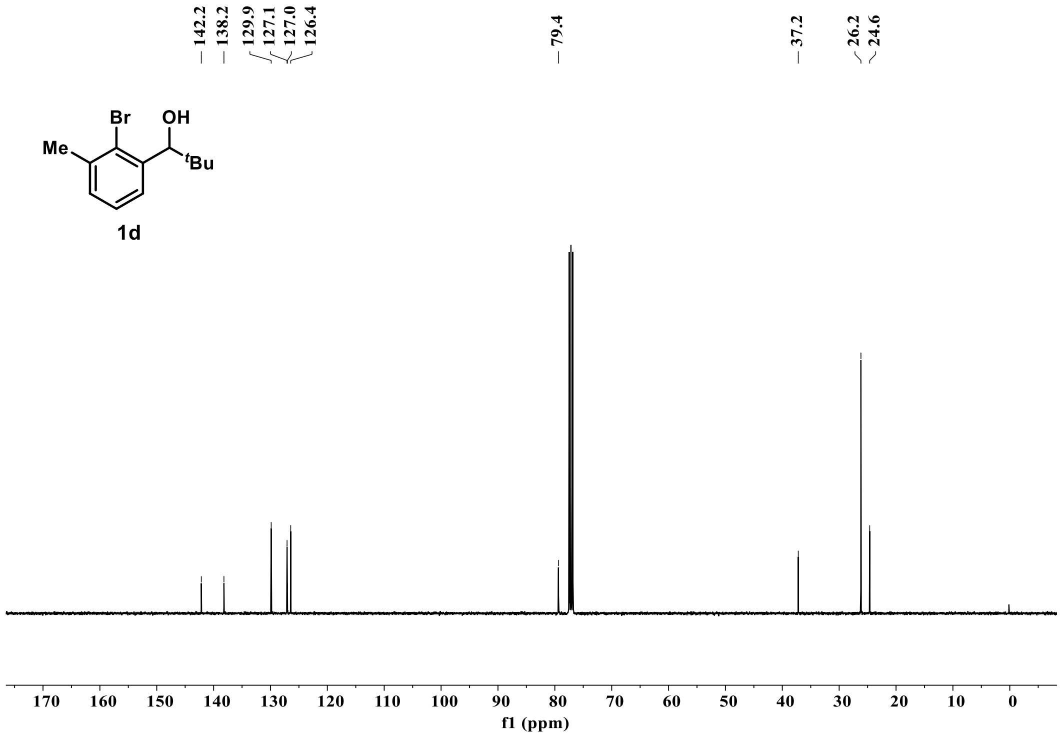

^{1}H NMR (400 MHz, CDCl3)

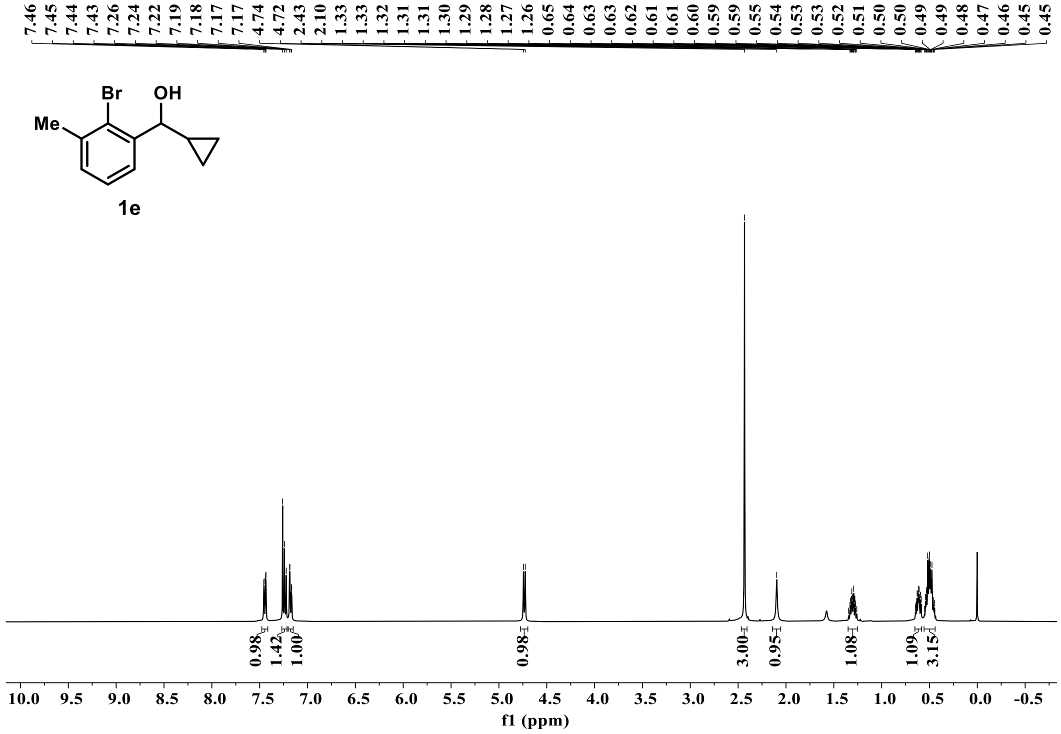

^{13}C NMR (100 MHz, CDCl3)

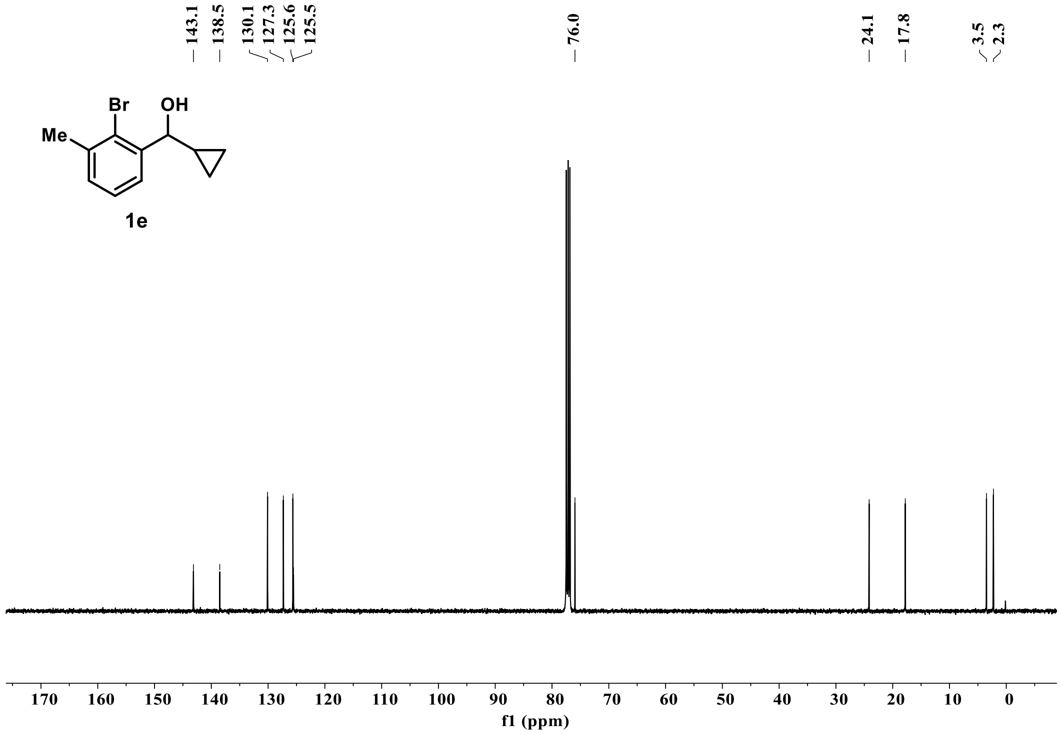

^{1}H NMR (400 MHz, $CDCl_3$)

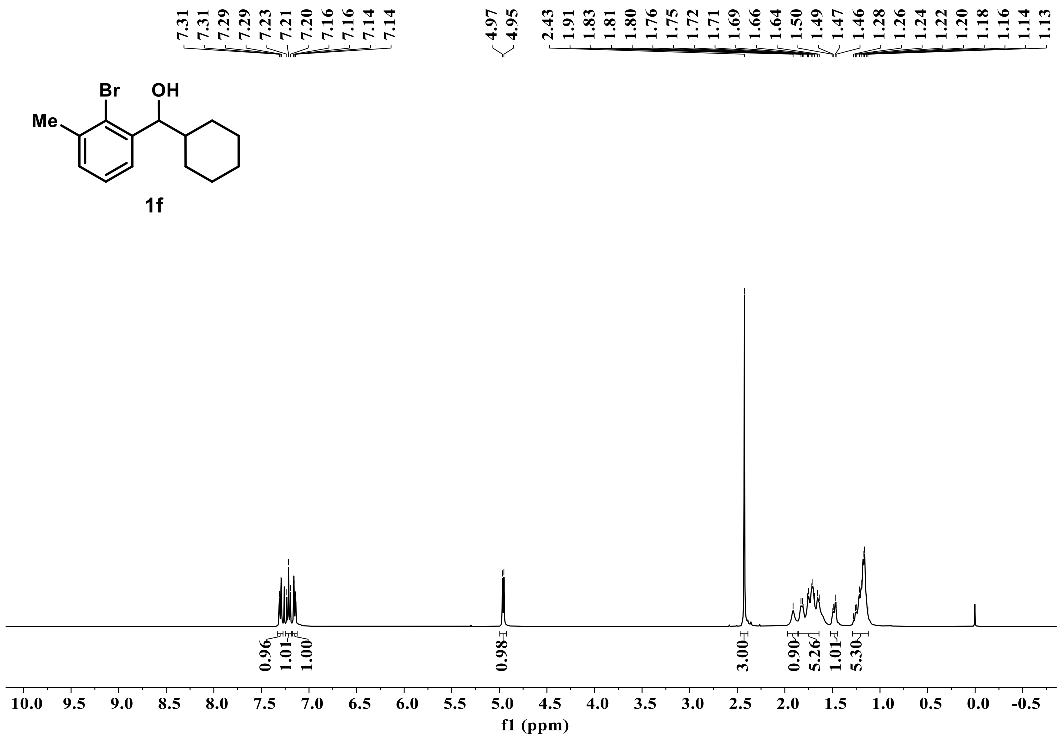

^{13}C NMR (100 MHz, $CDCl_3$)

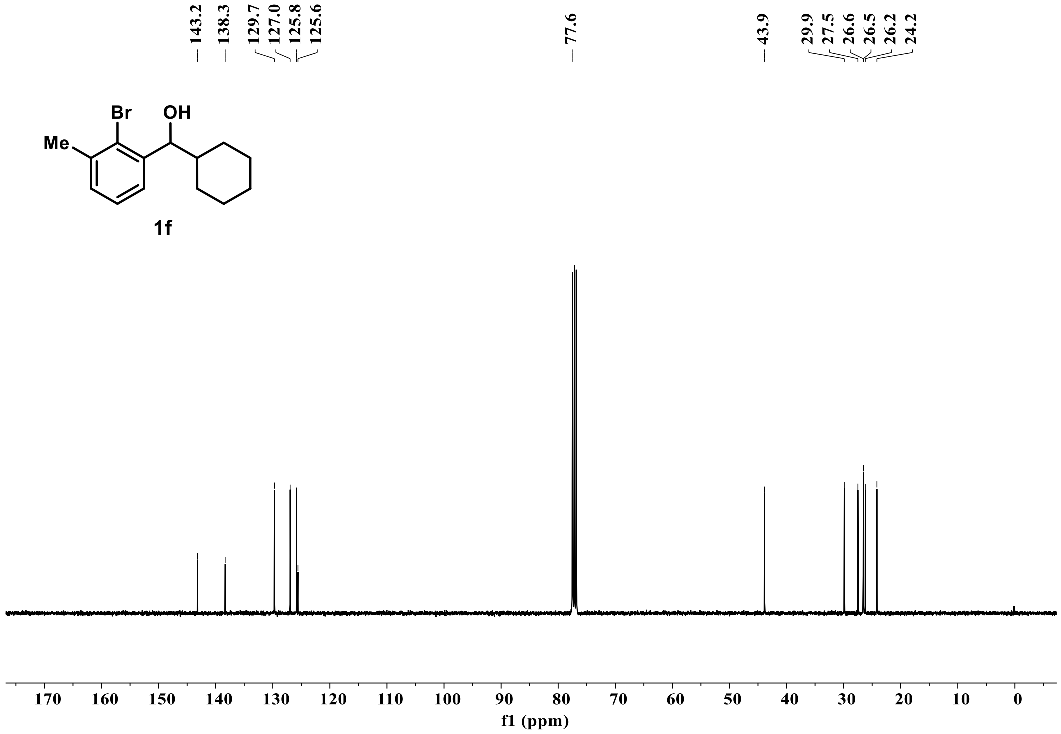

^{1}H NMR (400 MHz, $CDCl_3$)

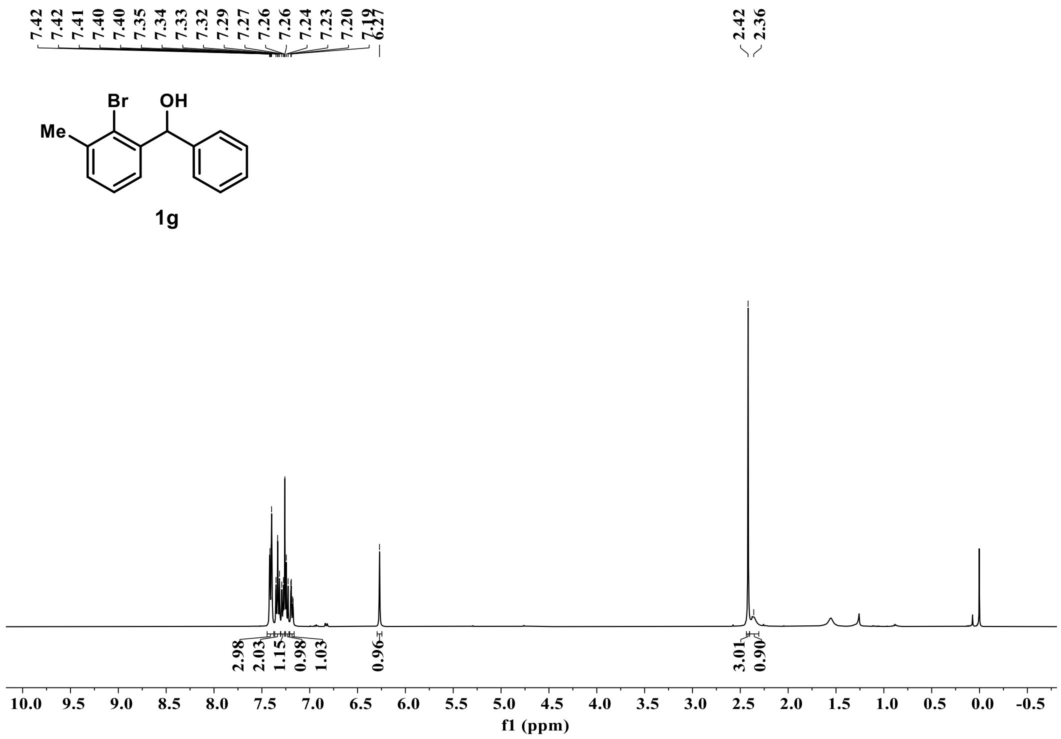

^{13}C NMR (100 MHz, $CDCl_3$)

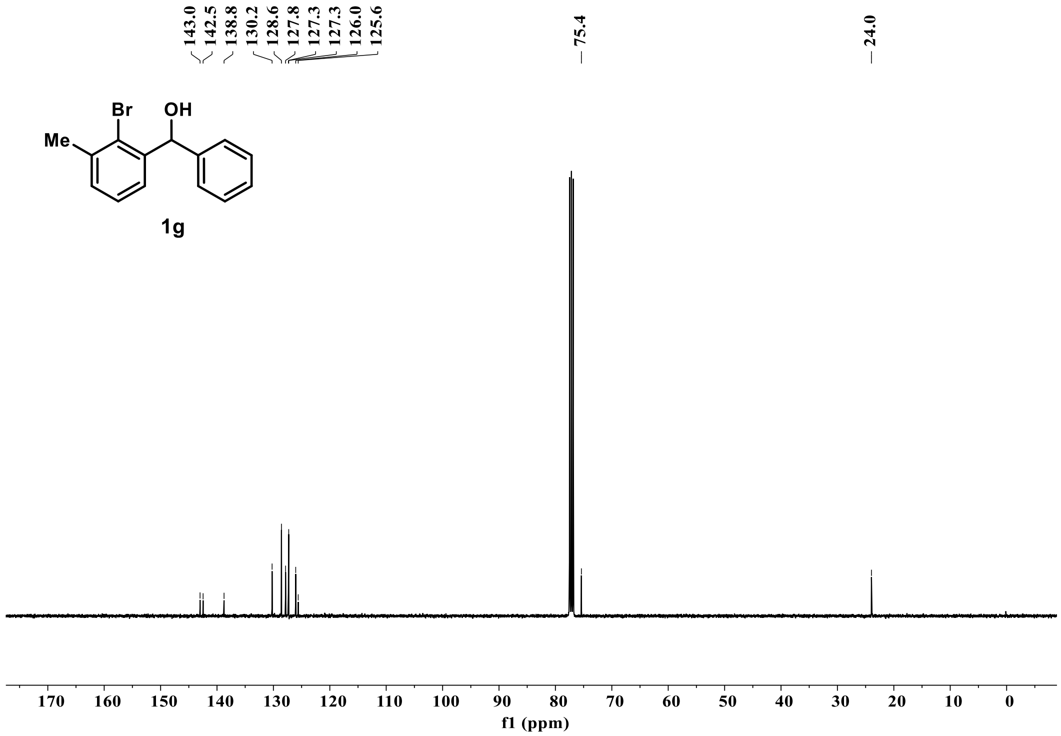

^{1}H NMR (400 MHz, $CDCl_3$)

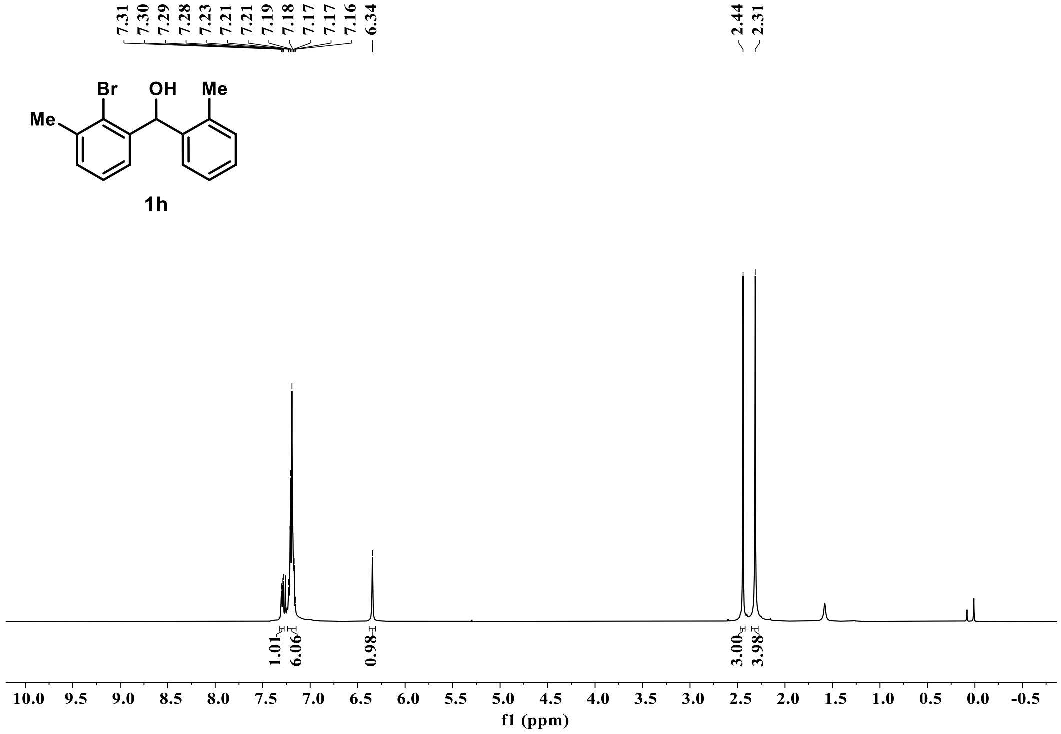

^{13}C NMR (100 MHz, $CDCl_3$)

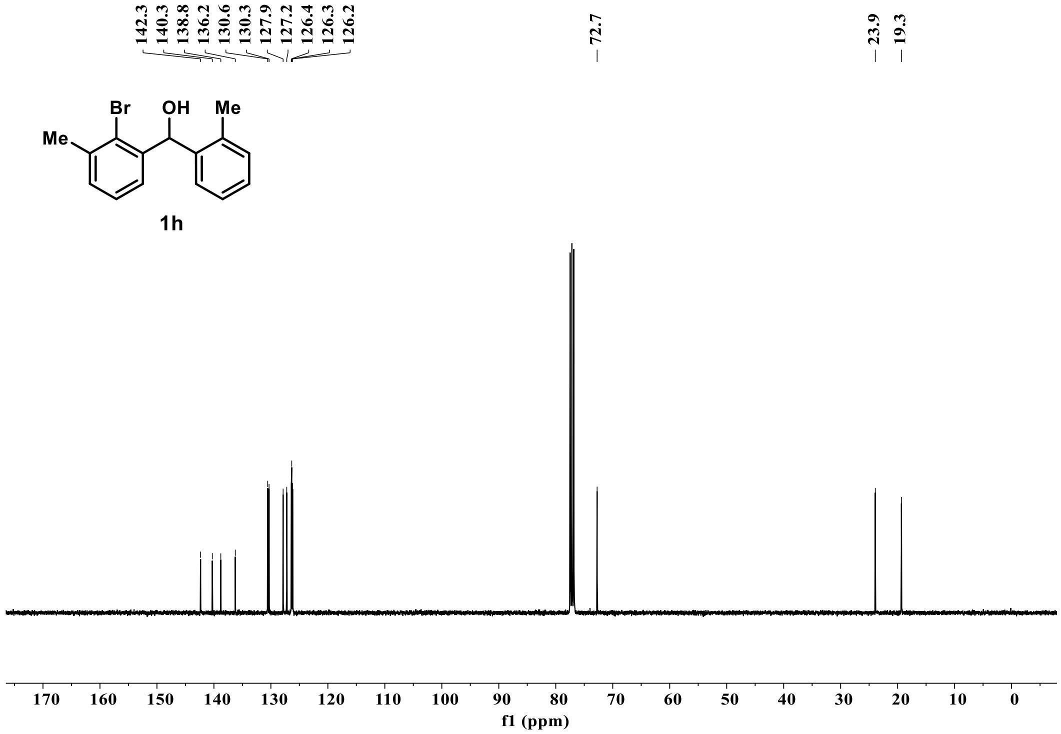

^{1}H NMR (400 MHz, $CDCl_3$)

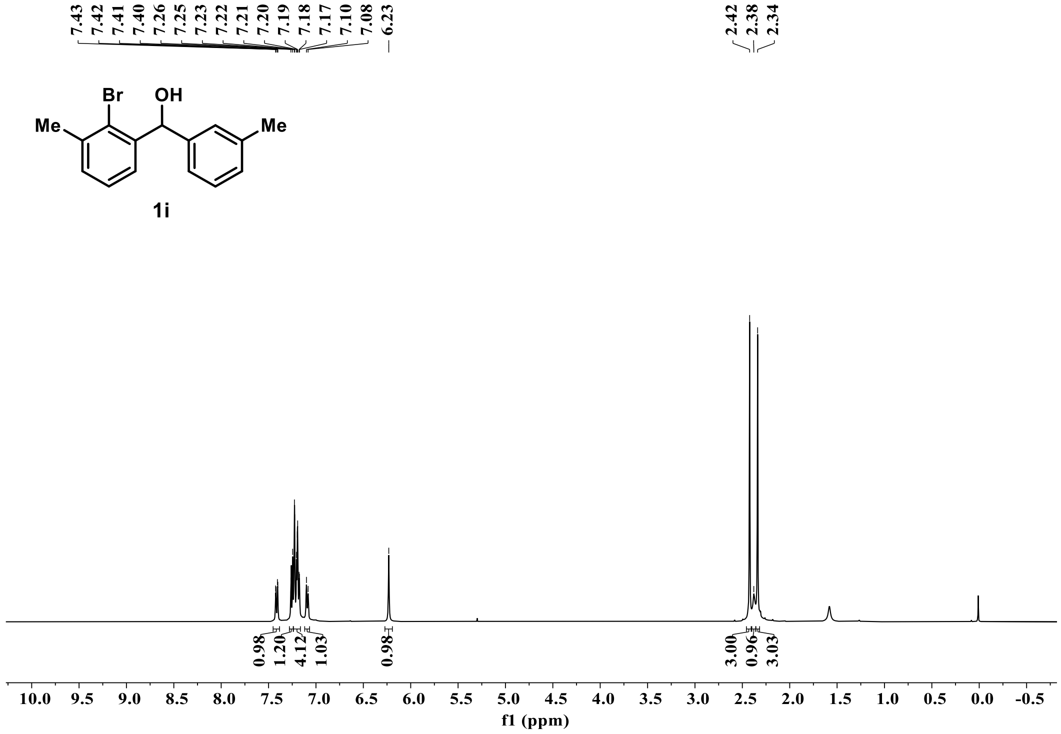

^{13}C NMR (100 MHz, $CDCl_3$)

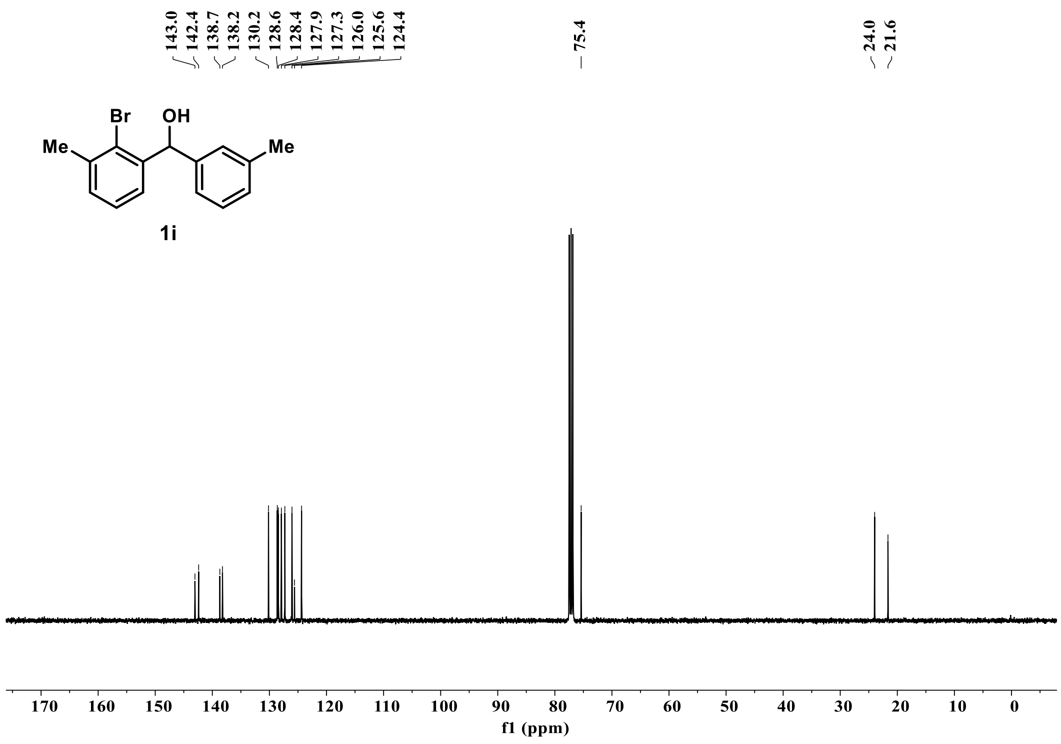

^{1}H NMR (400 MHz, $CDCl_3$)

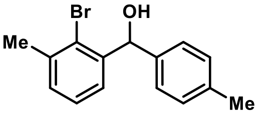

1j

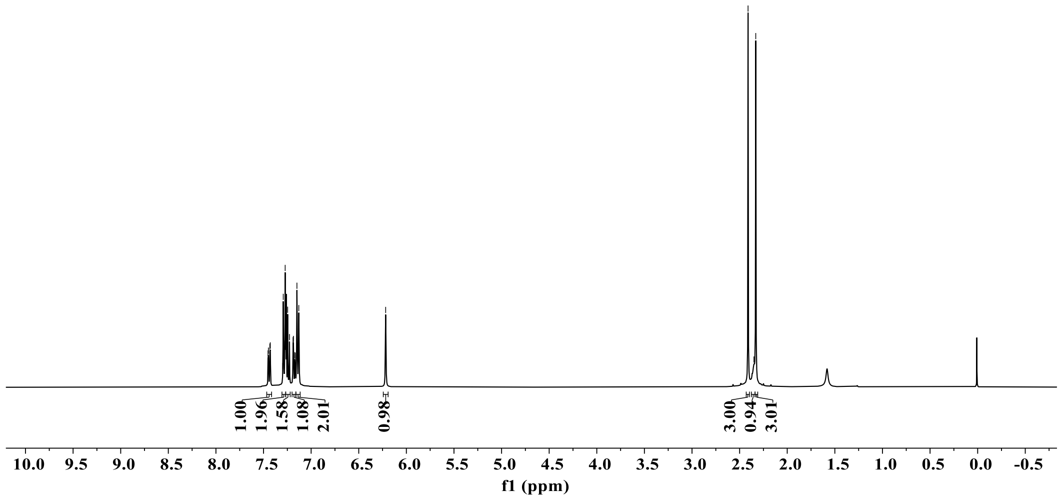

^{13}C NMR (100 MHz, $CDCl_3$)

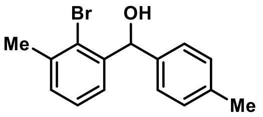

1j

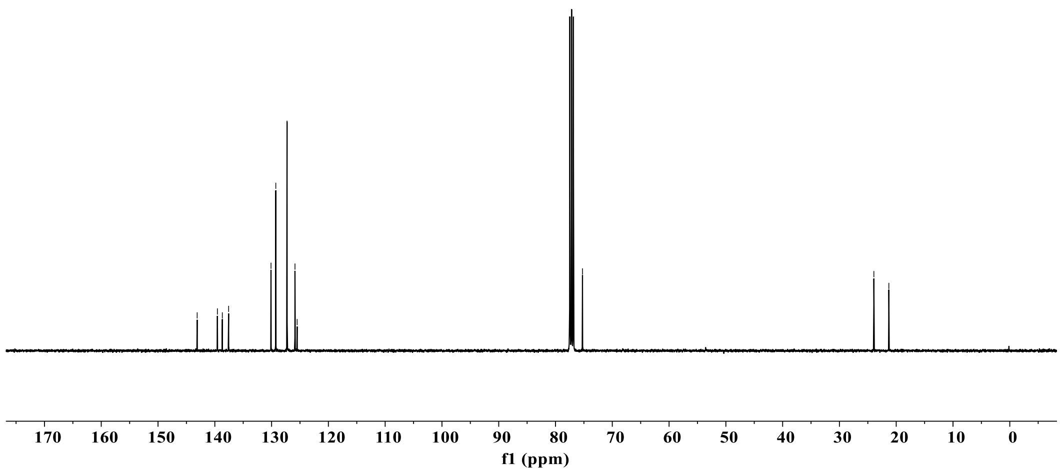

^{1}H NMR (400 MHz, $CDCl_3$)

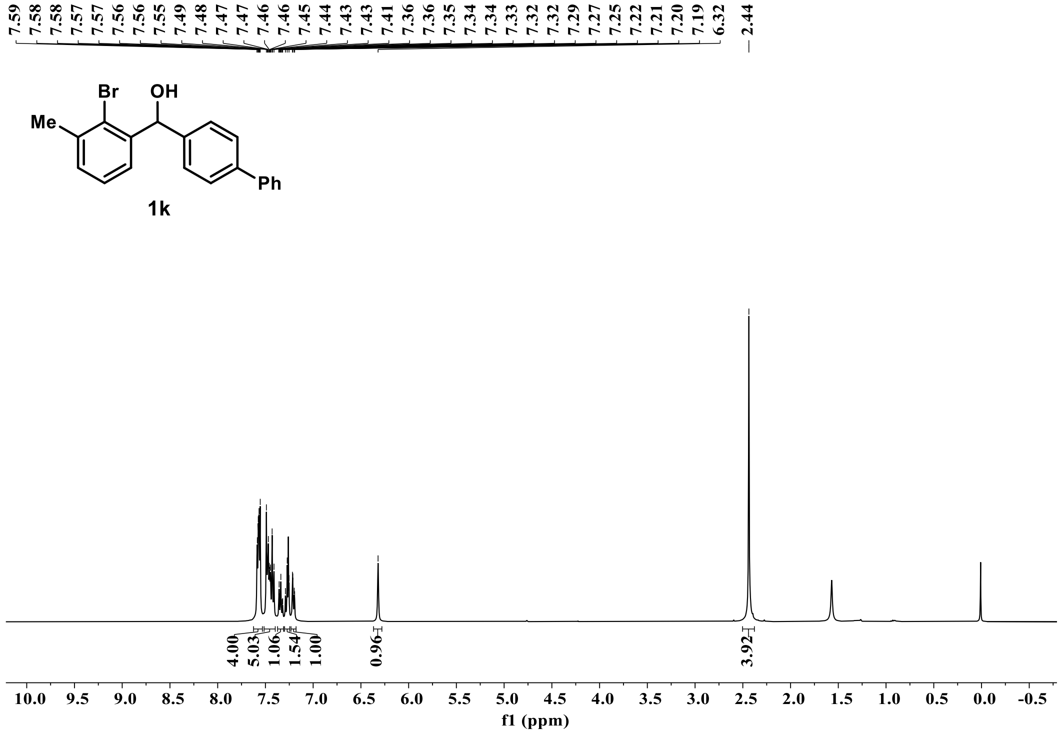

^{13}C NMR (100 MHz, $CDCl_3$)

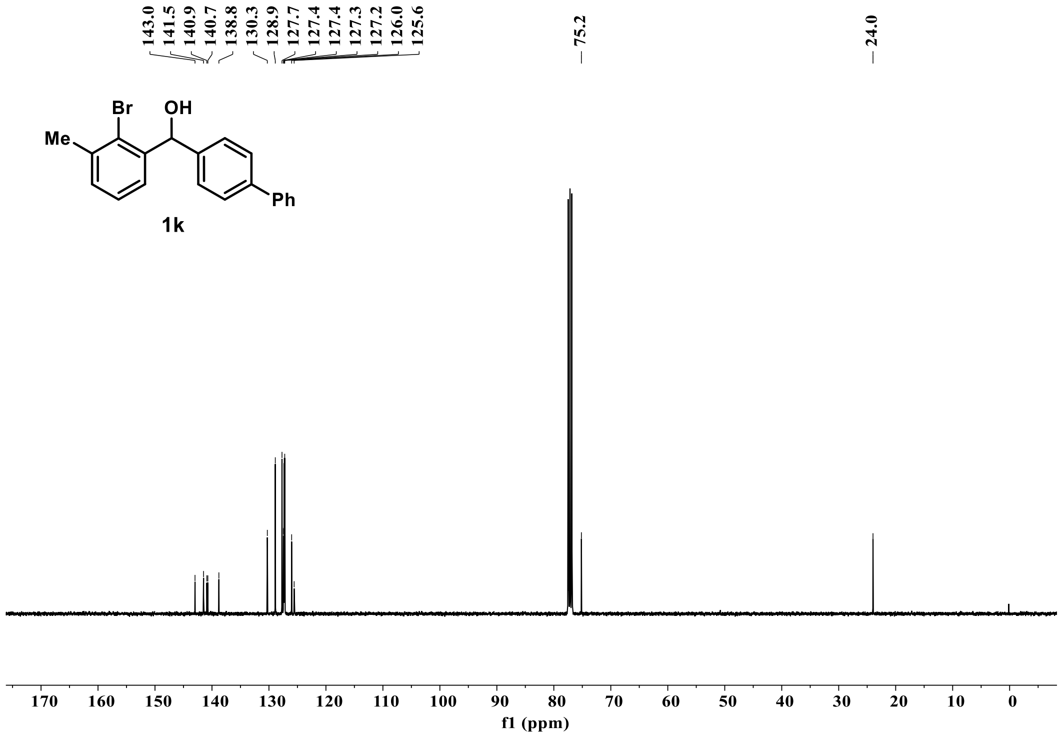

^{1}H NMR (400 MHz, $CDCl_3$)

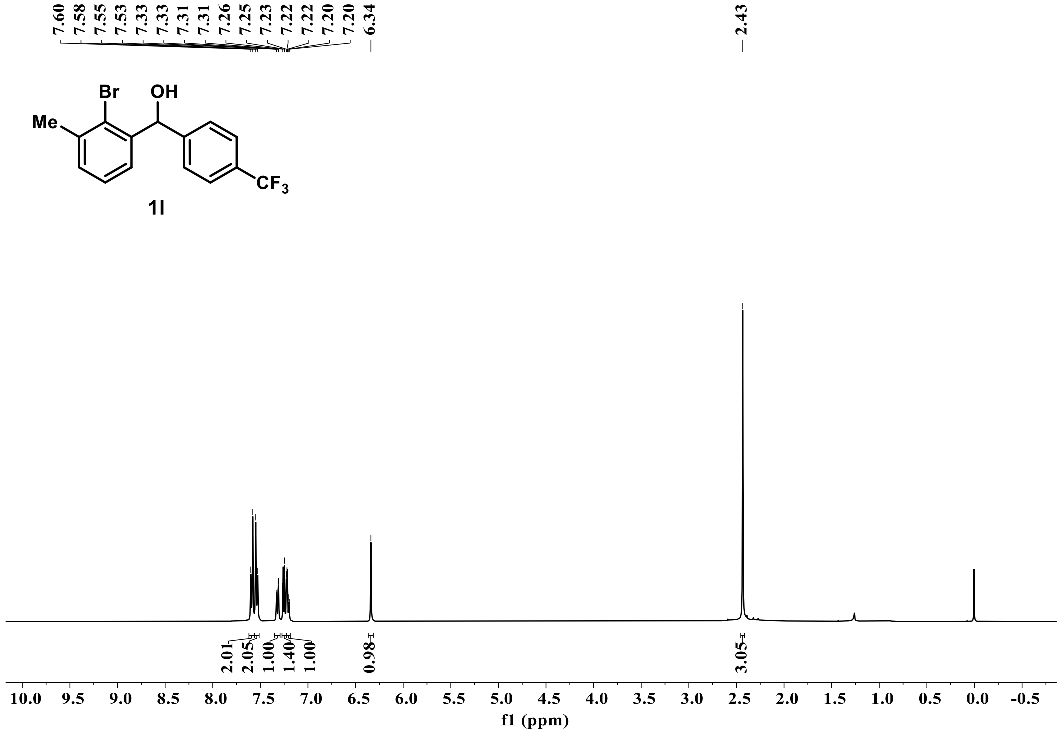

^{13}C NMR (100 MHz, $CDCl_3$)

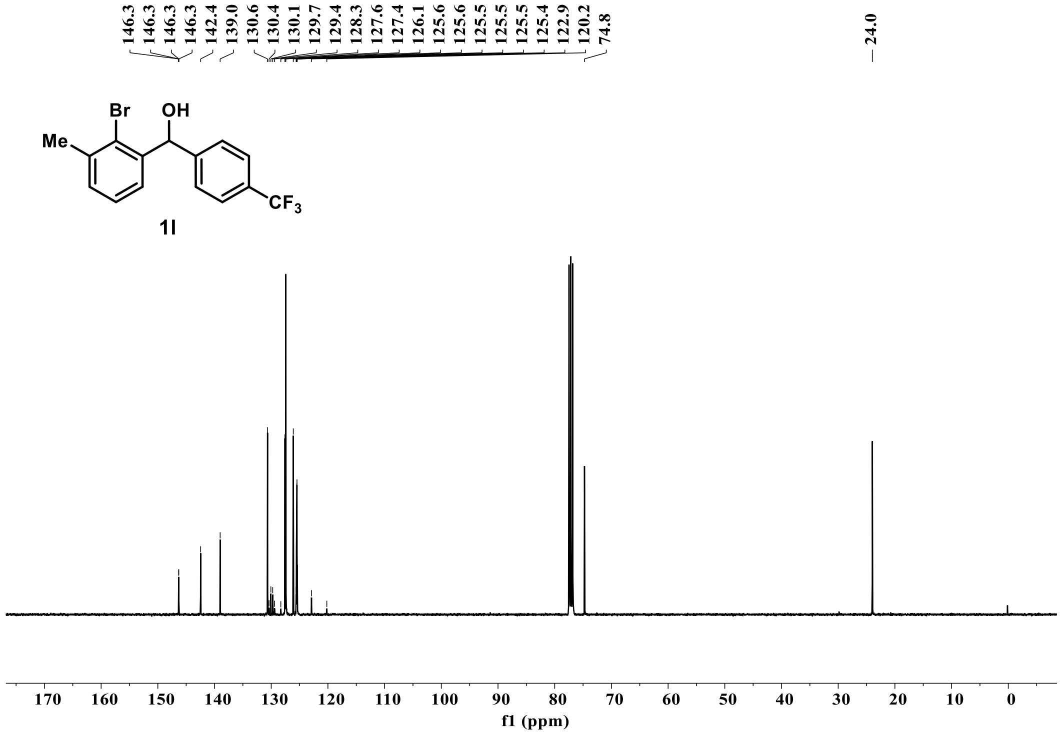

^{19}F NMR (376 MHz, $CDCl_3$)

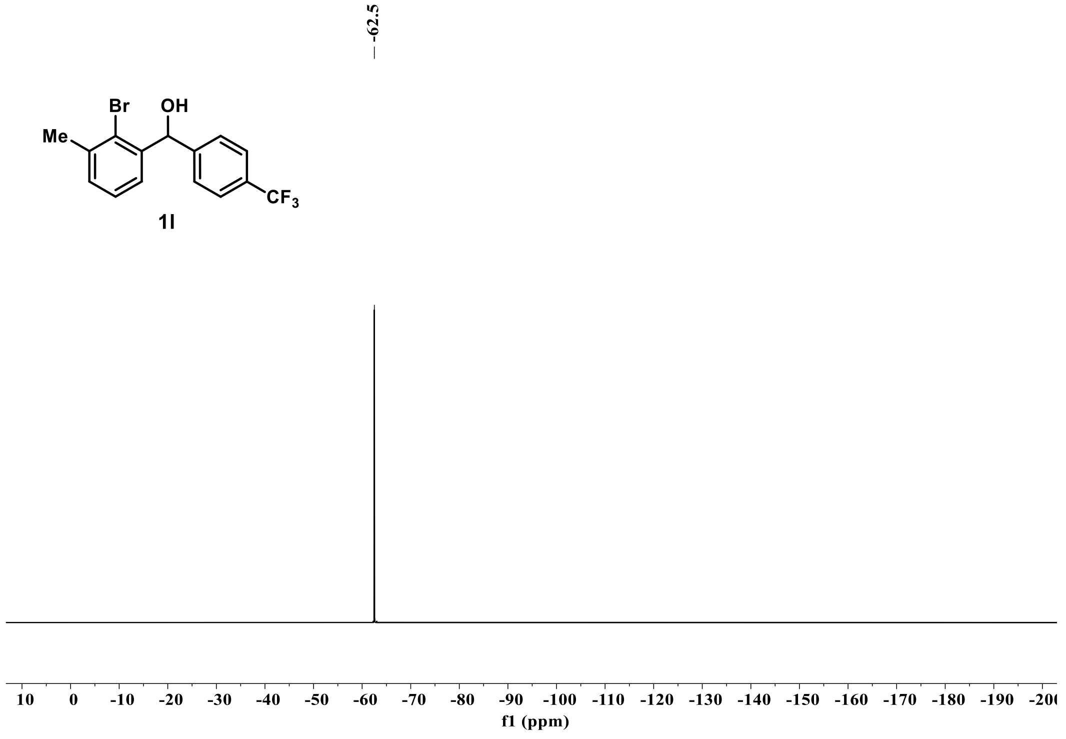

^{1}H NMR (400 MHz, $CDCl_3$)

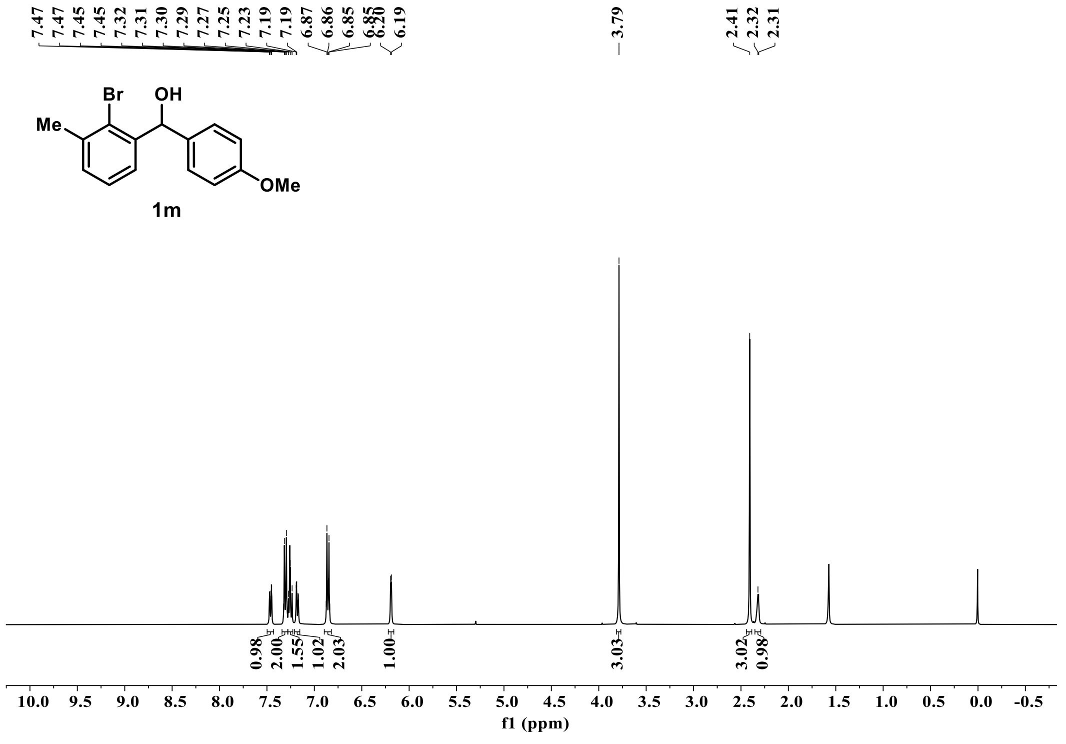

^{13}C NMR (100 MHz, $CDCl_3$)

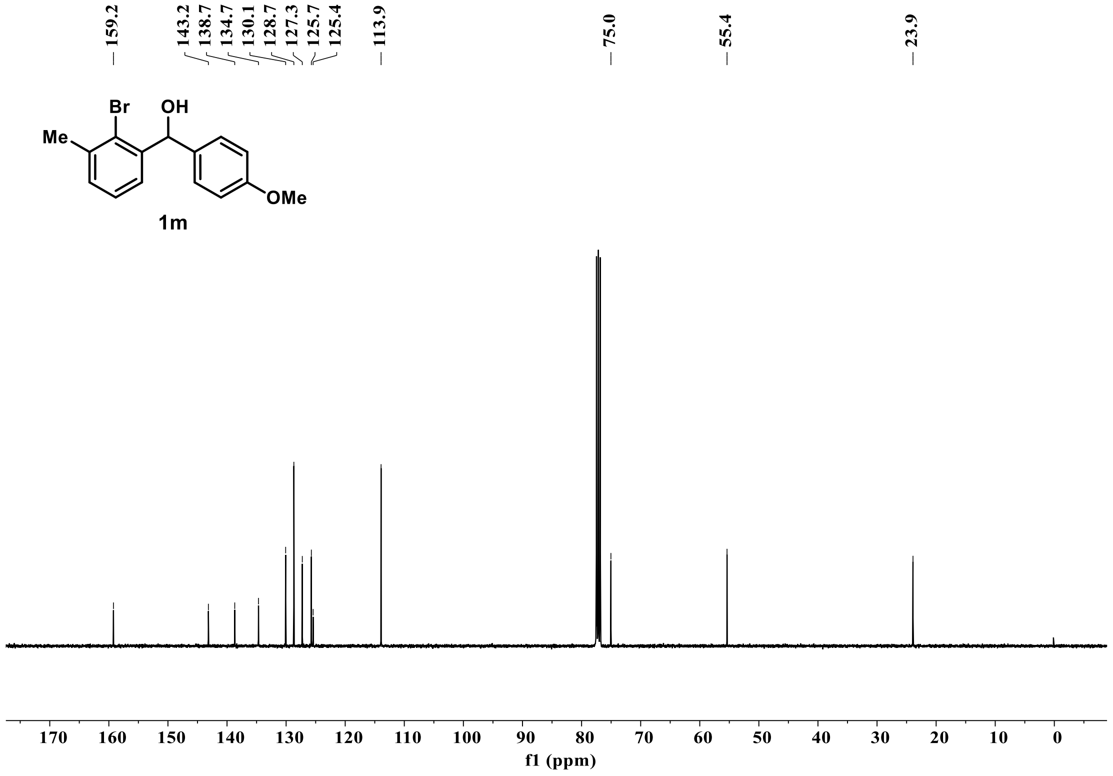

^{1}H NMR (400 MHz, $CDCl_3$)

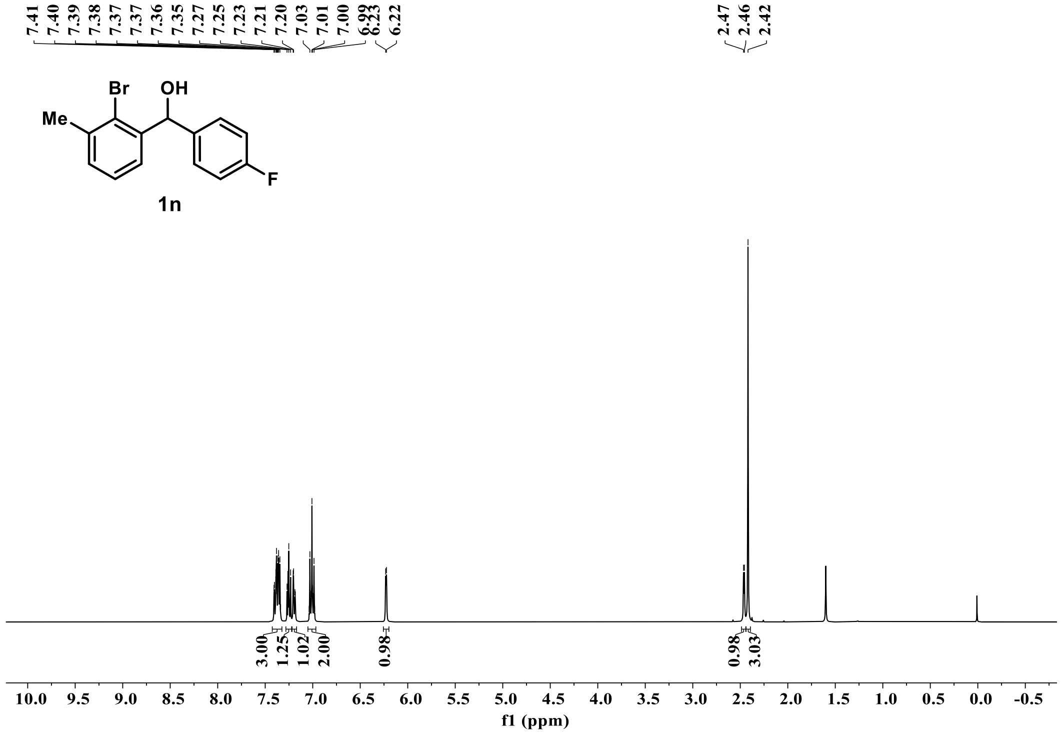

^{13}C NMR (100 MHz, $CDCl_3$)

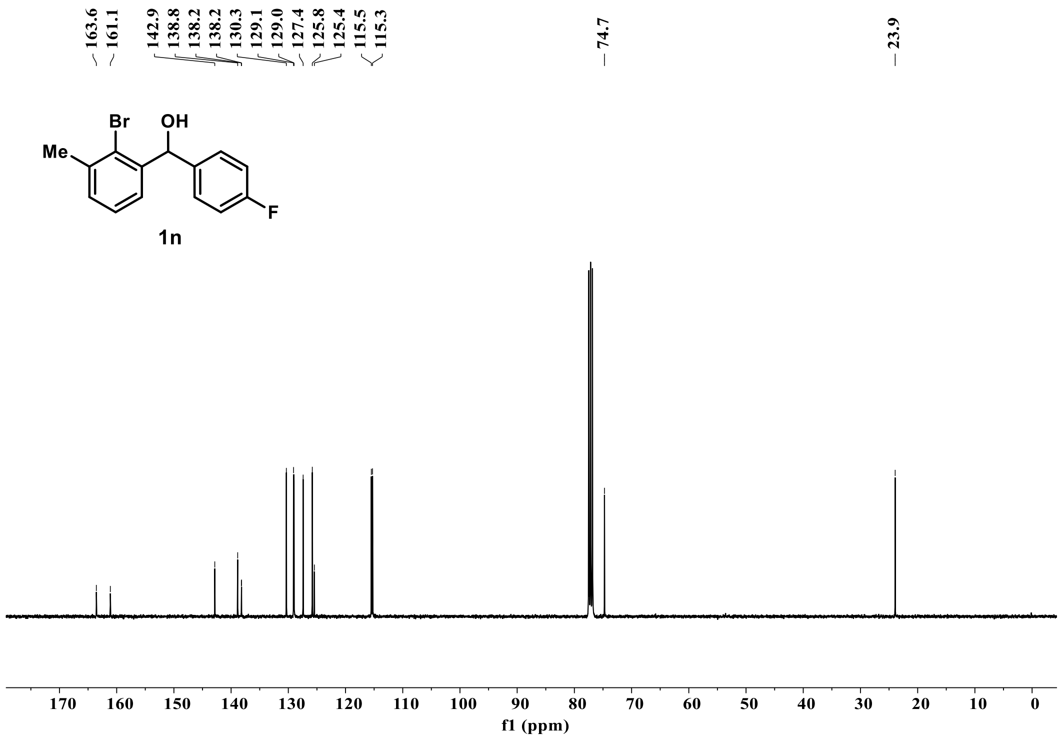

^{19}F NMR (376 MHz, $CDCl_3$)

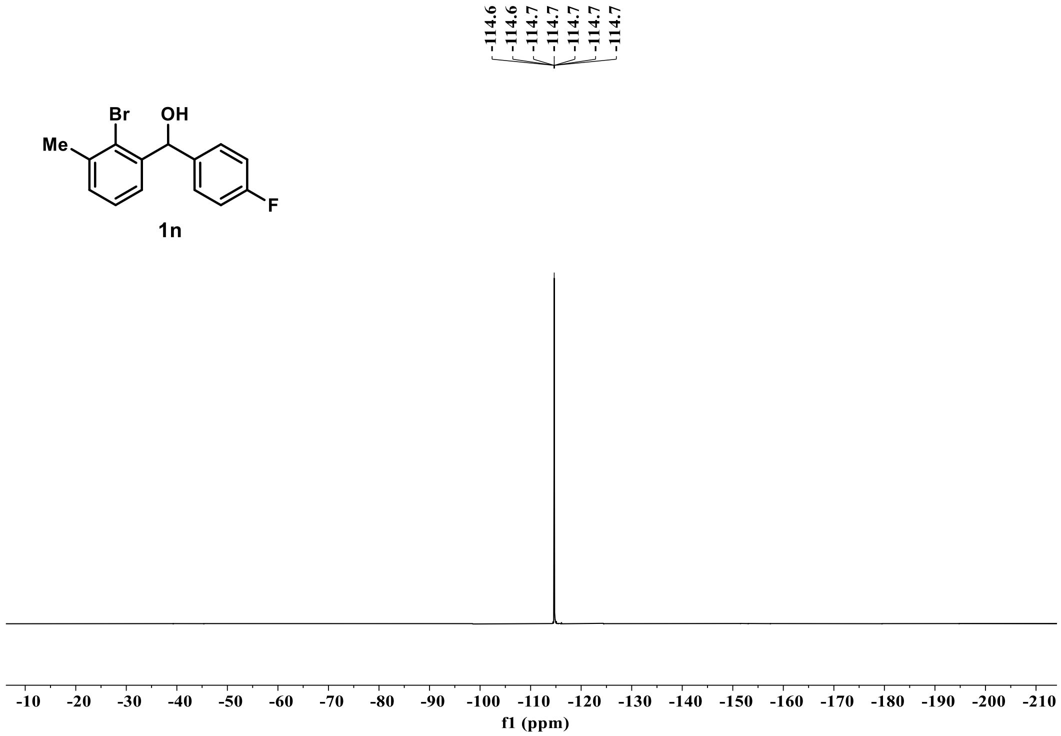

^{1}H NMR (400 MHz, $CDCl_3$)

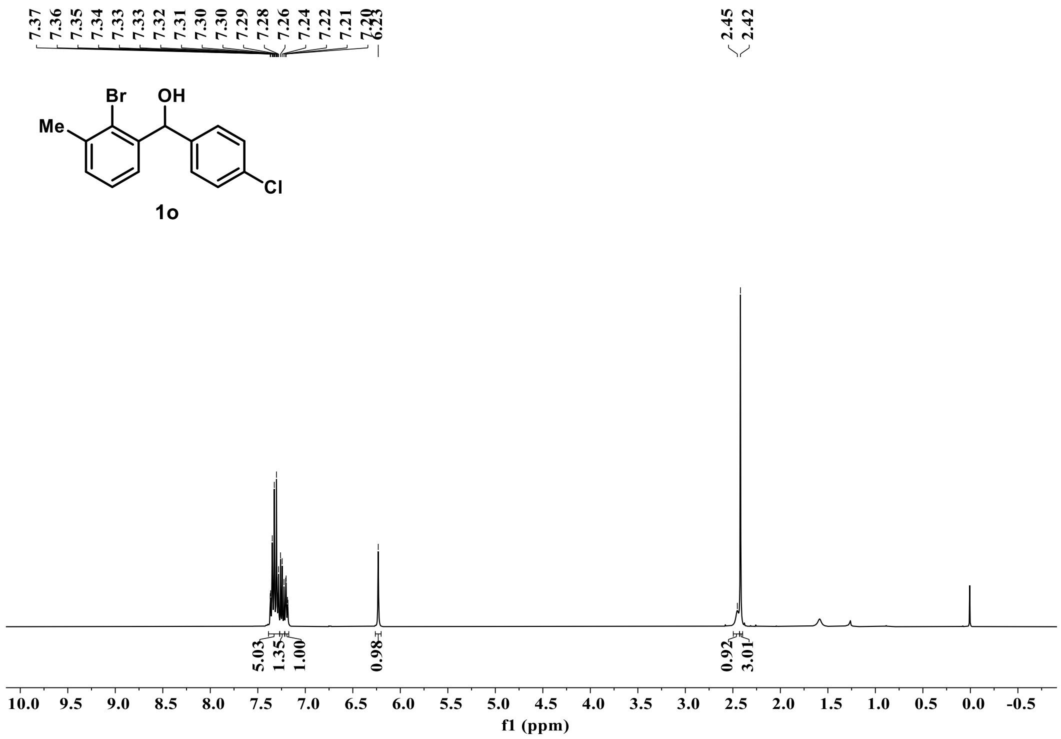

^{13}C NMR (100 MHz, $CDCl_3$)

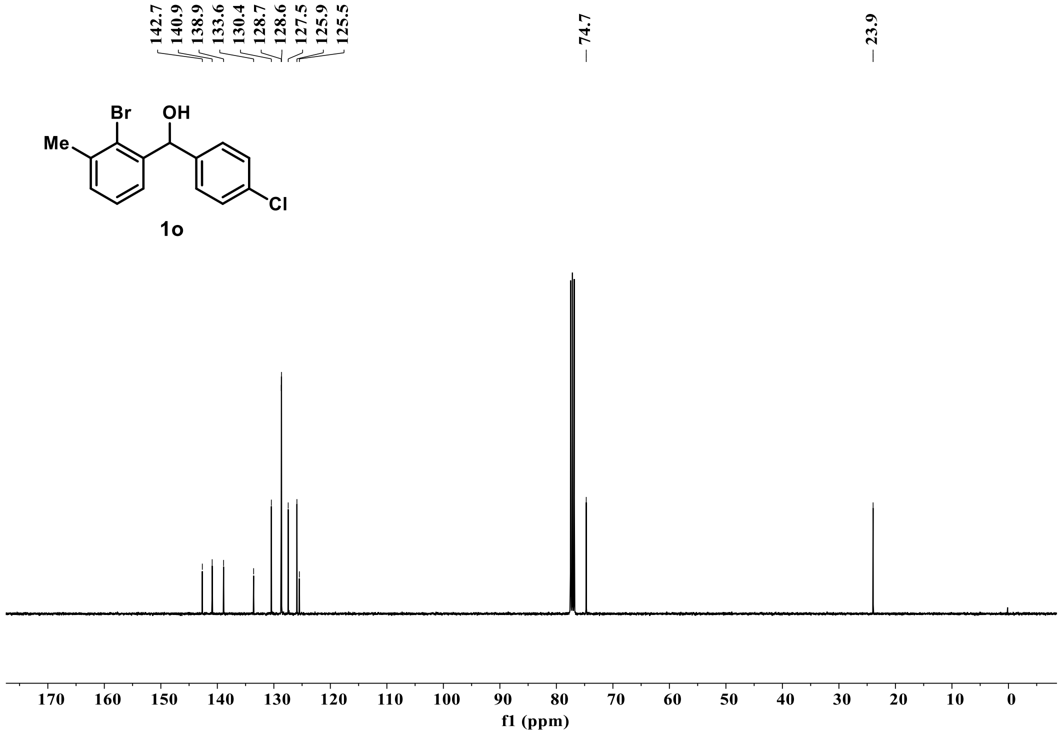

^{1}H NMR (400 MHz, CDCl$_3$)

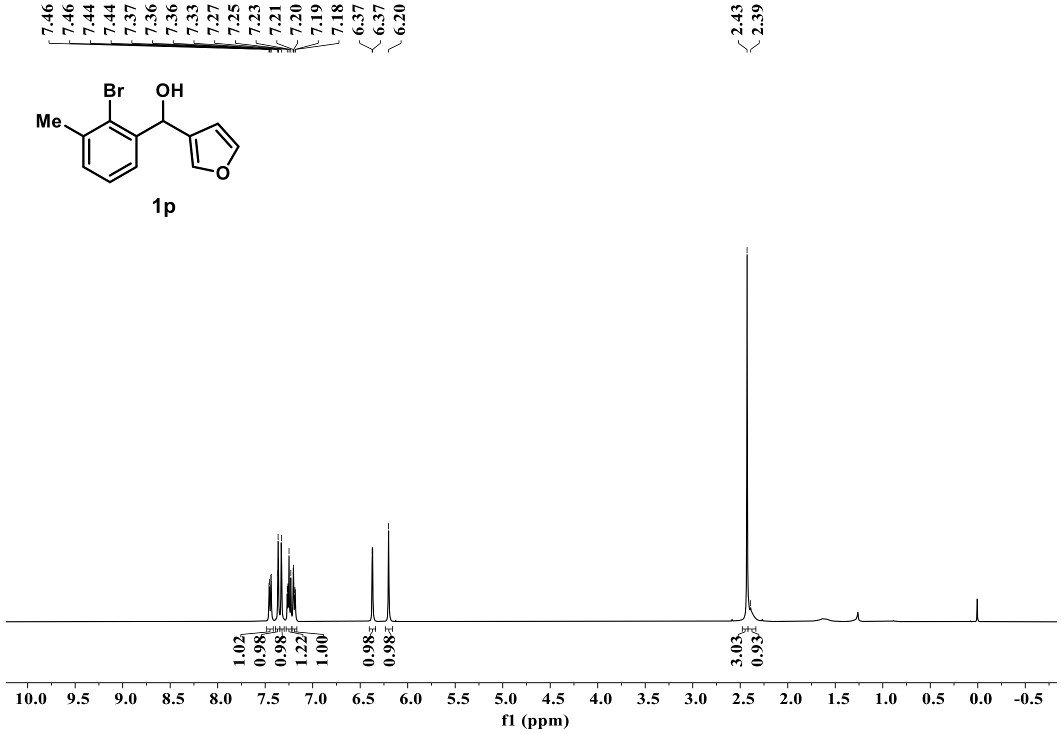

^{13}C NMR (100 MHz, CDCl$_3$)

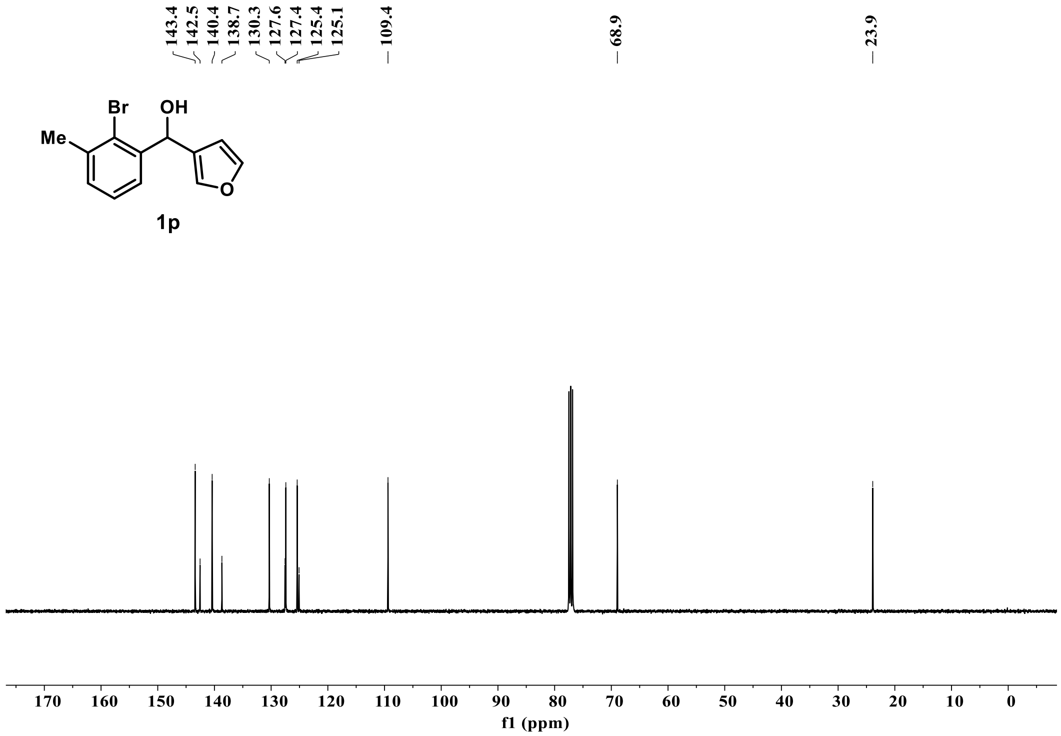

^{1}H NMR (400 MHz, $CDCl_3$)

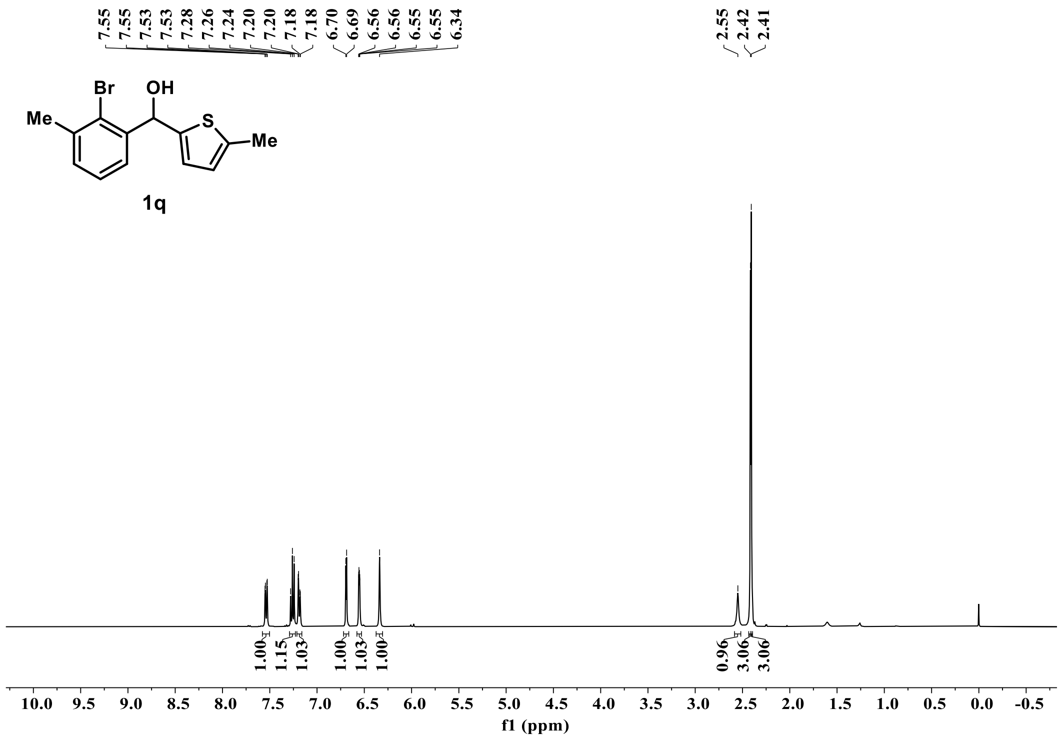

^{13}C NMR (100 MHz, $CDCl_3$)

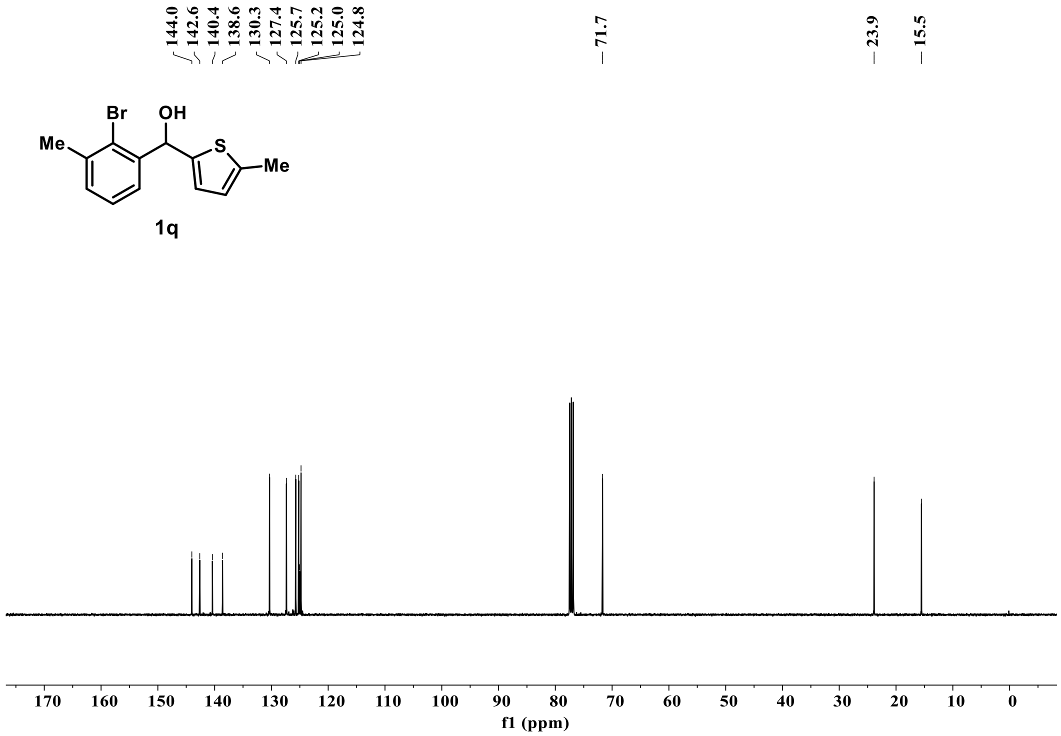

^{1}H NMR (400 MHz, $CDCl_3$)

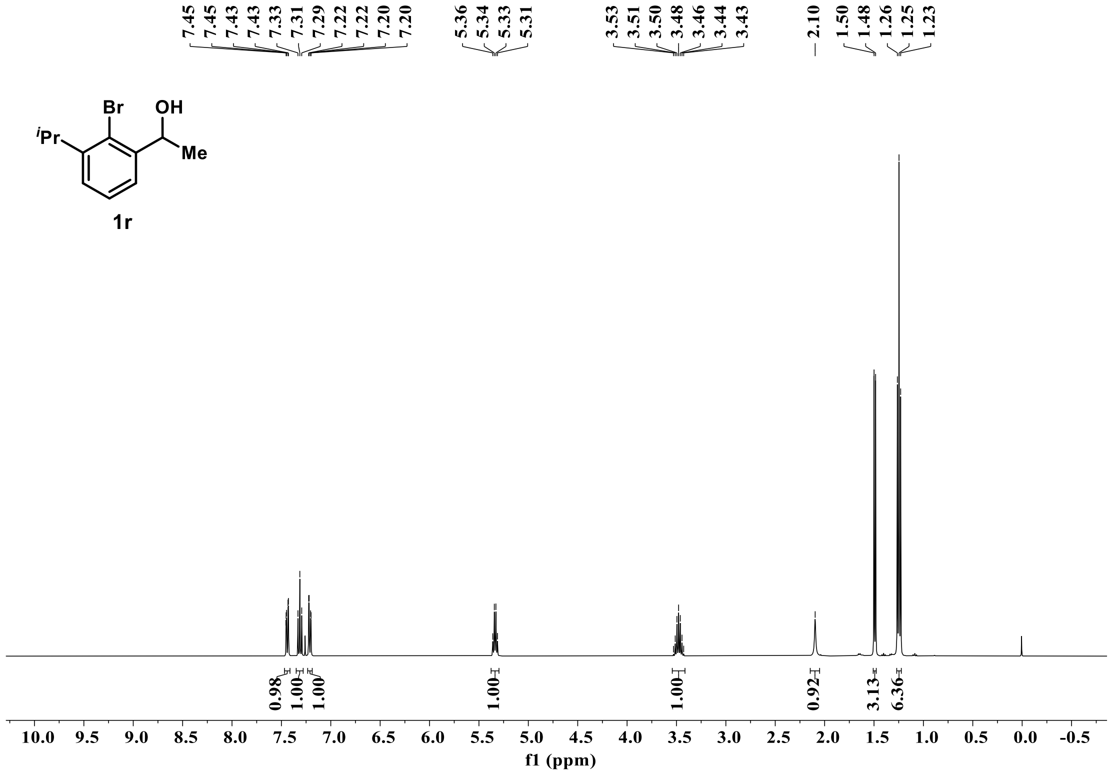

^{13}C NMR (100 MHz, $CDCl_3$)

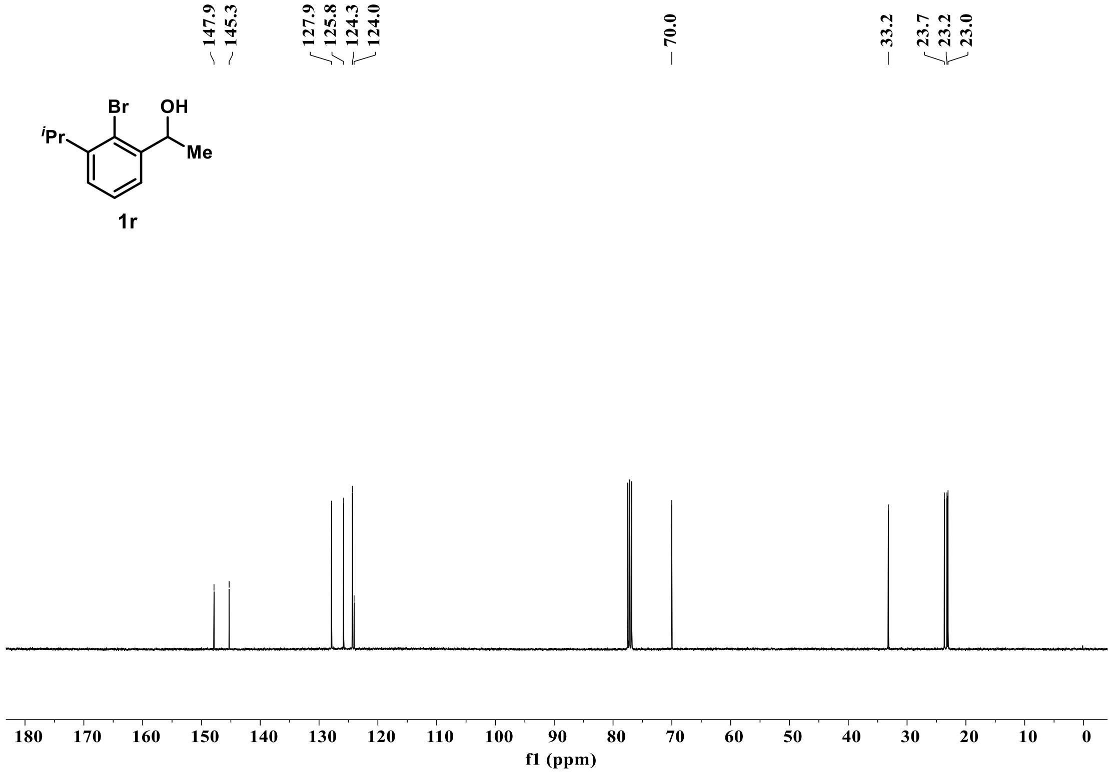

^{1}H NMR (400 MHz, $CDCl_3$)

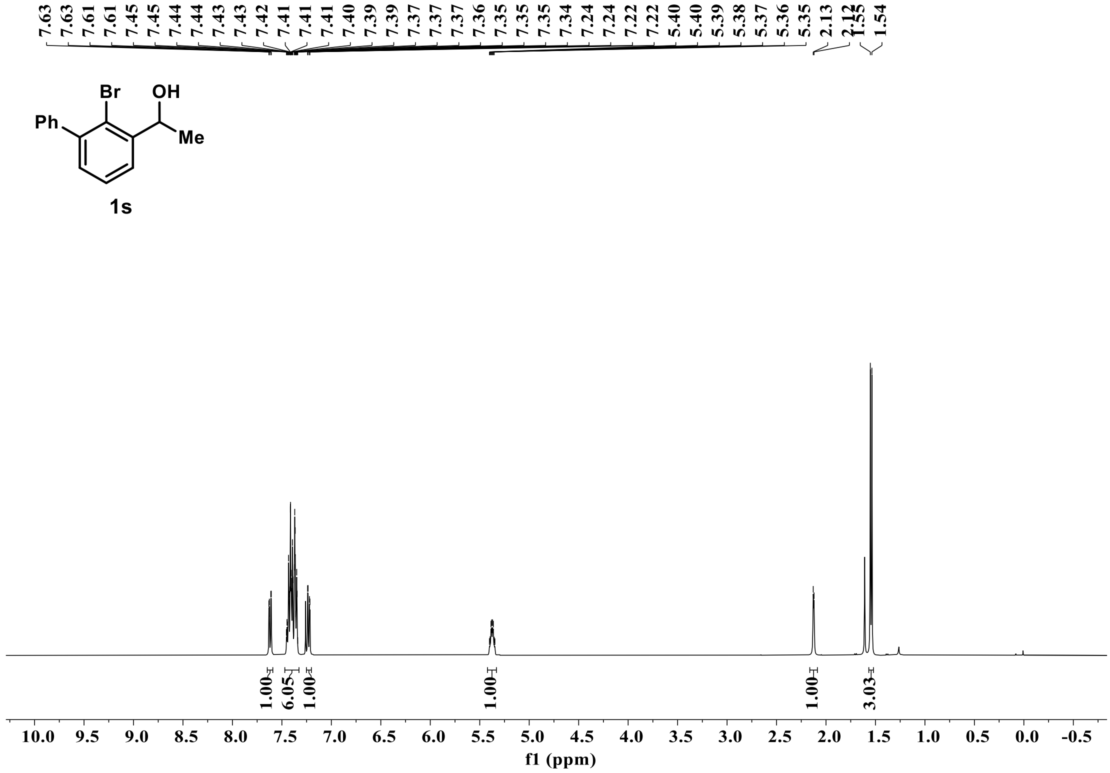

^{13}C NMR (100 MHz, $CDCl_3$)

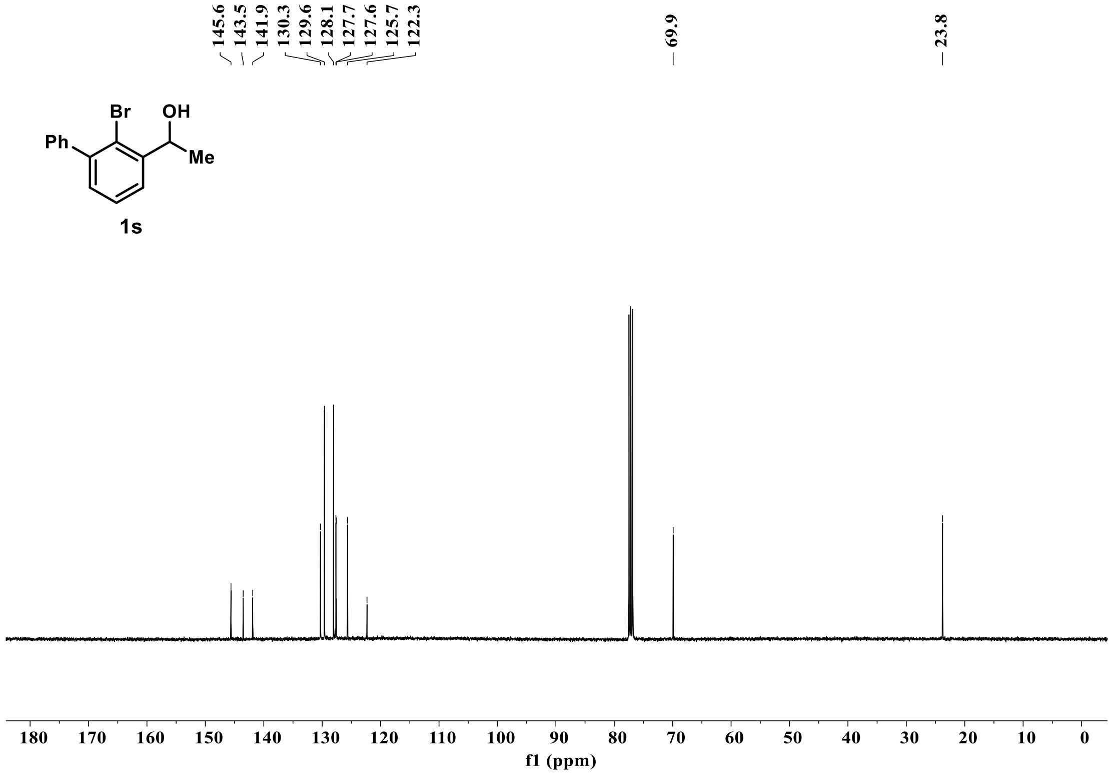

^{1}H NMR (400 MHz, $CDCl_3$)

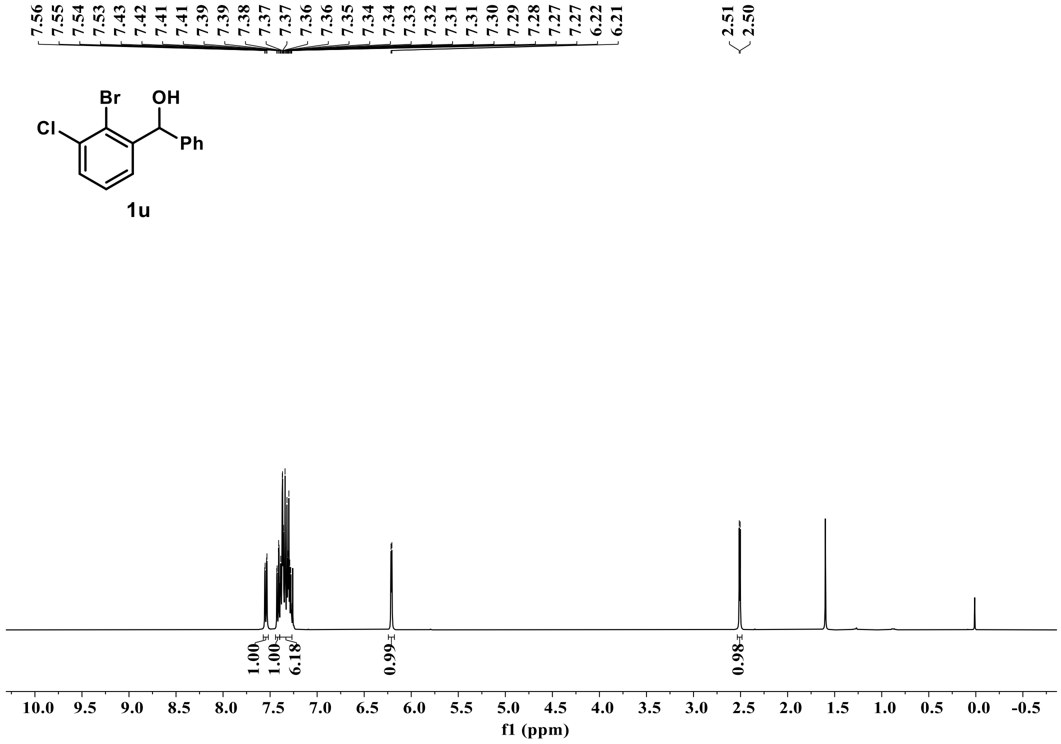

^{13}C NMR (100 MHz, $CDCl_3$)

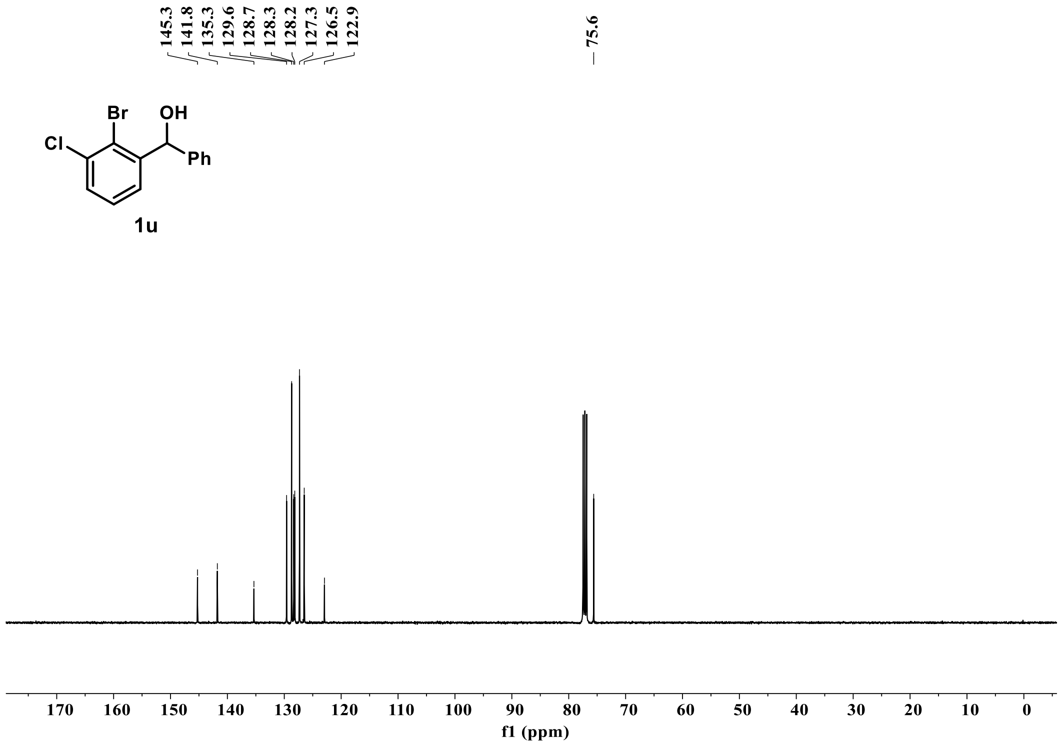

^1H NMR (400 MHz, $CDCl_3$)

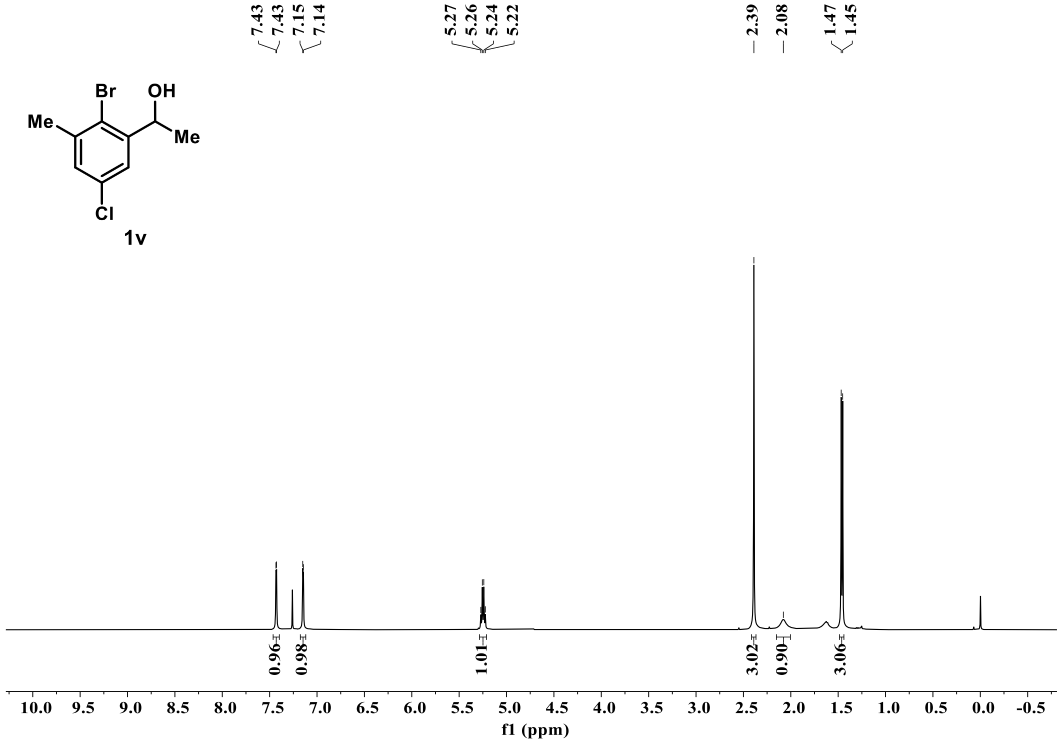

^{13}C NMR (100 MHz, $CDCl_3$)

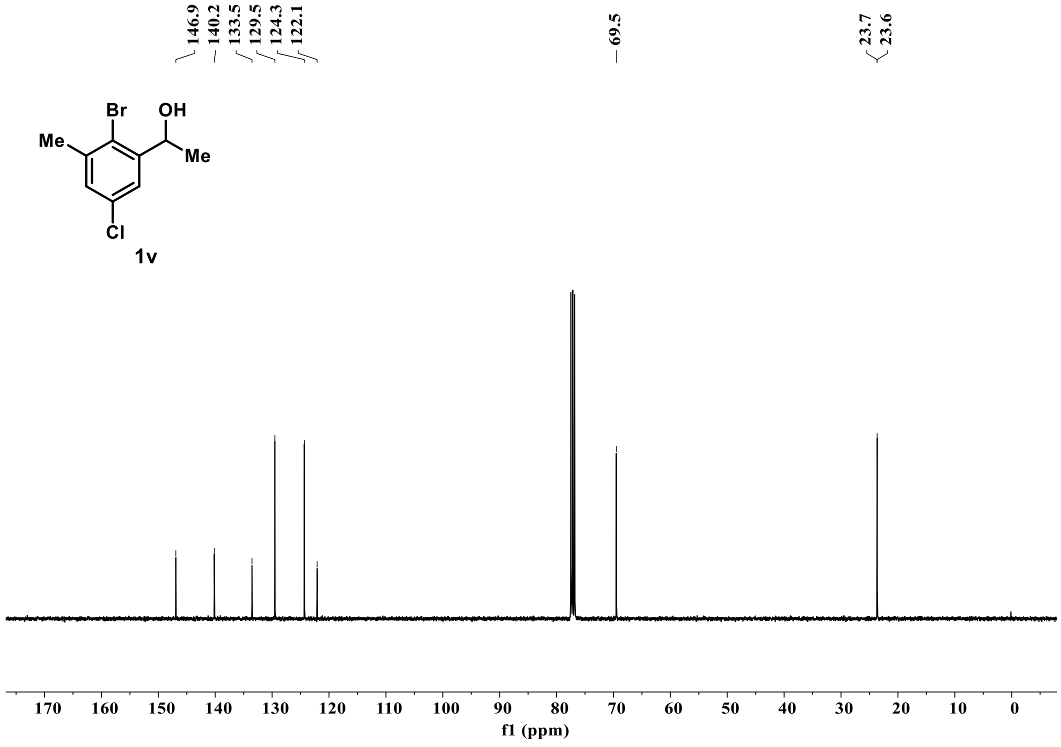

^{1}H NMR (400 MHz, $CDCl_3$)

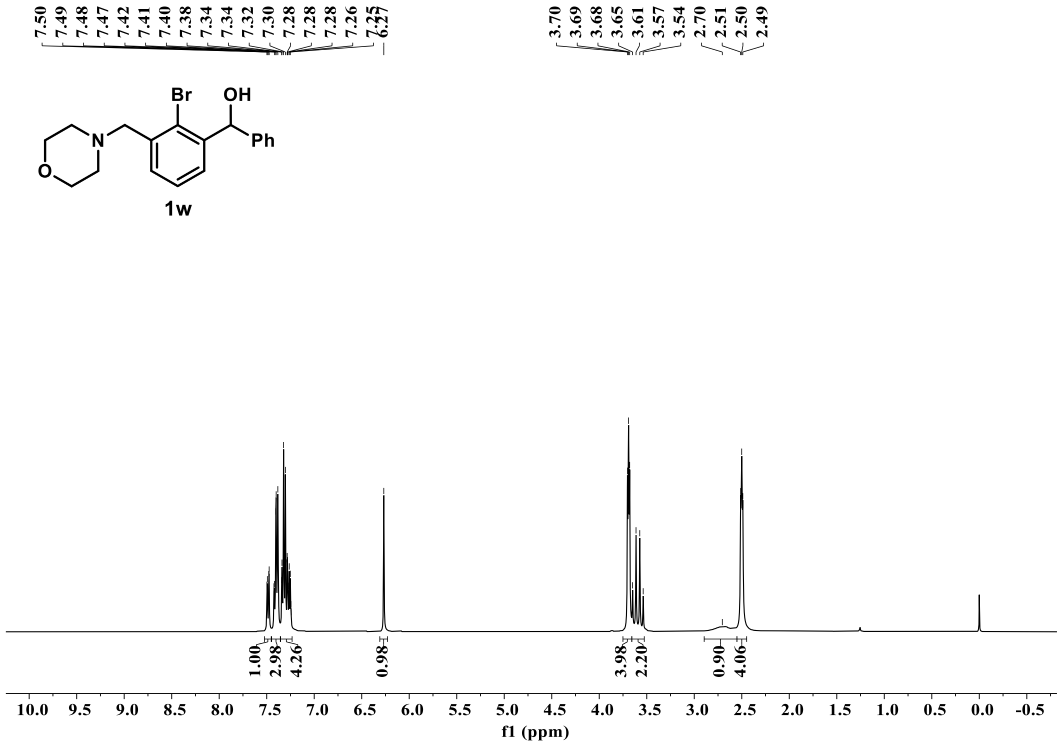

^{13}C NMR (100 MHz, $CDCl_3$)

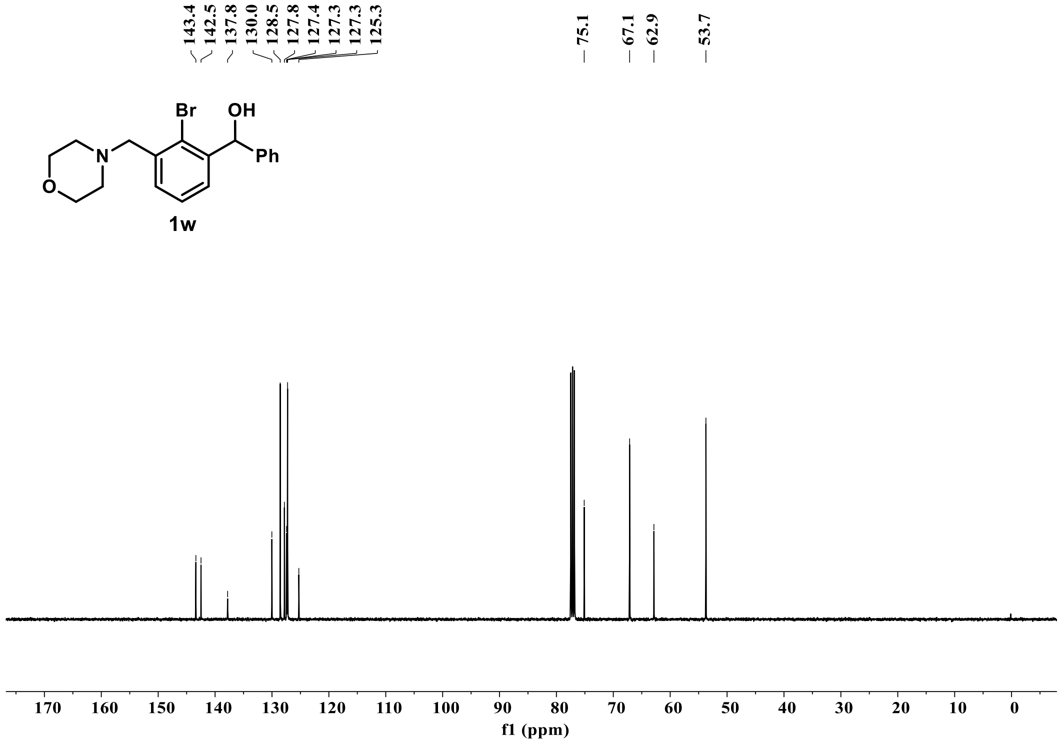

^{1}H NMR (400 MHz, DMSO-d_6)

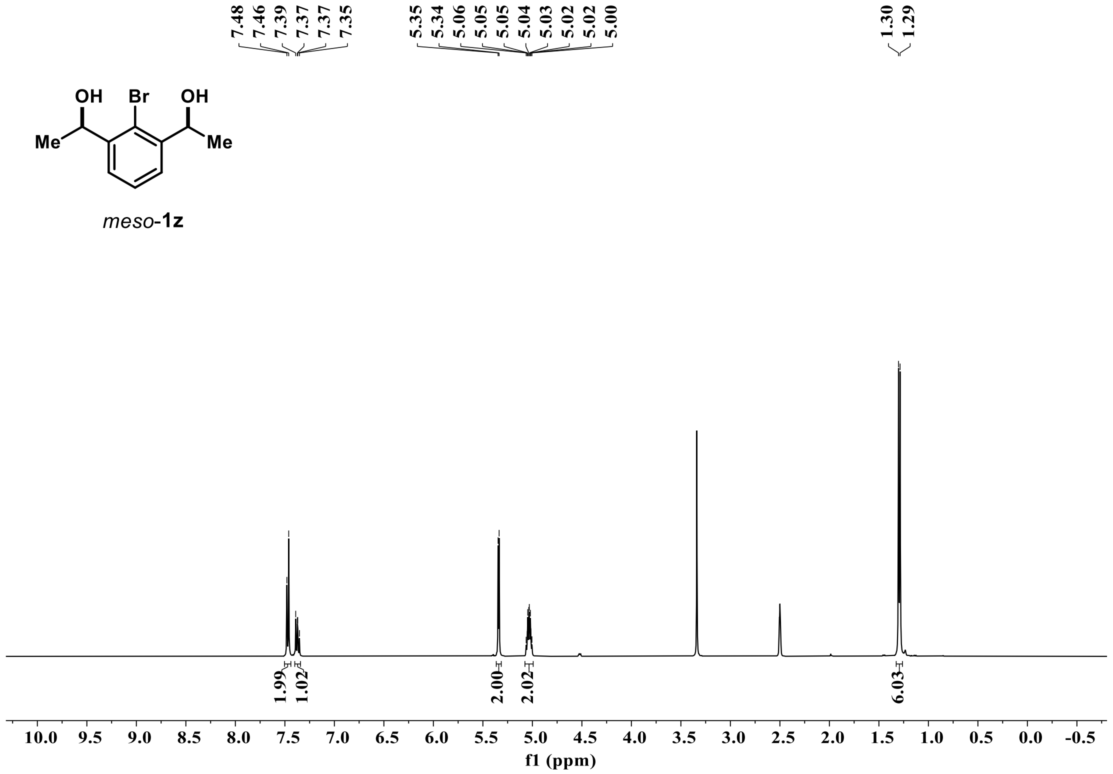

^{13}C NMR (100 MHz, DMSO-d_6)

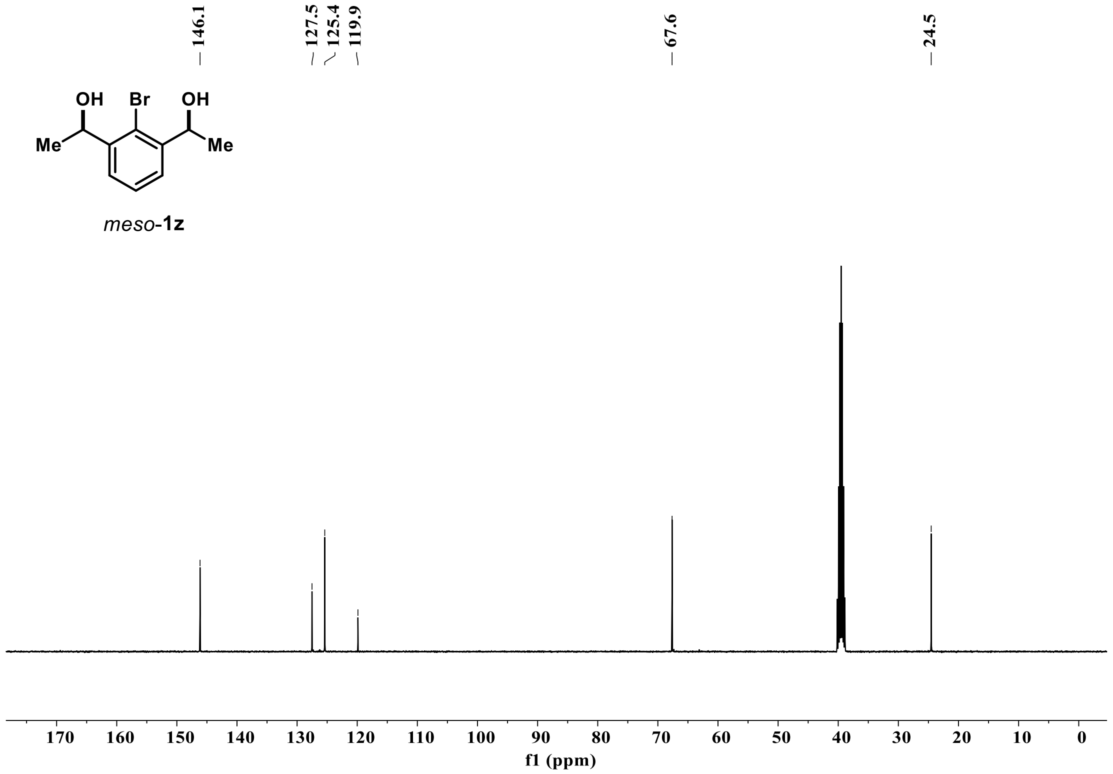

^{1}H NMR (400 MHz, DMSO-d_6)

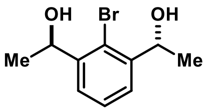

rac-**1z′**

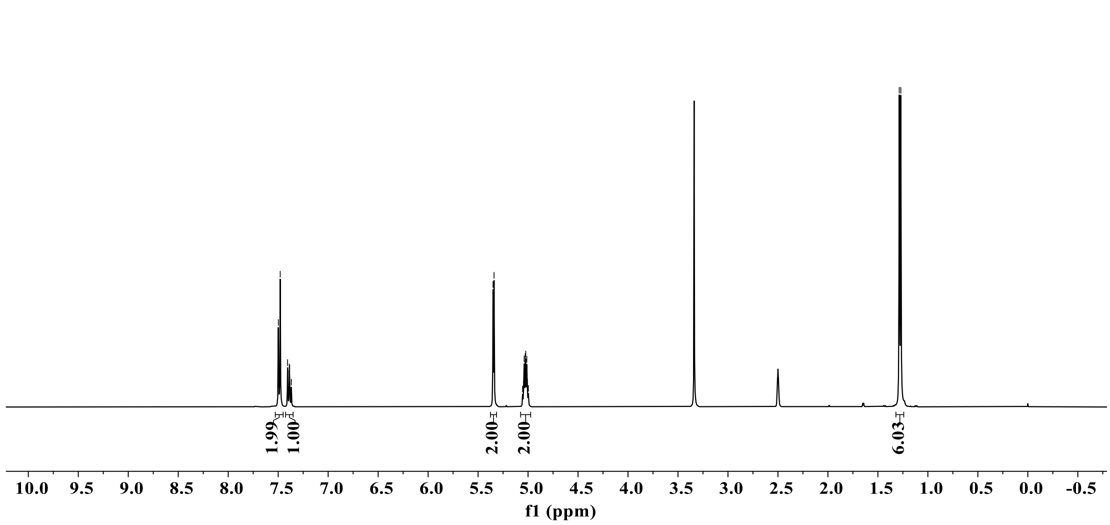

^{13}C NMR (100 MHz, DMSO-d_6)

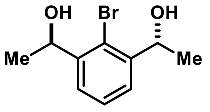

rac-**1z′**

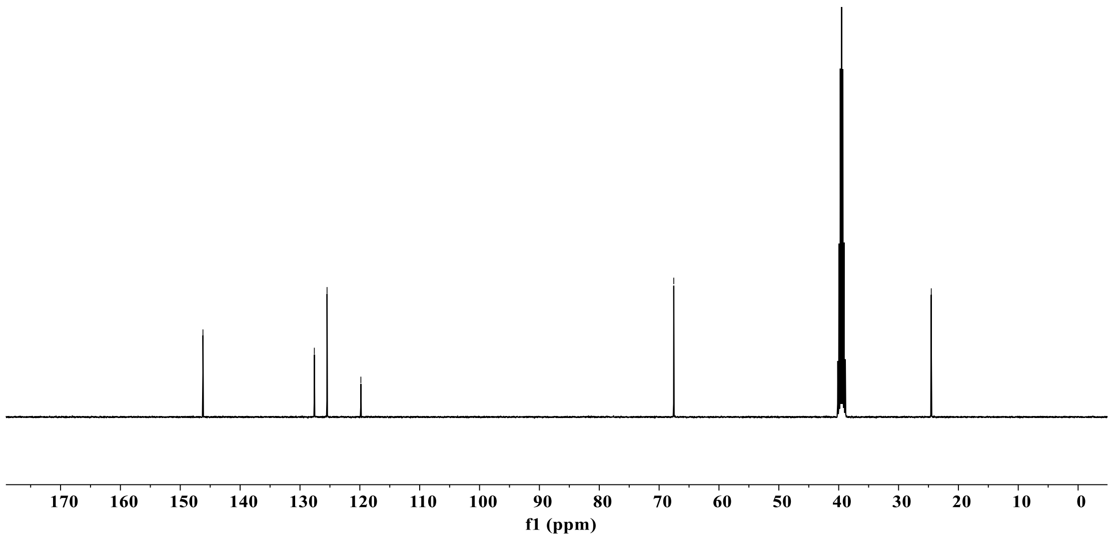

^{1}H NMR (400 MHz, $CDCl_3$)

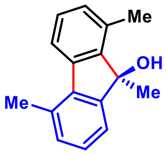

3aa

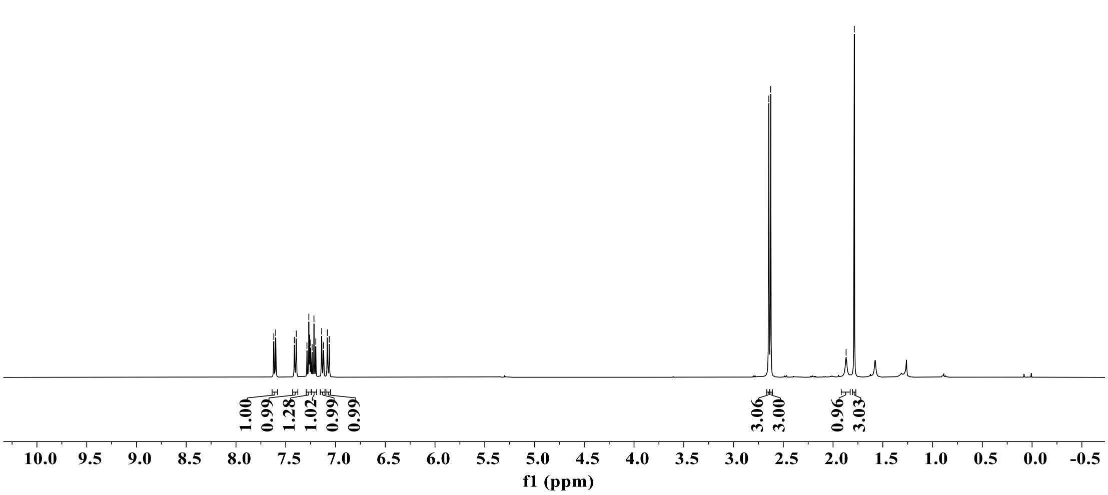

^{13}C NMR (100 MHz, $CDCl_3$)

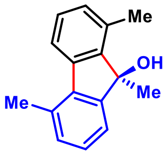

3aa

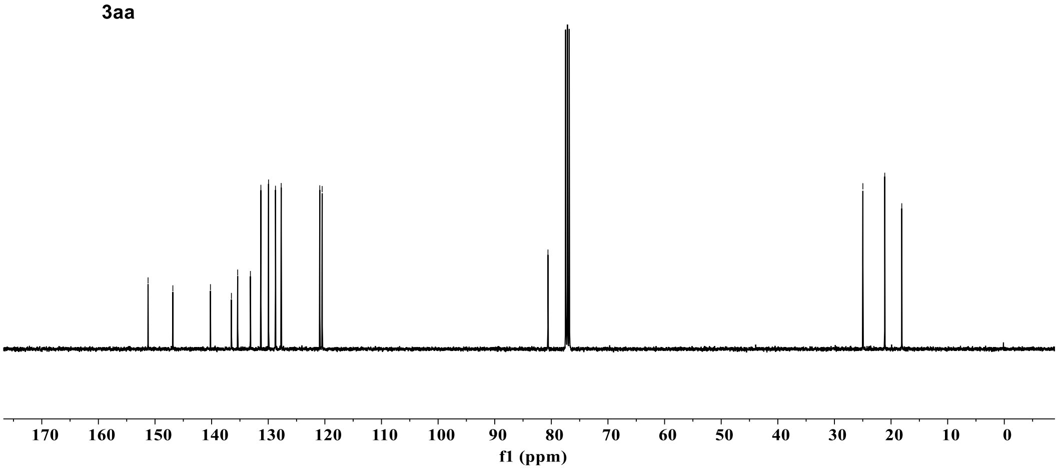

^{1}H NMR (400 MHz, $CDCl_3$)

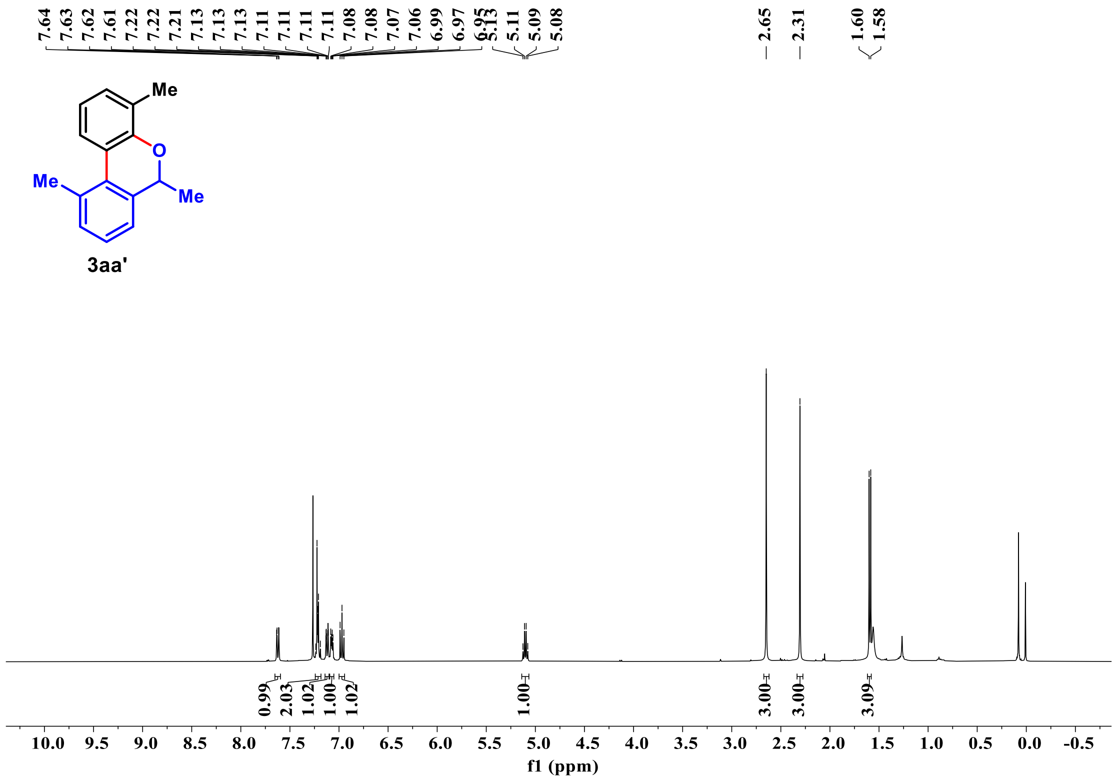

^{13}C NMR (100 MHz, $CDCl_3$)

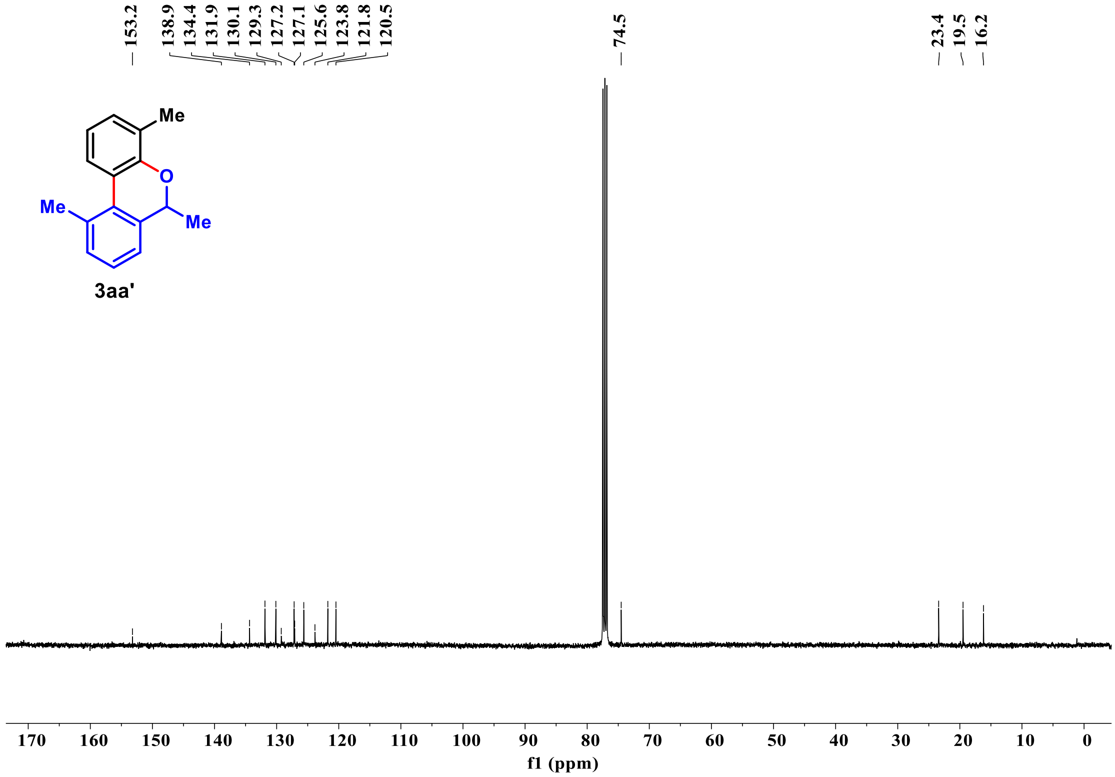

^{1}H NMR (400 MHz, $CDCl_3$)

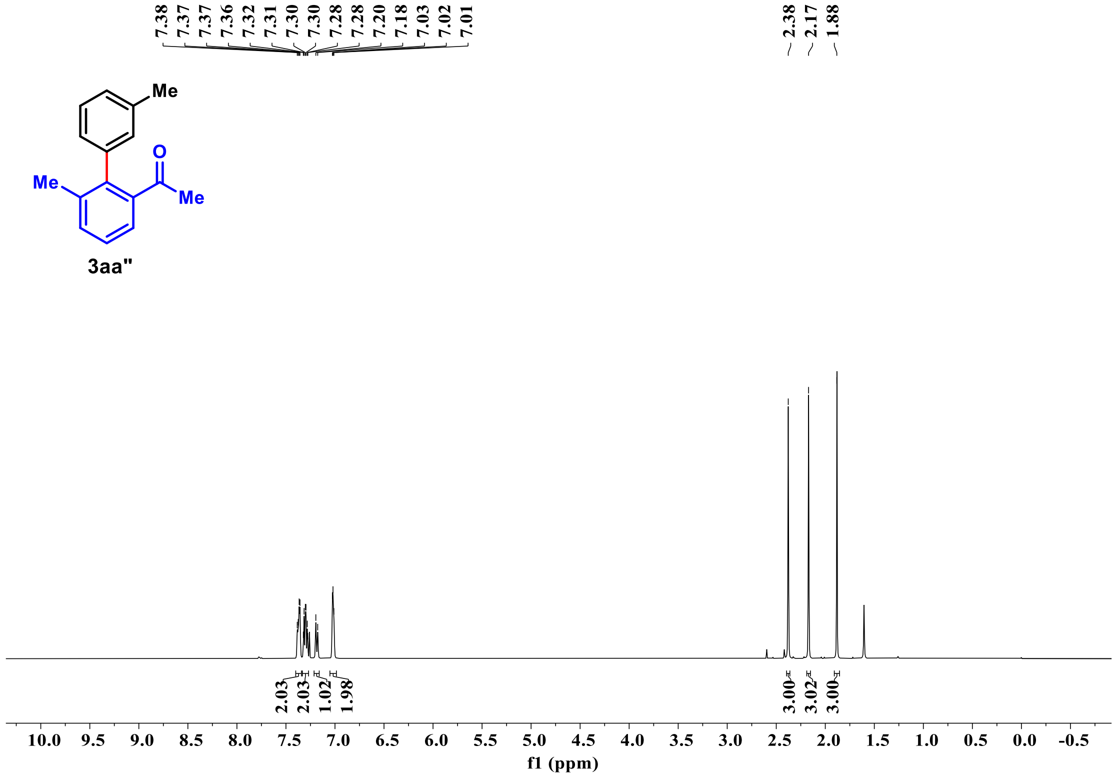

^{13}C NMR (100 MHz, $CDCl_3$)

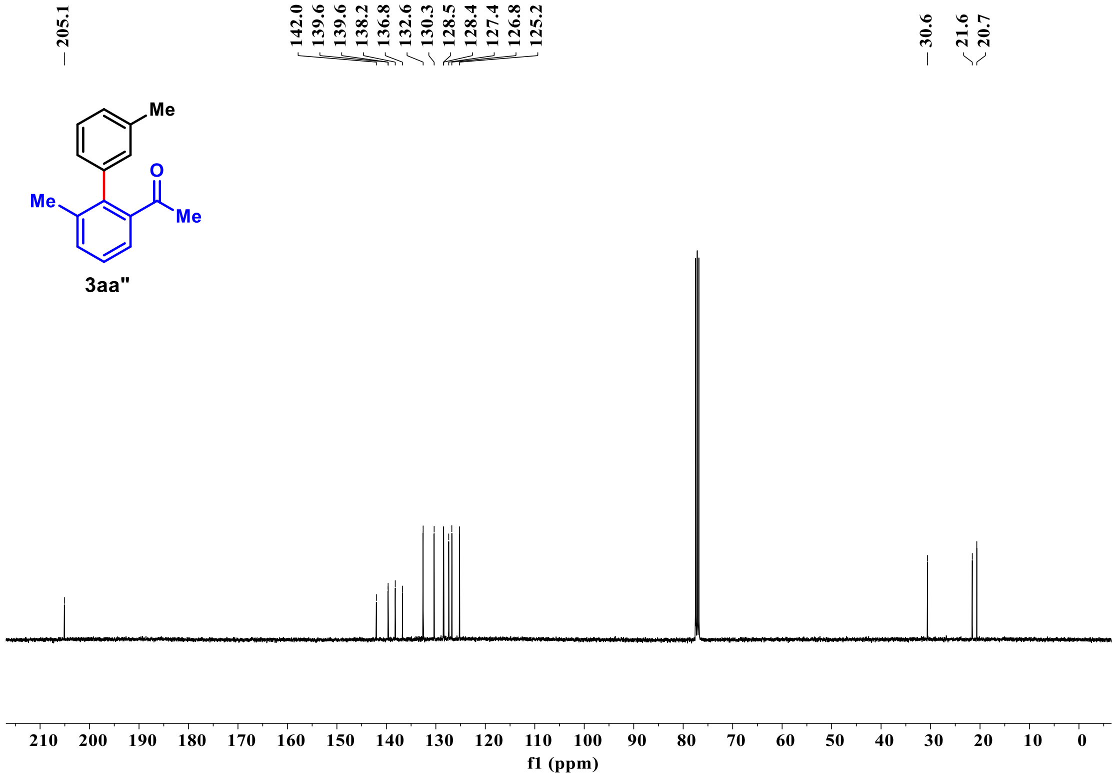

^{1}H NMR (400 MHz, $CDCl_3$)

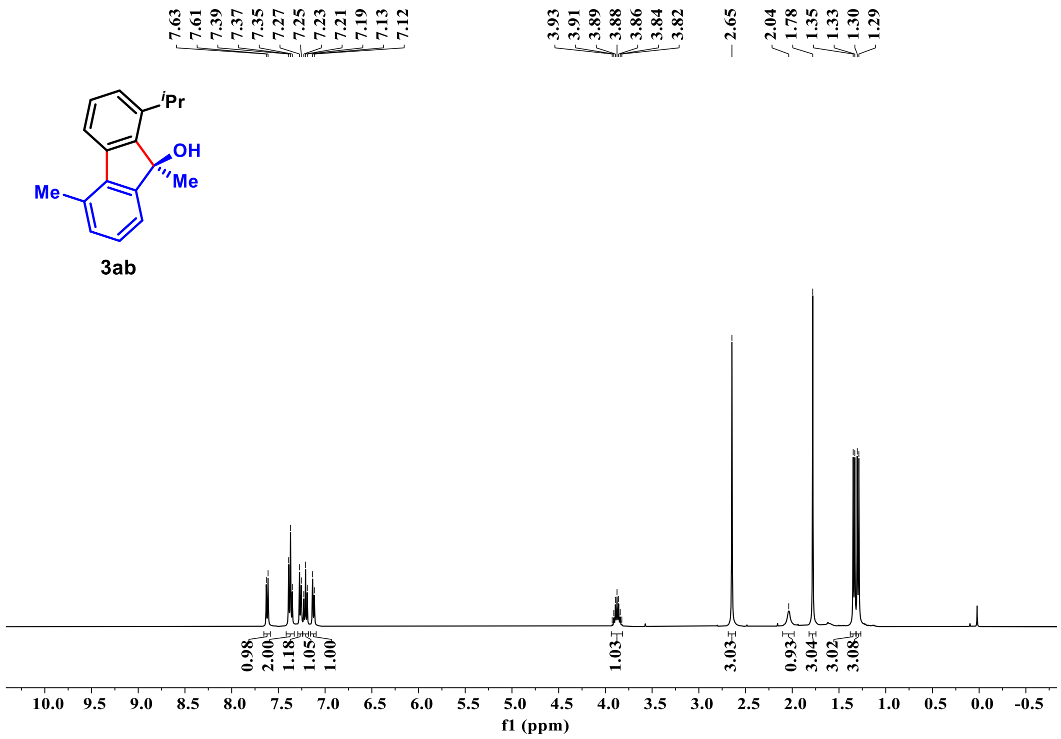

^{13}C NMR (100 MHz, $CDCl_3$)

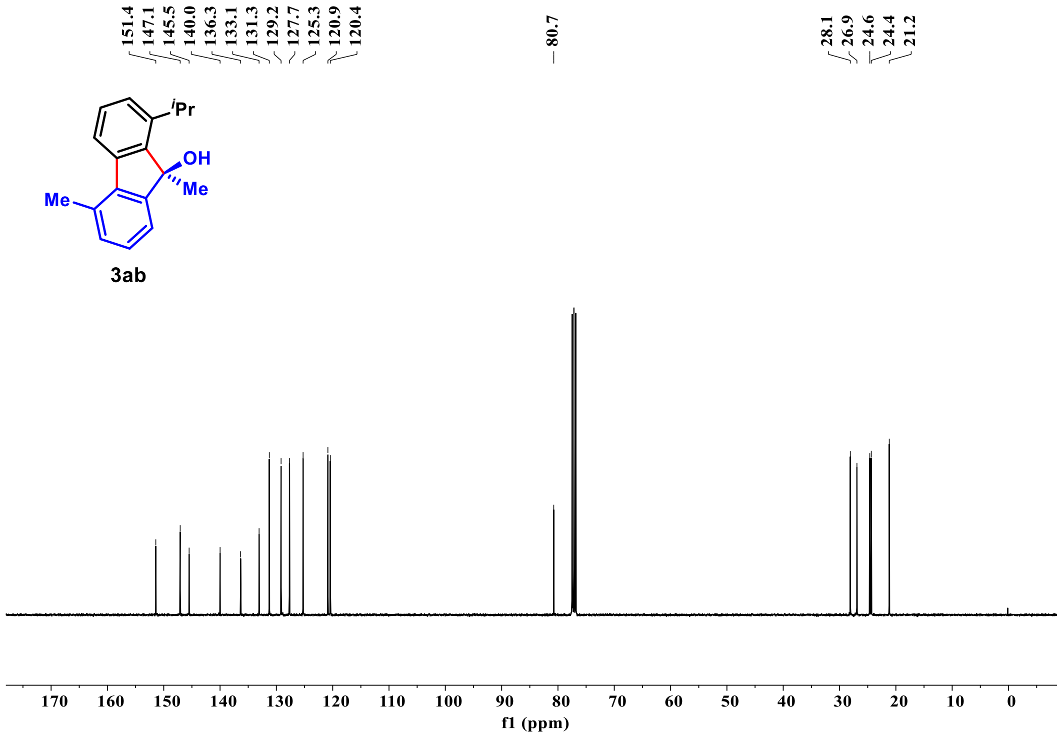

^{1}H NMR (400 MHz, $CDCl_3$)

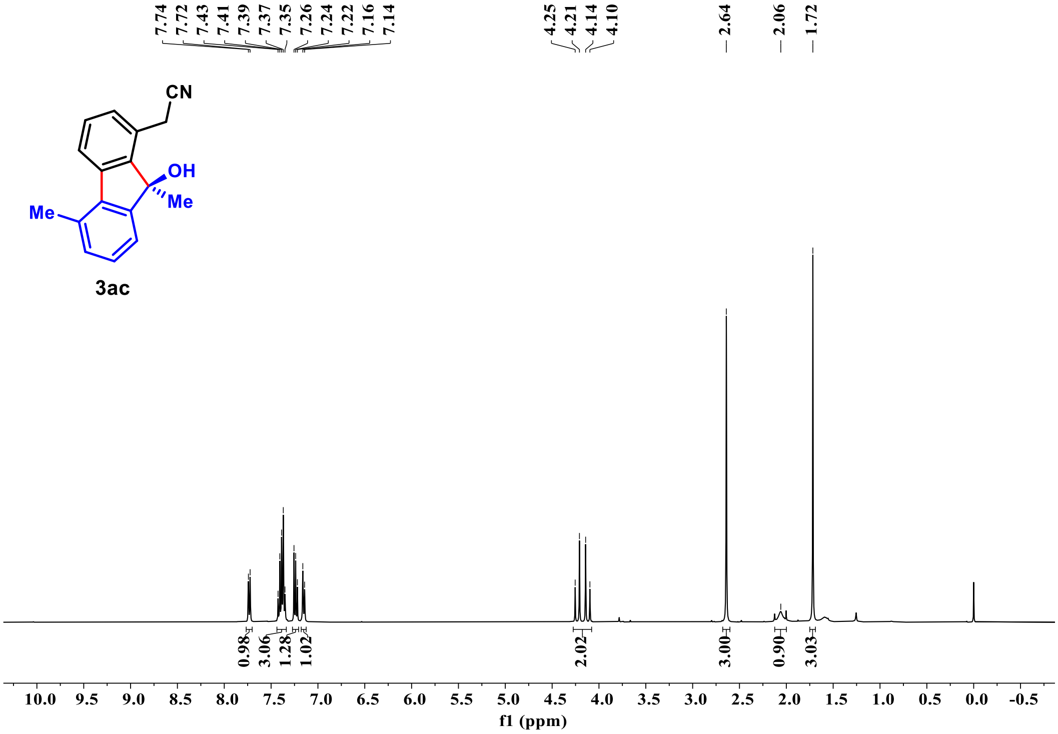

^{13}C NMR (100 MHz, $CDCl_3$)

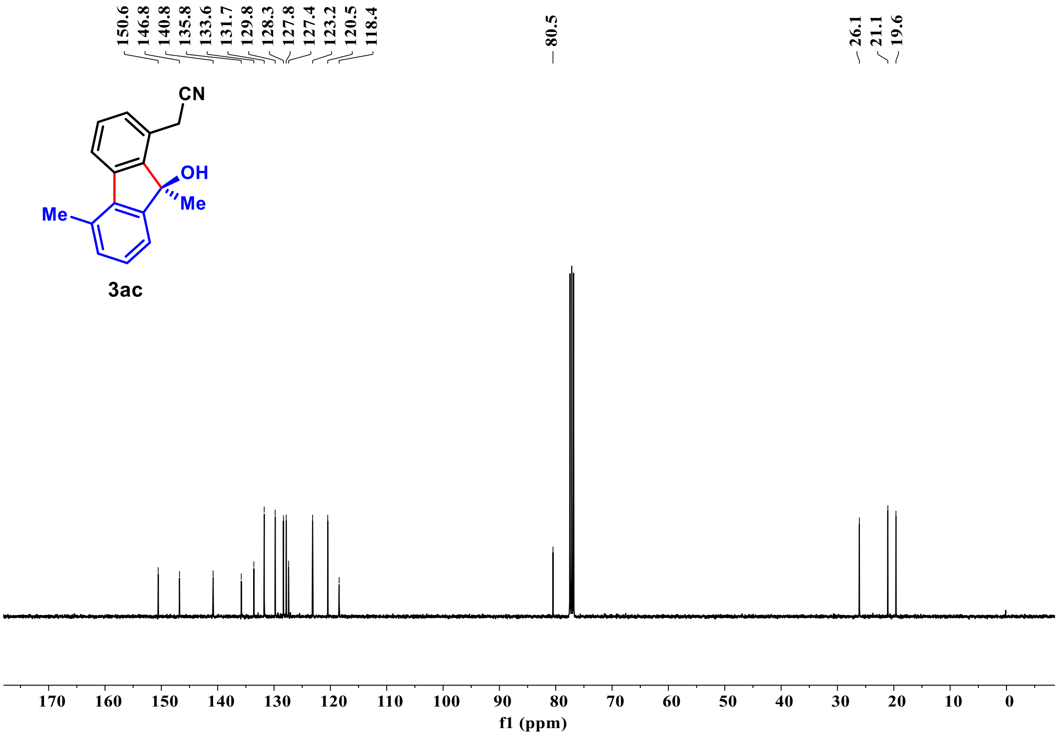

^{1}H NMR (400 MHz, CDCl$_3$)

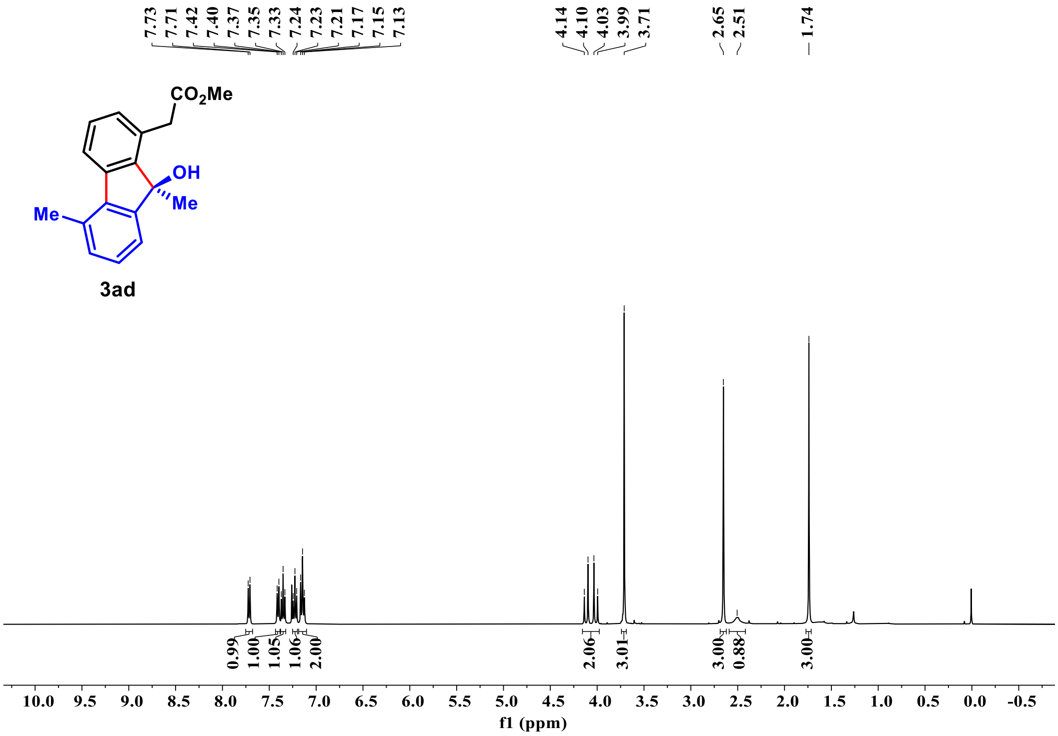

^{13}C NMR (100 MHz, CDCl$_3$)

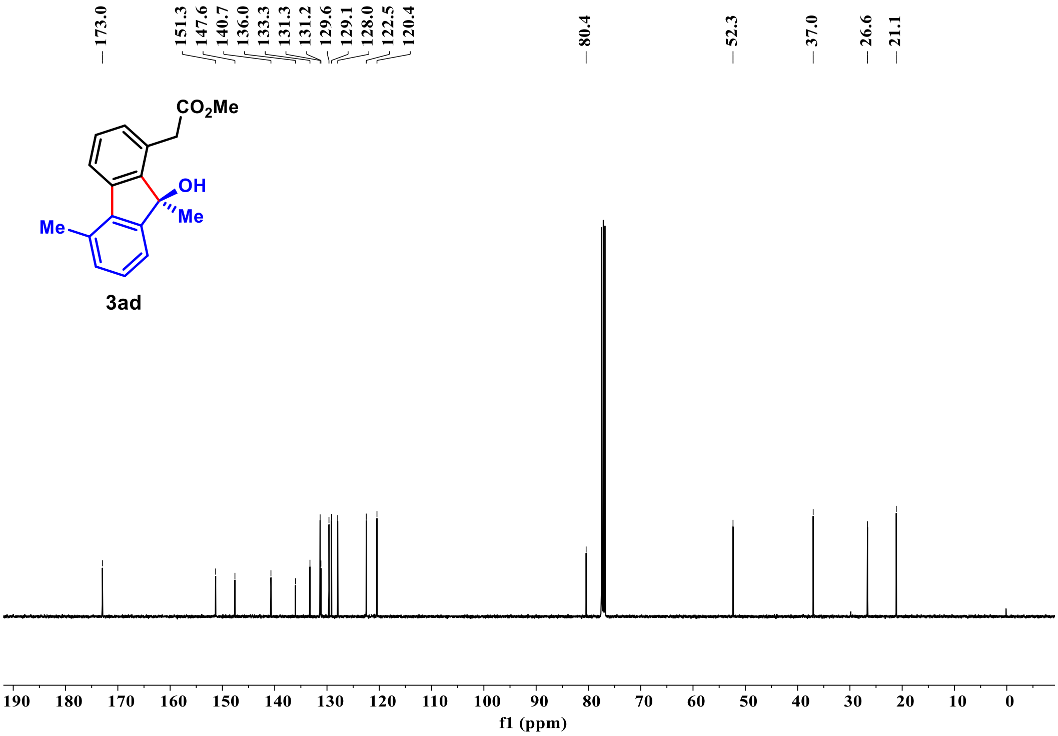

^{1}H NMR (400 MHz, CDCl$_3$)

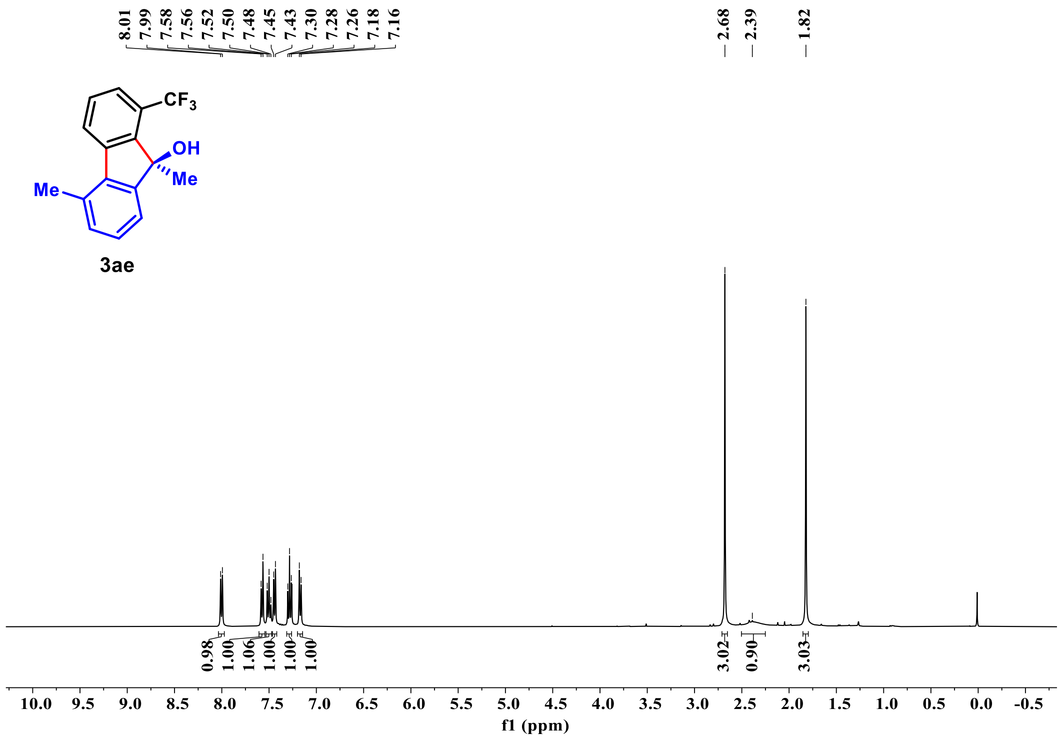

^{13}C NMR (100 MHz, CDCl$_3$)

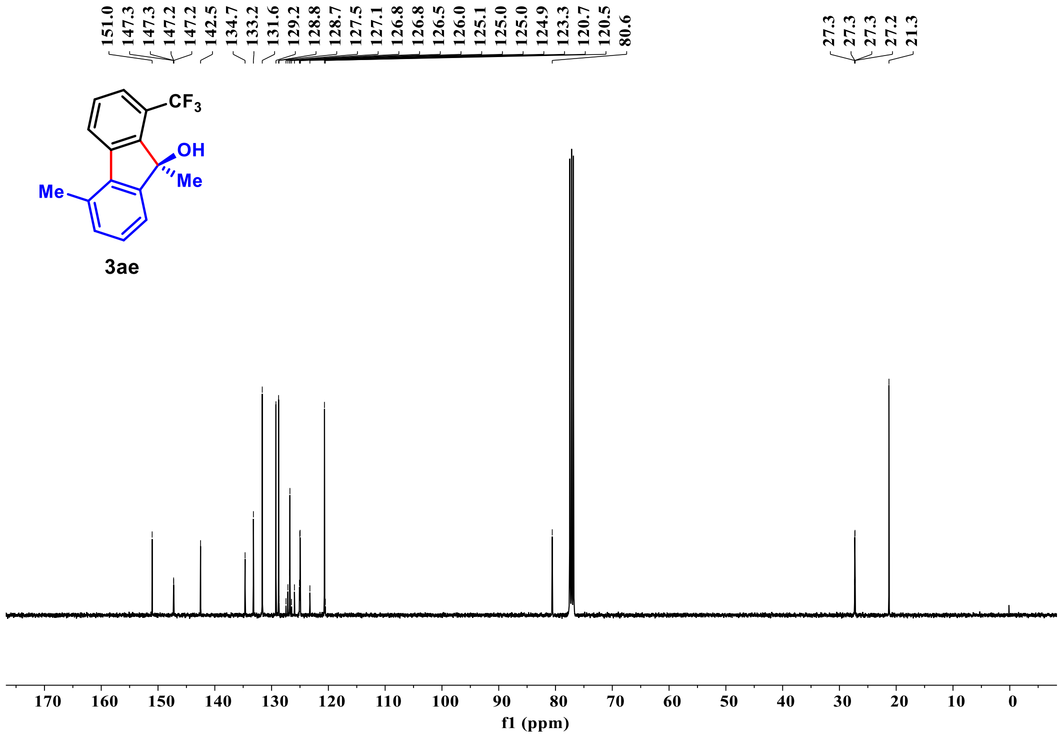

^{19}F NMR (376 MHz, $CDCl_3$)

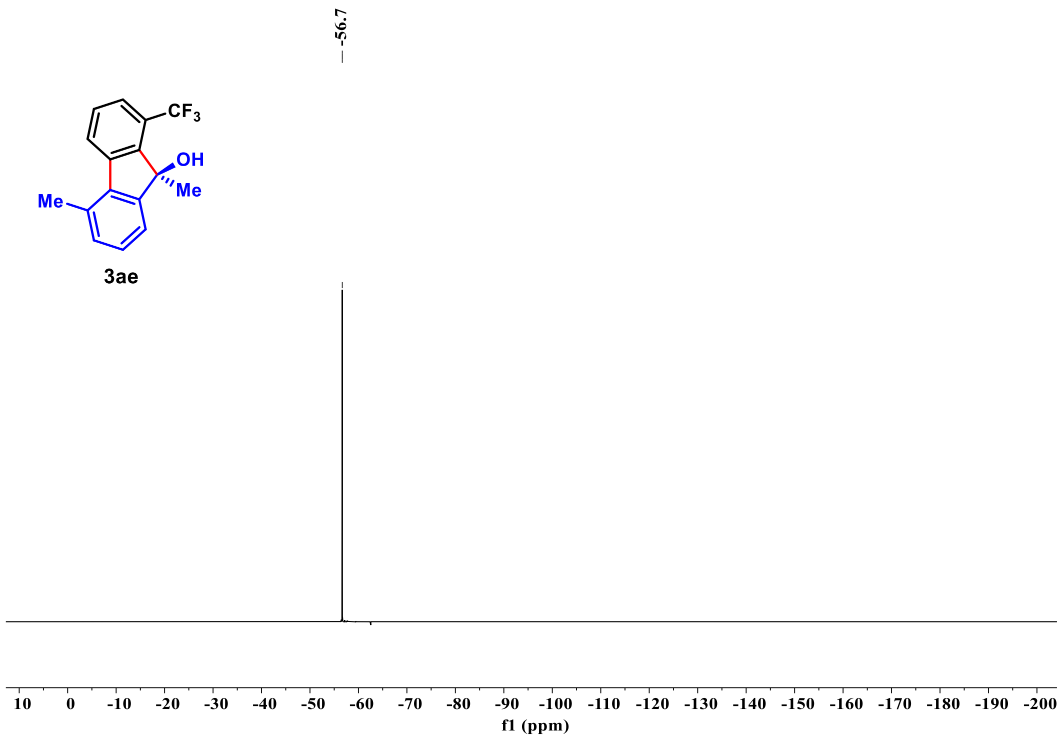

^{1}H NMR (400 MHz, $CDCl_3$)

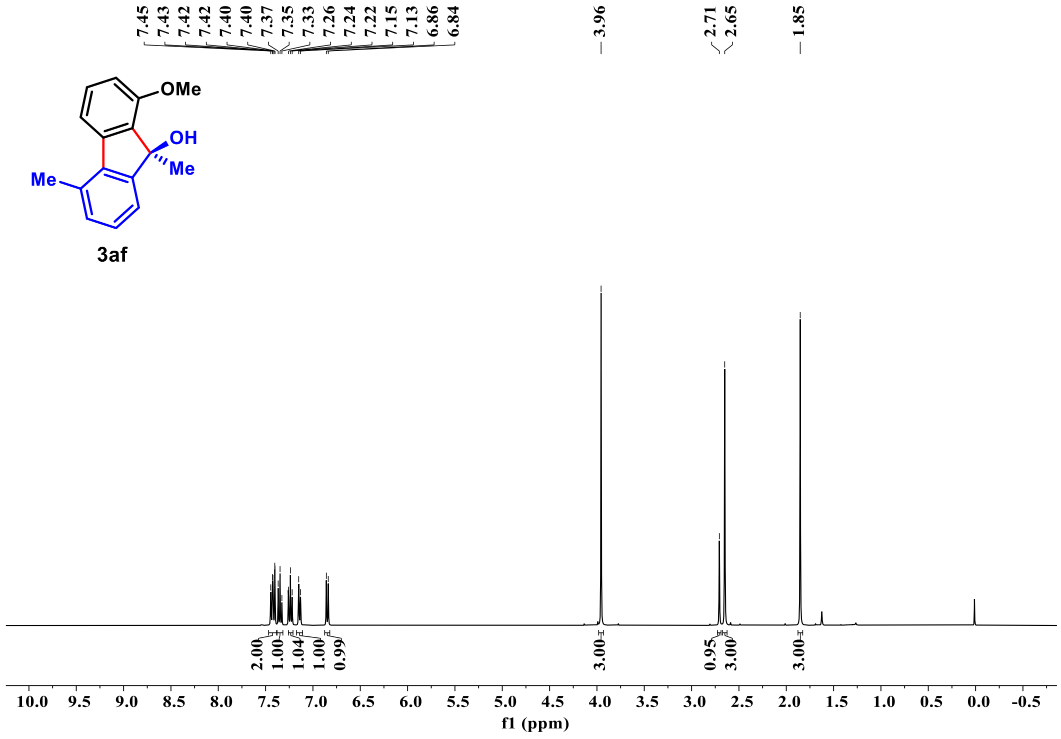

^{13}C NMR (100 MHz, $CDCl_3$)

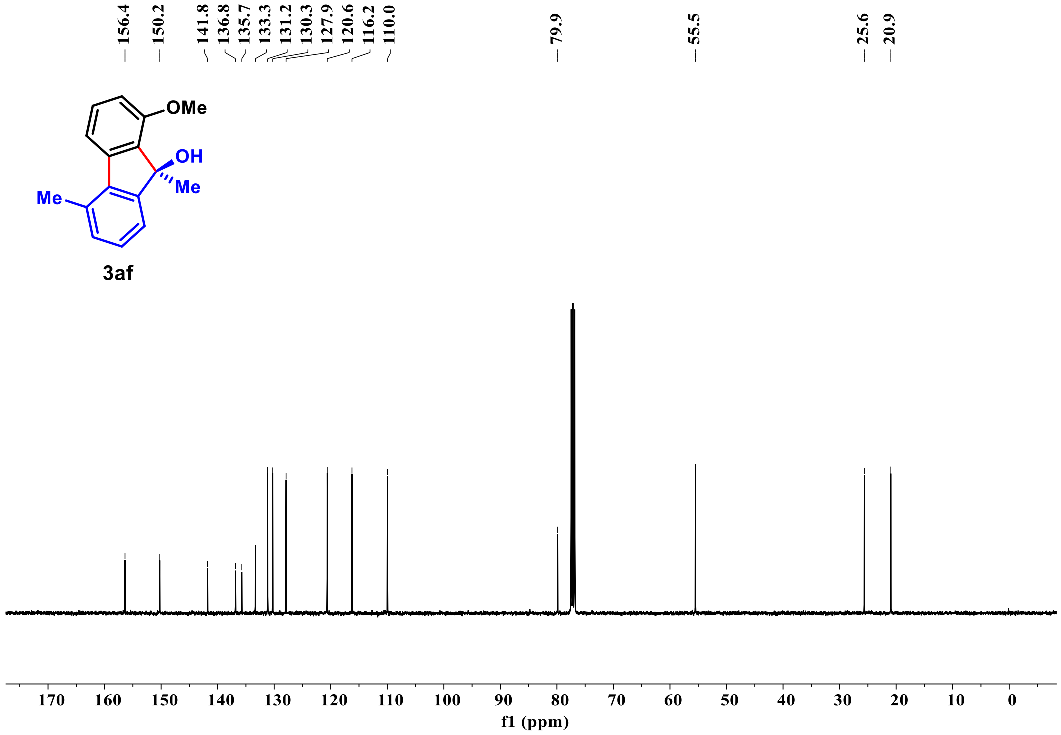

^{1}H NMR (400 MHz, $CDCl_3$)

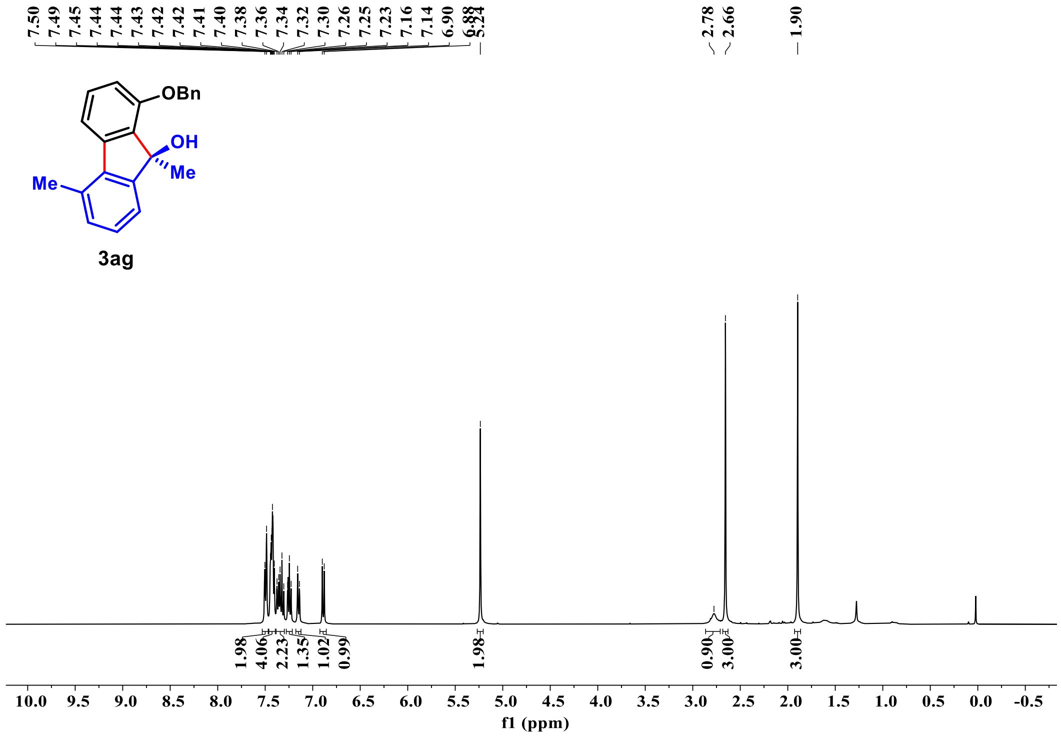

^{13}C NMR (100 MHz, $CDCl_3$)

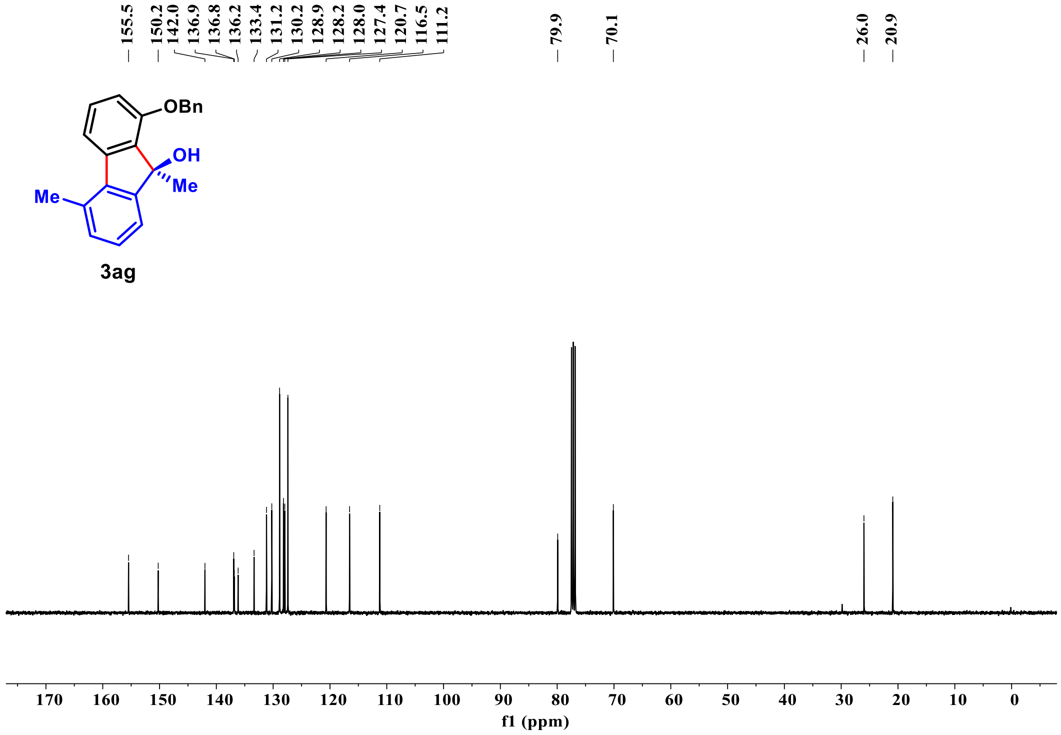

^{1}H NMR (400 MHz, $CDCl_3$)

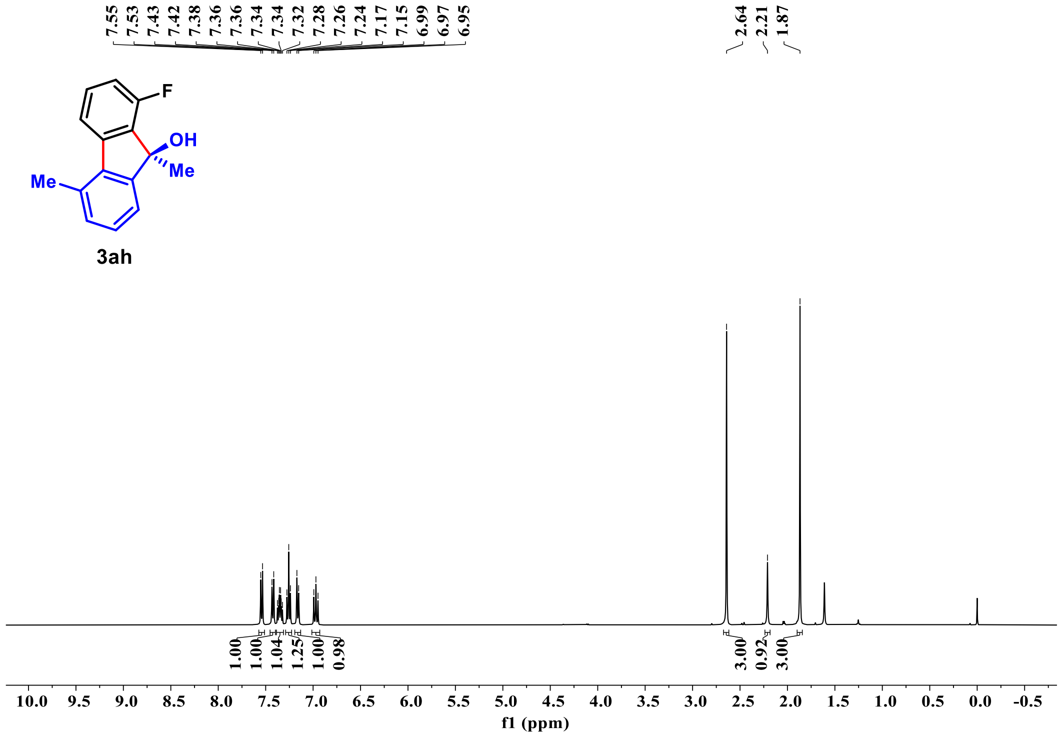

^{13}C NMR (100 MHz, $CDCl_3$)

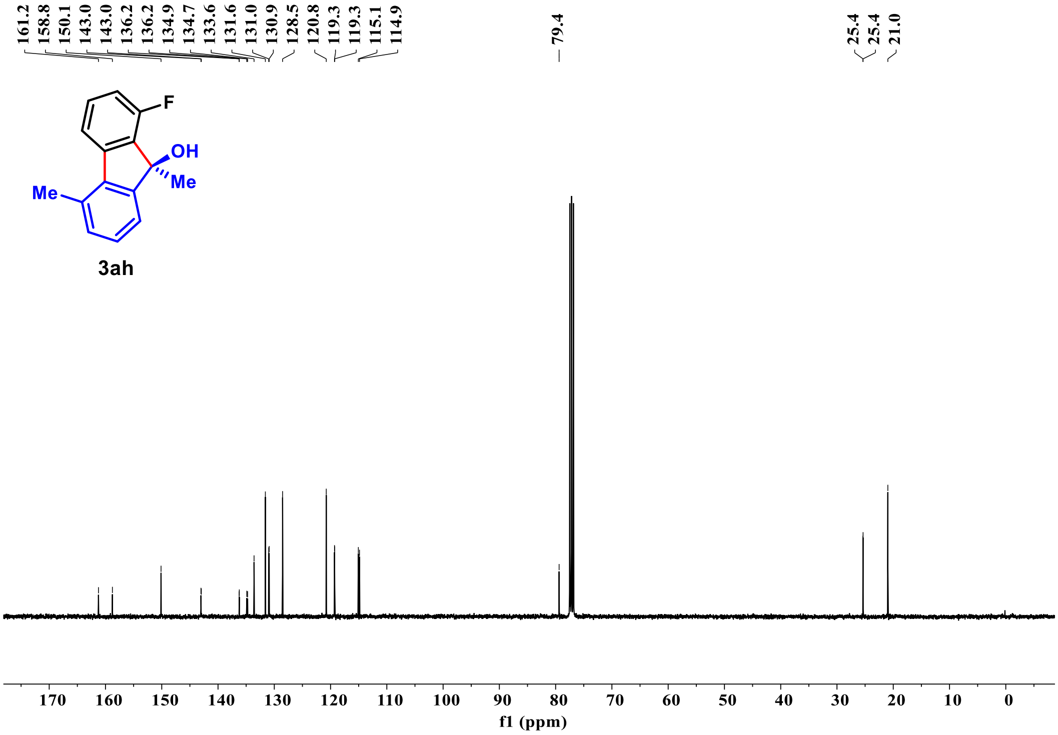

^{19}F NMR (376 MHz, $CDCl_3$)

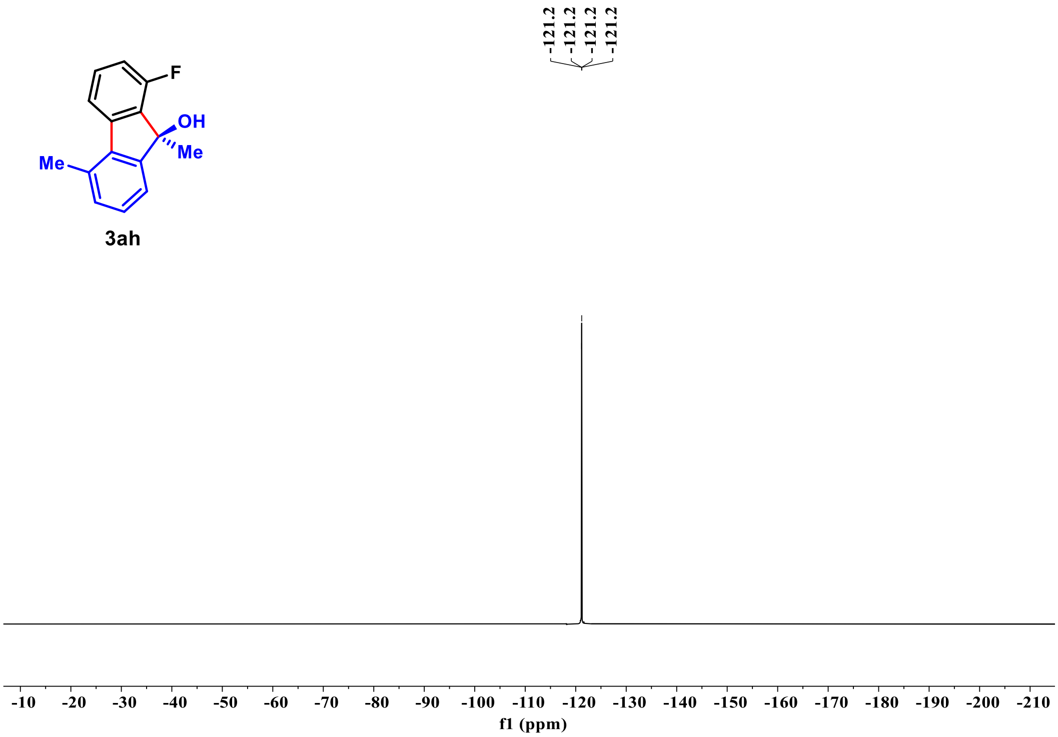

^{1}H NMR (400 MHz, CDCl$_3$)

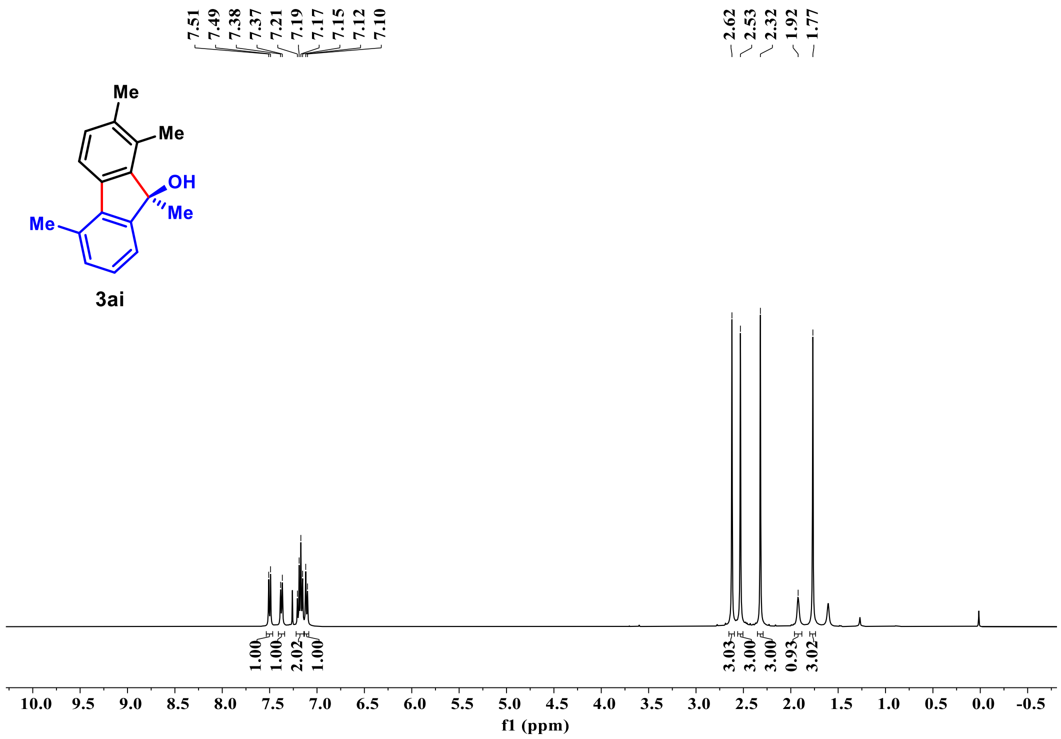

^{13}C NMR (100 MHz, CDCl$_3$)

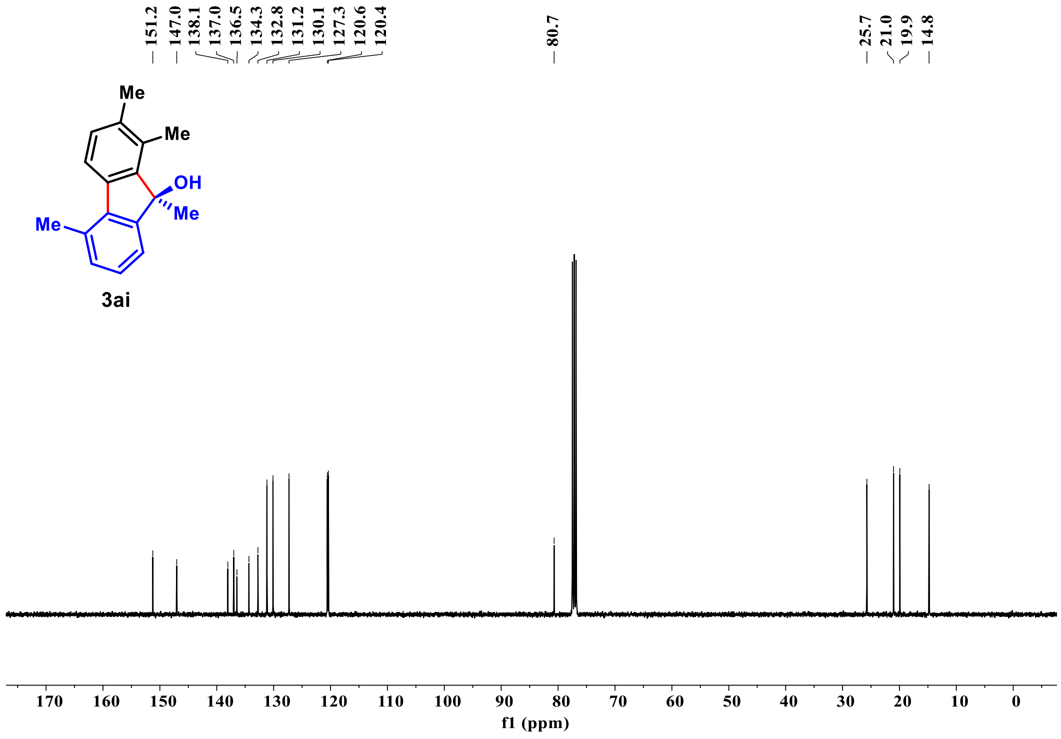

^{1}H NMR (400 MHz, $CDCl_3$)

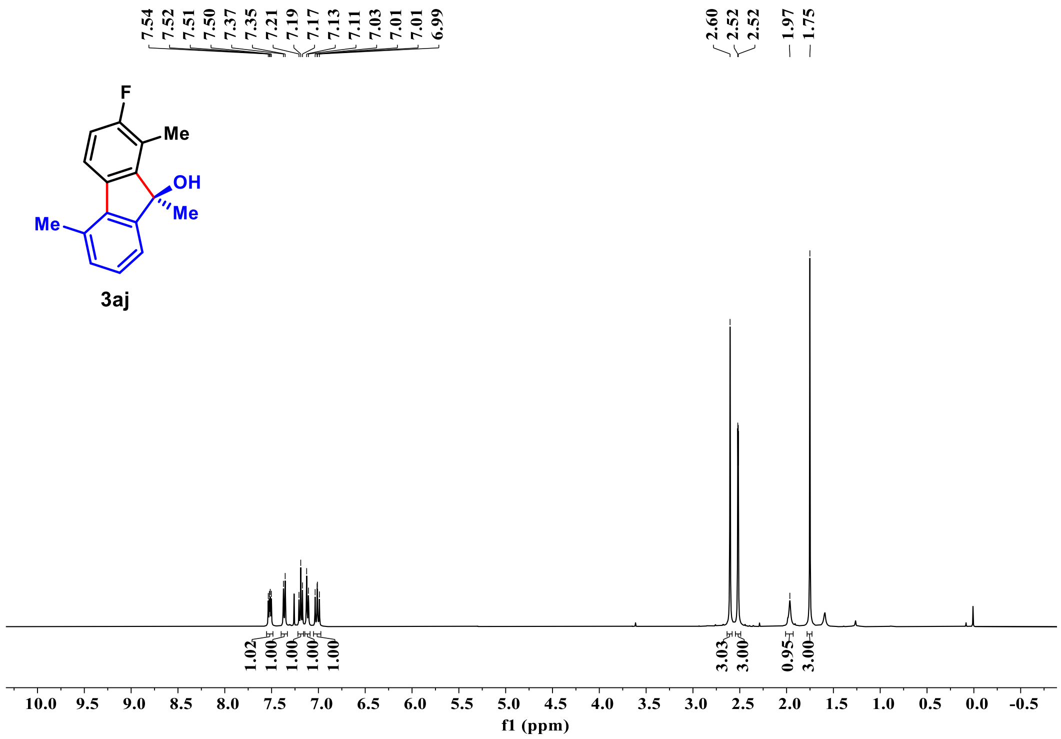

^{13}C NMR (100 MHz, $CDCl_3$)

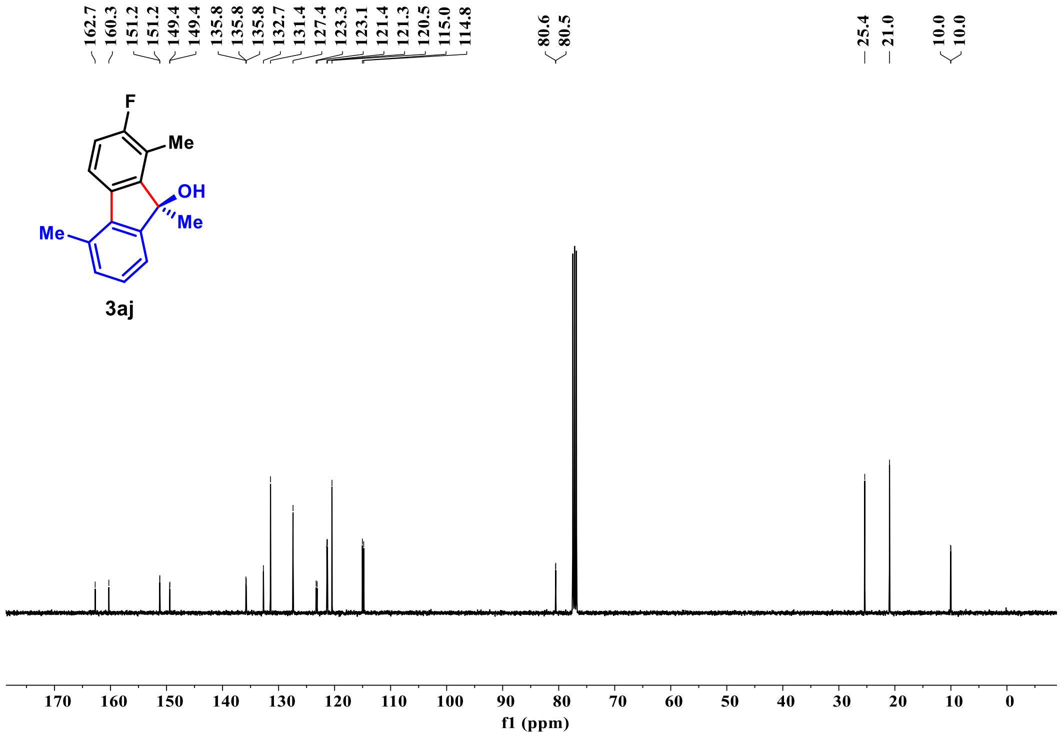

^{19}F NMR (376 MHz, $CDCl_3$)

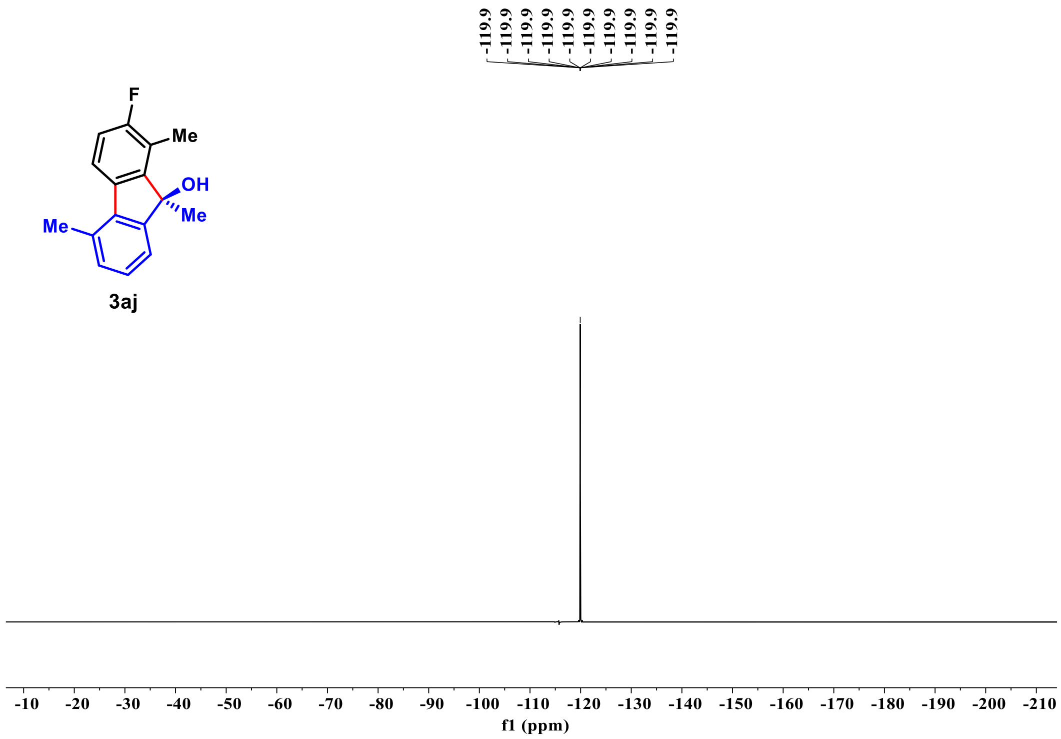

^{1}H NMR (400 MHz, $CDCl_3$)

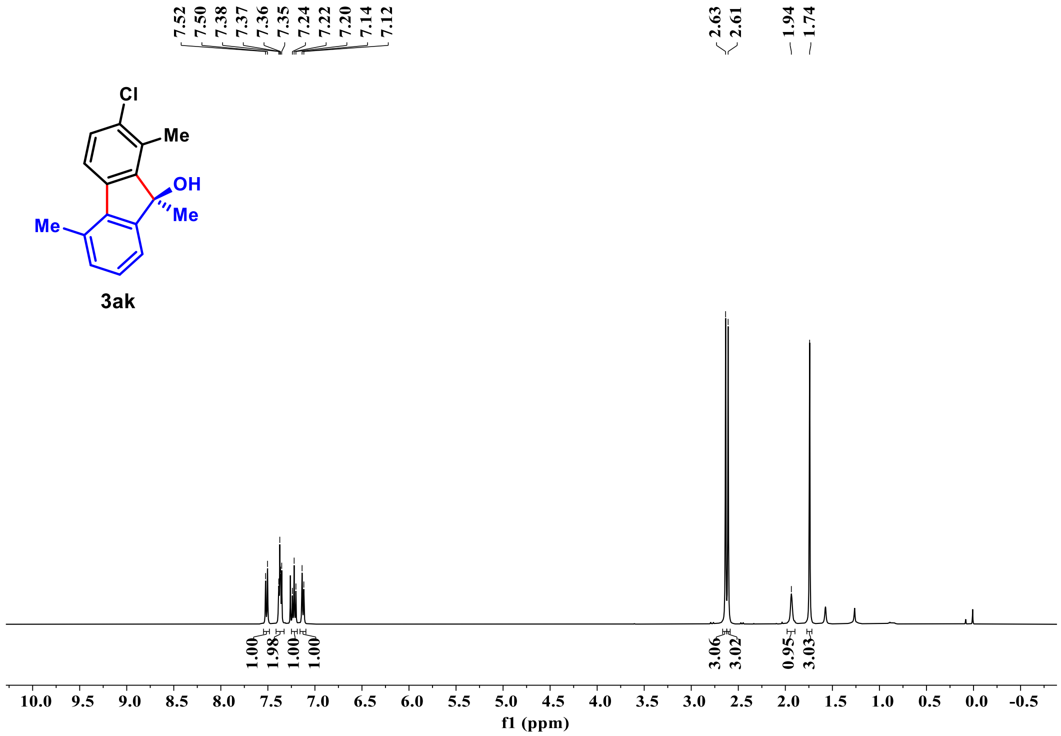

^{13}C NMR (100 MHz, $CDCl_3$)

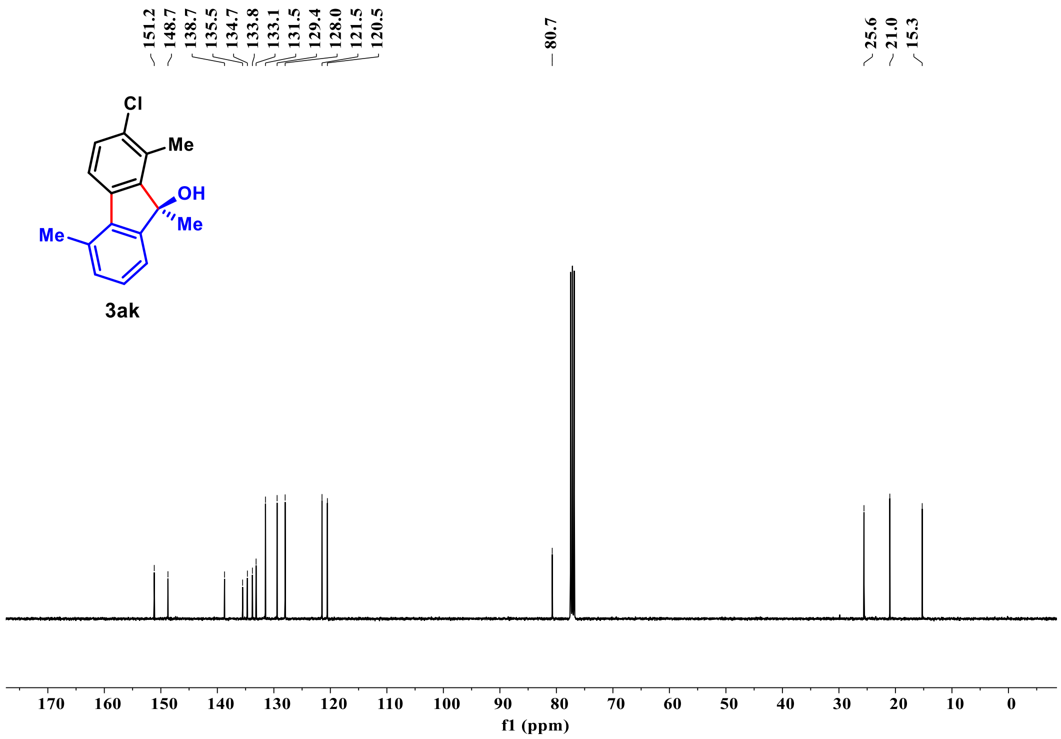

^{1}H NMR (400 MHz, $CDCl_3$)

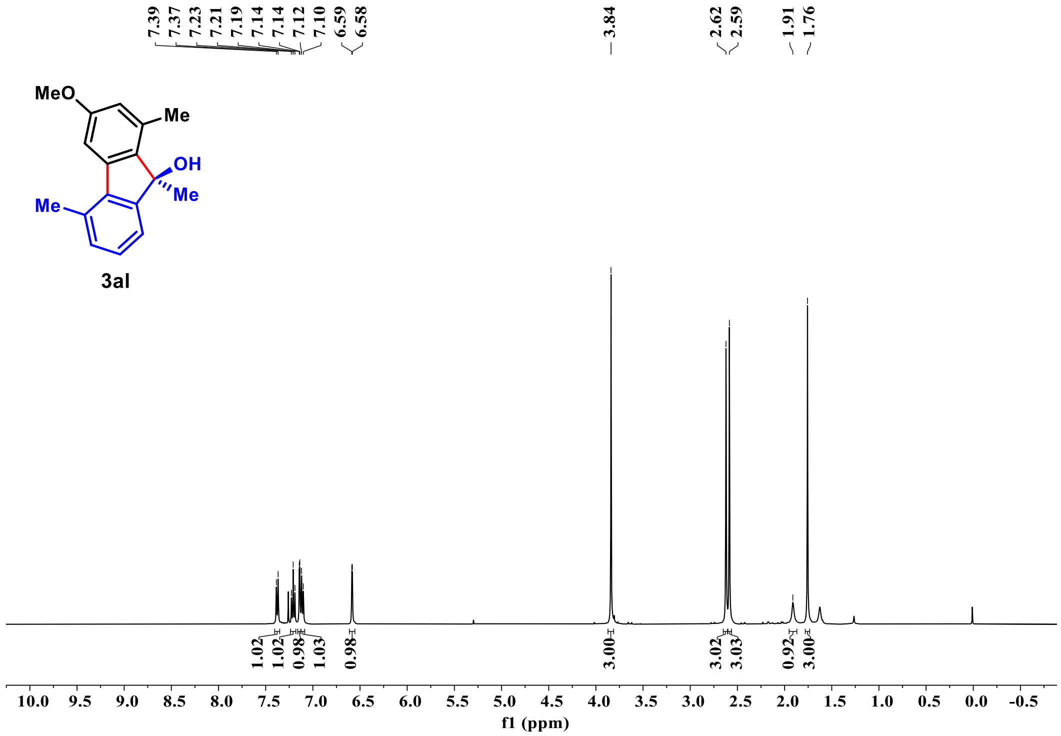

^{13}C NMR (100 MHz, $CDCl_3$)

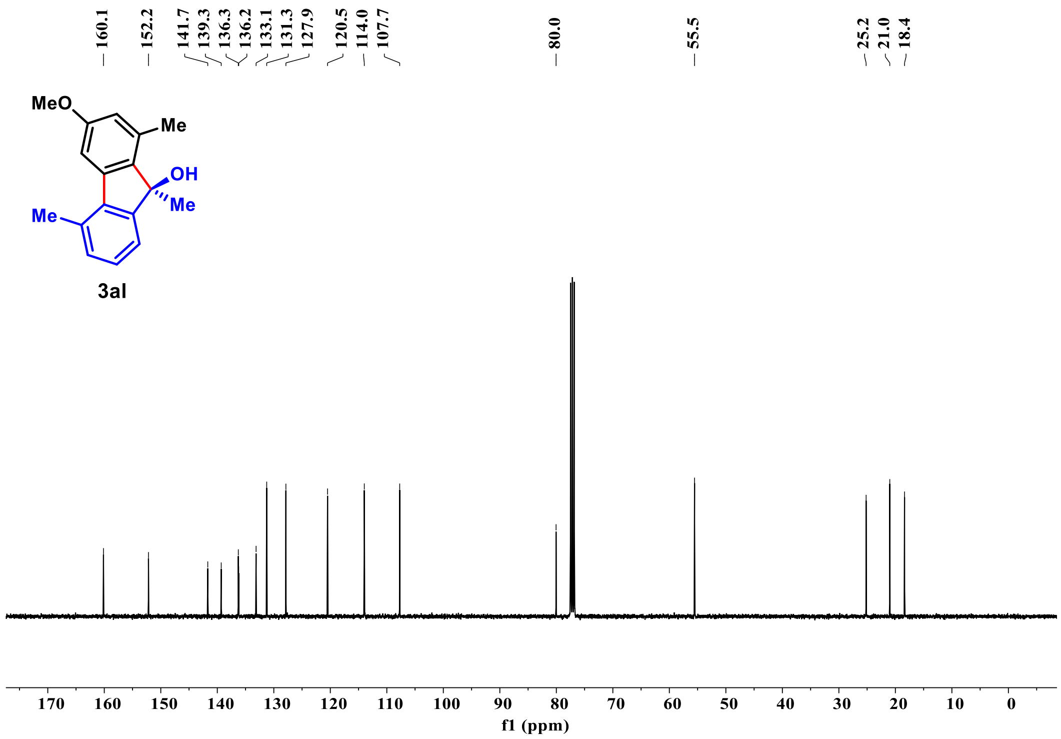

3al

^{1}H NMR (400 MHz, $CDCl_3$)

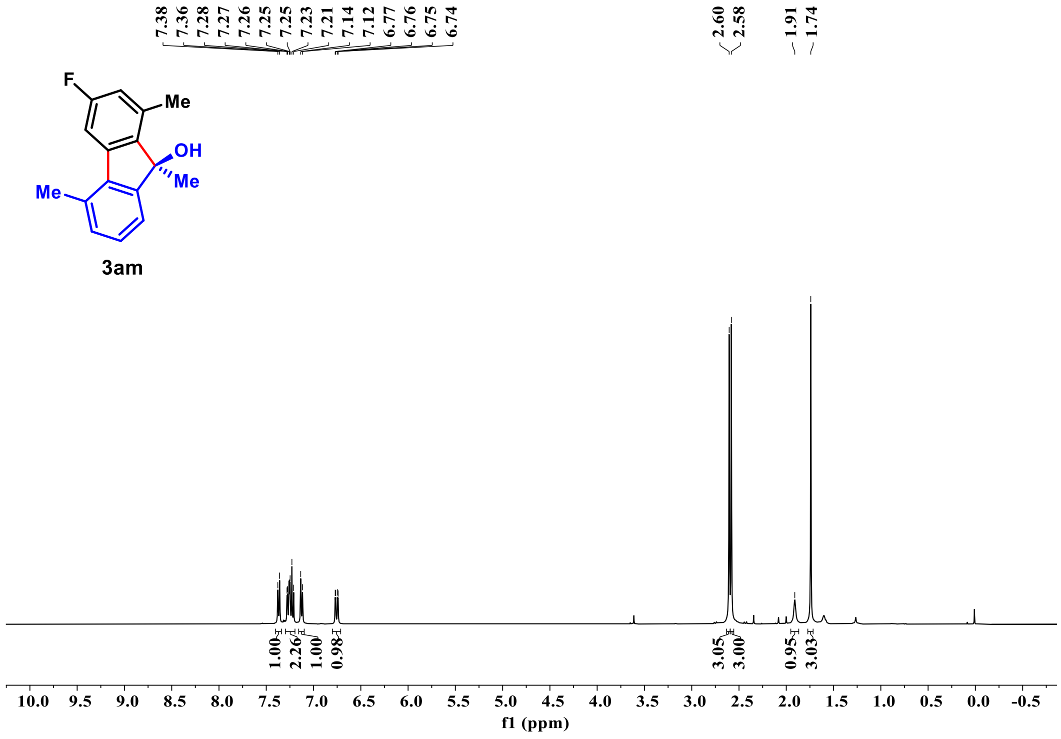

3am

^{13}C NMR (100 MHz, $CDCl_3$)

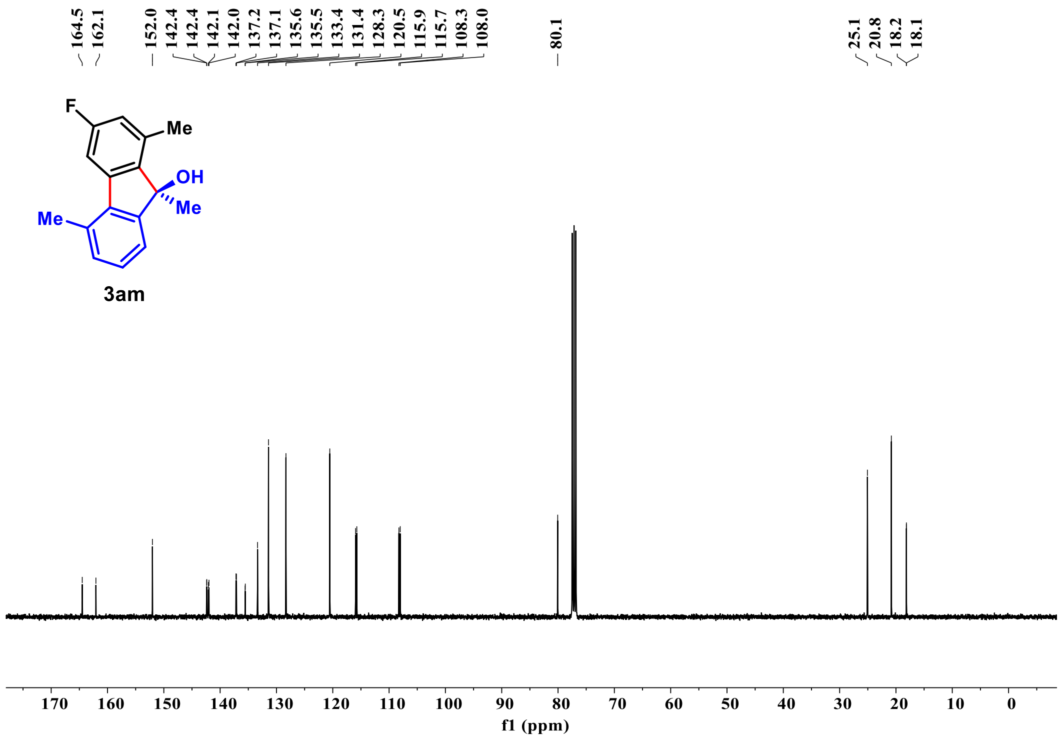

^{19}F NMR (376 MHz, $CDCl_3$)

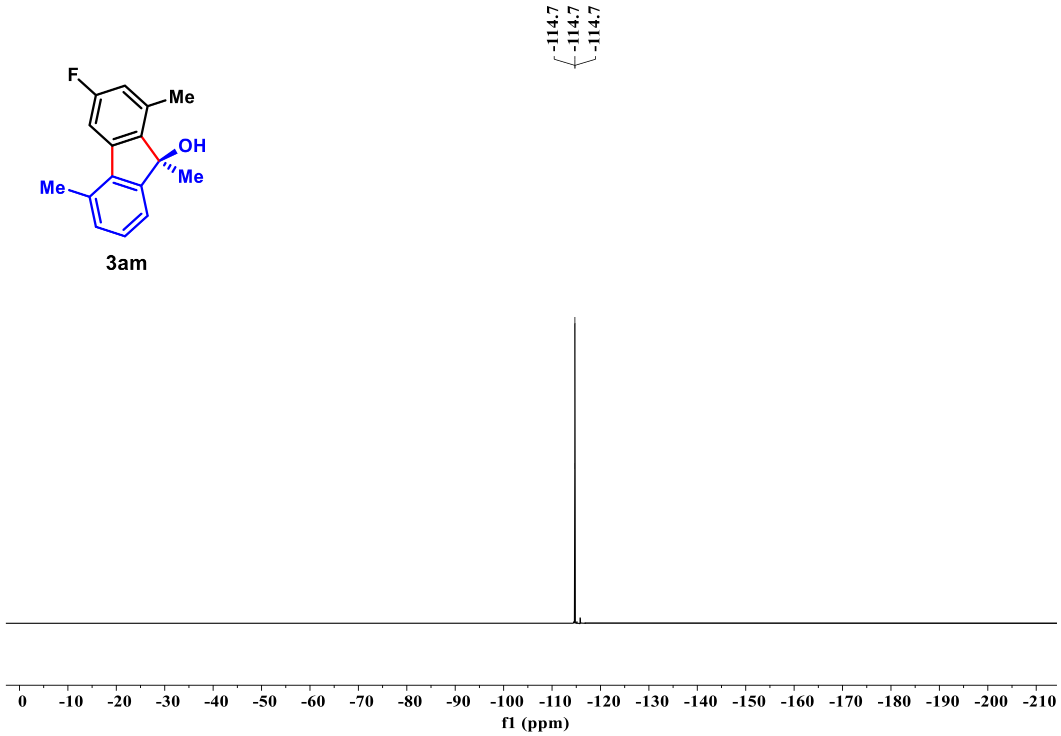

^{1}H NMR (400 MHz, $CDCl_3$)

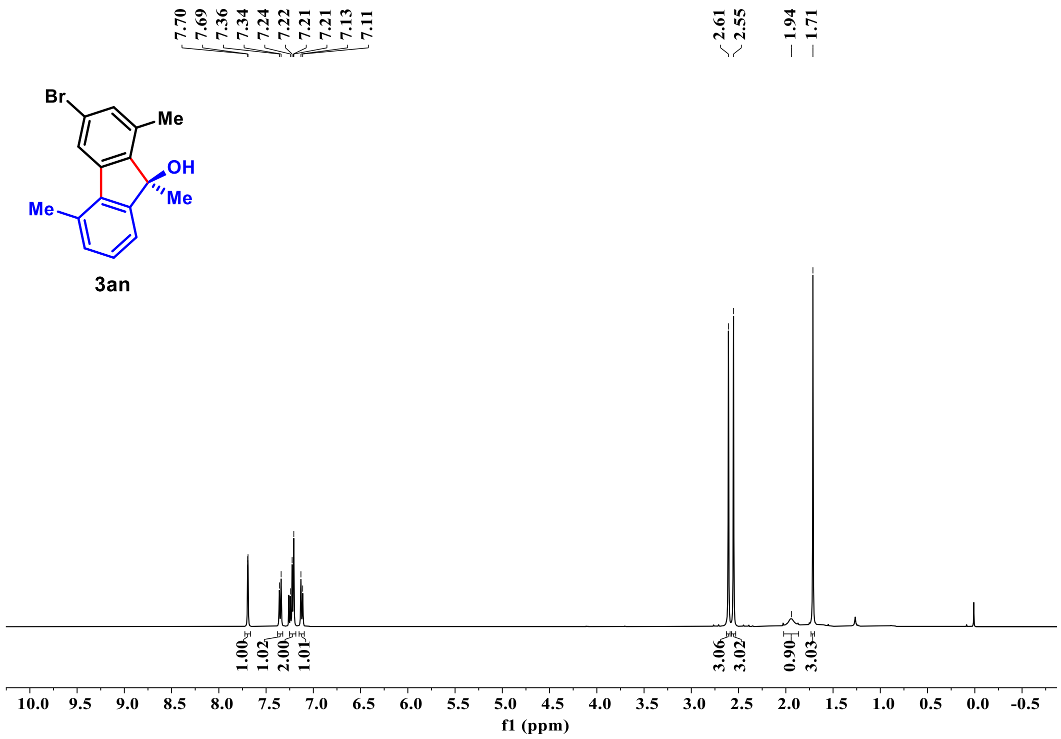

^{13}C NMR (100 MHz, $CDCl_3$)

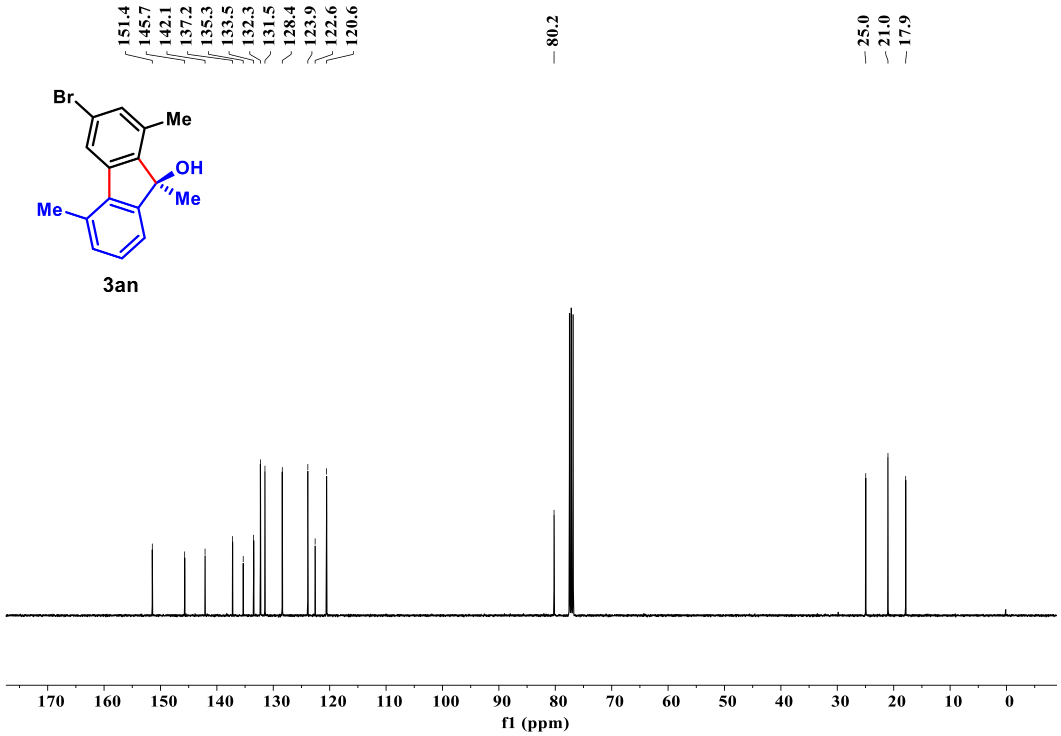

^{1}H NMR (400 MHz, $CDCl_3$)

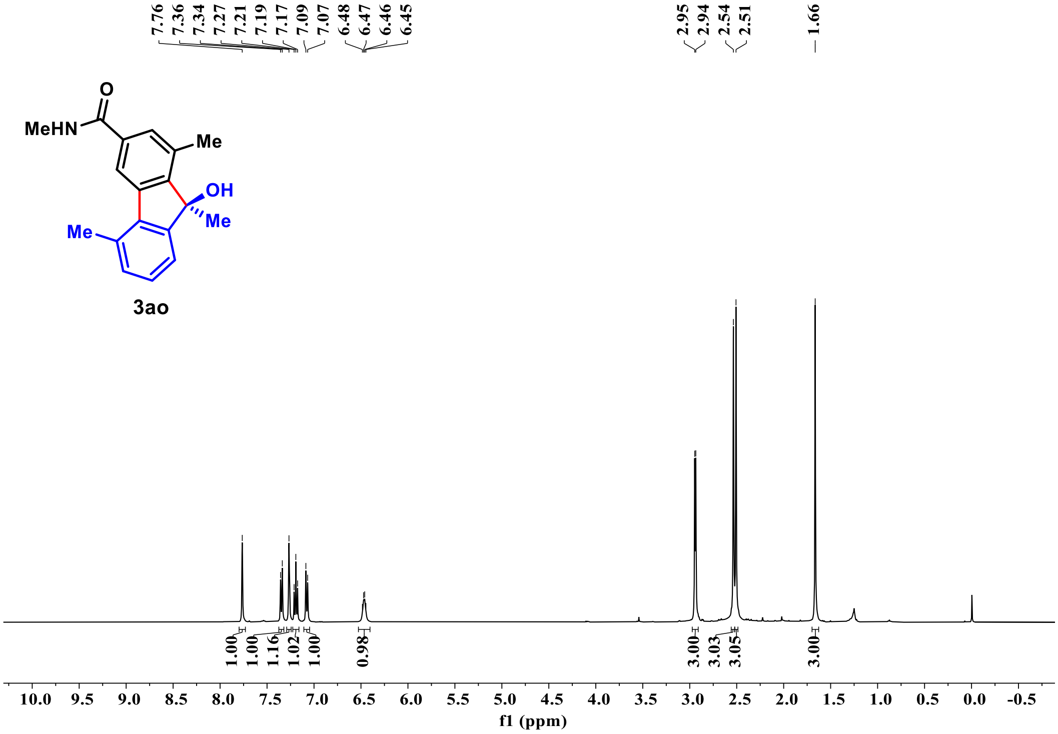

^{13}C NMR (100 MHz, $CDCl_3$)

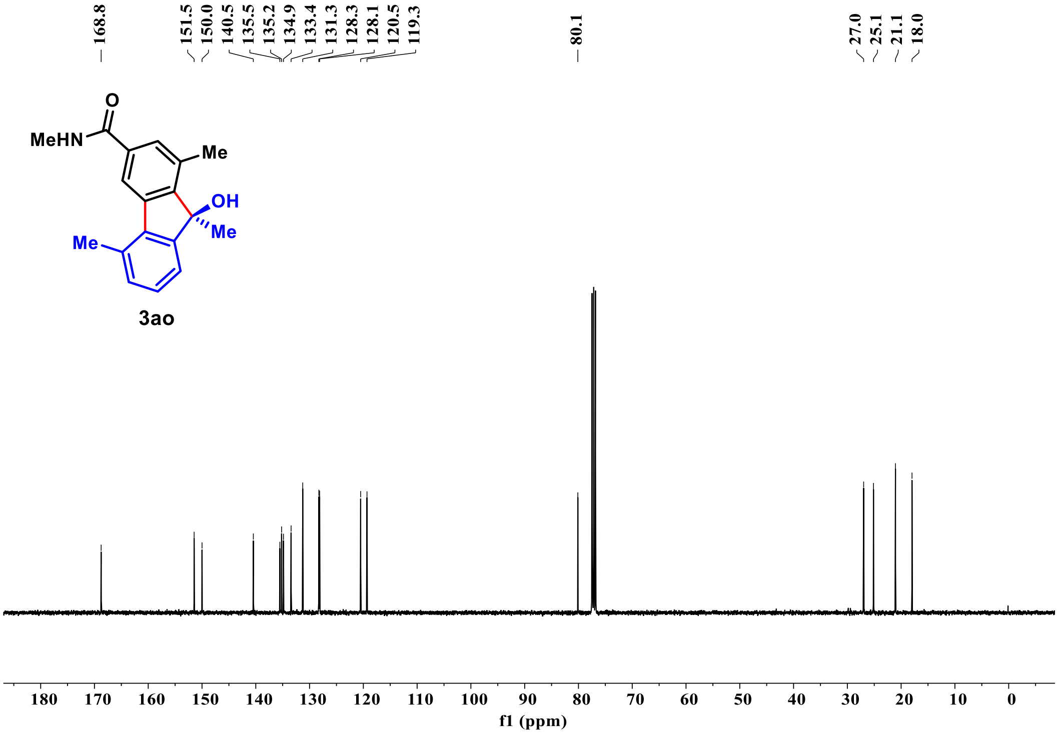

^{1}H NMR (400 MHz, $CDCl_3$)

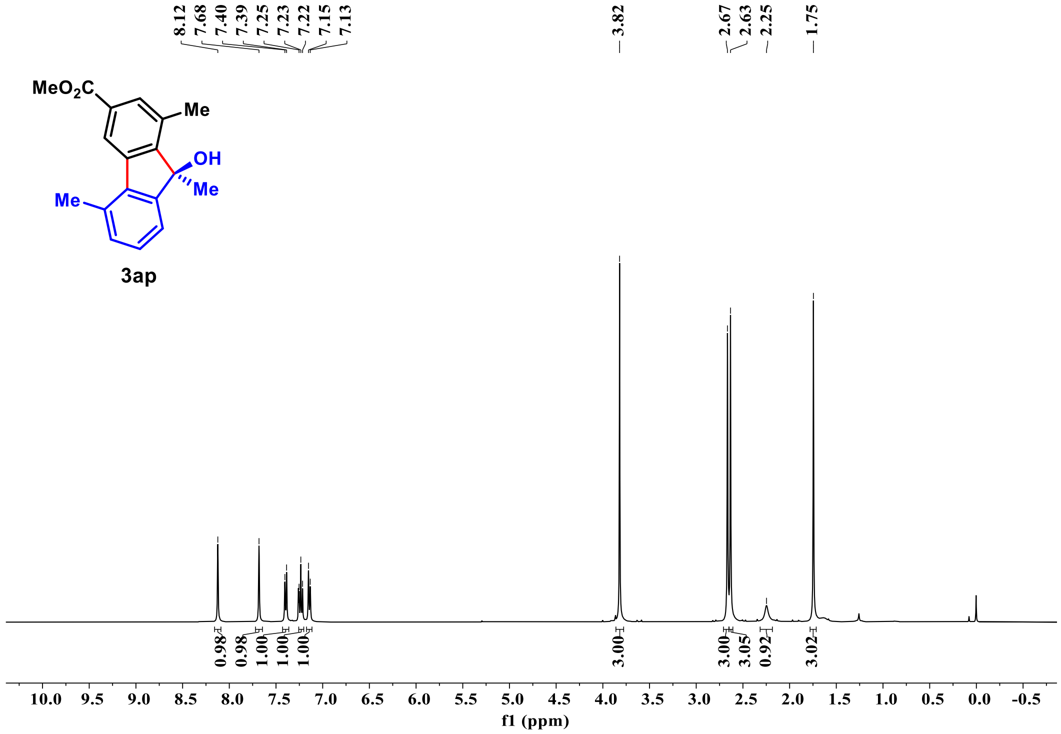

^{13}C NMR (100 MHz, $CDCl_3$)

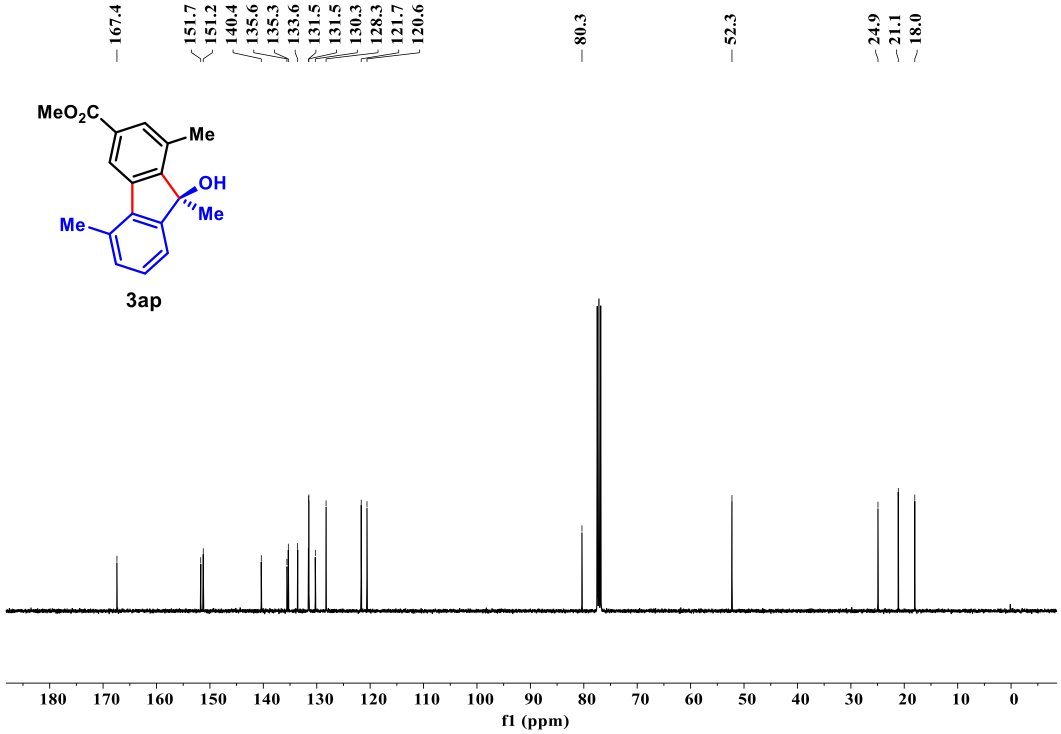

^{1}H NMR (400 MHz, $CDCl_3$)

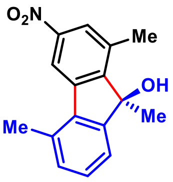

3aq

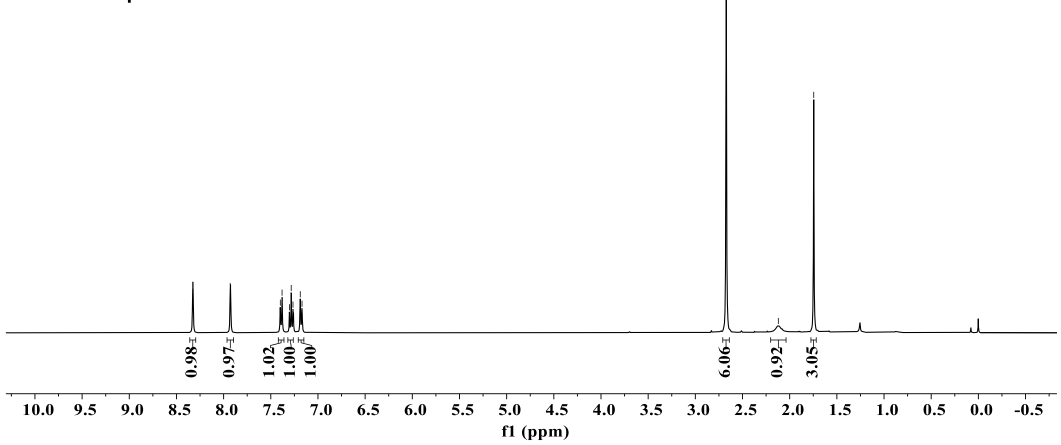

^{13}C NMR (100 MHz, $CDCl_3$)

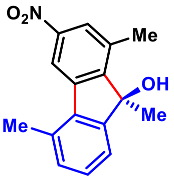

3aq

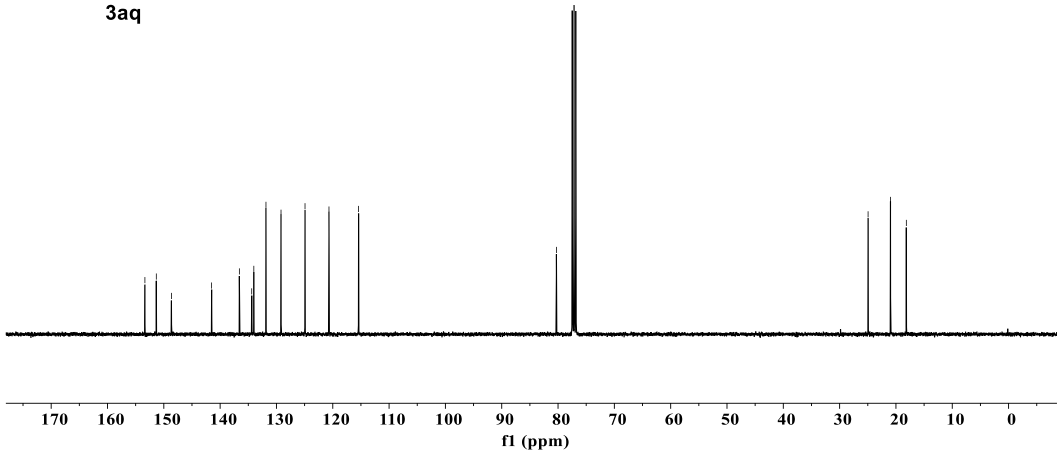

^{1}H NMR (400 MHz, CDCl$_3$)

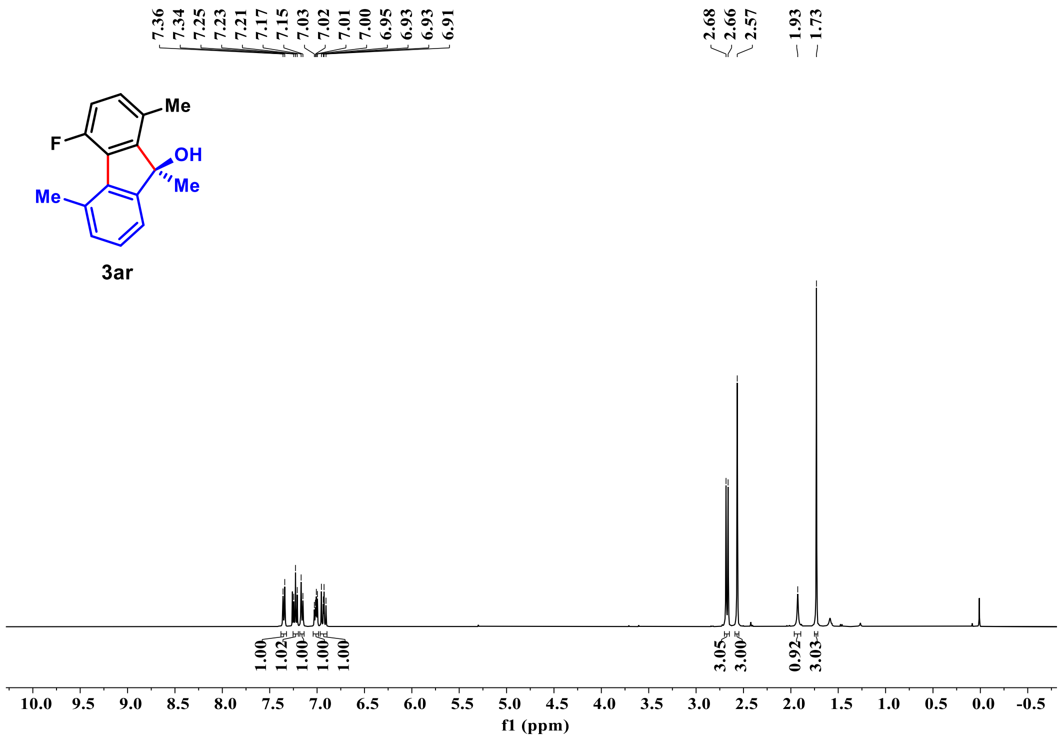

^{13}C NMR (100 MHz, CDCl$_3$)

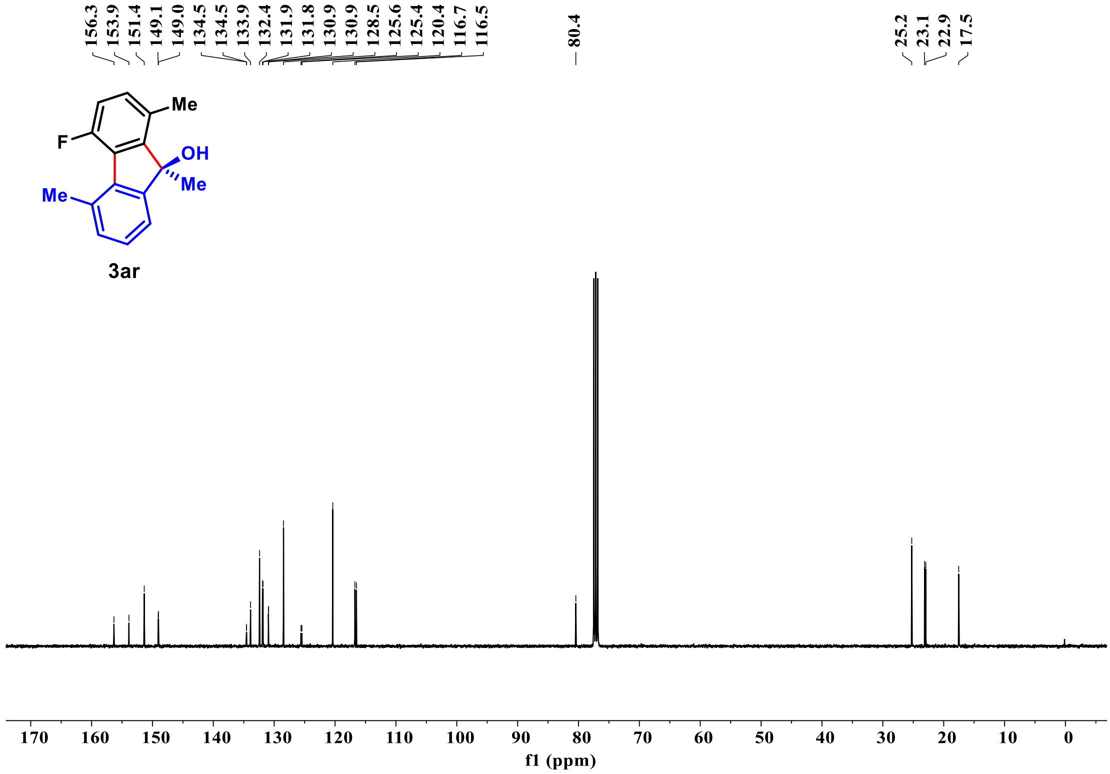

^{19}F NMR (376 MHz, $CDCl_3$)

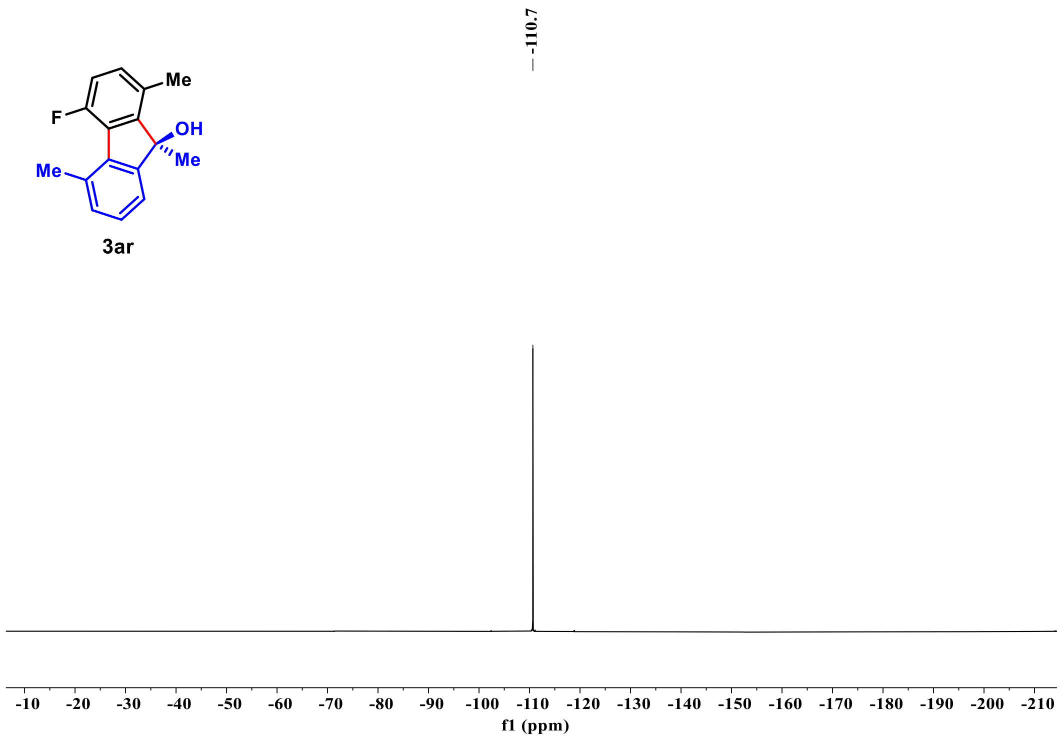

^{1}H NMR (400 MHz, $CDCl_3$)

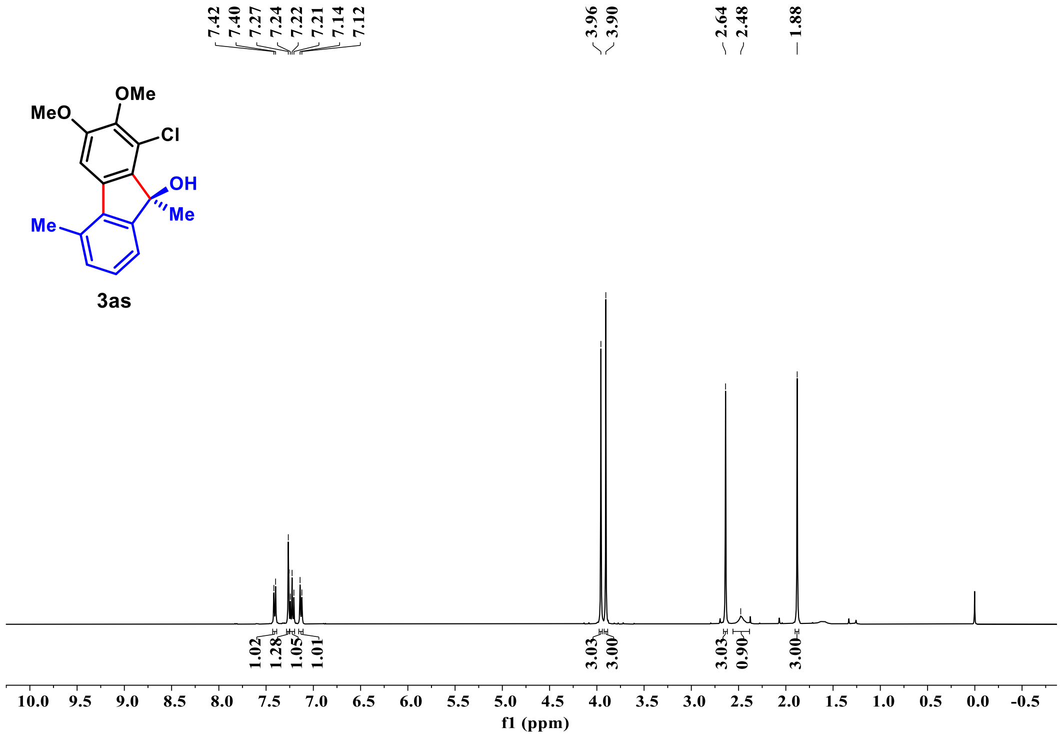

^{13}C NMR (100 MHz, $CDCl_3$)

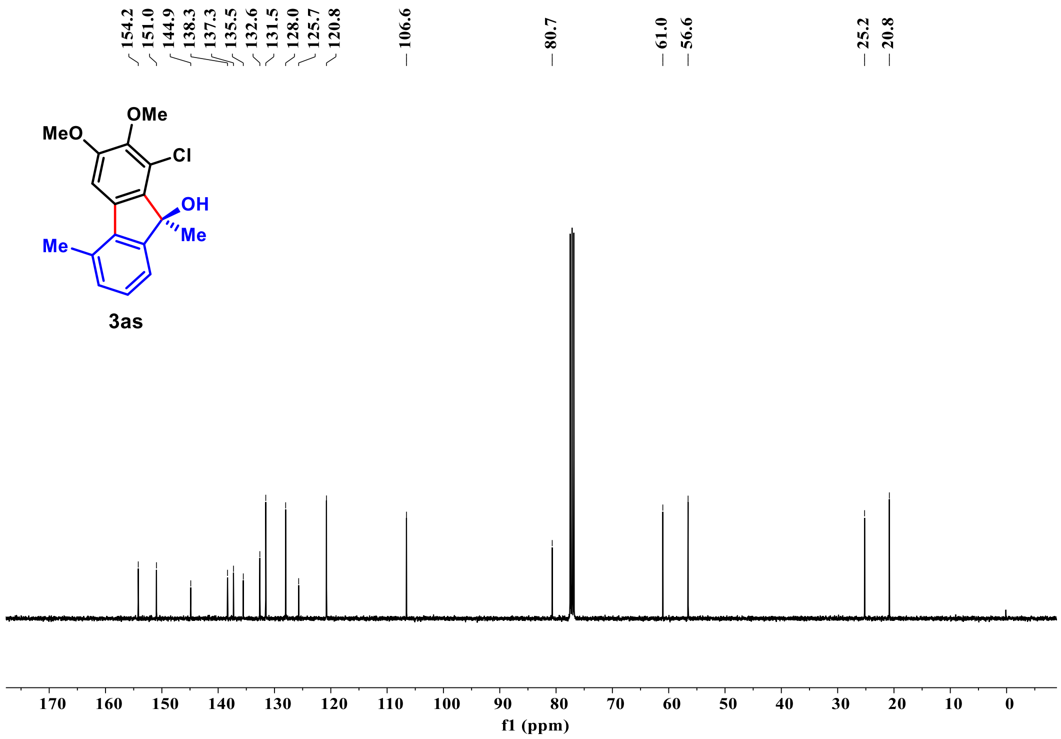

^{1}H NMR (400 MHz, $CDCl_3$)

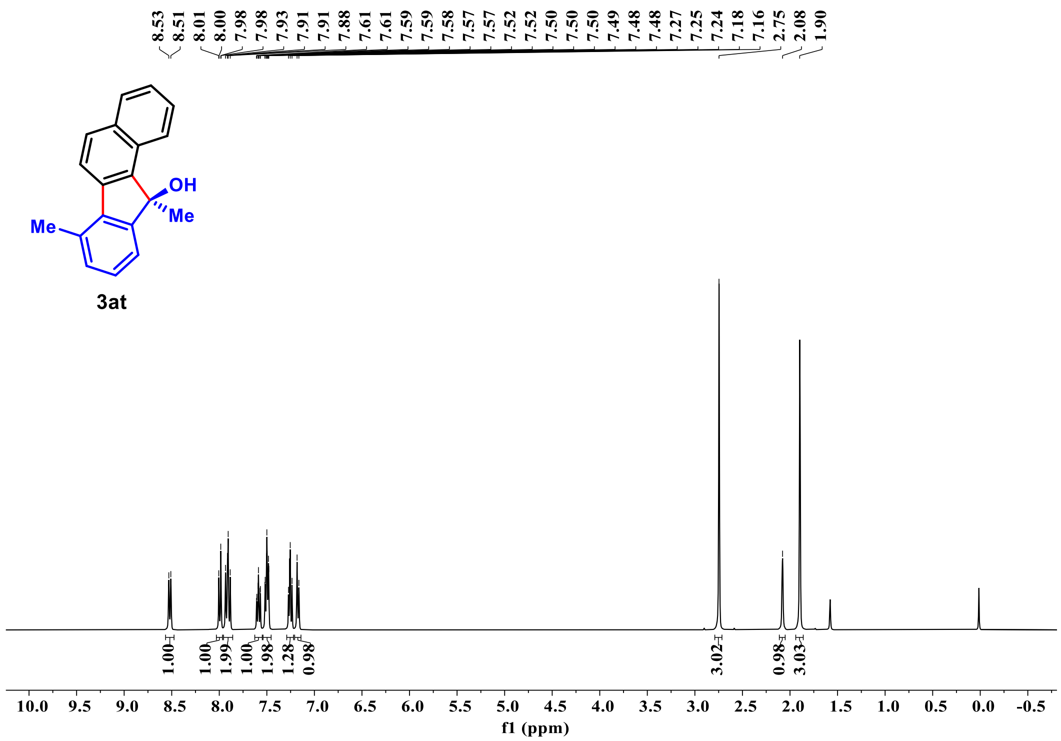

^{13}C NMR (100 MHz, $CDCl_3$)

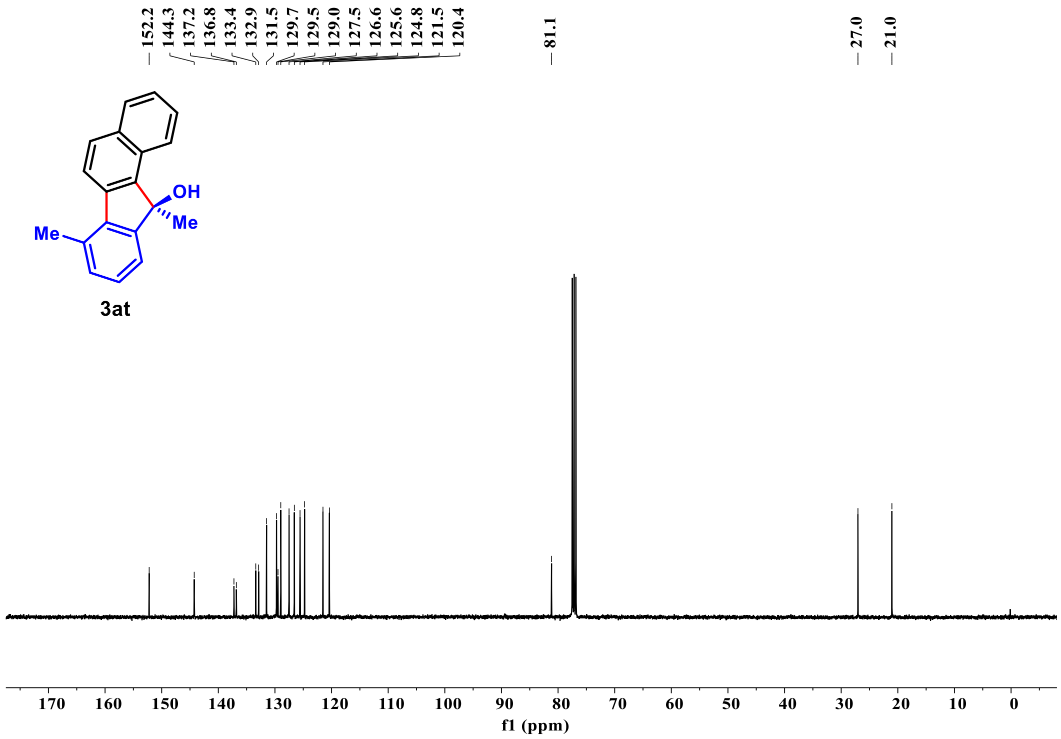

^{1}H NMR (400 MHz, $CDCl_3$)

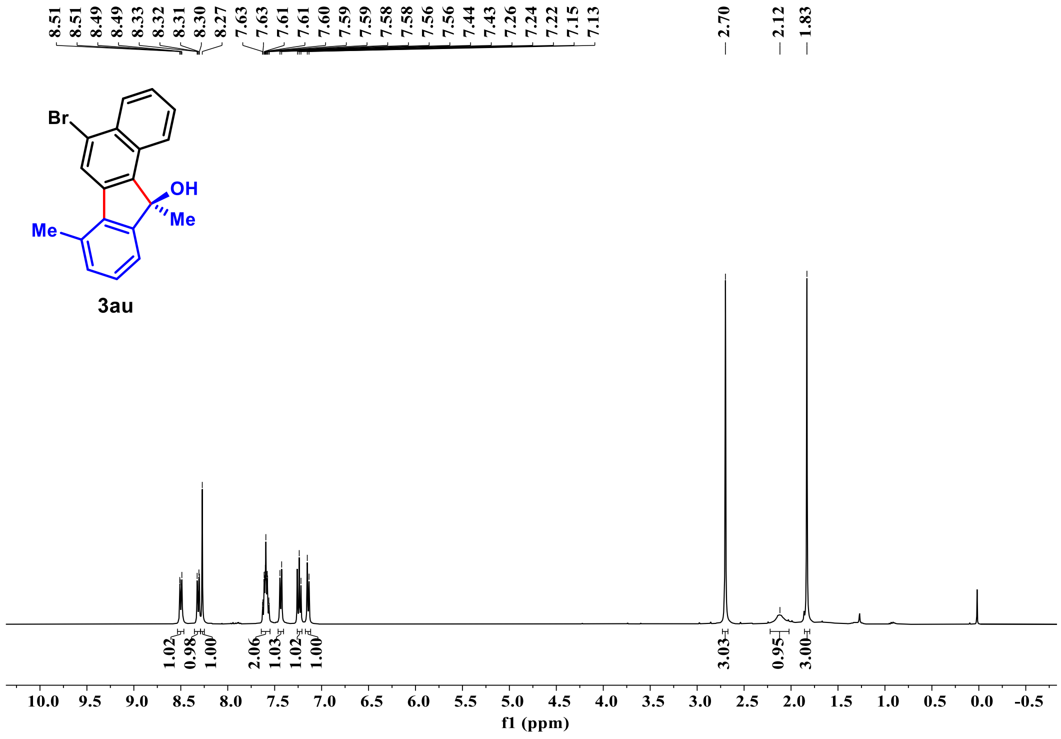

^{13}C NMR (100 MHz, $CDCl_3$)

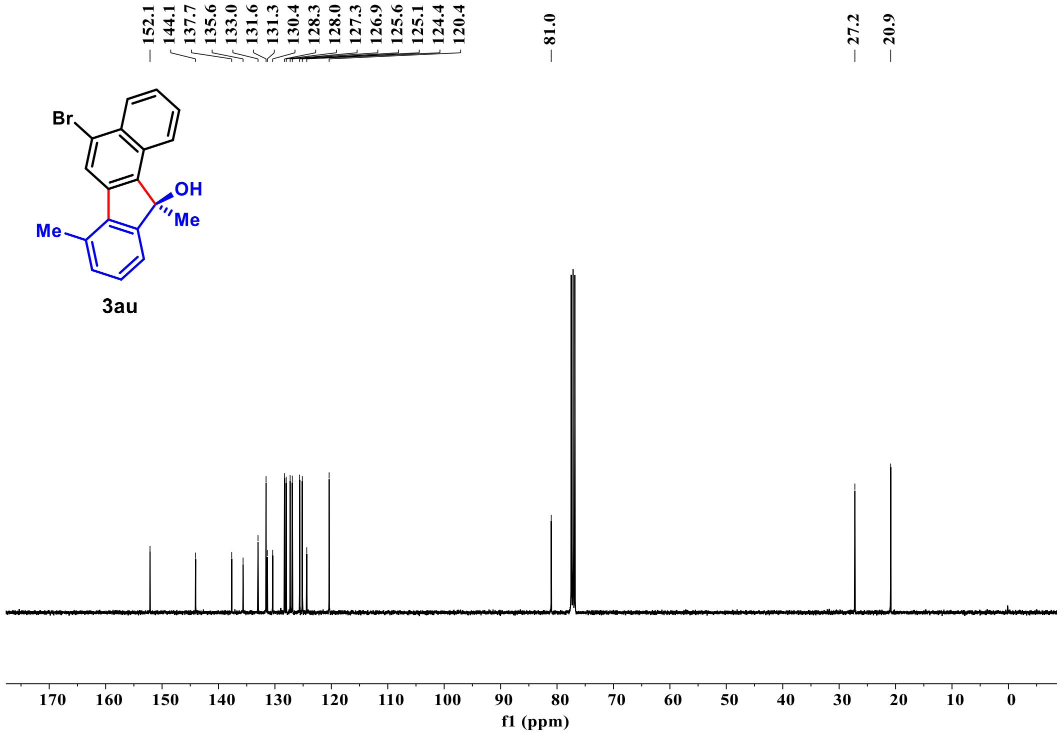

^{1}H NMR (400 MHz, $CDCl_3$)

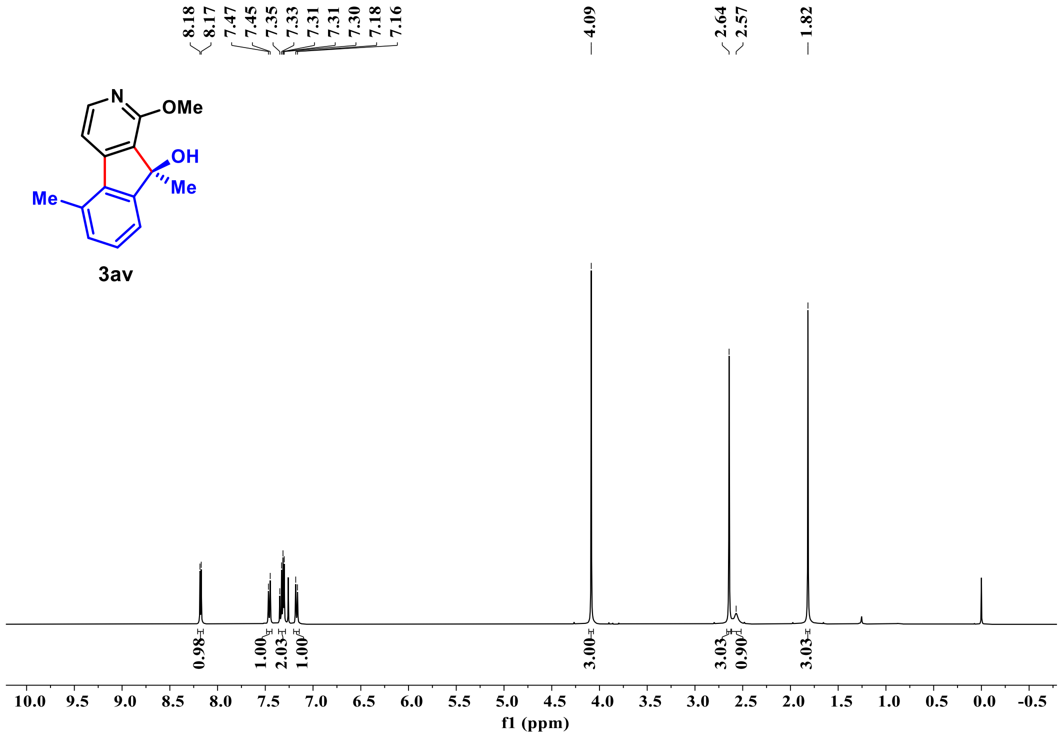

^{13}C NMR (100 MHz, $CDCl_3$)

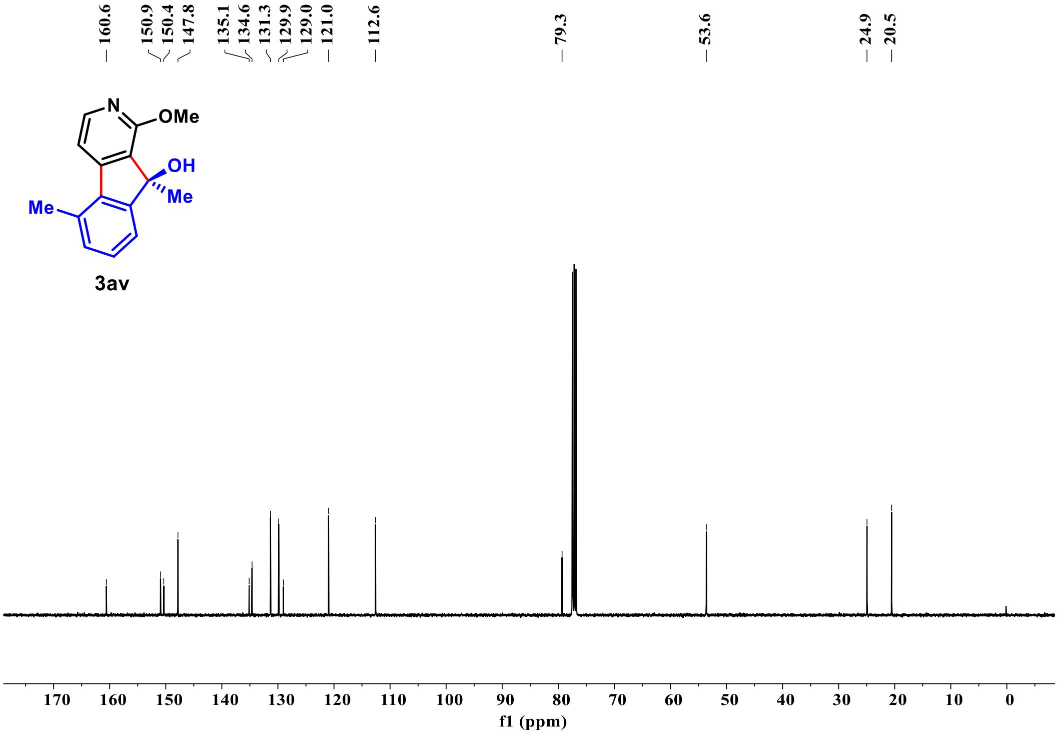

^{1}H NMR (400 MHz, $CDCl_3$)

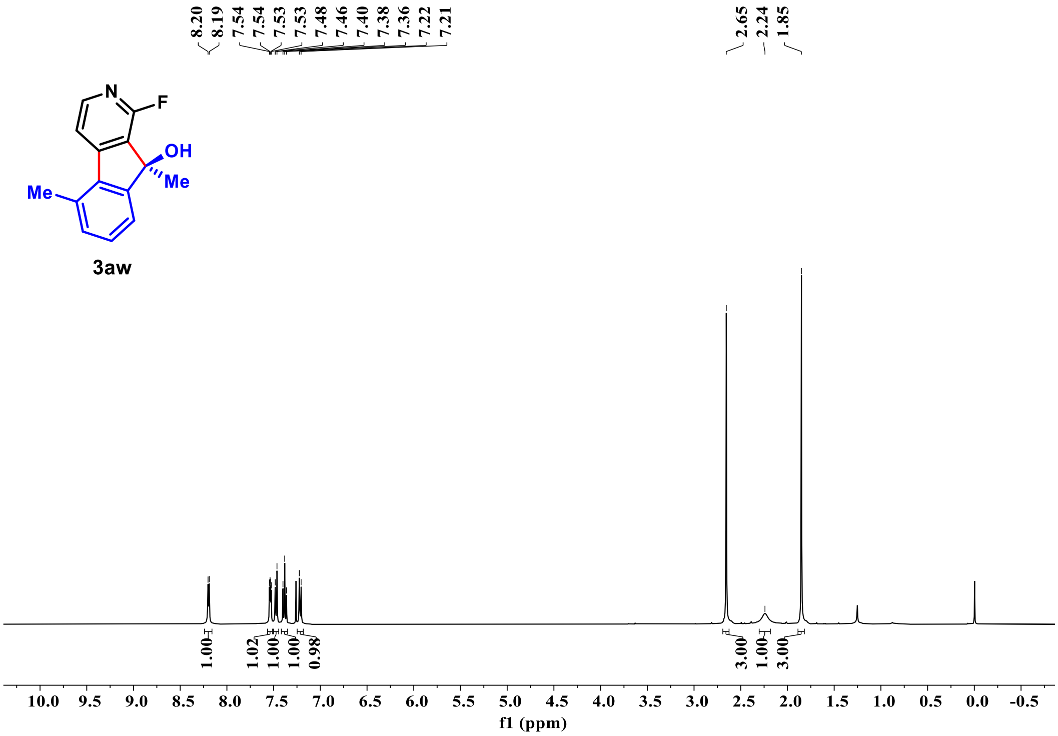

^{13}C NMR (100 MHz, $CDCl_3$)

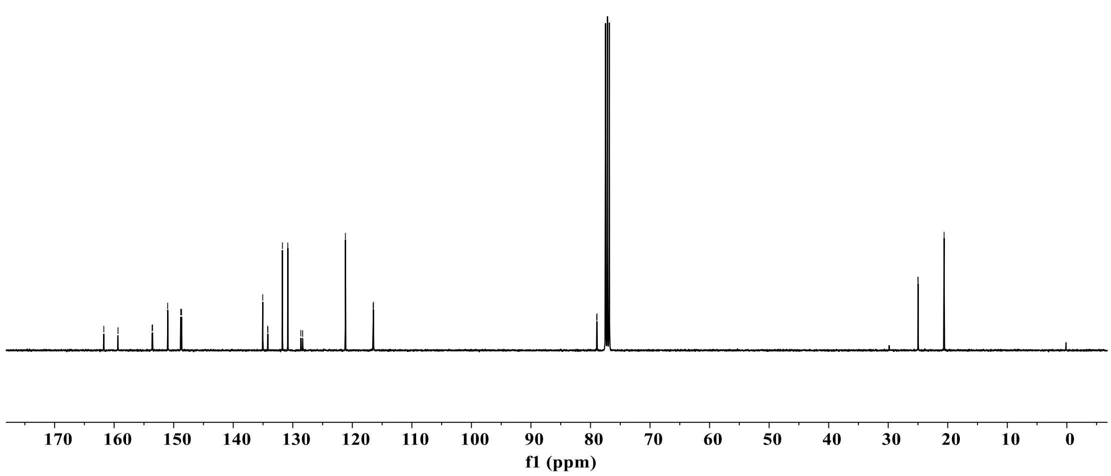

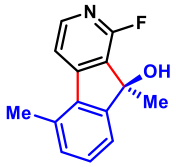

3aw

^{19}F NMR (376 MHz, $CDCl_3$)

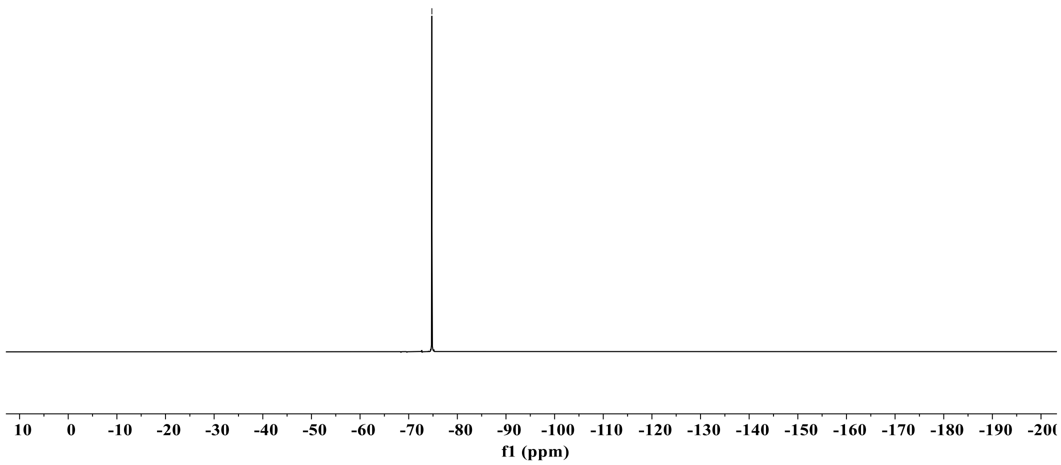

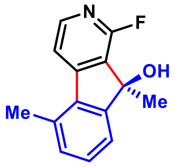

3aw

^{1}H NMR (400 MHz, $CDCl_3$)

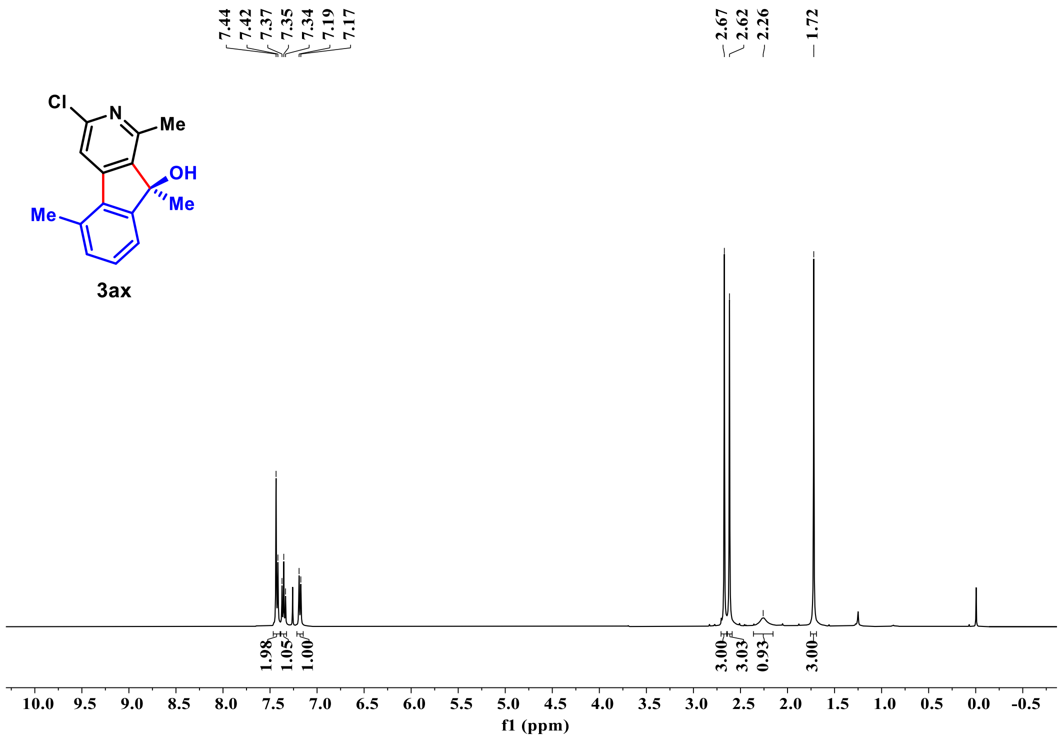

^{13}C NMR (100 MHz, $CDCl_3$)

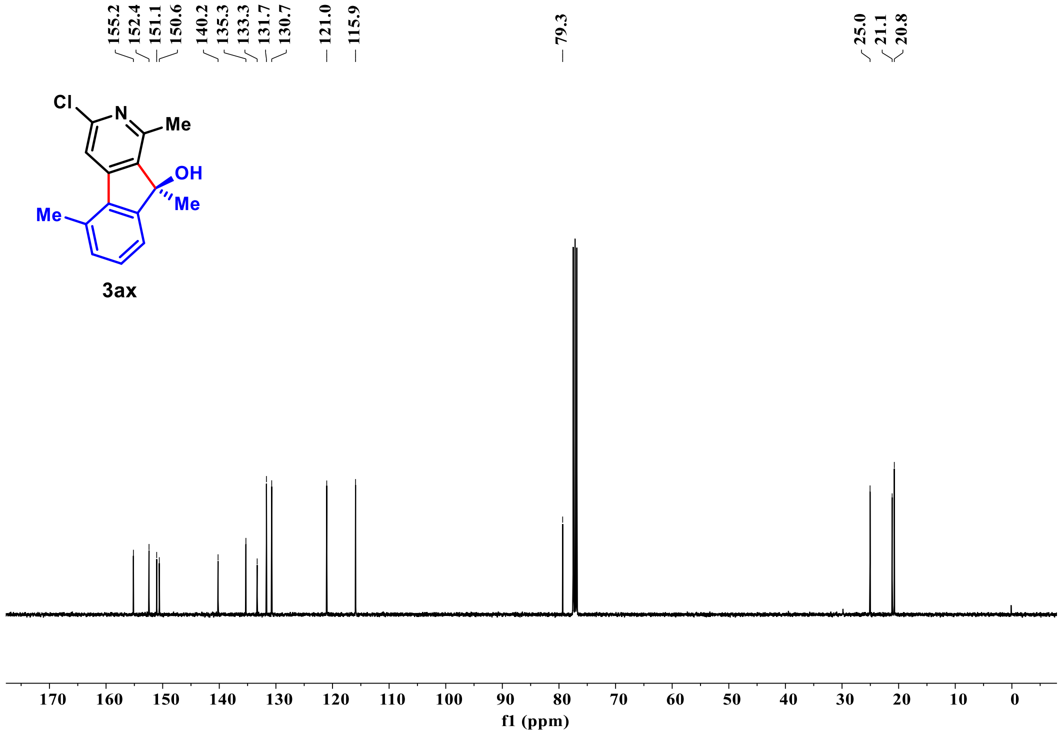

^{1}H NMR (400 MHz, $CDCl_3$)

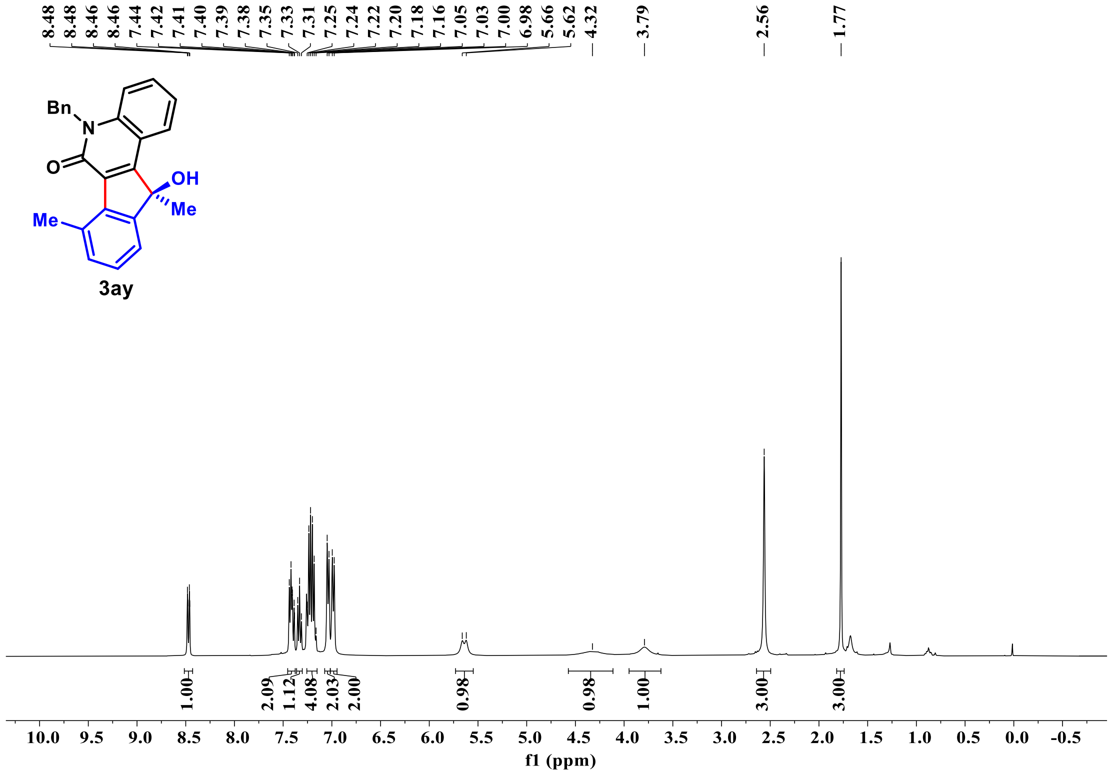

^{13}C NMR (100 MHz, $CDCl_3$)

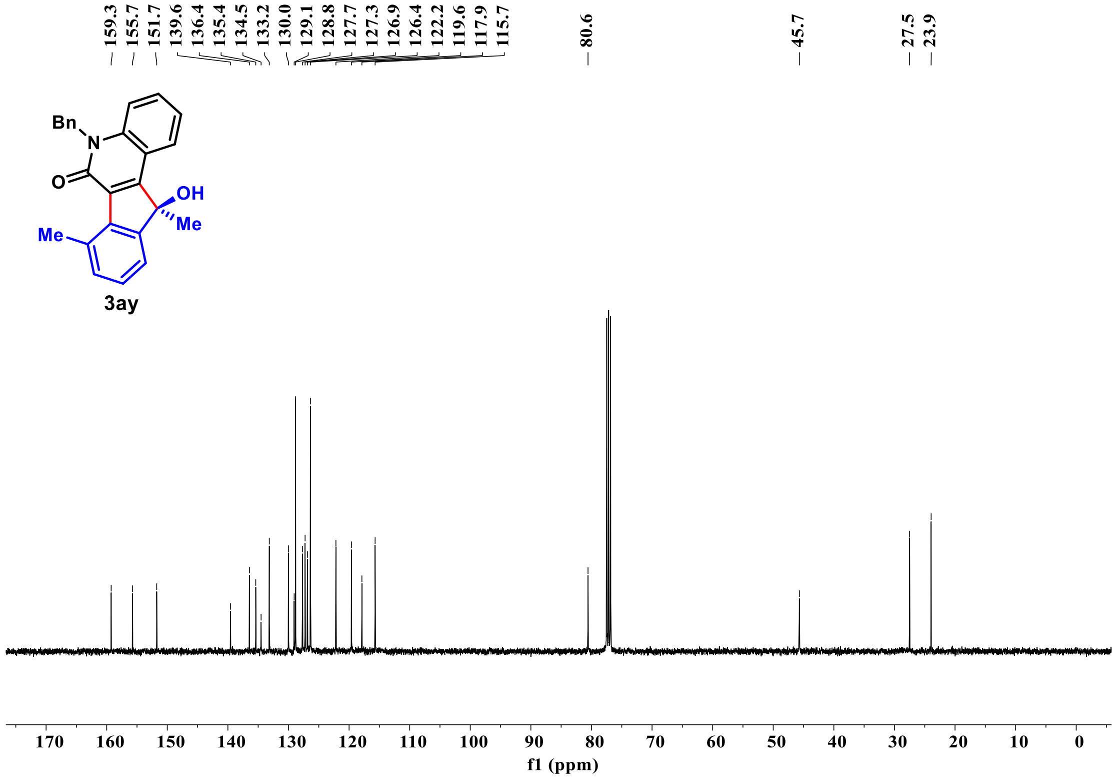

^{1}H NMR (400 MHz, CDCl$_3$)

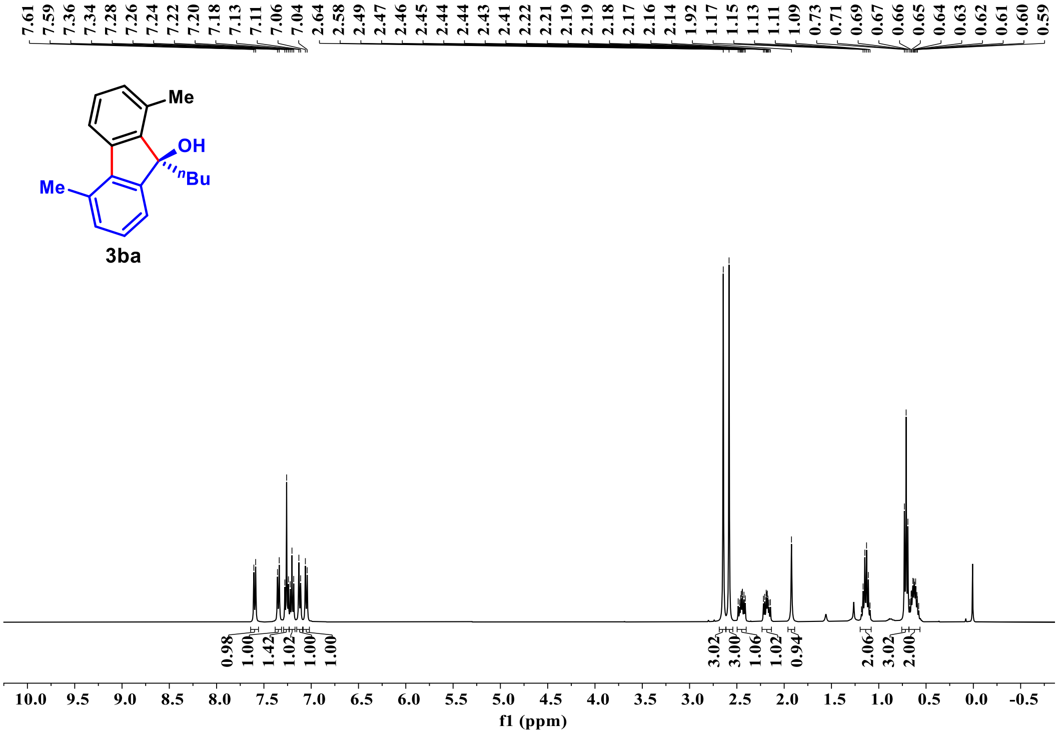

^{13}C NMR (100 MHz, CDCl$_3$)

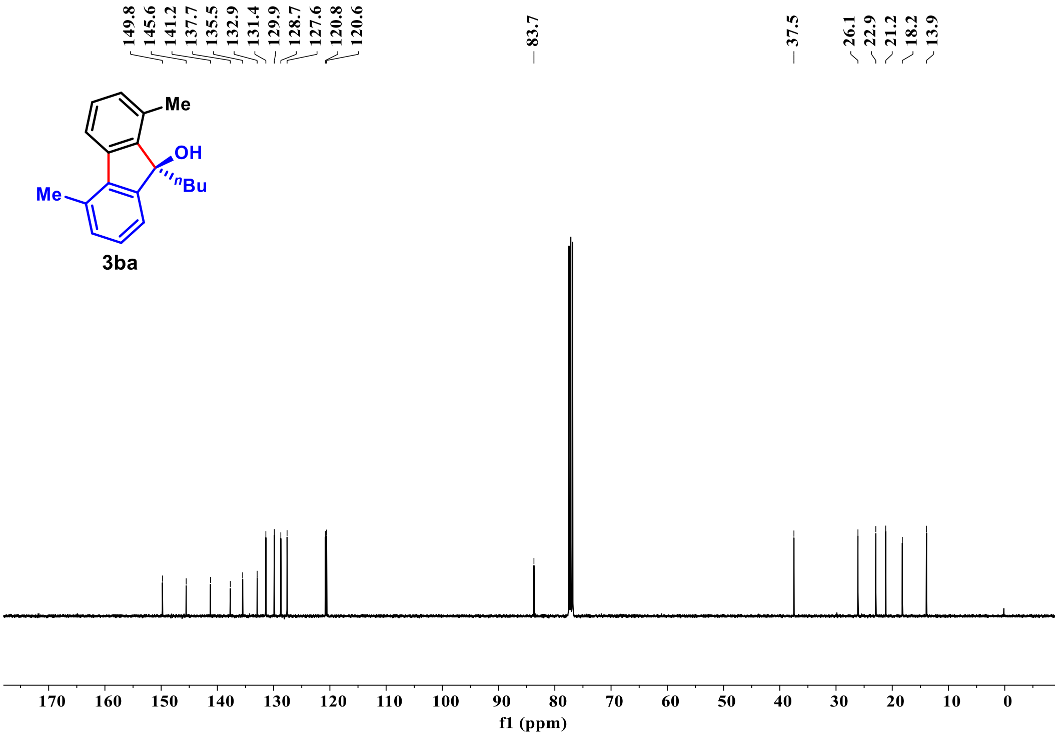

^{1}H NMR (400 MHz, $CDCl_3$)

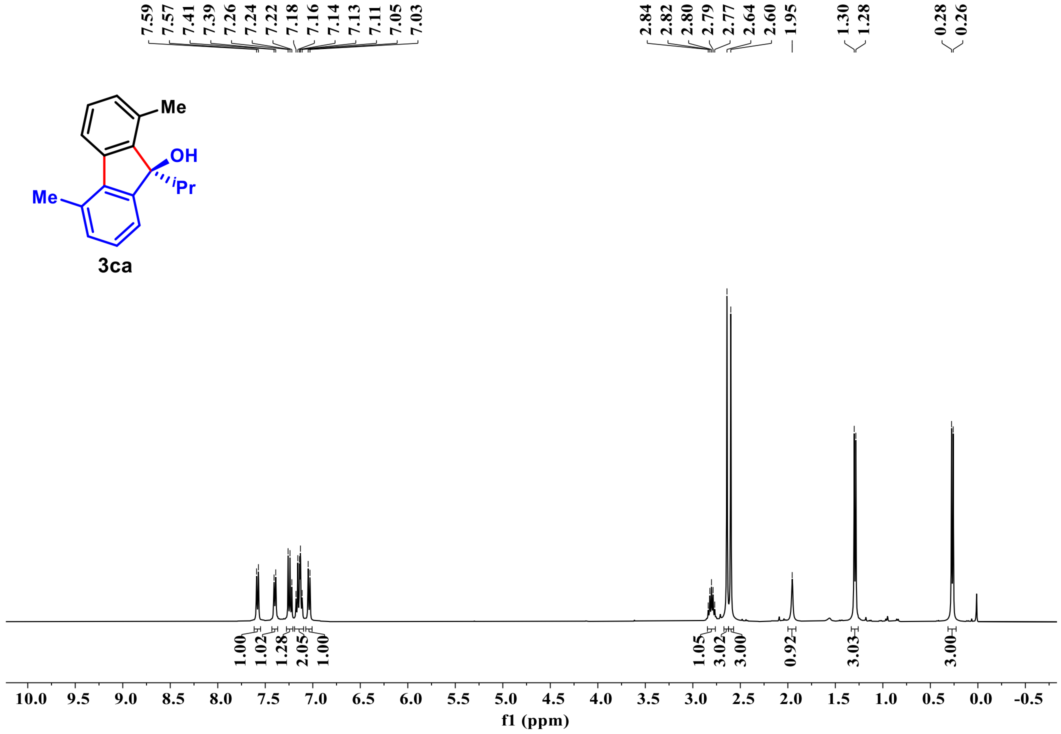

^{13}C NMR (100 MHz, $CDCl_3$)

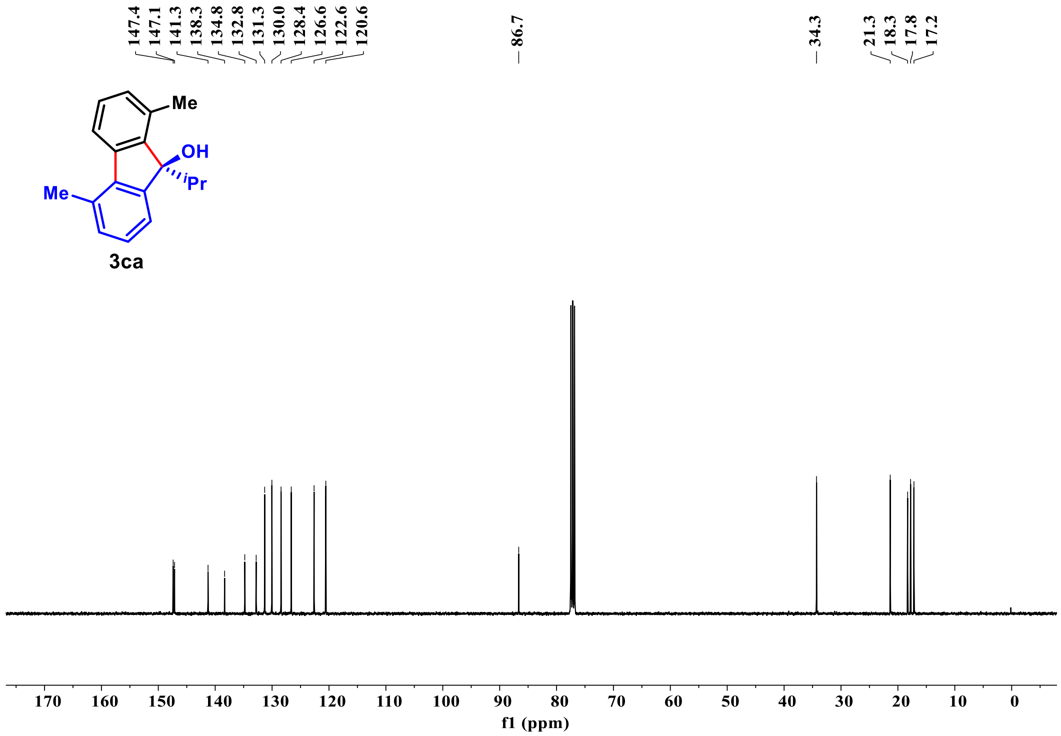

^{1}H NMR (400 MHz, CDCl$_3$)

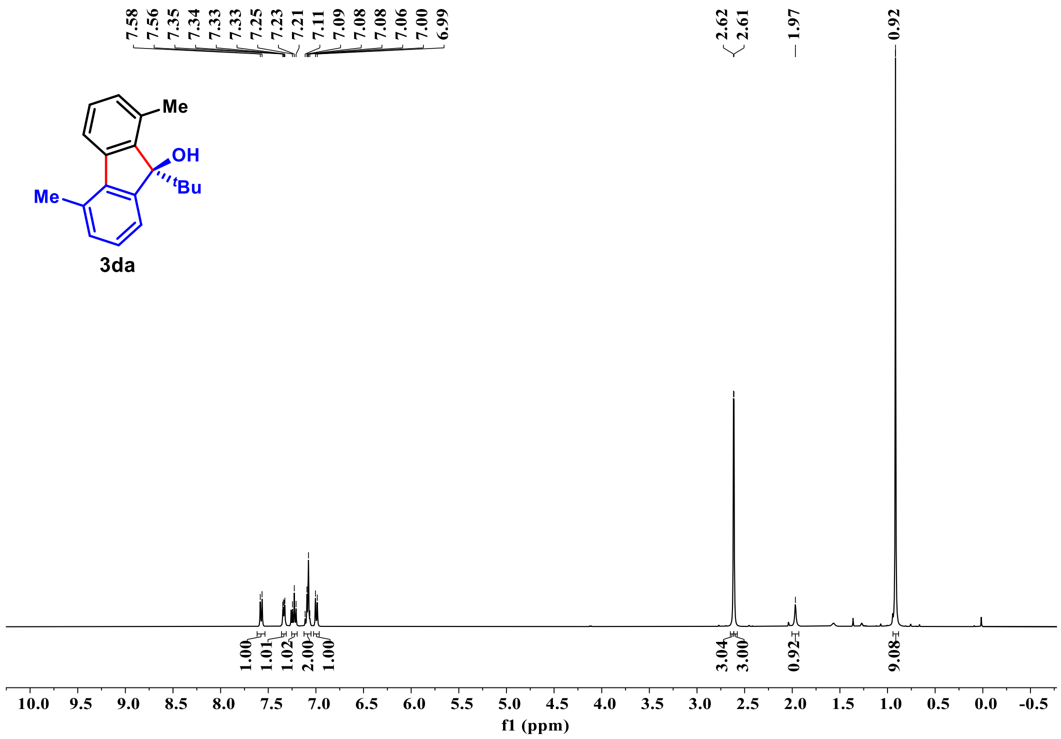

^{13}C NMR (100 MHz, CDCl$_3$)

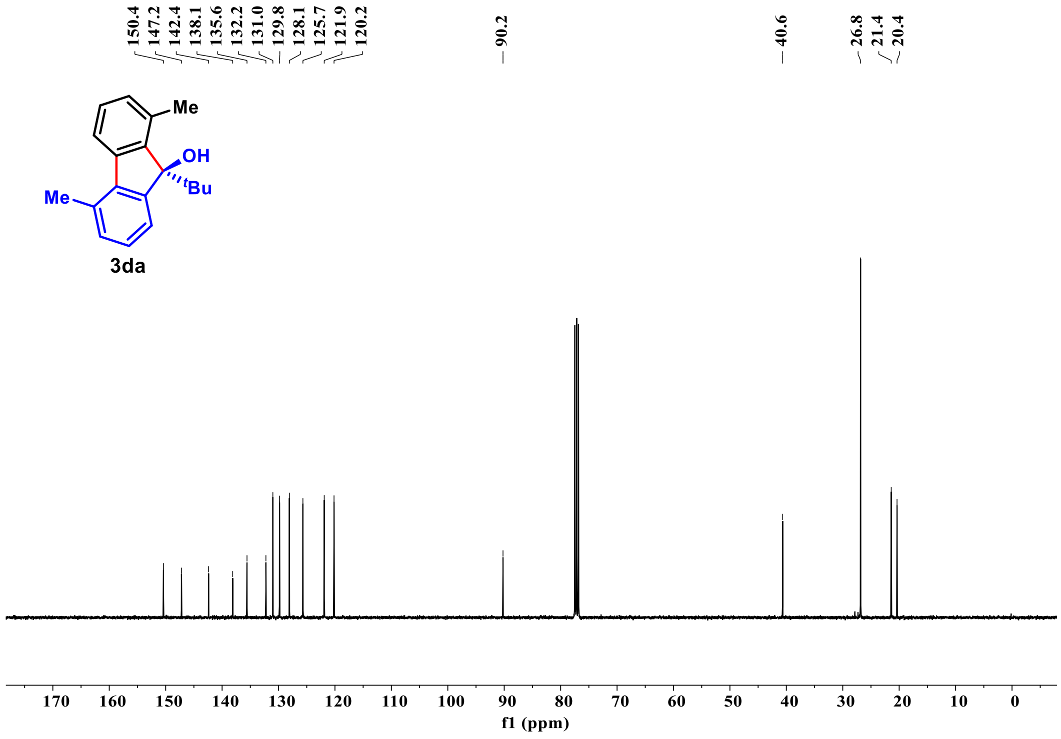

^{1}H NMR (400 MHz, $CDCl_3$)

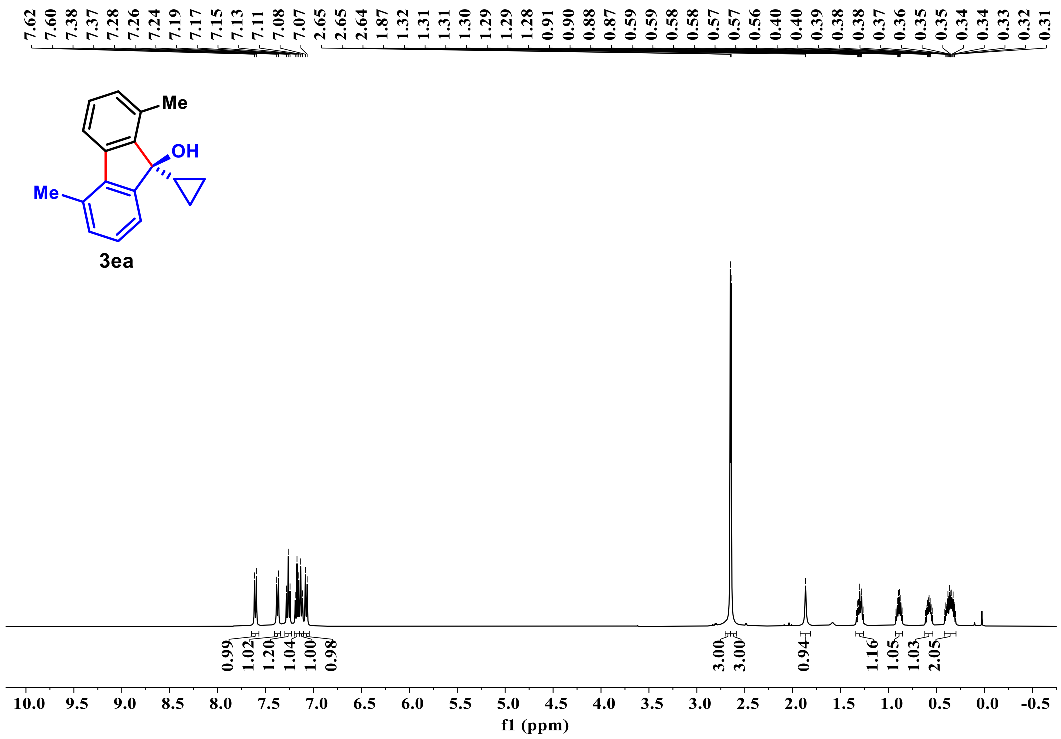

^{13}C NMR (100 MHz, $CDCl_3$)

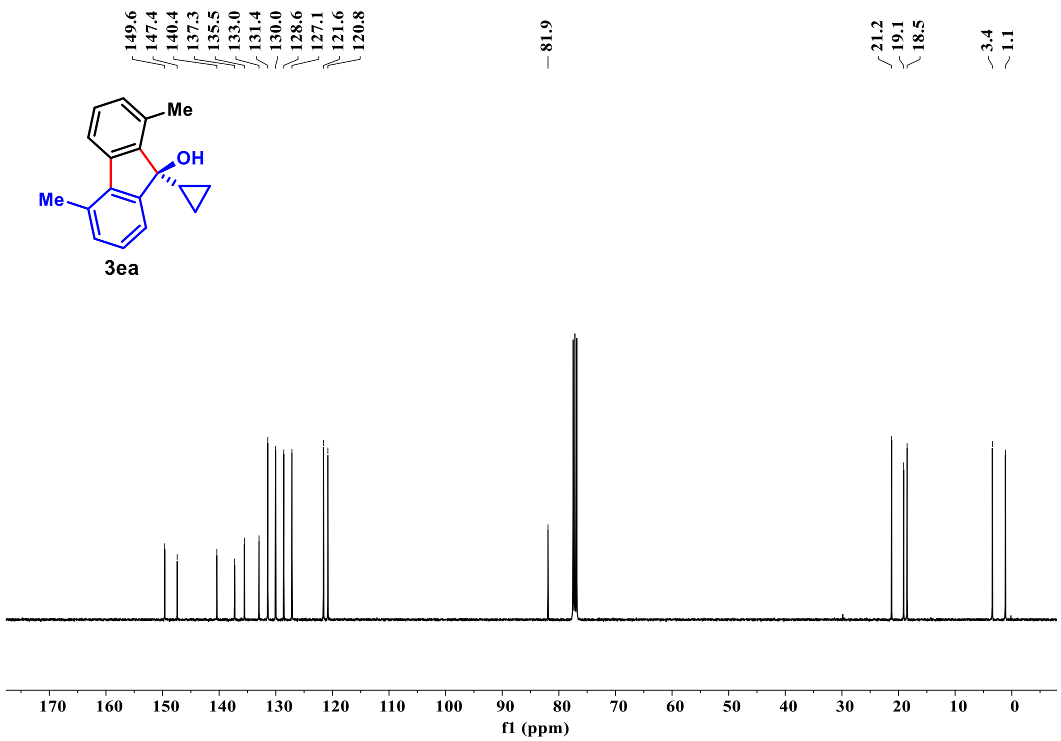

^{1}H NMR (400 MHz, $CDCl_3$)

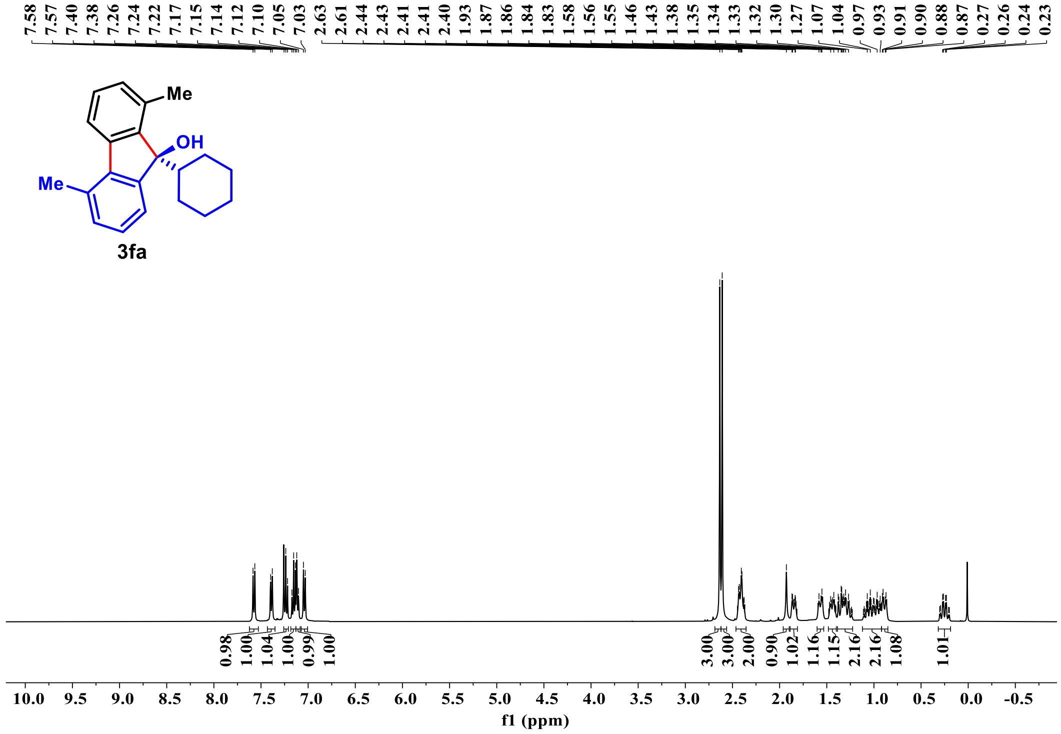

^{13}C NMR (100 MHz, $CDCl_3$)

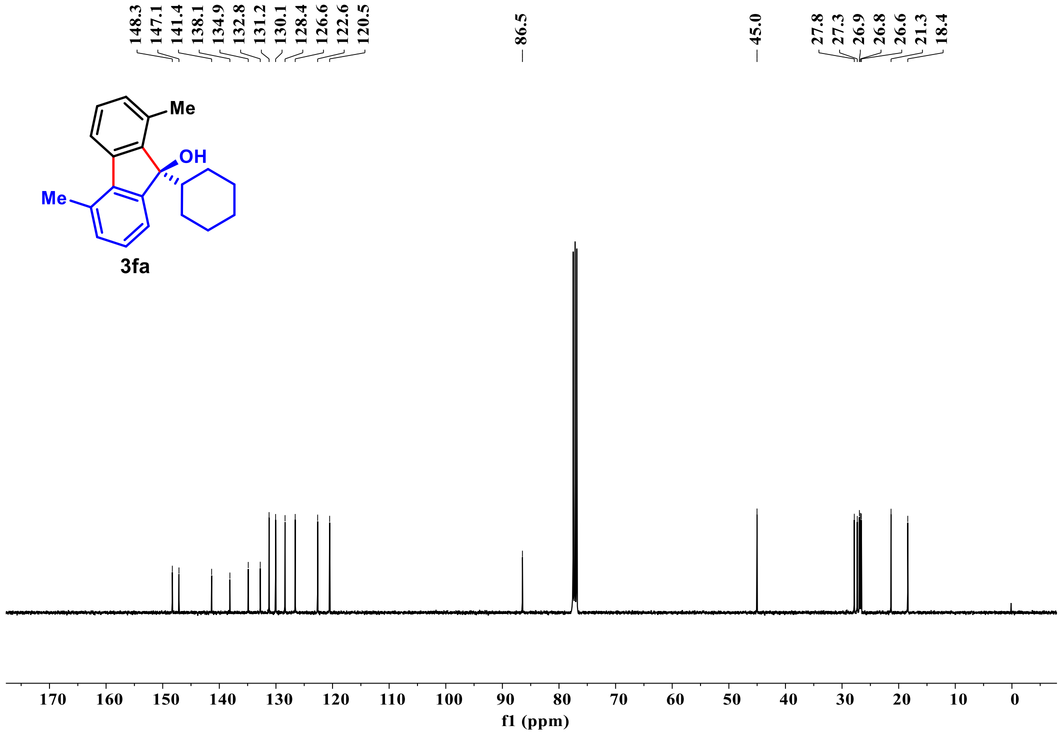

^{1}H NMR (400 MHz, $CDCl_3$)

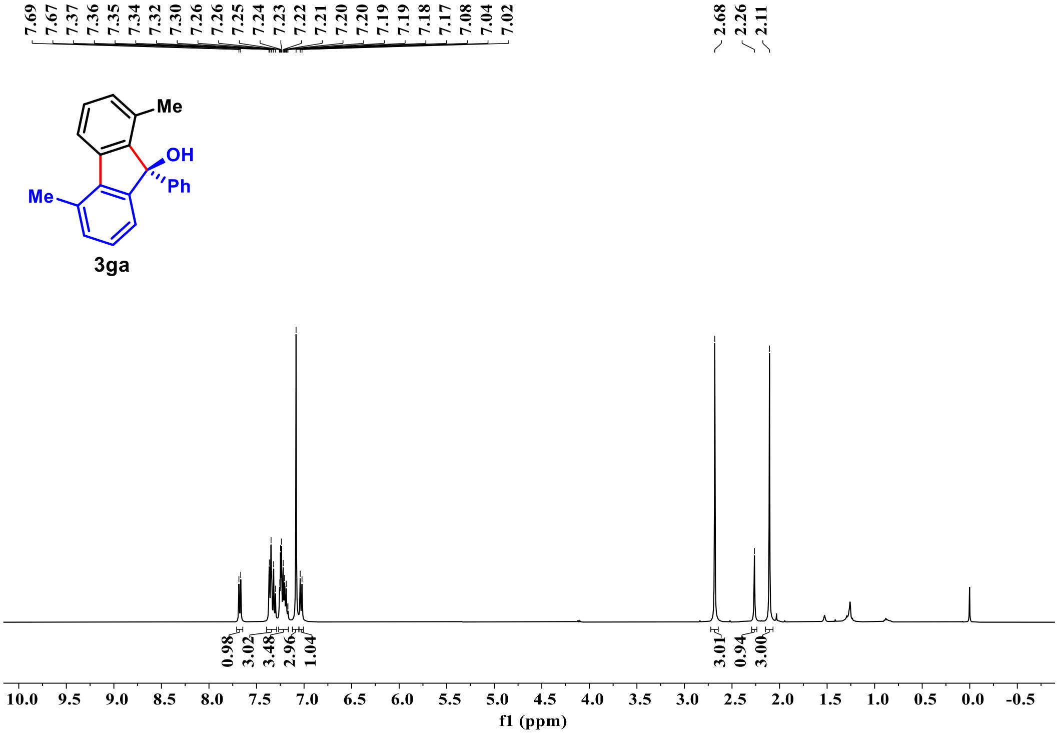

^{13}C NMR (100 MHz, $CDCl_3$)

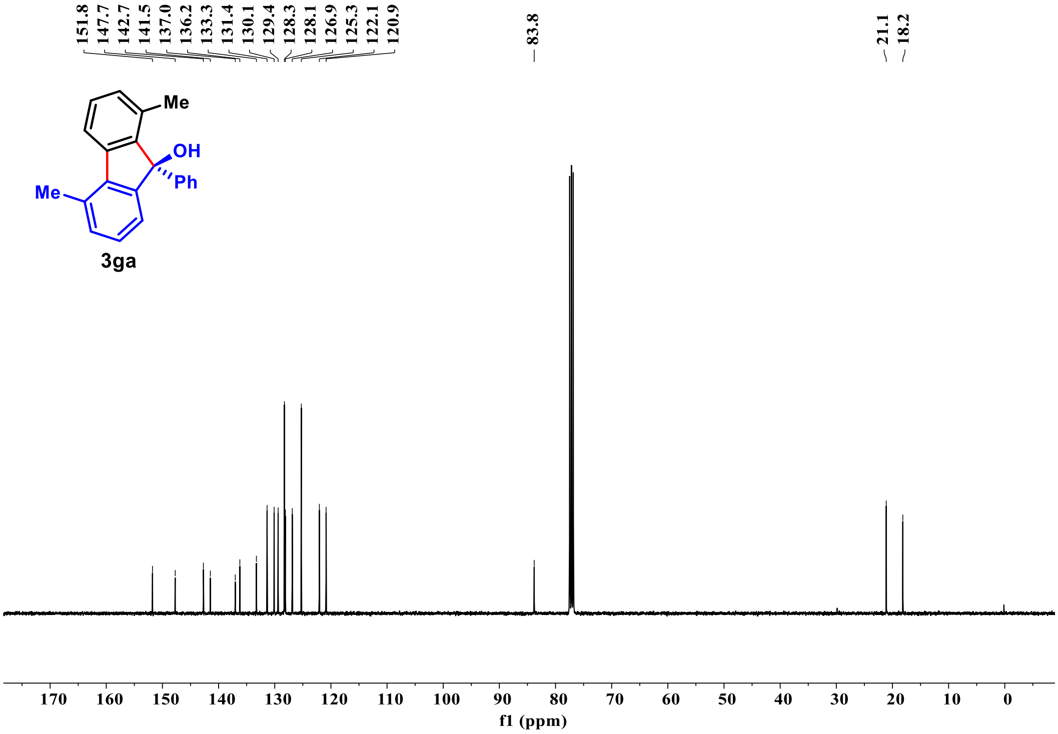

^{1}H NMR (400 MHz, $CDCl_3$)

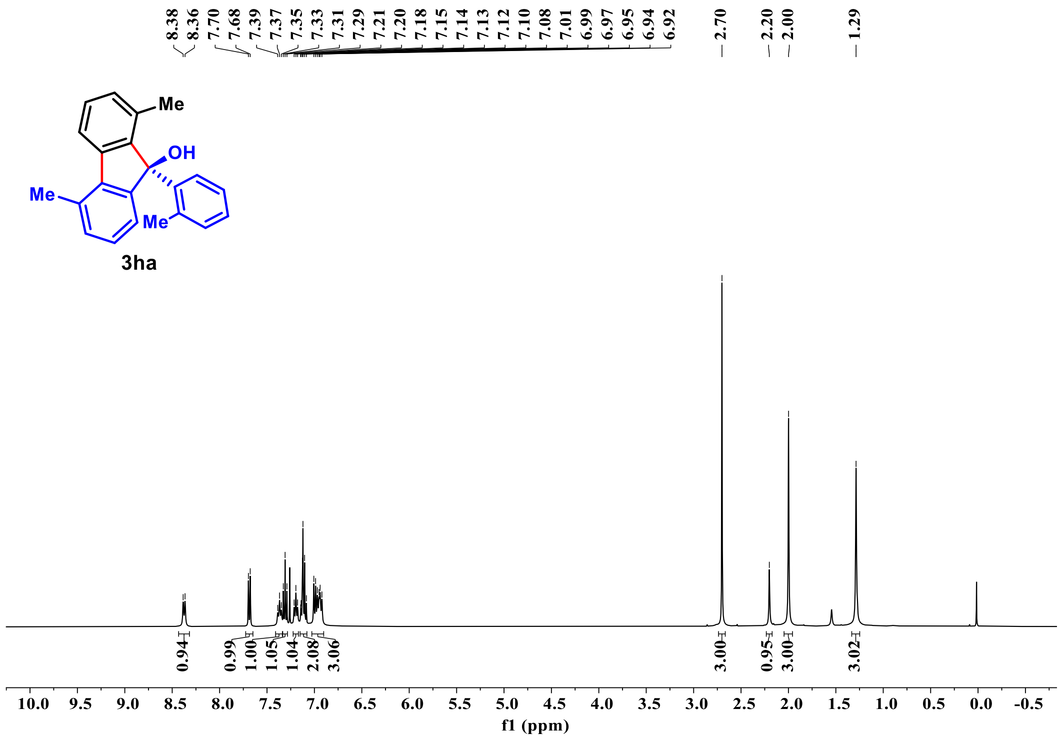

^{13}C NMR (100 MHz, $CDCl_3$)

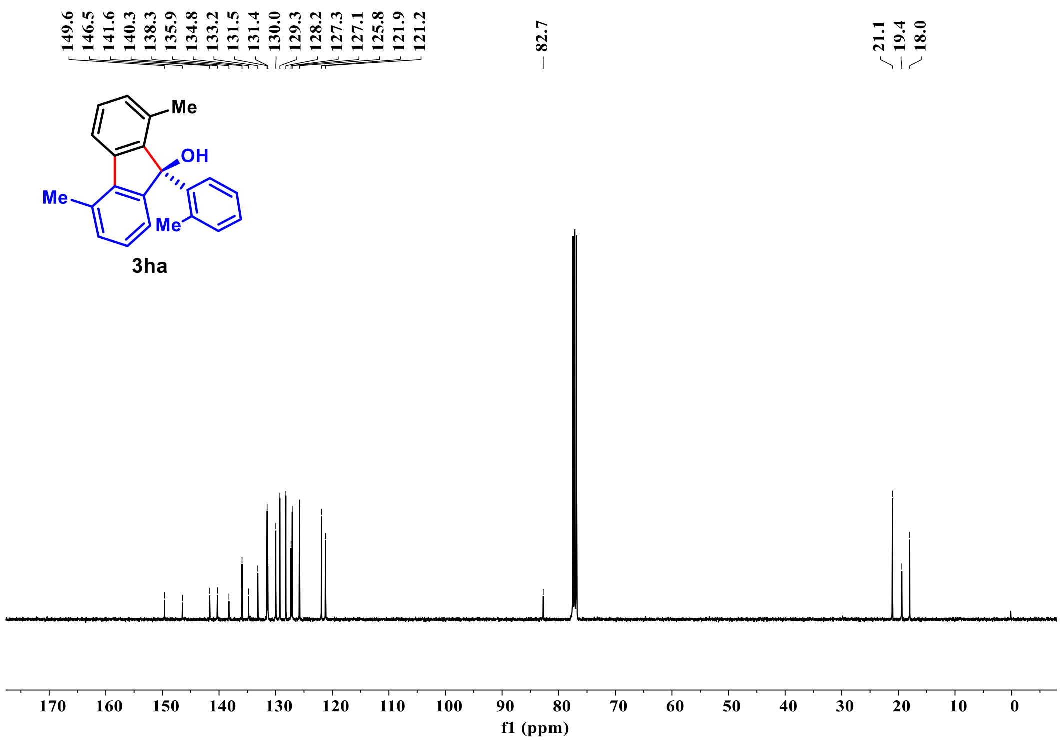

^{1}H NMR (400 MHz, $CDCl_3$)

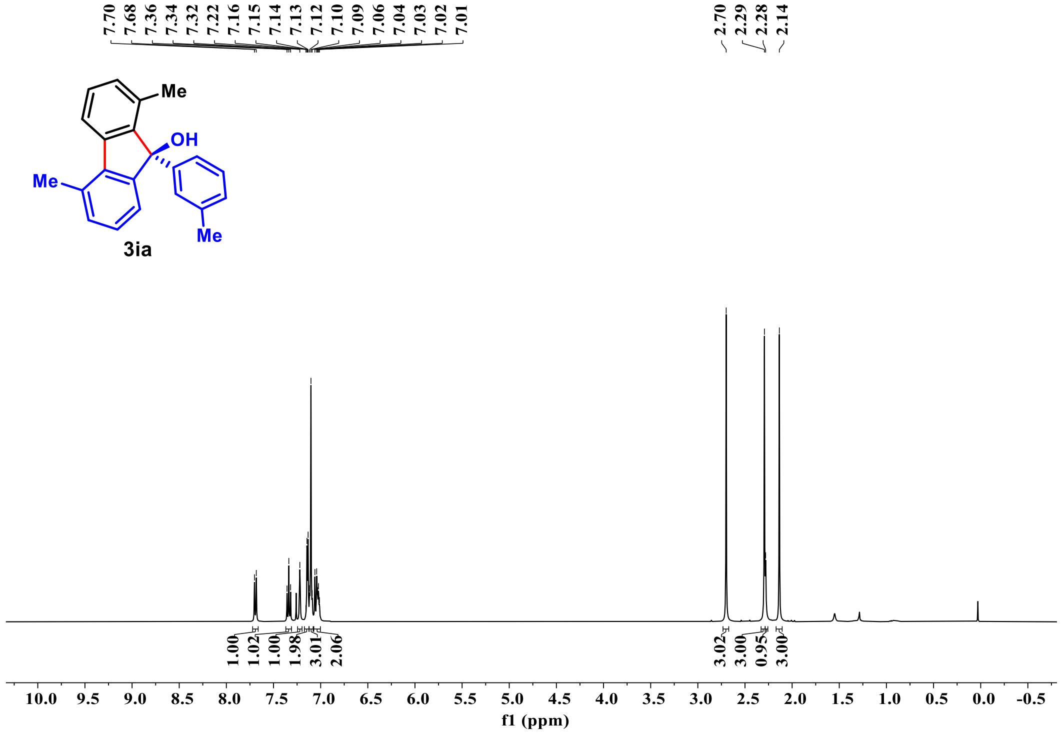

^{13}C NMR (100 MHz, $CDCl_3$)

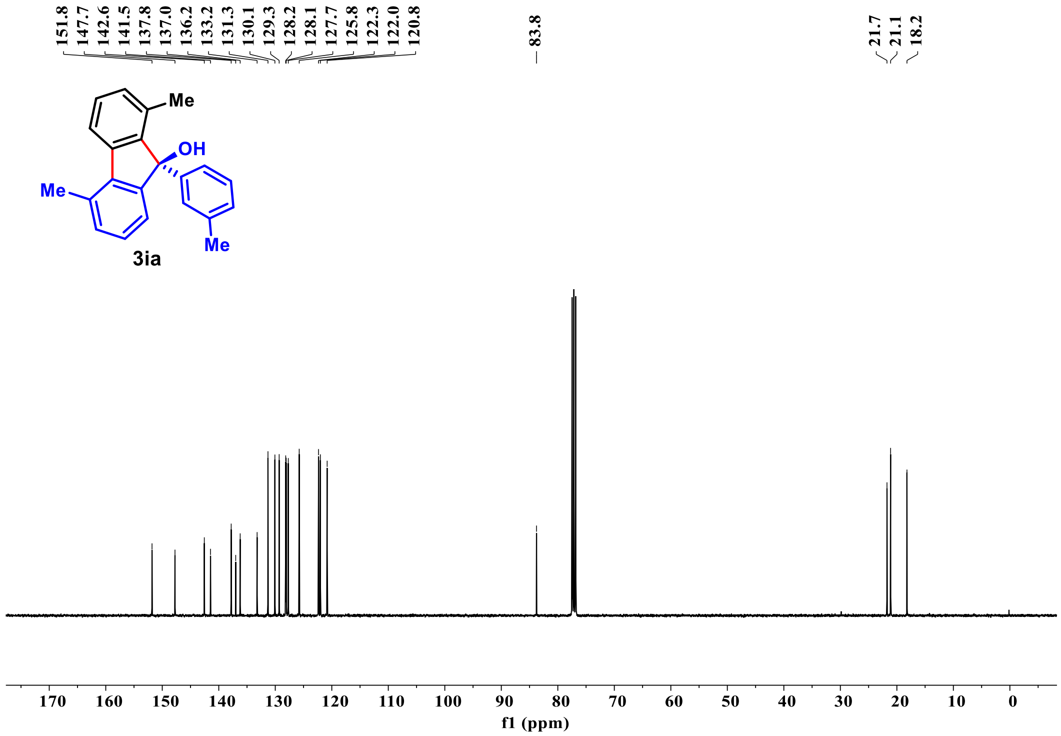

^{1}H NMR (400 MHz, $CDCl_3$)

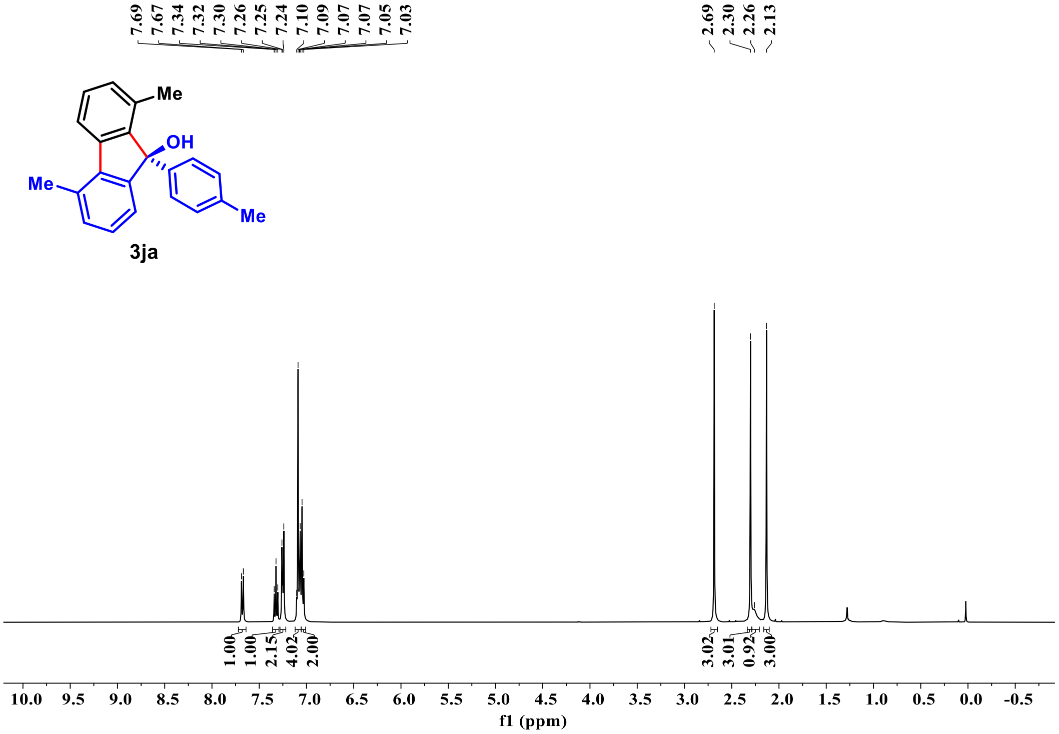

^{13}C NMR (100 MHz, $CDCl_3$)

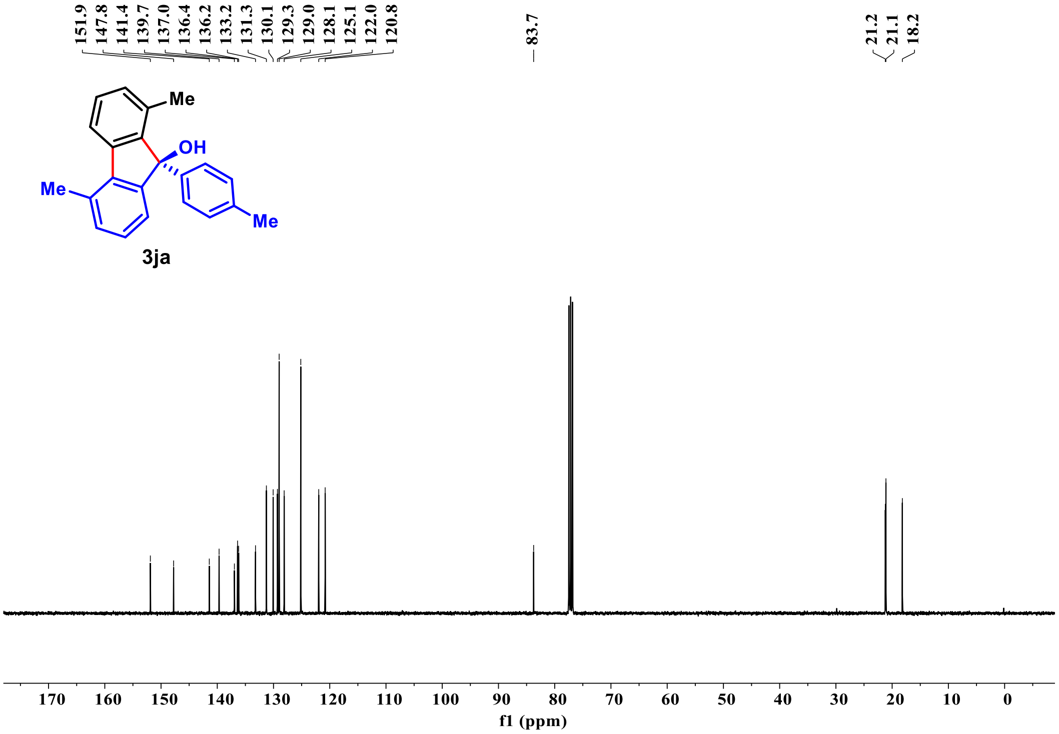

^{1}H NMR (400 MHz, $CDCl_3$)

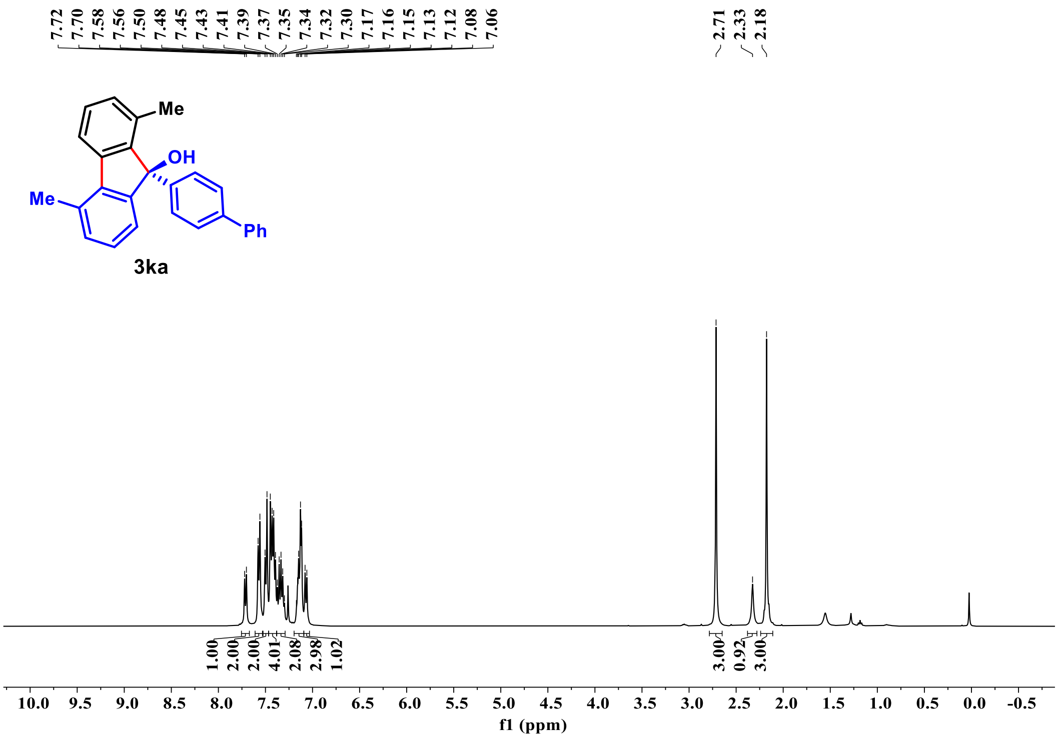

^{13}C NMR (100 MHz, $CDCl_3$)

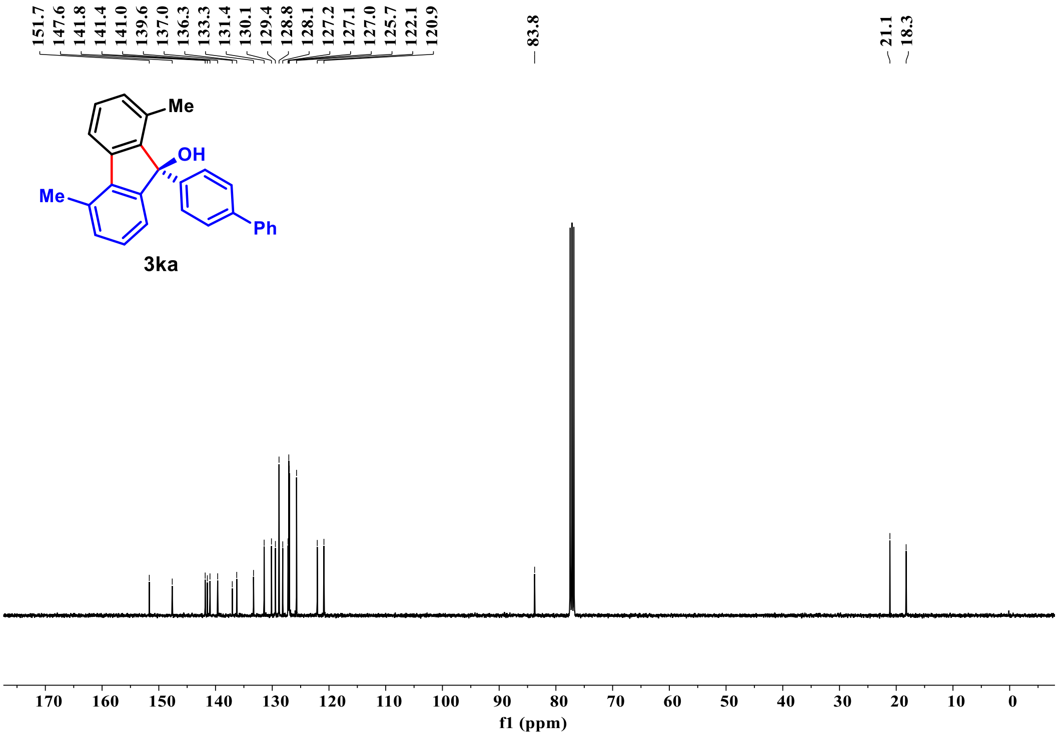

^{1}H NMR (400 MHz, $CDCl_3$)

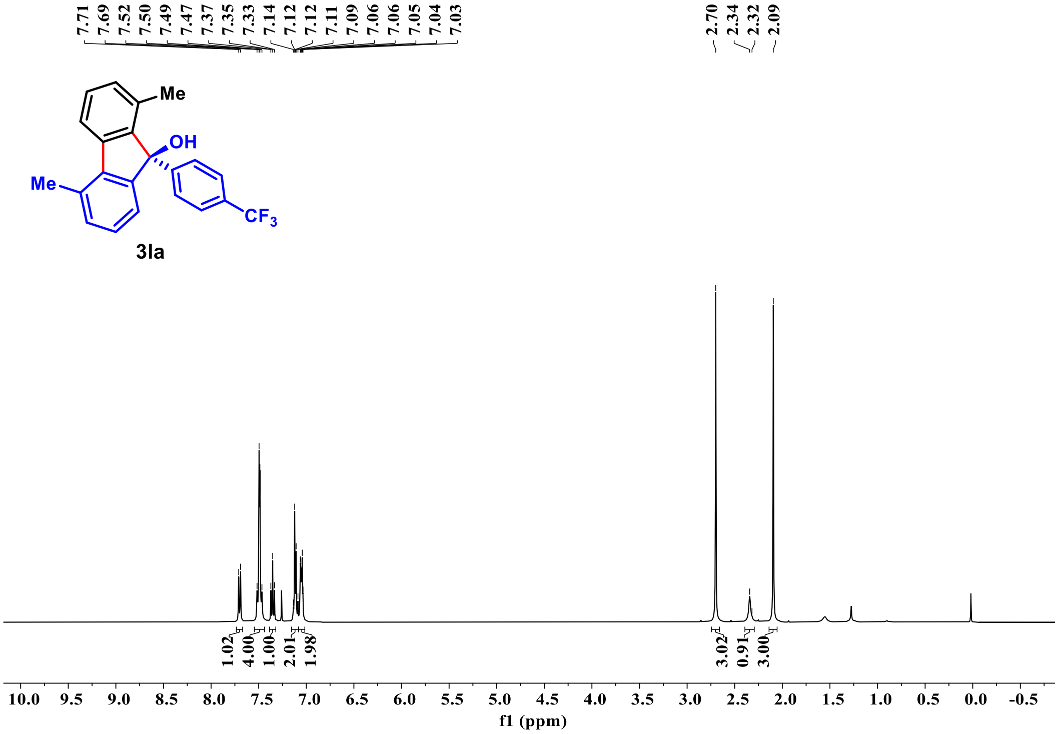

^{13}C NMR (100 MHz, $CDCl_3$)

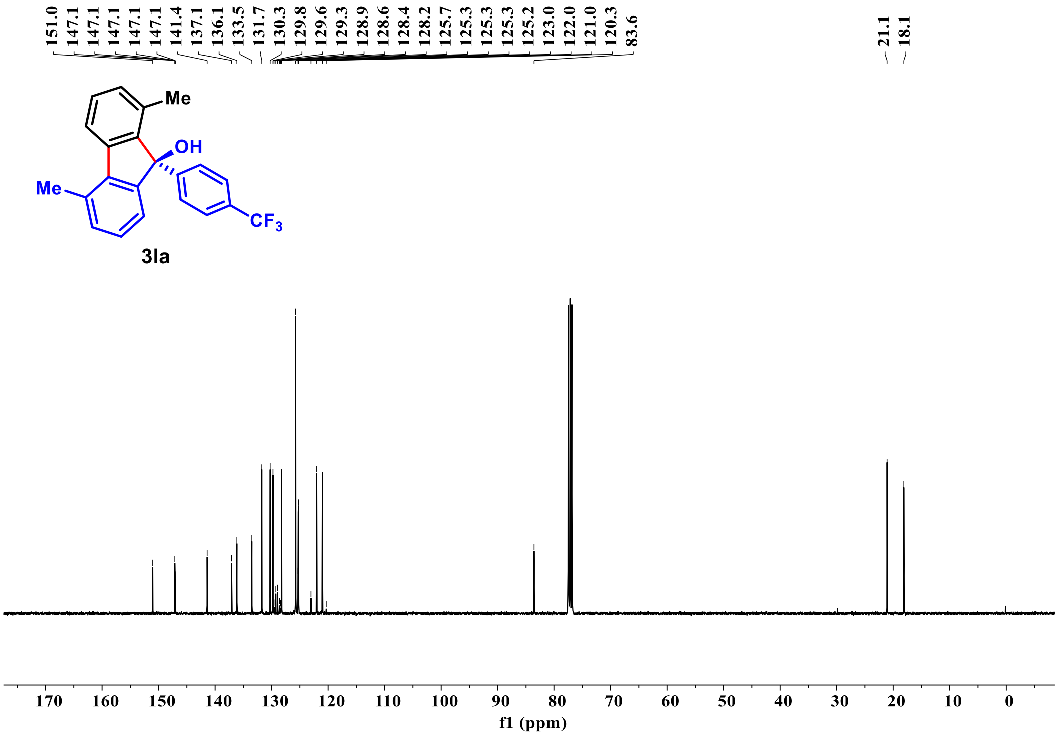

^{19}F NMR (376 MHz, $CDCl_3$)

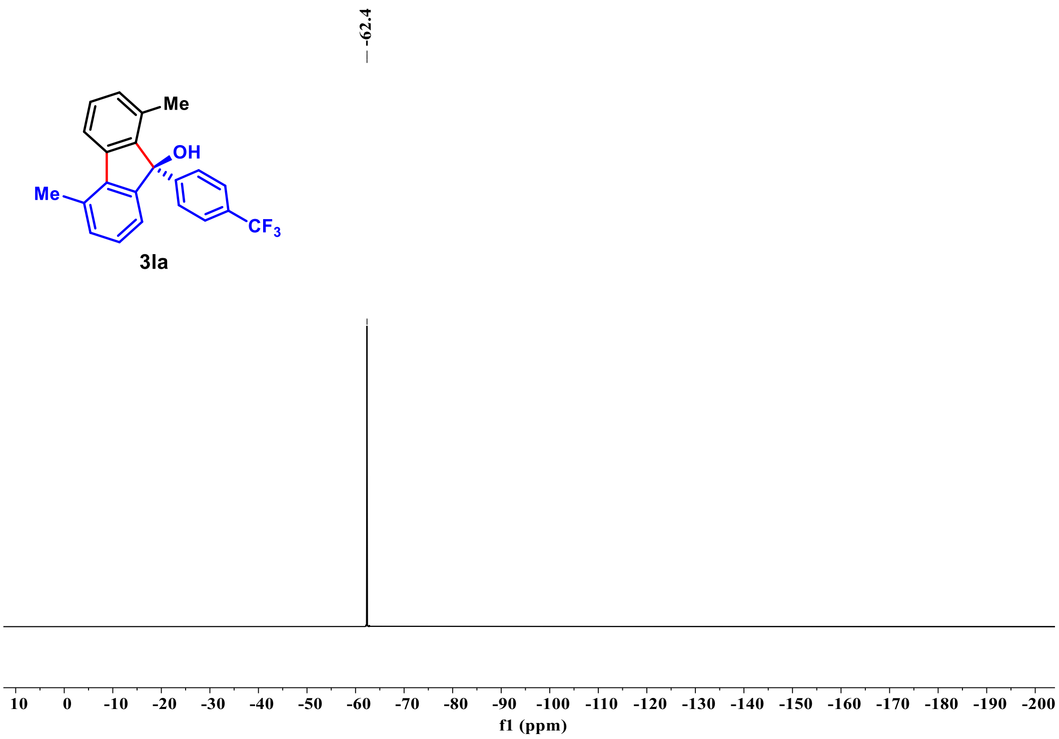

^{1}H NMR (400 MHz, $CDCl_3$)

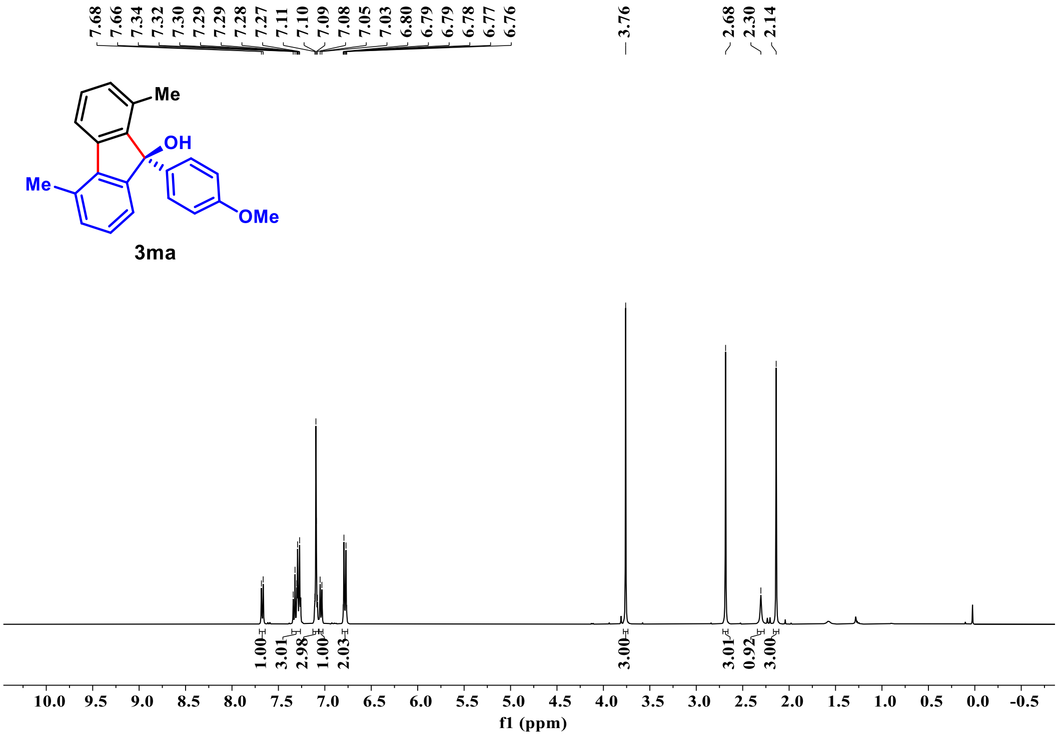

^{13}C NMR (100 MHz, $CDCl_3$)

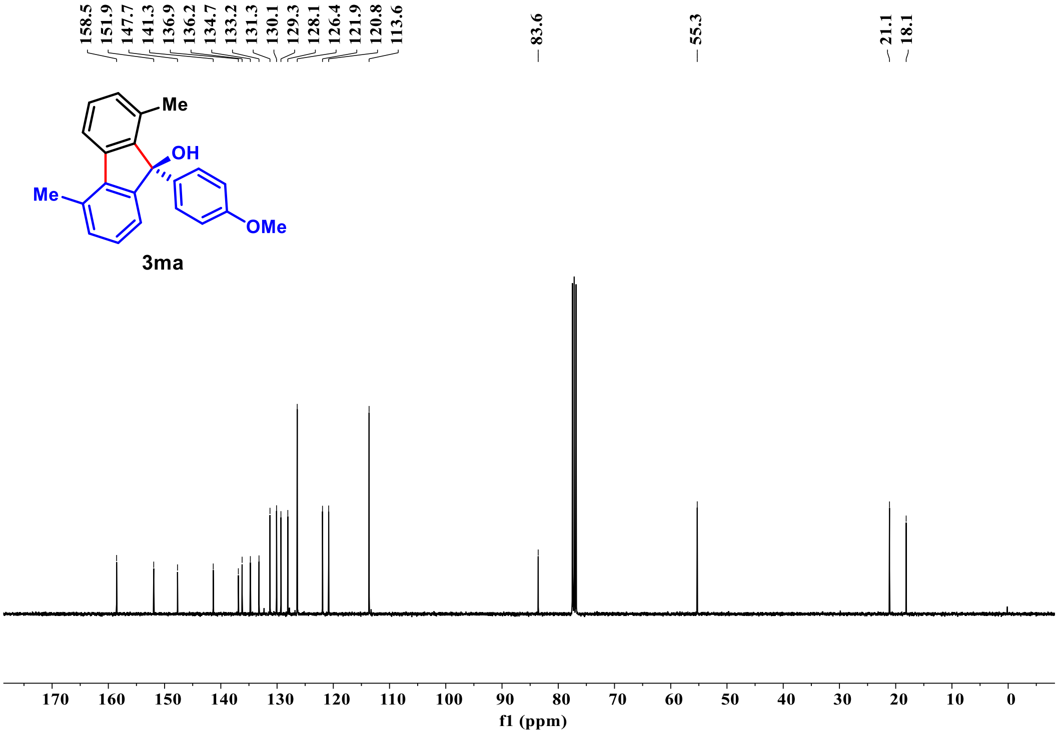

^{1}H NMR (400 MHz, $CDCl_3$)

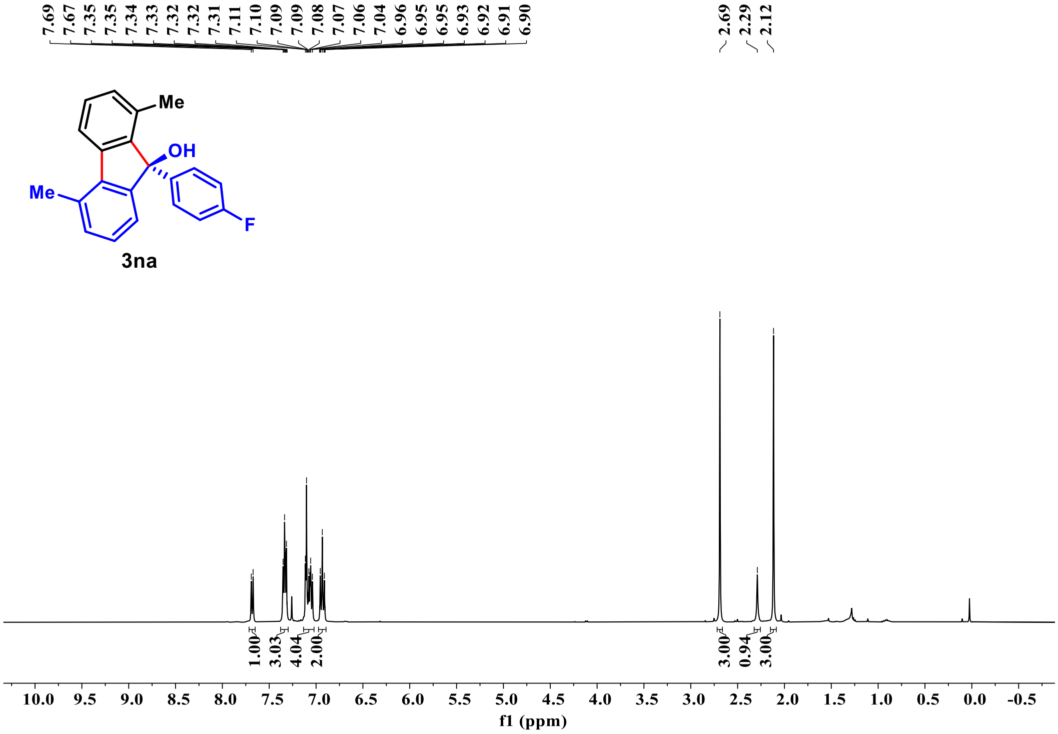

^{13}C NMR (100 MHz, $CDCl_3$)

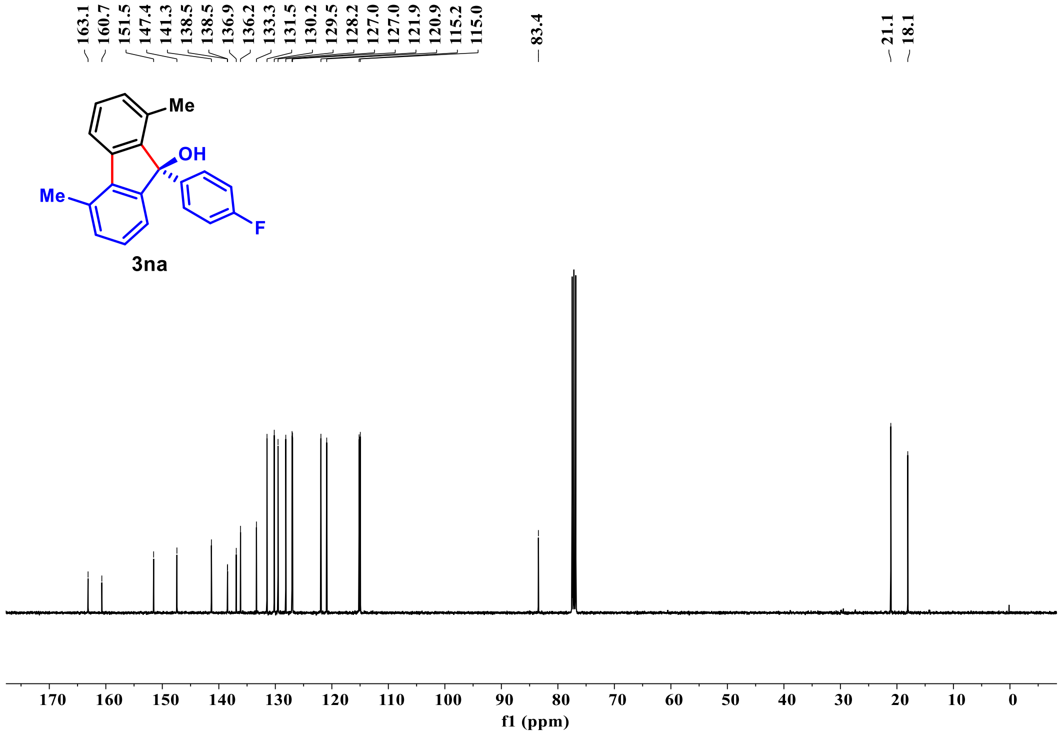

^{19}F NMR (376 MHz, $CDCl_3$)

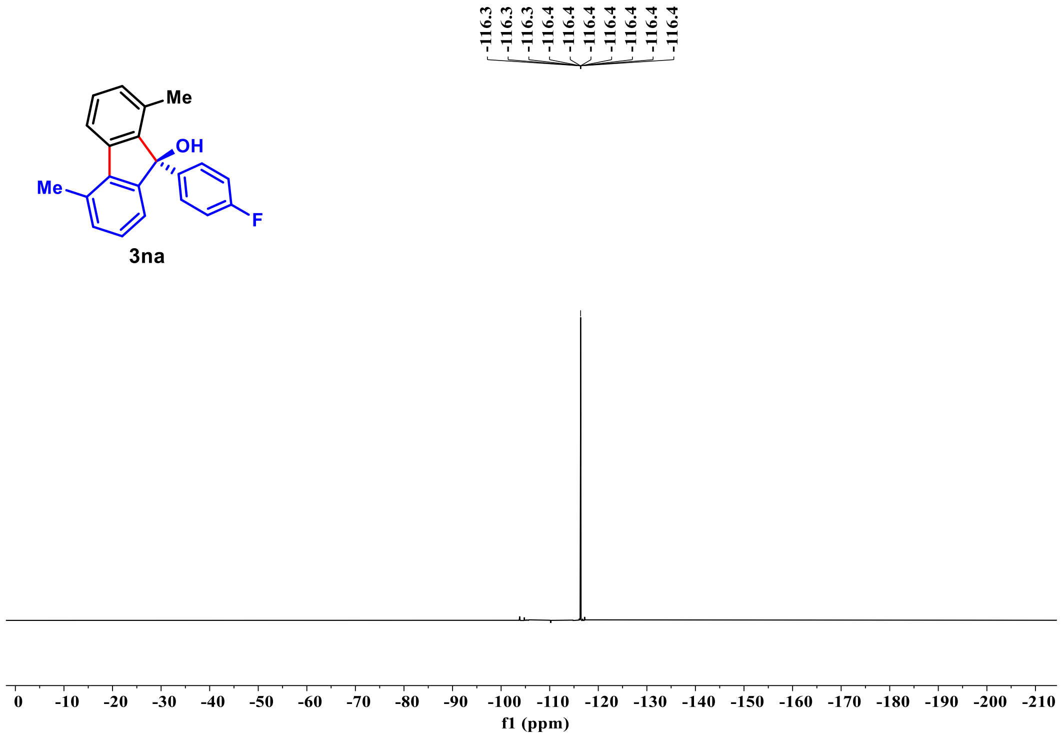

^{1}H NMR (400 MHz, $CDCl_3$)

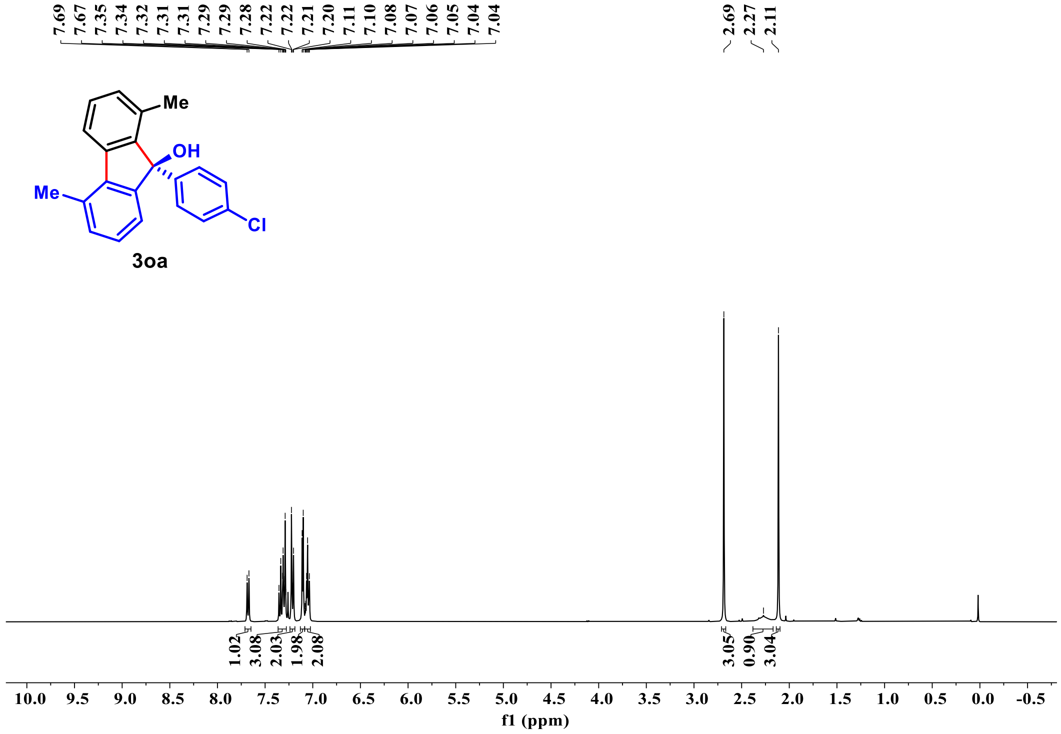

^{13}C NMR (100 MHz, $CDCl_3$)

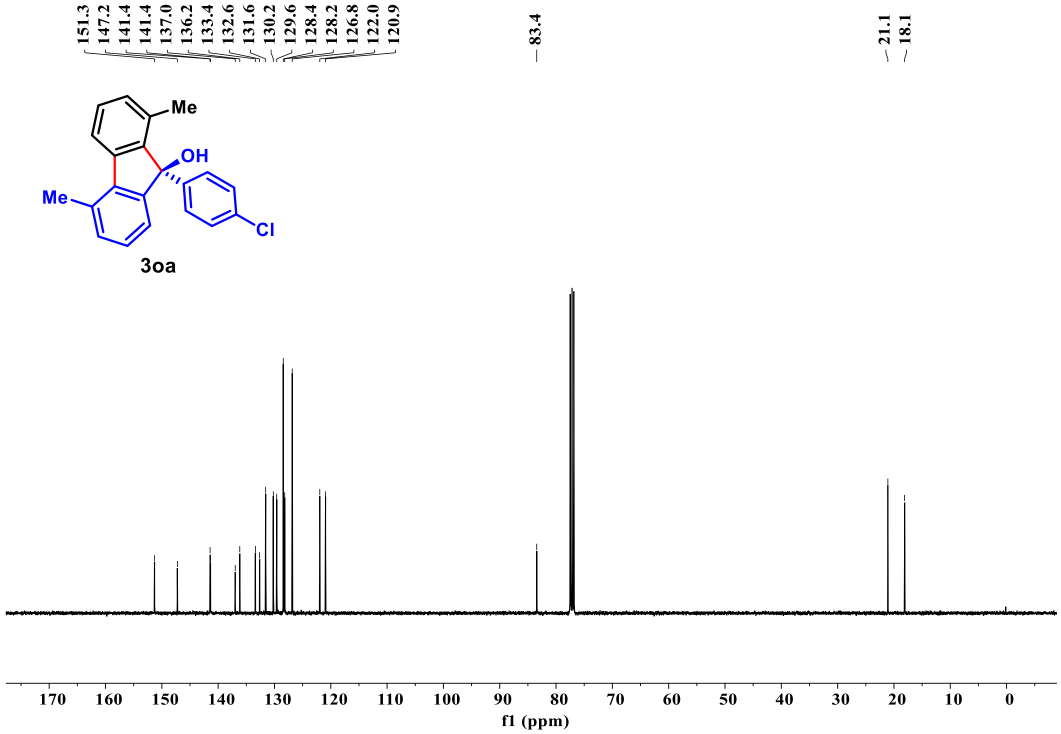

^{1}H NMR (400 MHz, $CDCl_3$)

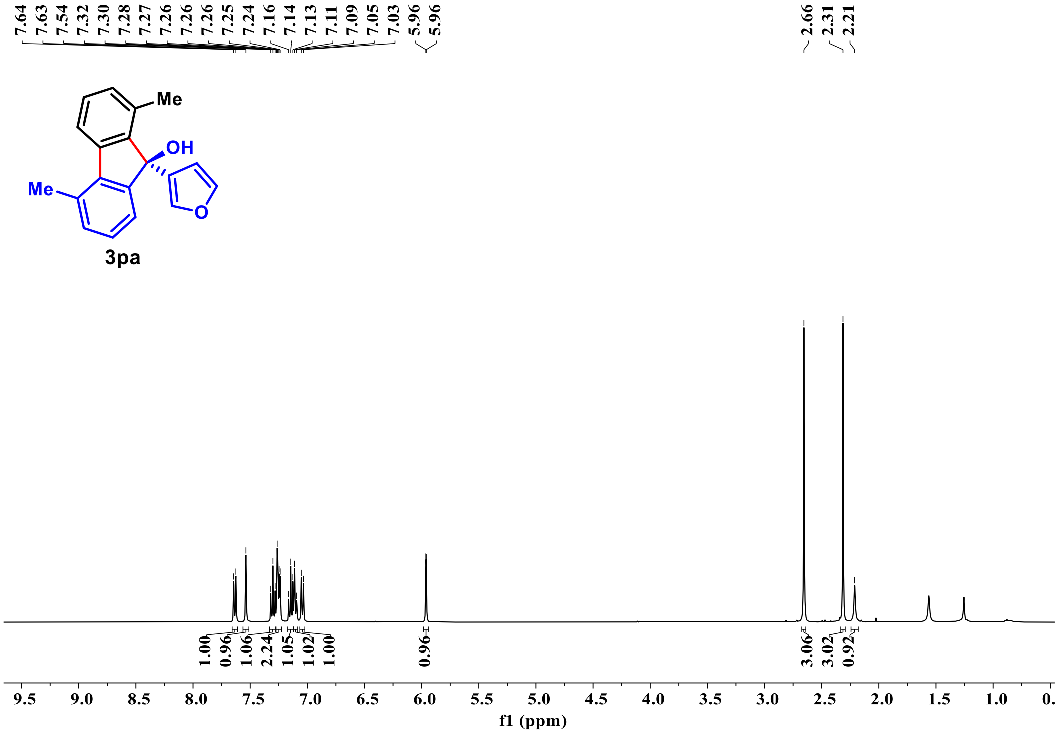

^{13}C NMR (100 MHz, $CDCl_3$)

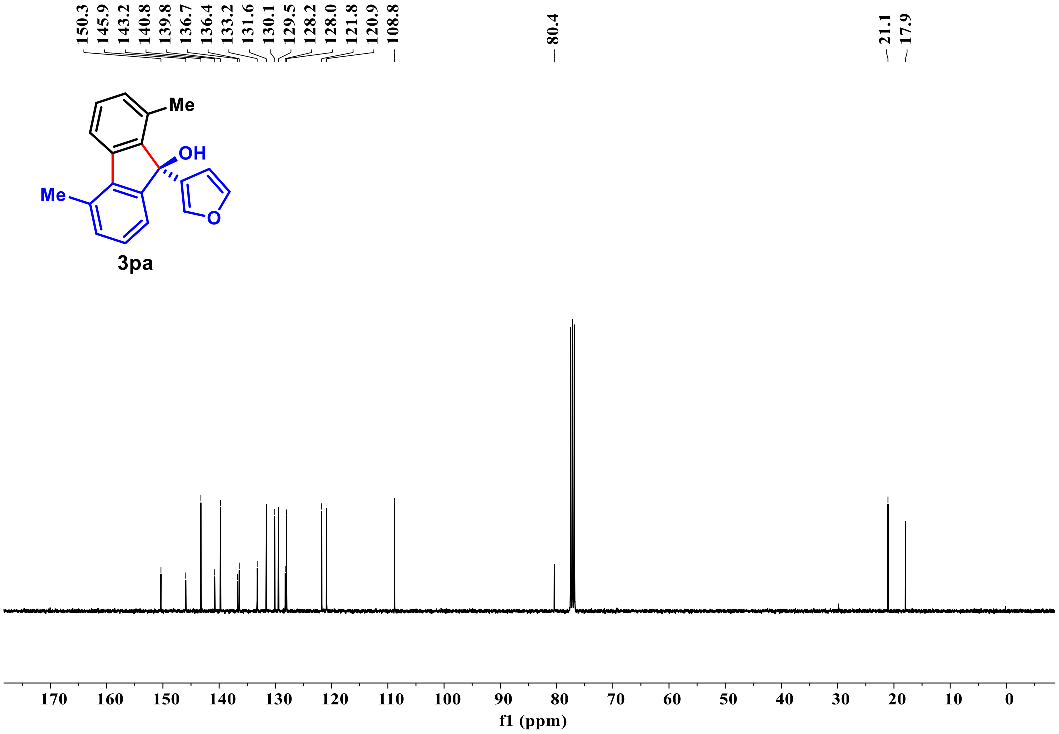

^{1}H NMR (400 MHz, $CDCl_3$)

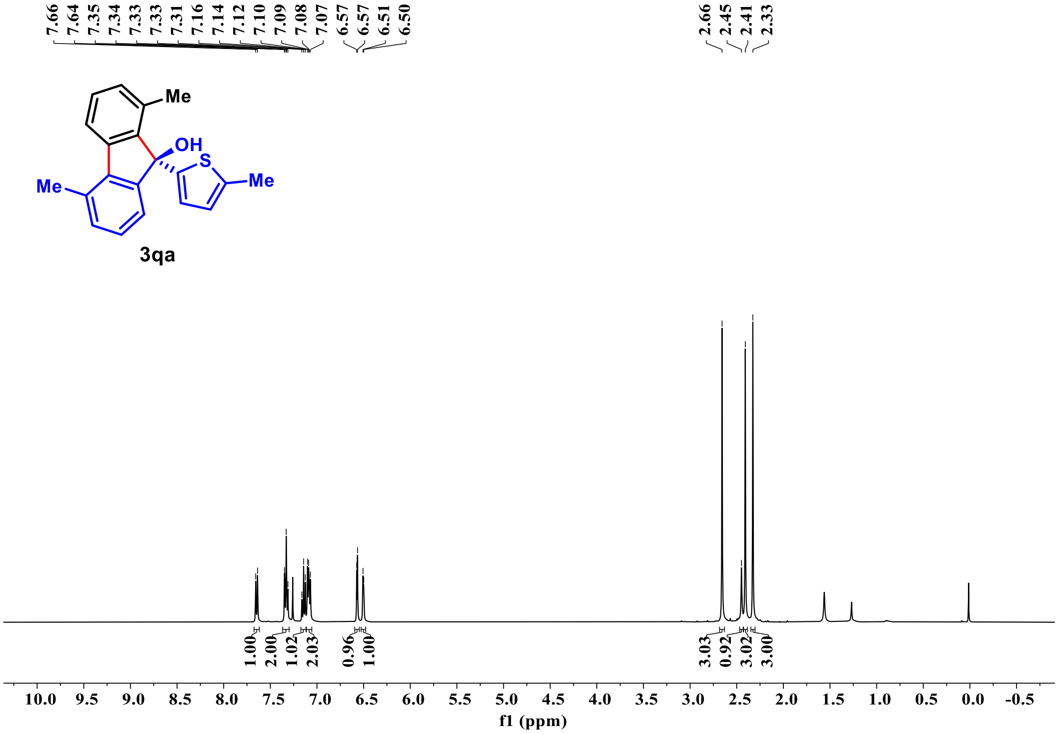

^{13}C NMR (100 MHz, $CDCl_3$)

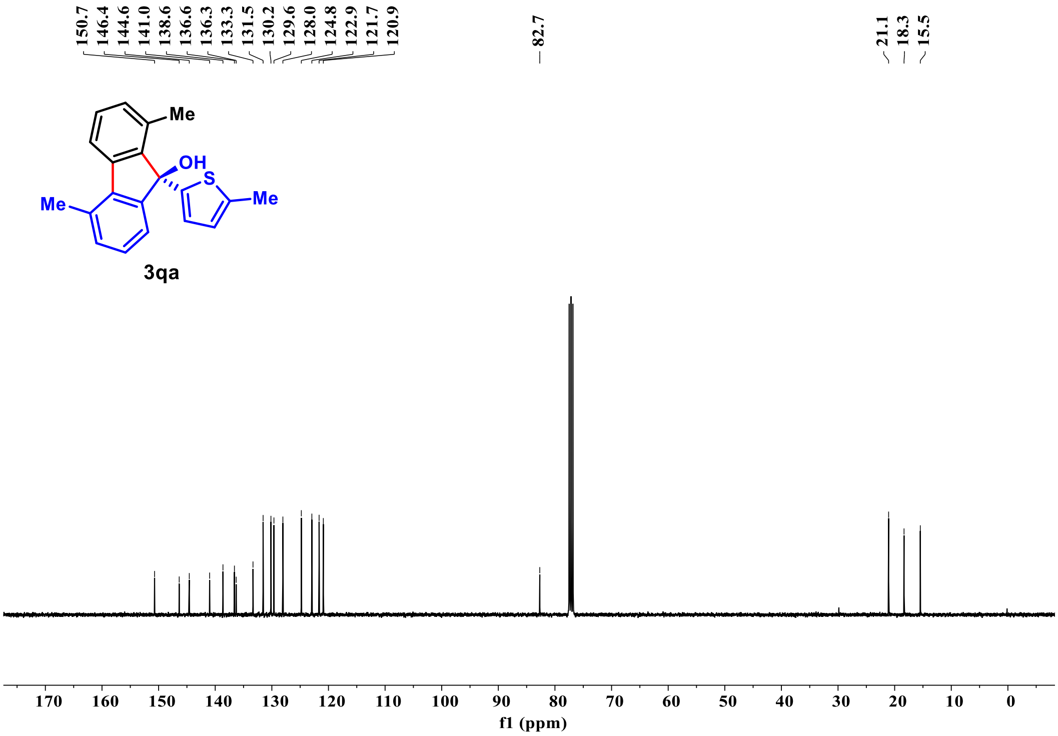

^{1}H NMR (400 MHz, $CDCl_3$)

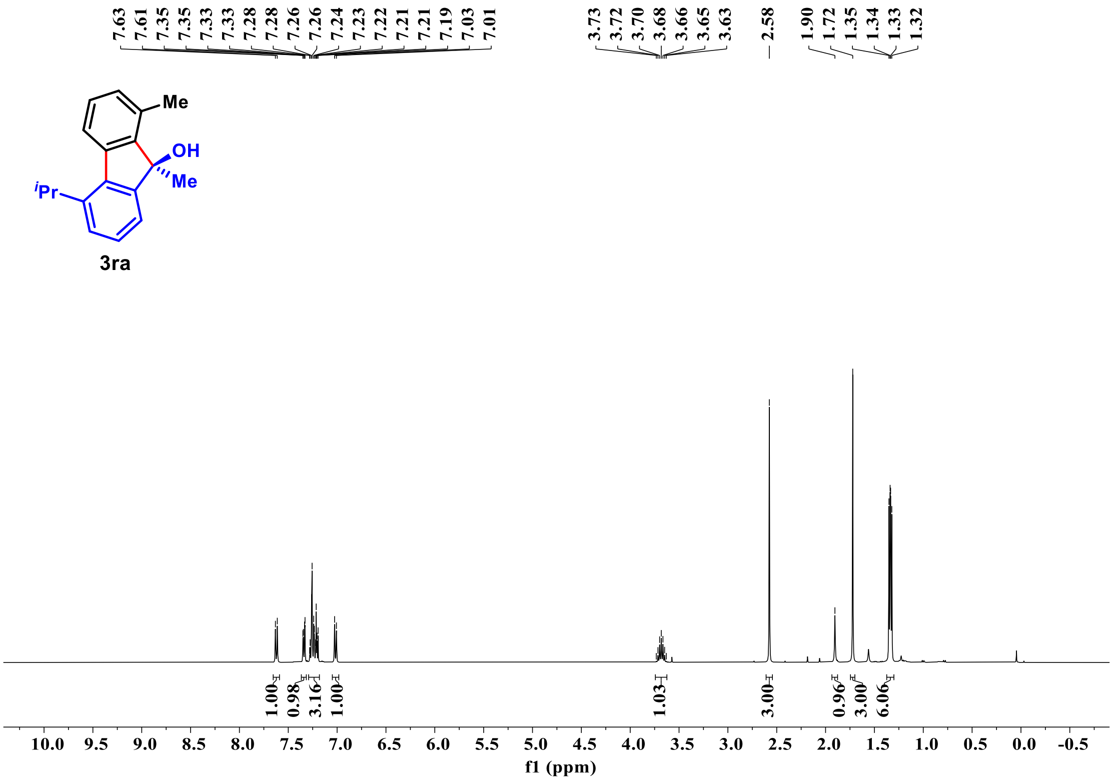

^{13}C NMR (100 MHz, $CDCl_3$)

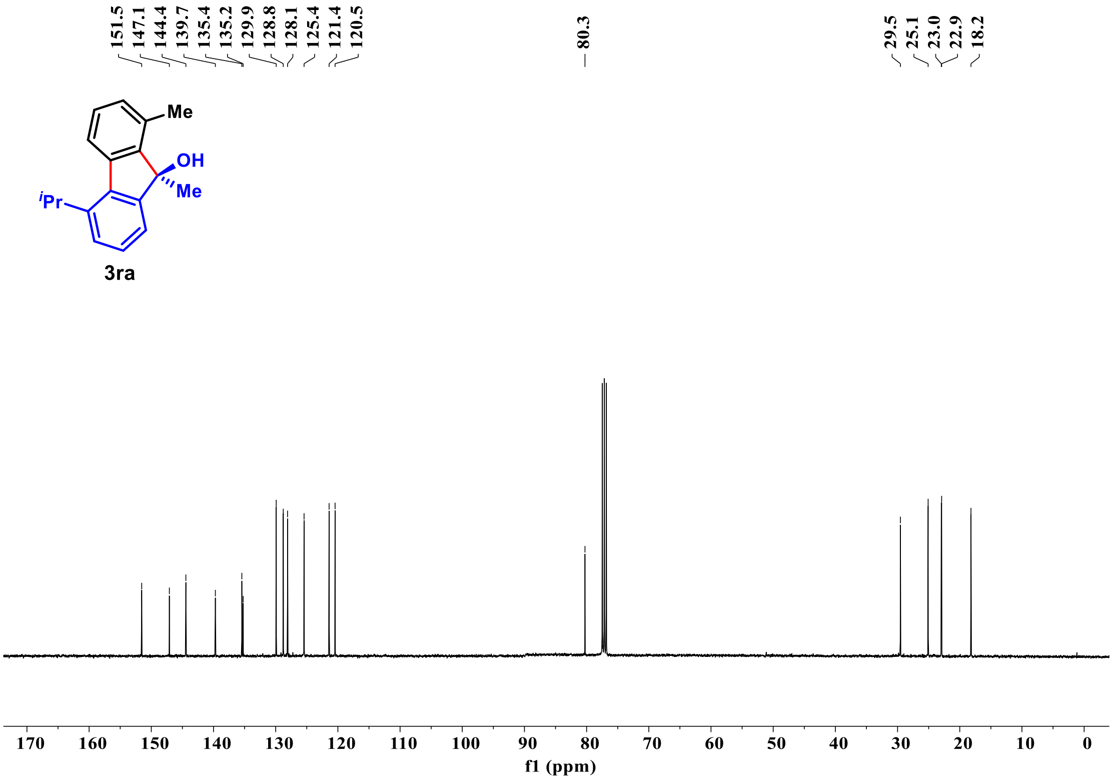

^{1}H NMR (400 MHz, $CDCl_3$)

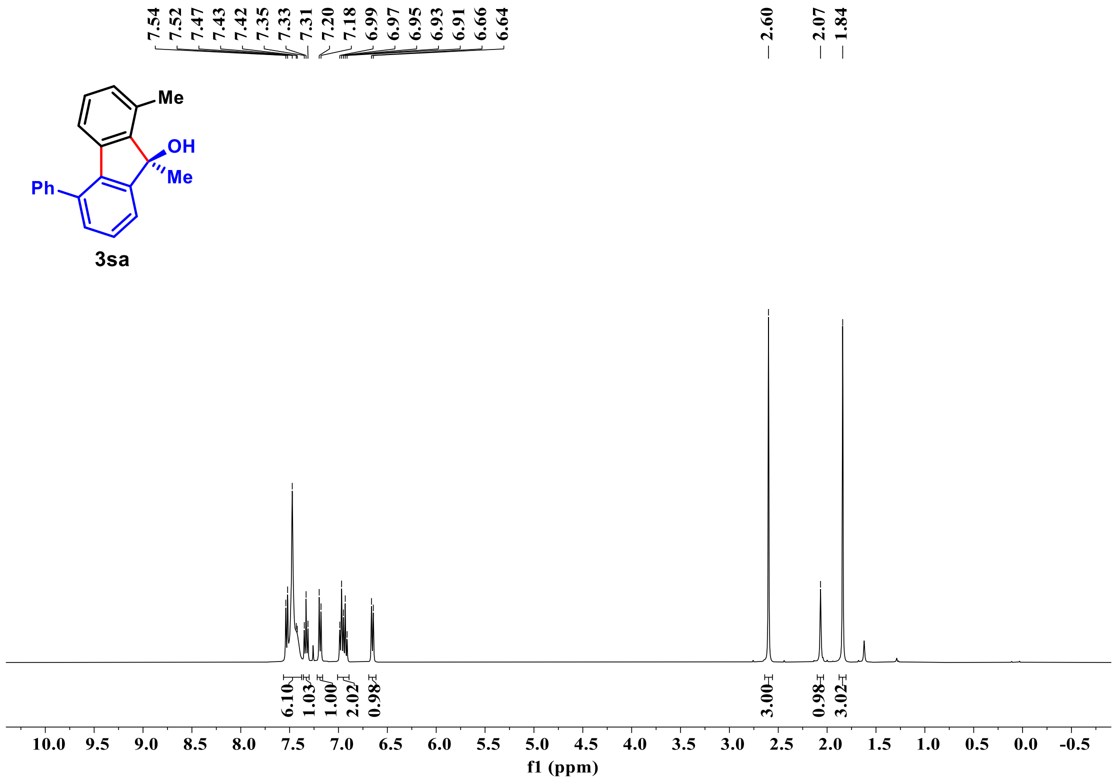

^{13}C NMR (100 MHz, $CDCl_3$)

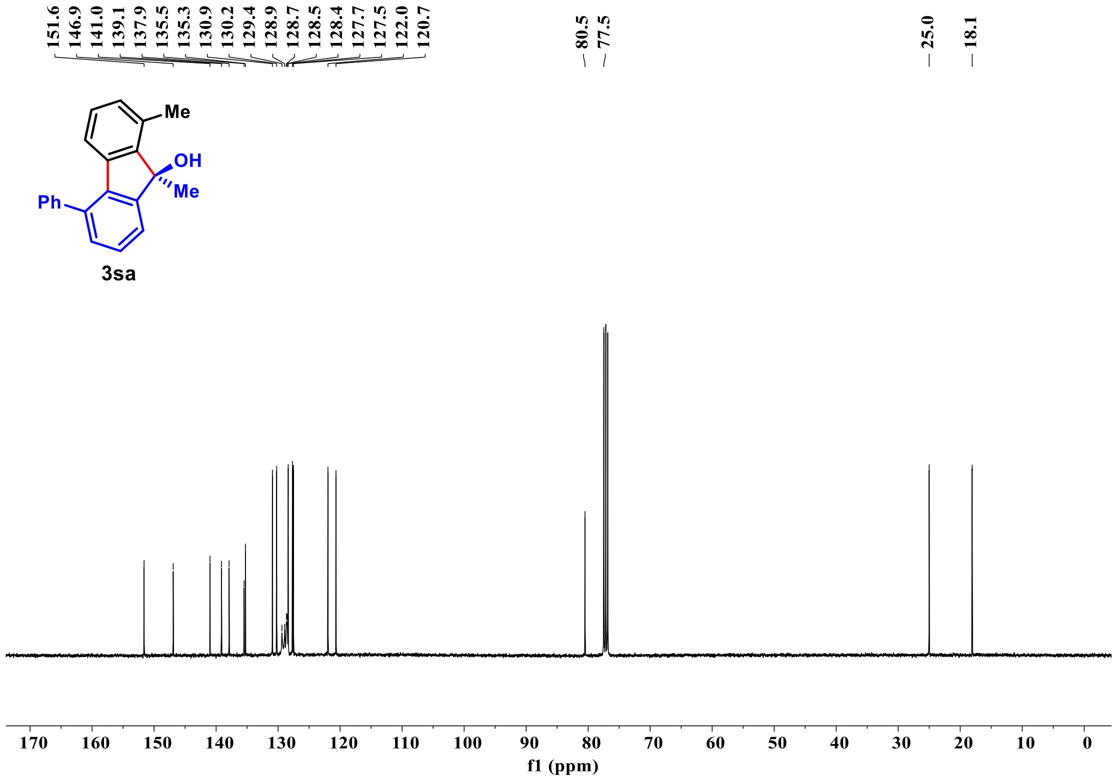

^{1}H NMR (400 MHz, $CDCl_3$)

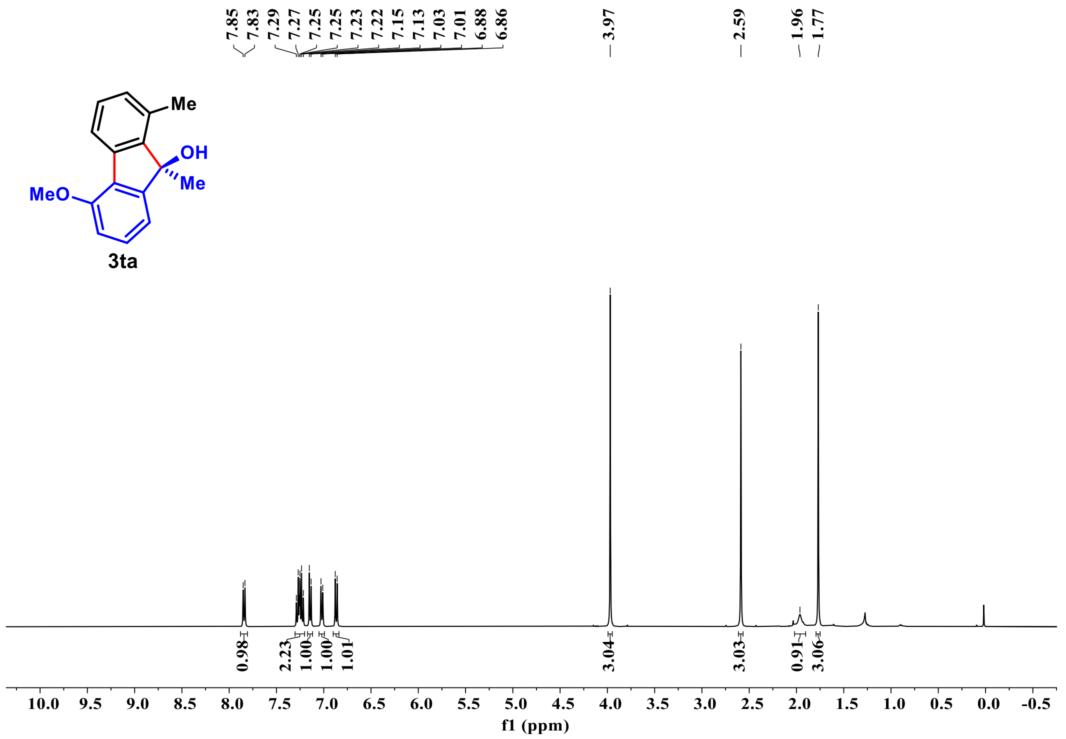

^{13}C NMR (100 MHz, $CDCl_3$)

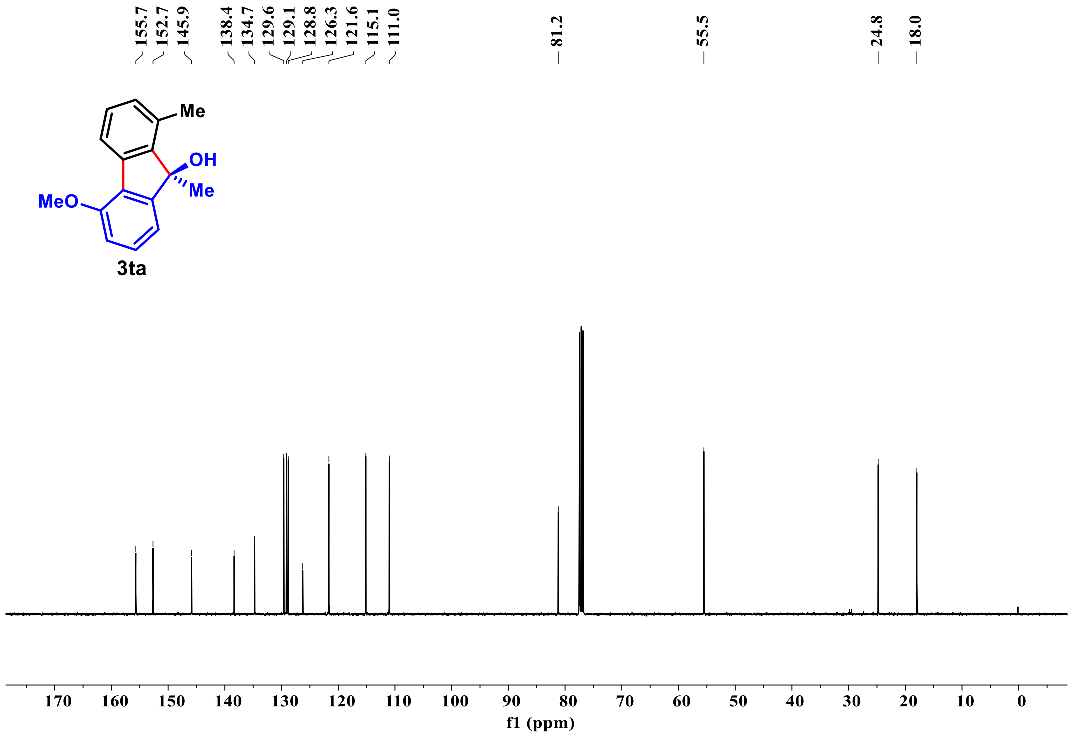

^{1}H NMR (400 MHz, $CDCl_3$)

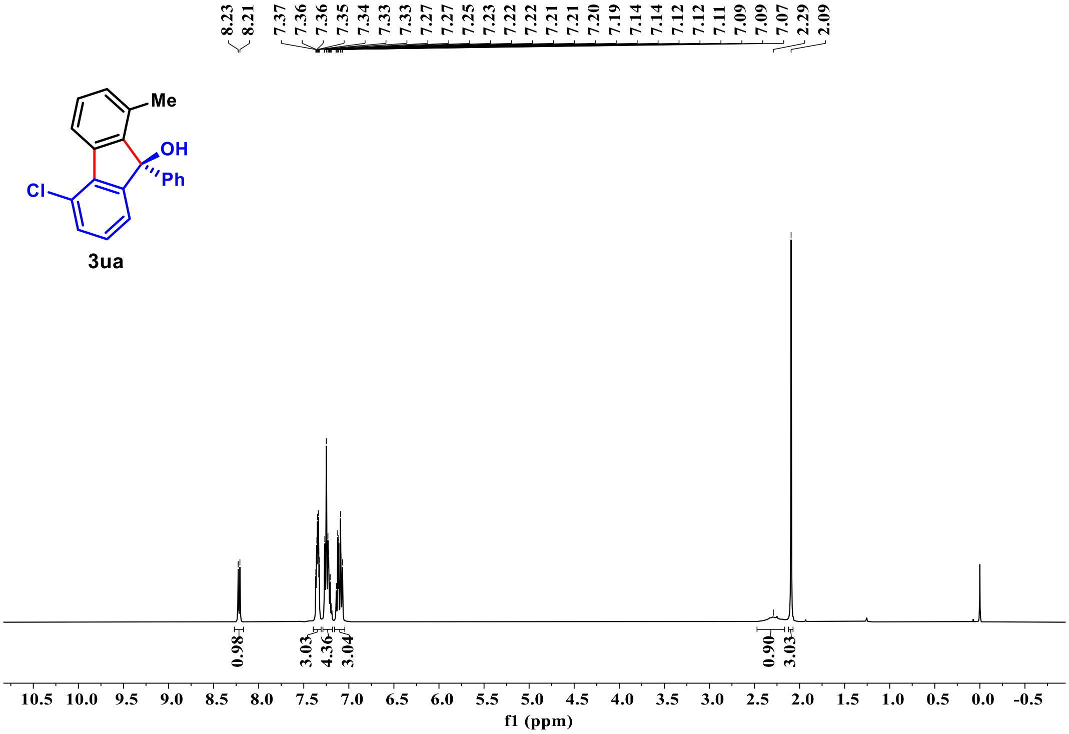

^{13}C NMR (100 MHz, $CDCl_3$)

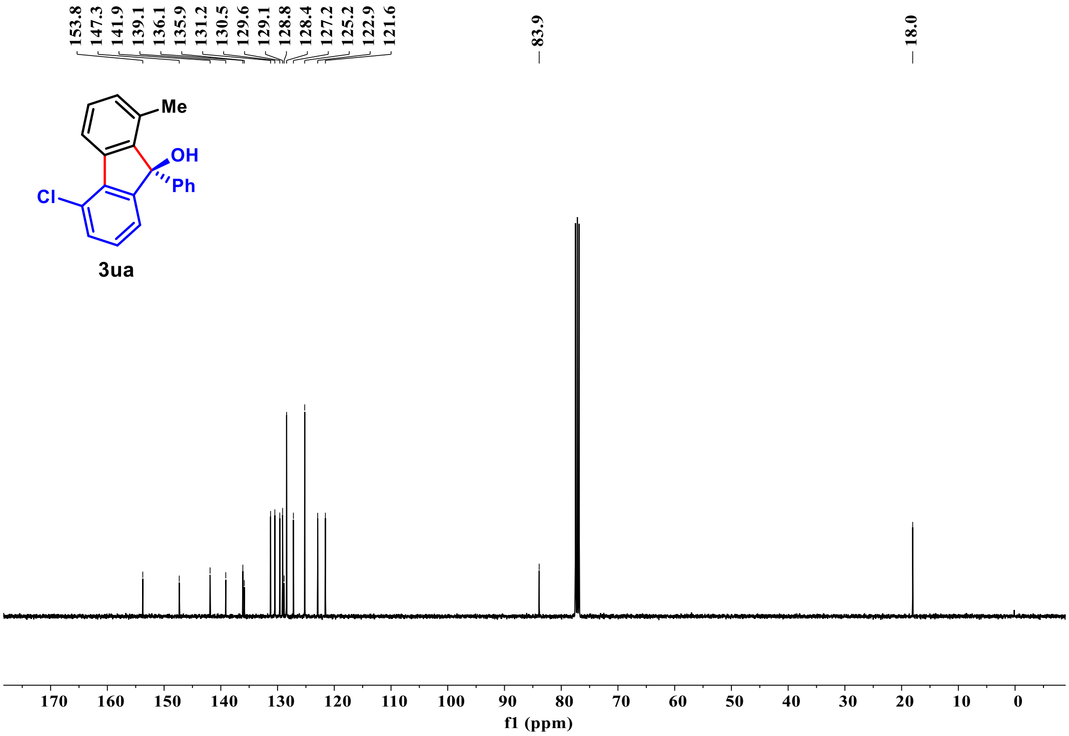

^{1}H NMR (400 MHz, $CDCl_3$)

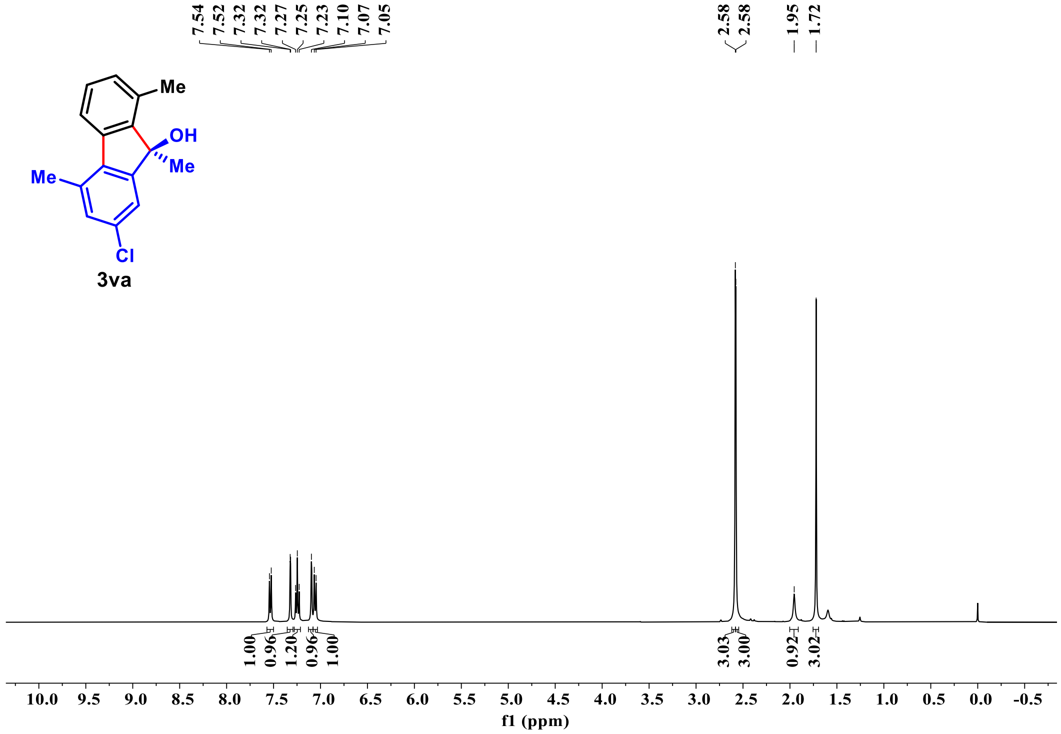

^{13}C NMR (100 MHz, $CDCl_3$)

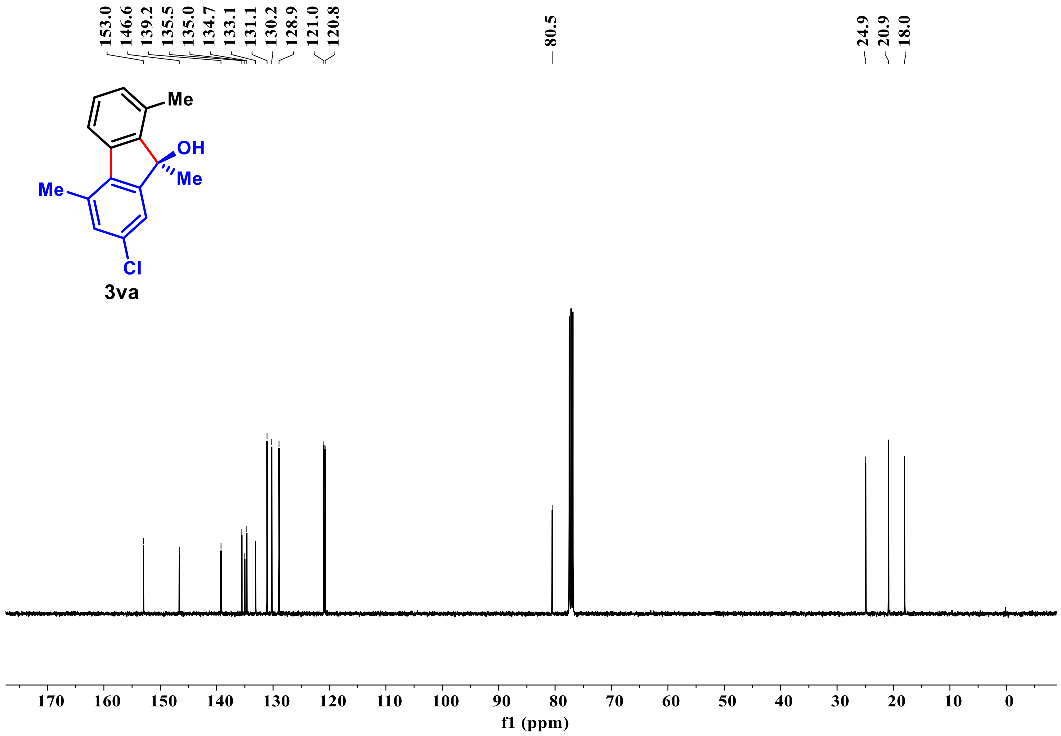

^{1}H NMR (400 MHz, $CDCl_3$)

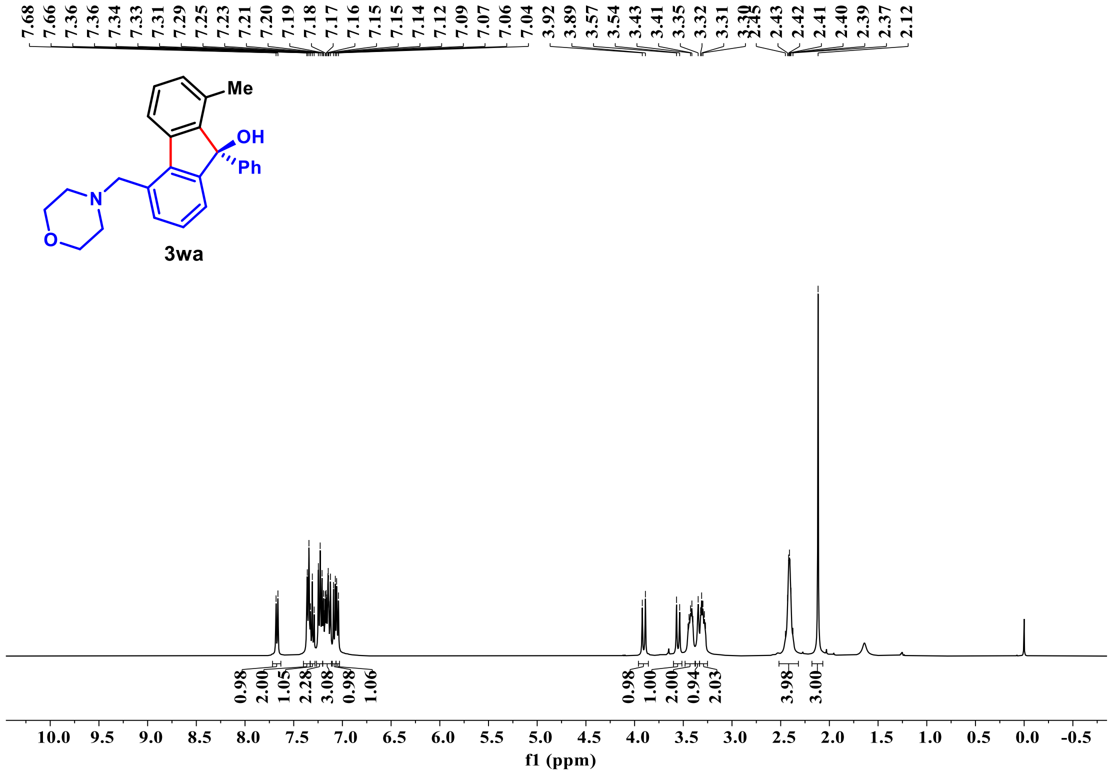

^{13}C NMR (100 MHz, $CDCl_3$)

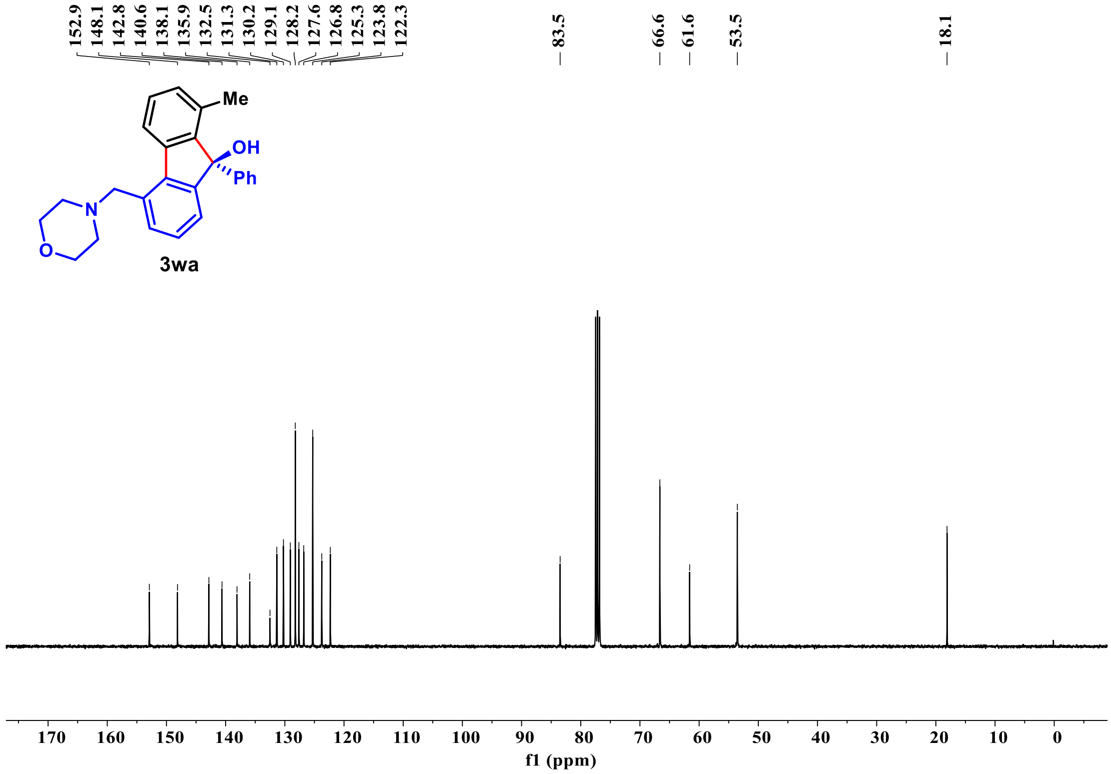

^{1}H NMR (400 MHz, CDCl$_3$)

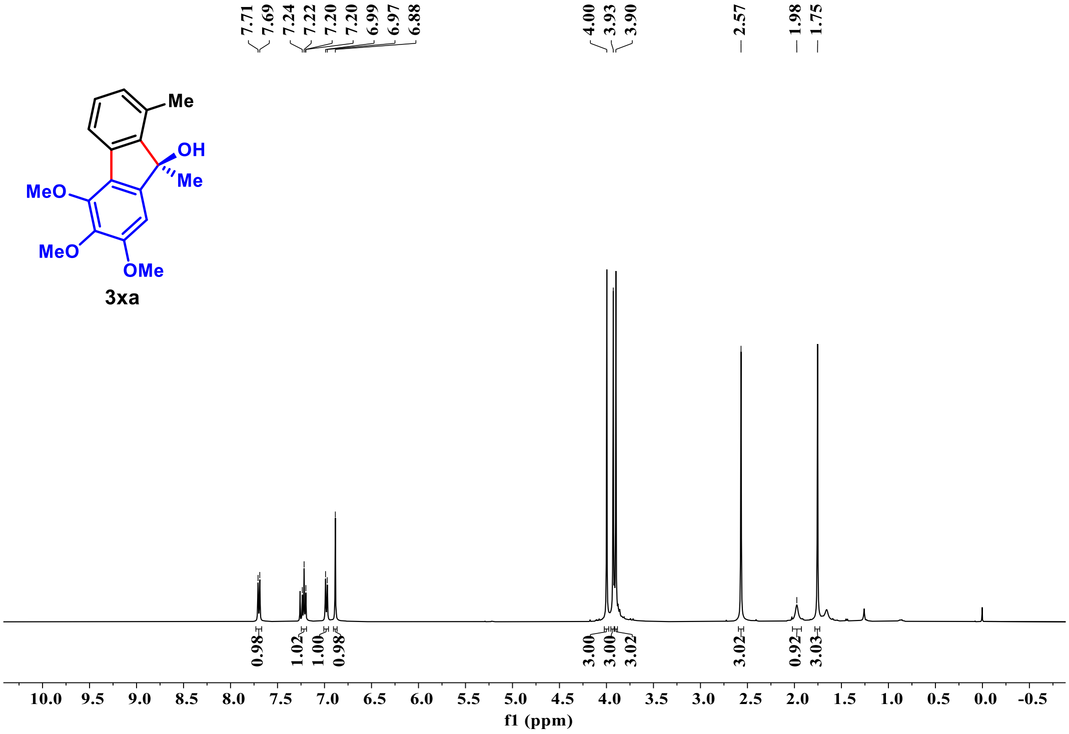

^{13}C NMR (100 MHz, CDCl$_3$)

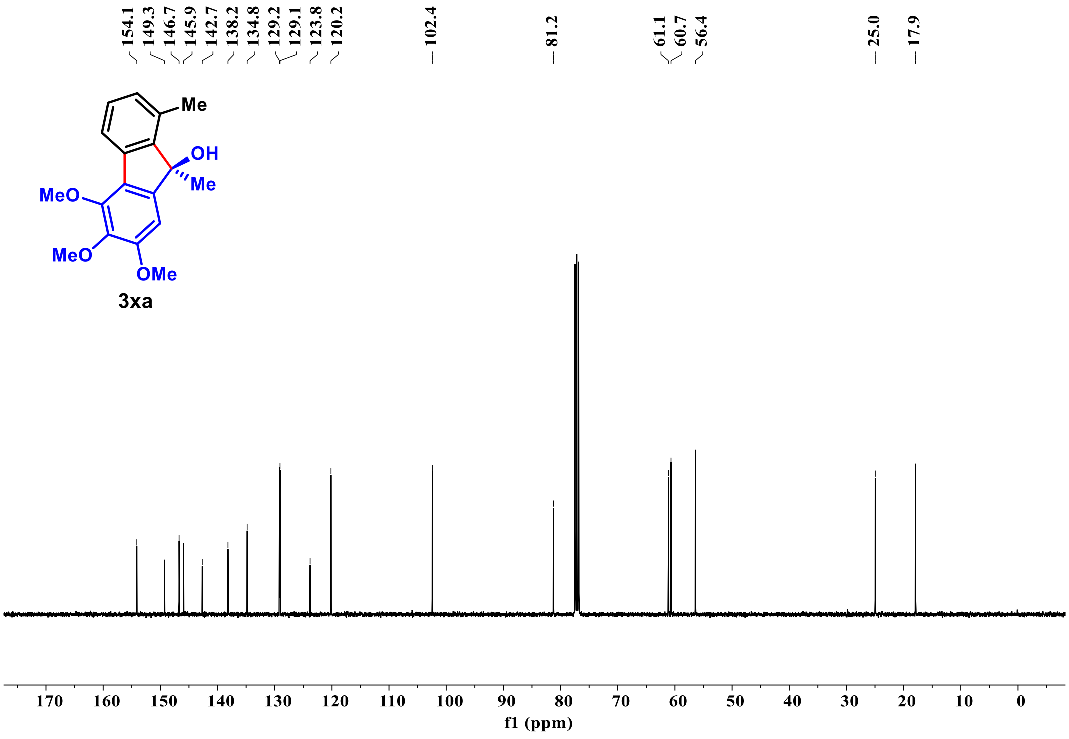

^{1}H NMR (400 MHz, $CDCl_3$)

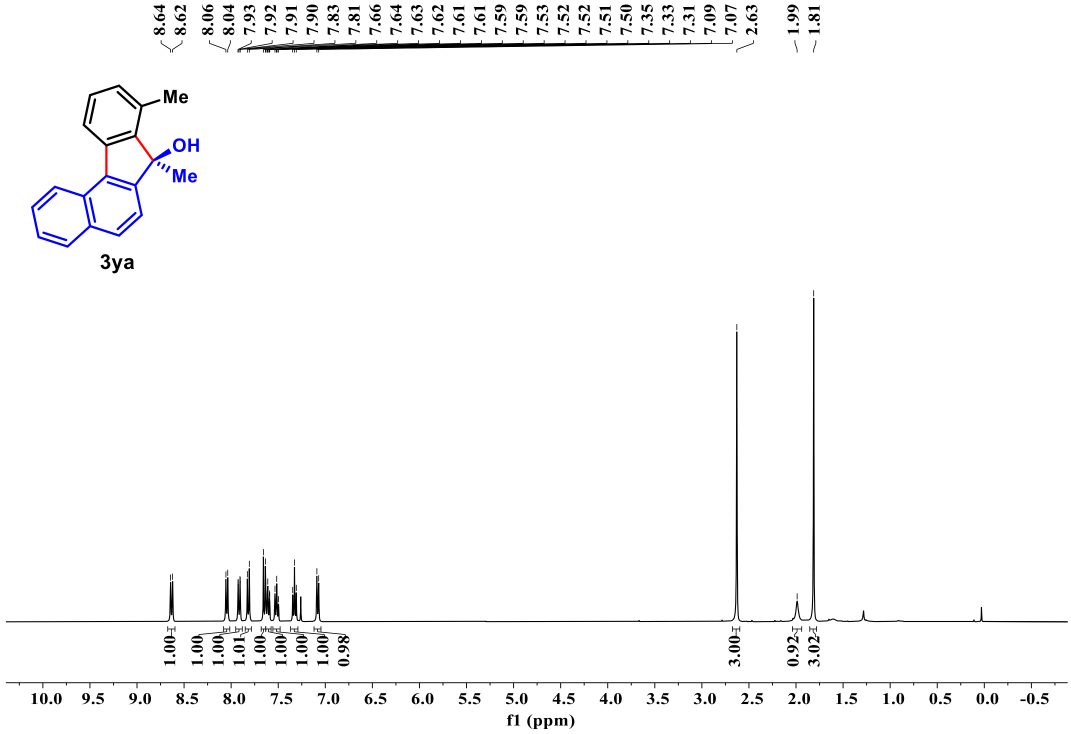

^{13}C NMR (100 MHz, $CDCl_3$)

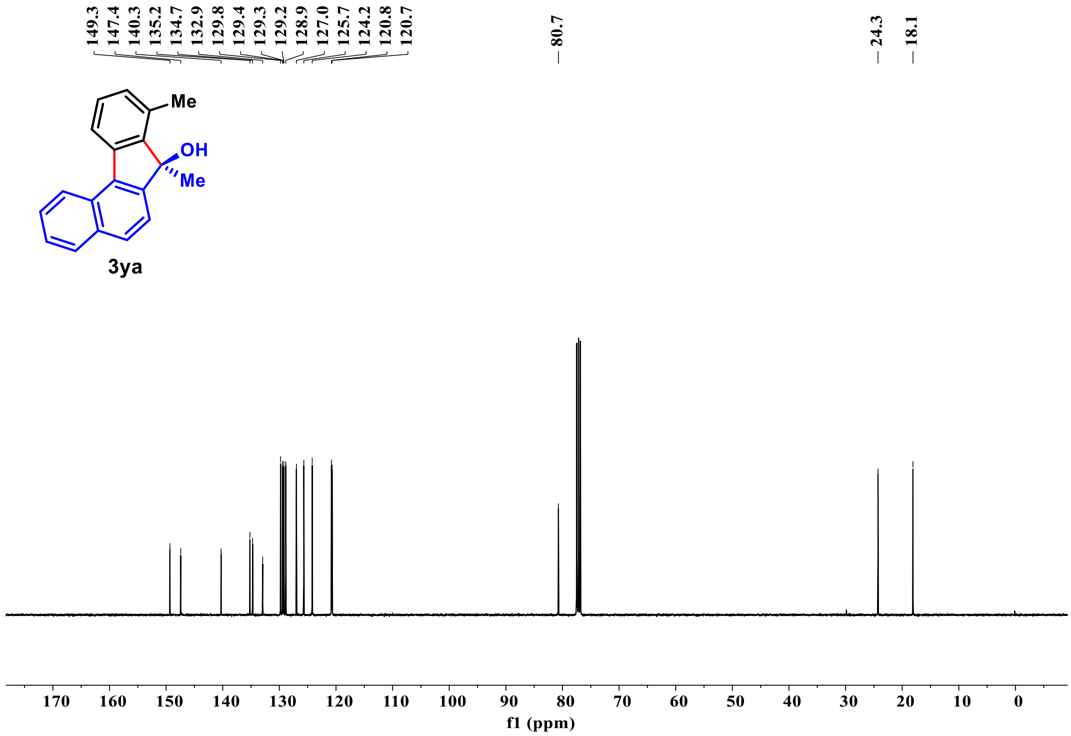

^{1}H NMR (400 MHz, MeOH-d_4)

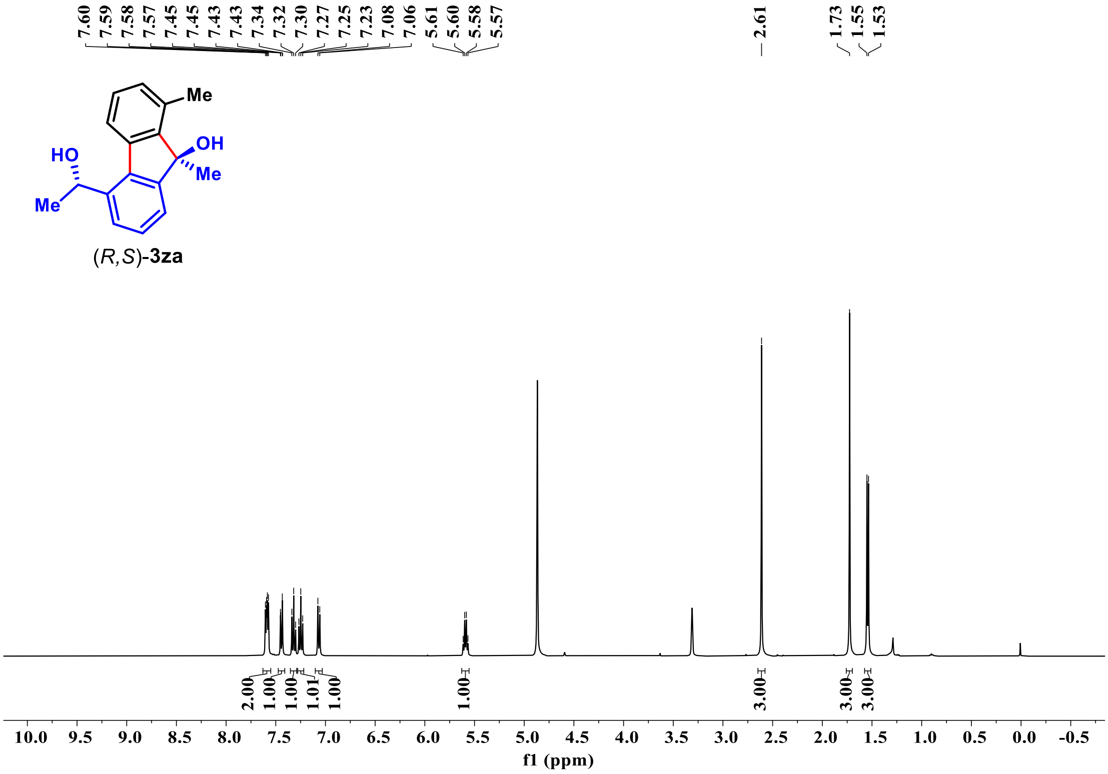

^{13}C NMR (100 MHz, MeOH-d_4)

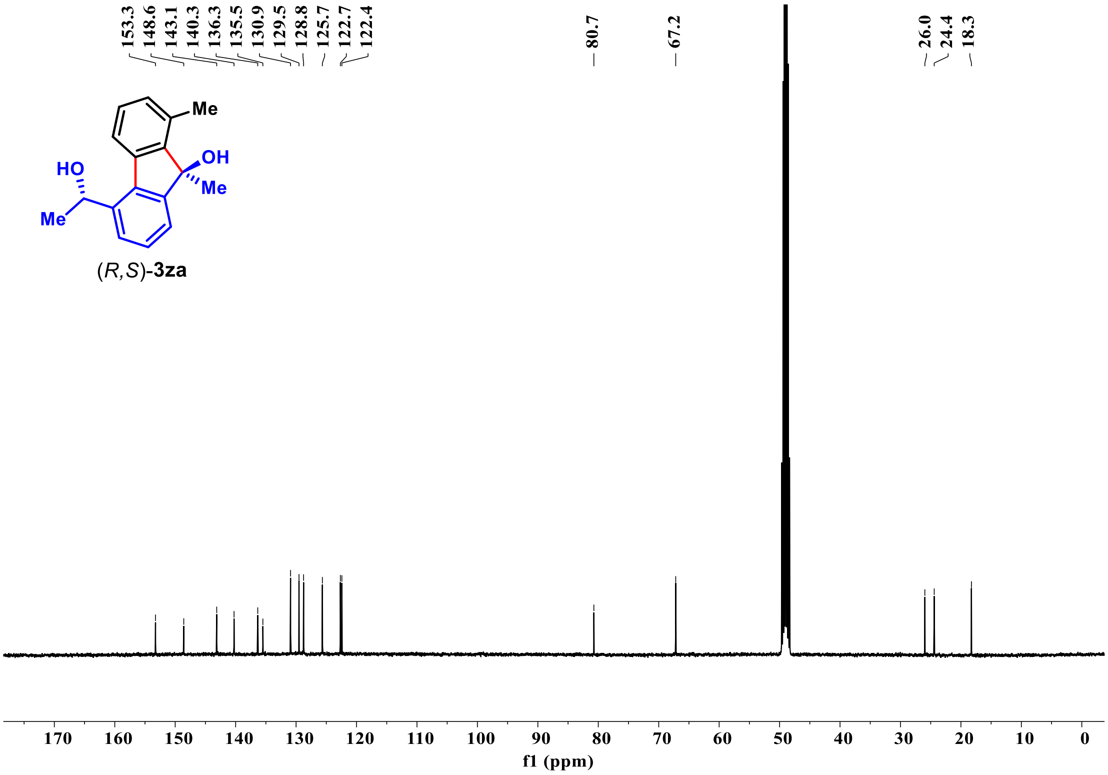

^{1}H NMR (400 MHz, MeOH-d_4)

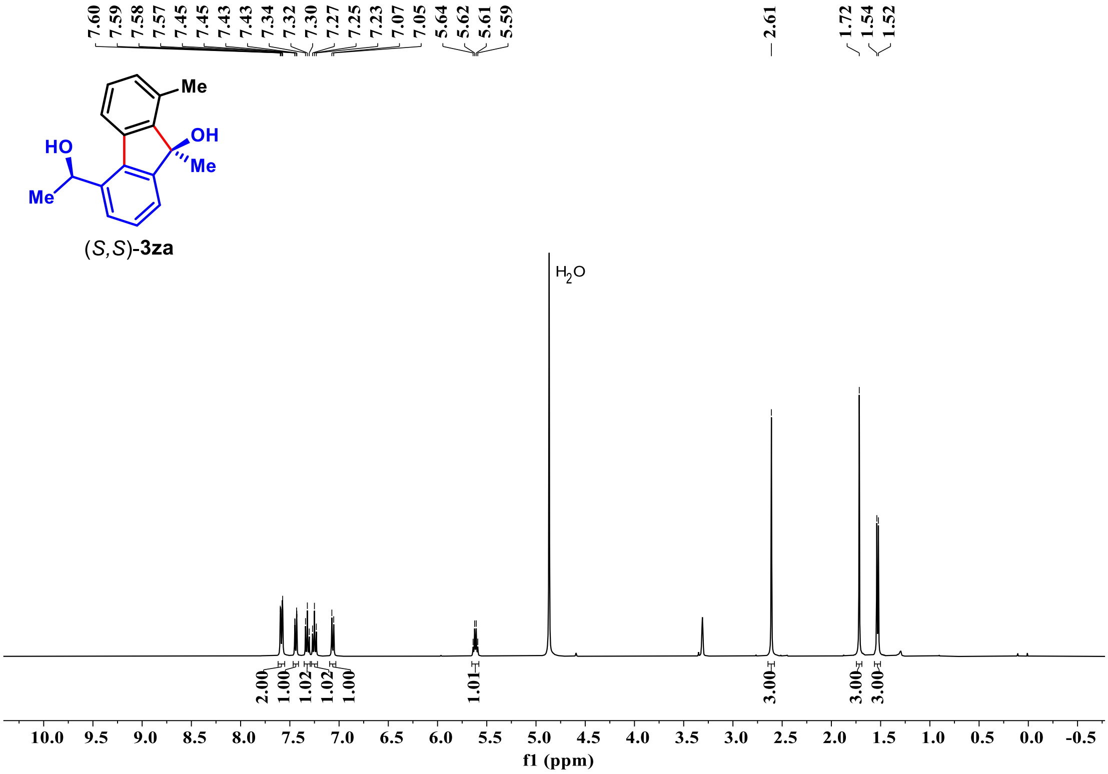

^{13}C NMR (100 MHz, MeOH-d_4)

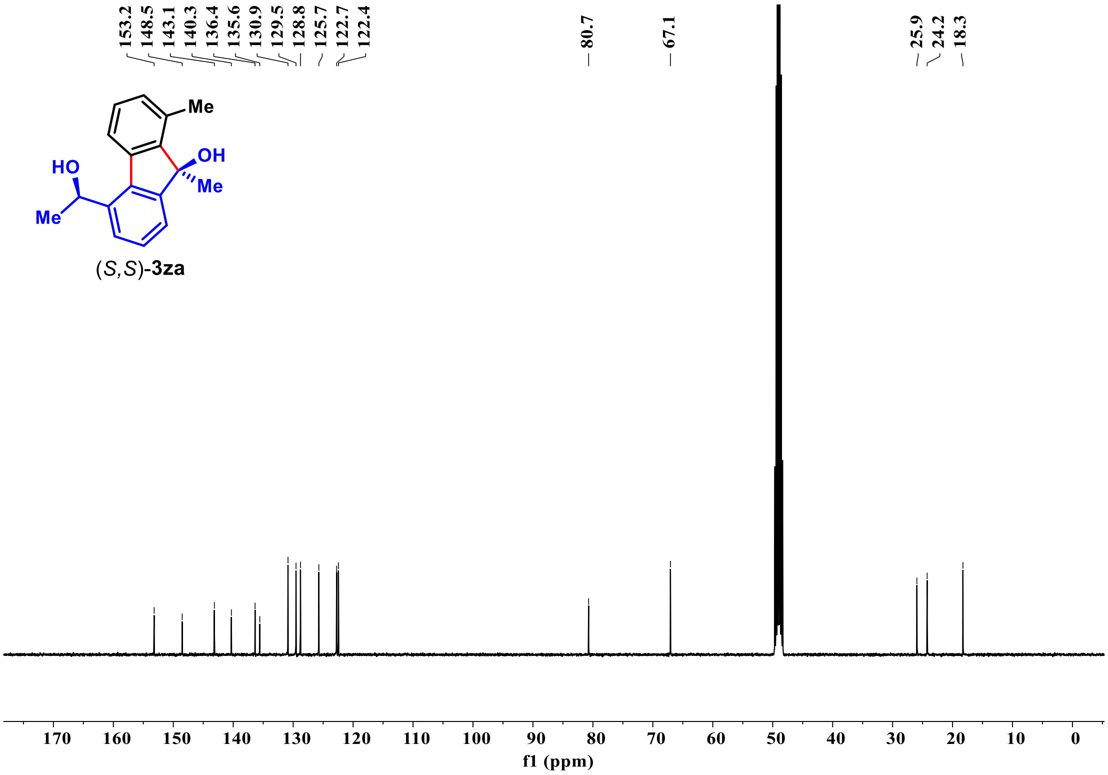

^{1}H NMR (400 MHz, $CDCl_3$)

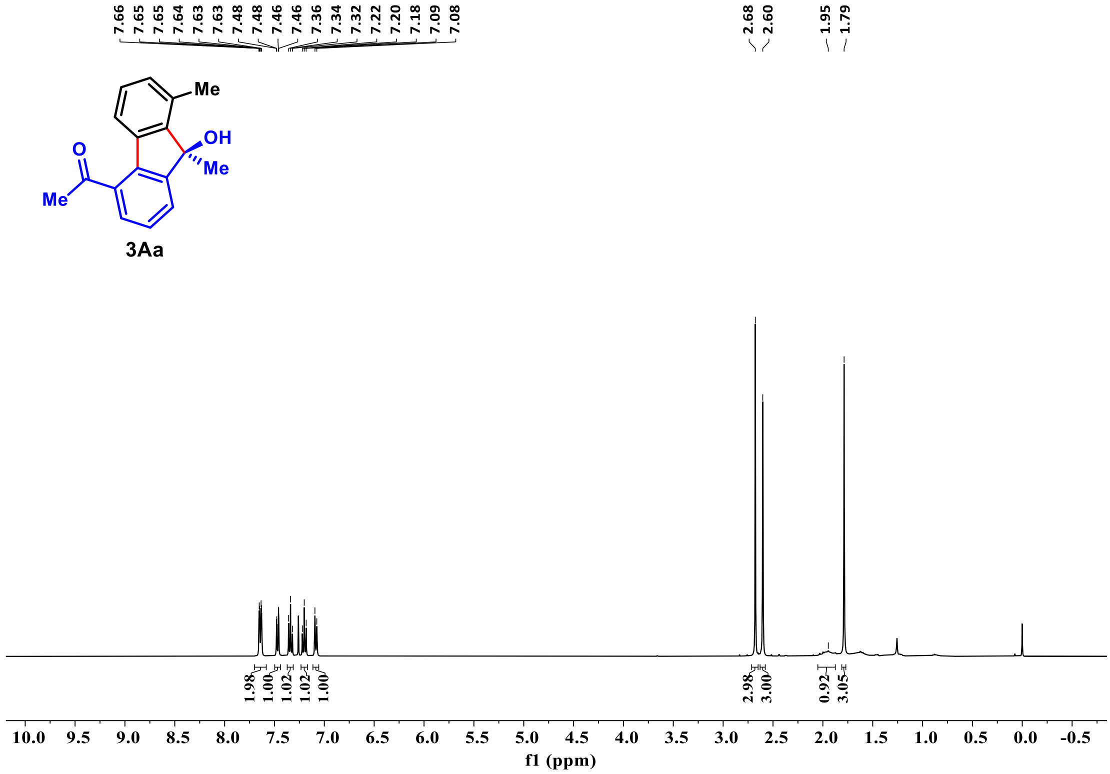

^{13}C NMR (100 MHz, $CDCl_3$)

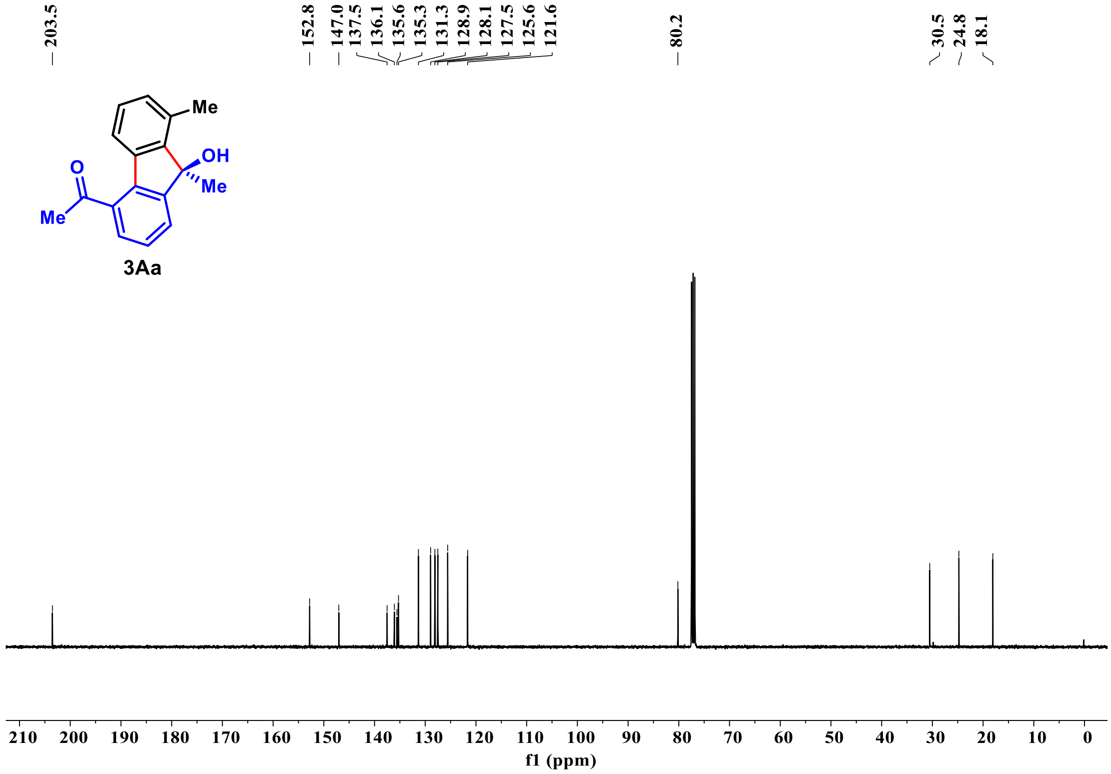

Supplement: SC-015-D4SC01004C-s001 [file SC-015-D4SC01004C-s001.pdf]
